# Organocatalytic Asymmetric Allylic Benzylborylation via Fluoride-Assisted Catalytic Generation of α-Boryl Carbanionic Intermediates

**DOI:** 10.1021/acs.orglett.4c03242

**Published:** 2024-09-20

**Authors:** Jordi Duran, Paula Rodríguez, Ward Vermeer, Xavier Companyó

**Affiliations:** Department of Inorganic and Organic Chemistry, Section of Organic Chemistry, University of Barcelona, carrer Martí i Franquès 1, 08028 Barcelona, Spain

## Abstract

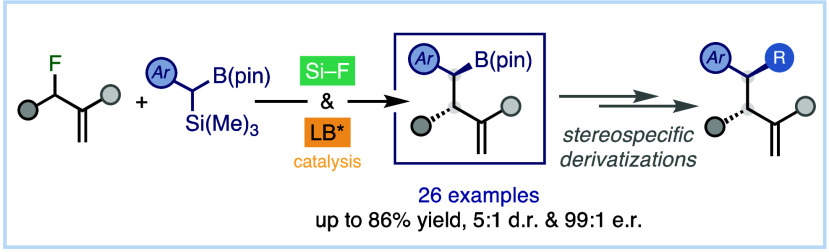

Herein we describe the organocatalytic asymmetric allylic benzylborylation of allyl fluorides with α-silyl benzylboronic esters. The catalytic protocol leverages the singular features of fluoride as an unconventional leaving group, enabling the catalytic generation of reactive α-boryl carbanion species through desilylative activation. It allows the construction of a wide set of homoallylic benzylated organoboronates bearing two contiguous stereocenters. The chiral boronate installed in the products serves as a synthetic lynchpin to construct complex chemical architectures in a stereospecific manner.

Chiral organoboronates are fundamental building blocks in modern organic chemistry.^1^ Besides their significant biological activities,^2^ enantioenriched organoboron compounds are key synthetic intermediates that can be further transformed into a variety of functional groups in stereoselective and stereospecific manner.^3^ As a result, enormous efforts have been made over the past few decades to discover new asymmetric C–B bond-forming.^4^ An alternative approach to synthesize enantioenriched organoboronate compounds relies on the use of α-boryl carbanions in C–C bond formation reactions.^5^ The empty p-orbital on the boron atom adjacent to the carbanion enhances the ability of such ionic species to participate in selective catalytic transformations.^6^ In this context, α-boryl organometallic nucleophiles have recently been implemented in transition-metal catalyzed asymmetric allylic alkylations (AAAs),^7,8^ one of the most versatile catalytic strategies for the stereoselective formation of C–C bonds.^9^ These transformations can be divided into deborylative^7^ and deprotonative^8^ methods according to the activation of the organoboron pronucleophile (Figure 1A,B). On the one hand, the alkoxide-promoted AAA with *gem*-diborylmethanes, catalyzed by either chiral copper or iridium complexes, proceeds via deborylation of the pronucleophile to afford homoallylic boronic esters with a single stereocenter (Figure 1A).^7^ On the other hand, the treatment of *gem*-diborylmethane or benzylboronates with a strong lithiated base, together with stoichiometric zinc salts under chiral iridium catalysis, enables the stereoselective construction of homoallylic organoboronates via deprotonative activation of the pronucleophile (Figure 1B).^8^ Either way, upon the effect of the stoichiometric Lewis or Brønsted base, the ensuing α-boryl carbanion is *in situ* transmetalated to form the corresponding α-boryl organometallic intermediate that subsequently undergoes transition-metal catalyzed AAA.

**Figure 1:**
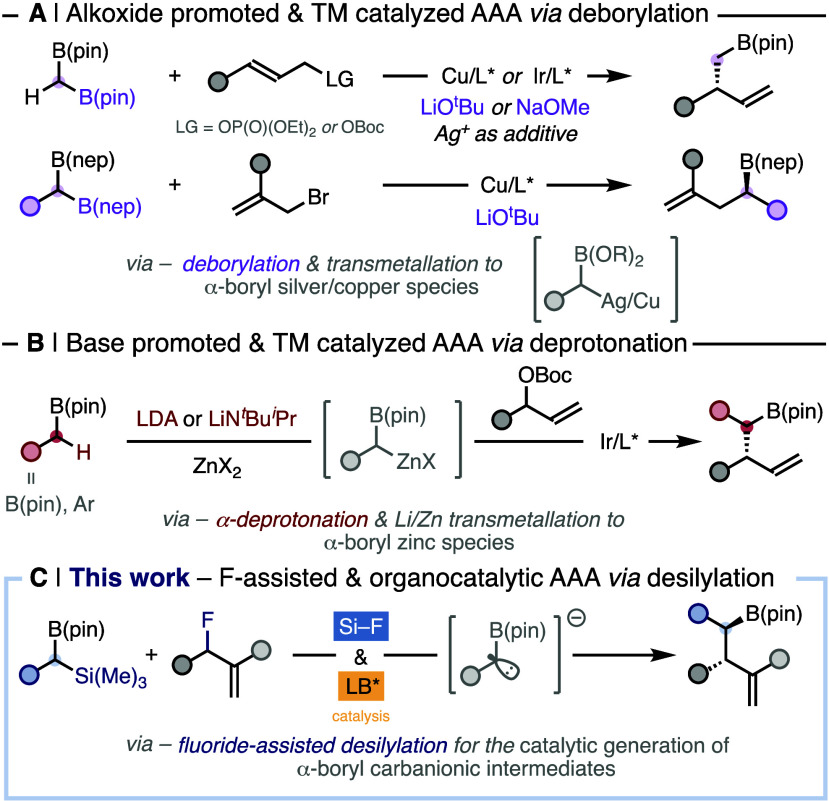
(A and B) Transition-metal catalyzed asymmetric allylic alkylborylations. (C) Organocatalytic asymmetric benzylborylation.

Allyl fluorides are emerging as alternative electrophiles in AAA reactions.^10^ The unique properties of fluoride when acting as a leaving group have allowed the discovery of novel reactivity pathways that would otherwise be unattainable.^11^

Herein we report the organocatalytic asymmetric construction of chiral homoallylic boronic esters via Lewis-base-catalyzed AAA of racemic allyl fluorides with racemic α-silyl benzylboronic esters (Figure 1C). The protocol harnesses the dual role of fluoride as an unconventional leaving group and as a borylated pronucleophile activator, thus avoiding the use of external stoichiometric Lewis and Brønsted bases. The catalyst-triggered, fluoride-assisted desilylative activation of the α-silyl boronates simultaneously generates an α-boryl carbanion in catalytic amounts together with an electrophilic chiral ammonium intermediate, that subsequently couple to forge the new C–C bond in a stereoselective manner. Therefore, the present protocol circumvents the transmetalation step to stabilize the α-boryl carbanion, as the nucleophilic intermediate is transiently generated upon electrophile activation. A wide range of homoallylic benzylated organoboron compounds bearing two contiguous stereocenters are formed in a regio-, diastereo- and enantiocontrolled fashion. In addition, the chiral boryl ester moiety serves as a synthetic lynchpin for the construction of biologically relevant, complex chemical scaffolds through a series of stereospecific product manipulations.

Initially, we focused on identifying a suitable α-boryl pronucleophile under racemic conditions (Table 1A).

**Table 1:**
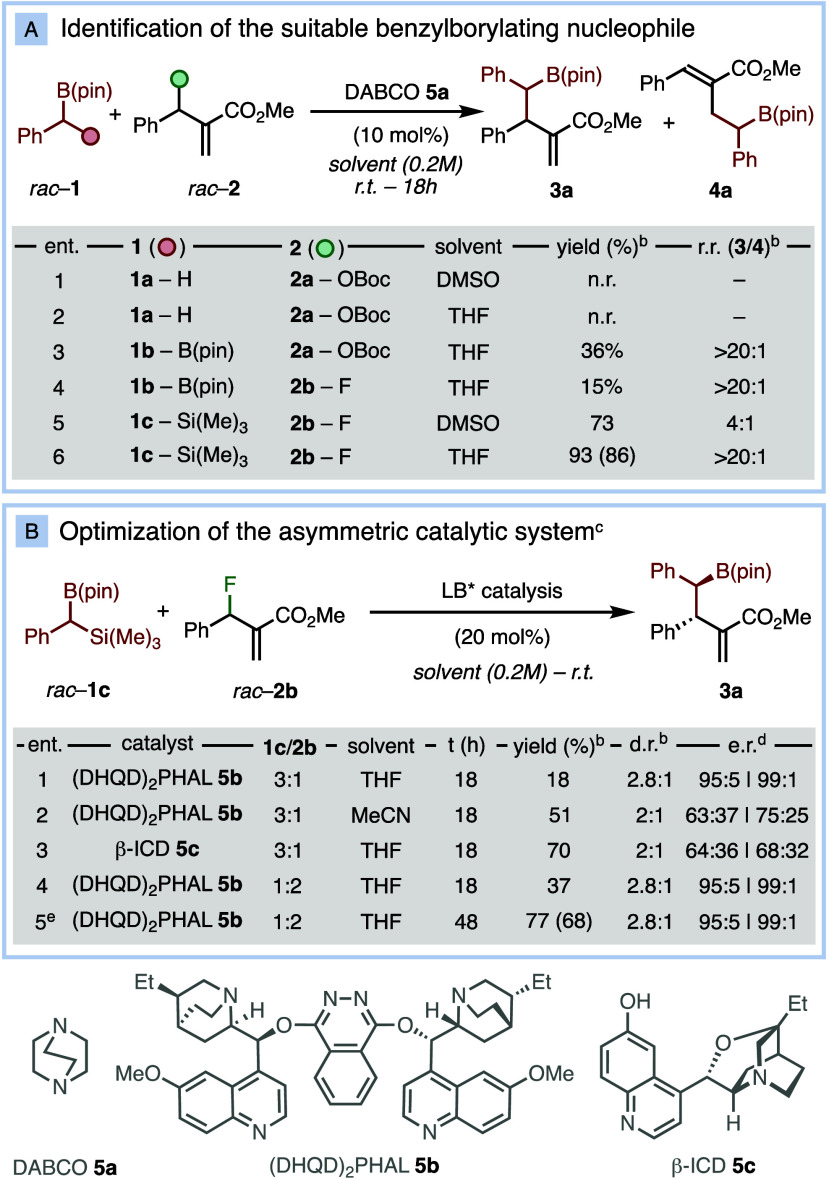
Reaction Design and Optimization^a^

a Selected results.

b Determined by ^1^H NMR using 1,3,5-trimetoxybenzene (in parentheses the isolated yield).

c Complete regiocontrol (>20:1 r.r.).

d Determined by chiral HPLC.

e Run at 0.4 M.

Benzylboronic ester **1a** was tested with allyl carbonate **2a** in polar aprotic solvents (Table 1A, entries 1,2). Upon ionization, *tert*-butoxide could partially deprotonate **1a** to form the α-boryl carbanion.^8^ However, no reaction was observed after 18 h. Subsequently we tested the reaction of benzyldiboronate **1b** with allyl carbonate **2a** (Table 1A, entry 3). In this case, the ionized *tert*-butoxide could activate the nucleophile via ate complex formation and deborylation.^7,12^ Although product **3a** was formed in 36% yield, full conversion of the starting material took place, suggesting its degradation under the reaction conditions. As *gem*-diboronates are known to be activated under the effect of a stoichiometric fluoride anion,^13^ we also investigated the reaction between **1b** and allyl fluoride **2b**, affording product **3a** in 15% yield (Table 1A, entry 4). At this point, we shifted our attention to the α-silyl benzyl boronic ester **1c**. Its reaction with allyl fluoride **2b** could generate the α-boryl carbanion via fluoride-assisted desilylation. In DMSO, the reaction between **1c** and **2b** afforded product **3a** in 73% yield as a 4:1 mixture of regioisomers (Table 1A, entry 5). By using a less polar solvent such as THF, product **3a** was isolated in 86% yield with excellent regiocontrol (>20:1, Table 1A, entry 6). Subsequently, we focused on optimizing the asymmetric catalytic transformation (Table 1B). The use of (DHQD)_2_PHAL **5b** as chiral Lewis-base in THF (Table 1B, entry 1) afforded product **3a** in 2.8:1 dr, excellent enantiocontrol for the two diastereoisomers albeit in a low 18% yield after 18 h. Other solvents and chiral Lewis bases produced the final products in higher yields but in lower stereoselectivities (Table 1B, entries 2–3).^14^ By using the allyl fluoride **2b** in excess (Table 1B, entry 4) and increasing the reaction concentration to 0.4 M, product **3a** was isolated in 68% yield as a single regioisomer (>20:1 r.r.), 2.8:1 d.r., 95:5 e.r. for the major diastereoisomer and 99:1 e.r. for the minor diastereoisomer after 48 h (Table 1B, entry 5). The two diastereosiomers are readily separated by flash chromatography, allowing the isolation of the highly enantioenriched *syn*- and the *anti-*homoallylic borylated adducts in the diastereopure form.

Using the optimized conditions, we next explored the generality of the catalytic transformation (Figure 2). We found that **1** can accommodate different halogenated groups at the *para* position of the aryl ring, affording products **3b**, **3c**, and **3d** in good yields, good diastereoselectivities (5:1 dr), and excellent enantioselectivities. Nucleophiles with an electron-rich aromatic ring are also compatible with the reaction conditions, including *para*-, *meta*- and *ortho*-methyl (**3f**-**3h**), *para*-methoxy (**3i**), 2-thienyl (**3j**) and β-naphthyl (**3k**) groups. The catalytic protocol is also amenable for the construction of product **3l** featuring a quaternary borylated stereogenic carbon adjacent to a tertiary stereocenter. Various allyl fluorides **2** with different electronic and steric properties are competent electrophiles, including electron-withdrawing (**3m**-**3r**, **3w** and **3x**) and electron-donating substituents (**3s**-**3v**). Furthermore, the ester residue can be successfully modified (**3y** and **3z**, Figure 2). The reaction can be scaled up to 1.0 mmol scale, consistently yielding product **3a** in excellent results (68% yield, 2.8:1 d.r., 95:5/99:1 e.r., Figure 2). The absolute configuration of the major diastereoisomer was determined by anomalous X-ray diffraction of product **3c**, while the absolute configuration of the minor diastereoisomer was established by chemical correlation (see Figure 3A, path *iv*).

**Figure 2:**
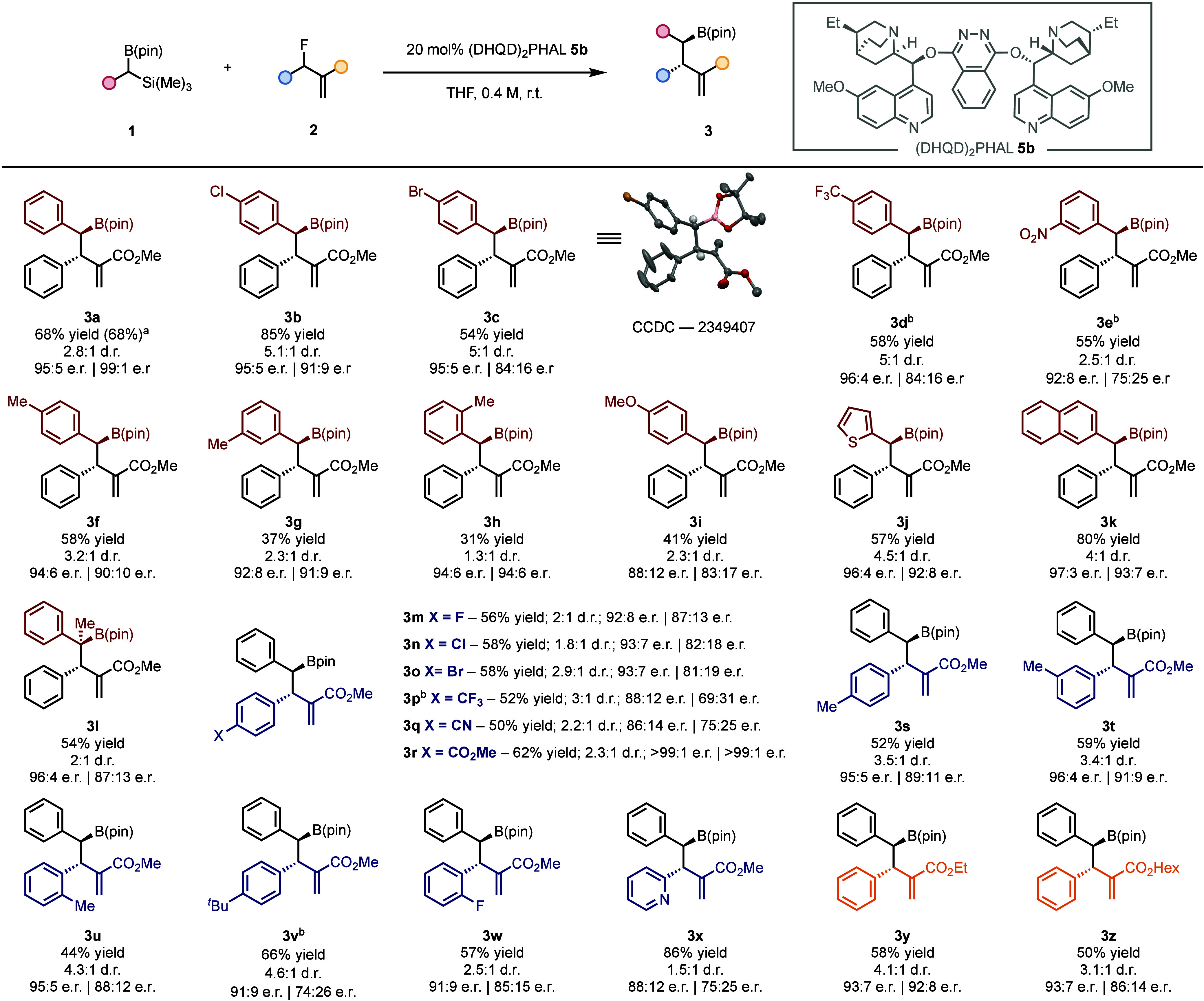
Reaction scope. General conditions: 0.2 mmol of **1**, 2.0 equiv of **2**, 0.5 mL of THF and 20 mol % of catalyst **5b** are stirred in a vial until full conversion. ^a^Performed on a 1.0 mmol scale. ^b^3.0 equiv of **2**.

**Figure 3:**
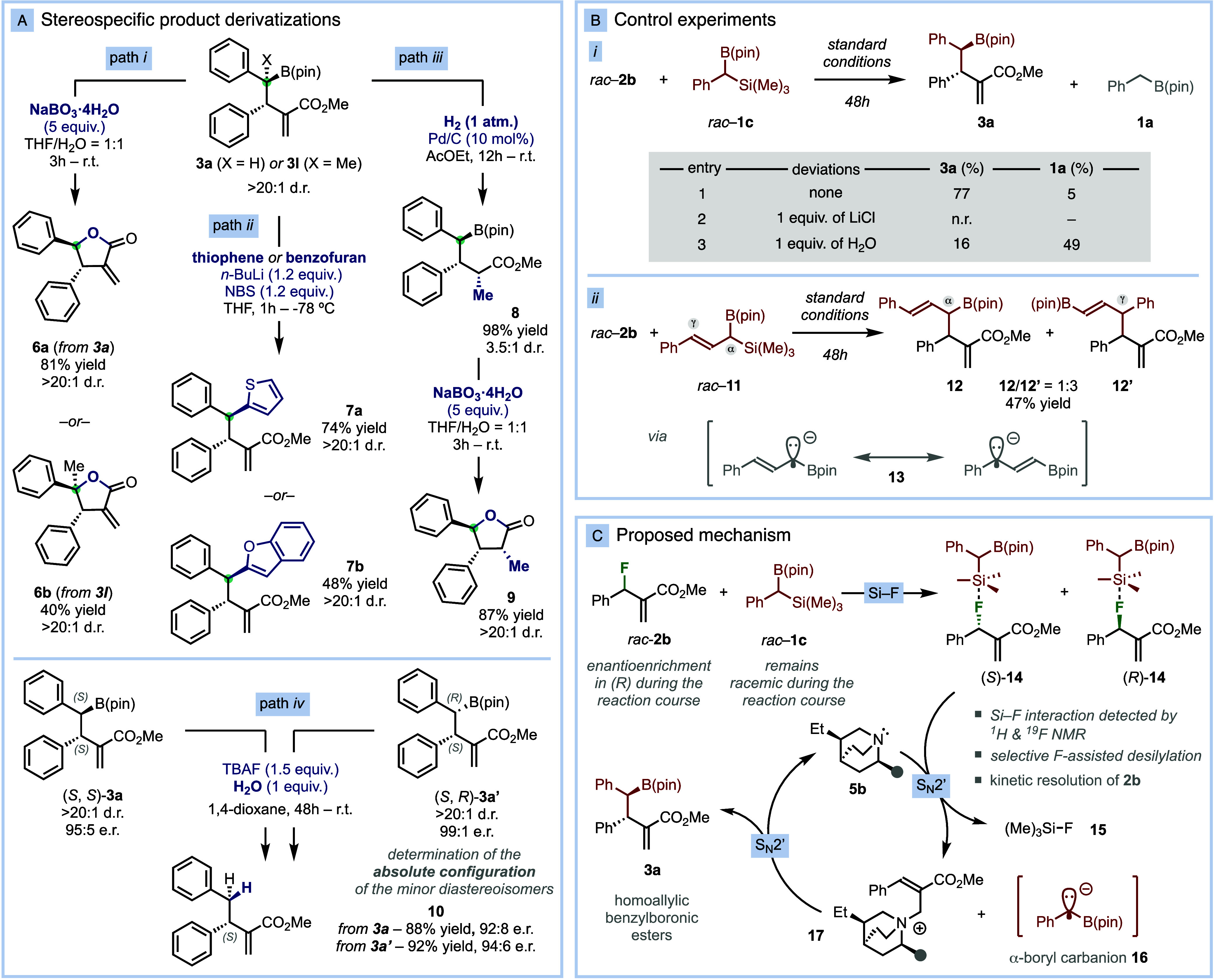
(A) Stereospecific product derivatizations to increase chemical complexity. (B) Control experiments. (C) Mechanistic proposal.

To enhance the versatility of the method, we developed a series of stereospecific product manipulations leveraging the singular chemical properties of the chiral boronic ester moiety (Figure 3A).

First, oxidation of the B–C bond of **3a** with sodium perborate^15^ led to the formation of the corresponding hydroxylated intermediate, which undergoes 5-*exo*-trig cyclization to yield the α-methylene-γ-butyrolactone **6a** in 81% yield and excellent stereospecificity (Figure 3A, path i). Oxidation/lactonization of adduct **3l** results in the formation of lactone **6b** bearing a quaternary stereocenter as a single diastereoisomer (Figure 3A, path i). α-Methylene-γ-butyrolactones embody an important structural motif found in bioactive natural products and pharmaceuticals.^16^ Second, treatment of **3a** with aryllithium species^17^ enables the stereospecific coupling with electron-rich aromatics, including thiophene (**7a**) and benzofuran (**7b**) (Figure 3A, path ii). Third, catalytic hydrogenation of the alkene moiety of **3a** affords the reduced product **8** in quantitative yield as a separable 3.5:1 mixture of diastereoisomers. Subsequent oxidation/lactonization of the major diastereoisomer of **8** forms lactone **9** bearing three contiguous stereocenters in 87% yield and diastereopure form (Figure 3A, path iii). Finally, fluoride-promoted protodeborylation^18^ of **3a** results in the formation of the product of formal asymmetric allylic benzylation (*S*)-**10** in 88% yield and 92:8 e.r. Protodeborylation of diastereoisomer **3a**′ also afforded product (*S*)-**10**, confirming the absolute configuration of the minor diastereoisomers as (*S*, *R*)-**3**′.

To gain insights into the reaction mechanism, we performed a series of control experiments, spectroscopic studies, and kinetic analysis.^14^ The α-silyl benzylboronic ester **1c** has the silicon and boron Lewis acid sites susceptible to react with fluoride.^19^ By *in situ*^1^H, ^11^B and ^19^F NMR, we confirmed that desilylation is the primary chemical process that takes place in a fast and irreversible manner upon treatment of **1c** with TBAF.^14^ NMR titration experiments between **2b** and **1c** showed a shift in both the ^19^F resonance of **2b** and the ^1^H signals of the trimethylsilyl moiety of **1c**, suggesting the formation of intermediate **14**.^14^ The addition of 1 equiv. of LiCl to the reaction completely suppressed reactivity (Figure 3Bi, entry 2). Given the ability of lithium ions to activate C–F bonds for nucleophilic displacement,^11g^ the presence of lithium in solution hampers the formation of intermediate **14** shutting down reactivity. Addition of water to the reaction mixture drastically reduced the yield of product **3a** in favor of the protodesilylated benzylboronic ester **1a** (Figure 3Bi, entry 3).

The reaction of α-silyl allyl boronic ester **11** with **2b** under standard reaction conditions affords a mixture of α- and γ- alkylated products **12** and **12′** (Figure 3Bii). These observations are consistent with the intermediacy of α-boryl carbanionic species such as **13** and **16**. In the presence of water **16** is protonated to form **1a**, while **13** can react through both mesomeric forms. Next, we monitored the kinetic and the stereochemical reaction profiles under optimized catalytic conditions.^14^ While racemic allyl fluoride **2b** undergoes kinetic resolution, resulting in steady enantioenrichment in the (*R*) form, **1c** remains racemic throughout the reaction course. Considering all this mechanistic information and the literature precedents,^11^ we propose that the organocatalytic asymmetric benzylborylation proceeds via two consecutive S_N_2′-S_N_2′ events, as depicted in Figure 3C. Initially, reagents **1c** and **2b** form an acid–base Lewis pair **14** via Si–F interaction. Subsequently, catalyst **5b** attacks (*S*)-**14** to generate ammonium intermediate **17** through S_N_2′ addition and simultaneously effect the fluoride-assisted desilylation of **1c** to form the α-boryl carbanion **16**. This step is responsible for the observed kinetic resolution of **2b**. Finally, a second S_N_2′ addition of species **16** to **17** forges product **3a** and regenerates catalyst **5b** for subsequent turnover.

In summary, we have developed a catalytic asymmetric methodology capable of constructing homoallylic benzylated organoboron compounds with two adjacent stereogenic carbons. The transformation relies on the catalyst-triggered, fluoride-assisted desilylative activation of the borylated pronucleophile, allowing the protocol to proceed under mild organocatalytic conditions without the need for stoichiometric strong bases and activating agents. In addition, the chiral boryl ester installed into the homoallylic borylated products serves as a versatile synthetic handle to construct complex chemical architectures in stereospecific manner.^20^

## Data Availability

The data underlying this study are available in the published article and its Supporting Information.

## Supporting Information Available

Experimental procedures, characterization data, mechanistic investigations, copies of the NMR spectra and HPLC traces (PDF)
