# Supplementary material for: Organocatalytic Asymmetric Allylic Benzylborylation via Fluoride-Assisted Catalytic Generation of α-Boryl Carbanionic Intermediates
Source: Org Lett. 2024 Sep 20;26(39):8394–9. doi: 10.1021/acs.orglett.4c03242 (PMC11459515; doi:10.1021/acs.orglett.4c03242)
Supplement: Supplementary file 1 — ol4c03242_si_001.pdf [file ol4c03242_si_001.pdf]

Supporting Information

Organocatalytic Asymmetric Allylic Benzylborylation *via* Fluoride-assisted Catalytic Generation of α-Boryl Carbanionic Intermediates

Jordi Duran, Paula Rodríguez, Ward Vermeer and Xavier Companyó*

Section of Organic Chemistry-Department of Inorganic and Organic Chemistry
University of Barcelona
carrer Martí i Franquès 1, 08028 Barcelona, Spain

E-mail: x.companyo@ub.edu

Table of Contents

A. General Information **4**

B. Synthesis of Starting Materials **5**

B.1. General Procedure A – Synthesis of Morita-Baylis-Hillman alcohols *5*

B.2. General Procedure B – Synthesis of allyl fluorides *6*

B.3. General Procedure C – Synthesis of Boroxines *7*

B.4. General Procedure D – Synthesis of α-silyl benzylboronic esters *8*

B.5. General Procedure E – Synthesis of the quaternary α-silyl benzylboronic ester 1l *9*

B.6. Synthesis of catalysts 5e, 5h and 5i *10*

C. Optimisation of the Racemic Allylic Benzylborylation **12**

C.1. Optimisation – Reactivity of allyl fluoride, carbonate and acetate with benzyl boronic acid pinacol ester *12*

C.2. Optimisation – Reactivity of allyl fluoride and carbonate with benzyldiboronate *13*

C.3. Optimisation – Reactivity of allyl fluoride, carbonate and acetate with α-silyl benzylboronic ester *14*

D. Allylic Benzylborylation of allyl fluorides with α-silyl benzylboronic esters – Racemic Reaction **15**

E. Optimisation of the Asymmetric Allylic Benzylborylation **16**

E.1. Optimisation – Catalyst screening *16*

E.2. Optimisation – Solvent screening *18*

E.3. Optimisation – Temperature, ratio of reagents and concentration *19*

F. Asymmetric Allylic Benzylborylation of allyl fluorides with α-silyl benzylboronic esters **20**

G. Mechanistic Studies **47**

G.1. Identification of the Si-F interaction – ^{19}F, ^{1}H and ^{11}B NMR Titrations *47*

G.2. Desilylation of 1c *50*

G.3. S_N2'–S_N2' Mechanism – Blank reactions *55*

G.4. Kinetic profiles – Kinetic resolution of 2b *62*

G.5. Kinetic profiles – Diastereochemical profile *63*

G.6. Control experiment – Study of the potential erosion of the diastereoselectivity promoted by the catalyst. *64*

H. Derivatisations **65**

H.1. Oxidation and cyclisation – Lactonisation *65*

H.2. Heteroaromatic substitution – Stereoospecific sp^2–sp^3 coupling of 3a with electronrich aromatics. *66*

H.3. Hydrogenation and lactonisation – Alkene reduction, oxidation and cyclisation *67*

H.4. Protodeborylation *70*

I. References **71**

J. Determination of the absolute configuration. X-ray crystallographic data of compound 3c **72**

K. NMR Spectra **85**

L. Chiral HPLC Chromatograms **199**

A. General Information

NMR experiments: NMR spectra (^{1}H, ^{13}C, ^{19}F and ^{11}B) were recorded on a Bruker Avance III HD 400 (400 MHz) spectrometer equipped with a CryoProbe™ Prodigy or a Bruker Avance Neo 500 (500 MHz) equipped with a broadband *i*Prob. The chemical shifts (δ) for ^{1}H and ^{13}C are given in ppm relative to residual signals of the solvents ($CHCl_3$, 7.26 ppm, ^{1}H NMR, 77.16 ppm ^{13}C NMR). Coupling constants are given in Hz. The following abbreviations are used to indicate the multiplicity: s, singlet; d, doublet; t, triplet; q, quartet; m, multiplet; bs, broad signal. NMR yields were calculated by using 1,3,5-trimethoxybenzene as internal standards.

High-Resolution Mass Spectra (HRMS): were obtained with an Agilent Technologies G1969A LC/MSD-TOF instrument using electrospray (ESI^{+})

Chiral High-Performance Liquid Chromatography (HPLC): analyses were performed on a Shimadzu LC-20AD instrument with DGU-20A5 and a SPD-20A UV/VIS detector or a Shimadzu LC-40D instrument with DGU-405 and a SPD-M40 UV/VIS detector using Phenomenex Lux® 5 µm Cellulose-1, Cellulose-2 or Amylose-1 LC Columns 254 x 4.6 mm in *n*-hexane/2-propanol mixtures.

Chiral Gas Chromatography (GC): analyses were performed on a Shimadzu GC-2010 instrument using an Agilent CP-Chirasil Dex CB GC Column 25 m x 0.32 mm x 0.25 µm-20 to 200/225 C.

Gas Chromatography (GC): analyses were performed on a Shimadzu GC-2010 Pro instrument using an Agilent DB-5 GC column 30 m x 0.250 mm x 0.25 µm-60 to 325/350 C.

Chromatographic purification of products was accomplished by flash chromatography in Sigma Aldrich silica gel 60 N (spherical, particle size 63−210 mm). Thin-layer chromatography (TLC) was carried out with TLC plates with silica gel 60 F254. Visualization of the developed chromatography was performed by checking UV absorbance (254nm) as well as with aqueous ceric ammonium molybdate and potassium permanganate solutions. Organic solutions were concentrated under reduced pressure on Büchi and IKA rotary evaporators.

Optical rotations were measured at 25 ºC in a Jasco P-2000 polarimeter employing a sodium lamp in $CHCl_3$.

Melting points were determined in a StuartTM melting point SMP3 instrument.

Materials: Commercial grade reagents and solvents were purchased at the highest commercial quality from Sigma Aldrich, Apollo Scientific, TCI, Fluorochem and BLD Pharma and used as received, unless otherwise stated. Catalysts **5a**, **5b**, **5c**, **5d**, **5f**, **5g** and **5j** were commercially available from Sigma-Aldrich. Catalysts **5e**,[5b] **5h**[5d] and **5i**[5e] have been synthesized following reported procedures.

B. Synthesis of Starting Materials

B.1. General Procedure A – *Synthesis of Morita-Baylis-Hillman alcohols*

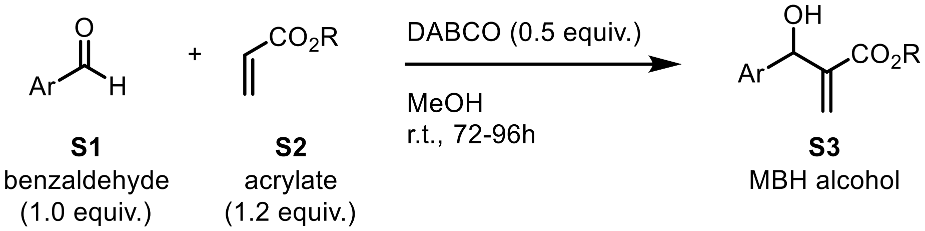

The reaction was carried out following the previously described procedure.[1a]

To a round bottom flask equipped with a magnetic stirring bar, MeOH (0.75 equiv., 37.5 mmol), the corresponding benzaldehyde **S1** (1.0 equiv., 50 mmol) and acrylate **S2** (1.2 equiv., 60 mmol) were added at room temperature. Then, DABCO **5a** (0.5 equiv., 25 mmol) was added, and the solution was stirred for 72-96 h until full consumption of the aldehyde, determined by TLC analysis. The crude reaction mixture was purified by flash chromatography on silica gel using mixtures of hexane/ethyl acetate as eluent to obtain alcohols **S3** in pure form.

All the spectroscopic data of the Morita-Baylis-Hillman alcohols match with the previously reported data.[1]

B.2. General Procedure B – *Synthesis of allyl fluorides*

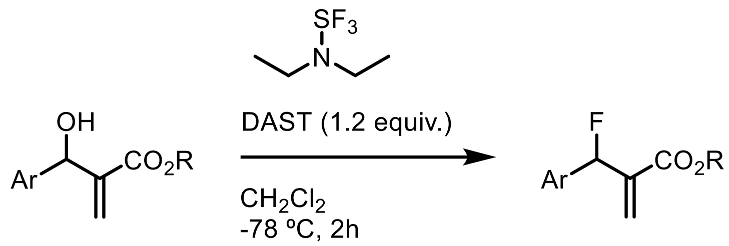

The reaction was carried out following the previously described procedure.[2a]

Into a two-necked round-bottom flask equipped with a magnetic stirring bar, the corresponding MBH alcohol **S3** (1.0 equiv., 15 mmol) was dissolved in dry CH_2Cl_2 (0.33 M) at -78 °C under nitrogen atmosphere. To this solution, DAST (1.2 equiv., 18 mmol) was added dropwise. The reaction mixture was stirred for 2 h at -78 °C and then quenched with saturated $NaHCO_3$ aqueous solution. The aqueous layer was extracted with CH_2Cl_2 (3x50 mL). The combined organic layers were dried over anhydrous $MgSO_4$ and concentrated under reduced pressure. The crude product **2** was purified by flash chromatography on silica gel using mixtures of hexane/CH_2Cl_2 to obtain allyl fluorides **2** in pure form.

All the spectroscopic data of the allyl fluorides match with the previously reported data.[2]

B.3. General Procedure C – *Synthesis of Boroxines*

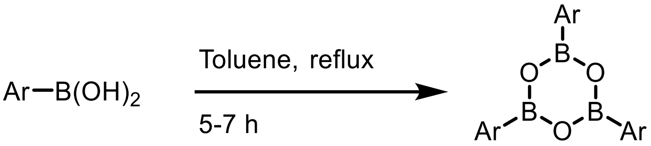

S4
Aryl boronic acid

S5
Boroxine

The reaction was carried out following the previously described procedure.[3a]

In a round bottom flask equipped with a magnetic stirring bar, boronic acid derivative **S4** (5 mmol) was dissolved in toluene (10 mL, 0.5 M) and refluxed with an oil bath for 5-7 h using a Dean-Stark apparatus. Subsequently, the solvent was removed under reduced pressure and the residue was dried overnight at 60 ºC under high vacuum obtaining the corresponding boroxine. The boroxine **S5** was directly used in the next step without further purification.

All the spectroscopic data of the boroxines match with the previously reported data.[3a,b]

***B.4. General Procedure D** – Synthesis of α-silyl benzylboronic esters*

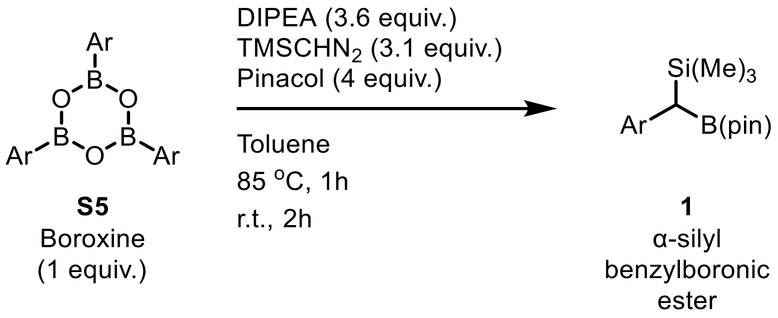

The reaction was carried out following the previously described procedure.[3a]

(Trimethylsilyl)diazomethane (3.1 equiv., 4.65 mmol, 2 M in hexane) was added to a mixture of boroxine **S5** (1.0 equiv., 1,5 mmol) and *N*,*N*-diisopropylethylamine (DIPEA) (3.6 equiv., 5.4 mmol) in toluene (0.2 M) under argon atmosphere . The reaction mixture was stirred at 85 ˚C with an oil bath for 1 h and allowed to cool down at room temperature. Then, pinacol (4.0 equiv., 6 mmol) was added and the reaction mixture was stirred at room temperature for 2 h. The reaction was quenched with saturated aqueous solution of NH_4Cl, and the aqueous phase was extracted with ethyl acetate. The combined organic layers were washed with brine, dried with $MgSO_4$ and concentrated in vacuo. The crude residue was purified by silica gel flash column chromatography on silica gel using mixtures of ethyl acetate/hexane to afford the α-silyl benzylboronic ester products **1**.

All the spectroscopic data of the α-silyl benzylboronic esters match with the previously reported data.[3a,c,d]

B.5. General Procedure E – Synthesis of the quaternary α-silyl benzylboronic ester ***1l***

Borylation of styrene S6

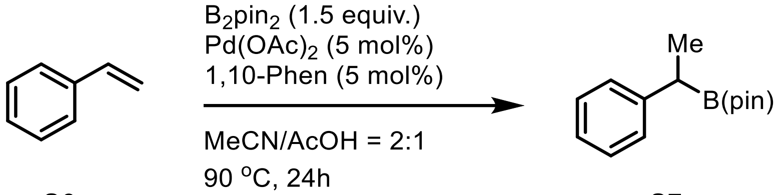

S6 Styrene (1 equiv.) **S7**

In a sealed Schlenk flask, $Pd(OAc)_2$ (108 mg, 0.05 equiv., 0.48 mmol), 1,10-phenantroline (86 mg, 0.05 equiv., 0.48 mmol), B_2pin_2 (3.66 g, 1.5 equiv., 14.4 mmol), MeCN/AcOH = 2:1 (50 mL) and styrene **S6** (1 g, 1 equiv., 9.6 mmol) were successively added and vigorously stirred under nitrogen atmosphere at 90 °C for 24 h. Subsequently, the mixture was cooled down to room temperature, neutralized with saturated $NaHCO_{3(aq)}$ solution and the resulting solution was extracted with AcOEt. The combined organic layers were washed with brine and dried over anhydrous $MgSO_4$. The solvent was removed under vacuum, and the crude product was purified by flash column chromatography using mixtures of hexane/ethyl acetate, to obtain product **S7** as a colourless oil in 51% yield.

Spectroscopic data of compound ***S7*** *match with those previously reported.*[4a]

Silylation of the tertiary pinacol boronic ester S7

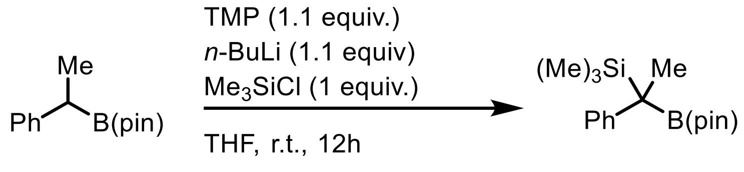

S7 (1.1 equiv.) **1l**

A solution of 2,2,6,6-tetramethylpiperidine (TMP) (367 µL, 1.1 equiv, 2.16 mmol) in THF (3.5 mL) was cooled to 0 °C, treated with *n*-BuLi (880 µL, 1.1 equiv, 2.5 M in hexane) dropwise, and stirred for 15 min. The freshly prepared LiTMP solution was added dropwise to a solution of **S7** (500 mg, 2.16 mmol, 1.1 equiv) in THF (3.5 mL) at 0 °C. After stirring at 0 °C for 30 min, the resulting solution was added dropwise to a solution of trimethylsilyl chloride (Me_3SiCl, 255 µL, 1 equiv, 2 mmol) in THF (3.5 mL) and stirred at room temperature for 12 h. The reaction was quenched with saturated aq. NH_4Cl solution and diluted with water and ethyl acetate. The aqueous phase was extracted with ethyl acetate 3 times. The combined organic layers were dried ($MgSO_4$) and concentrated in vacuo. The crude product was purified by flash chromatography on silica gel to afford product **1l** as a white solid in 45% yield.

Spectroscopic data of compound ***1l*** *match with those previously reported.*[4b]

B.6. Synthesis of catalysts 5e, 5h and 5i

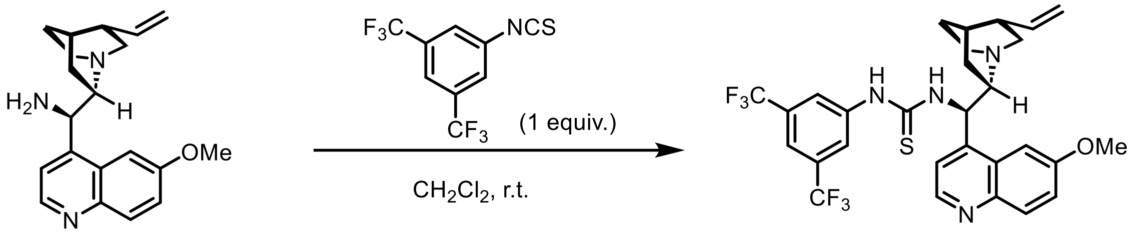

9-aminoquinine 5e

To a solution of 9-aminoquinine[5a] (1 mmol) in CH_2Cl_2 (5 mL) was slowly added to a solution of isothiocyanate or (1 mmol) in CH_2Cl_2 (5 mL). The mixture was stirred at room temperature until reaction completion. The pure product was obtained after purification by column chromatography on silica gel, affording the catalyst **5e** in 80% yield.

1-(3,5-bis(trifluoromethyl)phenyl)-3-((*R*)-((2*S*,4*S*,5*R*)-4-ethyl-1-methyl-5-vinylpiperidin-2-yl)(6-methoxyquinolin-4-yl)methyl)thiourea (5e)

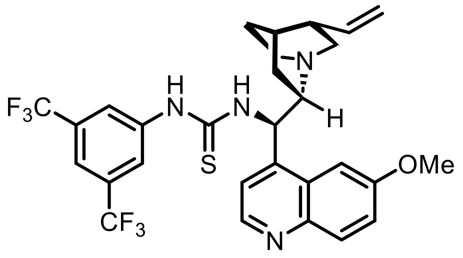

Spectroscopic data match with those previously reported.[5b]

^{1}H NMR (400 MHz, $CDCl_3$) δ 8.03 – 8.01 (m, 1H), 7.82 (brs, 3H), 7.69 – 7.66 (m, 2H), 7.40 (d, *J* = 8.6 Hz, 1H), 7.20 (brs, 1H), 5.72 – 5.70 (m, 1H), 5.03-5.00 (m, 2H), 3.97 (s, 3H), 3.58 (s, 1H), 3.39 (br s, 1H), 3.20 – 3.18 (brm, 1H), 2.79 – 2.72 (m, 2H), 2.35 – 2.32 (m, 1H), 1.73 (brs, 2H), 1.43 – 1.39 (m, 1H), 1.28 – 1.22 (m, 1H), 1.00 – 0.95 (m, 1H) ppm.

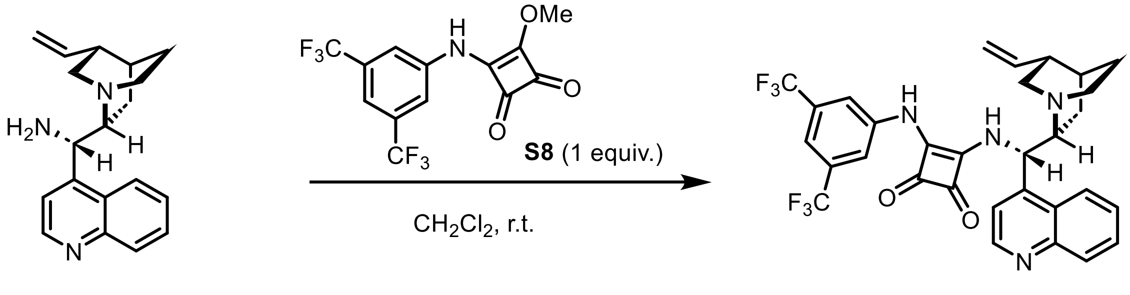

9-aminocinchonidine 5h

To a solution of 9-aminocinchonidine[5a] (1 mmol) in CH_2Cl_2 (5 mL) was slowly added to a solution of **S8**[5c] (1 mmol) in CH_2Cl_2 (5 mL). The mixture was stirred at room temperature until reaction completion. The pure product was obtained after purification by column chromatography on silica gel, affording the catalyst **5h** in 76% yield.

3-((3,5-bis(trifluoromethyl)phenyl)amino)-4-(((*S*)-quinolin-4-yl-((1*S*,2*S*,4*S*,5*R*)-5-vinylquinuclidin-2-yl)methyl)amino)cyclobut-3-ene-1,2-dione (5h)

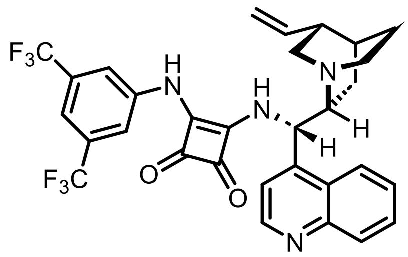

Spectroscopic data match with those previously reported.[5d]

^{1}H NMR (400 MHz, DMSO-d$_6$) δ 10.26 (s, 1H), 8.98 (d, *J* = 4.5 Hz, 1H), 8.45 (m, 2H), 8.09 (d, *J* = 8.0 Hz, 1H), 7.98 (s, 2H), 7.82 (m, 1H), 7.78 – 7.68 (m, 2H), 7.66 (s, 1H), 6.22 – 5.73 (m, 2H), 5.19 – 4.83 (m, 2H), 3.53 – 3.37 (m, 1H), 3.20 (m, 2H), 2.83 – 2.59 (m, 2H), 2.29 (s, 1H), 1.55 (d, *J* = 33.3 Hz, 3H), 1.38 (s, 1H), 0.74 (s, 1H).

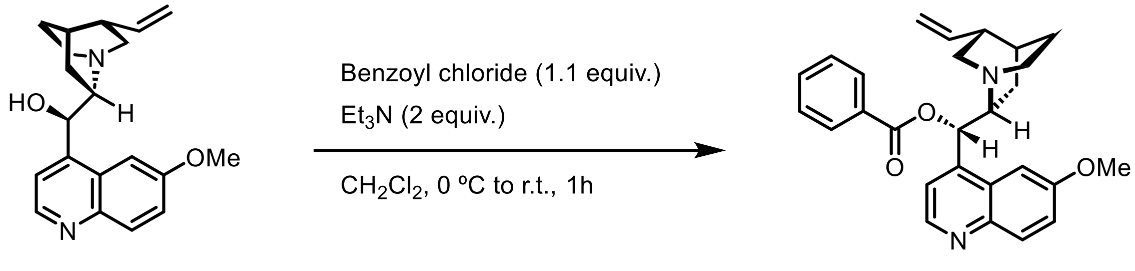

quinine **5i**

To a solution of quinine (1.0 mmol) and Et$_3$N (275 μL, 2.0 equiv., 2.0 mmol) in CH$_2$Cl$_2$ (0.1 M) were added benzoyl chloride (128 μL, 1.1 equiv., 1.1 mmol) at 0 °C and warmed up to room temperature. The reaction mixture was stirred for 1h and quenched with saturated aqueous solution of NaHCO$_3$. To the reaction mixture was added ethyl acetate and washed with saturated aqueous solution of NaHCO$_3$ and brine. The organic layer was dried over Na$_2$SO$_4$, concentrated in vacuo. The residue was purified by silica-gel column chromatography to give catalyst **5i** in 49% yield.

(1*R*)-(6-Methoxyquinolin-4-yl)(8-vinylquinuclidin-2-yl)methyl benzoate (5i)

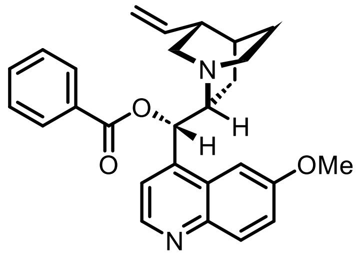

Spectroscopic data match with those previously reported.[5e]

^{1}H NMR (400 MHz, CDCl$_3$) δ 8.73 (d, *J* = 4.5 Hz, 1H), 8.12 – 8.10 (m, 2H), 8.03 (d, *J* = 9.2 Hz, 1H), 7.63 – 7.59 (m, 1H), 7.54 (d, *J* = 2.6 Hz, 1H), 7.51 – 7.47 (m, 2H), 7.43 (d, *J* = 4.5 Hz, 1H), 7.39 (dd, *J* = 9.2, 2.7 Hz, 1H), 6.81 (d, *J* = 6.1 Hz, 1H), 5.84 (ddd, *J* = 17.3, 10.2, 7.4 Hz, 1H), 5.06 – 4.99 (m, 2H), 3.98 (s, 3H), 3.54 – 3.49 (m, 1H), 3.26 (dddd, *J* = 16.4, 8.0, 5.6, 2.4 Hz, 1H), 3.14 (dd, *J* = 13.9, 10.2 Hz, 1H), 2.79 – 2.68 (m, 2H), 2.34 (br s, 1H), 1.9 6– 1.89 (m, 2H), 1.85–1.75 (m, 2H), 1.65 – 1.57 (m, 1H) ppm.

C. Optimisation of the Racemic Allylic Benzylborylation

C.1. ***Optimisation*** *– Reactivity of allyl fluoride, carbonate and acetate with benzyl boronic acid pinacol ester*

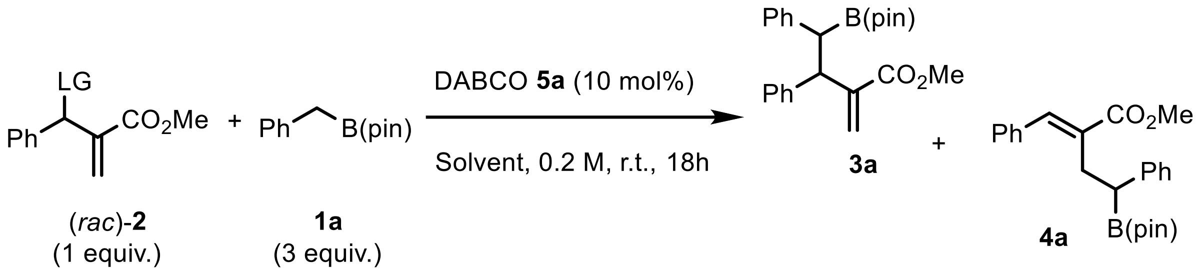

Entry	Solvent	Leaving Group (LG)	Conv. (%)	NMR yield (%)[a]	r.r.[a] (3a:4a)
**1**	DMSO	OBoc (**2a**)	-	n.d.	-
**2**	DMSO	F (**2b**)	-	n.d.	-
**3**	DMSO	OAc (**2c**)	-	n.d.	-
**4**	THF	OBoc (**2a**)	-	n.d.	-
**5**	THF	F (**2b**)	-	n.d.	-
**6**	THF	OAc (**2c**)	-	n.d.	-
**7**	CH_2Cl_2	OBoc (**2a**)	-	n.d.	-
**8**	CH_2Cl_2	F (**2b**)	-	n.d.	-
**9**	CH_2Cl_2	OAc (**2c**)	-	n.d.	-

Table S1. Reactivity of allyl fluoride and carbonates with benzyl boronic acid pinacol ester. [a] Determined by ^{1}H NMR spectroscopy using 1,3,5-trimethoxybenzene as the internal standard. n.d. = product not detected.

[The benzylborylation of allyl fluoride ***2b****, allyl carbonate* ***2a*** *and allyl acetate* ***2c*** *with benzyl boronic acid pinacol ester* ***1a*** *does not take place in any tested condition.]*

***C.2. Optimisation** – Reactivity of allyl fluoride and carbonate with benzyldiboronate*

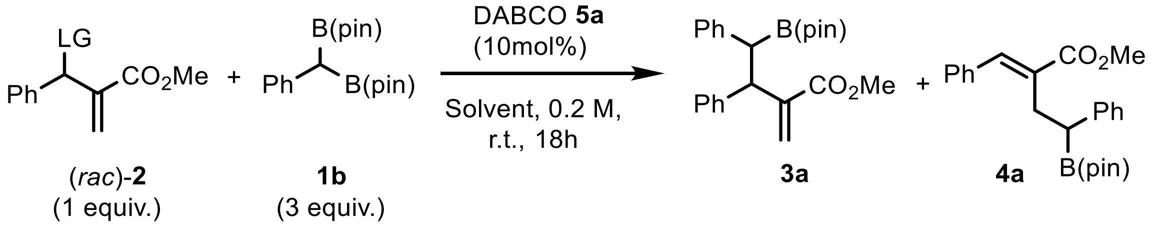

Entry	Solvent	Leaving Group (LG)	Conv. (%)	NMR yield (%)[a]	r.r.[a] (3a:4a)
**1**	CH_2Cl_2	OBoc (**2a**)	>99	25	>20:1
**2**	CH_2Cl_2	F (**2b**)	23	3	>20:1
**3**	THF	OBoc (**2a**)	>99	36	>20:1
**4**	THF	F (**2b**)	59	15	>20:1

Table S2. Reactivity of allyl fluoride with benzyldiboronate. [a]Determined by ^{1}H NMR spectroscopy using 1,3,5-trimethoxybenzene as the internal standard, [b]Determined by HPLC analysis using a chiral column.

*[The benzylborylation of allyl fluoride (**2b**) and allyl carbonate (**2a**) with benzyldiboronate **1b** proceeds in low yields]*

*C.3. **Optimisation** – Reactivity of allyl fluoride, carbonate and acetate with α-silyl benzylboronic ester*

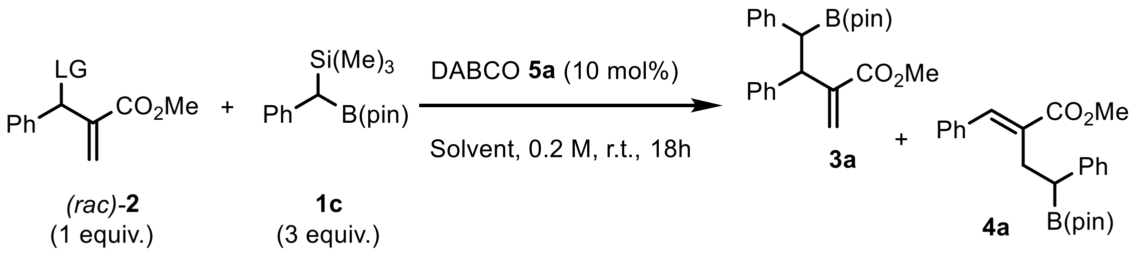

Entry	Solvent	Leaving Group (LG)	Conv. (%)	NMR yield (%)[a]	r.r.[a] (3a:4a)	d.r.[a]
**1**	DMSO	OBoc (**2a**)	-	n.d.	-	-
**2**	DMSO	OAc (**2c**)	-	n.d.	-	-
**3**	DMSO	F (**2b**)	90	73	4:1	1.8:1
**4**	CH_2Cl_2	F (**2b**)	92	80	>20:1	2:1
**5**	THF	F (**2b**)	>99	86	>20:1	2.2:1

Table S3. Reactivity of allyl fluoride and carbonate with α-silyl benzylboronic ester. [a]Determined by ^{1}H NMR spectroscopy using 1,3,5-trimethoxybenzene as the internal standard. n.d. = product not detected.

*[The allylic alkylation of allyl fluoride **2b** with α-silyl benzylboronic ester **1c** catalysed by DABCO proceed with complete regiocontrol in THF solution.]*

D. Allylic Benzylborylation of allyl fluorides with α-silyl benzylboronic esters – Racemic Reaction

General procedure

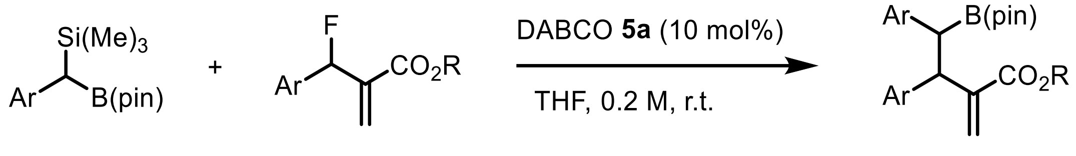

(rac)-**1** α-silyl benzylboronic esters (2.0 equiv.)

(*rac*)-**2** Allyl fluoride (1.0 equiv.)

3

The corresponding allyl fluoride **2** (1 equiv., 0.2 mmol) was weighted into a 5 mL vial equipped with a magnetic stirring bar, and dissolved with 1 mL of distilled THF (0.2 M). Subsequently, the corresponding α-silyl benzylboronic ester **1** (2 equiv., 0.4 mmol) and a 10 mol% of DABCO **5a** were successively added. The reaction mixture was stirred at room temperature until full consumption of the starting material was detected either by ^{1}H NMR. The crude product **3** was directly purified by flash column chromatography on silica gel using mixtures of *n*-hexane/ethyl acetate as eluent.

E. Optimisation of the Asymmetric Allylic Benzylborylation

E.1. Optimisation – Catalyst screening

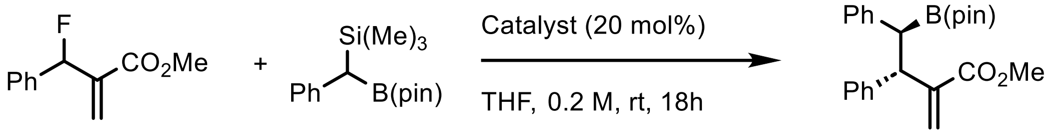

(rac)-**2b** (1 equiv.) *(rac)*-**1c** (3 equiv.) **3a**

Entry	Catalyst	NMR yield (%)[a]	r.r.[a]	d.r.[a]	e.r. (maj)[b]	e.r. (min)[b]
**1**	**$(DHQD)_2$PHAL 5b**	**18**	**>20:1**	**2.8:1**	**95:5**	**99:1**
**2**	β-isocupreidine **5c**	70[c]	>20:1	2.0:1	64:36	68:32
**3**	$(DHQD)_2$pyr **5d**	Traces	n.d.	n.d.	n.d.	n.d.
**4**	Thiourea quinine **5e**	Traces	n.d.	n.d.	n.d.	n.d.
**5**	$(DHQD)_2$AQN **5f**	4	>20:1	3.6:1	n.d.	n.d.
**6**	Quinine **5g**	6	>20:1	2.2:1	n.d.	n.d.
**7**	Squaramide cinchonidine **5h**	Traces	n.d.	n.d.	n.d.	n.d.
**8**	Benzoate quinine **5i**	Traces	n.d.	n.d.	n.d.	n.d.
**9**	$(DHQ)_2$AQN **5j**	Traces	n.d.	n.d.	n.d.	n.d.

Table S4. Optimisation of the chiral Lewis-base catalyst. [a] Determined by ^{1}H NMR spectroscopy using 1,3,5-trimethoxybenzene as the internal standard, [b] Determined by HPLC analysis using a chiral column. [c] Isolated yield.

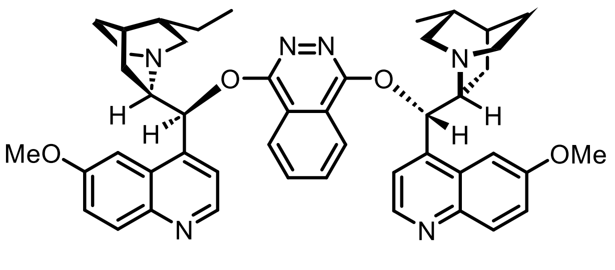

5b

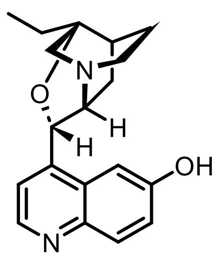

5c

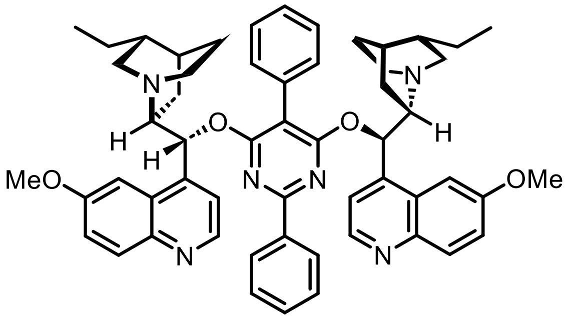

5d

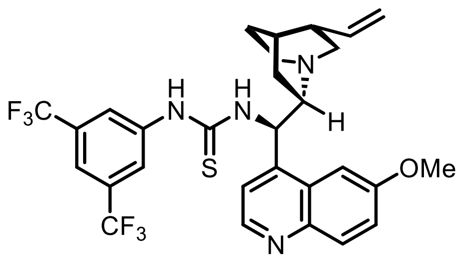

5e

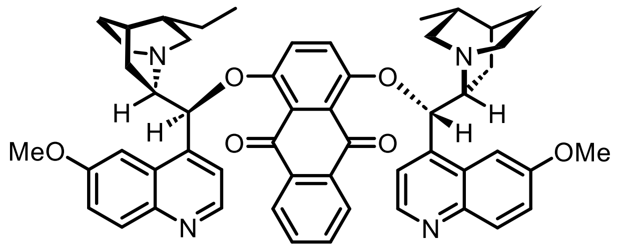

5f

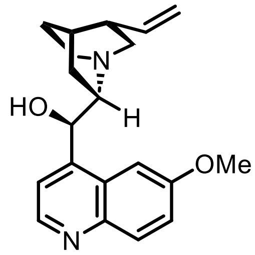

5g

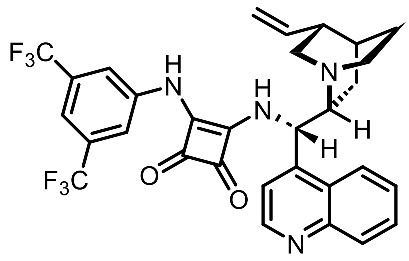

5h

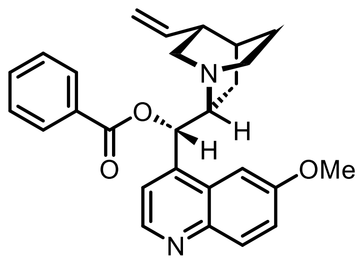

5i

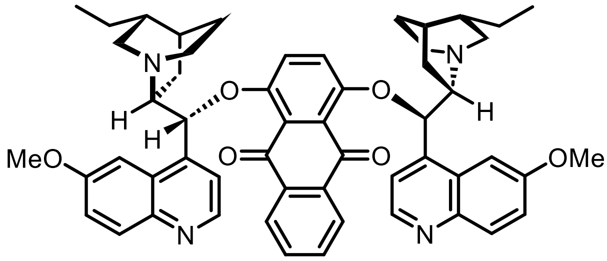

5j

Figure S1. Catalysts **5b-j**.

E.2. Optimisation – Solvent screening

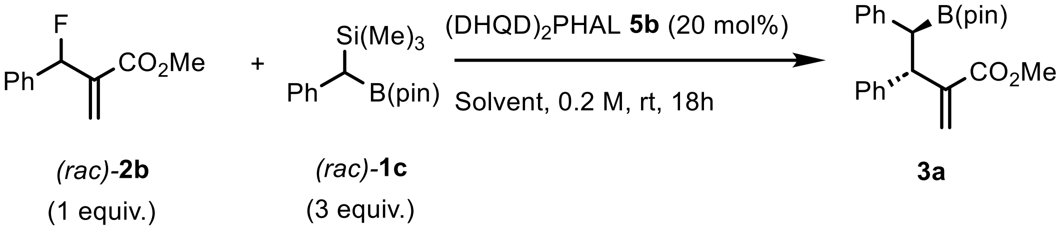

Entry	Solvent	NMR yield (%)[a]	r.r.[a]	d.r.[a]	e.r.[b]	e.r.[b]
**1**	**THF**	**18**	**>20:1**	**2.8:1**	**95:5**	**99:1**
**2**	Dioxane	3	>20:1	2.0:1	84:16	n.d.
**3**	Et_2O	traces	n.d.	n.d.	n.d.	n.d.
**4**	MeCN	51	>20:1	2.0:1	63:37	75:25
**5**	tBuOMe	traces	n.d.	n.d.	n.d.	n.d.
**6**	DMSO	16	1:2.0	2.8:1	n.d.	n.d.
**7**	DMF	14	1:1.9	3.0:1	n.d.	n.d.
**8**	2-Me-THF	2	>20:1	2.6:1	n.d.	n.d.
**9**	DCE	11	>20:1	2.4:1	97:3	67:33
**10**	CH_2Cl_2	10	>20:1	2.3:1	74:2	87:13
**11**	1,2-DME	13	>20:1	3.0:1	95:5	50:50
**12**	Toluene	traces	n.d.	n.d.	n.d.	n.d.
**13**	$CF_3C_6H_5$	traces	n.d.	n.d.	n.d.	n.d.
**14**	THF/CH_2Cl_2 = (9:1)	10	>20:1	2.4:1	n.d.	n.d.
**15**	THF/MeCN = (9:1)	24	>20:1	2.9:1	78:22	66:34

Table S5. Optimization of the solvent. [a] Determined by ^1H NMR spectroscopy using 1,3,5-trimethoxybenzene as the internal standard, [b] Determined by HPLC analysis using a chiral column. DCE: 1,2-dichloroethane, THF: tetrahydrofuran, ^{t}BuOMe: tert-butyl methyl ether, DMSO: dimethyl sulfoxide, DMF: *N*,*N*-Dimethylformamide, 1,2-DME: 1,2-dimethoxyethane.

*E.3. **Optimisation** – Temperature, ratio of reagents and concentration*

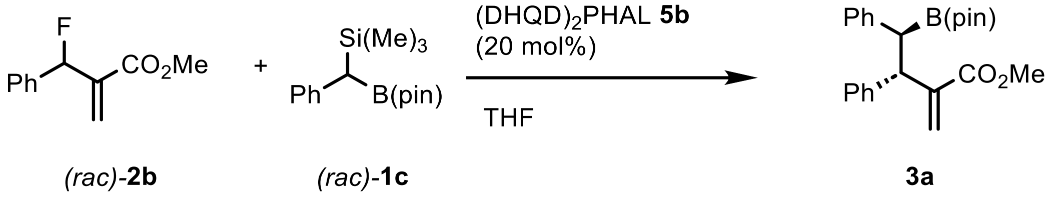

Entry	Ratio of reagents (2b:1c)	T (°C)	Conc. (M)	Time (h)	NMR yield (%)[a]	r.r.[a]	d.r.[a]	e.r.[b]	e.r.[b]
**1**	1:3	4	0,2	18	4	>20:1	2.8:1	n.d.	n.d.
**2**	1:3	25	0,2	18	18	>20:1	2.8:1	95:5	99:1
**3**	1:3	40	0,2	18	33	>20:1	2.0:1	92:8	90:10
**4**	1:1	25	0,2	18	9	>20:1	2.8:1	95:5	99:1
**5**	1:5	25	0,2	18	4	>20:1	2.8:1	n.d.	n.d.
**6**	**2:1**	**25**	**0,2**	**18**	**37**	>20:1	**2.8:1**	**95:5**	**99:1**
**7**	2:1	25	0,2	48	52	>20:1	2.8:1	95:5	99:1
**8**	2:1	25	0,3	48	63	>20:1	2.8:1	95:5	99:1
**9**	**2:1**	**25**	**0,4**	**48**	**77 (68)**[c]	>20:1	**2.8:1**	**95:5**	**99:1**

Table S6. Optimisation of the temperature, ratio of reagents and concentration. [a] Determined by ^{1}H NMR spectroscopy using 1,3,5-trimethoxybenzene as the internal standard, [b] Determined by HPLC analysis using a chiral column. [c] Isolated yield.

F. Asymmetric Allylic Benzylborylation of allyl fluorides with α-silyl benzylboronic esters

General Procedure

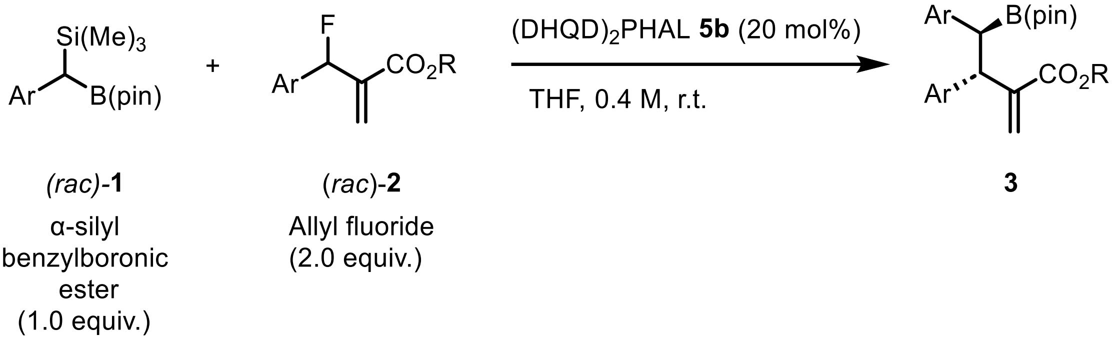

The corresponding allyl fluoride **2** (2 or 3 equiv., 0.4 or 0.6 mmol) was weighted into a 5 mL vial equipped with a magnetic stirring bar and dissolved with 1 mL of distilled THF (0.4 M). Subsequently, the corresponding α-silyl benzylboronic ester **1** (1 equiv., 0.2 mmol) and a 20 mol% of $(DHQD)_2PHAL$ **5b** were added. The reaction mixture was stirred at room temperature until full conversion of the starting material determined by ^1H NMR analysis. The crude product **3** was directly purified by flash column chromatography on silica gel using mixtures of *n*-hexane/ethyl acetate as eluent.

methyl 2-methylene-3,4-diphenyl-4-(4,4,5,5-tetramethyl-1,3,2-dioxaborolan-2-yl)butanoate

***3a** was prepared following the procedure described in section **F1**.*

68% yield (53 mg, 0.14 mmol) of a 2.8:1 diastereomeric ratio. The two diastereoisomers were readily separated by flash chromatography using *n*-hexane/ethyl acetate = 95:5. For the **1 mmol synthetic method example:** 68% yield (267 mg, 0.68 mmol).

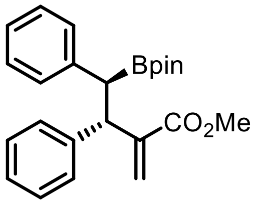

Major diastereomer (3*S*,4*S*)-3a, white solid. **^{1}H NMR** (400 MHz, $CDCl_3$) δ 7.11 – 6.93 (m, 10H), 6.37 (d, *J* = 0.6 Hz, 1H), 5.95 (dd, *J* = 1.2, 0.6 Hz, 1H), 4.37 (dd, *J* = 12.9, 1.0 Hz, 1H), 3.67 (s, 3H), 2.86 (d, *J* = 12.8 Hz, 1H), 1.18 (s, 6H), 1.12 (s, 6H) ppm; **^{13}C NMR** (101 MHz, $CDCl_3$) δ 167.3, 144.8, 141.9, 140.0, 129.3, 128.3, 128.1, 127.9, 126.1, 125.6, 123.7, 83.7, 52.0, 48.8, 38.5, 24.7, 24.5 ppm; **^{11}B NMR** (128 MHz, $CDCl_3$) δ 32.76 ppm; **HRMS (ESI)** Calculated for $[C_{24}H_{30}BO_4]^+$ ($[M+H]^+$) 393.2231. Found 393.2230; **mp** = 106–108; **$[\alpha]_D^{25}$** = +187.1 (c =2.21, $CHCl_3$); **HPLC** Phenomenex Lux Cellulose-1 (95:5 *n*-Hexane:2-Propanol, 1 mL/min, 254 nm); t_R (major) = 4.1 min, t_R (minor) = 4.6 min (95:5 e.r.).

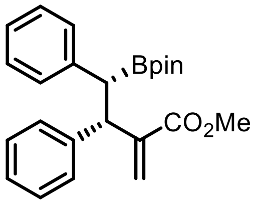

Minor diastereomer (3*S*,4*R*)-3a', white solid. **^{1}H NMR** (400 MHz, $CDCl_3$) δ 7.46 – 7.38 (m, 2H), 7.31 – 7.26 (m, 3H), 7.26 – 7.21 (m, 3H), 7.19 – 7.09 (m, 2H), 6.09 (d, *J* = 0.9 Hz, 1H), 5.63 (t, *J* = 0.9 Hz, 1H), 4.61 (d, *J* = 13.0 Hz, 1H), 3.57 (s, 3H), 3.17 (d, *J* = 13.0 Hz, 1H), 0.88 (s, 6H), 0.86 (s, 6H) ppm; **^{13}C NMR** (101 MHz, $CDCl_3$) δ 167.5, 142.8, 142.3, 140.4, 129.0, 128.8, 128.4, 128.3, 126.7, 125.6, 125.0, 83.4, 51.8, 47.7, 37.0, 24.4, 24.2 ppm; **^{11}B NMR** (128 MHz, $CDCl_3$) δ 31.53 ppm; **HRMS (ESI)** Calculated for $[C_{24}H_{29}BO_4Na]^+$ ($[M+Na]^+$) 415.2051. Found 415.2046; **mp** = 107–109; **$[\alpha]_D^{25}$** = +82.7 (c = 1.10, $CHCl_3$); **HPLC** Phenomenex Lux Cellulose-1 (95:5 *n*-Hexane:2-Propanol, 1 mL/min, 254 nm); t_R (major) = 7.6 min, t_R (minor) = 8.1 min (99:1 e.r.).

methyl 4-(4-chlorophenyl)-2-methylene-3-phenyl-4-(4,4,5,5-tetramethyl-1,3,2-dioxaborolan-2-yl)butanoate

***3b** was prepared following the procedure described in section **F1**.*

85% yield (67 mg, 0.15 mmol) of a 5.1:1 diastereomeric ratio. The two diastereoisomers were readily separated by flash chromatography using *n*-hexane/ethyl acetate = 95:5.

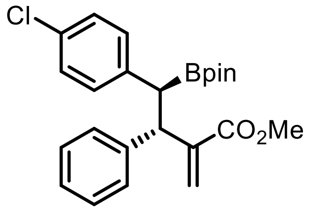

Major diastereomer (3*S*,4*S*)-3b, white solid. **^{1}H NMR** (500 MHz, $CDCl_3$) δ 7.09 – 6.96 (m, 9H), 6.37 (s, 1H), 5.94 (d, *J* = 1.1 Hz, 1H), 4.34 (dd, *J* = 12.9, 1.0 Hz, 1H), 3.67 (s, 3H), 2.86 (d, *J* = 12.9 Hz, 1H), 1.18 (s, 6H), 1.12 (s, 6H) ppm; **^{13}C NMR** (101 MHz, $CDCl_3$) δ 167.1, 144.5, 141.6, 138.6, 131.3, 130.5, 128.3, 128.2, 128.0, 126.2, 123.7, 83.8, 52.0, 48.7, 38.0, 24.7, 24.5 ppm; **^{11}B NMR** (128 MHz, $CDCl_3$) δ 32.82 ppm; **HRMS (ESI)** Calculated for $[C_{24}H_{29}BClO_4]^+$ ($[M+H]^+$) 427.1841. Found 427.1844; **mp** = 55–57; **$[\alpha]_D^{25}$** = +198.9 (c = 2.47, $CHCl_3$); **HPLC** Phenomenex Lux Cellulose-1 (98:2 *n*-Hexane:2-Propanol, 1 mL/min, 254 nm); t_R (major) = 4.8 min, t_R (minor) = 6.0 min (95:5 e.r.).

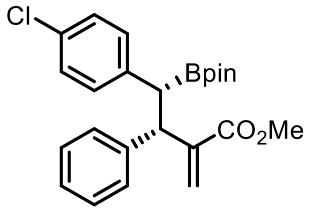

Minor diastereomer (3*S*,4*S*)-3b', white solid. **^{1}H NMR** (500 MHz, $CDCl_3$) δ 7.43 – 7.38 (m, 2H), 7.29 – 7.15 (m, 7H), 6.09 (d, *J* = 0.8 Hz, 1H), 5.60 (t, *J* = 0.9 Hz, 1H), 4.55 (d, *J* = 13.0 Hz, 1H), 3.57 (s, 3H), 3.16 (d, *J* = 12.9 Hz, 1H), 0.88 (s, 6H), 0.86 (s, 6H) ppm; **^{13}C NMR** (101 MHz, $CDCl_3$) δ 167.3, 142.4, 142.1, 139.1, 131.3, 130.1, 128.9, 128.6, 128.3, 126.8, 125.1, 83.6, 51.8, 47.9, 36.5, 24.4, 24.2 ppm; **^{11}B NMR** (128 MHz, $CDCl_3$) δ 32.25 ppm; **HRMS (ESI)** Calculated for $[C_{24}H_{29}BClO_4]^+$ ($[M+H]^+$) 427.1841. Found 427.1844; **mp** = 102–104; **$[\alpha]_D^{25}$** = +70.7 (c = 0.45, $CHCl_3$); **HPLC** Phenomenex Lux Cellulose-1 (98:2 *n*-Hexane:2-Propanol, 1 mL/min, 254 nm); t_R (major) = 5.0 min, t_R (minor) = 13.7 min (91:9 e.r.).

methyl 4-(4-bromophenyl)-2-methylene-3-phenyl-4-(4,4,5,5-tetramethyl-1,3,2-dioxaborolan-2-yl)butanoate

***3c** was prepared following the procedure described in section **F1**.*

54% yield (47 mg, 0.11 mmol) of a 5:1 diastereomeric ratio. The two diastereoisomers were readily separated by flash chromatography using *n*-hexane/ethyl acetate = 95:5.

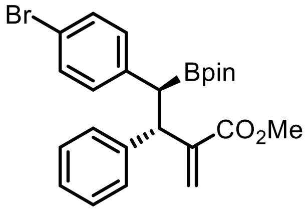

Major diastereomer (3*S*,4*S*)-3c, white solid. **^{1}H NMR** (400 MHz, $CDCl_3$) δ 7.20 (d, *J* = 8.44 Hz, 2H), 7.10 – 7.04 (m, 2H), 7.03 – 6.96 (m, 3H), 6.94 (d, *J* = 8.47 Hz, 2H), 6.37 (s, 1H), 5.94 (d, *J* = 1.10 Hz, 1H), 4.33 (dd, *J* = 12.83, 1.01 Hz, 1H), 3.67 (s, 3H), 2.85 (d, *J* = 12.88 Hz, 1H), 1.18 (s, 6H), 1.12 (s, 6H) ppm; **^{13}C NMR** (101 MHz, $CDCl_3$) δ 167.0, 144.5, 141.5, 139.2, 131.2, 130.9, 128.2, 128.0, 126.2, 123.7, 119.5, 83.8, 52.0, 48.6, 38.0, 24.7, 24.5 ppm; **^{11}B NMR** (128 MHz, $CDCl_3$) δ 32.92 ppm; **HRMS (ESI)** Calculated for $[C_{24}H_{29}BBrO_4]^+$ ($[M+H]^+$) 471.1336. Found 471.1348; **mp** = 53–55; **$[\alpha]_D^{25}$** = +182.3 (c =1.01, $CHCl_3$); **HPLC** Phenomenex Lux Cellulose-1 (95:5 *n*-Hexane:2-Propanol, 1 mL/min, 254 nm); t_R (major) = 4.2 min, t_R (minor) = 4.8 min (95:5 e.r.).

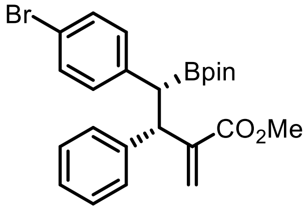

Minor diastereomer (3*S*,4*R*)-3c', white solid. **^{1}H NMR** (400 MHz, $CDCl_3$) δ 7.42 – 7.38 (m, 2H), 7.36 (d, *J* = 8.45 Hz, 2H), 7.30 – 7.23 (m, 2H), 7.21 – 7.14 (m, 3H), 6.09 (d, *J* = 0.78 Hz, 1H), 5.60 (t, *J* = 0.89 Hz, 1H), 4.55 (dd, *J* = 12.92, 0.79 Hz, 1H), 3.58 (s, 3H), 3.15 (d, *J* = 12.98 Hz, 1H), 0.88 (s, 6H), 0.86 (s, 6H) ppm; **^{13}C NMR** (101 MHz, $CDCl_3$) δ 167.3, 142.4, 142.1, 139.6, 131.5, 130.5, 128.9, 128.3, 126.8, 125.1, 119.4, 83.6, 51.8, 47.8, 36.5, 24.4, 24.2 ppm; **^{11}B NMR** (128 MHz, $CDCl_3$) δ 31.91 ppm; **HRMS (ESI)** Calculated for $[C_{24}H_{29}BBrO_4]^+$ ($[M+H]^+$) 471.1336. Found 471.1349; **mp** = 111–113; **$[\alpha]_D^{25}$** = +61.1 (c = 1.23, $CHCl_3$); **HPLC** Phenomenex Lux Cellulose-1 (95:5 *n*-Hexane:2-Propanol, 1 mL/min, 254 nm); t_R (major) = 4.0 min, t_R (minor) = 7,6 min (84:16 e.r.).

methyl 2-methylene-3-phenyl-4-(4,4,5,5-tetramethyl-1,3,2-dioxaborolan-2-yl)-4-(4-(trifluoromethyl)phenyl)butanoate

***3d** was prepared following the procedure described in section **F1**.*

58% yield (53 mg, 0.12 mmol) of a 5:1 diastereomeric ratio. The two diastereoisomers were readily separated by flash chromatography using *n*-hexane/ethyl acetate = 95:5.

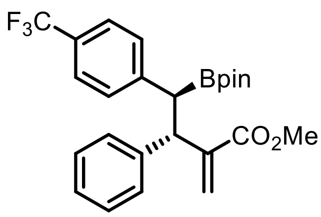

Major diastereomer (3*S*,4*S*)-3d, white solid. **^{1}H NMR** (400 MHz, $CDCl_3$) δ 7.36 – 7.31 (m, 2H), 7.21 – 7.15 (m, 2H), 7.10 – 6.93 (m, 5H), 6.39 (s, 1H), 5.96 (d, J = 1.03 Hz, 1H), 4.40 (dd, J = 12.73, 0.94 Hz, 1H), 3.68 (s, 3H), 2.98 (d, J = 12.85 Hz, 1H), 1.18 (s, 6H), 1.13 (s, 6H) ppm; **^{13}C NMR** (101 MHz, $CDCl_3$) δ 167.0, 144.5, 144.4, 141.4, 135.1, 129.5, 128.1, 128.1, 127.9 (q, $^{2}J_{CF}$ = 32.9 Hz), 126.4, 125.1 (q, $^{3}J_{CF}$ = 3.82 Hz), 124.5 (q, $^{1}J_{CF}$ = 271.6 Hz) 123.8, 84.0, 52.1, 48.6, 38.6, 24.7, 24.5 ppm; **^{19}F NMR** (376 MHz, $CDCl_3$) δ -62.3 (s) ppm; **^{11}B NMR** (128 MHz, $CDCl_3$) δ 30.87 ppm; **HRMS (ESI)** Calculated for $[C_{25}H_{29}BO_4F_3]^+$ ($[M+H]^+$) 461.2105. Found 461.2111; **mp** = 92–94; **$[\alpha]_D^{25}$** = +138.8 (c = 1.84, $CHCl_3$); **HPLC** Phenomenex Lux Cellulose-2 (98:2 *n*-Hexane:2-Propanol, 1 mL/min, 254 nm); t_R (major) = 4.1 min, t_R (minor) = 4.6 min (96:4 e.r.).

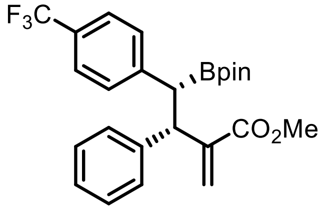

Minor diastereomer (3*S*,4*R*)-3d', white solid. **^{1}H NMR** (400 MHz, $CDCl_3$) δ 7.53 – 7.47 (m, 2H), 7.44 – 7.38 (m, 4H), 7.31 – 7.25 (m, 2H), 7.22 – 7.14 (m, 1H), 6.09 (d, J = 0.72 Hz, 1H), 5.60 (d, J = 0.92 Hz, 1H), 4.62 (dd, J = 12.99, 0.82 Hz, 1H), 3.57 (s, 3H), 3.27 (d, J = 13.02 Hz, 1H), 0.89 (s, 6H), 0.86 (s, 6H) ppm; **^{13}C NMR** (101 MHz, $CDCl_3$) δ 167.3, 144.9, 142.2, 142.1, 129.0, 128.9, 128.4, 127.9 (q, $^{2}J_{CF}$ = 32.3 Hz), 126.9, 125.4 (q, $^{3}J_{CF}$ = 3.82 Hz), 125.1, 124.5 (q, $^{1}J_{CF}$ = 271.5 Hz), 83.7, 51.9, 47.7, 37.2, 24.4, 24.2 ppm; **^{19}F NMR** (376 MHz, $CDCl_3$) δ -62.2 (s) ppm; **^{11}B NMR** (128 MHz, $CDCl_3$) δ 32.25 ppm; **HRMS (ESI)** Calculated for $[C_{25}H_{29}BO_4F_3]^+$ ($[M+H]^+$) 461.2105. Found 461.2116; **mp** = 125–127; **$[\alpha]_D^{25}$** = +26.3 (c = 0.50, $CHCl_3$); **HPLC** Phenomenex Lux Cellulose-1 (95:5 *n*-Hexane:2-Propanol, 1 mL/min, 254 nm); t_R (major) = 4.1 min, t_R (minor) = 9.0 min (84:16 e.r.).

methyl 2-methylene-4-(3-nitrophenyl)-3-phenyl-4-(4,4,5,5-tetramethyl-1,3,2-dioxaborolan-2-yl)butanoate

***3e** was prepared following the procedure described in section **F1**.*

55% (48 mg, 0.11 mmol) of a 2.5:1 diastereomeric ratio. The two diastereoisomers were readily separated by flash chromatography using *n*-hexane/ethyl acetate = 90:10.

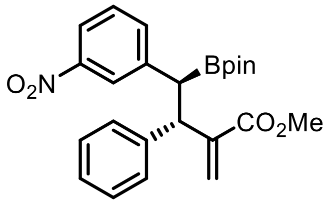

Major diastereomer (3*S*,4*S*)-3e, colourless oil. **^{1}H NMR** (400 MHz, $CDCl_3$) δ 8.00 (t, J = 2.0 Hz, 1H), 7.88 (ddd, J = 8.1, 2.3, 1.1 Hz, 1H), 7.33 (ddd, J = 7.7, 1.8, 1.2 Hz, 1H), 7.25 – 7.18 (m, 1H), 7.09 – 7.03 (m, 2H), 7.02 – 6.93 (m, 3H), 6.40 (s, 1H), 5.97 (d, J = 1.1 Hz, 1H), 4.38 (dd, J = 12.9, 1.0 Hz, 1H), 3.68 (s, 3H), 3.01 (d, J = 12.9 Hz, 1H), 1.18 (s, 6H), 1.14 (s, 6H) ppm; **^{13}C NMR** (101 MHz, $CDCl_3$) δ 166.8, 148.1, 144.0, 142.5, 140.9, 135.5, 128.8, 128.1, 128.0, 126.4, 123.9, 123.8, 120.8, 84.0, 52.0, 48.8, 38.3, 24.6, 24.3 ppm; **^{11}B NMR** (128 MHz, $CDCl_3$) δ 31.92 ppm; **HRMS (ESI)** Calculated for $[C_{24}H_{29}BNO_6]^+$ ($[M+H]^+$) 438.2082. Found 438.2079, **$[\alpha]_D^{25}$** = +145.5 (c =0.80, $CHCl_3$); **HPLC** Phenomenex Lux Cellulose-2 (95:5 *n*-Hexane:2-Propanol, 1 mL/min, 300 nm); t_R (major) = 5.9 min, t_R (minor) = 7.0 min (92:8 e.r.).

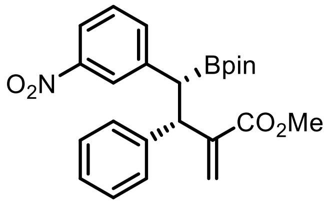

Minor diastereomer (3*S*,4*R*)-3e', colourless oil. **^{1}H NMR** (400 MHz, $CDCl_3$) δ 8.19 (t, J = 2.0 Hz, 1H), 8.00 (ddd, J = 8.2, 2.3, 1.1 Hz, 1H), 7.68 – 7.60 (m, 1H), 7.48 – 7.37 (m, 3H), 7.32 – 7.26 (m, 2H), 7.24 – 7.11 (m, 1H), 6.09 (d, J = 0.7 Hz, 1H), 5.61 (t, J = 0.8 Hz, 1H), 4.59 (d, J = 12.9 Hz, 1H), 3.58 (s, 3H), 3.35 (d, J = 12.9 Hz, 1H), 0.89 (s, 6H), 0.86 (s, 6H) ppm; **^{13}C NMR** (101 MHz, $CDCl_3$) δ 167.1, 148.5, 143.1, 141.9, 141.9, 135.1, 129.3, 128.8, 128.4, 127.1, 125.4, 123.7, 121.0, 83.9, 51.9, 48.3, 36.9, 24.4, 24.1 ppm; **^{11}B NMR** (128 MHz, $CDCl_3$) δ 31.80 ppm; **HRMS (ESI)** Calculated for $[C_{24}H_{29}BNO_6]^+$ ($[M+H]^+$) 438.2082. Found 438.2069; **$[\alpha]_D^{25}$** = +43.0 (c = 0.20, $CHCl_3$); **HPLC** Phenomenex Lux Cellulose-1 (95:5 *n*-Hexane:2-Propanol, 1 mL/min, 225 nm); t_R (major) = 5.2 min, t_R (minor) = 13.9 min (75:25 e.r.).

methyl 2-methylene-3-phenyl-4-(4,4,5,5-tetramethyl-1,3,2-dioxaborolan-2-yl)-4-(p-tolyl)butanoate

***3f** was prepared following the procedure described in section **F1**.*

58% yield (47 mg, 0.12 mmol) of a 3.2:1 diastereomeric ratio. The two diastereoisomers were readily separated by flash chromatography using *n*-hexane/ethyl acetate = 95:5.

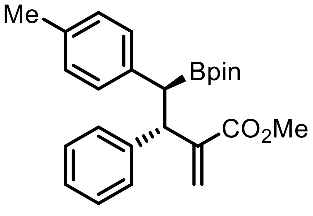

Major diastereomer (3*S*,4*S*)-3f, white solid. **^{1}H NMR** (400 MHz, $CDCl_3$) δ 7.10 – 6.85 (m, 9H), 6.37 (s, 1H), 5.95 (s, 1H), 4.42 – 4.30 (m, 1H), 3.67 (s, 3H), 2.86 (d, J = 12.84 Hz, 1H), 2.20 (s, 3H), 1.19 (s, 6H), 1.13 (s, 6H) ppm; **^{13}C NMR** (101 MHz, $CDCl_3$) δ 167.2, 145.0, 142.1, 136.7, 134.8, 129.1, 128.8, 128.3, 127.8, 126.0, 123.6, 83.6, 52.0, 48.7, 38.0, 24.7, 24.5, 21.1ppm; **^{11}B NMR** (128 MHz, $CDCl_3$) δ 31.81 ppm; **HRMS (ESI)** Calculated for $[C_{25}H_{32}BO_4]^+$ ($[M+H]^+$) 407.2388. Found 407.2396; **mp** = 98–100; **$[\alpha]_D^{25}$** = +151.4 (c =1.69, $CHCl_3$); **HPLC** Phenomenex Lux Cellulose-2 (98:2 *n*-Hexane:2-Propanol, 1 mL/min, 240 nm); t_R (major) = 4.9 min, t_R (minor) = 5.3 min (94:6 e.r.).

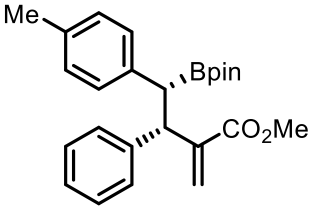

Minor diastereomer (3*S*,4*R*)-3f', white solid. **^{1}H NMR** (400 MHz, $CDCl_3$) δ 7.46 – 7.39 (m, 2H), 7.29 – 7.22 (m, 2H), 7.18 (dd, J = 8.37, 2.31 Hz, 3H), 7.04 (d, J = 7.83 Hz, 2H), 6.10 (d, J = 0.93 Hz, 1H), 5.64 (t, J = 1.04 Hz, 1H), 4.58 (d, J = 12.96 Hz, 1H), 3.57 (s, 3H), 3.13 (d, J = 12.95 Hz, 1H), 2.28 (s, 3H), 0.88 (s, 6H), 0.86 (s, 6H) ppm; **^{13}C NMR** (101 MHz, $CDCl_3$) δ 167.5, 142.9, 142.4, 137.2, 134.9, 129.2, 129.0, 128.7, 128.2, 126.6, 124.9, 83.3, 51.7, 47.7, 36.5, 24.4, 24.2, 21.1 ppm; **^{11}B NMR** (128 MHz, $CDCl_3$) δ 32.41 ppm; **HRMS (ESI)** Calculated for $[C_{25}H_{32}BO_4]^+$ ($[M+H]^+$) 407.2388. Found 407.2396; **mp** = 129–131; **$[\alpha]_D^{25}$** = +55.6 (c = 0.75, $CHCl_3$); **HPLC** Phenomenex Lux Cellulose-1 (95:5 *n*-Hexane:2-Propanol, 1 mL/min, 254 nm); t_R (major) = 4.6 min, t_R (minor) = 9.0 min (90:10 e.r.).

methyl 2-methylene-3-phenyl-4-(4,4,5,5-tetramethyl-1,3,2-dioxaborolan-2-yl)-4-(m-tolyl)butanoate

***3g** was prepared following the procedure described in section **F1**.*

37% yield (28 mg, 0.07 mmol) of a 2.3:1 diastereomeric ratio. The two diastereoisomers were readily separated by flash chromatography using *n*-hexane/ethyl acetate = 95:5.

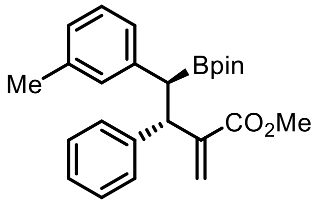

Major diastereomer (3*S*,4*S*)-3g, yellow oil. **^{1}H NMR** (500 MHz, $CDCl_3$) δ 7.05 (ddd, J = 7.8, 6.6, 1.0 Hz, 2H), 7.01 – 6.93 (m, 4H), 6.89 – 6.79 (m, 3H), 6.37 (s, 1H), 5.94 (t, J = 0.9 Hz, 1H), 4.36 (dd, J = 12.8, 1.0 Hz, 1H), 3.67 (s, 3H), 2.84 (d, J = 12.8 Hz, 1H), 2.18 (s, 3H), 1.18 (s, 6H), 1.13 (s, 6H) ppm; **^{13}C NMR** (101 MHz, $CDCl_3$) δ 167.3, 144.9, 142.0, 139.8, 137.5, 130.1, 128.2, 127.9, 127.8, 126.3, 126.3, 126.0, 123.7, 83.6, 52.0, 48.7, 38.4, 24.7, 24.5, 21.4 ppm; **^{11}B NMR** (128 MHz, $CDCl_3$) δ 33.28 ppm; **HRMS (ESI)** Calculated for $[C_{25}H_{32}BO_4]^+$ ($[M+H]^+$) 407.2388. Found 407.2388; **$[\alpha]_D^{25}$** = +155.4 (c =0.95, $CHCl_3$); **HPLC** Phenomenex Lux Cellulose-1 (95:5 *n*-Hexane:2-Propanol, 1 mL/min, 227 nm); t_R (major) = 4.2 min, t_R (minor) = 4.7 min (92:8 e.r.).

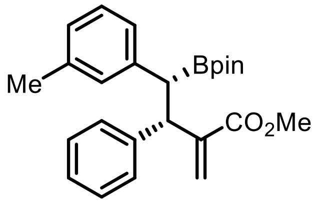

Minor diastereomer (3*S*,4*R*)-3g', yellow oil. **^{1}H NMR** (500 MHz, $CDCl_3$) δ 7.44 – 7.39 (m, 2H), 7.29 – 7.23 (m, 2H), 7.18 – 7.14 (m, 1H), 7.14 – 7.07 (m, 3H), 6.93 (dtd, J = 7.1, 1.7, 0.9 Hz, 1H), 6.10 (d, J = 0.9 Hz, 1H), 5.65 (t, J = 1.0 Hz, 1H), 4.59 (d, J = 12.9 Hz, 1H), 3.57 (s, 3H), 3.13 (d, J = 13.0 Hz, 1H), 2.30 (s, 3H), 0.88 (s, 6H), 0.86 (s, 6H) ppm; **^{13}C NMR** (101 MHz, $CDCl_3$) δ 167.5, 142.9, 142.3, 140.2, 137.8, 129.6, 129.0, 128.3, 128.2, 126.6, 126.4, 125.8, 124.9, 83.4, 51.7, 47.6, 36.9, 24.4, 24.1, 21.6 ppm; **^{11}B NMR** (128 MHz, $CDCl_3$) δ 31.96 ppm; **HRMS (ESI)** Calculated for $[C_{25}H_{32}BO_4]^+$ ($[M+H]^+$) 407.2388. Found 407.2387; **$[\alpha]_D^{25}$** = +7.6 (c = 0.54, $CHCl_3$); **HPLC** Phenomenex Lux Cellulose-1 (95:5 *n*-Hexane:2-Propanol, 1 mL/min, 208 nm); t_R (major) = 7.7 min, t_R (minor) = 9.0 min (91:9 e.r.).

methyl 2-methylene-3-phenyl-4-(4,4,5,5-tetramethyl-1,3,2-dioxaborolan-2-yl)-4-(o-tolyl)butanoate

***3h** was prepared following the procedure described in section **F1**.*

31% yield (25 mg, 0.06 mmol) of a 1.3:1 diastereomeric ratio. The two diastereoisomers were readily separated by flash chromatography using *n*-hexane/ethyl acetate = 95:5.

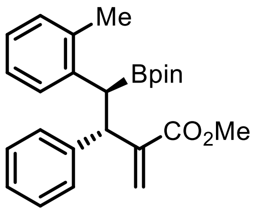

Major diastereomer (3*S*,4*S*)-3h, white solid. **^{1}H NMR** (400 MHz, $CDCl_3$) δ 7.48 (dd, *J* = 7.8, 1.4 Hz, 1H), 7.09 (td, *J* = 7.5, 1.6 Hz, 1H), 7.04 – 6.97 (m, 3H), 6.97 – 6.89 (m, 3H), 6.86 (ddt, *J* = 7.6, 1.6, 0.7 Hz, 1H), 6.43 (d, *J* = 0.6 Hz, 1H), 6.01 (t, *J* = 0.9 Hz, 1H), 4.43 (dd, *J* = 12.7, 1.1 Hz, 1H), 3.67 (s, 3H), 3.16 (d, *J* = 12.7 Hz, 1H), 1.95 (s, 3H), 1.16 (s, 6H), 1.10 (s, 6H) ppm; **^{13}C NMR** (101 MHz, $CDCl_3$) δ 167.3, 144.7, 141.8, 138.3, 136.8, 130.0, 128.7, 128.2, 127.7, 126.1, 125.7, 125.3, 123.9, 83.5, 52.0, 48.7, 33.3, 24.6, 24.5, 20.1 ppm; **^{11}B NMR** (128 MHz, $CDCl_3$) δ 32.03 ppm; **HRMS (ESI)** Calculated for $[C_{25}H_{32}BO_4]^+$ ($[M+H]^+$) 407.2388. Found 407.2392; **mp** = 119–121; **$[\alpha]_D^{25}$** = +145.0 (c = 0.76, $CHCl_3$); **HPLC** Phenomenex Lux Cellulose-2 (98:2 *n*-Hexane:2-Propanol, 1 mL/min, 254 nm); t_R (major) = 4.2 min, t_R (minor) = 4.8 min (94:6 e.r.).

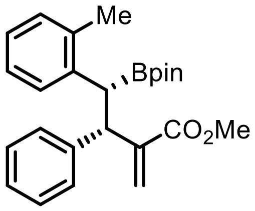

Minor diastereomer (3*S*,4*R*)-3h', white solid. **^{1}H NMR** (400 MHz, $CDCl_3$) δ 7.47 – 7.42 (m, 2H), 7.36 (dd, *J* = 7.7, 1.3 Hz, 1H), 7.30 – 7.24 (m, 2H), 7.21 – 7.15 (m, 1H), 7.15 – 7.06 (m, 2H), 7.01 (td, *J* = 7.4, 1.4 Hz, 1H), 6.05 (d, *J* = 0.9 Hz, 1H), 5.45 (t, *J* = 1.0 Hz, 1H), 4.78 (dd, *J* = 12.9, 0.9 Hz, 1H), 3.57 (s, 3H), 3.33 (d, *J* = 12.8 Hz, 1H), 2.42 (s, 3H), 0.87 (s, 6H), 0.84 (s, 6H) ppm; **^{13}C NMR** (101 MHz, $CDCl_3$) δ 167.6, 142.8, 142.6, 138.6, 136.1, 130.2, 129.1, 128.3, 127.5, 126.7, 126.2, 125.2, 124.5, 83.3, 51.8, 47.0, 32.9, 24.3, 24.3, 20.5 ppm; **^{11}B NMR** (128 MHz, $CDCl_3$) δ 32.53 ppm; **HRMS (ESI)** Calculated for $[C_{25}H_{32}BO_4]^+$ ($[M+H]^+$) 407.2388. Found 407.2391; **mp** = 86–88; **$[\alpha]_D^{25}$** = +123.9 (c = 0.47, $CHCl_3$); **HPLC** Phenomenex Lux Cellulose-1 (95:5 *n*-Hexane:2-Propanol, 1 mL/min, 254 nm); t_R (major) = 13.2 min, t_R (minor) = 5.8 min (94:6 e.r.).

methyl 4-(4-methoxyphenyl)-2-methylene-3-phenyl-4-(4,4,5,5-tetramethyl-1,3,2-dioxaborolan-2-yl)butanoate

***3i** was prepared following the procedure described in section **F1**.*

41% yield (35 mg, 0.08 mmol) of a 2.3:1 diastereomeric ratio. The two diastereoisomers were readily separated by flash chromatography using *n*-hexane/ethyl acetate = 98:2.

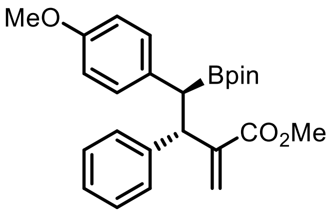

Major diastereomer (3*S*,4*S*)-3i, white solid. **^{1}H NMR** (400 MHz, $CDCl_3$) δ 7.44 – 7.37 (m, 2H), 7.29 – 7.23 (m, 2H), 7.20 (d, *J* = 8.70 Hz, 2H), 7.18 – 7.13 (m, 1H), 6.83 – 6.75 (m, 2H), 6.10 (d, *J* = 0.93 Hz, 1H), 5.63 (d, *J* = 1.05 Hz, 1H), 4.54 (d, *J* = 12.97 Hz, 1H), 3.77 (s, 3H), 3.57 (s, 3H), 3.10 (d, *J* = 12.93 Hz, 1H), 0.88 (s, 6H), 0.86 (s, 6H) ppm; **^{13}C NMR** (101 MHz, $CDCl_3$) δ 167.5, 157.6, 142.9, 142.4, 132.3, 129.7, 129.0, 128.2, 126.6, 125.0, 113.9, 83.4, 55.2, 51.7, 48.0, 36.0, 24.4, 24.2 ppm; **^{11}B NMR** (128 MHz, $CDCl_3$) δ 33.14 ppm; **HRMS (ESI)** Calculated for $[C_{25}H_{32}BO_5]^+$ ($[M+H]^+$) 423.2337. Found 423.2346; **mp** = 110–112; **$[\alpha]_D^{25}$** = +50.8 (c = 0.57, $CHCl_3$); **HPLC** Phenomenex Lux Cellulose-1 (98:2 *n*-Hexane:2-Propanol, 1 mL/min, 254 nm); t_R (major) = 6.4 min, t_R (minor) = 15.1 min (88:12 e.r.).

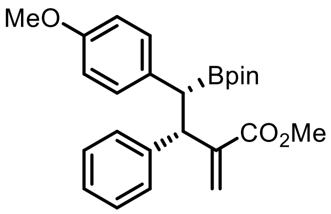

Minor diastereomer (3*S*,4*R*)-3i', white solid. **^{1}H NMR** (400 MHz, $CDCl_3$) δ 7.09 – 7.03 (m, 2H), 6.98 (dtd, *J* = 11.19, 6.54, 1.78 Hz, 5H), 6.67 – 6.58 (m, 2H), 6.36 (d, *J* = 0.62 Hz, 1H), 5.94 (t, *J* = 0.88 Hz, 1H), 4.31 (dd, *J* = 12.84, 0.99 Hz, 1H), 3.69 (s, 3H), 3.67 (s, 3H), 2.80 (d, *J* = 12.83 Hz, 1H), 1.18 (s, 6H), 1.12 (s, 6H) ppm; **^{13}C NMR** (101 MHz, $CDCl_3$) δ 167.3, 157.5, 144.9, 142.1, 131.9, 130.2, 128.3, 127.8, 126.0, 123.7, 113.5, 83.6, 55.1, 52.0, 48.9, 37.6, 24.7, 24.5 ppm; **^{11}B NMR** (128 MHz, $CDCl_3$) δ 32.68 ppm; **HRMS (ESI)** Calculated for $[C_{25}H_{32}BO_5]^+$ ($[M+H]^+$) 423.2337. Found 423.2349; **mp** = 80–82; **$[\alpha]_D^{25}$** = +83.3 (c = 0.41, $CHCl_3$); **HPLC** Phenomenex Lux Cellulose-1 (98:2 *n*-Hexane:2-Propanol, 1 mL/min, 254 nm); t_R (major) = 5.5 min, t_R (minor) = 6.6 min (83:17 e.r.).

methyl 2-methylene-3-phenyl-4-(4,4,5,5-tetramethyl-1,3,2-dioxaborolan-2-yl)-4-(thiophen-2-yl)butanoate

***3j** was prepared following the procedure described in section **F1**.*

57% yield (45 mg, 0.11 mmol) of a 4.5:1 diastereomeric ratio. The two diastereoisomers were readily separated by flash chromatography using *n*-hexane/ethyl acetate = 95:5.

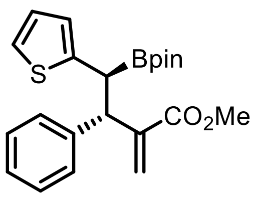

Major diastereomer (3*S*,4*S*)-3j, white solid. **^{1}H NMR** (400 MHz, $CDCl_3$) δ 7.15 – 7.09 (m, 2H), 7.09 – 7.02 (m, 3H), 6.94 (dd, J = 5.2, 1.2 Hz, 1H), 6.74 (dd, J = 5.1, 3.4 Hz, 1H), 6.62 (dd, J = 3.5, 1.2 Hz, 1H), 6.37 (s, 1H), 5.92 (d, J = 1.1 Hz, 1H), 4.35 – 4.25 (m, 1H), 3.67 (s, 3H), 3.18 (d, J = 12.7 Hz, 1H), 1.22 (s, 6H), 1.17 (s, 6H) ppm; **^{13}C NMR** (101 MHz, $CDCl_3$) δ 167.0, 144.3, 142.6, 141.8, 128.2, 128.0, 126.6, 126.3, 125.5, 123.8, 123.4, 84.0, 52.0, 49.9, 32.9, 24.7, 24.5 ppm; **^{11}B NMR** (128 MHz, $CDCl_3$) δ 32.37 ppm; **HRMS (ESI)** Calculated for $[C_{22}H_{28}BSO_4]^+$ ($[M+H]^+$) 399.1795. Found 399.1796; **mp** = 122–124; **$[\alpha]_D^{25}$** = +171.8 (c = 1.89, $CHCl_3$); **HPLC** Phenomenex Lux Cellulose-1 (95:5 *n*-Hexane:2-Propanol, 1 mL/min, 254 nm); t_R (major) = 4.5 min, t_R (minor) = 4.9 min (96:4 e.r.).

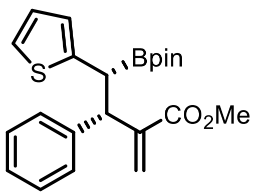

Minor diastereomer (3*S*,4*R*)-3j', white solid. **^{1}H NMR** (400 MHz, $CDCl_3$) δ 7.44 – 7.33 (m, 2H), 7.30 – 7.22 (m, 2H), 7.21 – 7.14 (m, 1H), 7.06 (dd, J = 5.1, 1.3 Hz, 1H), 6.94 – 6.80 (m, 2H), 6.16 (d, J = 0.8 Hz, 1H), 5.72 (d, J = 1.0 Hz, 1H), 4.46 (d, J = 12.9 Hz, 1H), 3.61 (s, 3H), 3.54 (d, J = 12.8 Hz, 1H), 0.91 (s, 6H), 0.90 (s, 6H) ppm; **^{13}C NMR** (101 MHz, $CDCl_3$) δ 167.3, 143.5, 142.3, 142.1, 128.9, 128.3, 126.8, 125.0, 123.2, 83.7, 51.8, 49.8, 31.4, 24.4, 24.2 ppm; **^{11}B NMR** (128 MHz, $CDCl_3$) δ 31.95 ppm; **HRMS (ESI)** Calculated for $[C_{22}H_{28}BSO_4]^+$ ($[M+H]^+$) 399.1795. Found 399.1790; **mp** = 120–122; **$[\alpha]_D^{25}$** = +57.7 (c = 0.36, $CHCl_3$); **HPLC** Phenomenex Lux Cellulose-1 (98:2 *n*-Hexane:2-Propanol, 1 mL/min, 254 nm); t_R (major) = 15.7 min, t_R (minor) = 14.2 min (92:8 e.r.).

methyl 2-methylene-4-(naphthalen-2-yl)-3-phenyl-4-(4,4,5,5-tetramethyl-1,3,2-dioxaborolan-2-yl)butanoate

***3k** was prepared following the procedure described in section **F1**.*

80% yield (71 mg, 0.16 mmol) of a 4.0:1 diastereomeric ratio. The two diastereoisomers were readily separated by flash chromatography using *n*-hexane/ethyl acetate = 95:5.

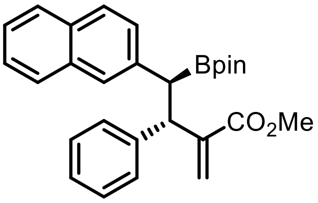

Major diastereomer (3*S*,4*S*)-3k, white solid. **^{1}H NMR** (400 MHz, $CDCl_3$) δ 7.73 – 7.65 (m, 2H), 7.60 (d, *J* = 8.5 Hz, 1H), 7.58 – 7.54 (m, 1H), 7.41 – 7.32 (m, 2H), 7.28 (dd, *J* = 8.5, 1.8 Hz, 1H), 7.07 – 6.99 (m, 4H), 6.98 – 6.92 (m, 1H), 6.43 (d, *J* = 0.5 Hz, 1H), 6.03 (t, *J* = 0.8 Hz, 1H), 4.57 (dd, *J* = 12.8, 1.0 Hz, 1H), 3.71 (s, 3H), 3.11 (d, *J* = 12.8 Hz, 1H), 1.20 (s, 6H), 1.13 (s, 6H) ppm; **^{13}C NMR** (101 MHz, $CDCl_3$) δ 167.2, 144.9, 141.8, 137.6, 133.7, 131.9, 128.2, 127.9, 127.9, 127.6, 127.6, 127.6, 127.5, 126.1, 125.6, 125.0, 123.8, 83.7, 52.0, 48.5, 38.7, 24.7, 24.5 ppm; **^{11}B NMR** (128 MHz, $CDCl_3$) δ 33.45 ppm; **HRMS (ESI)** Calculated for $[C_{28}H_{32}BO_4]^+$ ($[M+H]^+$) 443.2388. Found 443.2394; **mp** = 135–137; **$[\alpha]_D^{25}$** = +177.1 (c = 3.29, $CHCl_3$); **HPLC** Phenomenex Lux Cellulose-1 (95:5 *n*-Hexane:2-Propanol, 1 mL/min, 254 nm); t_R (major) = 4.6 min, t_R (minor) = 5.4 min (97:3 e.r.).

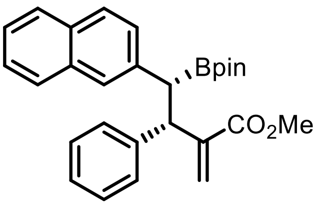

Minor diastereomer (3*S*,4*R*)-3k', white solid. **^{1}H NMR** (400 MHz, $CDCl_3$) δ 7.81 – 7.72 (m, 4H), 7.49 (ddd, J = 7.9, 6.3, 1.5 Hz, 3H), 7.46 – 7.37 (m, 2H), 7.34 – 7.27 (m, 2H), 7.23 – 7.16 (m, 1H), 6.07 (d, J = 0.9 Hz, 1H), 5.69 (t, J = 1.0 Hz, 1H), 4.76 (dd, J = 12.9, 0.9 Hz, 1H), 3.54 (s, 3H), 3.37 (d, J = 13.0 Hz, 1H), 0.88 (s, 6H), 0.86 (s, 6H) ppm; **^{13}C NMR** (101 MHz, $CDCl_3$) δ 167.4, 142.7, 142.2, 138.1, 133.9, 132.0, 129.0, 128.3, 128.0, 127.7, 127.7, 127.4, 127.1, 126.7, 125.8, 125.1, 125.1, 83.5, 51.7, 47.7, 37.3, 24.4, 24.2 ppm; **^{11}B NMR** (128 MHz, $CDCl_3$) δ 31.98 ppm; **HRMS (ESI)** Calculated for $[C_{28}H_{32}BO_4]^+$ ($[M+H]^+$) 443.2388. Found 443.2395; **mp** = 147–149; **$[\alpha]_D^{25}$** = +91.2 (c = 0.75, $CHCl_3$); **HPLC** Phenomenex Lux Cellulose-1 (95:5 *n*-Hexane:2-Propanol, 1 mL/min, 254 nm); t_R (major) = 5.0 min, t_R (minor) = 10.6 min (93:7 e.r.).

methyl 2-methylene-3,4-diphenyl-4-(4,4,5,5-tetramethyl-1,3,2-dioxaborolan-2-yl)pentanoate

***3l** was prepared following the procedure described in section **F1**.*

54% yield (44 mg, 0.11 mmol) of a 2.0:1 diastereomeric ratio. The two diastereoisomers were readily separated by flash chromatography using *n*-hexane/ethyl acetate = 95:5.

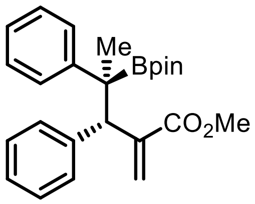

Major diastereomer (3*S*,4*R*)-3l, white solid. **^{1}H NMR** (400 MHz, $CDCl_3$) δ 7.49 – 7.43 (m, 2H), 7.42 – 7.37 (m, 2H), 7.28 – 7.22 (m, 4H), 7.21 – 7.15 (m, 1H), 7.15 – 7.09 (m, 1H), 6.13 (d, *J* = 0.8 Hz, 1H), 5.43 (t, *J* = 1.1 Hz, 1H), 5.12 (d, *J* = 1.2 Hz, 1H), 3.47 (s, 3H), 1.46 (s, 3H), 1.05 (s, 6H), 1.02 (s, 6H) ppm; **^{13}C NMR** (101 MHz, $CDCl_3$) δ 168.2, 145.3, 143.3, 138.9, 129.7, 128.3, 127.8, 127.2, 126.2, 125.7, 124.8, 83.7, 53.7, 52.0, 24.8, 24.6, 17.8 ppm; **^{11}B NMR** (128 MHz, $CDCl_3$) δ 32.90 ppm; **HRMS (ESI)** Calculated for $[C_{25}H_{32}BO_4]^+$ ($[M+H]^+$) 407.2388. Found 407.2393; **mp** = 93–95; **$[\alpha]_D^{25}$** = +35.5 (c = 1.77, $CHCl_3$); **HPLC** Phenomenex Lux Cellulose-1 (80:20 *n*-Hexane:2-Propanol, 1 mL/min, 254 nm); t_R (major) = 9.5 min, t_R (minor) = 8.7 min (96:4 e.r.).

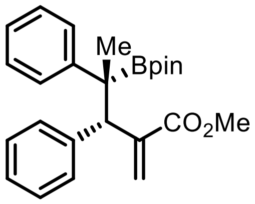

Minor diastereomer (3*S*,4*S*)-3l', white solid. **^{1}H NMR** (400 MHz, $CDCl_3$) δ 7.36 – 7.31 (m, 2H), 7.28 – 7.20 (m, 2H), 7.17 – 7.12 (m, 1H), 7.09 – 7.00 (m, 3H), 6.75 – 6.68 (m, 2H), 6.44 (s, 1H), 6.01 (d, *J* = 1.4 Hz, 1H), 4.71 (d, *J* = 1.3 Hz, 1H), 3.59 (s, 3H), 1.33 (s, 3H), 1.19 (s, 6H), 1.13 (s, 6H) ppm; **^{13}C NMR** (101 MHz, $CDCl_3$) δ 168.6, 144.4, 141.8, 141.2, 130.4, 128.0, 127.9, 127.8, 126.7, 126.4, 125.4, 83.7, 51.8, 50.6, 24.3, 16.8 ppm; **^{11}B NMR** (128 MHz, $CDCl_3$) δ 34.44 ppm; **HRMS (ESI)** Calculated for $[C_{25}H_{32}BO_4]^+$ ($[M+H]^+$) 407.2388. Found 407.2394; **mp** = 136–138; **$[\alpha]_D^{25}$** = +94.1 (c = 1.03, $CHCl_3$); **HPLC** Phenomenex Lux Cellulose-1 (98:2 *n*-Hexane:2-Propanol, 1 mL/min, 254 nm); t_R (major) = 4.9 min, t_R (minor) = 4.4 min (87:13 e.r.).

methyl 3-(4-fluorophenyl)-2-methylene-4-phenyl-4-(4,4,5,5-tetramethyl-1,3,2-dioxaborolan-2-yl)butanoate

***3m** was prepared following the procedure described in section **F1**.*

56% yield (46 mg, 0.11 mmol) of a 2.0:1 diastereomeric ratio. The two diastereoisomers were readily separated by flash chromatography using *n*-hexane/ethyl acetate = 95:5.

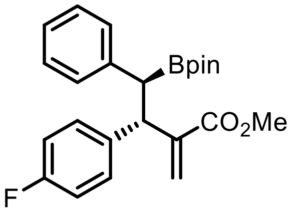

Major diastereomer (3*S*,4*S*)-3m, white solid. **^{1}H NMR** (500 MHz, $CDCl_3$) δ 7.14 – 6.97 (m, 5H), 6.95 – 6.84 (m, 2H), 6.72 (t, *J* = 8.7 Hz, 2H), 6.38 (s, 1H), 5.95 (s, 1H), 4.35 (d, *J* = 12.9 Hz, 1H), 3.67 (s, 3H), 2.81 (d, *J* = 12.8 Hz, 1H), 1.18 (s, 6H), 1.12 (s, 6H) ppm; **^{13}C NMR** (101 MHz, $CDCl_3$) δ 167.1, 161.3 (d, $^1J_{CF}$ = 243.8 Hz), 144.7, 139.8,137.6 (d, $^4J_{CF}$ = 3.0 Hz), 129.6 (d, $^3J_{CF}$ = 8.0 Hz), 129.3, 128.2, 125.7, 123.7, 114.6 (d, $^2J_{CF}$ = 21.2 Hz), 83.7, 52.0, 48.2, 38.7, 24.7, 24.5 ppm; **^{19}F NMR** (376 MHz, $CDCl_3$) δ -117.41 (m) ppm; **^{11}B NMR** (128 MHz, $CDCl_3$) δ 31.14 ppm; **HRMS (ESI)** Calculated for $[C_{24}H_{29}BFO_4]^+$ ($[M+H]^+$) 411.2128. Found 411.2149; **mp** = 126–128; **$[\alpha]_D^{25}$** = +179.0 (c = 0.71, $CHCl_3$); **HPLC** Phenomenex Lux Amylose-1 (99:1 *n*-Hexane:2-Propanol, 1 mL/min, 230 nm); t_R (major) = 9.4 min, t_R (minor) = 6.1 min (92:8 e.r.).

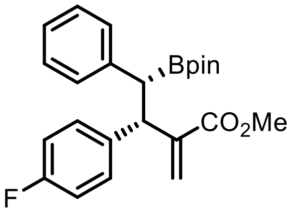

Minor diastereomer (3*S*,4*R*)-3m', white solid. **^{1}H NMR** (500 MHz, $CDCl_3$) δ 7.42 – 7.36 (m, 2H), 7.30 – 7.20 (m, 4H), 7.15 – 7.09 (m, 1H), 6.99 – 6.92 (m, 2H), 6.08 (s, 1H), 5.61 (s, 1H), 4.59 (d, *J* = 13.0 Hz, 1H), 3.57 (s, 3H), 3.13 (d, *J* = 13.0 Hz, 1H), 0.89 (m, 12H) ppm; **^{13}C NMR** (101 MHz, $CDCl_3$) δ 167.3, 161.8 (d, $^1J_{CF}$ = 244.4 Hz), 142.1, 140.2, 138.6 (d, $^3J_{CF}$ = 3.1 Hz), 130.5 (d, $^3J_{CF}$ = 7.9 Hz), 128.6 (d, $^2J_{CF}$ = 23.7 Hz), 125.7, 125.1, 115.0, 114.8, 83.5, 51.8, 47.0, 37.1, 24.4, 24.2 ppm; **^{19}F NMR** (376 MHz, $CDCl_3$) δ -116.66 (ddd, *J* = 14.1, 8.9, 5.5 Hz) ppm; **^{11}B NMR** (128 MHz, $CDCl_3$) δ 33.03 ppm; **HRMS (ESI)** Calculated for $[C_{24}H_{29}BFO_4]^+$ ($[M+H]^+$) 411.2128. Found 411.2130; **mp** = 101–103; **$[\alpha]_D^{25}$** = +69 (c = 0.48, $CHCl_3$); **HPLC** Phenomenex Lux Cellulose-1 (95:5 *n*-Hexane:2-Propanol, 1 mL/min, 254 nm); t_R (major) = 7.6 min, t_R (minor) = 4.2 min (87:13 e.r.).

methyl 3-(4-chlorophenyl)-2-methylene-4-phenyl-4-(4,4,5,5-tetramethyl-1,3,2-dioxaborolan-2-yl)butanoate

***3n** was prepared following the procedure described in section **F1**.*

58% yield (49 mg, 0.12 mmol) of a 1.8:1 diastereomeric ratio. The two diastereoisomers were readily separated by flash chromatography using *n*-hexane/ethyl acetate = 95:5.

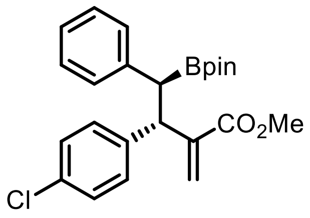

Major diastereomer (3*S*,4*S*)-3n, white solid. **^{1}H NMR** (500 MHz, $CDCl_3$) δ 7.13 – 6.96 (m, 7H), 6.92 – 6.84 (m, 2H), 6.38 (s, 1H), 5.95 (s, 1H), 4.35 (d, *J* = 12.8 Hz, 1H), 3.67 (d, *J* = 1.9 Hz, 3H), 2.81 (d, *J* = 12.8 Hz, 1H), 1.18 (s, 6H), 1.11 (s, 6H) ppm; **^{13}C NMR** (101 MHz, $CDCl_3$) δ 167.0, 144.4, 140.6, 139.6, 131.7, 129.5, 129.2, 128.3, 128.0, 125.8, 123.9, 83.7, 52.1, 48.3, 38.5, 24.7, 24.5 ppm; **^{11}B NMR** (160 MHz, $CDCl_3$) δ 32.55 ppm; **HRMS (ESI)** Calculated for $[C_{24}H_{29}BClO_4]^+$ ($[M+H]^+$) 427.1841. Found 427.1848; **mp** = 162–164; **$[\alpha]_D^{25}$** = +165.4 (c = 1.67, $CHCl_3$); **HPLC** Phenomenex Lux Cellulose-2 (98:2 *n*-Hexane:2-Propanol, 1 mL/min, 254 nm); t_R (major) = 4.2 min, t_R (minor) = 4.8 min (93:7 e.r.).

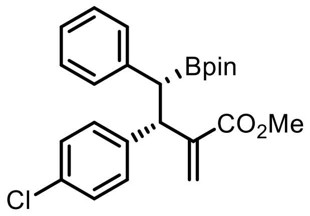

Minor diastereomer (3*S*,4*R*)-3n', white solid. **^{1}H NMR** (500 MHz, $CDCl_3$) δ 7.39 – 7.35 (m, 2H), 7.29 – 7.21 (m, 6H), 7.15 – 7.10 (m, 1H), 6.10 (s, 1H), 5.62 (s, 1H), 4.58 (d, *J* = 12.9 Hz, 1H), 3.57 (s, 3H), 3.13 (d, *J* = 13.0 Hz, 1H), 0.90 (s, 12H) ppm; **^{13}C NMR** (101 MHz, $CDCl_3$) δ 167.2, 141.9, 141.4, 140.0, 132.4, 130.4, 128.7, 128.5, 128.3, 125.7, 125.4, 83.6, 51.8, 47.2, 36.9, 24.4, 24.2 ppm; **^{11}B NMR** (128 MHz, $CDCl_3$) δ 33.09 ppm; **HRMS (ESI)** Calculated for $[C_{24}H_{29}BClO_4]^+$ ($[M+H]^+$) 427.1841. Found 427.1847; **mp** = 118–120; **$[\alpha]_D^{25}$** = +64.1 (c = 0.71, $CHCl_3$); **HPLC** Phenomenex Lux Cellulose-1 (95:5 *n*-Hexane:2-Propanol, 1 mL/min, 254 nm); t_R (major) = 8.2 min, t_R (minor) = 4.2 min (82:18 e.r.).

methyl 3-(4-bromophenyl)-2-methylene-4-phenyl-4-(4,4,5,5-tetramethyl-1,3,2-dioxaborolan-2-yl)butanoate

***3o** was prepared following the procedure described in section **F1**.*

58% yield (55 mg, 0.12 mmol) of a 2.9:1 diastereomeric ratio. The two diastereoisomers were readily separated by flash chromatography using *n*-hexane/ethyl acetate = 95:5.

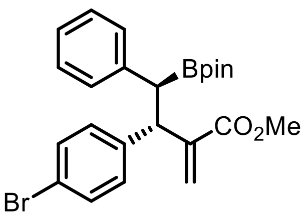

Major diastereomer (3*S*,4*S*)-3o, white solid. **^{1}H NMR** (400 MHz, $CDCl_3$) δ 7.43 – 7.38 (m, 2H), 7.34 – 7.29 (m, 2H), 7.29 – 7.20 (m, 4H), 7.14 – 7.09 (m, 1H), 6.10 (d, *J* = 0.8 Hz, 1H), 5.61 (t, *J* = 0.9 Hz, 1H), 4.56 (dd, *J* = 12.9, 0.9 Hz, 1H), 3.57 (s, 3H), 3.12 (d, *J* = 12.9 Hz, 1H), 0.90 (s, 12H) ppm; **^{13}C NMR** (101 MHz, $CDCl_3$) δ 167.2, 141.9, 141.8, 140.0, 131.3, 130.8, 128.7, 128.5, 125.7, 125.4, 120.5, 83.6, 51.8, 47.3, 36.8, 24.4, 24.2, 14.3 ppm; **^{11}B NMR** (128 MHz, $CDCl_3$) δ 32.62 ppm; **HRMS (ESI)** Calculated for $[C_{24}H_{29}BBrO_4]^+$ ($[M+H]^+$) 471.1336. Found 471.1338; **mp** = 107–109; **$[\alpha]_D^{25}$** = +37.6 (c = 0.78, $CHCl_3$); **HPLC** Phenomenex Lux Amylose-1 (99:1 *n*-Hexane:2-Propanol, 1 mL/min, 254 nm); t_R (major) = 12.3 min, t_R (minor) = 7.3 min (93:7 e.r.).

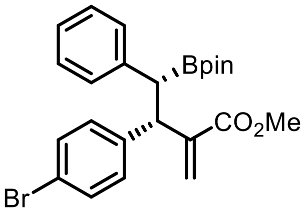

Minor diastereomer (3*S*,4*R*)-3o', white solid. **^{1}H NMR** (400 MHz, $CDCl_3$) δ 7.19 – 7.14 (m, 2H), 7.13 – 7.07 (m, 2H), 7.07 – 7.00 (m, 3H), 6.88 – 6.80 (m, 2H), 6.38 (s, 1H), 5.95 (d, *J* = 1.2 Hz, 1H), 4.34 (dd, *J* = 12.9, 1.1 Hz, 1H), 3.67 (s, 3H), 2.81 (d, *J* = 12.8 Hz, 1H), 1.17 (s, 6H), 1.11 (s, 6H) ppm; **^{13}C NMR** (101 MHz, $CDCl_3$) δ 167.0, 144.4, 141.1, 139.5, 130.9, 129.9, 129.2, 128.3, 125.8, 124.0, 119.9, 83.7, 52.1, 48.3, 38.4, 24.7, 24.5 ppm; **^{11}B NMR** (128 MHz, $CDCl_3$) δ 31.72 ppm; **HRMS (ESI)** Calculated for $[C_{24}H_{29}BBrO_4]^+$ ($[M+H]^+$) 471.1336. Found 471.1350; **mp** = 169–171; **$[\alpha]_D^{25}$** = +61.4 (c = 0.98, $CHCl_3$); **HPLC** Phenomenex Lux Cellulose-1 (98:2 *n*-Hexane:2-Propanol, 1 mL/min, 254 nm); t_R (major) = 11.5 min, t_R (minor) = 4.8 min (81:19 e.r.).

methyl 2-methylene-4-phenyl-4-(4,4,5,5-tetramethyl-1,3,2-dioxaborolan-2-yl)-3-(4-(trifluoromethyl)phenyl)butanoate

***3p** was prepared following the procedure described in section **F1**.*

52% yield (48 mg, 0.10 mmol) of a 3.0:1 diastereomeric ratio. The two diastereoisomers were readily separated by flash chromatography using *n*-hexane/ethyl acetate = 95:5.

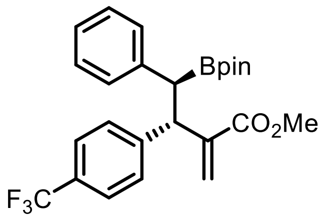

Major diastereomer (3*S*,4*S*)-3p, white solid. **^{1}H NMR** (400 MHz, $CDCl_3$) δ 7.30 (d, *J* = 8.0 Hz, 2H), 7.12 – 6.99 (m, 7H), 6.43 (s, 1H), 6.00 (d, *J* = 1.1 Hz, 1H), 4.44 (d, *J* = 12.9 Hz, 1H), 3.68 (s, 3H), 2.85 (d, *J* = 12.9 Hz, 1H), 1.18 (s, 6H), 1.12 (s, 6H) ppm; **^{13}C NMR** (101 MHz, $CDCl_3$) δ 166.9, 146.3, 144.1, 139.3, 129.2, 128.5, 128.3, 128.2 (q, $^2J_{CF}$ = 32.2 Hz), 125.9, 124.8 (q, $^3J_{CF}$ = 3.7 Hz), 124.4, 124.3 (q, $^1J_{CF}$ = 271.9 Hz), 83.8, 52.1, 48.7, 38.4, 24.7, 24.5 ppm; **^{19}F NMR** (376 MHz, $CDCl_3$) δ -62.4 (s) ppm; **^{11}B NMR** (128 MHz, $CDCl_3$) δ 32.15 ppm; **HRMS (ESI)** Calculated for $[C_{25}H_{29}BF_3O_4]^+$ ($[M+H]^+$) 461.2105. Found 461.2100; **mp** = 136–138; **$[\alpha]_D^{25}$** = +113.5 (c = 1.12, $CHCl_3$); **HPLC** Phenomenex Lux Amylose-1 (98:2 *n*-Hexane:2-Propanol, 1 mL/min, 240 nm); t_R (major) = 7.0 min, t_R (minor) = 4.7 min (88:12 e.r.).

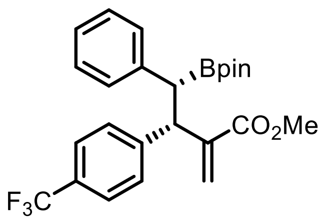

Minor diastereomer (3*S*,4*R*)-3p', white solid. **^{1}H NMR** (400 MHz, $CDCl_3$) δ 7.59 – 7.51 (m, 4H), 7.32 – 7.21 (m, 4H), 7.16 – 7.10 (m, 1H), 6.15 (d, *J* = 0.7 Hz, 1H), 5.68 (t, *J* = 0.9 Hz, 1H), 4.66 (d, *J* = 13.0 Hz, 1H), 3.58 (s, 3H), 3.17 (d, *J* = 12.9 Hz, 1H), 0.87 (m, 12H) ppm; **^{13}C NMR** (101 MHz, $CDCl_3$) δ 167.1, 147.1, 141.4, 139.8, 129.4, 129.1 (q, $^2J_{CF}$ = 32.5 Hz), 128.7, 128.6, 125.9, 125.1 (q, $^3J_{CF}$ = 3.8 Hz), 124.4 (q, $^1J_{CF}$ = 271.8 Hz), 83.6, 51.9, 47.7, 36.7, 24.3, 24.1 ppm; **^{19}F NMR** (376 MHz, $CDCl_3$) δ -62.4 (s) ppm; **^{11}B NMR** (128 MHz, $CDCl_3$) δ 32.41 ppm; **HRMS (ESI)** Calculated for $[C_{25}H_{29}BF_3O_4]^+$ ($[M+H]^+$) 461.2124. Found 461.2100; **mp** = 108–110; **$[\alpha]_D^{25}$** = +31.4 (c = 0.39, $CHCl_3$); **HPLC** Phenomenex Lux Cellulose-1 (98:2 *n*-Hexane:2-Propanol, 1 mL/min, 218 nm); t_R (major) = 10.6 min, t_R (minor) = 4.3 min (69:31 e.r.).

methyl 3-(4-cyanophenyl)-2-methylene-4-phenyl-4-(4,4,5,5-tetramethyl-1,3,2-dioxaborolan-2-yl)butanoate

***3q** was prepared following the procedure described in section **F1**.*

50% yield (42 mg, 0.10 mmol) of a 2.2:1 diastereomeric ratio. The two diastereoisomers were readily separated by flash chromatography using *n*-hexane/ethyl acetate = 90:10.

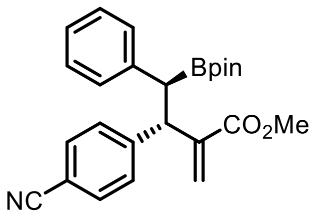

Major diastereomer (3*S*,4*S*)-3q, yellowish oil. **^{1}H NMR** (400 MHz, $CDCl_3$) δ 7.39 – 7.29 (m, 2H), 7.13 – 6.96 (m, 7H), 6.44 (s, 1H), 6.00 (d, J = 1.22 Hz, 1H), 4.40 (dd, J = 12.84, 1.08 Hz, 1H), 3.67 (s, 3H), 2.79 (d, J = 12.82 Hz, 1H), 1.18 (s, 6H), 1.12 (s, 6H) ppm; **^{13}C NMR** (101 MHz, $CDCl_3$) δ 166.7, 147.9, 143.5, 139.0, 131.7, 129.1, 128.9, 128.4, 126.0, 124.7, 119.2, 109.9, 83.9, 52.2, 49.2, 38.3, 24.7, 24.5 ppm; **^{11}B NMR** (160 MHz, $CDCl_3$) δ 32.23 ppm; **HRMS (ESI)** Calculated for $[C_{25}H_{29}BNO_4]^+$ ($[M+H]^+$) 418.2184. Found 418.2195; **$[\alpha]_D^{25}$** = +185.7 (c = 0.94, $CHCl_3$); **HPLC** Phenomenex Lux Amylose-1 (95:5 *n*-Hexane:2-Propanol, 1 mL/min, 254 nm); t_R (major) = 12.6 min, t_R (minor) = 8.0 min (86:14 e.r.).

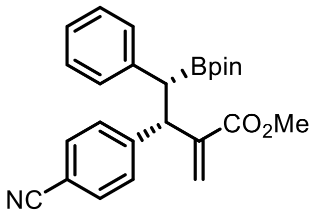

Minor diastereomer (3*S*,4*R*)-3q', yellowish solid. **^{1}H NMR** (400 MHz, $CDCl_3$) δ 7.61 – 7.52 (m, 4H), 7.30 – 7.20 (m, 4H), 7.19 – 7.09 (m, 1H), 6.15 (d, J = 0.62 Hz, 1H), 5.67 (t, J = 0.82 Hz, 1H), 4.64 (dd, J = 12.96, 0.93 Hz, 1H), 3.58 (s, 3H), 3.15 (d, J = 12.91 Hz, 1H), 0.90 (s, 6H), 0.88 (s, 6H); **^{13}C NMR** (101 MHz, $CDCl_3$) δ 166.9, 148.6, 141.0, 139.5, 132.1, 129.9, 128.7, 128.6, 126.3, 126.0, 119.1, 116.0, 110.5, 83.7, 51.9, 48.0, 36.4, 24.3, 24.2 ppm; **^{11}B NMR** (160 MHz, $CDCl_3$) δ 32.42 ppm; **HRMS (ESI)** Calculated for $[C_{25}H_{29}BNO_4]^+$ ($[M+H]^+$) 418.2184. Found 418.2188; **mp** = 140–142; **$[\alpha]_D^{25}$** = +44.2 (c = 0.91, $CHCl_3$); **HPLC** Phenomenex Lux Cellulose-1 (95:5 *n*-Hexane:2-Propanol, 1 mL/min, 254 nm); t_R (major) = 13.2 min, t_R (minor) = 9.3 min (75:25 e.r.).

methyl 4-(3-(methoxycarbonyl)-1-phenyl-1-(4,4,5,5-tetramethyl-1,3,2-dioxaborolan-2-yl)but-3-en-2-yl)benzoate

***3r** was prepared following the procedure described in section **F1**.*

62% yield (56 mg, 0.12 mmol) of a 2.3:1 diastereomeric ratio. The two diastereoisomers were readily separated by flash chromatography using *n*-hexane/ethyl acetate = 98:2.

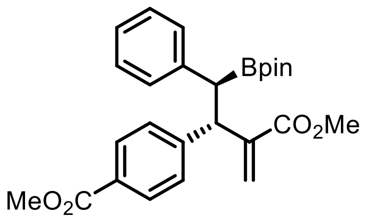

Major diastereomer (1*S*,2*S*)-3r, white solid. **^{1}H NMR** (400 MHz, $CDCl_3$) δ 7.75 – 7.68 (m, 2H), 7.11 – 6.95 (m, 7H), 6.42 (s, 1H), 5.99 (d, J = 1.16 Hz, 1H), 4.42 (dd, J = 12.81, 1.07 Hz, 1H), 3.81 (s, 3H), 3.66 (s, 3H), 2.84 (d, J = 12.77 Hz, 1H), 1.18 (s, 6H), 1.12 (s, 6H) ppm; **^{13}C NMR** (101 MHz, $CDCl_3$) δ 167.2, 167.0, 147.7, 144.1, 139.4, 129.3, 129.2, 128.3, 128.3, 128.0, 125.8, 124.3, 83.8, 52.1, 52.0, 48.9, 38.5, 24.7, 24.5ppm; **^{11}B NMR** (128 MHz, $CDCl_3$) δ 32.51 ppm; **HRMS (ESI)** Calculated for $[C_{26}H_{32}BO_6]^+$ ($[M+H]^+$) 451.2286. Found 451.2297; **mp** = 133–135; **$[\alpha]_D^{25}$** = +198.6 (c = 1.78, $CHCl_3$); **HPLC** Phenomenex Lux Amylose-1 (99:1 *n*-Hexane:2-Propanol, 1 mL/min, 254 nm); t_R (major) = 16.7 min, t_R (minor) = 23.2 min (>99:1 e.r.).

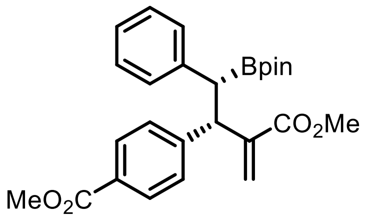

Minor diastereomer (1*S*,2*R*)-3r', white solid. **^{1}H NMR** (400 MHz, $CDCl_3$) δ 7.99 – 7.92 (m, 2H), 7.55 – 7.47 (m, 2H), 7.31 – 7.19 (m, 4H), 7.17 – 7.08 (m, 1H), 6.12 (d, J = 0.73 Hz, 1H), 5.64 (t, J = 0.88 Hz, 1H), 4.65 (d, J = 12.91 Hz, 1H), 3.90 (s, 3H), 3.56 (s, 3H), 3.17 (d, J = 12.90 Hz, 1H), 0.87 (s, 6H), 0.87 (s, 6H); **^{13}C NMR** (101 MHz, $CDCl_3$) δ 167.2, 167.2, 148.3, 141.6, 139.9, 129.6, 129.0, 128.8, 128.5, 125.9, 125.8, 83.6, 52.1, 51.8, 47.9, 36.7, 24.4, 24.2; **^{11}B NMR** (128 MHz, $CDCl_3$) δ 31.80 ppm; **HRMS (ESI)** Calculated for $[C_{26}H_{32}BO_6]^+$ ($[M+H]^+$) 451.2286. Found 451.2297; **mp** = 126–128; **$[\alpha]_D^{25}$** = +60.5 (c = 0.99, $CHCl_3$); **HPLC** Phenomenex Lux Cellulose-1 (99:1 *n*-Hexane:2-Propanol, 1 mL/min, 254 nm); t_R (major) = 11.0 min, t_R (minor) = 42.9 min (>99:1 e.r.).

methyl 2-methylene-4-phenyl-4-(4,4,5,5-tetramethyl-1,3,2-dioxaborolan-2-yl)-3-(p-tolyl)butanoate

***3s** was prepared following the procedure described in section **F1**.*

52% yield (47 mg, 0.10 mmol) of a 3.5:1 diastereomeric ratio. The two diastereoisomers were readily separated by flash chromatography using *n*-hexane/ethyl acetate = 95:5.

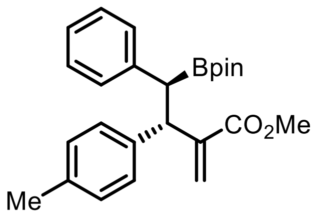

Major diastereomer (3*S*,4*S*)-3s, white solid. **^{1}H NMR** (500 MHz, $CDCl_3$) δ 7.12 – 7.05 (m, 4H), 7.05 – 6.97 (m, 1H), 6.91 – 6.82 (m, 4H), 6.35 (s, 1H), 5.93 (t, J = 0.9 Hz, 1H), 4.36 (dd, J = 12.8, 1.0 Hz, 1H), 3.68 (s, 3H), 2.88 (d, J = 12.8 Hz, 1H), 2.17 (s, 3H), 1.17 (s, 6H), 1.12 (s, 6H) ppm; **^{13}C NMR** (126 MHz, $CDCl_3$) δ 167.3, 145.1, 140.1, 138.9, 135.4, 129.3, 128.6, 128.1, 128.1, 125.5, 123.5, 83.6, 52.0, 48.2, 38.6, 24.7, 24.5, 21.1 ppm; **^{11}B NMR** (128 MHz, $CDCl_3$) δ 33.36 ppm; **HRMS (ESI)** Calculated for $[C_{25}H_{32}BO_4]^+$ ($[M+H]^+$) 407.2388. Found 407.2393; **mp** = 164–166; **$[\alpha]_D^{25}$** = +180.4 (c =1.85, $CHCl_3$); **HPLC** Phenomenex Lux Amylose-1 (95:5 *n*-Hexane:2-Propanol, 1 mL/min, 254 nm); t_R (major) = 5.0 min, t_R (minor) = 4.2 min (95:5 e.r.).

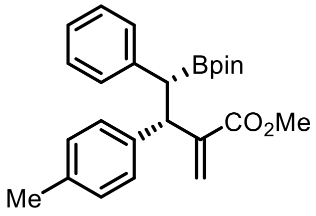

Minor diastereomer (3*S*,4*R*)-3s', white solid. **^{1}H NMR** (500 MHz, $CDCl_3$) δ 7.32 – 7.27 (m, 4H), 7.23 (dd, J = 8.5, 6.8 Hz, 2H), 7.11 (t, J = 7.2 Hz, 1H), 7.09 – 7.05 (m, 2H), 6.07 (d, J = 0.9 Hz, 1H), 5.61 (t, J = 1.0 Hz, 1H), 4.57 (d, J = 12.6 Hz, 1H), 3.56 (s, 3H), 3.15 (d, J = 12.9 Hz, 1H), 2.29 (s, 3H), 0.89 (s, 6H), 0.87 (s, 6H) ppm; **^{13}C NMR** (126 MHz, $CDCl_3$) δ 167.6, 142.5, 140.5, 139.7, 136.1, 128.9, 128.8, 128.8, 128.4, 125.5, 124.8, 83.4, 51.7, 47.2, 37.2, 24.4, 24.2, 21.2 ppm; **^{11}B NMR** (128 MHz, $CDCl_3$) δ 32.53 ppm; **HRMS (ESI)** Calculated for $[C_{25}H_{32}BO_4]^+$ ($[M+H]^+$) 407.2388. Found 407.2388; **mp** = 114–116; **$[\alpha]_D^{25}$** = +84.2 (c = 0.40, $CHCl_3$); **HPLC** Phenomenex Lux Cellulose-1 (95:5 *n*-Hexane:2-Propanol, 1 mL/min, 254 nm); t_R (major) = 8.5 min, t_R (minor) = 6.3 min (89:11 e.r.).

methyl 2-methylene-4-phenyl-4-(4,4,5,5-tetramethyl-1,3,2-dioxaborolan-2-yl)-3-(m-tolyl)butanoate

***3t** was prepared following the procedure described in section **F1**.*

59% yield (48 mg, 0.12 mmol) of a 3.4:1 diastereomeric ratio. The two diastereoisomers were readily separated by flash chromatography using *n*-hexane/ethyl acetate = 95:5.

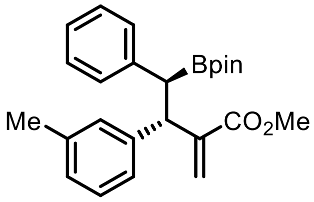

Major diastereomer (3*S*,4*S*)-3t, white solid. **^{1}H NMR** (400 MHz, $CDCl_3$) δ 7.13 – 7.04 (m, 4H), 7.04 – 6.98 (m, 1H), 6.96 – 6.90 (m, 1H), 6.84 – 6.75 (m, 3H), 6.38 (s, 1H), 5.95 (t, J = 0.87 Hz, 1H), 4.36 (dd, J = 12.83, 0.98 Hz, 1H), 3.68 (s, 3H), 2.87 (d, J = 12.76 Hz, 1H), 2.16 (s, 3H), 1.19 (s, 6H), 1.13 (s, 6H) ppm; **^{13}C NMR** (101 MHz, $CDCl_3$) δ 167.3, 144.9, 141.8, 140.0, 137.1, 129.3, 129.0, 128.0, 127.6, 126.8, 125.5, 125.3, 123.7, 83.6, 52.0, 48.5, 38.6, 24.7, 24.5, 21.4 ppm; **^{11}B NMR** (128 MHz, $CDCl_3$) δ 33.41 ppm; **HRMS (ESI)** Calculated for $[C_{25}H_{32}BO_4]^+$ ($[M+H]^+$) 407.2388. Found 407.2402; **mp** = 107–109; **$[\alpha]_D^{25}$** = +150.0 (c =1.43, $CHCl_3$); **HPLC** Phenomenex Lux Cellulose-1 (98:2 *n*-Hexane:2-Propanol, 1 mL/min, 254 nm); t_R (major) = 4.5 min, t_R (minor) = 5.4 min (96:4 e.r.).

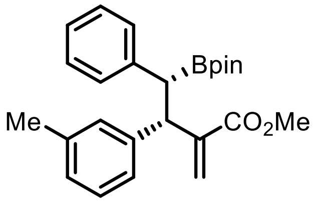

Minor diastereomer (3*S*,4*R*)-3t', white solid. **^{1}H NMR** (400 MHz, $CDCl_3$) δ 7.33 – 7.26 (m, 2H), 7.26 – 7.19 (m, 4H), 7.18 – 7.08 (m, 2H), 6.98 (dddt, J = 7.30, 1.92, 1.31, 0.67 Hz, 1H), 6.10 (d, J = 0.90 Hz, 1H), 5.63 (t, J = 0.96 Hz, 1H), 4.58 (dd, J = 13.00, 0.94 Hz, 1H), 3.57 (s, 3H), 3.14 (d, J = 12.94 Hz, 1H), 2.32 (s, 3H), 0.89 (s, 6H), 0.87 (s, 6H) ppm; **^{13}C NMR** (101 MHz, $CDCl_3$) δ 167.5, 142.6, 142.3, 140.5, 137.6, 129.8, 128.8, 128.4, 128.1, 127.4, 125.9, 125.6, 125.0, 83.4, 51.7, 47.5, 37.3, 24.4, 24.2, 21.6 ppm; **^{11}B NMR** (128 MHz, $CDCl_3$) δ 31.95 ppm; **HRMS (ESI)** Calculated for $[C_{25}H_{32}BO_4]^+$ ($[M+H]^+$) 407.2388. Found 407.2401; **mp** = 85–87; **$[\alpha]_D^{25}$** = +11.0 (c = 0.80, $CHCl_3$); **HPLC** Phenomenex Lux Cellulose-1 (98:2 *n*-Hexane:2-Propanol, 1 mL/min, 254 nm); t_R (major) = 13.7 min, t_R (minor) = 8.7 min (91:9 e.r.).

methyl 2-methylene-4-phenyl-4-(4,4,5,5-tetramethyl-1,3,2-dioxaborolan-2-yl)-3-(o-tolyl)butanoate

***3u** was prepared following the procedure described in section **F1**.*

44% yield (36 mg, 0.09 mmol) of a 4.3:1 diastereomeric ratio. The two diastereoisomers were readily separated by flash chromatography using *n*-hexane/ethyl acetate = 95:5.

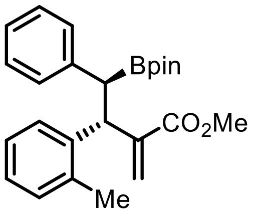

Major diastereomer (3*S*,4*S*)-3u, white solid. **^{1}H NMR** (400 MHz, $CDCl_3$) δ 7.18 (dd, J = 7.88, 1.38 Hz, 1H), 7.12 – 6.97 (m, 6H), 6.92 (td, J = 7.40, 1.43 Hz, 1H), 6.84 – 6.76 (m, 1H), 6.40 (s, 1H), 5.99 (dd, J = 1.21, 0.63 Hz, 1H), 4.63 (dd, J = 12.58, 1.06 Hz, 1H), 3.65 (s, 3H), 2.83 (d, J = 12.61 Hz, 1H), 2.01 (s, 3H), 1.24 (s, 6H), 1.17 (s, 6H) ppm; **^{13}C NMR** (101 MHz, $CDCl_3$) δ 167.2, 145.2, 140.3, 139.6, 137.3, 129.8, 129.7, 127.8, 126.9, 125.8, 125.7, 125.6, 123.8, 83.6, 51.9, 44.0, 39.3, 24.8, 24.6, 19.6 ppm; **^{11}B NMR** (128 MHz, $CDCl_3$) δ 32.34 ppm; **HRMS (ESI)** Calculated for $[C_{25}H_{32}BO_4]^+$ ($[M+H]^+$) 407.2388. Found 407.2389; **mp** = 109–111; **$[\alpha]_D^{25}$** = +146.5 (c =1.44, $CHCl_3$); **HPLC** Phenomenex Lux Cellulose-1 (98:2 *n*-Hexane:2-Propanol, 1 mL/min, 254 nm); t_R (major) = 4.5 min, t_R (minor) = 5.5 min (95:5 e.r.).

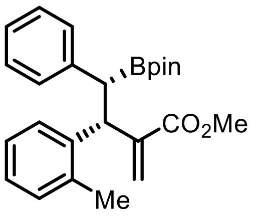

Minor diastereomer (3*S*,4*R*)-3u', white solid. **^{1}H NMR** (400 MHz, $CDCl_3$) δ 7.37 – 7.29 (m, 3H), 7.28 – 7.22 (m, 2H), 7.19 – 7.02 (m, 4H), 6.10 (d, J = 0.87 Hz, 1H), 5.63 (t, J = 0.96 Hz, 1H), 4.78 (dd, J = 12.65, 1.04 Hz, 1H), 3.51 (s, 3H), 3.13 (d, J = 12.58 Hz, 1H), 2.60 (s, 3H), 0.87 (s, 6H), 0.83 (s, 6H) ppm; **^{13}C NMR** (101 MHz, $CDCl_3$) δ 167.8, 142.8, 141.1, 140.5, 137.5, 130.5, 129.1, 128.4, 127.8, 126.5, 126.0, 125.6, 125.1, 83.4, 51.7, 42.9, 38.2, 24.5, 24.0, 20.2 ppm; **^{11}B NMR** (128 MHz, $CDCl_3$) δ 32.37 ppm; **HRMS (ESI)** Calculated for $[C_{25}H_{32}BO_4]^+$ ($[M+H]^+$) 407.2388. Found 407.2399; **mp** = 130–132; **$[\alpha]_D^{25}$** = +46.0 (c = 0.44, $CHCl_3$); **HPLC** Phenomenex Lux Cellulose-1 (98:2 *n*-Hexane:2-Propanol, 1 mL/min, 254 nm); t_R (major) = 13.8 min, t_R (minor) = 21.1 min (88:12 e.r.).

methyl 3-(4-(tert-butyl)phenyl)-2-methylene-4-phenyl-4-(4,4,5,5-tetramethyl-1,3,2-dioxaborolan-2-yl)butanoate

***3v** was prepared following the procedure described in section **F1**.*

66% yield (59 mg, 0.13 mmol) of a 4.6:1 diastereomeric ratio. The two diastereoisomers were readily separated by flash chromatography using *n*-hexane/ethyl acetate = 95:5.

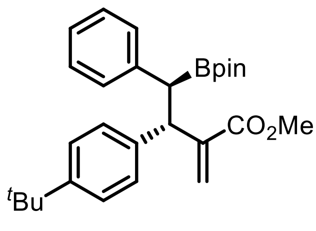

Major diastereomer (3*S*,4*S*)-3v, white solid. **^{1}H NMR** (400 MHz, $CDCl_3$) δ 7.10 – 6.97 (m, 7H), 6.93 – 6.87 (m, 2H), 6.35 (s, 1H), 5.94 (s, 1H), 4.36 (d, *J* = 12.8 Hz, 1H), 3.69 (s, 3H), 2.89 (d, *J* = 12.8 Hz, 1H), 1.21 – 1.16 (m, 15H), 1.12 (s, 6H) ppm; **^{13}C NMR** (101 MHz, $CDCl_3$) δ 167.4, 148.6, 145.1, 140.2, 138.7, 129.3, 128.0, 127.7, 125.4, 124.7, 123.6, 83.6, 52.0, 48.1, 38.6, 34.3, 31.4, 24.7, 24.5 ppm; **^{11}B NMR** (128 MHz, $CDCl_3$) δ 32.44 ppm; **HRMS (ESI)** Calculated for $[C_{28}H_{38}BO_4]^+$ ($[M+H]^+$) 449.2857. Found 449.2870; **mp** = 132–134; **$[\alpha]_D^{25}$** = +69.6 (c = 2.30, $CHCl_3$); **HPLC** Phenomenex Lux Cellulose-2 (98:2 *n*-Hexane:2-Propanol, 1 mL/min, 254 nm); t_R (major) = 3.9 min, t_R (minor) = 4.4 min (91:9 e.r.).

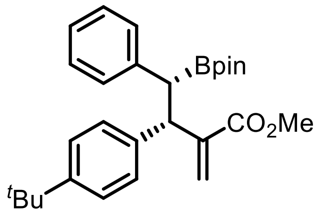

Minor diastereomer (3*S*,4*R*)-3v', white solid. **^{1}H NMR** (400 MHz, $CDCl_3$) δ 7.38 – 7.31 (m, 2H), 7.31 – 7.26 (m, 4H), 7.25 – 7.20 (m, 2H), 7.11 (ddt, *J* = 7.8, 6.6, 1.4 Hz, 1H), 6.09 (d, *J* = 0.9 Hz, 1H), 5.66 (s, 1H), 4.57 (d, *J* = 13.0 Hz, 1H), 3.58 (s, 3H), 3.15 (d, *J* = 13.0 Hz, 1H), 1.27 (s, 9H), 0.87 (s, 6H), 0.81 (s, 6H); **^{13}C NMR** (101 MHz, $CDCl_3$) δ 167.6, 149.5, 142.3, 140.5, 139.7, 128.8, 128.6, 128.4, 125.5, 125.1, 124.7, 83.4, 51.8, 47.1, 37.3, 31.5, 24.5, 24.1 ppm; **^{11}B NMR** (128 MHz, $CDCl_3$) δ 32.53 ppm; **HRMS (ESI)** Calculated for $[C_{28}H_{38}BO_4]^+$ ($[M+H]^+$) 449.2857. Found 449.2879; **mp** = 167–169; **$[\alpha]_D^{25}$** = +23.6 (c = 0.65, $CHCl_3$); **HPLC** Phenomenex Lux Cellulose-1 (95:5 *n*-Hexane:2-Propanol, 1 mL/min, 254 nm); t_R (major) = 7.5 min, t_R (minor) = 4.1 min (74:26 e.r.).

methyl 3-(2-fluorophenyl)-2-methylene-4-phenyl-4-(4,4,5,5-tetramethyl-1,3,2-dioxaborolan-2-yl)butanoate

***3w** was prepared following the procedure described in section **F1**.*

57% yield (47 mg, 0.11 mmol) of a 2.5:1 diastereomeric ratio. The two diastereoisomers were readily separated by flash chromatography using *n*-hexane/ethyl acetate = 90:10.

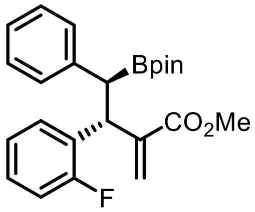

Major diastereomer (3*R*,4*S*)-3w, white solid. **^{1}H NMR** (400 MHz, $CDCl_3$) δ 7.14 – 7.04 (m, 5H), 7.03 – 6.98 (m, 1H), 6.97 – 6.92 (m, 1H), 6.88 (td, *J* = 7.4, 1.3 Hz, 1H), 6.71 (ddd, *J* = 10.5, 8.1, 1.3 Hz, 1H), 6.42 (s, 1H), 6.02 (s, 1H), 4.72 (d, *J* = 12.8 Hz, 1H), 3.67 (s, 3H), 3.00 (d, ^1J = 12.9 Hz, 1H), 1.18 (s, 6H), 1.13 (s, 6H) ppm; **^{13}C NMR** (101 MHz, $CDCl_3$) δ 167.0, 161.09 (d, $^1J_{CF}$ = 246.5 Hz), 143.4, 139.5, 129.8 (d, $^3J_{CF}$ = 4.6 Hz), 129.2, 129.0 (d, $^2J_{CF}$ = 14.1 Hz) 128.1, 127.7 (d, $^3J_{CF}$ = 8.4 Hz), 125.7, 124.7, 123.6 (d, $^4J_{CF}$ = 3.4 Hz), 115.1 (d, $^2J_{CF}$ = 23.2 Hz), 83.7, 52.0, 42.2, 37.1, 24.7, 24.5 ppm; **^{19}F NMR** (376 MHz, $CDCl_3$) δ –116.73 (dt, *J* = 11.8, 6.1 Hz) ppm; **^{11}B NMR** (128 MHz, $CDCl_3$) δ 32.52 ppm; **HRMS (ESI)** Calculated for $[C_{24}H_{29}BFO_4]^+$ ($[M+H]^+$) 411.2137. Found 411.2155; **mp** = 100–102; **$[\alpha]_D^{25}$** = +78.2 (c = 1.05, $CHCl_3$); **HPLC** Phenomenex Lux Cellulose-2 (98:2 *n*-Hexane:2-Propanol, 1 mL/min, 275 nm); t_R (major) = 4.8 min, t_R (minor) = 5.6 min (91:9 e.r.).

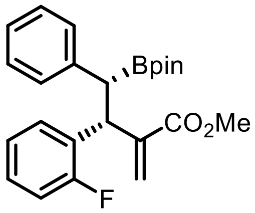

Minor diastereomer (3*R*,4*R*)-3w', white solid. **^{1}H NMR** (400 MHz, $CDCl_3$) δ 7.49 (td, *J* = 7.6, 1.8 Hz, 1H), 7.33 – 7.29 (m, 2H), 7.27 – 7.21 (m, 2H), 7.20 – 7.05 (m, 3H), 6.99 (ddd, *J* = 10.4, 8.1, 1.3 Hz, 1H), 6.11 (s, 1H), 5.66 (q, *J* = 1.0 Hz, 1H), 4.81 (d, *J* = 13.0 Hz, 1H), 3.56 (s, 3H), 3.32 (d, *J* = 13.0 Hz, 1H), 0.90 (s, 6H), 0.87 (s, 6H) ppm; **^{13}C NMR** (101 MHz, $CDCl_3$) δ 167.2, 161.1 (d, $^1J_{CF}$ = 247.6 Hz), 140.9, 140.1, 130.5 (d, $^3J_{CF}$ = 4.1 Hz), 129.9 (d, $^2J_{CF}$ = 14.0 Hz), 129.0, 128.5, 128.3 (d, $^3J_{CF}$ = 8.4 Hz), 126.5, 125.8, 123.9 (d, $^4J_{CF}$ = 3.6 Hz), 115.6 (d, $^2J_{CF}$ = 23.1 Hz), 83.5, 51.7, 41.7, 35.8, 24.3, 24.2. ppm; **^{19}F NMR** (376 MHz, $CDCl_3$) δ –115.15 (dt, *J* = 11.8, 5.9 Hz) ppm; **^{11}B NMR** (128 MHz, $CDCl_3$) δ 32.69 ppm; **HRMS (ESI)** Calculated for $[C_{24}H_{29}BFO_4]^+$ ($[M+H]^+$) 411.2137. Found 411.2141; **mp** = 110–112; **$[\alpha]_D^{25}$** = +35.0 (c = 0.60, $CHCl_3$); **HPLC** Phenomenex Lux Cellulose-1 (98:2 *n*-Hexane:2-Propanol, 1 mL/min, 254 nm); t_R (major) = 13.1 min, t_R (minor) = 11.4 min (85:15 e.r.).

methyl 2-methylene-4-phenyl-3-(pyridin-2-yl)-4-(4,4,5,5-tetramethyl-1,3,2-dioxaborolan-2-yl)butanoate

***3x** was prepared following the procedure described in section **F1**.*

86% yield (68 mg, 0.17 mmol) of a 1.5:1 diastereomeric ratio. The two diastereoisomers were readily separated by flash chromatography using *n*-hexane/ethyl acetate = 20:80.

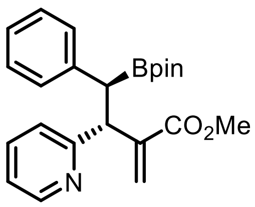

Major diastereomer (3*R*,4*S*)-3x, brown oil. **^{1}H NMR** (500 MHz, $CDCl_3$) δ 8.59 (ddd, *J* = 5.3, 1.7, 0.9 Hz, 1H), 7.58 (tt, *J* = 7.6, 2.0 Hz, 1H), 7.19 – 7.15 (m, 1H), 7.12 (d, *J* = 7.9 Hz, 1H), 7.08 – 7.03 (m, 2H), 7.00 – 6.93 (m, 3H), 6.32 (s, 1H), 5.67 (s, 1H), 4.72 (d, *J* = 10.5 Hz, 1H), 3.64 (s, 3H), 3.05 (d, *J* = 10.6 Hz, 1H), 1.15 (s, 6H), 1.13 (s, 6H) ppm; **^{13}C NMR** (101 MHz, $CDCl_3$) δ 167.4, 161.9, 146.0, 141.7, 141.3, 138.1, 129.8, 127.8, 126.5, 125.0, 124.5, 122.1, 82.0, 52.0, 50.1, 40.6, 25.3, 25.3 ppm; **^{11}B NMR** (128 MHz, $CDCl_3$) δ 23.71 ppm; **HRMS (ESI)** Calculated for $[C_{23}H_{29}BNO_4]^+$ ($[M+H]^+$) 394.2184. Found 394.2180; **$[\alpha]_D^{25}$** = +37.3 (c = 1.18, $CHCl_3$); **HPLC** Phenomenex Lux Cellulose-1 (85:15 *n*-Hexane:2-Propanol, 1 mL/min, 254 nm); t_R (major) = 5.6 min, t_R (minor) = 6.5 min (88:12 e.r.).

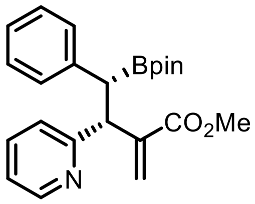

Minor diastereomer (3*R*,4*R*)-3x', brown oil. **^{1}H NMR** (500 MHz, $CDCl_3$) δ 8.65 (d, *J* = 5.6 Hz, 1H), 7.92 (t, *J* = 7.8 Hz, 1H), 7.47 (t, *J* = 6.9 Hz, 1H), 7.30 (dt, *J* = 17.2, 8.7 Hz, 3H), 7.21 (t, *J* = 7.8, 2H), 7.07 (t, *J* = 6.6, 1H), 6.28 (s, 1H), 5.66 (s, 1H), 4.69 (d, *J* = 11.5 Hz, 1H), 3.60 (s, 3H), 2.63 (d, *J* = 11.4, 1H), 1.06 (s, 6H), 0.90 (s, 6H) ppm; **^{13}C NMR** (101 MHz, $CDCl_3$) δ 174.6, 166.8, 163.1, 143.3, 141.1, 141.1, 139.6, 129.2, 129.1, 127.6, 124.5, 123.1, 122.8, 80.2, 52.2, 51.9, 46.0, 27.0, 25.9 ppm; **^{11}B NMR** (128 MHz, $CDCl_3$) δ 12.51 ppm; **HRMS (ESI)** Calculated for $[C_{23}H_{29}BNO_4]^+$ ($[M+H]^+$) 394.2184. Found 394.2179; **$[\alpha]_D^{25}$** = –50.8 (c = 0.94, $CHCl_3$); **HPLC** Phenomenex Lux Cellulose-1 (85: 15 *n*-Hexane:2-Propanol, 1 mL/min, 254 nm); t_R (major) = 7.6 min, t_R (minor) = 6.1 min (75:25 e.r.).

ethyl 2-methylene-3,4-diphenyl-4-(4,4,5,5-tetramethyl-1,3,2-dioxaborolan-2-yl)butanoate

***3y** was prepared following the procedure described in section **F1**.*

58% yield (47 mg, 0.12 mmol) of a 4.1:1 diastereomeric ratio. The two diastereoisomers were readily separated by flash chromatography using *n*-hexane/ethyl acetate = 98:2.

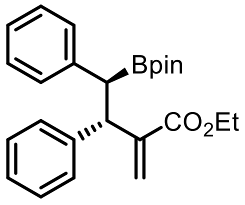

Major diastereomer (3*S*,4*S*)-3y, white solid. **^{1}H NMR** (500 MHz, $CDCl_3$) δ 7.33 – 7.09 (m, 10H), 6.58 (s, 1H), 6.15 (s, 1H), 4.57 (d, *J* = 12.9 Hz, 1H), 4.46 – 4.13 (m, 2H), 3.07 (d, *J* = 12.8 Hz, 1H), 1.45 – 1.34 (m, 9H), 1.32 (s, 6H) ppm; **^{13}C NMR** (101 MHz, $CDCl_3$) δ 166.7, 145.1, 142.0, 140.0, 129.3, 128.3, 128.1, 127.8, 126.0, 125.5, 123.4, 83.6, 60.8, 48.8, 38.6, 24.7, 24.5, 14.2 ppm; **^{11}B NMR** (128 MHz, $CDCl_3$) δ 32.42 ppm; **HRMS (ESI)** Calculated for $[C_{25}H_{32}BO_4]^+$ ($[M+H]^+$) 407.2388. Found 407.2391; **mp** = 72–75; **$[\alpha]_D^{25}$** = +177.1 (c = 1.76, $CHCl_3$); **HPLC** Phenomenex Lux Cellulose-1 (98:2 *n*-Hexane:2-Propanol, 1 mL/min, 254 nm); t_R (major) = 4.5 min, t_R (minor) = 5.0 min (93:7 e.r.).

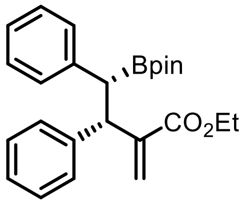

Minor diastereomer (3*S*,4*R*)-3y', white solid. **^{1}H NMR** (400 MHz, $CDCl_3$) δ 7.45 – 7.39 (m, 2H), 7.32 – 7.21 (m, 6H), 7.19 – 7.14 (m, 1H), 7.14 – 7.08 (m, 1H), 6.09 (d, *J* = 0.9 Hz, 1H), 5.62 (t, *J* = 1.0 Hz, 1H), 4.61 (d, *J* = 13.0 Hz, 1H), 4.08 – 3.94 (m, 2H), 3.18 (d, *J* = 12.9 Hz, 1H), 1.16 (t, *J* = 7.1 Hz, 3H), 0.88 (s, 6H), 0.86 (s, 6H) ppm; **^{13}C NMR** (101 MHz, $CDCl_3$) δ 167.1, 142.9, 142.6, 140.5, 129.0, 128.8, 128.4, 128.2, 126.6, 125.6, 124.8, 83.4, 60.6, 47.6, 37.1, 24.4, 24.2, 14.2 ppm; **^{11}B NMR** (128 MHz, $CDCl_3$) δ 32.40 ppm; **HRMS (ESI)** Calculated for $[C_{25}H_{32}BO_4]^+$ ($[M+H]^+$) 407.2388. Found 407.2393; **mp** = 97–99; **$[\alpha]_D^{25}$** = +77.6 (c = 0.57, $CHCl_3$); **HPLC** Phenomenex Lux Cellulose-1 (98:2 *n*-Hexane:2-Propanol, 1 mL/min, 254 nm); t_R (major) = 8.5 min, t_R (minor) = 13.0 min (92:8 e.r.).

hexyl 2-methylene-3,4-diphenyl-4-(4,4,5,5-tetramethyl-1,3,2-dioxaborolan-2-yl)butanoate

***3z** was prepared following the procedure described in section **F1**.*

50% yield (46 mg, 0.10 mmol) of a 3.1:1 diastereomeric ratio. The two diastereoisomers were readily separated by flash chromatography using *n*-hexane/ethyl acetate = 95:5.

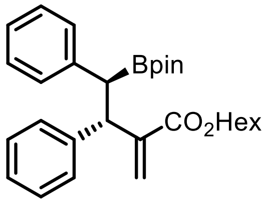

Major diastereomer (3*S*,4*S*)-3z, white solid. **^{1}H NMR** (400 MHz, $CDCl_3$) δ 7.11 – 6.93 (m, 10H), 6.38 (s, 1H), 5.94 (s, 1H), 4.36 (d, *J* = 12.8 Hz, 1H), 4.16 – 3.97 (m, 2H), 2.86 (d, *J* = 12.8 Hz, 1H), 1.61 – 1.51 (m, 2H), 1.29 – 1.22 (m, 6H), 1.18 (s, 6H), 1.12 (s, 6H), 0.87 (t, *J* = 6.8 Hz, 3H). ppm; **^{13}C NMR** (101 MHz, $CDCl_3$) *δ* 166.9, 145.1, 142.0, 140.0, 129.3, 128.3, 128.1, 127.8, 126.0, 125.5, 123.5, 83.6, 65.0, 48.8, 38.7, 31.5, 28.6, 25.7, 24.7, 24.5, 22.7, 14.1 ppm; **^{11}B NMR** (128 MHz, $CDCl_3$) δ 34.83 ppm; **HRMS (ESI)** Calculated for $[C_{29}H_{40}BO_4]^+$ ($[M+H]^+$) 463.3014. Found 463.3024; **mp** = 58–60; **$[\alpha]_D^{25}$** = +138.0 (c = 1.22, $CHCl_3$); **HPLC** Phenomenex Lux Amylose-1 (98:2 *n*-Hexane:2-Propanol, 1 mL/min, 254 nm); t_R (major) = 4.8 min, t_R (minor) = 4.1 min (93:7 e.r.).

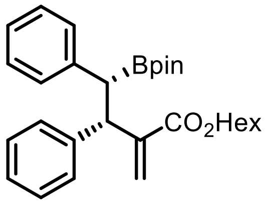

Minor diastereomer (3*S*,4*R*)-3z', yellowish solid. **^{1}H NMR** (400 MHz, $CDCl_3$) *δ* 7.45 – 7.39 (m, 2H), 7.31 – 7.23 (m, 6H), 7.18 – 7.08 (m, 2H), 6.09 (d, *J* = 0.9 Hz, 1H), 5.61 (s, 1H), 4.60 (d, *J* = 12.8 Hz, 1H), 4.01 – 3.90 (m, 2H), 3.17 (d, *J* = 12.9 Hz, 1H), 1.55 – 1.48 (m, 2H), 1.27 – 1.21 (m, 6H), 0.89 – 0.84 (m, 15H) ppm; **^{13}C NMR** (101 MHz, $CDCl_3$) δ 167.2, 142.9, 142.6, 140.5, 129.0, 128.8, 128.4, 128.3, 128.2, 126.6, 125.6, 124.8, 83.4, 64.9, 47.6, 31.6, 28.6, 25.7, 24.4, 24.2, 22.6, 14.1 ppm; **^{11}B NMR** (128 MHz, $CDCl_3$) δ 31.90 ppm; **HRMS (ESI)** Calculated for $[C_{29}H_{40}BO_4]^+$ ($[M+H]^+$) 463.3014. Found 463.3024; **mp** = 59–61; **$[\alpha]_D^{25}$** = +59.5 (c = 0.53, $CHCl_3$); **HPLC** Phenomenex Lux Cellulose-1 (98:2 *n*-Hexane:2-Propanol, 1 mL/min, 254 nm); t_R (major) = 7.1 min, t_R (minor) = 7.7 min (86:14 e.r.).

G. Mechanistic Studies

G.1. Identification of the Si-F interaction – ^{19}F, ^{1}H and ^{11}B NMR Titrations

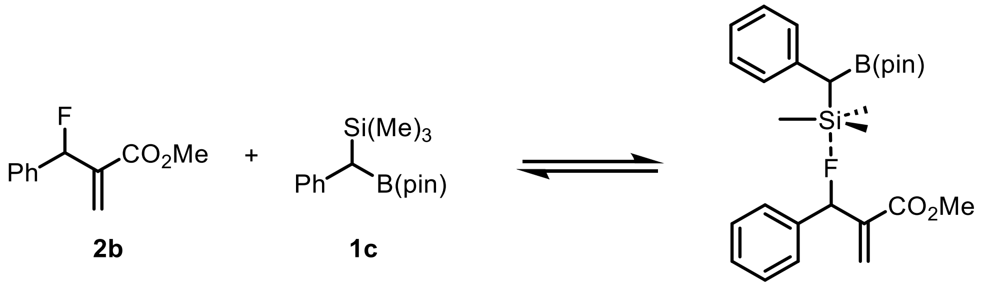

Procedure for the ^{19}F NMR titration of 2b with 1c

In an NMR tube, 0.1 mmol of **2b** were dissolved in 0.5 mL of THF_d_8, and a ^{19}F NMR spectrum was acquired to measure the initial point. Subsequently, increasing amounts of **1c** were added to the NMR tube, and after each addition a ^{19}F NMR spectrum was acquired.

The spectra were referenced through an external shift referencing the residual peak of the corresponding deuterated solvent in the ^{1}H NMR spectra by using the xiref command.

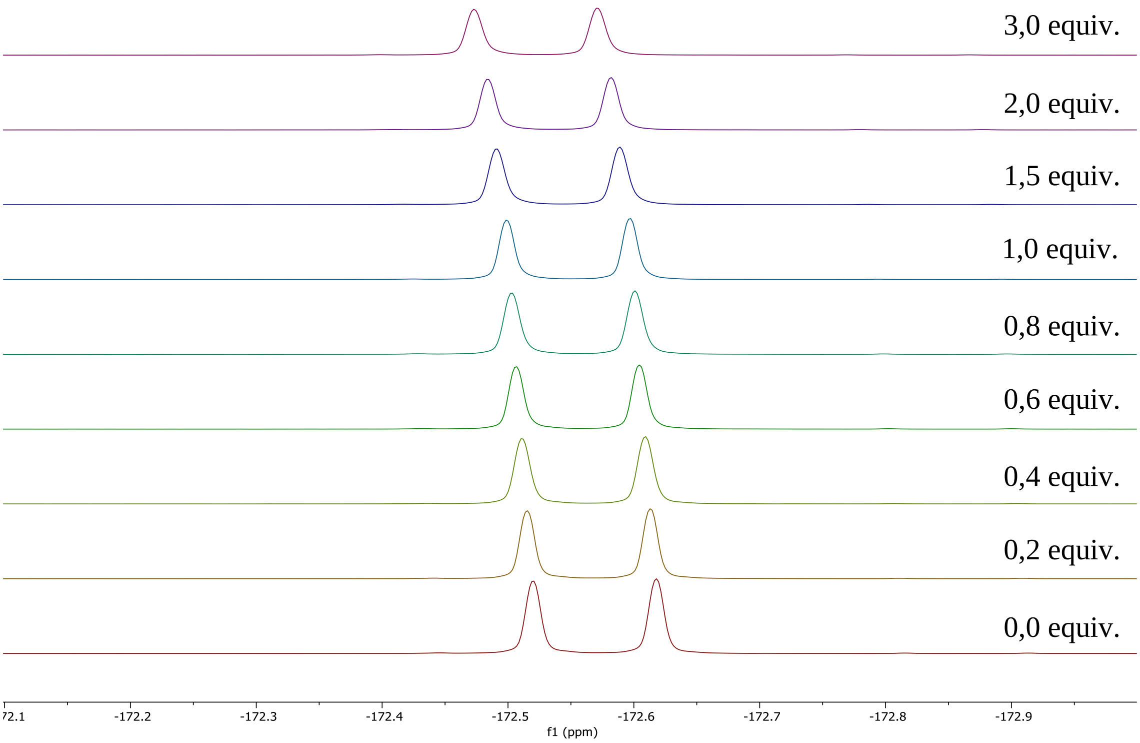

Figure S2. ^{19}F NMR spectra at 470 MHz NMR field in THF_d_8 of **2b** (bottom) with increasing amounts of **1c** (from 0.2 to 3 equiv.)

*[The shift of the ^{19}F resonance of **2b** in the presence of increasing amounts of **1c** suggests the formation of intermediate **14** via a Si-F interaction.]*

Procedure for the titration of 1c with 2b

In an NMR quartz tube, 0.1 mmol of **1c** were dissolved in 0.5 mL of THF_d$_8$, and a ^{1}H and ^{11}B NMR spectrum were acquired to measure the initial point. Subsequently, increasing amounts of **2b** were added to the NMR tube, and after each addition a ^{1}H and ^{11}B NMR spectrum were acquired.

^{1}H NMR Titration

The spectra were referenced through an external shift referencing the residual peak of the corresponding deuterated solvent in the ^{1}H NMR spectra.

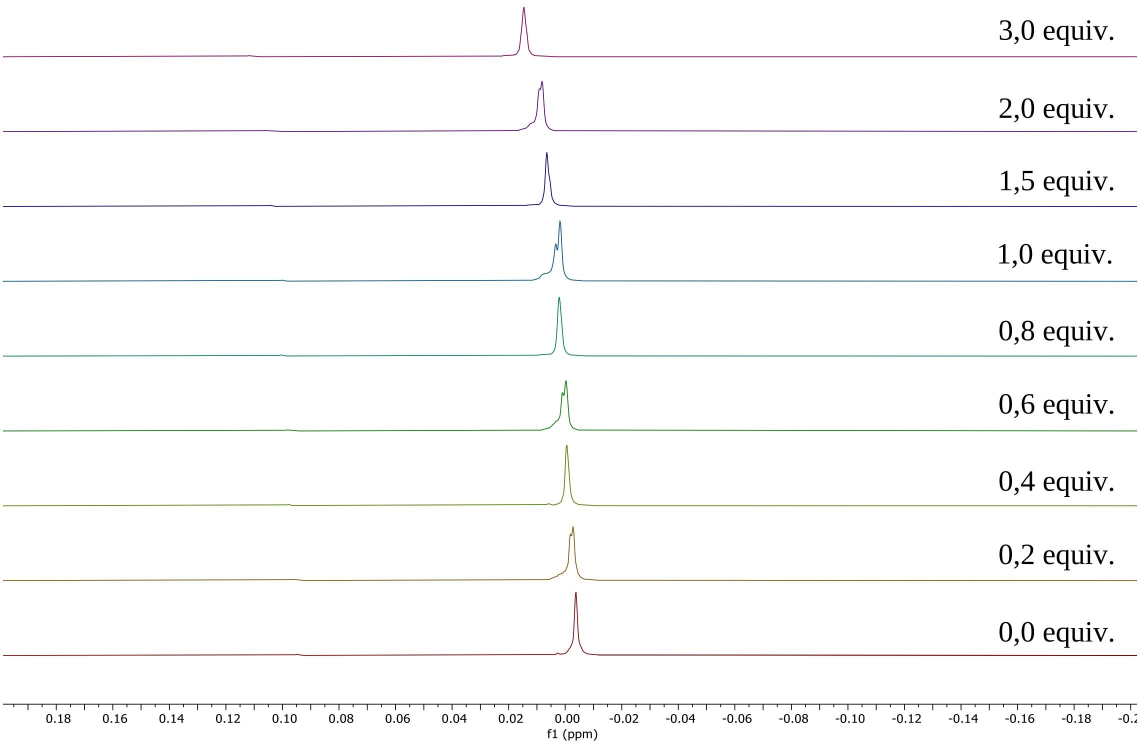

Figure S3. ^{1}H NMR spectra at 500 MHz NMR field in THF_d$_8$ of **1c** (bottom) with increasing amounts of **2b** (from 0.2 to 3 equiv.)

*[The shift of the ^{1}H resonance of the trimethylsilane moiety of **1c** in the presence of increasing amounts of **2b** suggests the formation of intermediate **14** via a Si-F interaction.]*

^11^B NMR Titration

The spectra were referenced through an external shift referencing the residual peak of the corresponding deuterated solvent in the ^{1}H NMR spectra by using the xiref command.

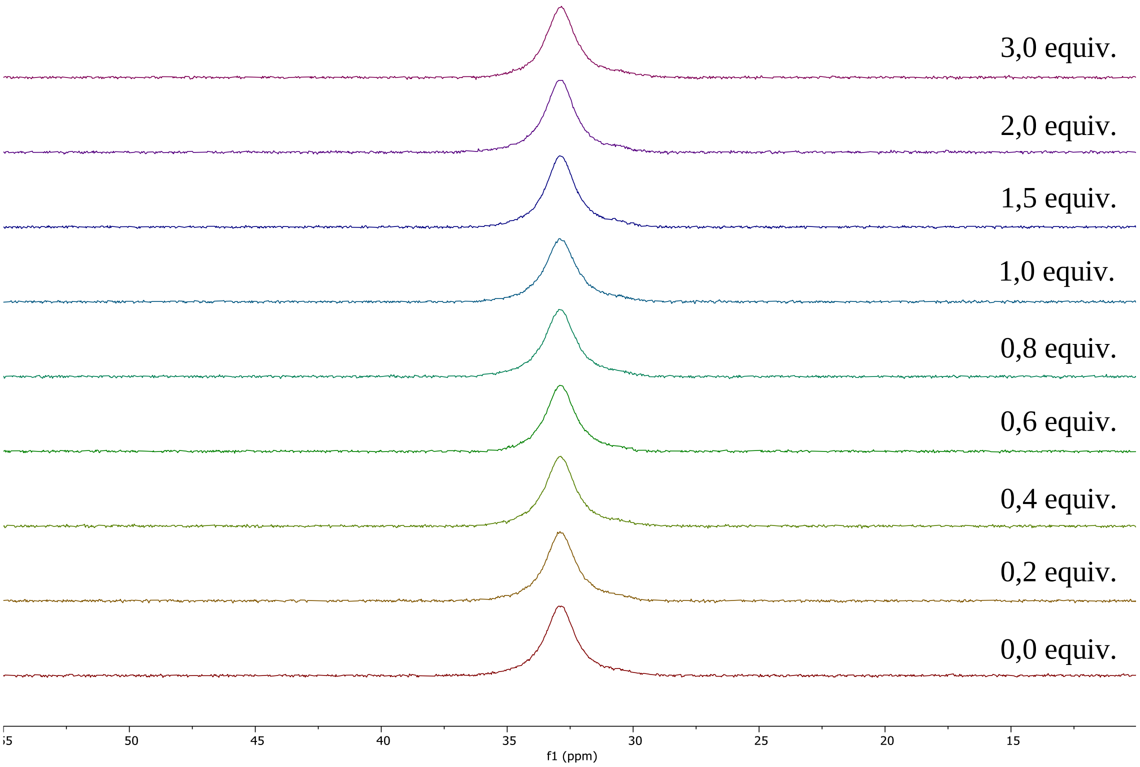

Figure S4. ^{11}B NMR spectra at 160 MHz NMR field in THF_d_8 of **1c** (bottom) with increasing amounts of **2b** (from 0.2 to 3 equiv.)

*[The absence of shift in the ^{11}B resonance of **1c** in the presence of increasing amounts of **2b** suggests the absence of a B-F interaction.]*

G.2. Desilylation of 1c

Procedure for the desilylation of 1c with TBAF

0.1 mmol of **1c** was dissolved in 0.5 mL of THF_d$_8$ in an NMR quartz tube. ^{1}H and ^{11}B NMR spectra were acquired to measure the initial point. 0.05 mmol (0.5 equiv.) of tetrabutylammonium fluoride (TBAF) 1.0 M in THF were added to the NMR tube, and ^{1}H, ^{11}B and ^{19}F NMR spectra of the resulting sample were subsequently recorded.

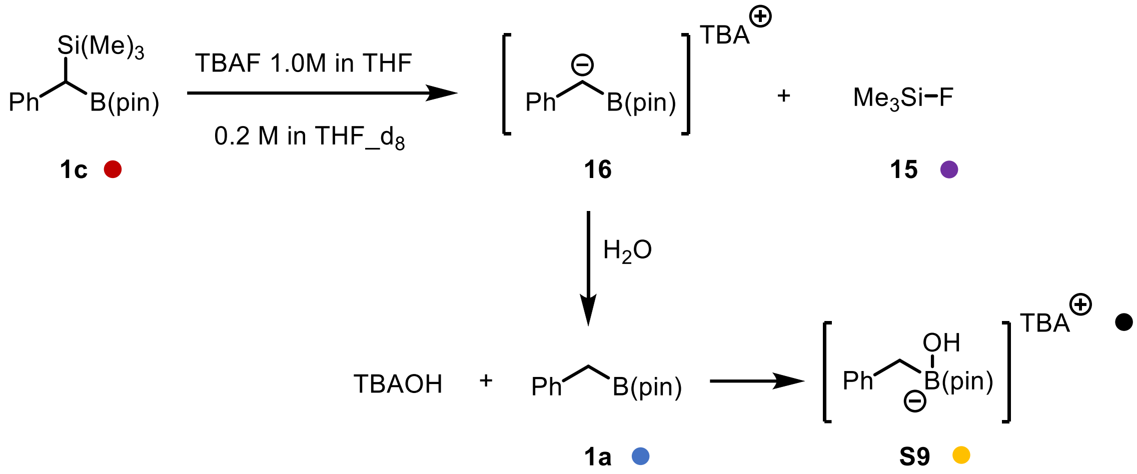

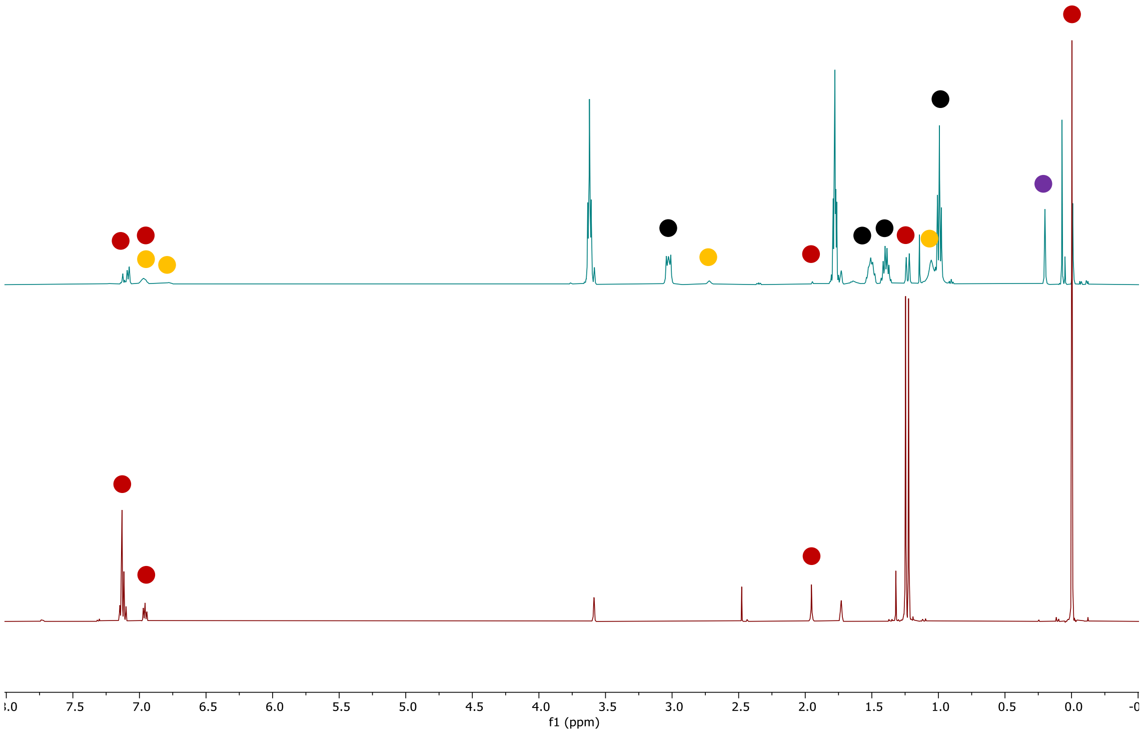

Figure S5. ^{1}H NMR spectra at 500 MHz NMR field in THF_d$_8$ of **1c** (bottom) with 0.5 equivalents of TBAF (top).

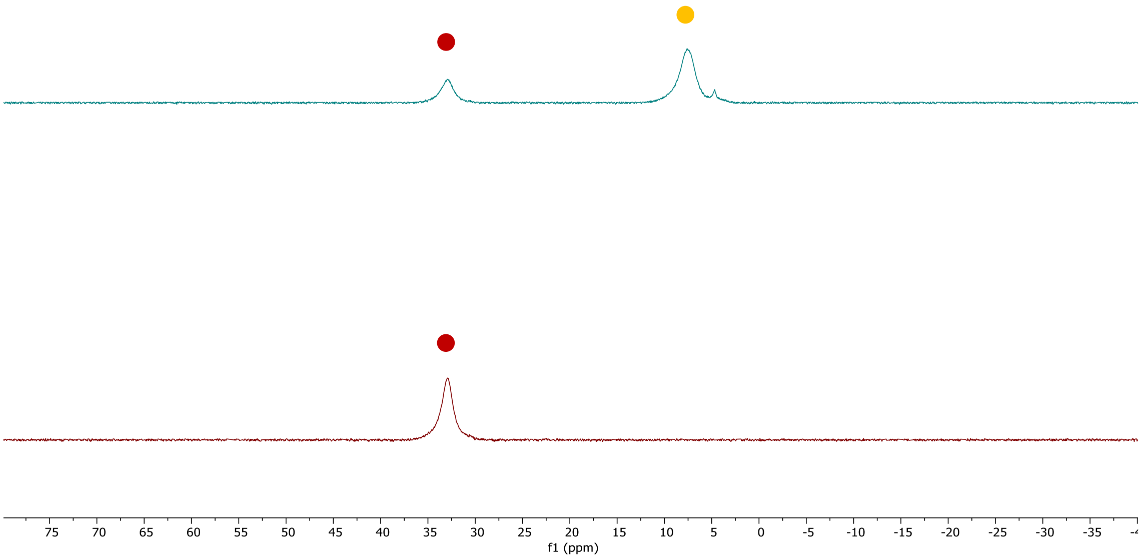

Figure S6. ^{11}B NMR spectra at 160 MHz NMR field in THF_d$_8$ of **1c** (bottom) with 0.5 equivalents of TBAF (top).

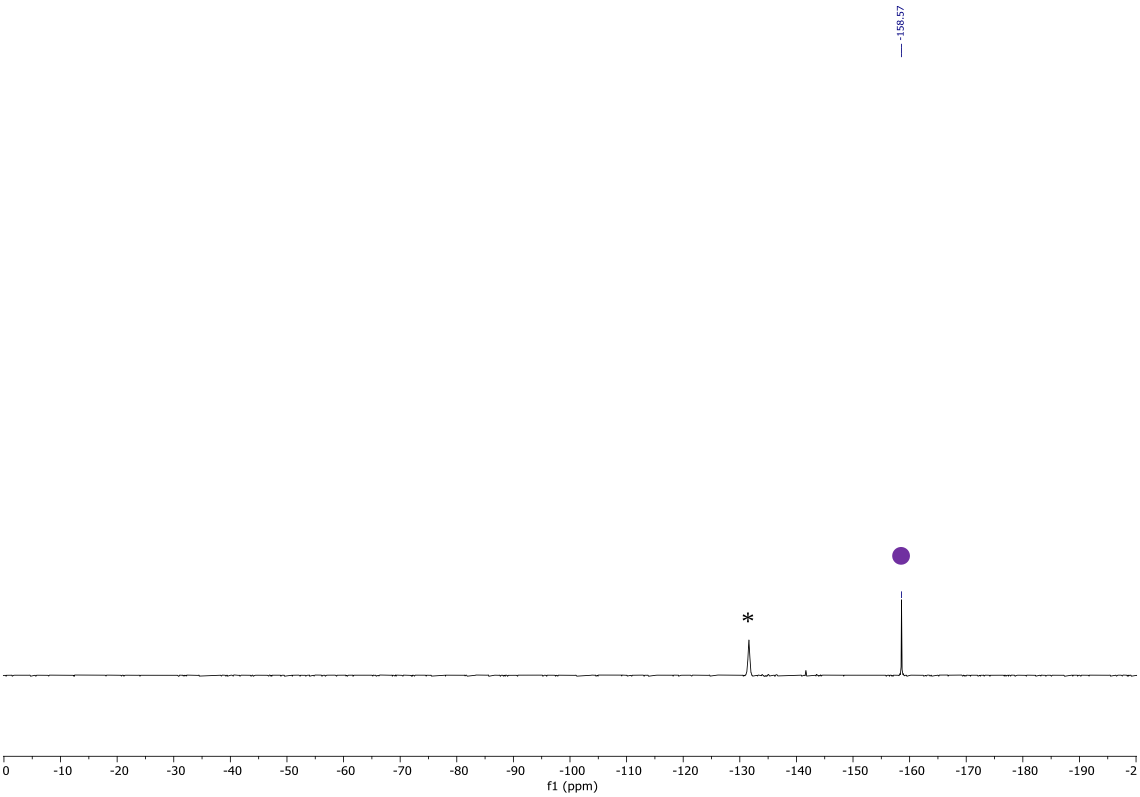

Figure S7. ^{19}F NMR spectra at 470 MHz NMR field in THF_d$_8$ of **1c** with 0.5 equivalents of TBAF. (*Unknown species).

*When **1c** (red dots) was treated with TBAF 1.0 M in THF (black dots), desilylation of **1c** occurred in fast and irreversible manner. As a result, Me_3Si-F **15** (purple dots) was formed and detected by ^1H (δ = 0.2 ppm) and ^{19}F NMR (δ = -158.6 ppm). However, the α-boryl carbanion **16** could not be detected.[6a] This observation is due to the fact that anhydrous tetrabutylammonium fluoride is not stable in THF solution and, as a consequence, commercially available TBAF solutions contain ~5% of water (removal of this water results in decomposition of TBAF by Hofmann elimination).[6b-c] Therefore, the water present in the TBAF solution rapidly protonates species **16** to form benzylboronic acid pinacol ester **1a** and tetrabutylammonium hydroxide (TBAOH). Subsequently, **1a** reacted with hydroxyl anions to generate the "ate" complex **S9** (yellow dots), which was identified by ^{11}B NMR (δ = 7.6 ppm).*

Blank experiment of 1b with TBAF

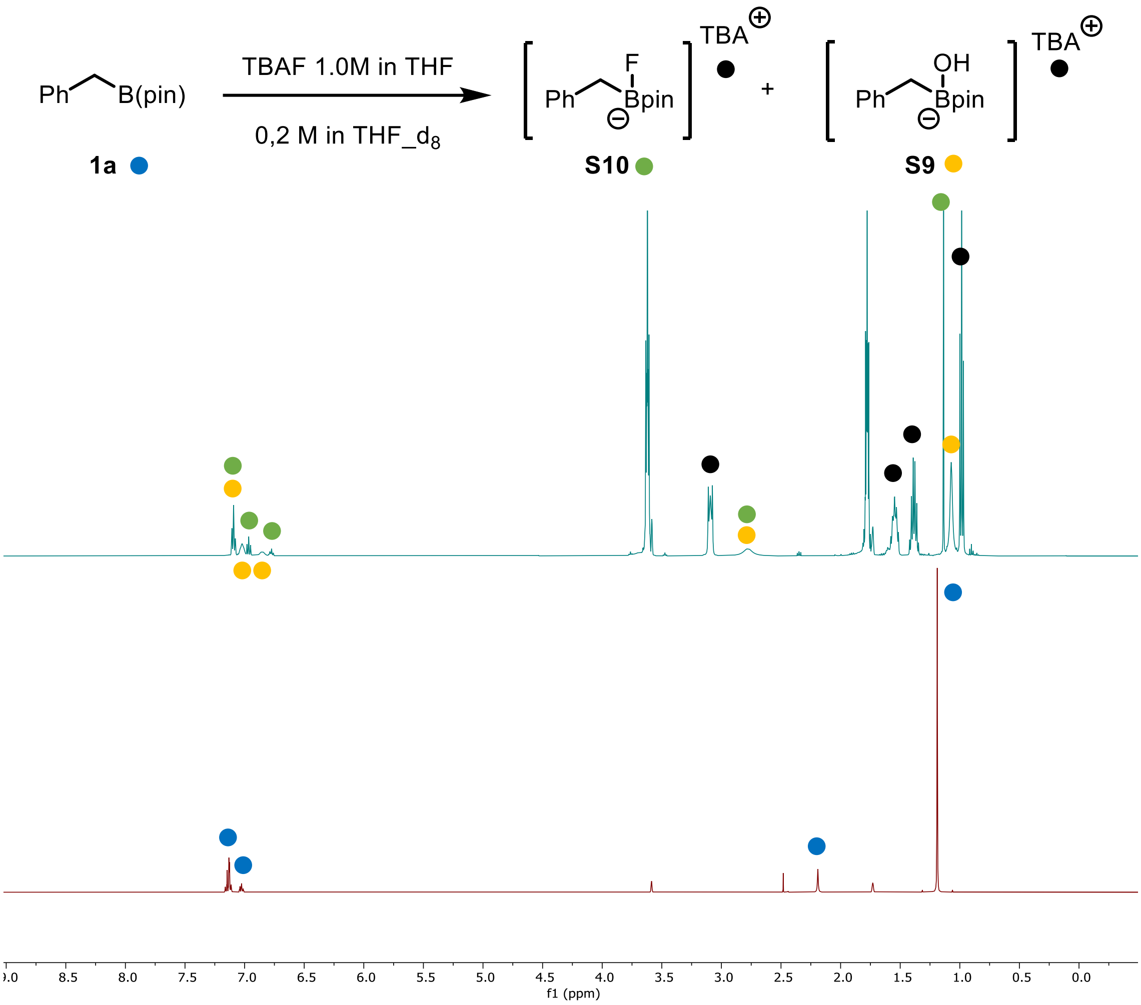

Figure S8. ^1H NMR spectra at 500 MHz NMR field in THF_d_8 of **1a** (bottom) with 0.5 equivalent of TBAF (top, after 10 min from the addition).

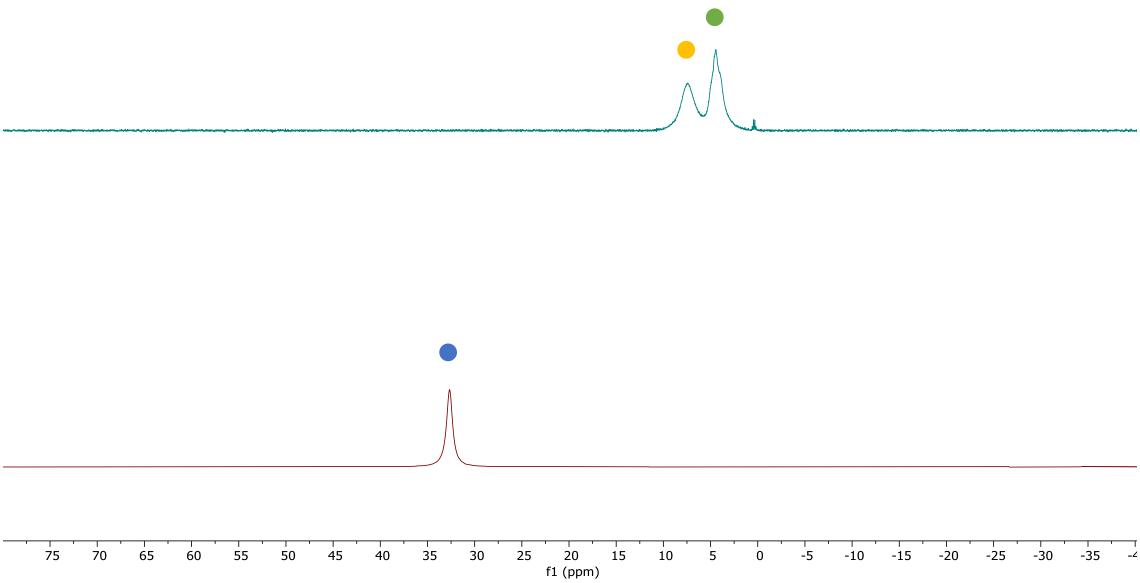

Figure S9. ^{11}B NMR spectra at 160 MHz NMR field in THF_d$_8$ of **1a** (bottom) with 0.5 equivalent of TBAF (top, after 10 min from the addition).

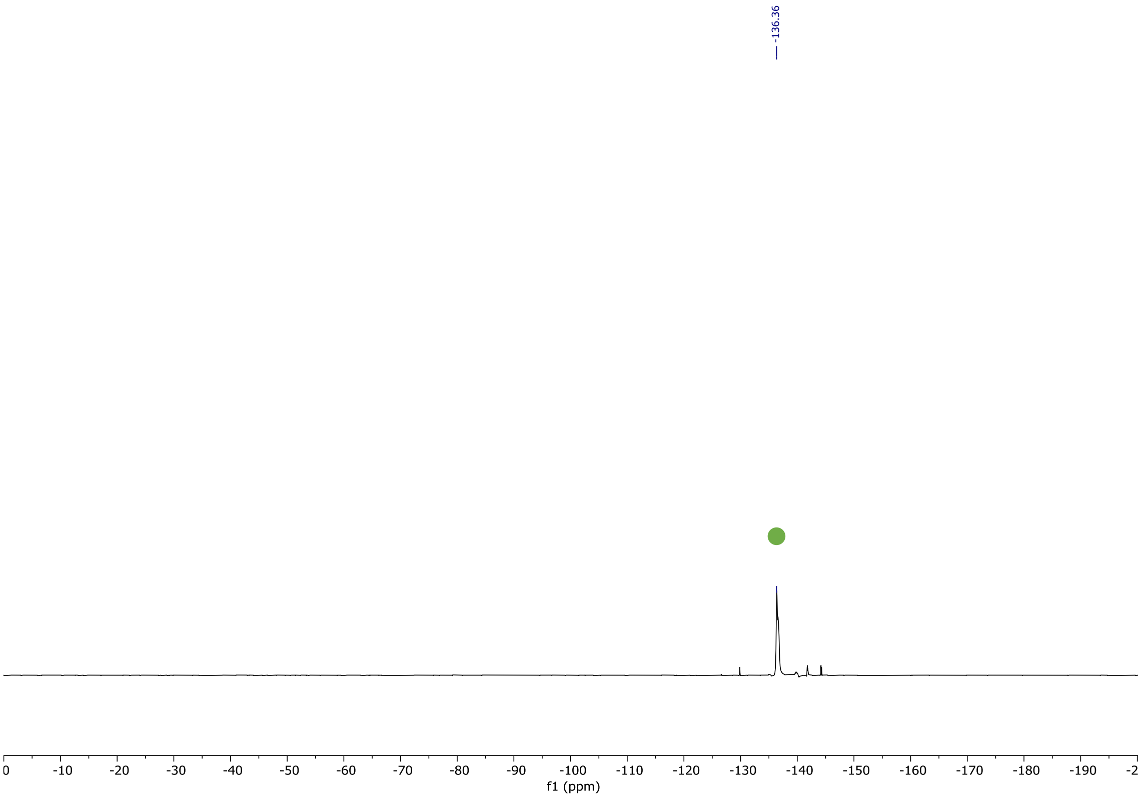

Figure S10. ^{19}F NMR spectra at 470 MHz NMR field in THF_d$_8$ of **1a** with 0.5 equivalent of TBAF (10 min from the addition).

*When **1a** (blue dots) was treated with 0.5 equivalents of TBAF 1.0 M in THF (black dots), the "ate" complex **S10** (green dots) was formed and detected by ^{1}H, ^{11}B (δ = 4.4 ppm) and ^{19}F NMR (δ = -136.4 ppm). However, as TBAF in THF solution contains water,[6d] the "ate" complex **S9** (yellow dots) was also formed and detected by ^{1}H and ^{11}B NMR (δ = 7.6 ppm), as seen before.*

Blank experiment of 1a with TBAOH

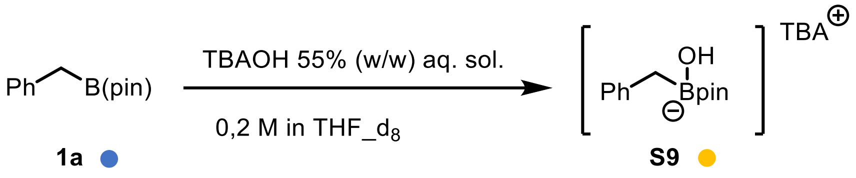

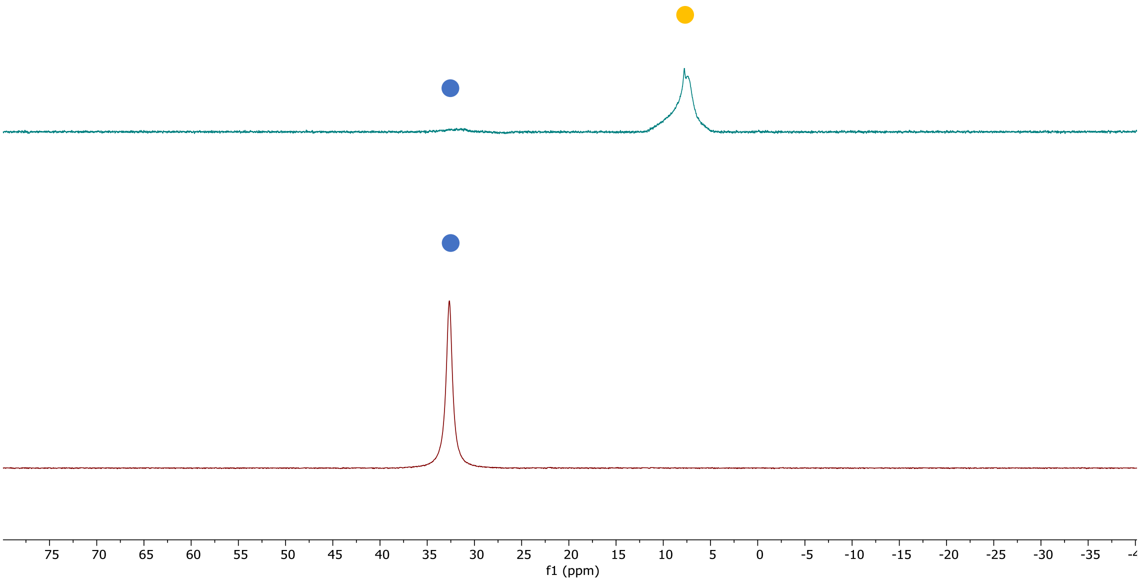

Figure S11. ^{11}B NMR spectra at 160 MHz NMR field in THF_d$_8$ of **1a** (bottom) with 0.5 equivalent of TBAOH (top, after 10 min from the addition).

*When **1a** (blue dots) was treated with 0.5 equivalents of TBAOH 55% (w/w) aqueous solution, the "ate" complex **S9** (yellow dots) was formed and identified by ^{11}B NMR (δ = 7.6 ppm).*

G.3. S$_N$2'–S$_N$2' Mechanism – Blank reactions

Effect of H_2O in the reaction outcome

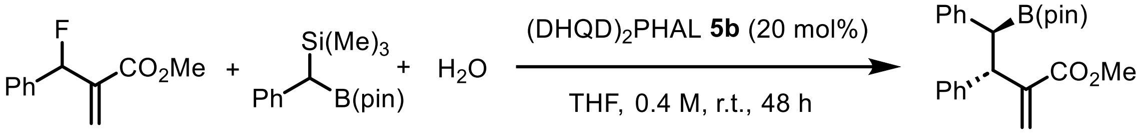

(rac)-**2b**
(2 equiv.)

(rac)-**1c**
(1 equiv.)

3a

Entry	Equiv. of H_2O	Conv. (%)	yield 3a (%)[a]	yield 1a (%)[a]
**1**	none	95	77	5
**2**	1	92	16	49
**3**	5	94	5	68

Table S7. Reactivity under optimized conditions in the presence of H_2O. [a] Determined by ^{1}H NMR spectroscopy using 1,3,5-trimethoxybenzene as the internal standard.

*[When the optimized reaction was run in the presence of H_2O, the yield of product **3a** decreased significantly in favour of $PhCH_2Bpin$ (**1a**) in 49% and 68% yield, respectively. This observation is consistent with the formation of the α-boryl carbanion species, which is protonated in the presence of H_2O to furnish **1a**].*

Effect of Lithium salts in the reaction outcome

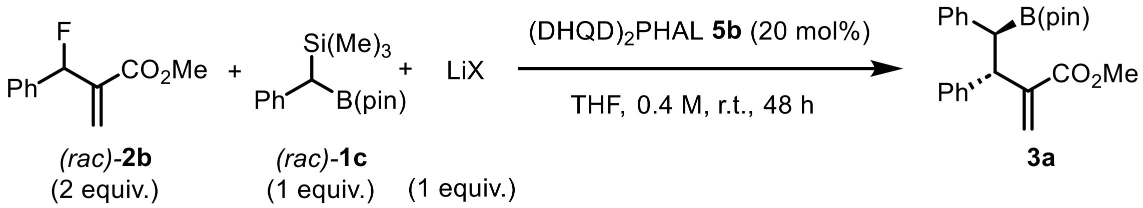

Entry	Lithium salt	Conv. (%)	yield 3a (%)[a]
**1**	none	95	77
**2**	LiCl	0	0
**3**	$LiClO_4$	0	0

Table S8. Reactivity under optimized conditions in the presence of Li salts. [a] Determined by ^{1}H NMR spectroscopy using 1,3,5-trimethoxybenzene as the internal standard.

*[When the optimized reaction was run in the presence of LiCl and $LiClO_4$, the reaction did not proceed. The ability of lithium ions to interact with fluorine in C-F bonds[4b, 7] ampers the formation of intermediate **14** via Si-F interaction. This experiment evidences the crucial role of the Si-F interaction in the reactivity.]*

Reactivity of allyl α-silyl benzylboronic ester 11

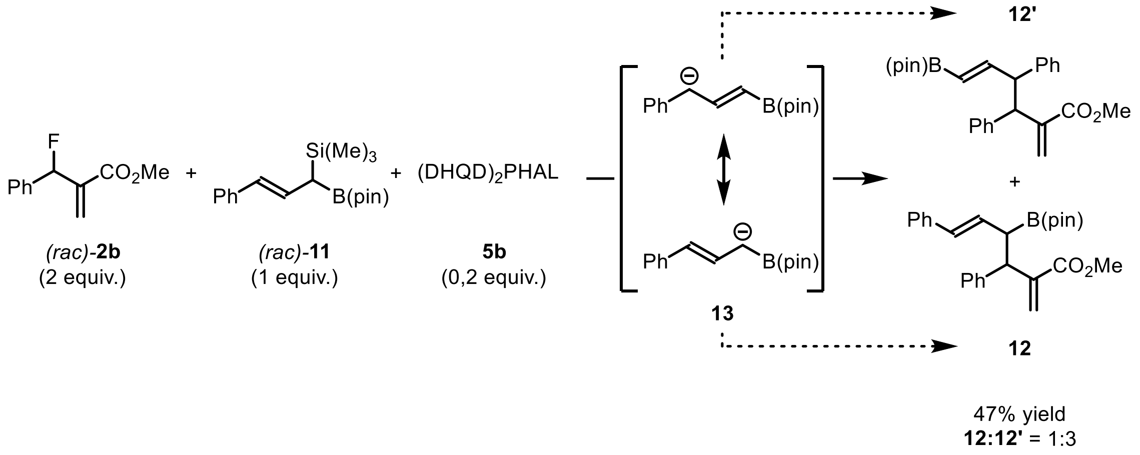

*[Subjecting the α-silyl allyl boronic ester **11** to standard reaction conditions produced a mixture of α- and γ-alkylated products **12** and **12'**. This observation is consistent with the formation of the carbanion **13** upon fluorine-assisted desilylation, which can react on both mesomeric forms.]*

Characterization of 12 and 12'

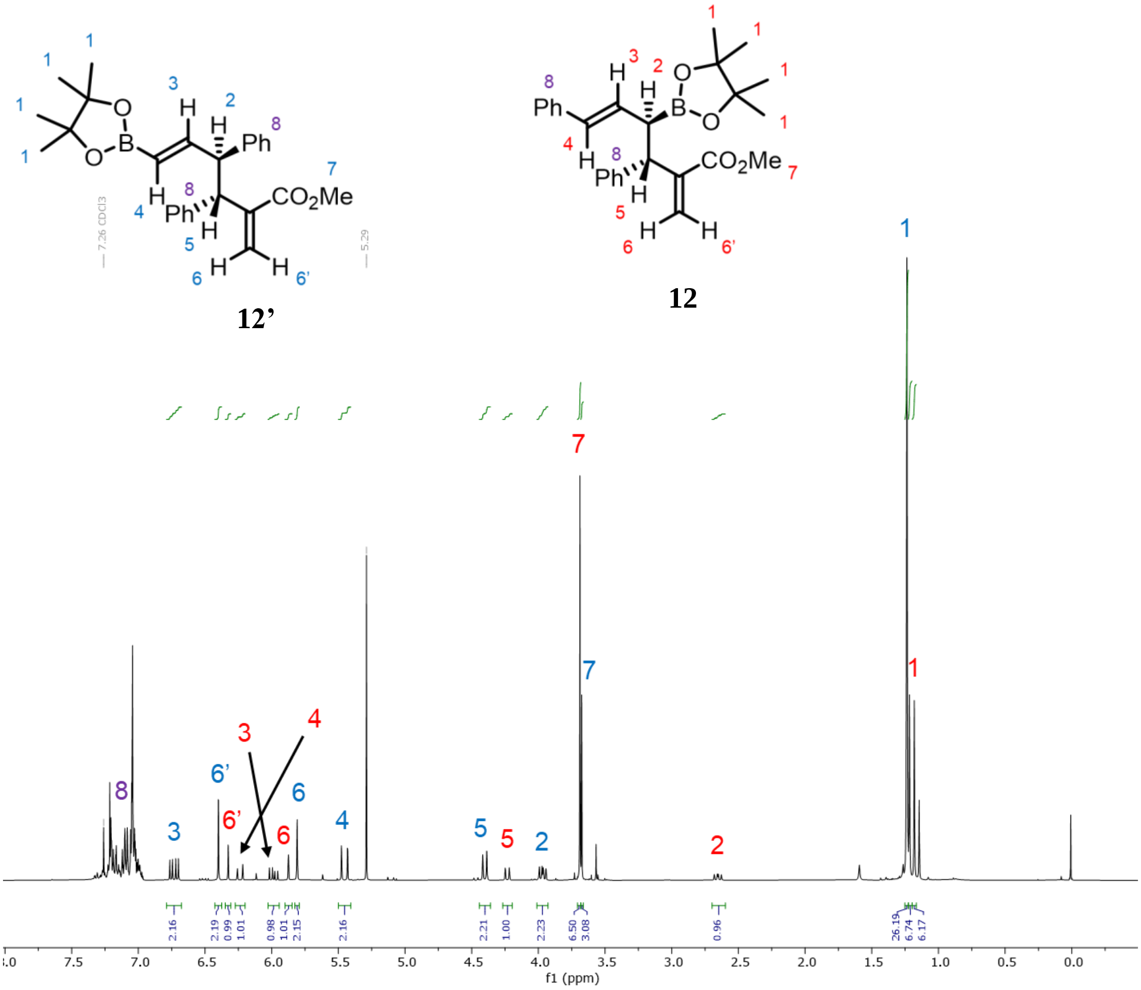

Figure S12. ^{1}H NMR spectrum of **12** and **12'** at 400 MHz in $CDCl_3$.

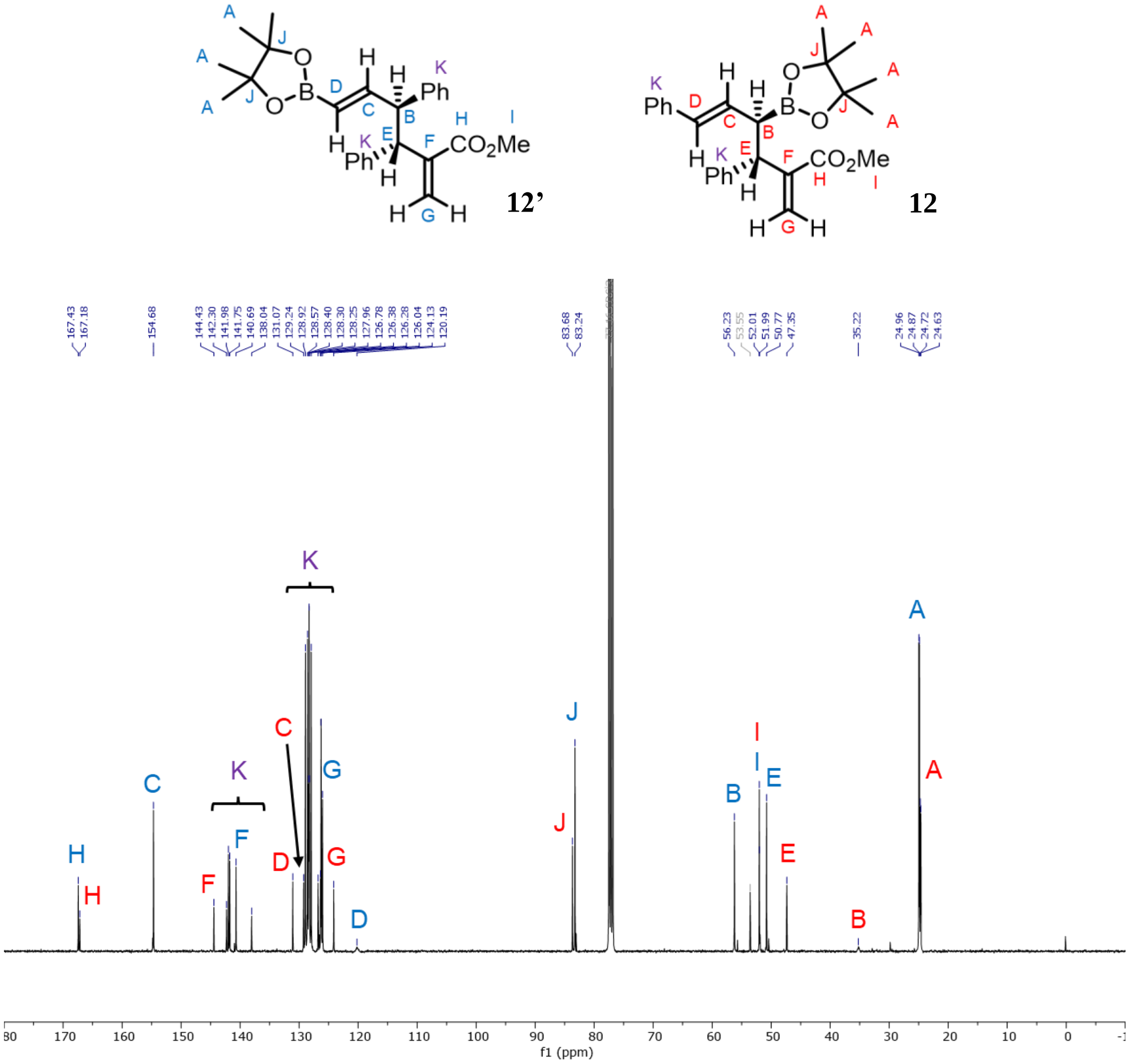

Figure S13. ^{13}C NMR spectrum of **12** and **12'** at 400 MHz in $CDCl_3$.

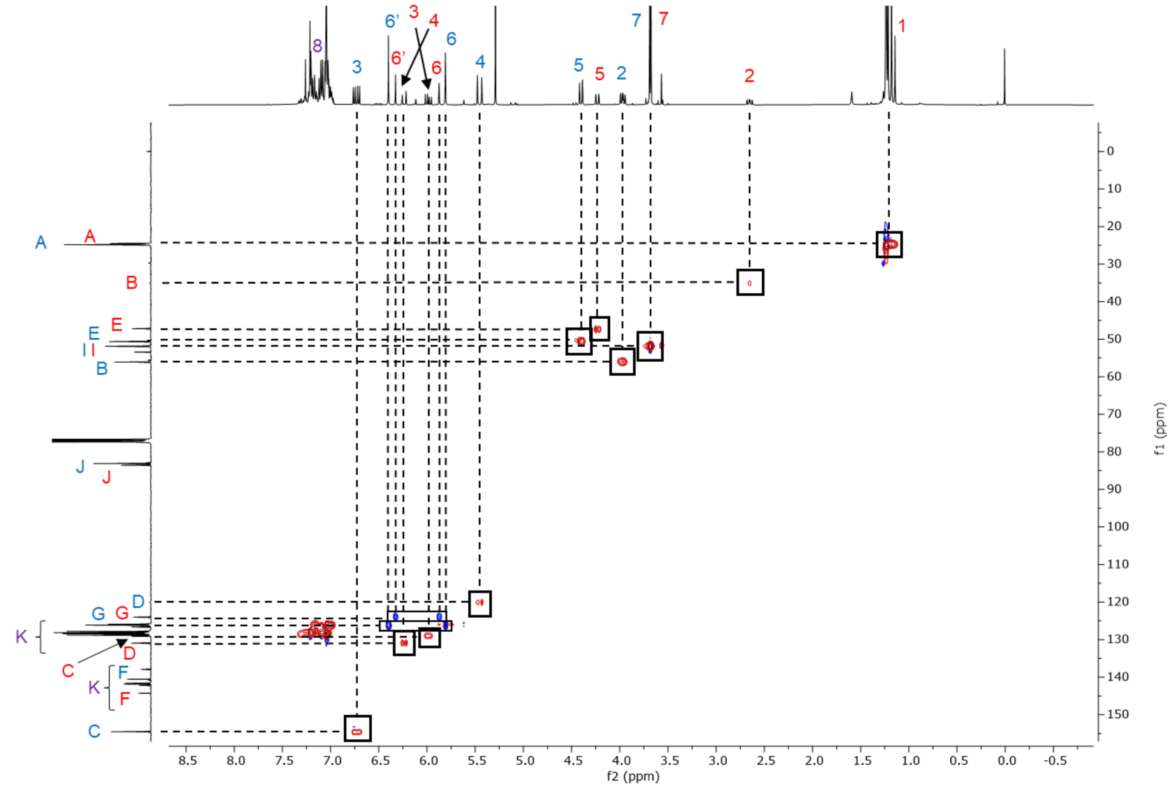

Figure S14. HSQC spectrum of **12** and **12’** at 400 MHz in $CDCl_3$.

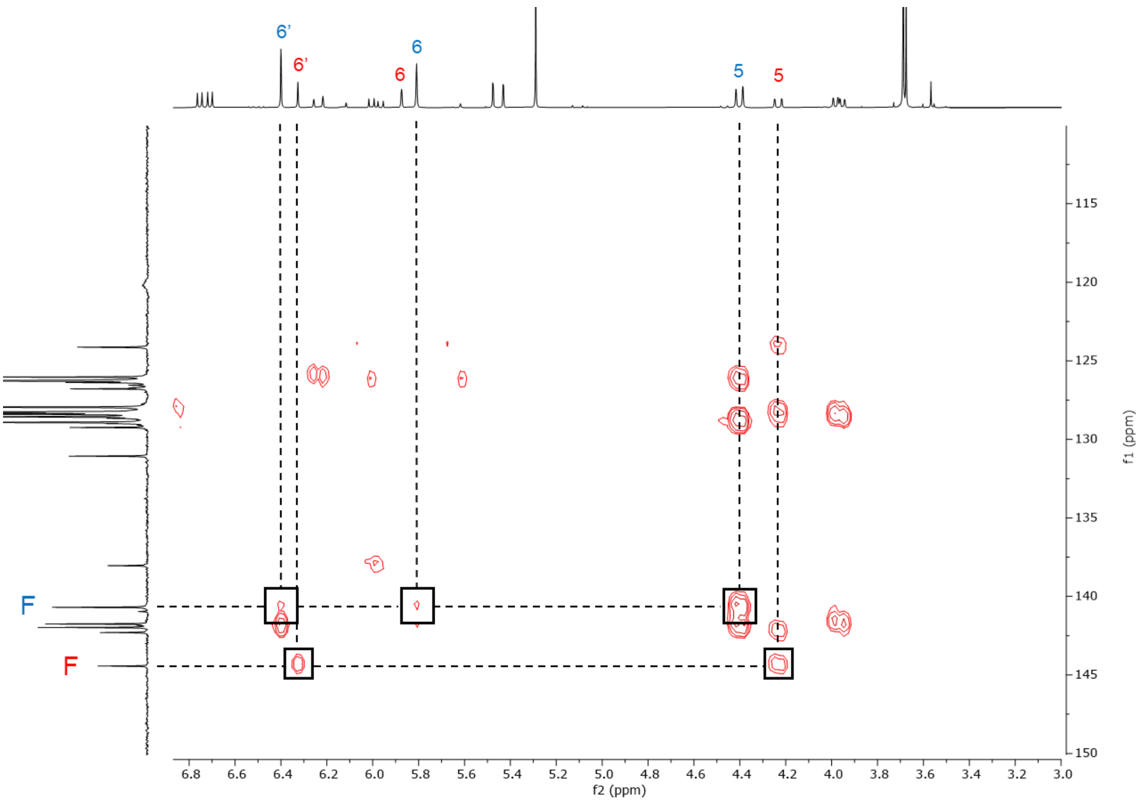

Figure S15. HMBC spectrum of **12** and **12’** at 400 MHz in $CDCl_3$.

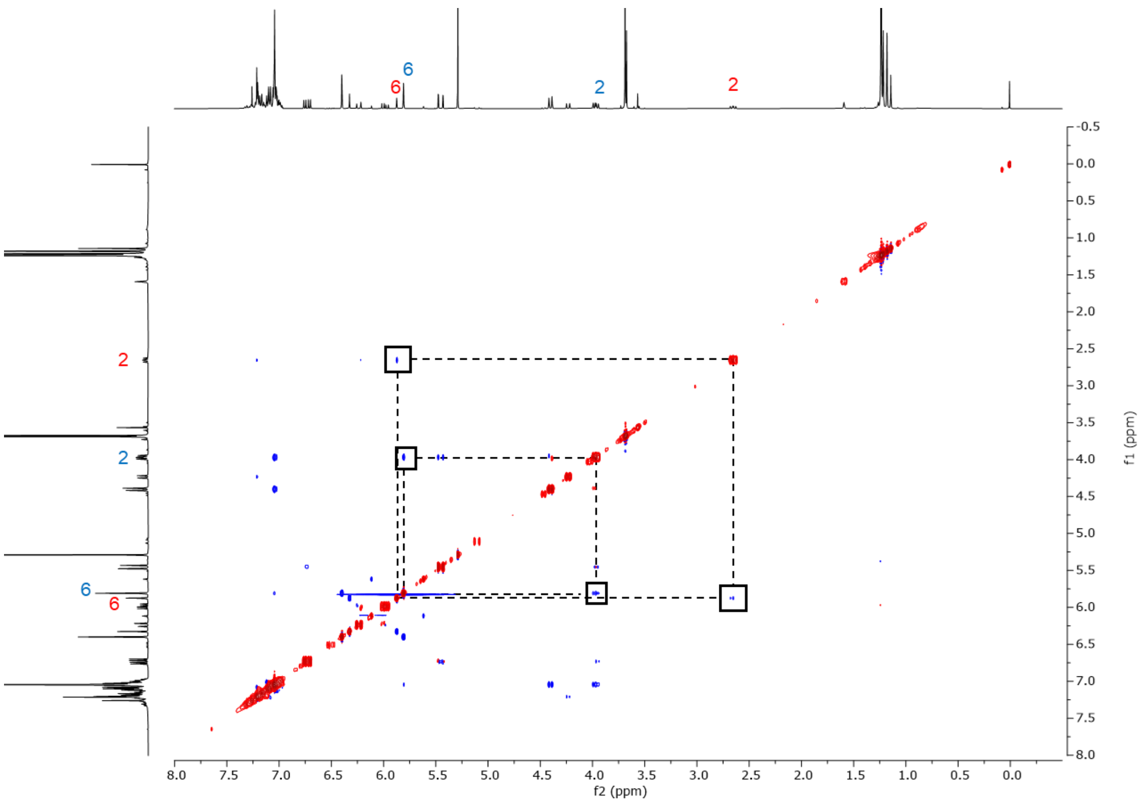

Figure S16. 2D NOESY spectrum of **12** and **12’** at 400 MHz in $CDCl_3$.

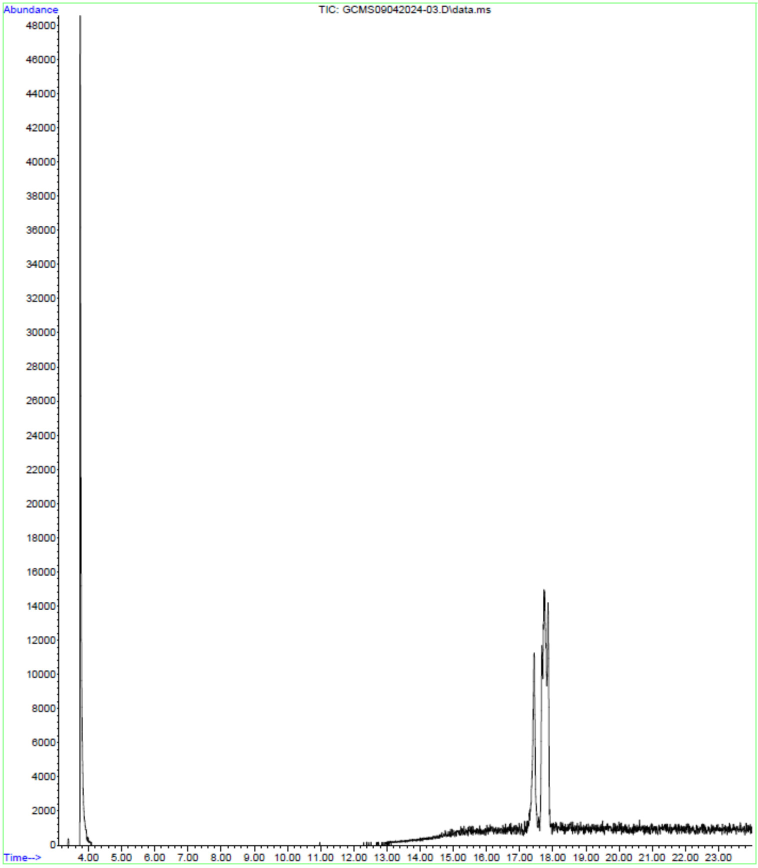

Figure S17. GC-MS chromatogram of **12** and **12’**

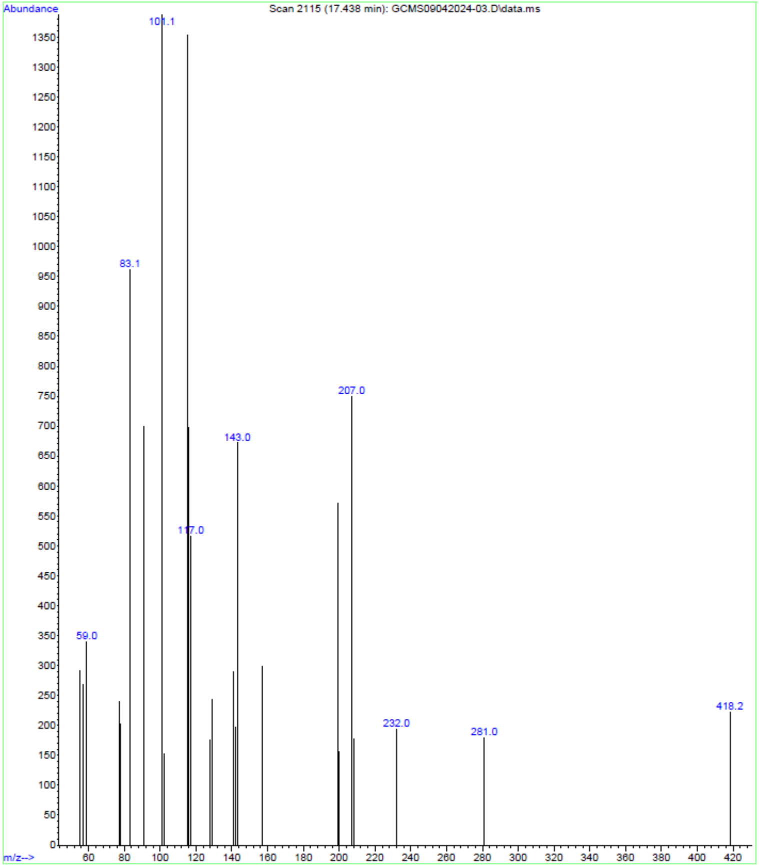

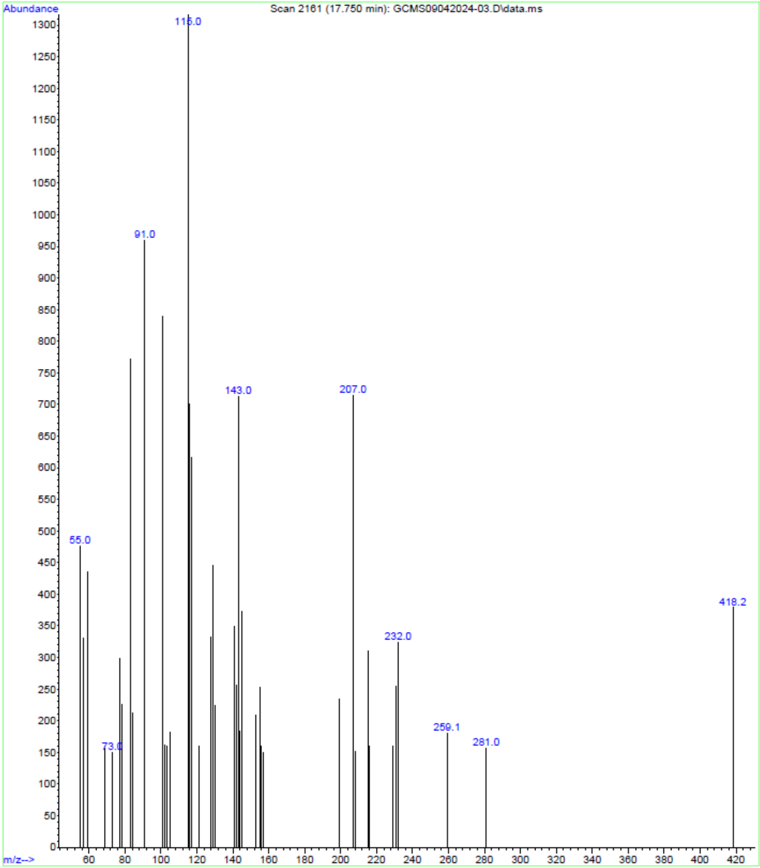

Figure S18. Mass spectra of **12** and **12'**. Both regioisomers have the same mass, but different fragmentation patterns.

G.4. Kinetic profiles – Kinetic resolution of 2b

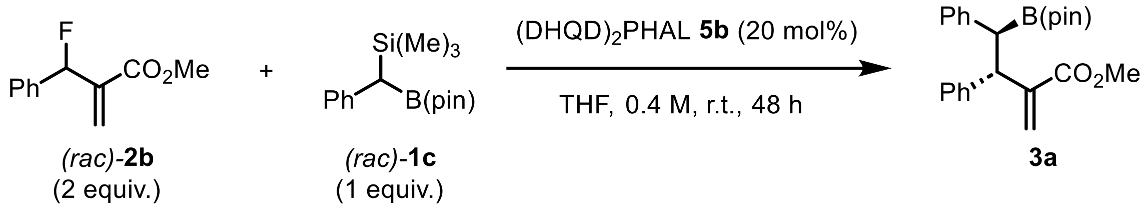

Starting Materials Evolution

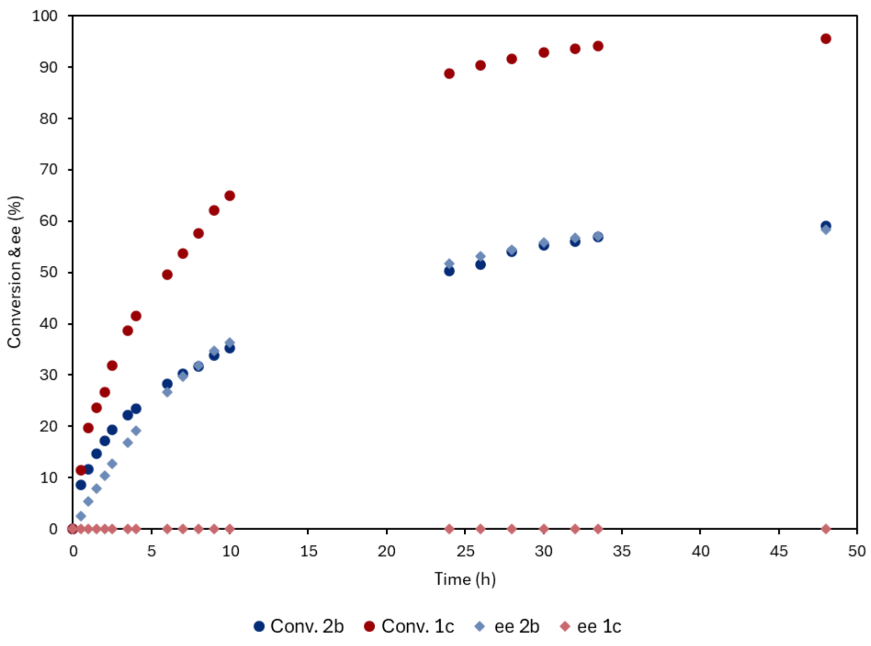

Figure S19. Kinetic and stereochemical profiles (conv. and ee *vs*. time) under optimised catalytic conditions analyzed by conventional and chiral GC.

[The α-silyl benzylboronic ester ***1c*** *remains racemic during the whole reaction course, while allyl fluoride* ***2b*** *undergoes kinetic resolution, resulting in its enantioenrichement in (R) form.]*

G.5. Kinetic profiles – Diastereochemical profile

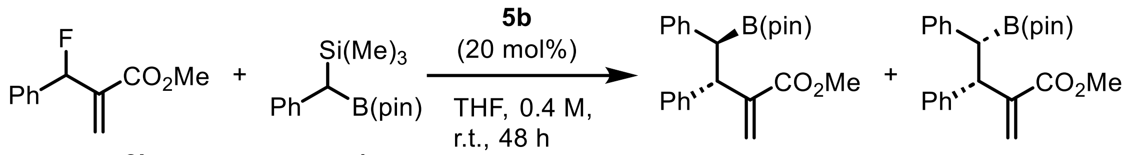

(*rac*)-**2b**
(2 equiv.)

(*rac*)-**1c**
(1 equiv.)

3a

3a'

Product Formation

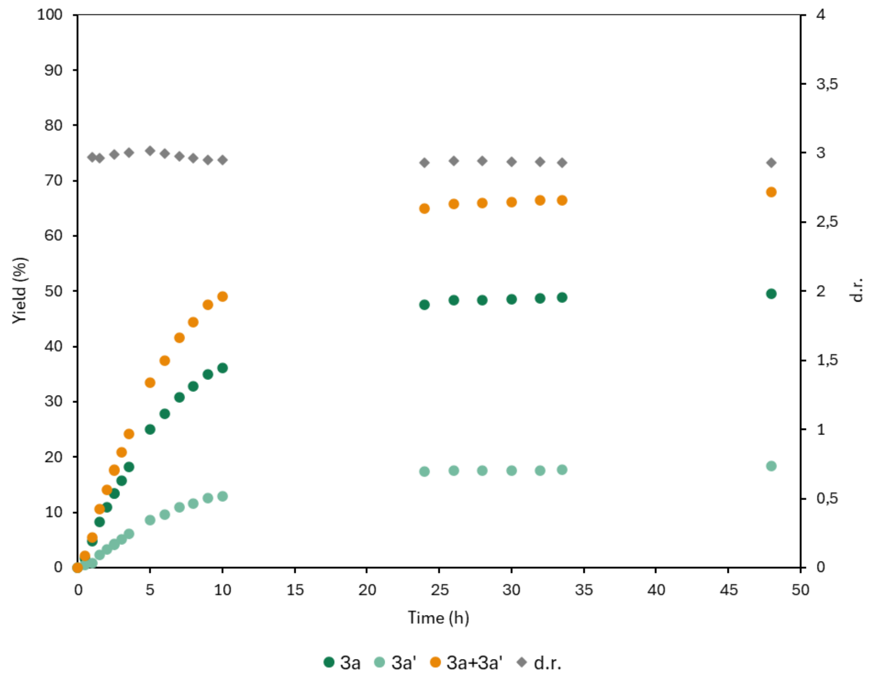

Figure S20. Kinetic and diastereomeric profiles (yields –left axis– and d.r. –right axis– of products **3a** and **3a'** *vs.* time) analysed by GC.

[The d.r. of the product remains constant during the whole reaction course.]

G.6. Control experiment *– Study of the potential erosion of the diastereoselectivity promoted by the catalyst.*

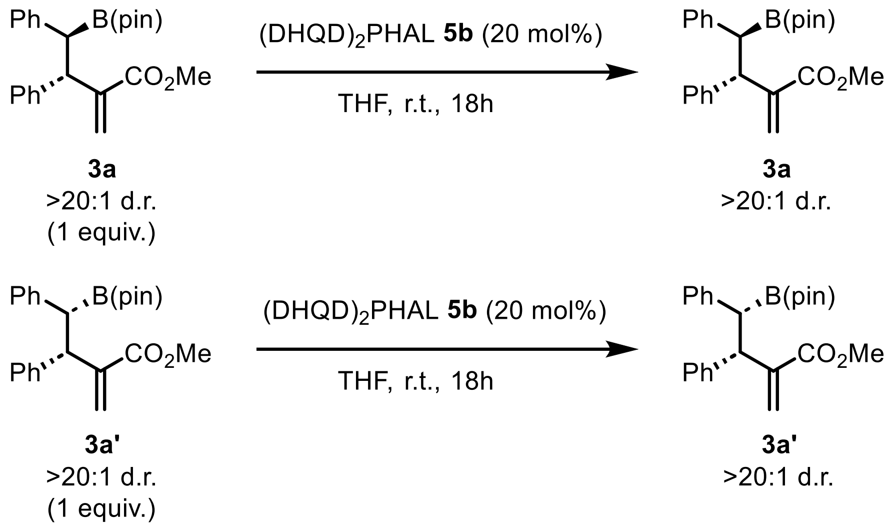

Diastereopure products **3a** and **3a’** (1 equiv., 0.1 mmol, >20:1 d.r.) were weighted independently into a 5 mL vial equipped with a magnetic stirring bar and dissolved with 0.25 mL of THF (0.4 M). Subsequently, 20 mol% of catalyst was added in each vial. Both mixtures were stirred for 18 h at room temperature. Then, THF was dried under vacuum, each crude mixture was diluted in $CDCl_3$ and transferred into two NMR tubes for ^{1}H NMR analysis.

[After 18 h, neither of the two diastereoisomers showed epimerisation. Therefore, the diastereoselectivity is not eroded by the effect of the catalyst ***5b****]*

H. Derivatisations

H.1. Oxidation and cyclisation – Lactonisation

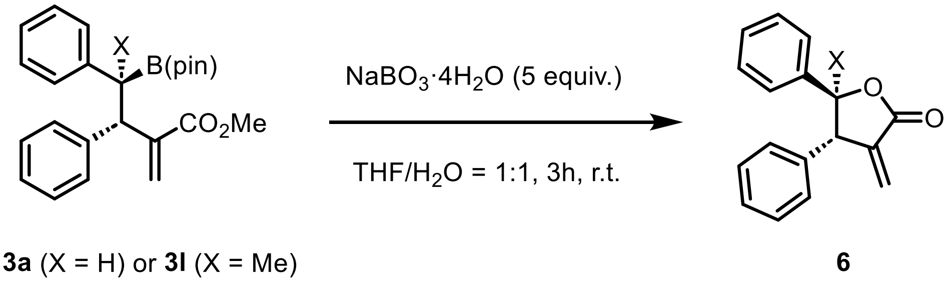

The reaction was carried out adapting the previously described procedure.[8a]

$NaBO_3 \cdot 4H_2O$ (5 equiv.) was added to a solution of the corresponding chiral homoallylic boronic ester **3a** or **3l** (1.0 equiv.) in THF/H_2O (1:1) at room temperature. The mixture was stirred vigorously for 3 h, and subsequently H_2O was added. The resulting solution was extracted three times with ethyl acetate. The combined organic layers were dried over anhydrous $MgSO_4$, filtered, and concentrated under vacuum. Purification by flash column chromatography on silica gel using *n*-hexane/ethyl acetate = 90:10 as eluent afforded the corresponding α-methylene-γ-butyrolactone **6a** or **6b**.

(4*S*,5*S*)-3-methylene-4,5-diphenyldihydrofuran-2(3*H*)-one (6a)

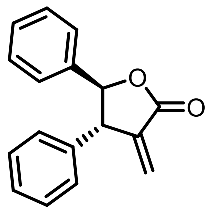

6a** was prepared following the abovementioned procedure employing the homoallylic boronic ester **3a

81% yield, >20:1 d.r. (20 mg, 0.08 mmol), white solid. Spectroscopic data match with a different method reported previously that synthesise the same product in racemic form.[8b]

^{1}H NMR (400 MHz, $CDCl_3$) δ 7.42 – 7.33 (m, 6H), 7.24 – 7.14 (m, 4H), 6.45 (d, *J* = 3.3 Hz, 1H), 5.46 (d, *J* = 3.0 Hz, 1H), 5.38 (d, *J* = 7.7 Hz, 1H), 4.05 (dt, *J* = 7.8, 3.1 Hz, 1H) ppm. **$[\alpha]_D^{25}$** = +8.7 (c = 0.11, $CHCl_3$).

(4*S*,5*S*)-5-methyl-3-methylene-4,5-diphenyldihydrofuran-2(3*H*)-one (6b)

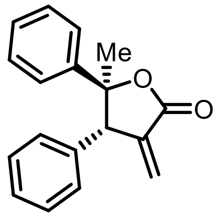

***6b** was prepared following the abovementioned procedure employing the homoallylic boronic ester **3l**.*

40% yield, >20:1 d.r. (7 mg, 0.03 mmol), colourless oil. **^{1}H NMR** (400 MHz, $CDCl_3$) δ 7.61 (t, *J* = 2.9 Hz, 1H), 7.50 – 7.36 (m, 9H), 7.31 (dd, *J* = 7.8, 1.0 Hz, 1H), 3.43 (d, *J* = 2.8 Hz, 2H), 1.79 (s, 3H) ppm. **^{13}C NMR** (101 MHz, $CDCl_3$) δ 171.5, 145.1, 137.3, 134.8, 130.1, 130.0, 129.1, 128.8, 127.8, 125.2, 124.3, 84.2, 43.1, 30.6 ppm. **HRMS (ESI)** Calculated for $[C_{18}H_{17}O_2]^+$ ($[M+H]^+$) 265.1223. Found 265.1223. **$[\alpha]_D^{25}$** = +28.6 (c = 0.08, $CHCl_3$).

***H.2. Heteroaromatic substitution** – Stereoospecific sp^2–sp^3 coupling of **3a** with electronrich aromatics.*

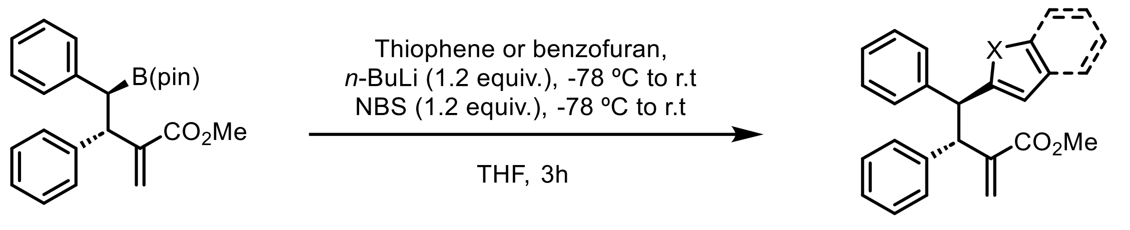

3a (1 equiv.) **7a** (X = S) or **7b** (X = O)

The reaction was carried out by adapting a procedure previously described.[9]

A solution of thiophene or benzofuran (1.2 equiv., 0,12 mmol) in THF (0.3M) was cooled to –78 °C and treated with *n*-BuLi (1.2 equiv., 1.6M in hexanes). The cooling bath was removed, and the mixture was stirred at room temperature for 1 h. The mixture was cooled again to –78 °C, **3a** (1.0 equiv., 0,1 mmol) was added dropwise as a solution in THF (0.5M) and stirred at –78 °C for 1 h. Subsequently, a solution of NBS (1.2 eq., 0,12 mmol) in THF (0.3M) was added dropwise at the same temperature. After 1h at –78 °C, a saturated aqueous solution of $Na_2S_2O_3$ was added and the reaction mixture was allowed to warm up to room temperature. The reaction mixture was diluted with Et_2O and water. The aqueous layer was extracted with Et_2O. The combined organic layers were dried with anhydrous $MgSO_4$, filtered and concentrated under vacuum. The crude material was purified by flash column chromatography on silica gel eluting with *n*-hexane/ethyl acetate = 90:10.

methyl (3*S*,4*S*)-2-methylene-3,4-diphenyl-4-(thiophen-2-yl)butanoate (7a)

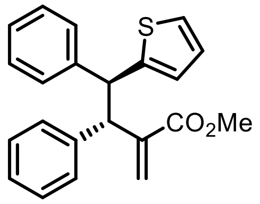

***7a** was prepared following the abovementioned procedure.*[9]

74% yield, >20:1 d.r. (26 mg, 0.074 mmol), reddish solid. **^{1}H NMR** (500 MHz, $CDCl_3$) δ 7.13 – 7.06 (m, 9H), 7.02 (dddd, *J* = 9.2, 6.8, 4.9, 3.1 Hz, 2H), 6.95 (dt, *J* = 3.4, 1.0 Hz, 1H), 6.89 (dd, *J* = 5.1, 3.5 Hz, 1H), 6.28 (s, 1H), 5.83 (d, *J* = 0.8 Hz, 1H), 4.85 (d, *J* = 12.2 Hz, 1H), 4.74 (dd, *J* = 12.2, 0.9 Hz, 1H), 3.65 (s, 3H) ppm. **^{13}C NMR** (101 MHz, $CDCl_3$) δ 167.1, 147.2, 142.5, 141.9, 140.5, 128.7, 128.2, 128.1, 128.0, 126.6, 126.4, 126.3, 125.5, 124.5, 123.7, 52.7, 51.9, 50.6 ppm. **HRMS (ESI)** Calculated for $[C_{22}H_{21}O_2S]^+$ ($[M+H]^+$) 349.1257. Found 349.1253. **mp** = 138–140; **[α]$_D$^{25}** = +197.9 (c = 1.39, $CHCl_3$).

methyl (3*S*,4*S*)-4-(benzofuran-2-yl)-2-methylene-3,4-diphenylbutanoate (7b)

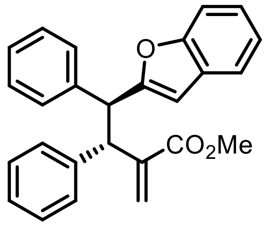

***7b** was prepared following the abovementioned procedure.*[9]

48% yield, >20:1 d.r. (18 mg, 0.048 mmol), yellow solid. **^{1}H NMR** (500 MHz, $CDCl_3$) δ 7.51 – 7.45 (m, 1H), 7.42 – 7.38 (m, 1H), 7.22 – 7.13 (m, 4H), 7.13 – 7.01 (m, 8H), 6.57 (t, *J* = 0.7 Hz, 1H), 6.24 (s, 1H), 5.88 (t, *J* = 0.7 Hz, 1H), 4.83 (dd, *J* = 12.4, 0.9 Hz, 1H), 4.75 (d, *J* = 12.3 Hz, 1H), 3.64 (s, 3H) ppm. **^{13}C NMR** (101 MHz, $CDCl_3$) δ 167.1, 159.3, 154.7, 141.8, 140.0, 140.0, 128.9, 128.6, 128.3, 128.2, 126.8, 126.6, 125.3, 123.6, 122.7, 120.7, 111.2, 103.4, 52.1, 50.6, 49.7 ppm. **HRMS (ESI)** Calculated for $[C_{26}H_{23}O_3]^+$ ($[M+H]^+$) 383.1642. Found 383.1645. **mp** = 117–119; **[α]$_D$^{25}** = +216.0 (c = 1.15, $CHCl_3$).

H.3. Hydrogenation and lactonisation – Alkene reduction, oxidation and cyclisation

Alkene reduction

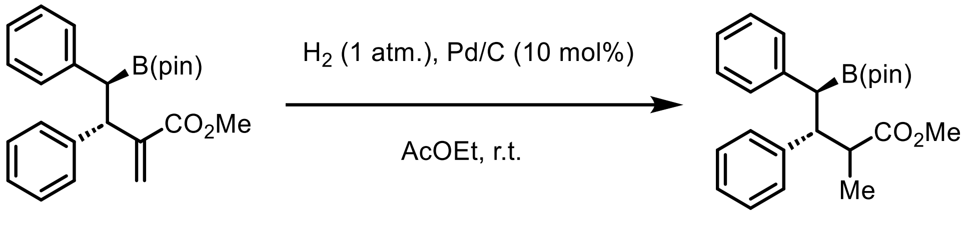

3a (1 equiv.) **8**

The reaction was carried out adapting the previously described procedure.[2d]

To a solution of compound **3a** (0.1 mmol) in ethyl acetate (0.1 M) was added 10 wt.% Pd/C (0.1 equiv.). The mixture was reacted overnight under hydrogen atmosphere at room temperature. Then, the reaction mixture was filtered through Celite, and the filtrate was concentrated under vacuum. Purification of the residue by flash column chromatography on silica gel using *n*-hexane/dichloromethane mixtures allowed to separate the hydrogenated products **8** and **8'** (98% yield, d.r. =3,5:1).

methyl 2-methyl-3,4-diphenyl-4-(4,4,5,5-tetramethyl-1,3,2-dioxaborolan-2-yl)butanoate

98% yield (39 mg, 0,1 mmol) of a 3,5:1 diastereomeric ratio. Pure samples of each diastereomer were obtained upon chromatographic purification using *n*-hexane/dichloromethane = 1:1.

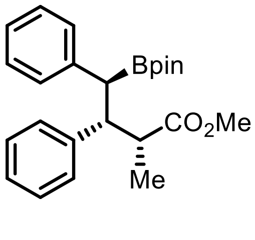

Major diastereomer (2*R*,3*R*,4*S*)-8, colourless oil. **^{1}H NMR** (400 MHz, $CDCl_3$) δ 7.11 – 6.88 (m, 10H), 3.59 (s, 3H), 3.44 (dd, *J* = 11.5, 6.5 Hz, 1H), 3.08 (p, *J* = 6.9 Hz, 1H), 2.92 (d, *J* = 11.5 Hz, 1H), 1.21 (s, 6H), 1.17 (s, 6H), 1,16 (d, *J* = 6.9 Hz, 3H) ppm; **^{13}C NMR** (101 MHz, $CDCl_3$) δ 175.5, 140.9, 140.6, 129.5, 129.1, 127.9, 127.6, 126.2, 125.2, 83.6, 51.3, 51.2, 44.5, 36.9, 24.7, 24.6, 16.3 ppm; **^{11}B NMR** (128 MHz, $CDCl_3$) δ 32.59 ppm; **HRMS (ESI)** Calculated for $[C_{24}H_{32}BO_4]^+$ ($[M+H]^+$) 395.2388. Found 395.2384; **$[\alpha]_D^{25}$** = +29,5 (c = 1.76, $CHCl_3$).

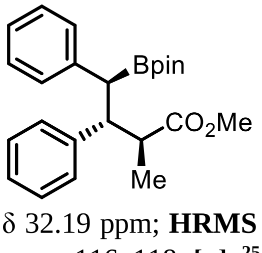

Minor diastereomer (2*S*,3*R*,4*S*)-8', white solid. **^{1}H NMR** (400 MHz, $CDCl_3$) δ 7.14 – 6.91 (m, 10H), 3.87 (dd, *J* = 10.8, 6.0 Hz, 1H), 3.64 (s, 3H), 3.04 (qd, *J* = 7.1, 6.0 Hz, 1H), 2.93 (d, *J* = 10.8 Hz, 1H), 1.20 (s, 6H), 1.18 (s, 6H), 1.07 (d, *J* = 7.1 Hz, 3H) ppm; **^{13}C NMR** (101 MHz, $CDCl_3$) δ 175.8, 140.0, 139.8, 129.8, 129.4, 127.9, 127.6, 126.4, 125.4, 83.8, 51.6, 49.0, 44.1, 36.0, 24.7, 12.4 ppm; **^{11}B NMR** (128 MHz, $CDCl_3$) δ 32.19 ppm; **HRMS (ESI)** Calculated for $[C_{24}H_{32}BO_4]^+$ ($[M+H]^+$) 395.2388. Found 395.2385; **mp** = 116–118; **$[\alpha]_D^{25}$** = +19.5 (c = 0.45, $CHCl_3$).

Oxidation and cyclisation of hydrogenated boronic ester 8

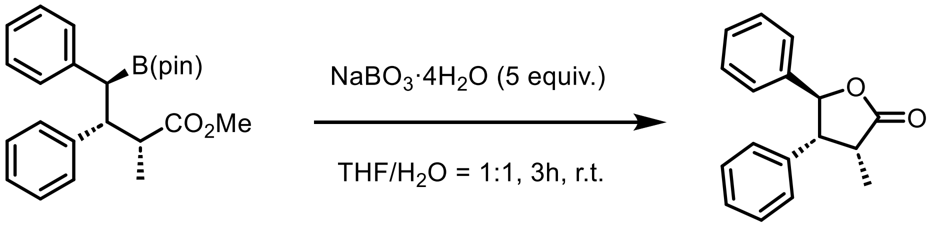

8 **9**

***9** was prepared following the procedure described in section H1 employing the hydrogenated boronic ester **8**.*

(3*R*,4*R*,5*S*)-3-methyl-4,5-diphenyldihydrofuran-2(3*H*)-one (9)

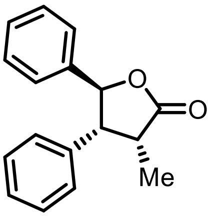

87% yield, >20:1 d.r. (14 mg, 0.03 mmol), white solid. **^{1}H NMR** (400 MHz, $CDCl_3$) δ 7.42 – 7.29 (m, 8H), 7.25 – 7.21 (m, 2H), 5.77 (d, J = 3.8 Hz, 1H), 3.68 (dd, J = 8.6, 3.8 Hz, 1H), 3.06 (dq, J = 8.7, 7.4 Hz, 1H), 0.92 (d, J = 7.4 Hz, 3H) ppm. **^{13}C NMR** (101 MHz, $CDCl_3$) δ 179.2, 139.2, 138.1, 129.2, 128.9, 128.4, 128.1, 127.9, 125.1, 83.9, 53.8, 38.2, 11.3 ppm. **HRMS (ESI)** Calculated for $[C_{17}H_{17}O_2]^+$ ($[M+H]^+$) 253.1223. Found 253.1226. **mp** = 63–65; **$[\alpha]_D^{25}$** = +66.9 (c = 0.93, $CHCl_3$).

Determination of the relative configuration of 9 by 2D NOESY

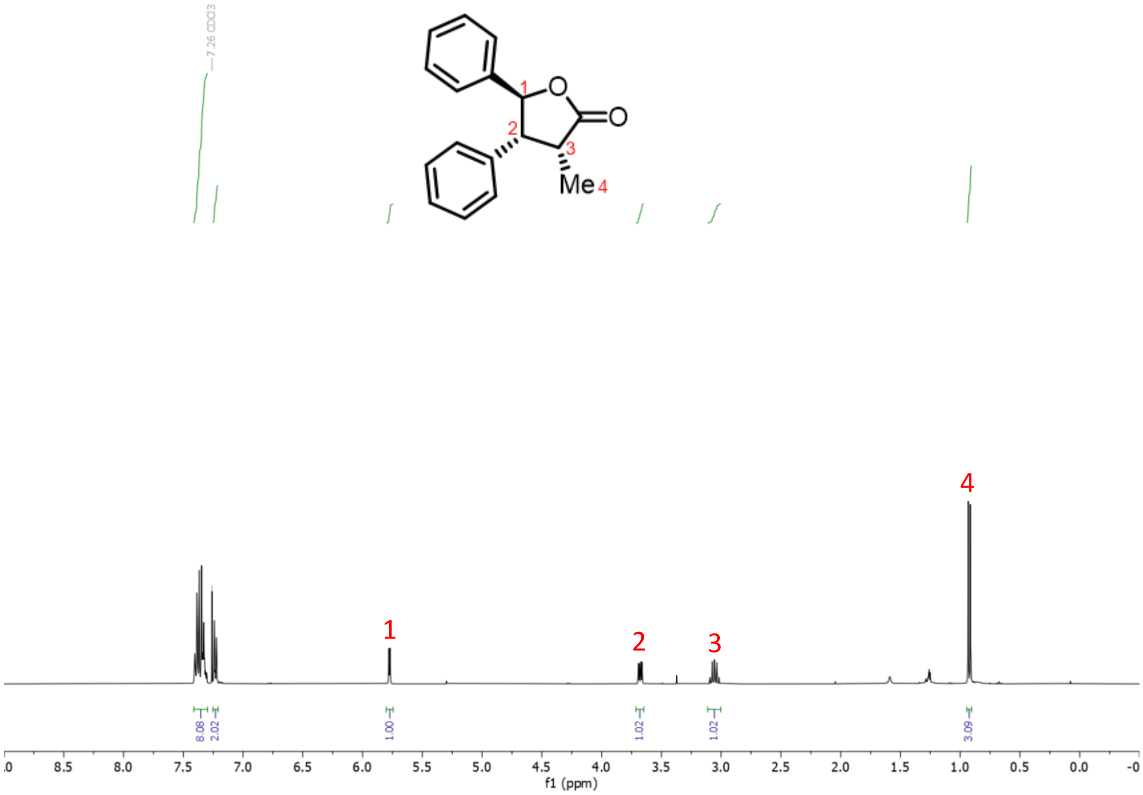

Figure S21. ^{1}H NMR spectrum of **9** at 400 MHz in $CDCl_3$.

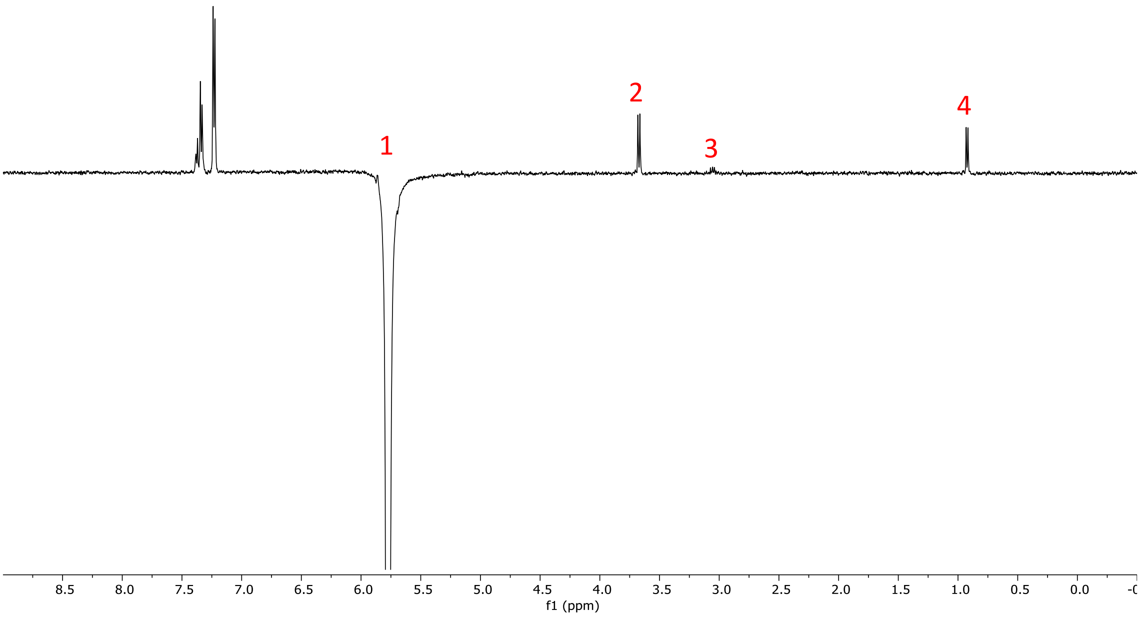

Figure S22. 1D NOESY spectra of **9** at 400 MHz in $CDCl_3$.

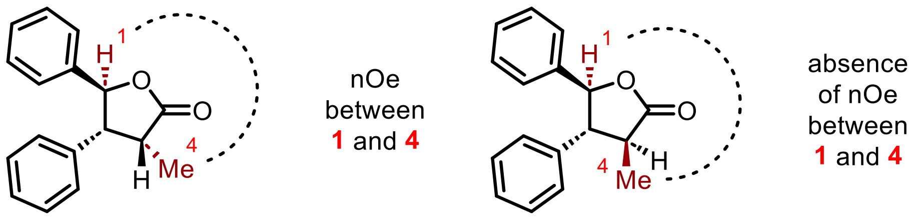

*[The nOe contact between **1** and **4** indicate that they are in cis in the lactone ring, confirming the relative configuration of C3 in **9**.]*

αH.4. Protodeborylation

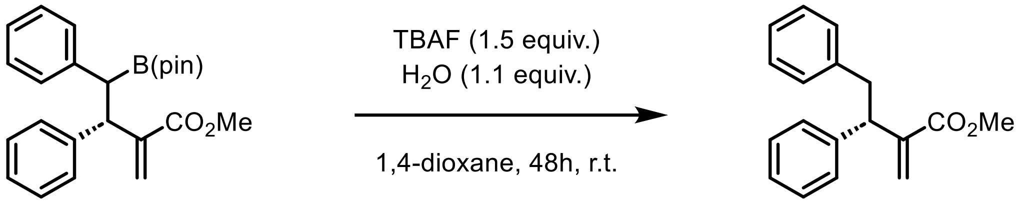

3a or **3a'** **10**

The reaction was carried out by adapting a procedure previously described.[10]

Homoallylic boronic ester **3a** or **3a'** (1 equiv., 0,05 mmol) was weighted into a 5 mL vial equipped with a magnetic stirring bar and dissolved with 1,4-dioxane (0.1 M). Subsequently, H_2O (1,1 equiv., 0,055 mmol) and tetrabutylammonium fluoride 1.0 M in THF (1,5 equiv., 0,075 mmol) were added. The reaction mixture was stirred at room temperature during 48 h. The crude product **10** was directly purified by flash column chromatography on silica gel using *n*-hexane/ethyl acetate = 90:10.

methyl (*S*)-2-methylene-3,4-diphenylbutanoate (10)

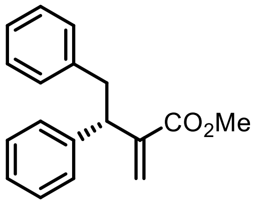

***10** was prepared following the abovementioned procedure.*[10]

^{1}H NMR (500 MHz, $CDCl_3$) δ 7.26 – 7.11 (m, 8H), 7.07 – 7.01 (m, 2H), 6.33 (t, *J* = 0.7 Hz, 1H), 5.72 (t, *J* = 1.1 Hz, 1H), 4.22 (ddd, *J* = 8.5, 6.9, 1.0 Hz, 1H), 3.65 (s, 3H), 3.21 (dd, *J* = 13.7, 6.9 Hz, 1H), 3.06 (dd, *J* = 13.7, 8.7 Hz, 1H) ppm; **^{13}C NMR** (101 MHz, $CDCl_3$) δ 167.4, 143.3, 142.0, 139.9, 129.1, 128.4, 128.3, 128.2, 126.6, 126.1, 124.9, 52.0, 48.1, 41.0 ppm; **HRMS (ESI)** Calculated for $[C_{18}H_{19}O_2]^+$ ($[M+H]^+$) 267.1380. Found 267.1374; **$[\alpha]_D^{25}$** = +106.0 (c = 1.18, $CHCl_3$); **HPLC** Phenomenex Lux Cellulose-1 (95:5 *n*-Hexane: 2-Propanol, 1 mL/min, 232 nm); t_R (major) = 6,1, t_R (minor) = 7,2. 88% yield, 92:8 e.r. from **3a** and 92% yield, 93:7 e.r. from **3a'** as a colourless oil.

I. References

[1] (a) X. Companyó, P. Y. Geant, A. Mazzanti, A. Moyano, R. Rios, *Tetrahedron* **2014**, *70*, 75-83. (b) M. S. Santos, F. Coelho, *RSC Adv*. **2012**, *2*, 3237-3241. (c) M. A. Helppi, A. L. Lehmkuhler, J. M. Marchi, C. M. Schmidt, M. T. Yip-Schneider, P. V. Ramachandran, *Bioorg. Med. Chem. Lett*. **2015**, *25*, 4270-4273. (d) C. Rasson, A. Stouse, A. Boreux, V. Cirriez, O. Riant, *Chem. Eur. J.* **2018**, *24*, 9234-9237. (e) N. S. Akins, N. Mishra, H. M. Harris, N. Dudhipala, S. J. Kim, A. W. Keasling, S. Majumdar, J. K. Zjawiony, J. J. Paris, N. M. Ashpole, H. V. Le, *Chem. Med. Chem*. **2022**, *17,* e202100684.

[2] (a) T. Nishimine, K. Fukushi, N. Shibata, H. Taira, E. Tokunaga, A. Yamano, M. Shiro, N. Shibata, *Angew. Chem. Int. Ed.* **2014**, *53*, 517-520 (b) T. Nishimine, H. Taira, E. Tokunaga, M. Shiro, N. Shibata, *Angew. Chem. Int. Ed.* **2016**, *55,* 359-363 (c) Y. Zi, M. Lange, C. Schultz, I. Vilotijevic, *Angew. Chem. Int. Ed.* **2019**, *58*, 10727-10731. (d) J. Duran, J. Mateos, A. Moyano, X. Companyó, *Chem. Sci.* **2023**, *14*, 7147-7153.

[3] (a) C. Bomio, M. A. Kabeshov, A. R. Lit, S. H. Lau, J. Ehlert, C. Battilocchio, S. V. Ley, *Chem. Sci*. **2017**, *8*, 6071-6075. (b) D. M. Lustosa, S. Barkai, I. Domb, A. Milo, *J. Org. Chem.* **2022**, *87*, 1850−1857. (c) C. Wu, Z. Bao, X. Xua, J. Wang, *Org. Biomol. Chem.* **2019**, *17*, 5714-5724. (d) E. Kohei, K. Fumiya, U. Yutaka, *Chem. Lett*. **2013**, *42*, 1363–1365.

[4] (a) J. Huang, W. Yan, C. Tan, W. Wu, H. Jiang, *Chem. Commun.* **2018**, *54*, 1770-1773. (b) H. Jin, J. Han, X. Liu, C. Feng, M. Zhan, *Org. Lett.* **2023**, *25,* 4168-4172.

[5] (a) C. Cassani, R. Martín-Rapún, E. Arceo, F. Bravo, P. Melchiorre, *Nat. Protoc.* **2013**, *8*, 325-344. (b) S. Bai, S. Liu, Y. Zhu, K. Zhao, Q. Wu, *Synlett* **2018**, *29*, 1921-1925. (c) H. Wu, Handoko, M. Raj, P. S. Arora, *Org. Lett.* **2017**, *19*, 5122-5125. (d) Y. Wang, Z. Wei, J. Cao, D. Liang, Y. Lin, H. Duan, *New J. Chem.* **2021**, *45*, 2609-2613 (e) D. Svestka, J. Otevrel, P. Bobal, *Adv. Syn. Catal.* **2022**, *364*, 2174-2183.

[6] (a) B. Lee, P. J. Chirik, *J. Am. Chem. Soc.* **2020**, *142*, 2429-2437. (b) R. K. Sharma, J. L. Fry, *J. Org. Chem*. **1983**, *48*, 2112-2114. (c) D. P. Cox, J. Terpinski, W. Lawrynowicz, *J. Org. Chem*. **1984**, *49*, 3216-3219. (d) R. I. Hogrefe, A. P. McCaffrey, L. U. Borozdina, E. S. McCampbell, M. M. Vaghefi, *Nucleic Acids Res.* **1993**, *21*, 4739-4741. (e) H. Sun, S. G. DiMagno, *J. Am. Chem. Soc*. **2005**, *127*, 2050–2051.

[7] (a) T. W. Butcher, J. L. Yang, W. M. Amberg, N. B. Watkins, N. D. Wilkinson, J. F. Hartwig, *Nature* **2020**, *583*, 548-553. (b) M. Bergeron, D. Guyader, J. F. Paquin, *Org. Lett.* **2012**, *14*, 5888-5891.

[8] (a) M. M. Hussain, H. Li, N. Hussain, M. Urena, P. J. Carroll, P. J. Walsh, *J. Am. Chem. Soc.* **2009**, *131*, 6516-6524. (b) X. Lei, Y. Li, Y. Lai, S. Hu, C. Qi, G. Wang, Y. Tang, *Angew. Chem. Int. Ed.* **2021**, *60*, 4221-4230.

[9] A. Bonet, M. Odachowski, D. Leonori, S. Essafi, V. K. Aggarwal, *Nat. Chem.* **2014**, *6*, 584-589.

[10] S. Nave, R. P. Sonawane, T. G. Elford, V. K. Aggarwal, *J. Am. Chem. Soc.* **2010**, *132*, 17096-17098.

J. Determination of the absolute configuration. X-ray crystallographic data of compound 3c

Crystal obtention

The crystal was obtained by slow evaporation from a *n*-pentane/CH_2Cl_2 = 5:1 solution.

A colourless needle-like specimen with approximate dimensions 0.253 mm x 0.066 mm x 0.029 mm, was used for the X-ray crystallographic analysis.

The X-ray intensity data were measured at 100K on a D8 Venture system equipped with a multilayer monochromator and a Mo microfocus (λ _= 0.71073 Å). The frames were integrated with the Bruker SAINT software package using a narrow-frame algorithm. The integration of the data using a monoclinic unit cell yielded a total of 68645 reflections to a maximum θ _angle of 30.58° (0.70 Å resolution), of which 14087 were independent (average redundancy 4.873, completeness = 99.9%, Rint = 14.61%, Rsig = 13.92%) and 8324 (59.09%) were greater than 2σ(F2). The final cell constants of a = 18.7106(17) Å, b = 6.3498(5) Å, c = 19.9016(17) Å, β _= 103.734(3)°, volume = 2296.9(3) Å3, are based upon the refinement of the XYZ-centroids of reflections above 20 σ(I). Data were corrected for absorption effects using the multi-scan method (SADABS). The calculated minimum and maximum transmission coefficients (based on crystal size) are 0.5346 and 0.7461.

The structure was solved and refined using the Bruker SHELXTL Software Package, using the space group *P*21, with Z = 2 for the formula unit, C48H56B2Br2O8. The final anisotropic full-matrix least-squares refinement on F2 with 572 variables converged at R1 = 6.88%, for the observed data and wR2 = 12.94% for all data. The goodness-of-fit was 1.008. The largest peak in the final difference electron density synthesis was 0.521 e-/Å3 and the largest hole was -0.874 e-/Å3 with an RMS deviation of 0.090 e-/Å3. On the basis of the final model, the calculated density was 1.363 g/cm3 and F(000), 976 e-.

Table S9 . Sample and crystal data for **3c**

Identification code	O42ACB021	
Chemical formula	$C_{48}H_{56}B_2Br_2O_8$	
Formula weight	942.36 g/mol	
Temperature	100(2) K	
Wavelength	0.71073 Å	
Crystal system	monoclinic	
Space group	P 1 21 1	
Unit cell dimensions	a = 18.7106(17) Å	α = 90°_
	b = 6.3498(5) Å	B = 103.734(3)°
	c = 19.9016(17) Å	γ = 90°
Volume	2296.9(3) Å3	
Z	2	
Density (calculated)	1.363 g/cm3	
Absorption coefficient	1.816 mm-1	
F(000)	976	

Table S10. Data collection and structure refinement for **3c**

Theta range for data collection	1.71 to 30.58°	
Index ranges	-26<=h<=25, -9<=k<=9, -27<=l<=28	
Reflections collected	68645	
Independent reflections	14087 [R(int) = 0.1461]	
Refinement method	Full-matrix least-squares on F2	
Refinement program	SHELXL-2019/1 (Sheldrick, 2019)	
Function minimized	Σ w(Fo2 – Fc2)2	
Data / restraints / parameters	14087 / 1 / 572	
Goodness-of-fit on F2	1.008	
Δ/σmax	0.001	
Final R indices	8324 data; I>2σ(I)	R1 = 0.0688, wR2 = 0.1085
	all data	R1 = 0.1404, wR2 = 0.1294
Weighting scheme	w=1/[σ2(F2)+(0.0220P)2+1.9640P]<br>where P=(F 2+2F 2)/3	
Absolute structure parameter	0.034(12)	
Largest diff. peak and hole	0.521 and -0.874 Å-3	
R.M.S. deviation from mean	0.090 eÅ-3	

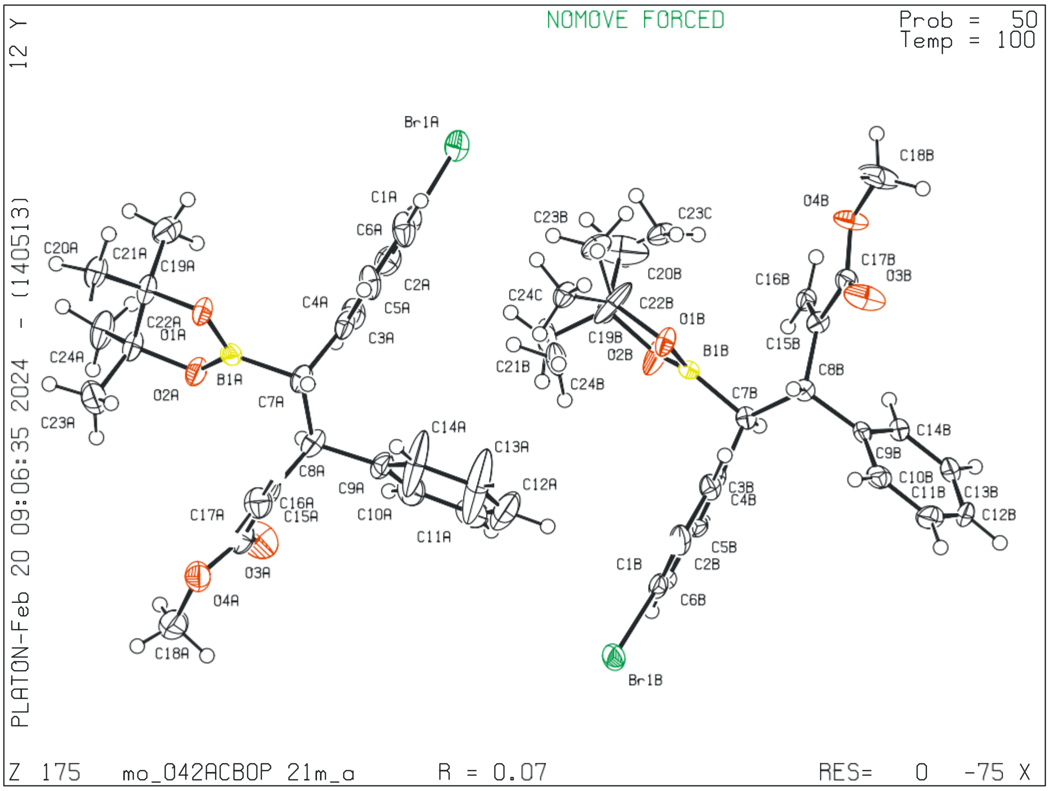

Figure S23. ORTEP diagram and atomic numbering of **3c**. Thermal ellipsoids drawn at the 50% probability level.

C7A	Chiral: S
C8A	Chiral: S
C7B	Chiral: S
C8B	Chiral: S

Table S11. Atomic coordinates and equivalent isotropic atomic displacement parameters (Å^2) for 3a U(eq) is defined as one third of the trace of the orthogonalized U_{ij} tensor.

	**x/a**	**y/b**	**z/c**	**U(eq)**
Br1A	0.36558(4)	0.12063(11)	0.98971(4)	0.0405(2)
Br1B	0.14705(4)	0.07364(10)	0.48361(4)	0.03663(19)
O1A	0.6542(2)	0.4331(7)	0.8560(2)	0.0285(10)
O1B	0.1270(2)	0.6909(6)	0.7990(2)	0.0258(10)
O2A	0.6236(2)	0.1952(7)	0.7681(2)	0.0314(11)
O2B	0.1022(3)	0.3623(7)	0.7569(3)	0.0421(13)
O3A	0.5118(3)	0.2343(8)	0.5761(3)	0.0528(15)
O3B	0.9333(3)	0.9959(7)	0.8562(2)	0.0448(15)
O4A	0.5973(3)	0.4646(8)	0.5615(3)	0.0394(13)
O4B	0.9405(3)	0.7066(7)	0.9189(2)	0.0350(12)
C1A	0.4102(4)	0.2309(12)	0.9210(3)	0.0313(17)
C1B	0.0998(4)	0.9471(11)	0.5481(3)	0.0272(15)
B1A	0.6035(4)	0.3666(11)	0.7992(4)	0.0225(15)
C2A	0.4172(4)	0.1097(12)	0.8662(3)	0.0351(16)
C3A	0.4526(4)	0.1907(11)	0.8172(4)	0.0340(17)
C4A	0.4797(4)	0.3928(11)	0.8231(3)	0.0261(15)

C5A	0.4717(4)	0.5098(11)	0.8788(4)	0.0338(18)
C6A	0.4376(4)	0.4341(13)	0.9284(4)	0.0380(19)
C7A	0.5252(4)	0.4750(10)	0.7744(3)	0.0254(15)
C8A	0.4914(4)	0.4337(11)	0.6971(3)	0.0287(15)
C9A	0.4129(4)	0.5140(10)	0.6725(3)	0.0302(16)
C10A	0.3604(4)	0.3906(15)	0.6313(4)	0.047(2)
C11A	0.2897(5)	0.463(2)	0.6056(5)	0.082(4)
C12A	0.2704(5)	0.657(2)	0.6219(7)	0.091(5)
C13A	0.3214(6)	0.7818(19)	0.6631(9)	0.132(7)
C14A	0.3928(5)	0.7113(13)	0.6886(7)	0.089(4)
C15A	0.5391(3)	0.5276(10)	0.6530(3)	0.0269(15)
C16A	0.5690(4)	0.7189(12)	0.6606(4)	0.0379(18)
C17A	0.5471(4)	0.3915(11)	0.5933(4)	0.0328(17)
C18A	0.6029(5)	0.3508(14)	0.4999(4)	0.047(2)
C19A	0.7088(4)	0.2651(11)	0.8712(4)	0.0299(15)
C20A	0.7822(4)	0.3601(12)	0.9042(4)	0.0371(17)
C21A	0.6847(4)	0.1107(15)	0.9222(4)	0.049(2)
C22A	0.7013(4)	0.1604(10)	0.8002(3)	0.0308(16)
C23A	0.7463(4)	0.2832(13)	0.7570(4)	0.046(2)
C24A	0.7185(4)	0.9279(11)	0.8004(4)	0.0394(19)
B1B	0.0802(3)	0.5647(11)	0.7542(3)	0.0177(12)
C2B	0.0963(4)	0.0579(12)	0.6073(3)	0.0304(16)
C3B	0.0649(4)	0.9632(10)	0.6554(3)	0.0240(14)
C4B	0.0362(3)	0.7603(10)	0.6456(3)	0.0177(13)
C5B	0.0409(3)	0.6553(10)	0.5859(3)	0.0219(13)
C6B	0.0724(3)	0.7468(11)	0.5370(3)	0.0250(14)
C7B	0.0090(3)	0.6469(9)	0.7018(3)	0.0180(12)
C8B	0.9606(3)	0.7812(9)	0.7391(3)	0.0191(13)
C9B	0.8867(3)	0.8318(9)	0.6906(3)	0.0214(13)
C10B	0.8750(4)	0.0254(10)	0.6557(3)	0.0283(15)
C11B	0.8089(4)	0.0651(13)	0.6090(3)	0.0392(19)
C12B	0.7536(4)	0.9174(13)	0.5957(3)	0.0375(18)
C13B	0.7640(4)	0.7278(12)	0.6305(3)	0.0342(16)
C14B	0.8300(3)	0.6870(10)	0.6770(3)	0.0257(14)
C15B	0.9503(4)	0.6695(9)	0.8040(3)	0.0198(13)
C16B	0.9498(4)	0.4643(10)	0.8124(3)	0.0205(14)
C17B	0.9408(4)	0.8113(10)	0.8607(3)	0.0243(14)
C18B	0.9310(6)	0.8311(13)	0.9759(4)	0.054(3)
C19B	0.1703(4)	0.3440(10)	0.8104(3)	0.0265(14)
C20B	0.1538(6)	0.1950(12)	0.8629(4)	0.066(3)
C21B	0.2247(4)	0.250(2)	0.7753(5)	0.083(4)
C22B	0.1864(4)	0.5677(12)	0.8372(4)	0.055(2)
C23B	0.2227(7)	0.595(2)	0.9042(6)	0.032(3)
C24B	0.2473(7)	0.650(2)	0.7868(7)	0.030(3)
C23C	0.1491(7)	0.593(2)	0.9120(5)	0.028(3)
C24C	0.2552(7)	0.671(2)	0.8601(7)	0.027(3)

Table S12. Bond lengths (Å) for **3c**

Br1A-C1A	1.897(7)	Br1B-C1B	1.901(7)
O1A-B1A	1.357(8)	O1A-C19A	1.458(7)
O1B-B1B	1.353(7)	O1B-C22B	1.423(8)
O2A-B1A	1.349(8)	O2A-C22A	1.460(7)
O2B-B1B	1.346(8)	O2B-C19B	1.458(8)
O3A-C17A	1.201(9)	O3B-C17B	1.182(7)
O4A-C17A	1.335(9)	O4A-C18A	1.448(9)
O4B-C17B	1.336(8)	O4B-C18B	1.428(8)
C1A-C2A	1.366(9)	C1A-C6A	1.384(11)
C1B-C6B	1.370(9)	C1B-C2B	1.386(9)
B1A-C7A	1.589(9)	C2A-C3A	1.401(10)
C2A-H2A	0.950000	C3A-C4A	1.375(10)
C3A-H3A	0.950000	C4A-C5A	1.372(9)
C4A-C7A	1.525(10)	C5A-C6A	1.382(10)
C5A-H5A	0.950000	C6A-H6A	0.950000
C7A-C8A	1.541(8)	C7A-H7A	1.000000
C8A-C15A	1.513(10)	C8A-C9A	1.522(9)
C8A-H8A	1.000000	C9A-C10A	1.367(9)
C9A-C14A	1.368(11)	C10A-C11A	1.379(13)
C10A-H10A	0.950000	C11A-C12A	1.344(17)
C11A-H11A	0.950000	C12A-C13A	1.359(18)
C12A-H12A	0.950000	C13A-C14A	1.386(13)
C13A-H13A	0.950000	C14A-H14A	0.950000
C15A-C16A	1.331(10)	C15A-C17A	1.505(10)
C16A-H16A	0.950000	C16A-H16B	0.950000
C18A-H18A	0.980000	C18A-H18B	0.980000
C18A-H18C	0.980000	C19A-C20A	1.500(9)
C19A-C22A	1.538(9)	C19A-C21A	1.554(10)
C20A-H20A	0.980000	C20A-H20B	0.980000
C20A-H20C	0.980000	C21A-H21A	0.980000
C21A-H21B	0.980000	C21A-H21C	0.980000
C22A-C24A	1.511(9)	C22A-C23A	1.548(10)
C23A-H23A	0.980000	C23A-H23B	0.980000
C23A-H23C	0.980000	C24A-H24A	0.980000
C24A-H24B	0.980000	C24A-H24C	0.980000
B1B-C7B	1.574(8)	C2B-C3B	1.375(9)
C2B-H2B	0.950000	C3B-C4B	1.392(9)
C3B-H3B	0.950000	C4B-C5B	1.384(8)
C4B-C7B	1.517(8)	C5B-C6B	1.380(9)
C5B-H5B	0.950000	C6B-H6B	0.950000
C7B-C8B	1.554(8)	C7B-H7B	1.000000
C8B-C9B	1.521(8)	C8B-C15B	1.526(8)
C8B-H8B	1.000000	C9B-C14B	1.381(9)
C9B-C10B	1.403(8)	C10B-C11B	1.382(9)
C10B-H10B	0.950000	C11B-C12B	1.375(11)
C11B-H11B	0.950000	C12B-C13B	1.380(10)
C12B-H12B	0.950000	C13B-C14B	1.380(9)

C13B-H13B	0.950000	C14B-H14B	0.950000
C15B-C16B	1.314(8)	C15B-C17B	1.487(9)
C16B-H16C	0.950000	C16B-H16D	0.950000
C18B-H18D	0.980000	C18B-H18E	0.980000
C18B-H18F	0.980000	C19B-C21B	1.489(10)
C19B-C20B	1.495(9)	C19B-C22B	1.522(10)
C20B-H20D	0.980000	C20B-H20E	0.980000
C20B-H20F	0.980000	C21B-H21D	0.980000
C21B-H21E	0.980000	C21B-H21F	0.980000
C22B-C23B	1.357(13)	C22B-C24C	1.420(14)
C22B-C24B	1.767(15)	C22B-C23C	1.794(15)
C23B-H23D	0.980000	C23B-H23E	0.980000
C23B-H23F	0.980000	C24B-H24D	0.980000
C24B-H24E	0.980000	C24B-H24F	0.980000
C23C-H23G	0.980000	C23C-H23H	0.980000
C23C-H23I	0.980000	C24C-H24G	0.980000
C24C-H24H	0.980000	C24C-H24I	0.980000

Table S13. Bond angles (°) for **3c**

B1A-O1A-C19A	105.2(5)	B1B-O1B-C22B	109.1(5)
B1A-O2A-C22A	106.1(5)	B1B-O2B-C19B	108.1(5)
C17A-O4A-C18A	115.3(7)	C17B-O4B-C18B	116.1(6)
C2A-C1A-C6A	120.4(7)	C2A-C1A-Br1A	120.6(6)
C6A-C1A-Br1A	118.9(6)	C6B-C1B-C2B	121.2(6)
C6B-C1B-Br1B	119.9(5)	C2B-C1B-Br1B	118.8(5)
O2A-B1A-O1A	114.7(6)	O2A-B1A-C7A	122.9(6)
O1A-B1A-C7A	122.3(6)	C1A-C2A-C3A	120.0(7)
C1A-C2A-H2A	120.000000	C3A-C2A-H2A	120.000000
C4A-C3A-C2A	120.5(7)	C4A-C3A-H3A	119.700000
C2A-C3A-H3A	119.700000	C5A-C4A-C3A	117.8(7)
C5A-C4A-C7A	120.8(6)	C3A-C4A-C7A	121.0(6)
C4A-C5A-C6A	123.1(7)	C4A-C5A-H5A	118.500000
C6A-C5A-H5A	118.500000	C5A-C6A-C1A	118.1(7)
C5A-C6A-H6A	120.900000	C1A-C6A-H6A	120.900000
C4A-C7A-C8A	114.8(6)	C4A-C7A-B1A	105.5(5)
C8A-C7A-B1A	111.4(5)	C4A-C7A-H7A	108.300000
C8A-C7A-H7A	108.300000	B1A-C7A-H7A	108.300000
C15A-C8A-C9A	109.8(5)	C15A-C8A-C7A	110.8(5)
C9A-C8A-C7A	113.5(6)	C15A-C8A-H8A	107.500000
C9A-C8A-H8A	107.500000	C7A-C8A-H8A	107.500000
C10A-C9A-C14A	117.8(8)	C10A-C9A-C8A	120.1(7)
C14A-C9A-C8A	122.0(7)	C9A-C10A-C11A	121.4(10)
C9A-C10A-H10A	119.300000	C11A-C10A-H10A	119.300000
C12A-C11A-C10A	120.3(11)	C12A-C11A-H11A	119.800000
C10A-C11A-H11A	119.800000	C11A-C12A-C13A	119.4(10)
C11A-C12A-H12A	120.300000	C13A-C12A-H12A	120.300000
C12A-C13A-C14A	120.7(12)	C12A-C13A-H13A	119.700000

C14A-C13A-H13A	119.700000	C9A-C14A-C13A	120.4(10)
C9A-C14A-H14A	119.800000	C13A-C14A-H14A	119.800000
C16A-C15A-C17A	119.7(7)	C16A-C15A-C8A	125.7(7)
C17A-C15A-C8A	114.5(6)	C15A-C16A-H16A	120.000000
C15A-C16A-H16B	120.000000	H16A-C16A-H16B	120.000000
O3A-C17A-O4A	123.7(7)	O3A-C17A-C15A	123.4(7)
O4A-C17A-C15A	112.8(6)	O4A-C18A-H18A	109.500000
O4A-C18A-H18B	109.500000	H18A-C18A-H18B	109.500000
O4A-C18A-H18C	109.500000	H18A-C18A-H18C	109.500000
H18B-C18A-H18C	109.500000	O1A-C19A-C20A	108.7(6)
O1A-C19A-C22A	102.6(5)	C20A-C19A-C22A	116.7(6)
O1A-C19A-C21A	107.5(6)	C20A-C19A-C21A	110.2(6)
C22A-C19A-C21A	110.5(6)	C19A-C20A-H20A	109.500000
C19A-C20A-H20B	109.500000	H20A-C20A-H20B	109.500000
C19A-C20A-H20C	109.500000	H20A-C20A-H20C	109.500000
H20B-C20A-H20C	109.500000	C19A-C21A-H21A	109.500000
C19A-C21A-H21B	109.500000	H21A-C21A-H21B	109.500000
C19A-C21A-H21C	109.500000	H21A-C21A-H21C	109.500000
H21B-C21A-H21C	109.500000	O2A-C22A-C24A	109.8(5)
O2A-C22A-C19A	101.5(5)	C24A-C22A-C19A	116.5(6)
O2A-C22A-C23A	107.3(5)	C24A-C22A-C23A	110.6(6)
C19A-C22A-C23A	110.4(6)	C22A-C23A-H23A	109.500000
C22A-C23A-H23B	109.500000	H23A-C23A-H23B	109.500000
C22A-C23A-H23C	109.500000	H23A-C23A-H23C	109.500000
H23B-C23A-H23C	109.500000	C22A-C24A-H24A	109.500000
C22A-C24A-H24B	109.500000	H24A-C24A-H24B	109.500000
C22A-C24A-H24C	109.500000	H24A-C24A-H24C	109.500000
H24B-C24A-H24C	109.500000	O2B-B1B-O1B	113.3(5)
O2B-B1B-C7B	123.1(5)	O1B-B1B-C7B	123.6(5)
C3B-C2B-C1B	118.9(7)	C3B-C2B-H2B	120.500000
C1B-C2B-H2B	120.500000	C2B-C3B-C4B	121.4(6)
C2B-C3B-H3B	119.300000	C4B-C3B-H3B	119.300000
C5B-C4B-C3B	117.8(6)	C5B-C4B-C7B	120.4(6)
C3B-C4B-C7B	121.5(5)	C6B-C5B-C4B	121.8(6)
C6B-C5B-H5B	119.100000	C4B-C5B-H5B	119.100000
C1B-C6B-C5B	118.9(6)	C1B-C6B-H6B	120.600000
C5B-C6B-H6B	120.600000	C4B-C7B-C8B	115.3(5)
C4B-C7B-B1B	105.5(5)	C8B-C7B-B1B	111.3(4)
C4B-C7B-H7B	108.200000	C8B-C7B-H7B	108.200000
B1B-C7B-H7B	108.200000	C9B-C8B-C15B	110.9(5)
C9B-C8B-C7B	111.0(5)	C15B-C8B-C7B	110.7(5)
C9B-C8B-H8B	108.000000	C15B-C8B-H8B	108.000000
C7B-C8B-H8B	108.000000	C14B-C9B-C10B	117.6(6)
C14B-C9B-C8B	121.6(5)	C10B-C9B-C8B	120.7(6)
C11B-C10B-C9B	120.2(7)	C11B-C10B-H10B	119.900000
C9B-C10B-H10B	119.900000	C12B-C11B-C10B	121.1(7)
C12B-C11B-H11B	119.400000	C10B-C11B-H11B	119.400000
C11B-C12B-C13B	119.3(7)	C11B-C12B-H12B	120.400000

C13B-C12B-H12B	120.400000	C14B-C13B-C12B	119.8(7)
C14B-C13B-H13B	120.100000	C12B-C13B-H13B	120.100000
C13B-C14B-C9B	122.0(6)	C13B-C14B-H14B	119.000000
C9B-C14B-H14B	119.000000	C16B-C15B-C17B	119.9(6)
C16B-C15B-C8B	125.0(6)	C17B-C15B-C8B	115.0(5)
C15B-C16B-H16C	120.000000	C15B-C16B-H16D	120.000000
H16C-C16B-H16D	120.000000	O3B-C17B-O4B	122.4(6)
O3B-C17B-C15B	125.2(6)	O4B-C17B-C15B	112.5(5)
O4B-C18B-H18D	109.500000	O4B-C18B-H18E	109.500000
H18D-C18B-H18E	109.500000	O4B-C18B-H18F	109.500000
H18D-C18B-H18F	109.500000	H18E-C18B-H18F	109.500000
O2B-C19B-C21B	105.4(6)	O2B-C19B-C20B	105.7(6)
C21B-C19B-C20B	110.6(8)	O2B-C19B-C22B	104.3(5)
C21B-C19B-C22B	115.9(7)	C20B-C19B-C22B	113.7(6)
C19B-C20B-H20D	109.500000	C19B-C20B-H20E	109.500000
H20D-C20B-H20E	109.500000	C19B-C20B-H20F	109.500000
H20D-C20B-H20F	109.500000	H20E-C20B-H20F	109.500000
C19B-C21B-H21D	109.500000	C19B-C21B-H21E	109.500000
H21D-C21B-H21E	109.500000	C19B-C21B-H21F	109.500000
H21D-C21B-H21F	109.500000	H21E-C21B-H21F	109.500000
C23B-C22B-O1B	126.9(9)	C24C-C22B-O1B	116.9(8)
C23B-C22B-C19B	118.4(8)	C24C-C22B-C19B	129.1(9)
O1B-C22B-C19B	105.2(5)	C23B-C22B-C24B	106.2(9)
O1B-C22B-C24B	93.6(6)	C19B-C22B-C24B	100.2(7)
C24C-C22B-C23C	101.2(8)	O1B-C22B-C23C	89.2(6)
C19B-C22B-C23C	106.9(7)	C22B-C23B-H23D	109.500000
C22B-C23B-H23E	109.500000	H23D-C23B-H23E	109.500000
C22B-C23B-H23F	109.500000	H23D-C23B-H23F	109.500000
H23E-C23B-H23F	109.500000	C22B-C24B-H24D	109.500000
C22B-C24B-H24E	109.500000	H24D-C24B-H24E	109.500000
C22B-C24B-H24F	109.500000	H24D-C24B-H24F	109.500000
H24E-C24B-H24F	109.500000	C22B-C23C-H23G	109.500000
C22B-C23C-H23H	109.500000	H23G-C23C-H23H	109.500000
C22B-C23C-H23I	109.500000	H23G-C23C-H23I	109.500000
H23H-C23C-H23I	109.500000	C22B-C24C-H24G	109.500000
C22B-C24C-H24H	109.500000	H24G-C24C-H24H	109.500000
C22B-C24C-H24I	109.500000	H24G-C24C-H24I	109.500000
H24H-C24C-H24I	109.500000		

Table S14. Torsion angles (°) for **3c**

C22A-O2A-B1A-O1A	-10.1(7)	C22A-O2A-B1A-C7A	173.2(6)
C19A-O1A-B1A-O2A	-10.8(7)	C19A-O1A-B1A-C7A	165.9(6)
C6A-C1A-C2A-C3A	0.2(10)	Br1A-C1A-C2A-C3A	-177.7(5)
C1A-C2A-C3A-C4A	-0.8(10)	C2A-C3A-C4A-C5A	0.8(10)
C2A-C3A-C4A-C7A	173.7(6)	C3A-C4A-C5A-C6A	-0.3(11)
C7A-C4A-C5A-C6A	-173.2(6)	C4A-C5A-C6A-C1A	-0.3(11)
C2A-C1A-C6A-C5A	0.3(10)	Br1A-C1A-C6A-C5A	178.3(5)

C5A-C4A-C7A-C8A	-138.8(6)	C3A-C4A-C7A-C8A	48.5(8)
C5A-C4A-C7A-B1A	98.1(7)	C3A-C4A-C7A-B1A	-74.5(8)
O2A-B1A-C7A-C4A	99.2(7)	O1A-B1A-C7A-C4A	-77.2(7)
O2A-B1A-C7A-C8A	-26.0(9)	O1A-B1A-C7A-C8A	157.6(6)
C4A-C7A-C8A-C15A	178.0(5)	B1A-C7A-C8A-C15A	-62.2(7)
C4A-C7A-C8A-C9A	54.0(7)	B1A-C7A-C8A-C9A	173.7(5)
C15A-C8A-C9A-C10A	100.2(7)	C7A-C8A-C9A-C10A	-135.2(7)
C15A-C8A-C9A-C14A	-77.5(10)	C7A-C8A-C9A-C14A	47.0(11)
C14A-C9A-C10A-C11A	0.9(13)	C8A-C9A-C10A-C11A	-176.9(7)
C9A-C10A-C11A-C12A	-1.3(15)	C10A-C11A-C12A-C13A	0.9(17)
C11A-C12A-C13A-C14A	0.(2)	C10A-C9A-C14A-C13A	-0.3(17)
C8A-C9A-C14A-C13A	177.5(11)	C12A-C13A-C14A-C9A	0.(2)
C9A-C8A-C15A-C16A	81.1(8)	C7A-C8A-C15A-C16A	-45.0(8)
C9A-C8A-C15A-C17A	-94.6(7)	C7A-C8A-C15A-C17A	139.3(6)
C18A-O4A-C17A-O3A	5.0(10)	C18A-O4A-C17A-C15A	-174.7(6)
C16A-C15A-C17A-O3A	-166.8(7)	C8A-C15A-C17A-O3A	9.2(10)
C16A-C15A-C17A-O4A	12.9(9)	C8A-C15A-C17A-O4A	-171.2(5)
B1A-O1A-C19A-C20A	149.9(6)	B1A-O1A-C19A-C22A	25.6(7)
B1A-O1A-C19A-C21A	-90.9(6)	B1A-O2A-C22A-C24A	149.0(6)
B1A-O2A-C22A-C19A	25.1(6)	B1A-O2A-C22A-C23A	-90.8(6)
O1A-C19A-C22A-O2A	-30.7(6)	C20A-C19A-C22A-O2A	-149.5(6)
C21A-C19A-C22A-O2A	83.6(6)	O1A-C19A-C22A-C24A	-150.0(6)
C20A-C19A-C22A-C24A	91.3(8)	C21A-C19A-C22A-C24A	-35.6(8)
O1A-C19A-C22A-C23A	82.8(6)	C20A-C19A-C22A-C23A	-35.9(8)
C21A-C19A-C22A-C23A	-162.9(6)	C19B-O2B-B1B-O1B	1.7(7)
C19B-O2B-B1B-C7B	179.2(5)	C22B-O1B-B1B-O2B	-0.9(8)
C22B-O1B-B1B-C7B	-178.3(6)	C6B-C1B-C2B-C3B	0.3(9)
Br1B-C1B-C2B-C3B	-177.3(5)	C1B-C2B-C3B-C4B	-0.6(9)
C2B-C3B-C4B-C5B	0.6(9)	C2B-C3B-C4B-C7B	173.4(6)
C3B-C4B-C5B-C6B	-0.4(9)	C7B-C4B-C5B-C6B	-173.2(5)
C2B-C1B-C6B-C5B	0.0(9)	Br1B-C1B-C6B-C5B	177.5(5)
C4B-C5B-C6B-C1B	0.1(9)	C5B-C4B-C7B-C8B	-142.7(5)
C3B-C4B-C7B-C8B	44.7(7)	C5B-C4B-C7B-B1B	94.0(6)
C3B-C4B-C7B-B1B	-78.6(7)	O2B-B1B-C7B-C4B	-103.2(7)
O1B-B1B-C7B-C4B	74.1(7)	O2B-B1B-C7B-C8B	131.1(6)
O1B-B1B-C7B-C8B	-51.7(7)	C4B-C7B-C8B-C9B	68.6(6)
B1B-C7B-C8B-C9B	-171.3(5)	C4B-C7B-C8B-C15B	-167.7(5)
B1B-C7B-C8B-C15B	-47.7(6)	C15B-C8B-C9B-C14B	-43.7(7)
C7B-C8B-C9B-C14B	79.9(7)	C15B-C8B-C9B-C10B	139.2(6)
C7B-C8B-C9B-C10B	-97.3(6)	C14B-C9B-C10B-C11B	-0.8(9)
C8B-C9B-C10B-C11B	176.5(5)	C9B-C10B-C11B-C12B	0.1(10)
C10B-C11B-C12B-C13B	1.0(10)	C11B-C12B-C13B-C14B	-1.4(10)
C12B-C13B-C14B-C9B	0.7(10)	C10B-C9B-C14B-C13B	0.4(9)
C8B-C9B-C14B-C13B	-176.8(6)	C9B-C8B-C15B-C16B	93.9(8)
C7B-C8B-C15B-C16B	-29.8(9)	C9B-C8B-C15B-C17B	-86.4(6)
C7B-C8B-C15B-C17B	149.9(5)	C18B-O4B-C17B-O3B	-0.3(11)
C18B-O4B-C17B-C15B	-179.5(7)	C16B-C15B-C17B-O3B	-172.3(8)
C8B-C15B-C17B-O3B	8.1(10)	C16B-C15B-C17B-O4B	6.9(10)

C8B-C15B-C17B-O4B	-172.8(5)	B1B-O2B-C19B-C21B	-124.3(8)
B1B-O2B-C19B-C20B	118.5(6)	B1B-O2B-C19B-C22B	-1.8(8)
B1B-O1B-C22B-C23B	-145.4(10)	B1B-O1B-C22B-C24C	150.2(9)
B1B-O1B-C22B-C19B	-0.3(8)	B1B-O1B-C22B-C24B	101.2(6)
B1B-O1B-C22B-C23C	-107.7(6)	O2B-C19B-C22B-C23B	149.9(9)
C21B-C19B-C22B-C23B	-94.7(11)	C20B-C19B-C22B-C23B	35.2(12)
O2B-C19B-C22B-C24C	-144.3(10)	C21B-C19B-C22B-C24C	-28.9(13)
C20B-C19B-C22B-C24C	101.0(12)	O2B-C19B-C22B-O1B	1.2(8)
C21B-C19B-C22B-O1B	116.6(7)	C20B-C19B-C22B-O1B	-113.5(7)
O2B-C19B-C22B-C24B	-95.3(7)	C21B-C19B-C22B-C24B	20.1(8)
C20B-C19B-C22B-C24B	150.0(8)	O2B-C19B-C22B-C23C	95.1(6)
C21B-C19B-C22B-C23C	-149.5(7)	C20B-C19B-C22B-C23C	-19.6(9)

Table S15. Anisotropic atomic displacement parameters (Å^2) for **3c.** The anisotropic atomic displacement factor exponent takes the form: $-2\pi^2[h^2 a^{*2} U_{11} + ... + 2 h k a^* b^* U_{12}]$

	U11	U22	U33	U23	U13	U12
Br1A	0.0253(4)	0.0522(5)	0.0430(4)	0.0007(4)	0.0058(3)	0.0010(3)
Br1B	0.0270(4)	0.0444(5)	0.0428(4)	0.0194(3)	0.0169(3)	0.0053(3)
O1A	0.018(2)	0.031(3)	0.034(3)	-0.0013(19)	0.0006(19)	0.0061(19)
O1B	0.018(2)	0.022(2)	0.033(2)	-0.0087(17)	-0.0025(18)	0.0050(17)
O2A	0.021(2)	0.037(3)	0.032(2)	-0.0076(19)	-0.0014(19)	0.0067(19)
O2B	0.025(3)	0.014(2)	0.073(3)	-0.008(2)	-0.017(2)	0.0024(18)
O3A	0.065(4)	0.039(3)	0.056(4)	-0.021(3)	0.019(3)	-0.015(3)
O3B	0.091(5)	0.016(3)	0.035(3)	0.0005(19)	0.030(3)	0.005(2)
O4A	0.029(3)	0.050(3)	0.039(3)	-0.006(2)	0.008(2)	0.001(2)
O4B	0.060(4)	0.030(3)	0.018(2)	-0.0003(19)	0.016(2)	0.000(2)
C1A	0.014(3)	0.049(5)	0.031(4)	-0.002(3)	0.004(3)	0.004(3)
C1B	0.017(3)	0.028(4)	0.036(4)	0.015(3)	0.006(3)	0.005(3)
B1A	0.020(4)	0.026(4)	0.023(4)	0.001(3)	0.007(3)	-0.003(3)
C2A	0.035(4)	0.027(4)	0.041(4)	0.002(3)	0.005(3)	0.009(3)
C3A	0.029(4)	0.035(4)	0.038(4)	-0.012(3)	0.008(3)	0.006(3)
C4A	0.016(3)	0.028(4)	0.032(4)	-0.007(3)	-0.002(3)	0.003(3)
C5A	0.019(4)	0.033(4)	0.050(5)	-0.011(3)	0.011(3)	-0.008(3)
C6A	0.023(4)	0.042(5)	0.048(5)	-0.020(4)	0.008(3)	-0.003(3)
C7A	0.023(3)	0.018(3)	0.033(4)	-0.003(3)	0.001(3)	0.004(3)
C8A	0.024(4)	0.025(4)	0.032(4)	-0.002(3)	-0.004(3)	0.003(3)
C9A	0.018(3)	0.034(4)	0.035(4)	0.008(3)	-0.001(3)	-0.003(3)
C10A	0.024(4)	0.075(6)	0.040(4)	-0.007(4)	0.004(3)	-0.019(4)
C11A	0.023(5)	0.186(14)	0.033(5)	0.005(7)	0.000(4)	-0.036(7)
C12A	0.027(5)	0.136(12)	0.106(9)	0.089(9)	0.005(6)	0.007(7)
C13A	0.024(6)	0.056(7)	0.30(2)	0.067(10)	0.008(9)	0.006(5)
C14A	0.019(4)	0.025(5)	0.205(13)	-0.003(6)	-0.009(6)	0.004(3)
C15A	0.015(3)	0.029(4)	0.030(4)	0.000(3)	-0.006(3)	0.000(3)
C16A	0.035(5)	0.037(4)	0.042(5)	-0.003(3)	0.009(4)	-0.002(3)
C17A	0.027(4)	0.028(4)	0.040(4)	-0.004(3)	0.002(3)	0.007(3)
C18A	0.044(5)	0.055(5)	0.039(5)	0.001(4)	0.007(4)	0.013(4)
C19A	0.017(3)	0.029(4)	0.041(4)	0.001(3)	0.002(3)	0.004(3)
C20A	0.020(4)	0.043(4)	0.044(4)	-0.002(3)	-0.001(3)	-0.001(3)

C21A	0.036(4)	0.066(6)	0.042(4)	0.021(4)	0.004(3)	0.004(5)
C22A	0.019(3)	0.030(4)	0.040(4)	-0.003(3)	0.000(3)	0.006(3)
C23A	0.038(5)	0.048(5)	0.060(5)	0.011(4)	0.025(4)	0.007(4)
C24A	0.027(4)	0.023(4)	0.064(5)	-0.009(3)	0.001(4)	0.006(3)
B1B	0.020(3)	0.018(3)	0.016(3)	0.001(3)	0.006(2)	-0.001(3)
C2B	0.024(3)	0.020(4)	0.049(4)	0.010(3)	0.014(3)	0.005(3)
C3B	0.025(4)	0.016(3)	0.033(4)	0.002(3)	0.011(3)	0.005(3)
C4B	0.006(3)	0.026(3)	0.020(3)	0.004(2)	0.000(2)	0.003(2)
C5B	0.020(3)	0.024(3)	0.020(3)	-0.003(2)	0.002(2)	0.000(3)
C6B	0.018(3)	0.038(4)	0.019(3)	0.005(3)	0.004(3)	0.003(3)
C7B	0.019(3)	0.015(3)	0.021(3)	-0.006(2)	0.005(2)	-0.002(2)
C8B	0.022(3)	0.014(3)	0.022(3)	-0.001(2)	0.006(3)	0.000(2)
C9B	0.020(3)	0.025(3)	0.021(3)	0.001(2)	0.010(3)	0.006(2)
C10B	0.036(4)	0.028(4)	0.024(3)	-0.001(3)	0.012(3)	0.009(3)
C11B	0.051(5)	0.040(4)	0.029(3)	0.012(3)	0.016(3)	0.029(4)
C12B	0.021(4)	0.064(5)	0.027(4)	0.007(3)	0.004(3)	0.021(4)
C13B	0.019(3)	0.057(5)	0.028(4)	0.002(3)	0.010(3)	0.002(3)
C14B	0.021(3)	0.034(4)	0.024(3)	0.004(3)	0.008(3)	0.006(3)
C15B	0.024(3)	0.013(3)	0.021(3)	-0.002(2)	0.003(3)	0.001(2)
C16B	0.019(3)	0.020(3)	0.022(3)	0.000(2)	0.004(3)	0.003(3)
C17B	0.026(4)	0.022(4)	0.026(3)	0.002(3)	0.009(3)	0.004(3)
C18B	0.094(8)	0.047(5)	0.029(4)	-0.002(4)	0.032(5)	0.010(5)
C19B	0.018(3)	0.022(3)	0.037(4)	0.008(3)	0.002(3)	0.002(2)
C20B	0.126(9)	0.033(5)	0.039(5)	-0.006(3)	0.019(5)	-0.043(5)
C21B	0.019(4)	0.181(12)	0.049(5)	-0.035(6)	0.007(4)	0.014(6)
C22B	0.042(4)	0.018(4)	0.080(5)	0.019(4)	-0.037(4)	-0.004(3)
C23B	0.041(8)	0.023(7)	0.026(6)	-0.002(6)	-0.003(5)	0.007(6)
C24B	0.022(6)	0.020(7)	0.052(8)	-0.001(6)	0.017(6)	-0.003(5)
C23C	0.039(7)	0.021(7)	0.021(6)	-0.001(5)	0.001(5)	0.009(6)
C24C	0.021(7)	0.030(8)	0.027(7)	-0.003(5)	-0.002(5)	-0.001(5)

Table S16. Hydrogen atomic coordinates and isotropic atomic displacement parameters (Å^2) for **3c**

	**x/a**	**y/b**	**z/c**	**U(eq)**
H2A	0.3982	-0.0295	0.8614	0.042000
H3A	0.4579	0.1052	0.7795	0.041000
H5A	0.4905	0.6494	0.8835	0.041000
H6A	0.4332	0.5192	0.9664	0.046000
H7A	0.5315	0.6305	0.7815	0.031000
H8A	0.4904	0.2778	0.6899	0.034000
H10A	0.3730	0.2522	0.6200	0.056000
H11A	0.2545	0.3751	0.5762	0.098000
H12A	0.2215	0.7055	0.6047	0.109000
H13A	0.3080	0.9191	0.6746	0.158000
H14A	0.4280	0.8005	0.7173	0.107000
H16A	0.5953	0.7689	0.6284	0.045000
H16B	0.5641	0.8054	0.6982	0.045000

H18A	0.6453	0.4029	0.4839	0.070000
H18B	0.5579	0.3721	0.4636	0.070000
H18C	0.6093	0.2003	0.5105	0.070000
H20A	0.8206	0.2528	0.9082	0.056000
H20B	0.7815	0.4126	0.9504	0.056000
H20C	0.7923	0.4769	0.8757	0.056000
H21A	0.7237	0.0068	0.9386	0.073000
H21B	0.6395	0.0382	0.8986	0.073000
H21C	0.6757	0.1896	0.9618	0.073000
H23A	0.7986	0.2487	0.7734	0.070000
H23B	0.7392	0.4348	0.7622	0.070000
H23C	0.7294	0.2440	0.7082	0.070000
H24A	0.7684	-0.0971	0.8285	0.059000
H24B	0.7153	-0.1196	0.7530	0.059000
H24C	0.6831	-0.1504	0.8200	0.059000
H2B	0.1153	1.1970	0.6144	0.037000
H3B	0.0628	1.0379	0.6962	0.029000
H5B	0.0219	0.5163	0.5783	0.026000
H6B	0.0750	0.6721	0.4962	0.030000
H7B	-0.0204	0.5221	0.6806	0.022000
H8B	-0.0132	0.9171	0.7535	0.023000
H10B	-0.0875	1.1295	0.6643	0.034000
H11B	-0.1984	1.1967	0.5856	0.047000
H12B	-0.2913	0.9456	0.5629	0.045000
H13B	-0.2742	0.6257	0.6226	0.041000
H14B	-0.1634	0.5553	0.7002	0.031000
H16C	-0.0566	0.4066	0.8546	0.025000
H16D	-0.0441	0.3735	0.7762	0.025000
H18D	-0.0651	0.7408	1.0166	0.081000
H18E	-0.1176	0.8978	0.9642	0.081000
H18F	-0.0308	0.9400	0.9861	0.081000
H20D	0.1992	0.1644	0.8978	0.099000
H20E	0.1337	0.0640	0.8400	0.099000
H20F	0.1178	0.2591	0.8853	0.099000
H21D	0.2730	0.2429	0.8079	0.124000
H21E	0.2282	0.3381	0.7357	0.124000
H21F	0.2088	0.1082	0.7591	0.124000
H23D	0.1915	0.5489	0.9345	0.048000
H23E	0.2348	0.7447	0.9125	0.048000
H23F	0.2682	0.5124	0.9139	0.048000
H24D	0.2933	0.5696	0.8003	0.045000
H24E	0.2578	0.8002	0.7943	0.045000
H24F	0.2247	0.6247	0.7378	0.045000
H23G	0.1812	0.5200	0.9512	0.042000
H23H	0.0999	0.5307	0.9023	0.042000
H23I	0.1461	0.7425	0.9233	0.042000
H24G	0.2479	0.8064	0.8810	0.041000
H24H	0.2771	0.6943	0.8206	0.041000

H24I	0.2881	0.5827	0.8944	0.041000

Table S17. Hydrogen Bonds (Angstrom, Deg) for **3c**

**Donor – H..Aceptor [ARU]**	**D – H**	**H…A**	**D…A**	**D-H…A**
C3B -- H3B .. O2B [x,1+y,z]	0.9500	2.4100	3.213(8)	142.00
C8A -- H8A .. O3A	1.0000	2.4100	2.826(8)	104.00
C16A -- H16A .. O4A	0.9500	2.3500	2.697(9)	101.00
C16B -- H16C .. O4B	0.9500	2.3000	2.658(7)	101.00
C20B -- H20E .. O1B [x,-1+y,z]	0.9800	2.5000	3.437(9)	160.00

K. NMR Spectra

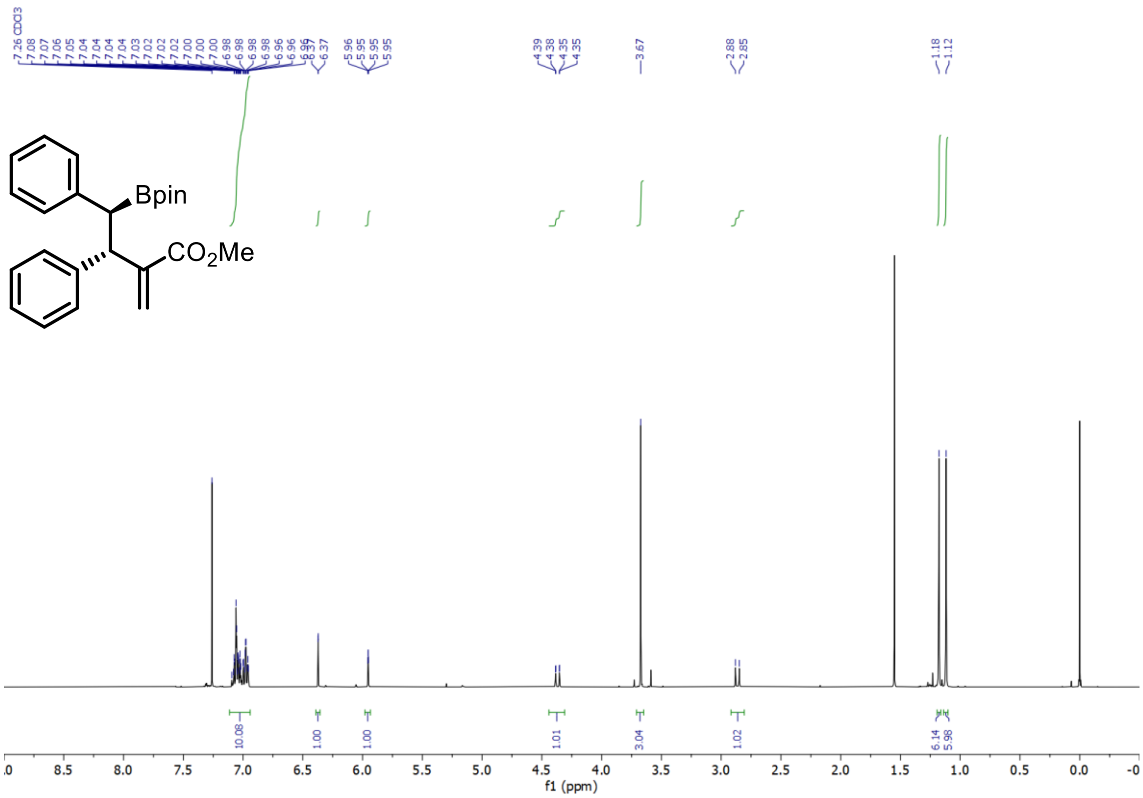

^{1}H NMR (400 MHz, $CDCl_3$) spectra of **3a**.

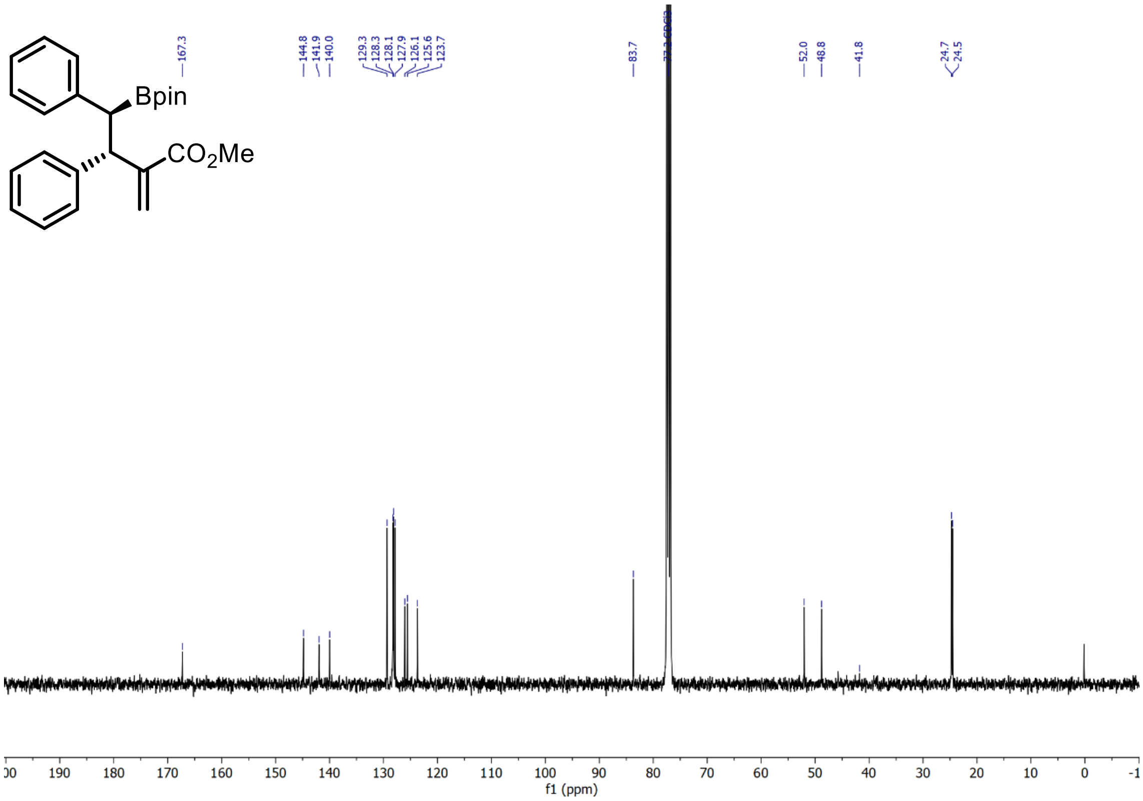

^{13}C NMR (101 MHz, $CDCl_3$) spectra of **3a**.

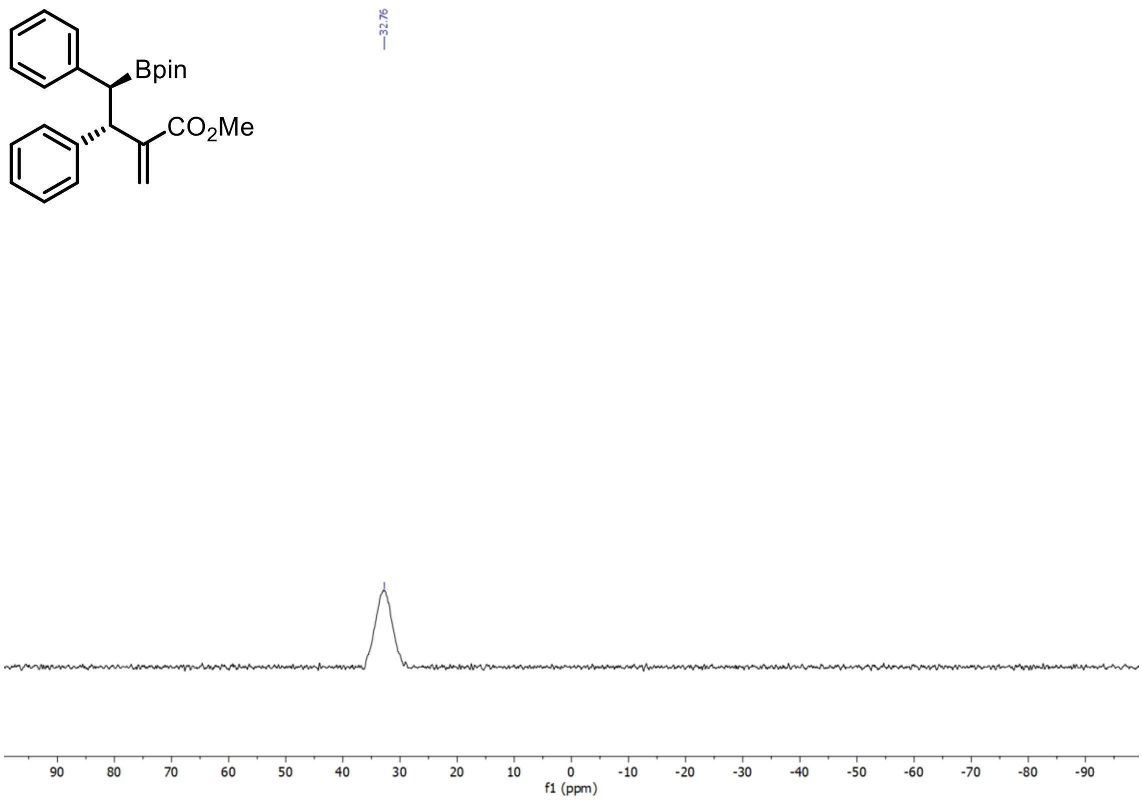

^{11}B NMR (128 MHz, $CDCl_3$) spectra of **3a**.

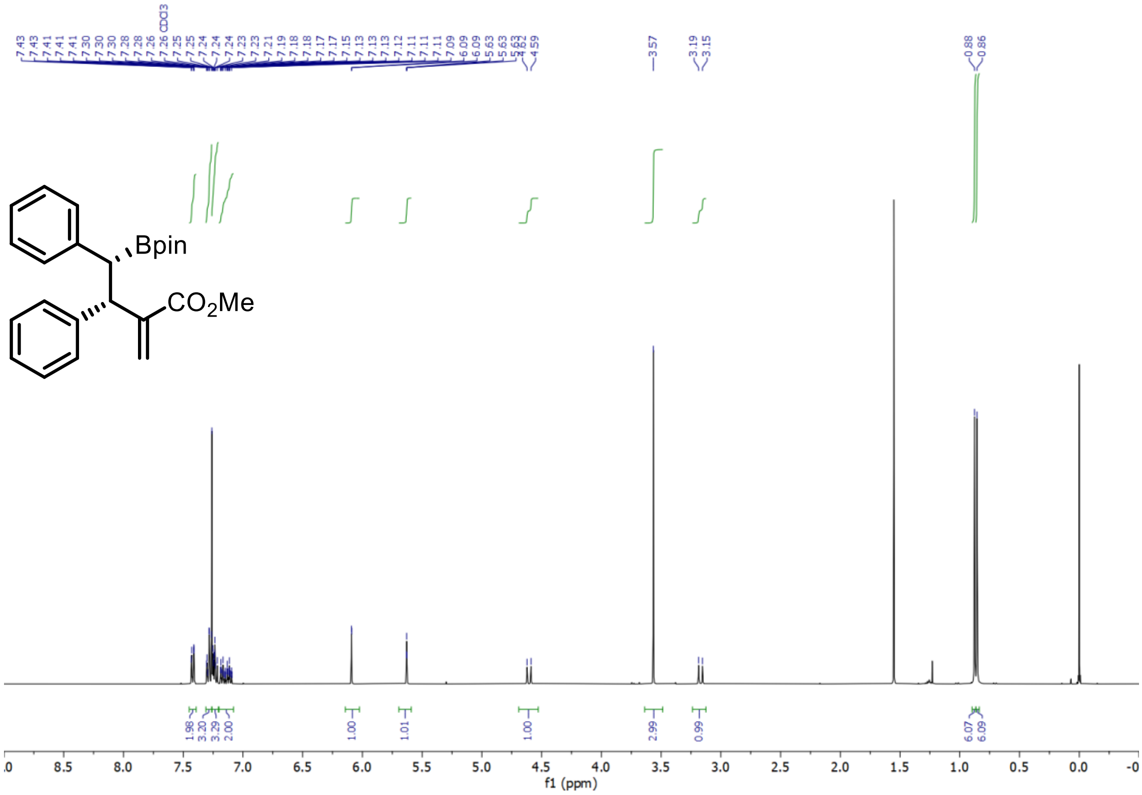

^{1}H NMR (400 MHz, $CDCl_3$) spectra of **3a'**.

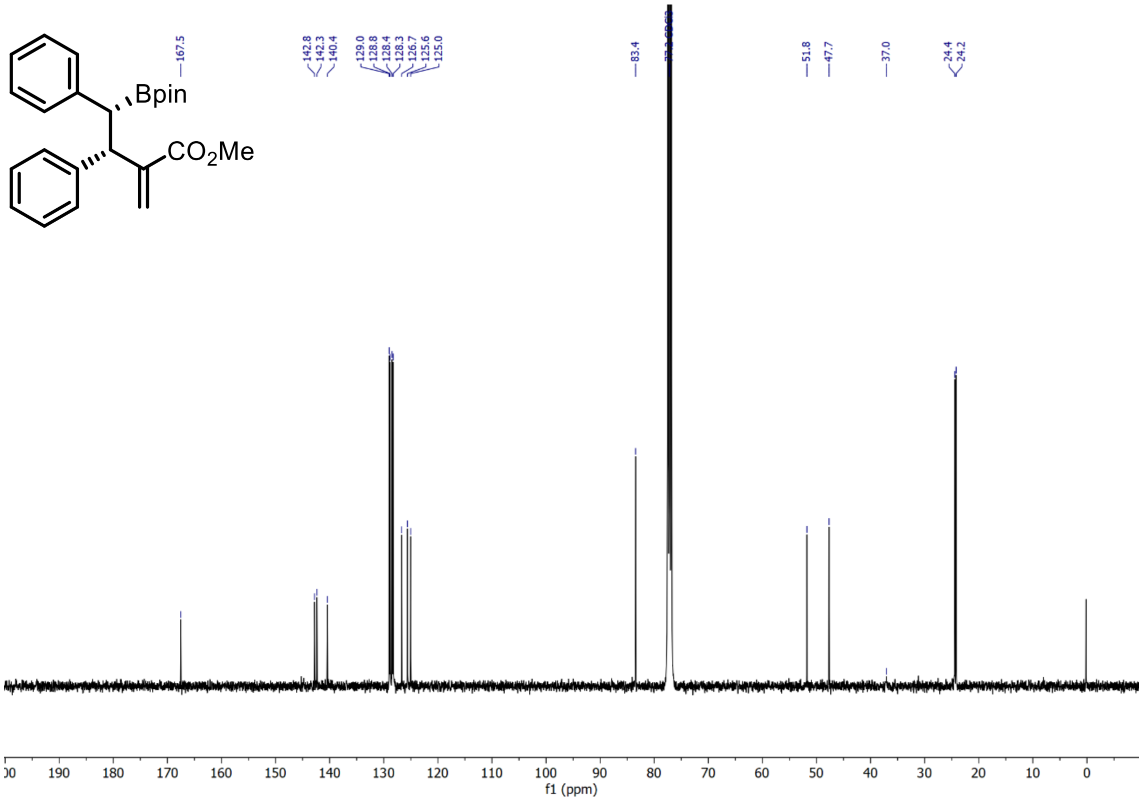

^{13}C NMR (101 MHz, $CDCl_3$) spectra of **3a'**.

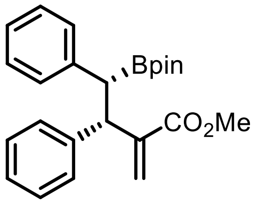

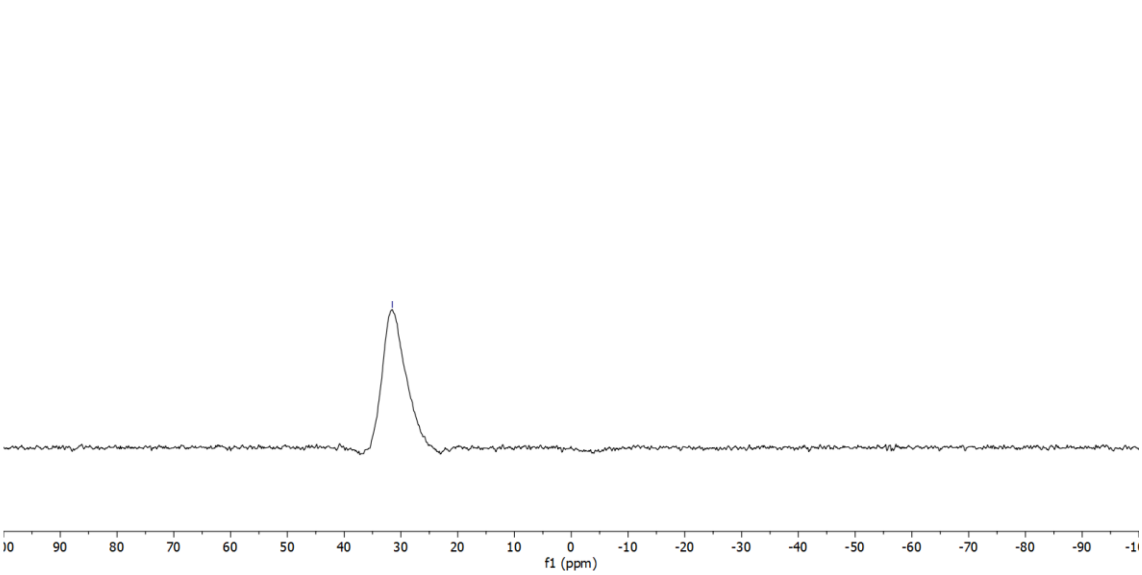

^{11}B NMR (128 MHz, $CDCl_3$) spectra of **3a’**.

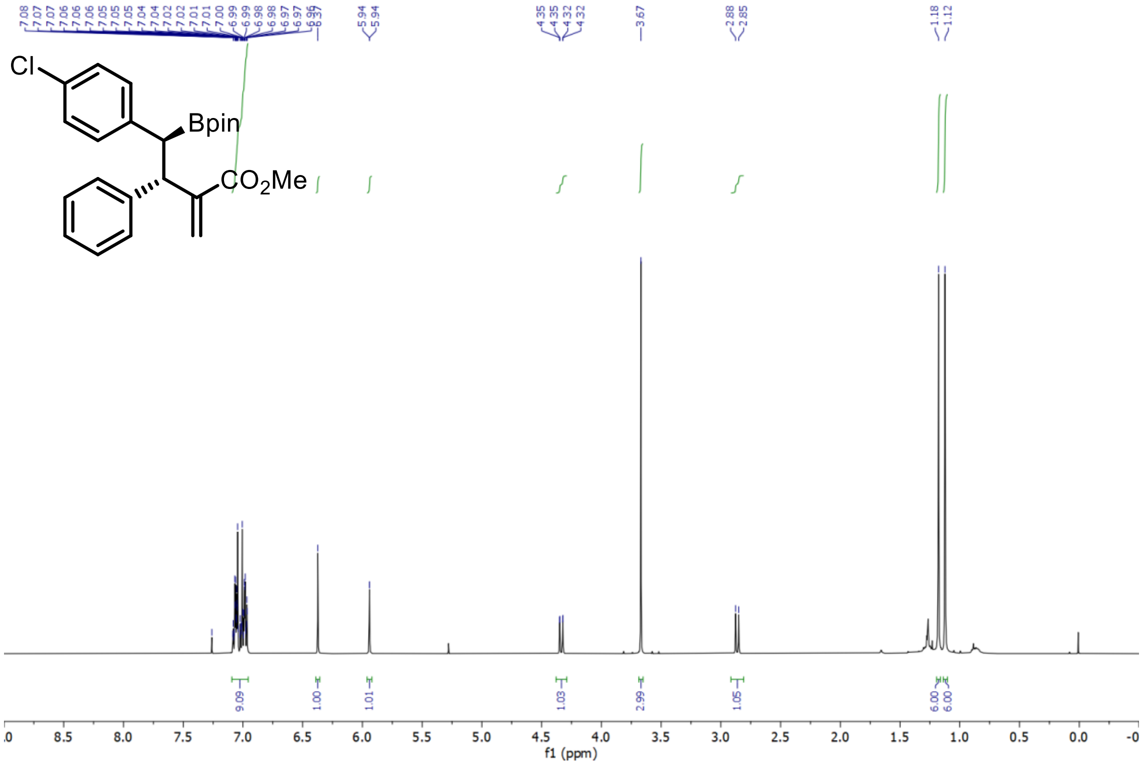

^{1}H NMR (500 MHz, $CDCl_3$) spectra of **3b**.

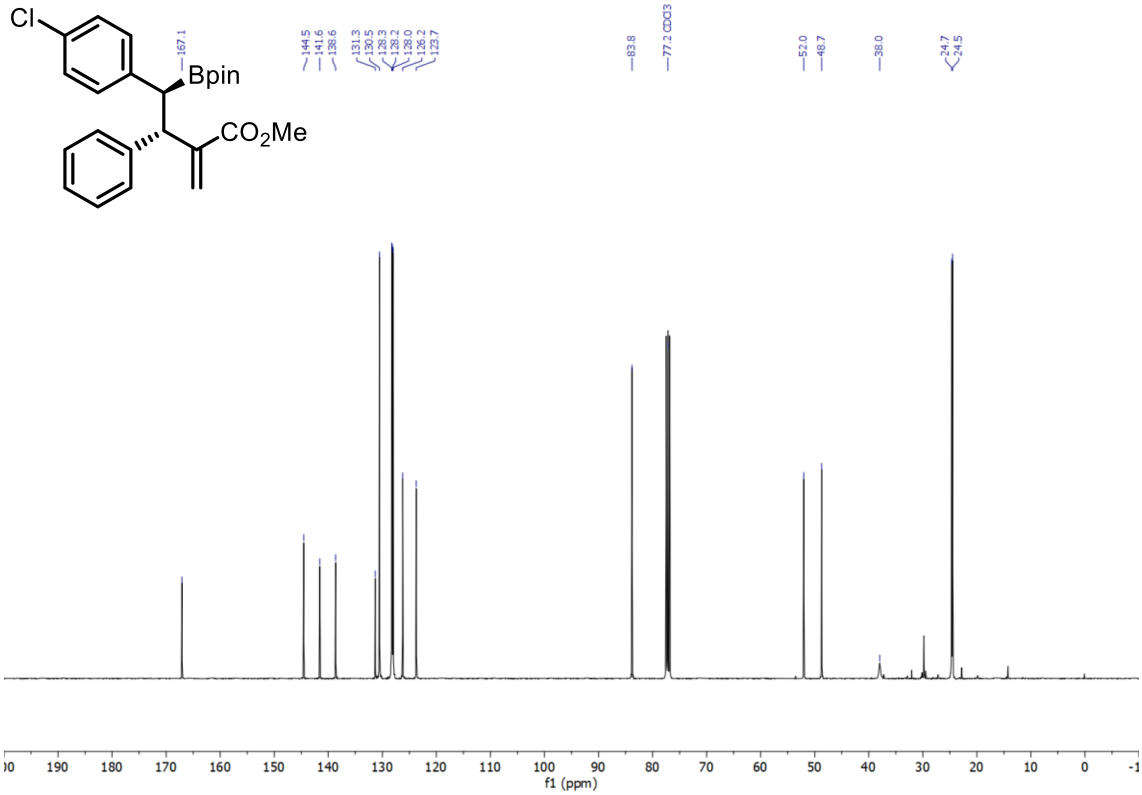

^{13}C NMR (101 MHz, $CDCl_3$) spectra of **3b**.

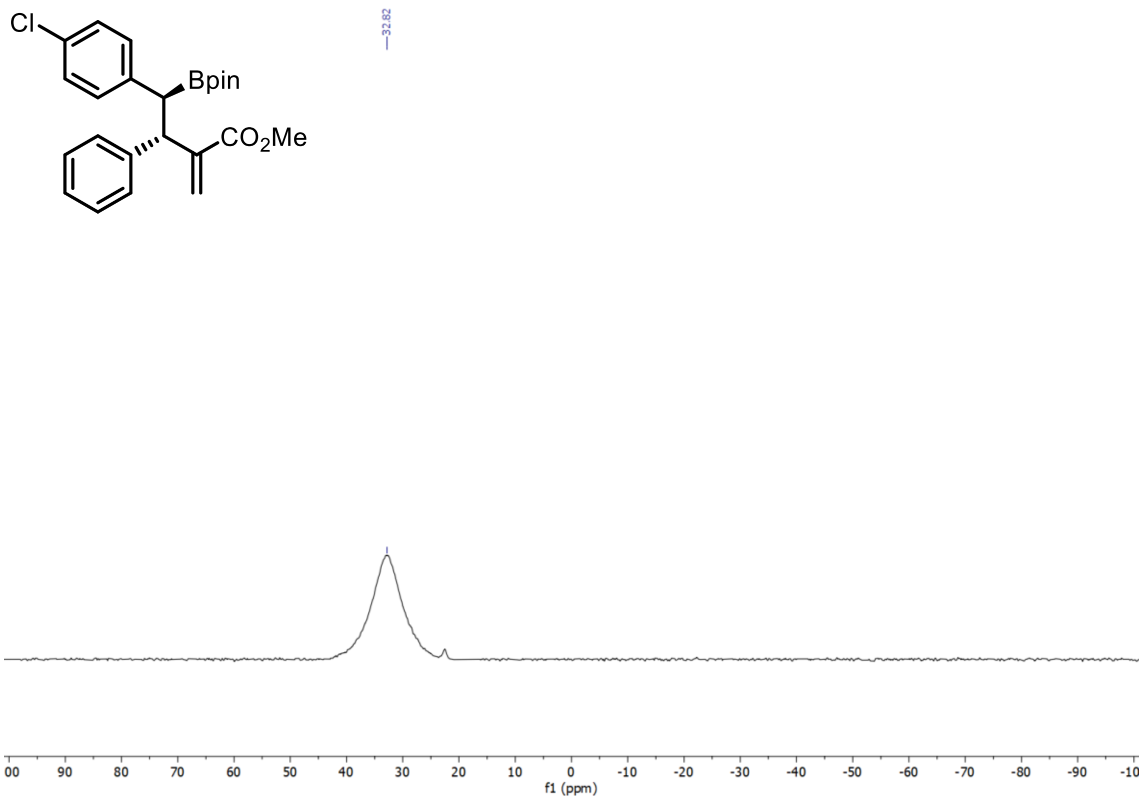

^{11}B NMR (128 MHz, $CDCl_3$) spectra of **3b**.

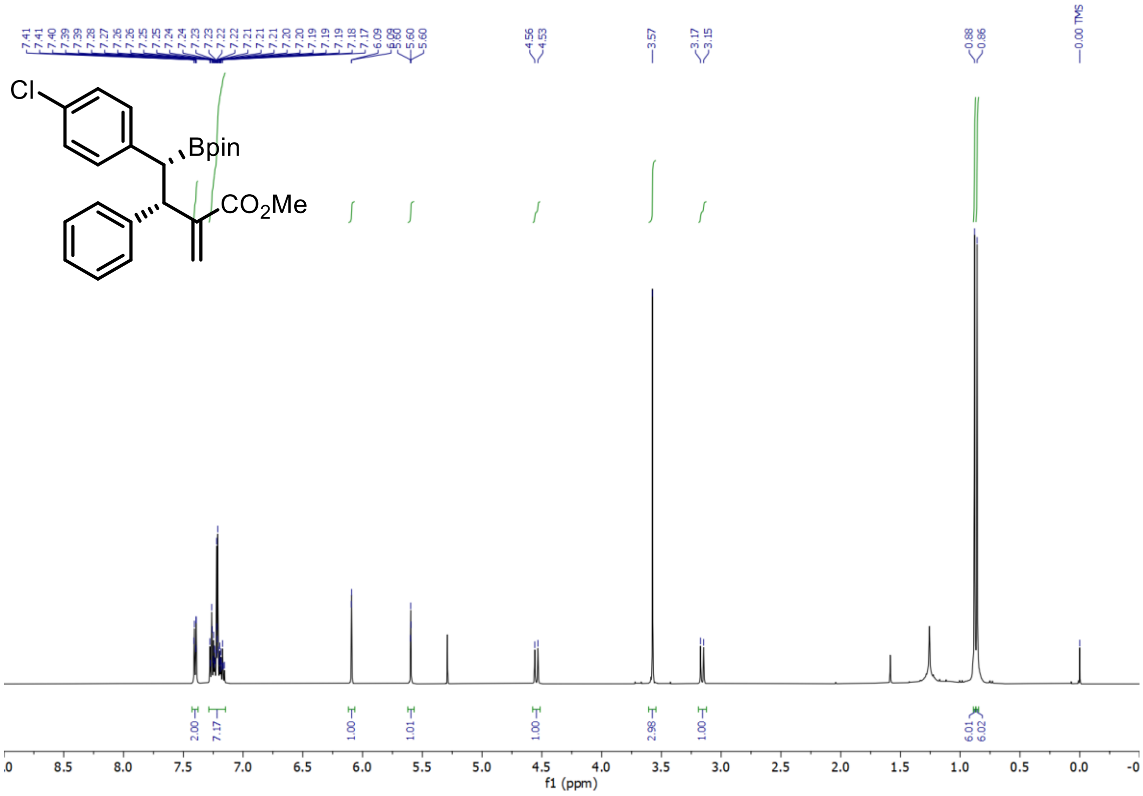

^{1}H NMR (500 MHz, $CDCl_3$) spectra of **3b'**.

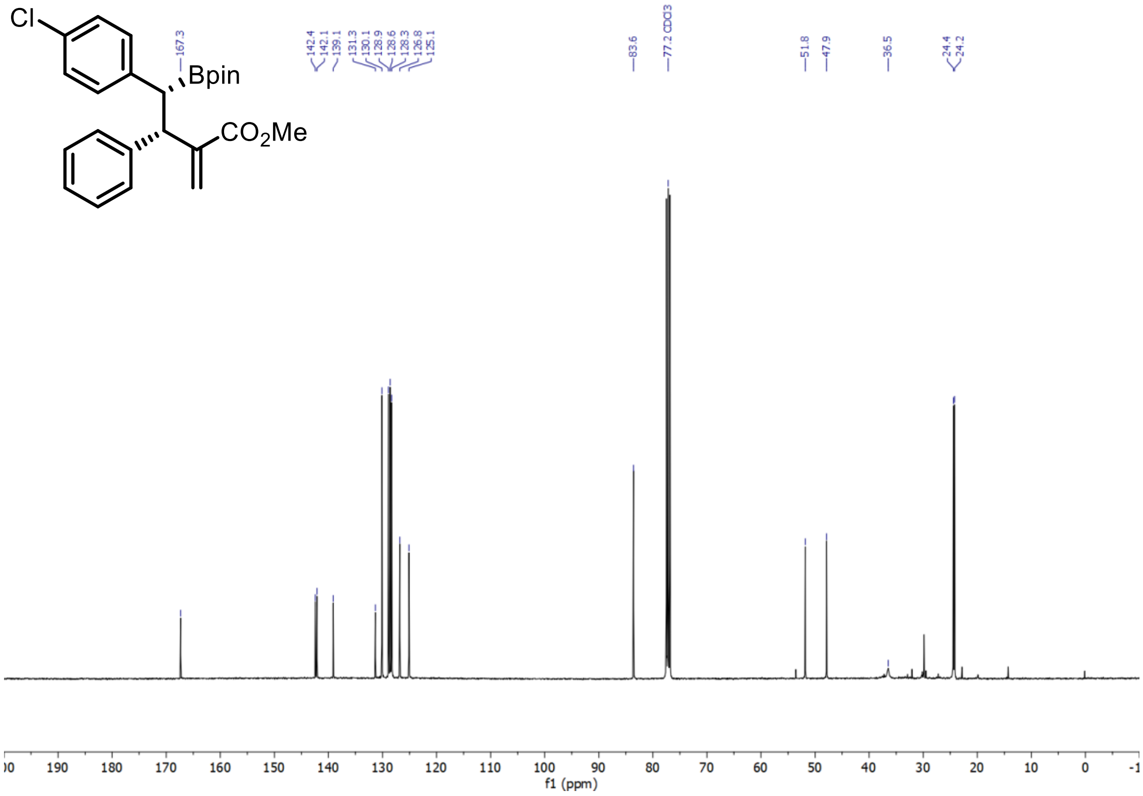

^{13}C NMR (101 MHz, $CDCl_3$) spectra of **3b'**.

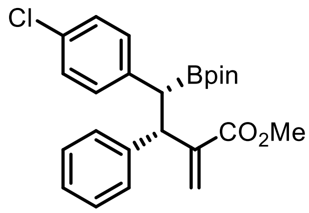

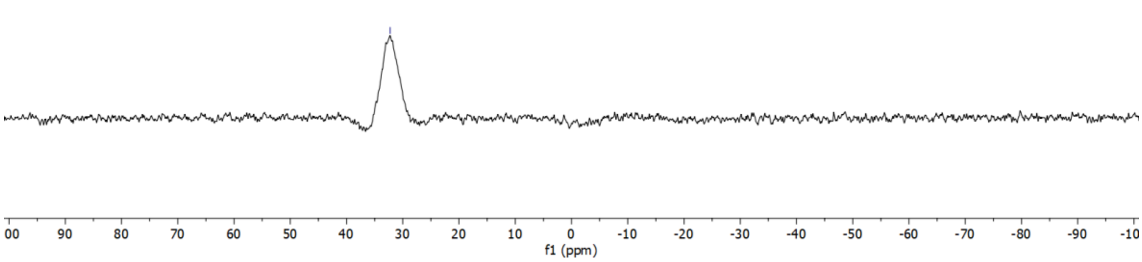

^{11}B NMR (128 MHz, $CDCl_3$) spectra of **3b’**.

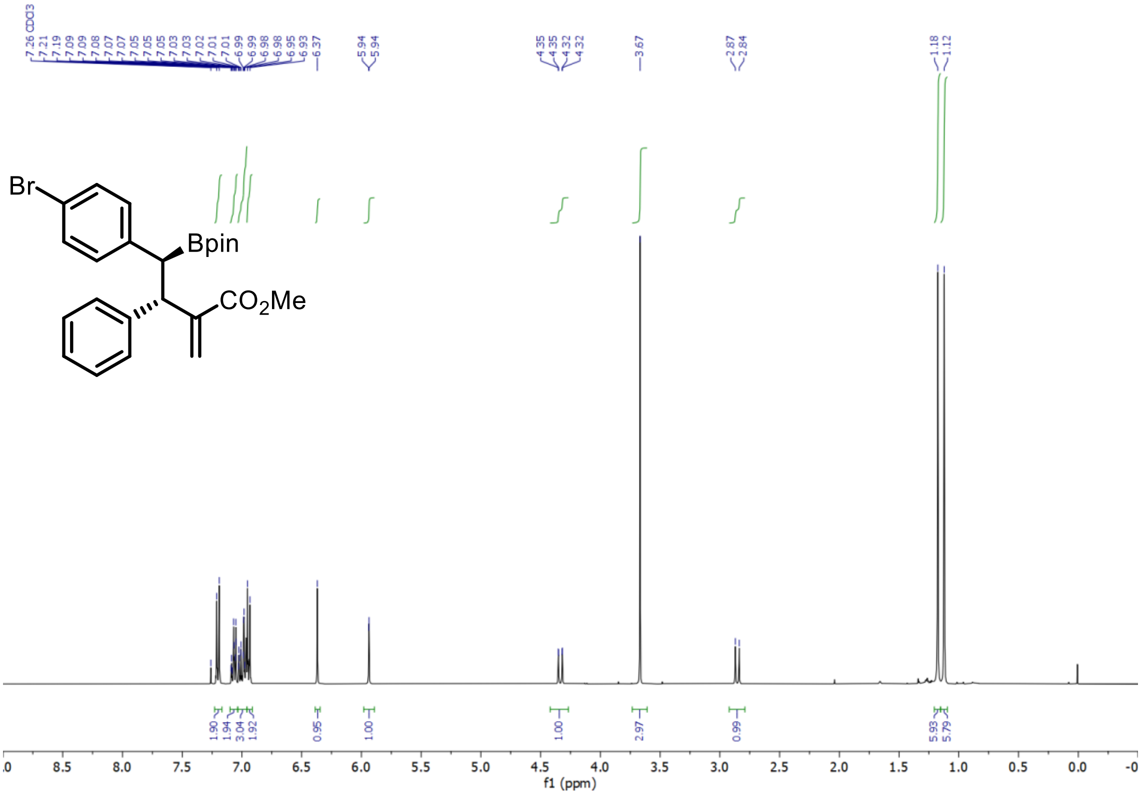

^{1}H NMR (400 MHz, $CDCl_3$) spectra of **3c**.

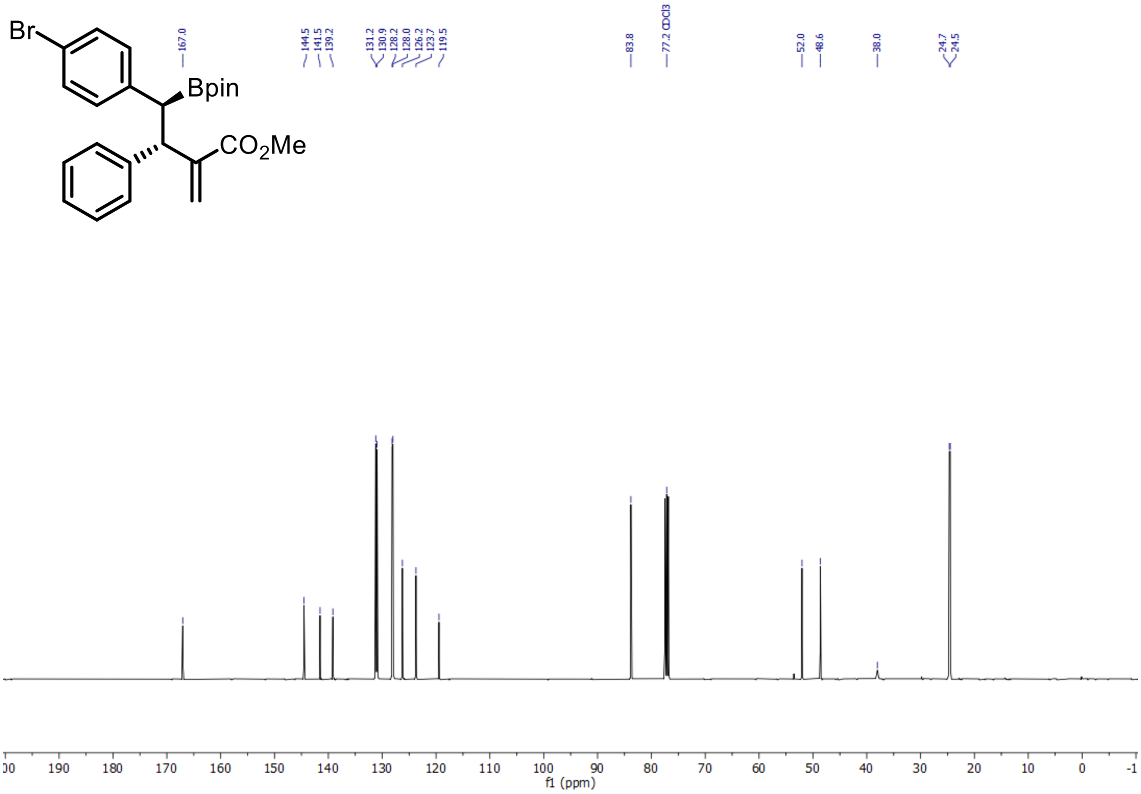

^{13}C NMR (101 MHz, $CDCl_3$) spectra of **3c**.

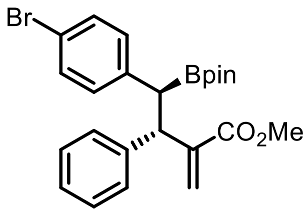

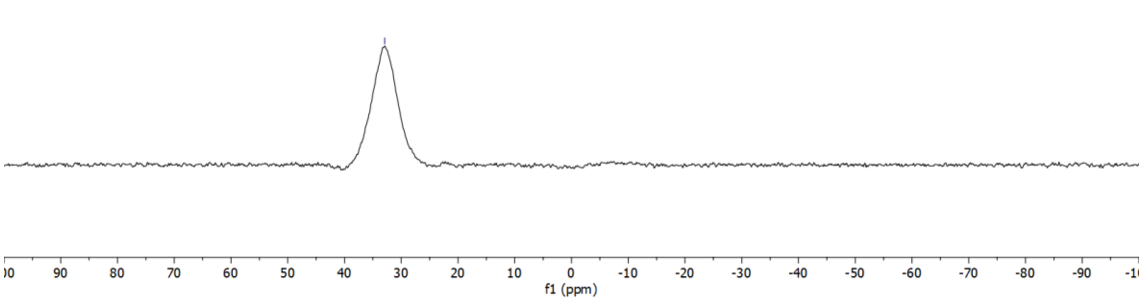

^{11}B NMR (128 MHz, $CDCl_3$) spectra of **3c**.

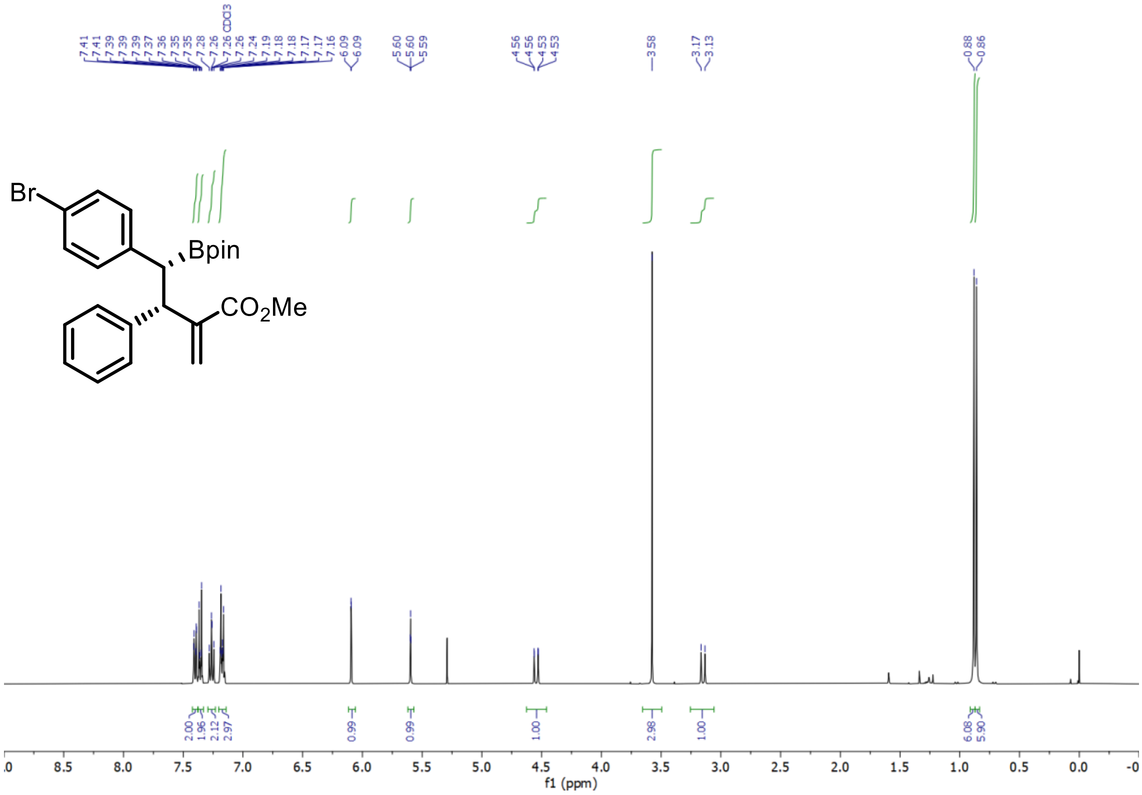

^{1}H NMR (400 MHz, $CDCl_3$) spectra of **3c’**.

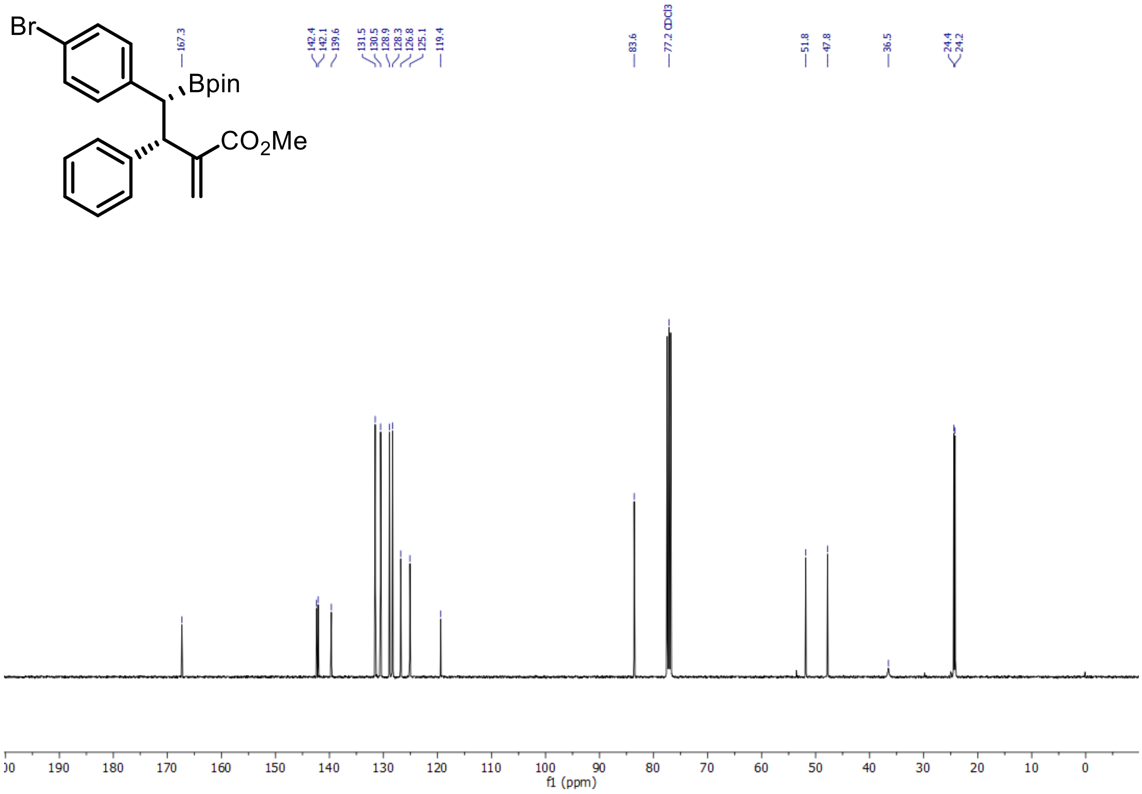

^{13}C NMR (101 MHz, $CDCl_3$) spectra of **3c’**.

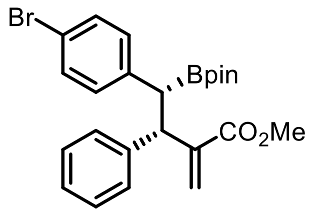

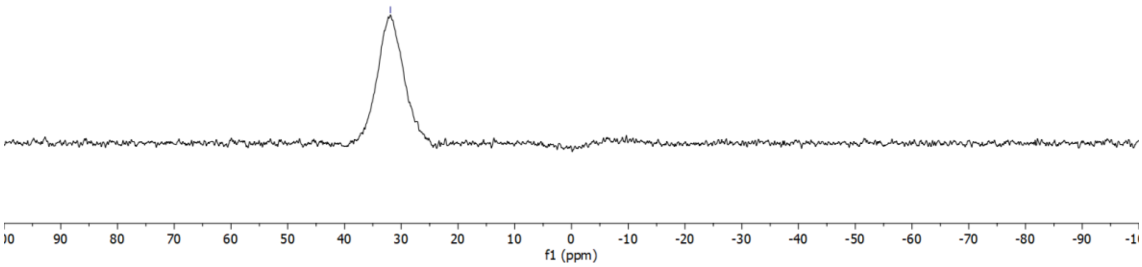

^{11}B NMR (128 MHz, $CDCl_3$) spectra of **3c'**.

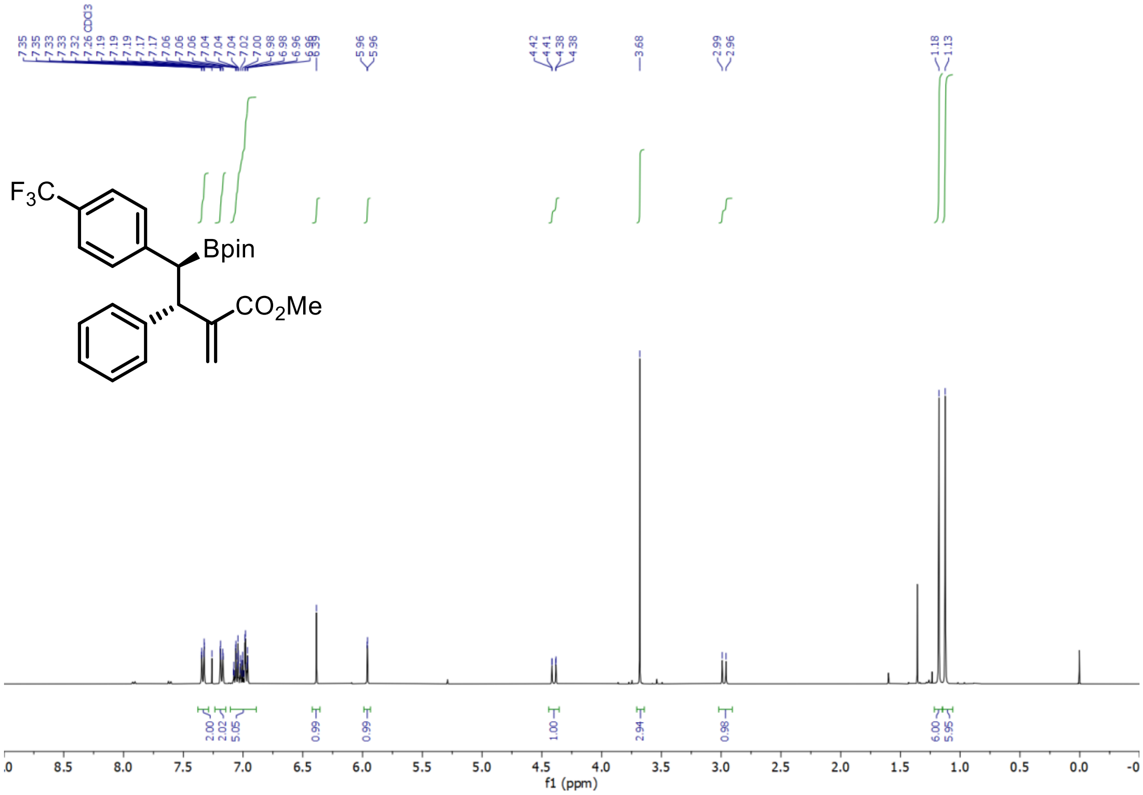

^{1}H NMR (400 MHz, $CDCl_3$) spectra of **3d**.

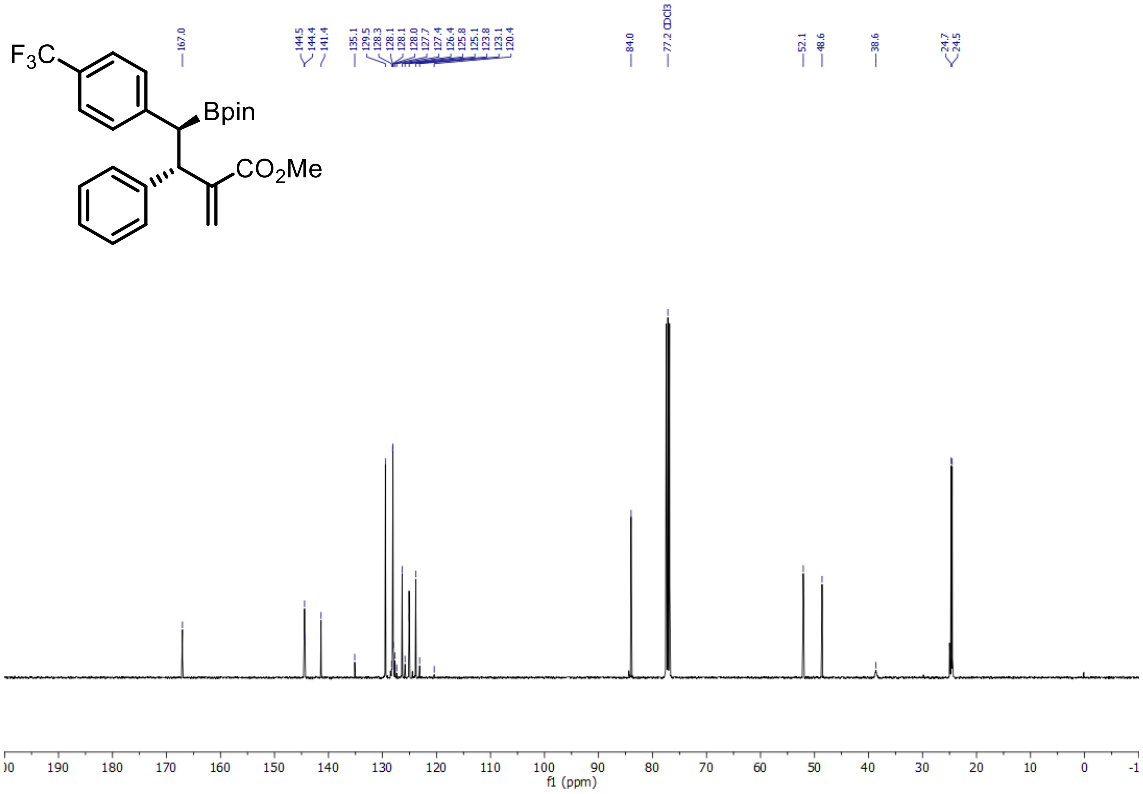

^{13}C NMR (101 MHz, $CDCl_3$) spectra of **3d**.

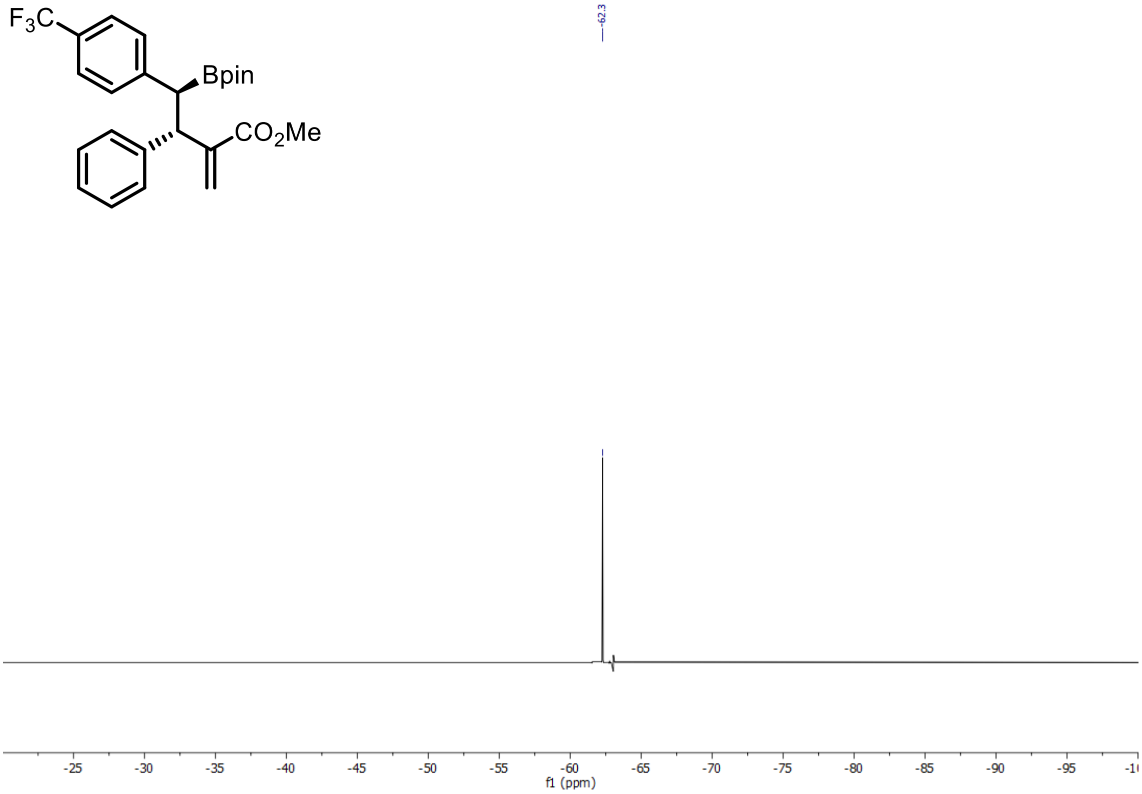

^{19}F NMR (376 MHz, $CDCl_3$) spectra of **3d**.

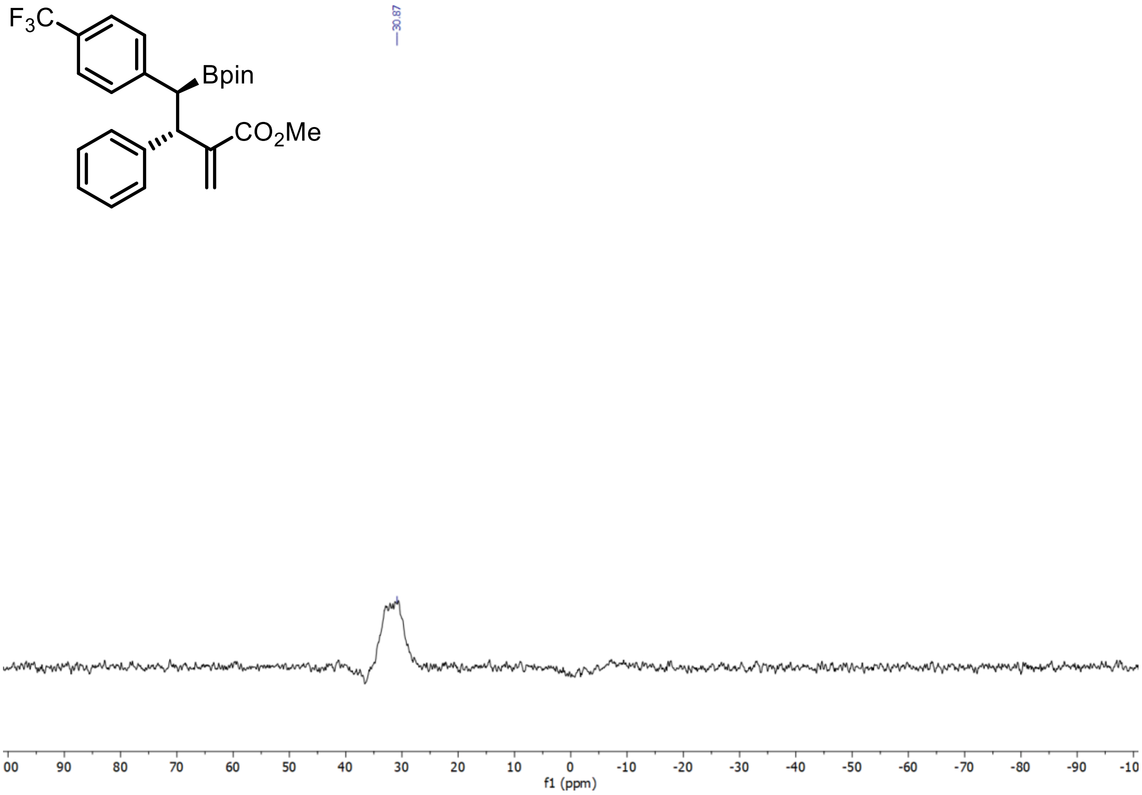

^{11}B NMR (128 MHz, $CDCl_3$) spectra of **3d**.

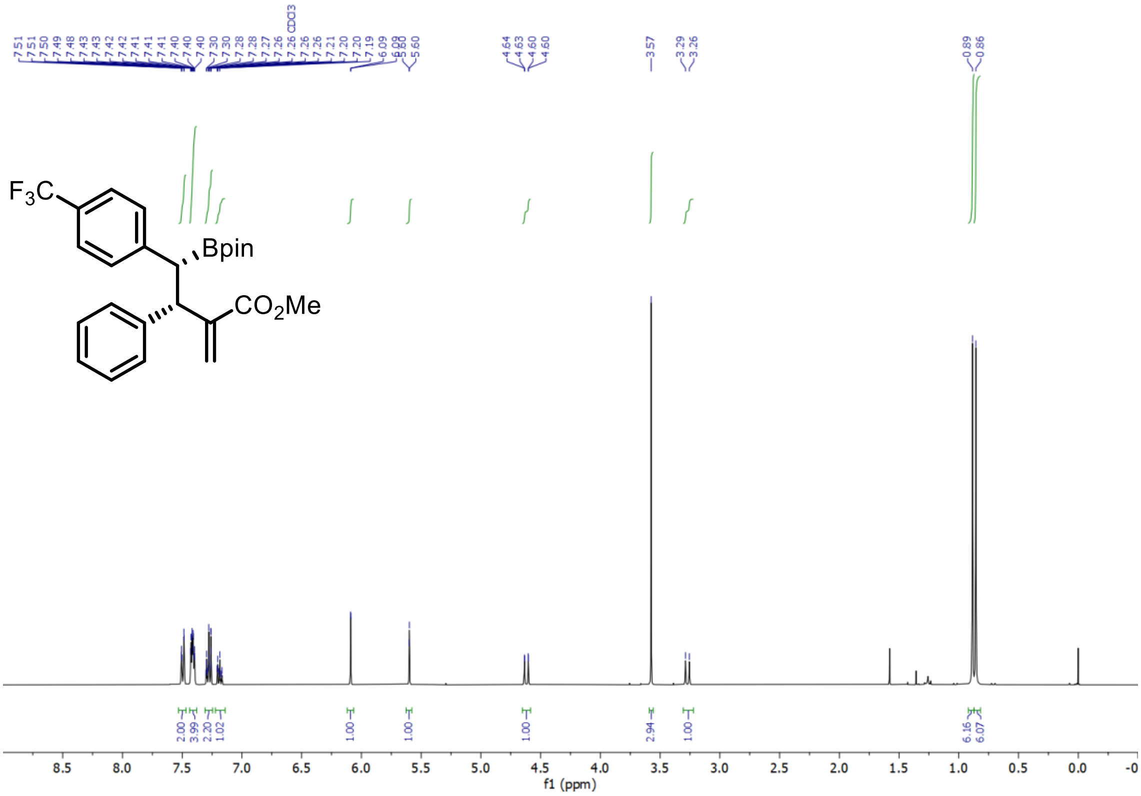

^{1}H NMR (400 MHz, $CDCl_3$) spectra of **3d’**.

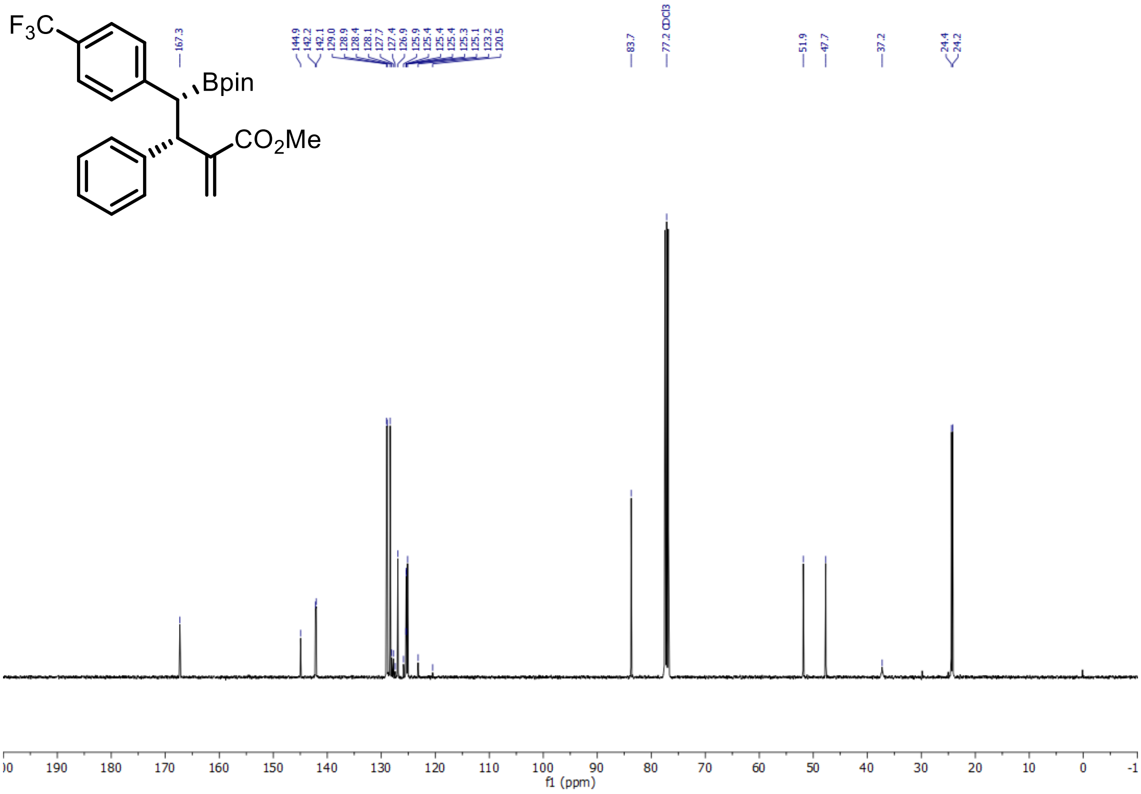

^{13}C NMR (101 MHz, $CDCl_3$) spectra of **3d’**.

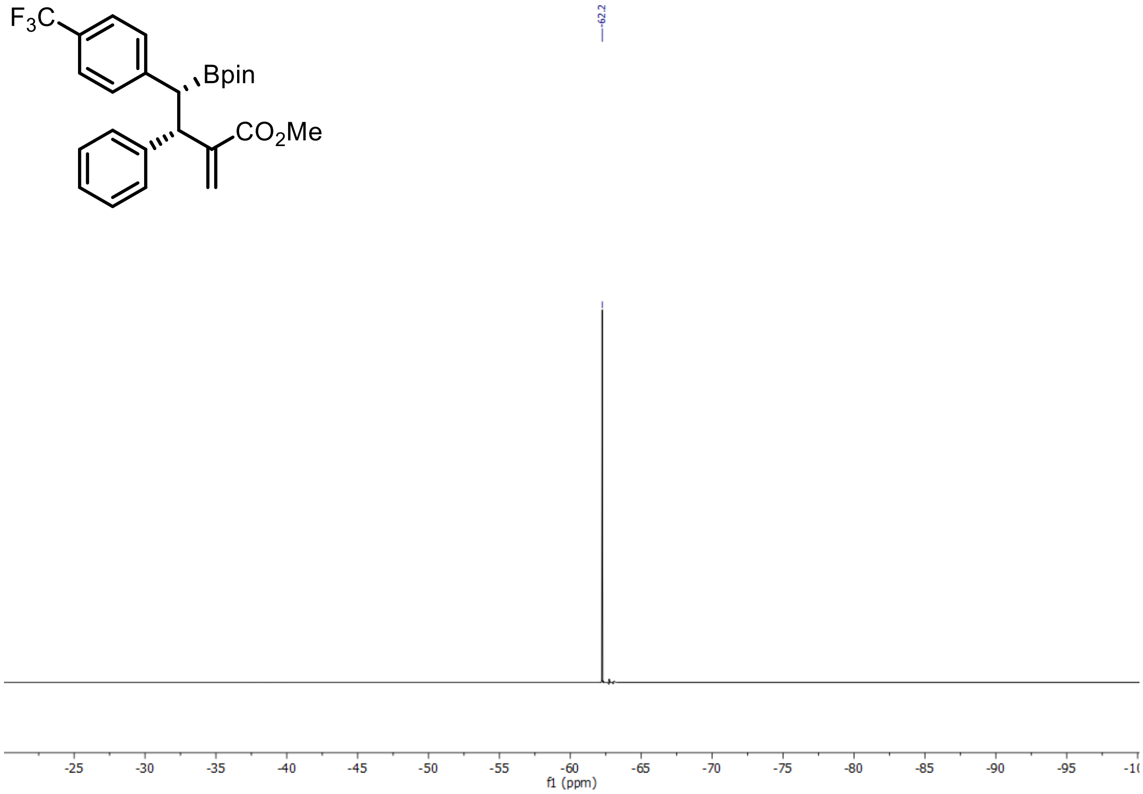

^{19}F NMR (376 MHz, $CDCl_3$) spectra of **3d'**.

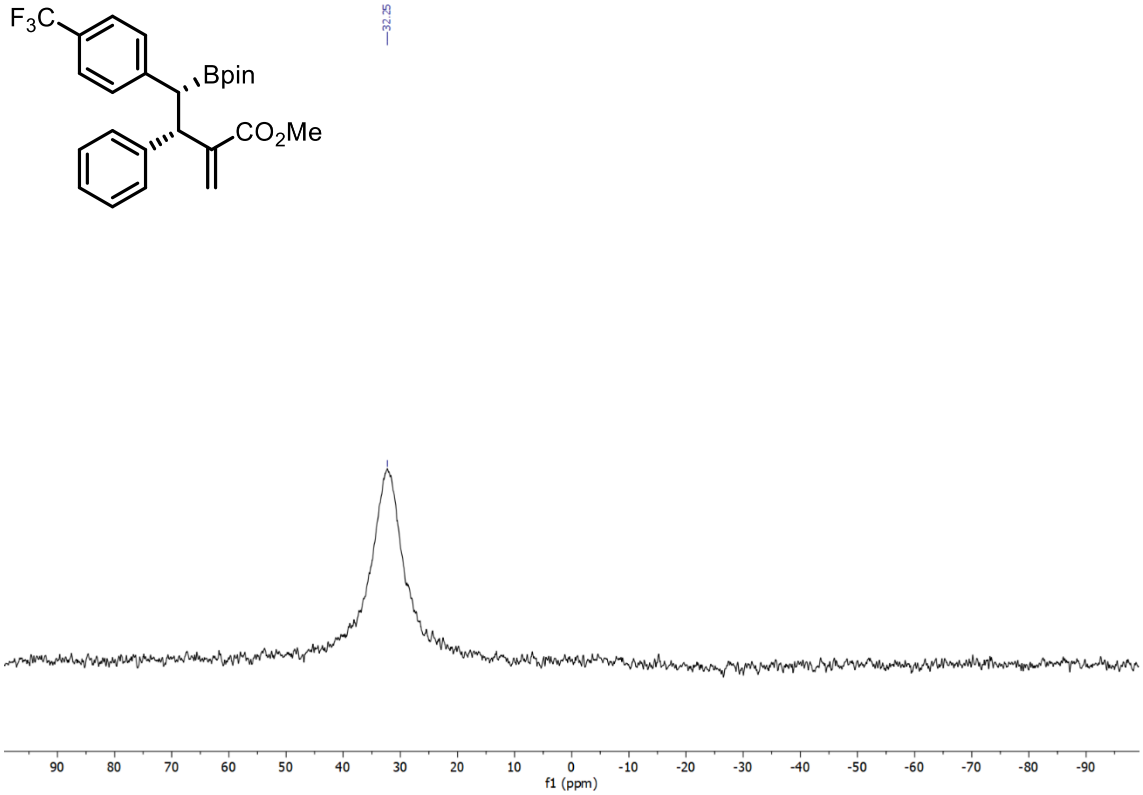

^{11}B NMR (128 MHz, $CDCl_3$) spectra of **3d'**.

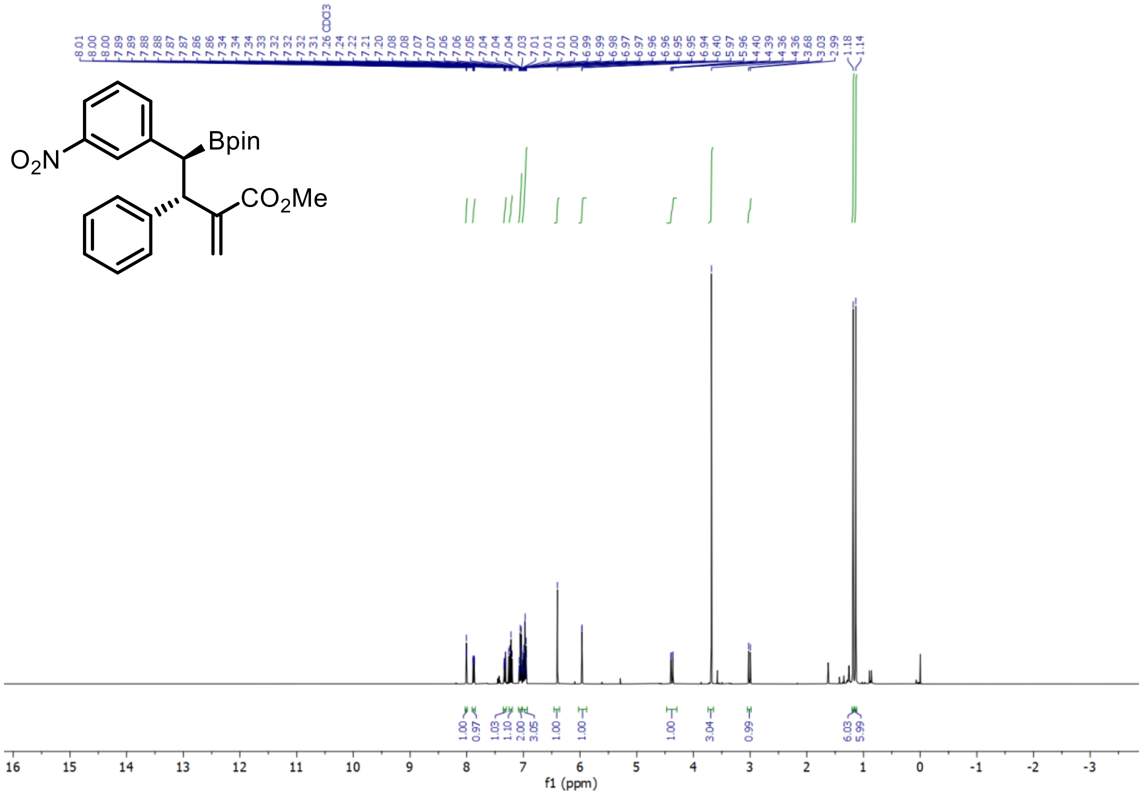

^{1}H NMR (400 MHz, $CDCl_3$) spectra of **3e**.

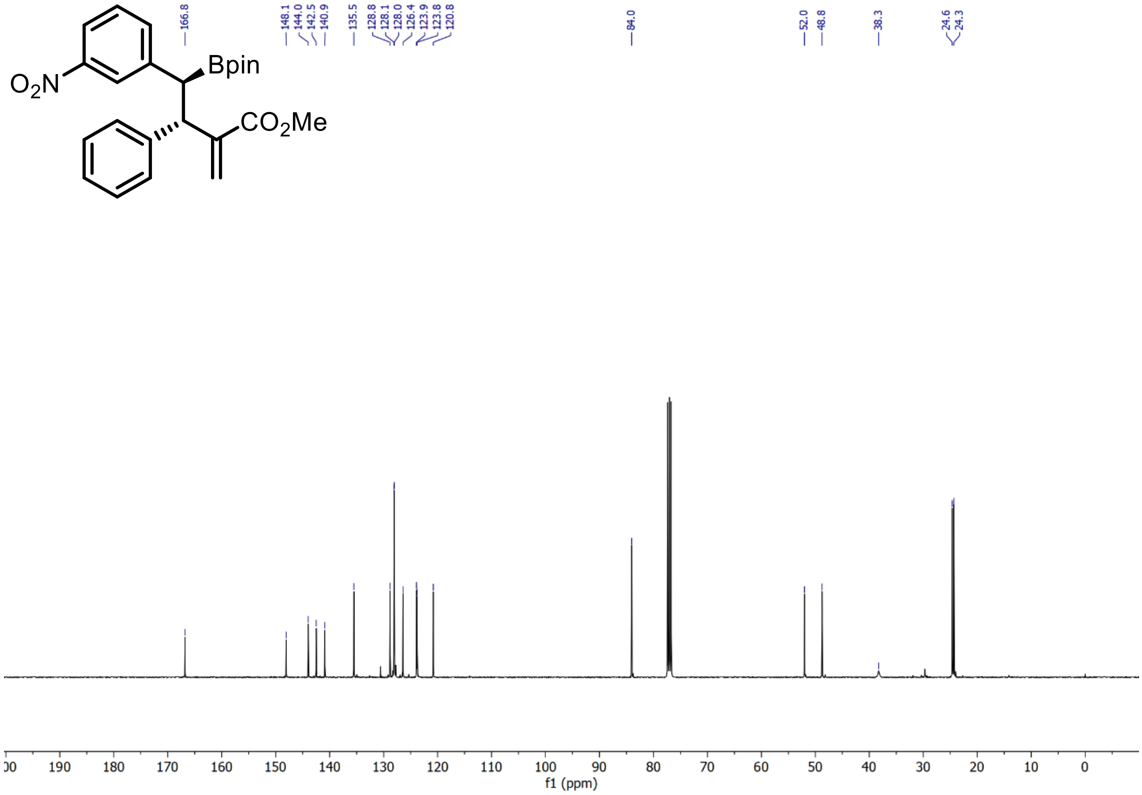

^{13}C NMR (101 MHz, $CDCl_3$) spectra of **3e.**

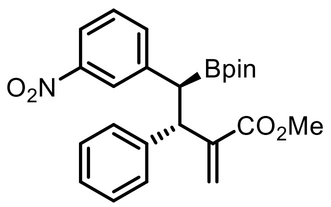

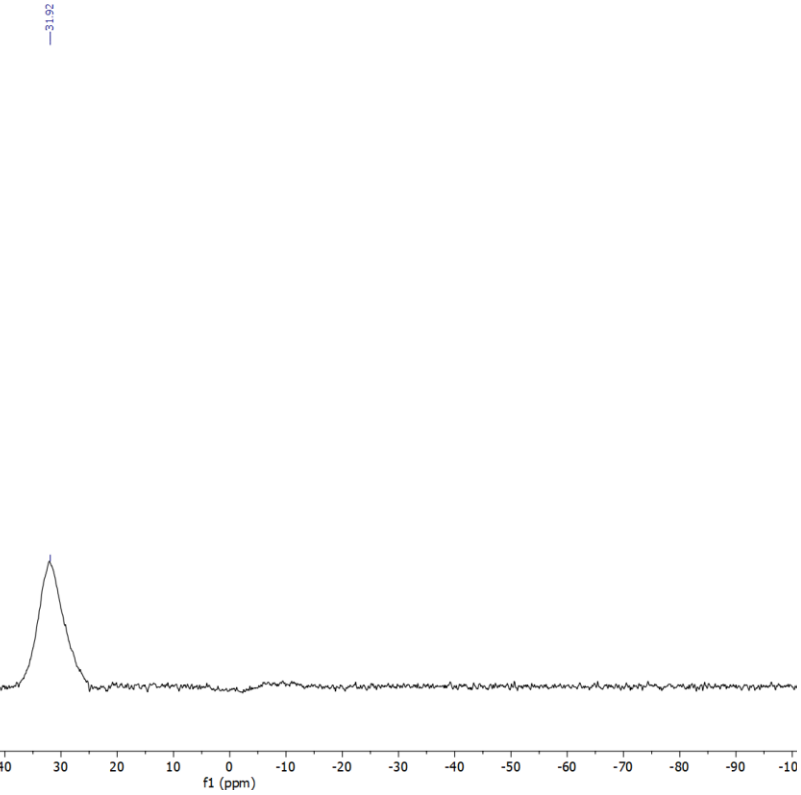

^{11}B NMR (128 MHz, $CDCl_3$) spectra of **3e.**

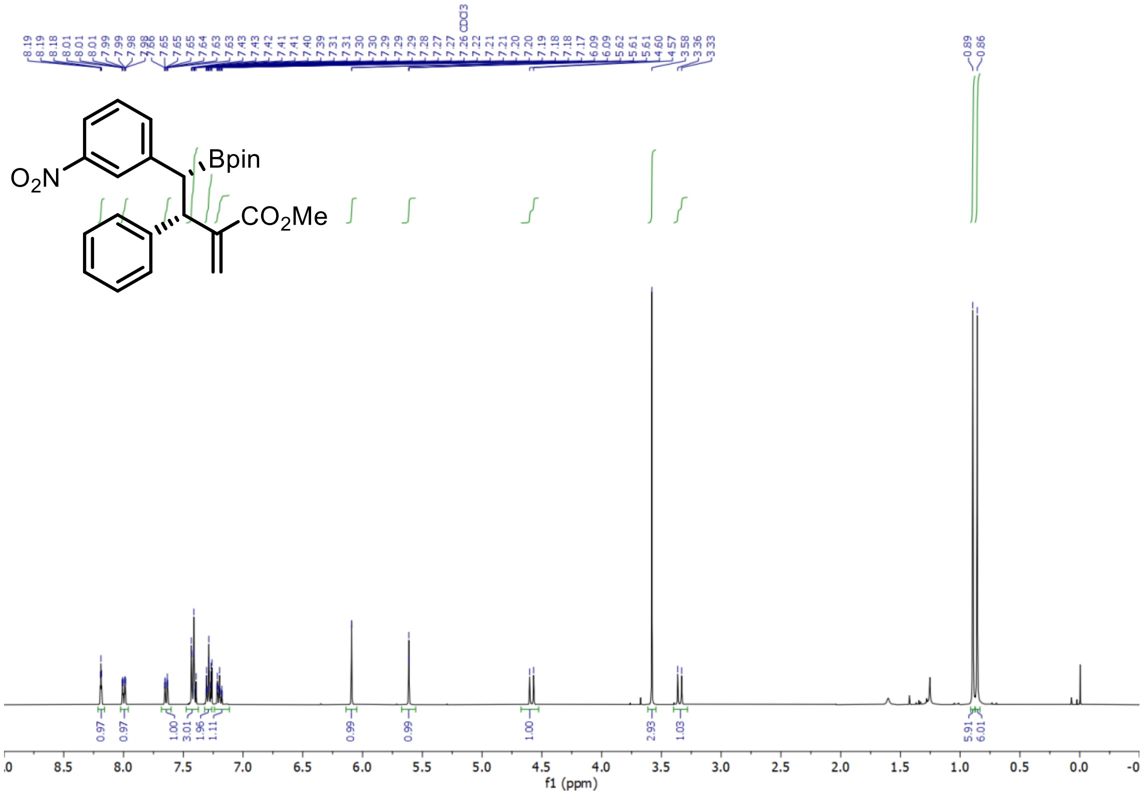

^{1}H NMR (400 MHz, $CDCl_3$) spectra of **3e’**.

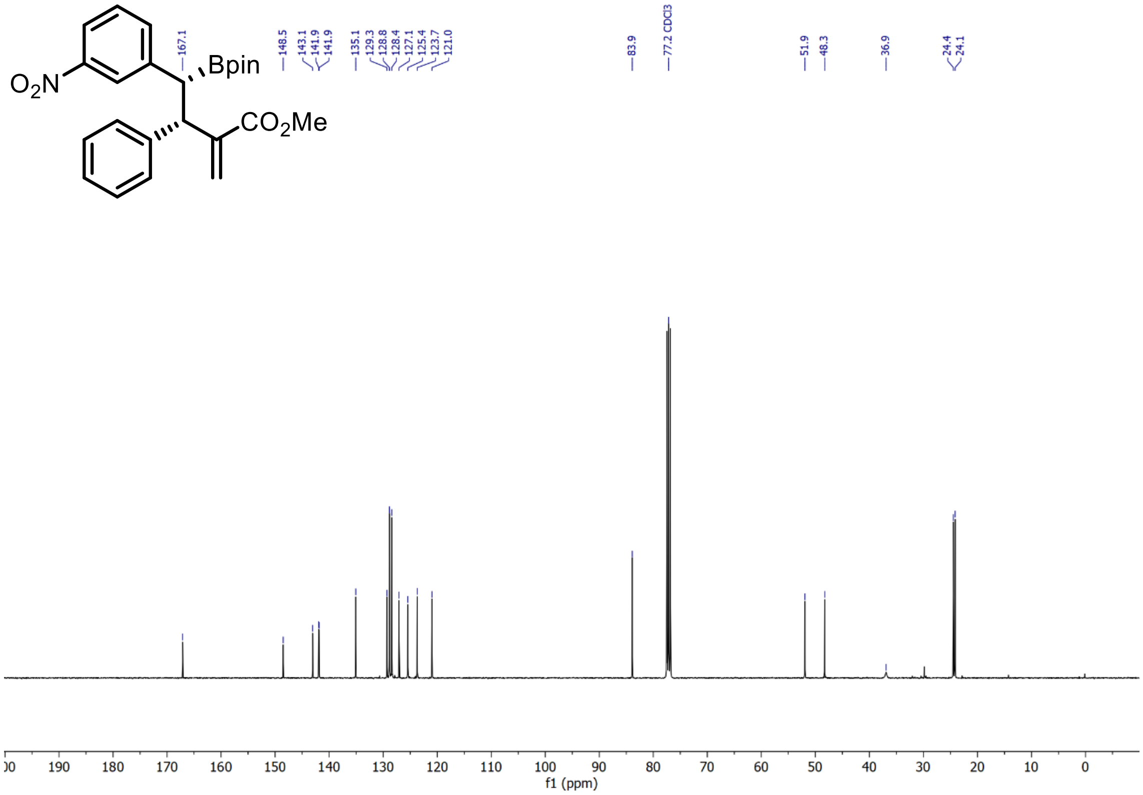

^{13}C NMR (101 MHz, $CDCl_3$) spectra of **3e’.**

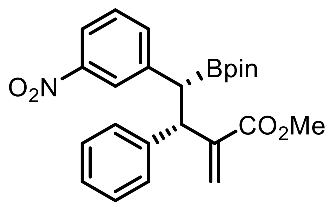

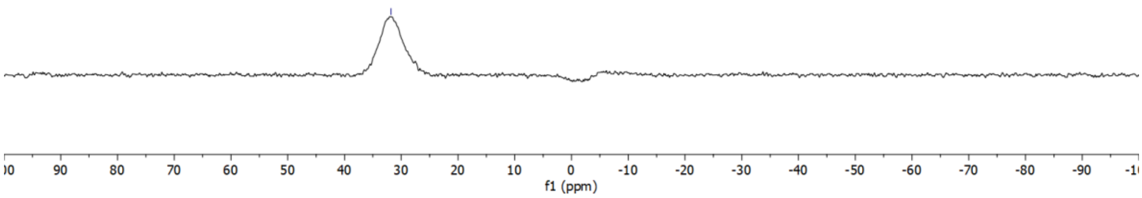

^{11}B NMR (128 MHz, $CDCl_3$) spectra of **3e'.**

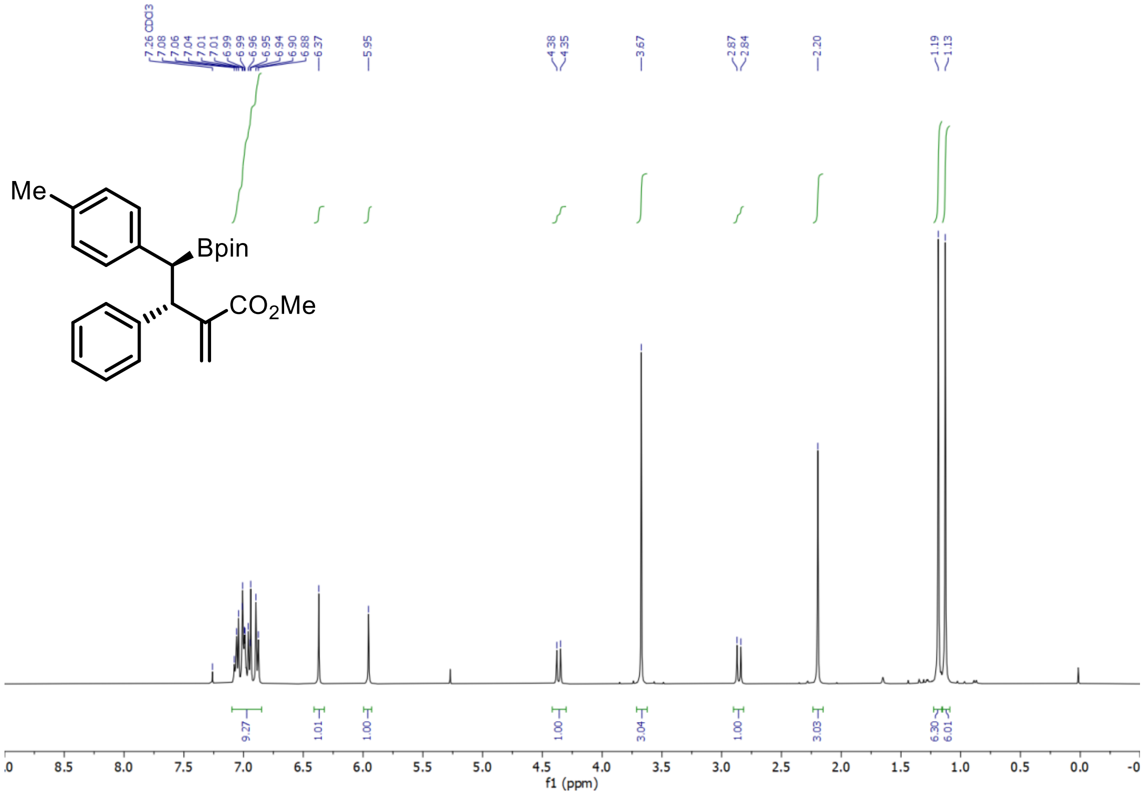

^{1}H NMR (400 MHz, $CDCl_3$) spectra of **3f**.

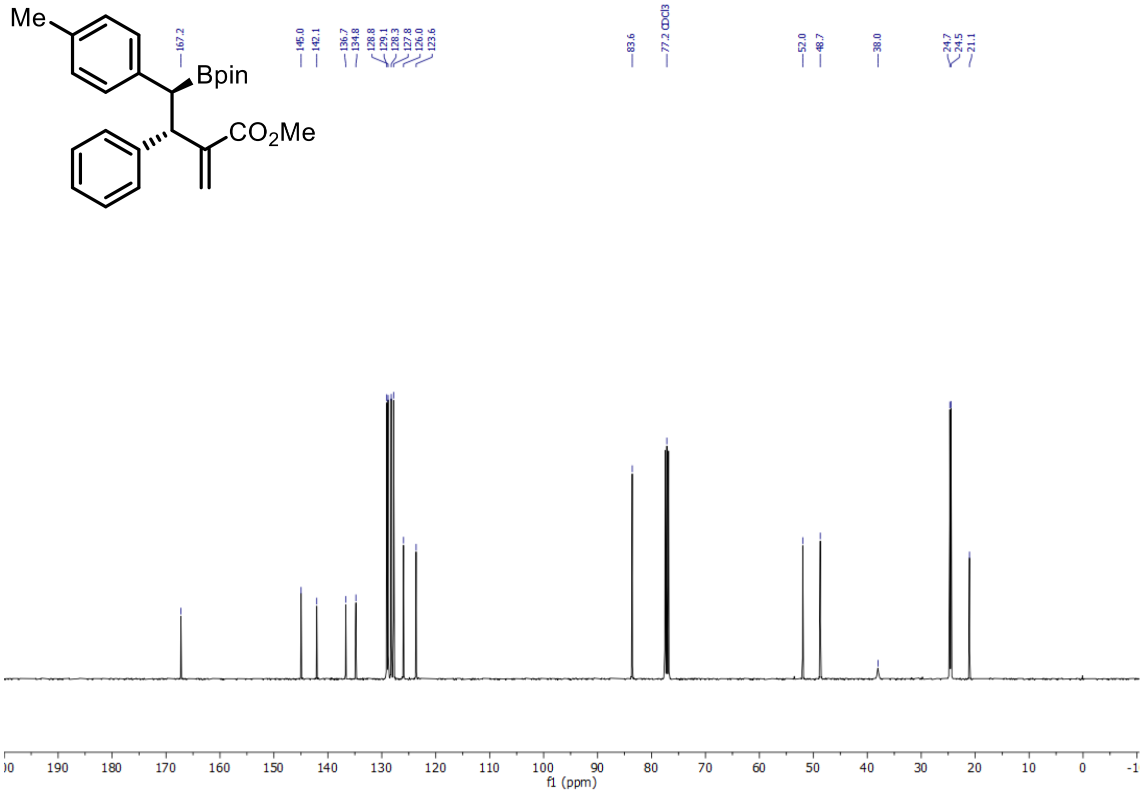

^{13}C NMR (101 MHz, $CDCl_3$) spectra of **3f.**

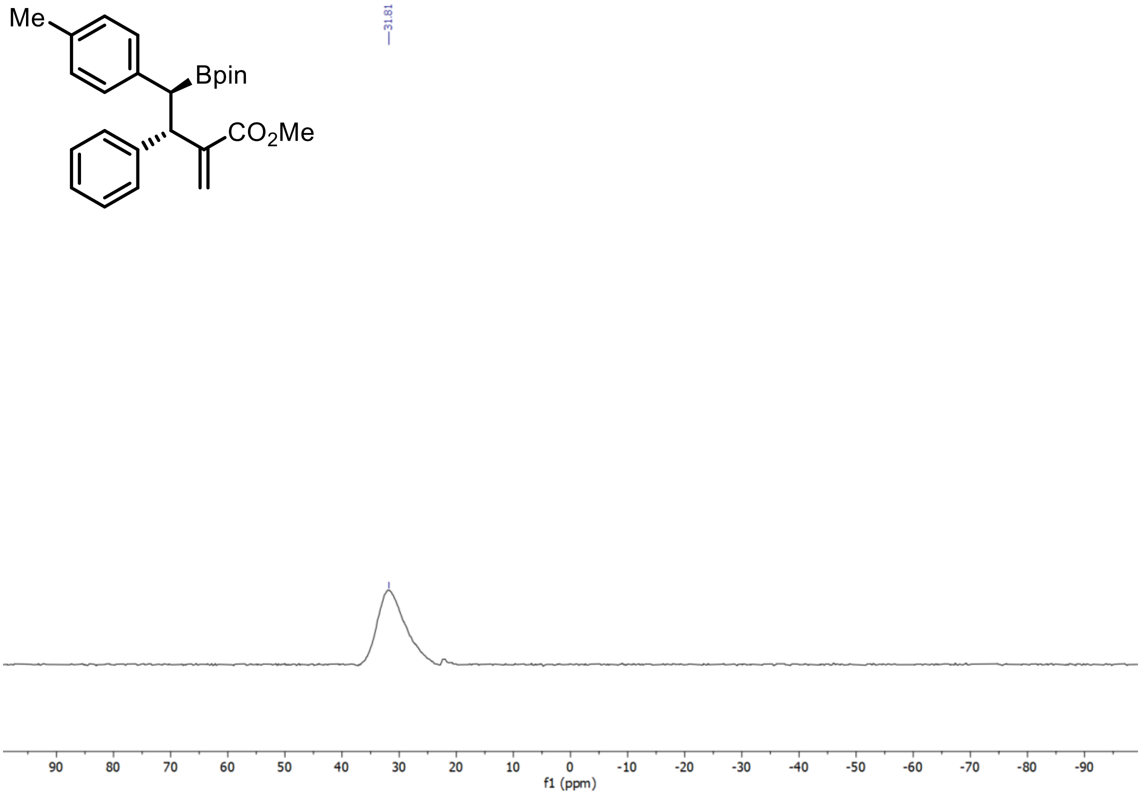

^{11}B NMR (128 MHz, $CDCl_3$) spectra of **3f.**

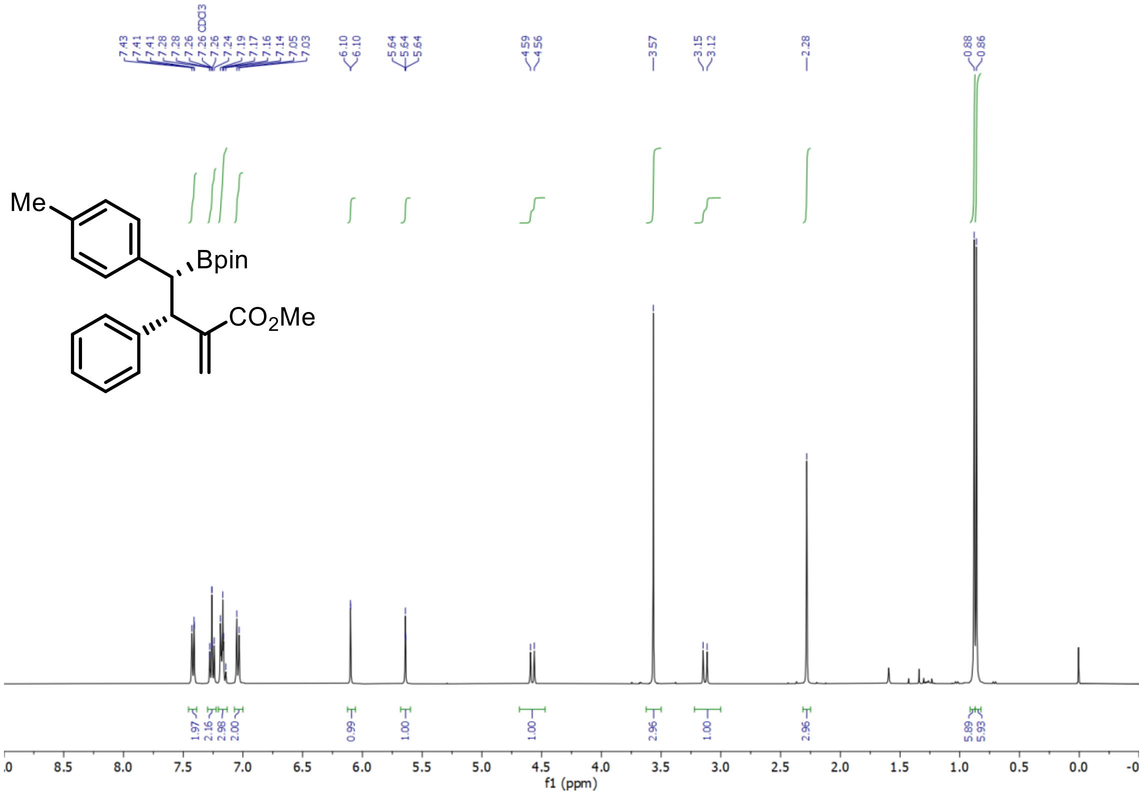

^{1}H NMR (400 MHz, $CDCl_3$) spectra of **3f'**.

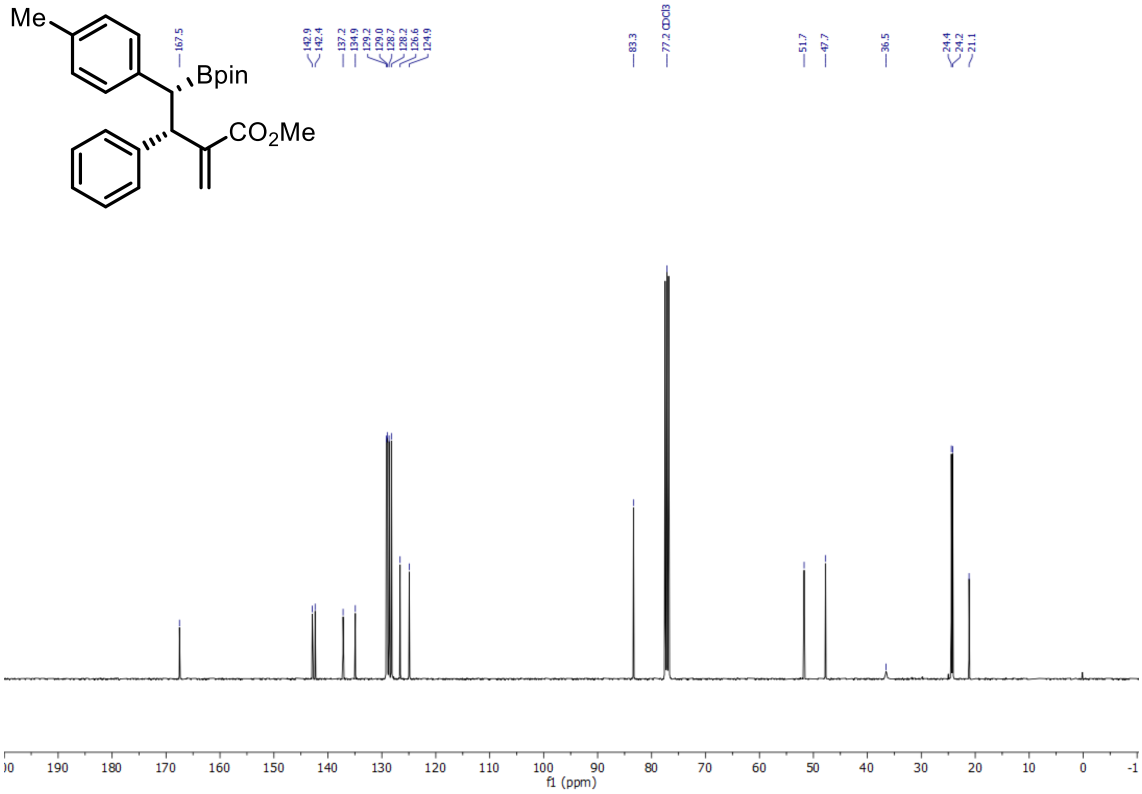

^{13}C NMR (101 MHz, $CDCl_3$) spectra of **3f'**.

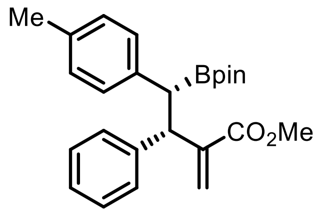

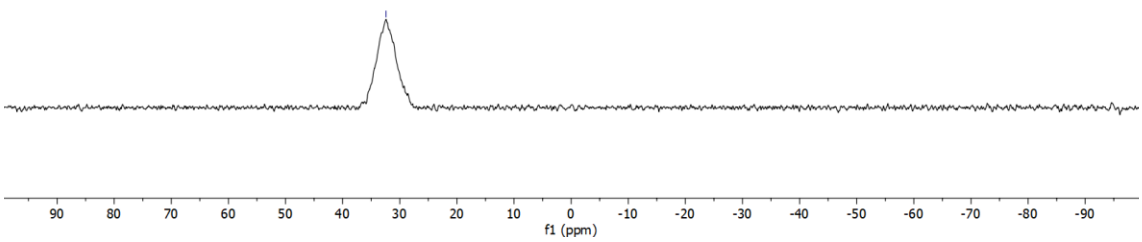

^{11}B NMR (128 MHz, $CDCl_3$) spectra of **3f'.**

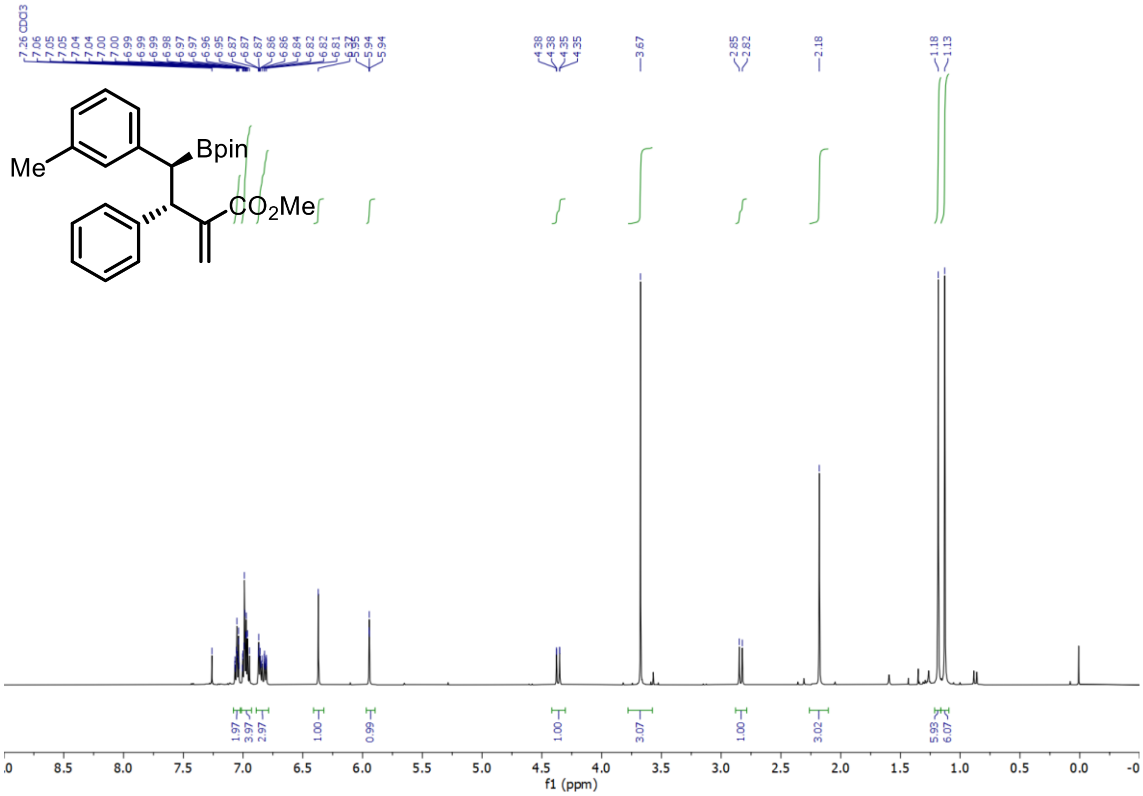

^{1}H NMR (500 MHz, $CDCl_3$) spectra of **3g**.

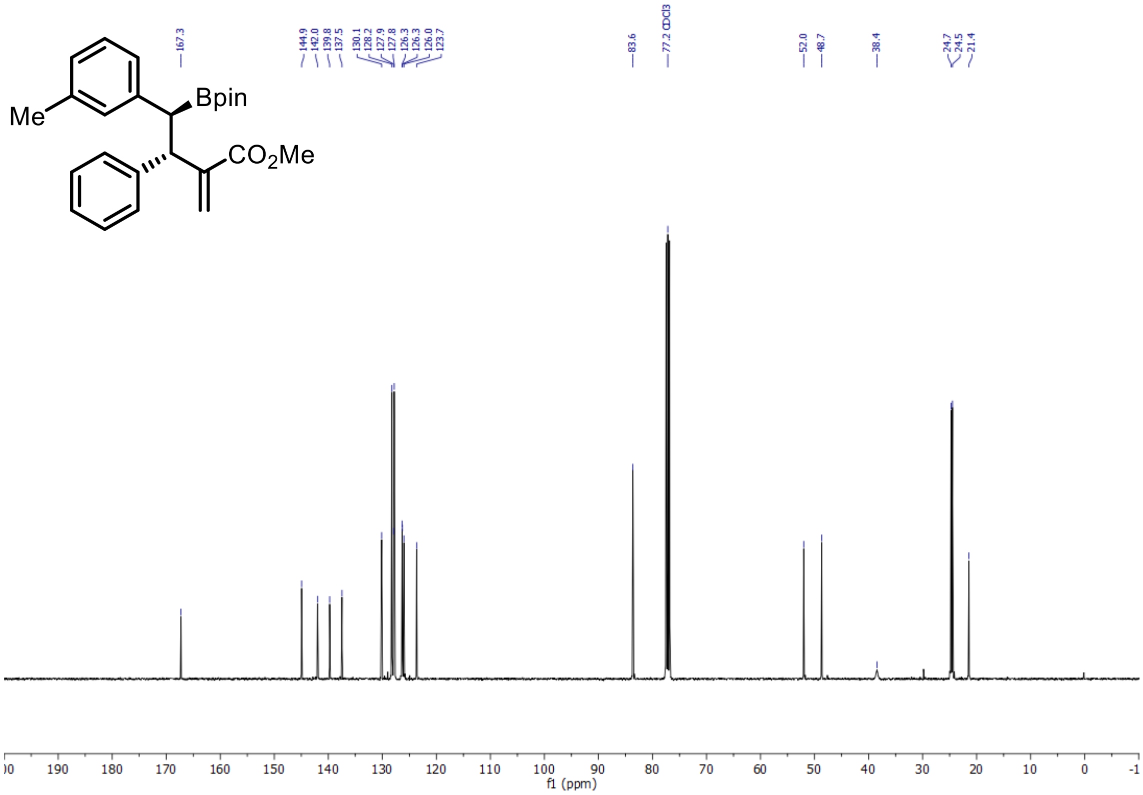

^{13}C NMR (101 MHz, $CDCl_3$) spectra of **3g**.

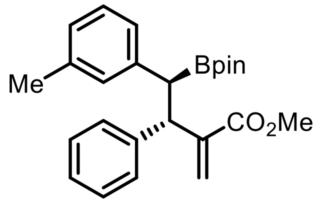

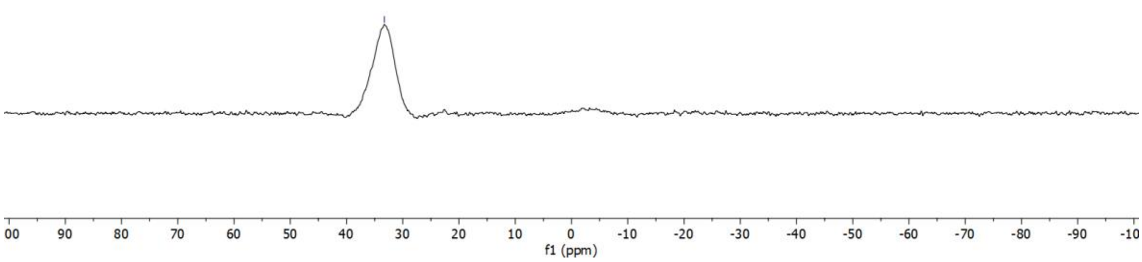

^{11}B NMR (128 MHz, $CDCl_3$) spectra of **3g**.

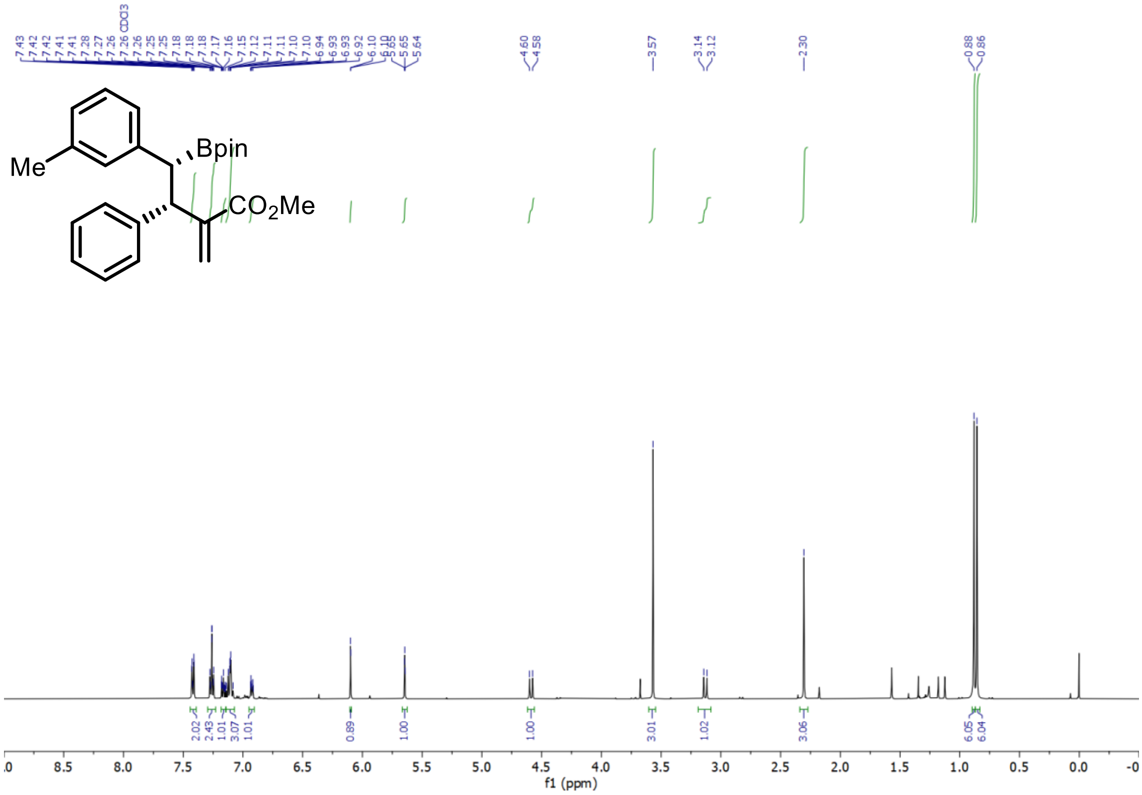

^{1}H NMR (500 MHz, $CDCl_3$) spectra of **3g'**.

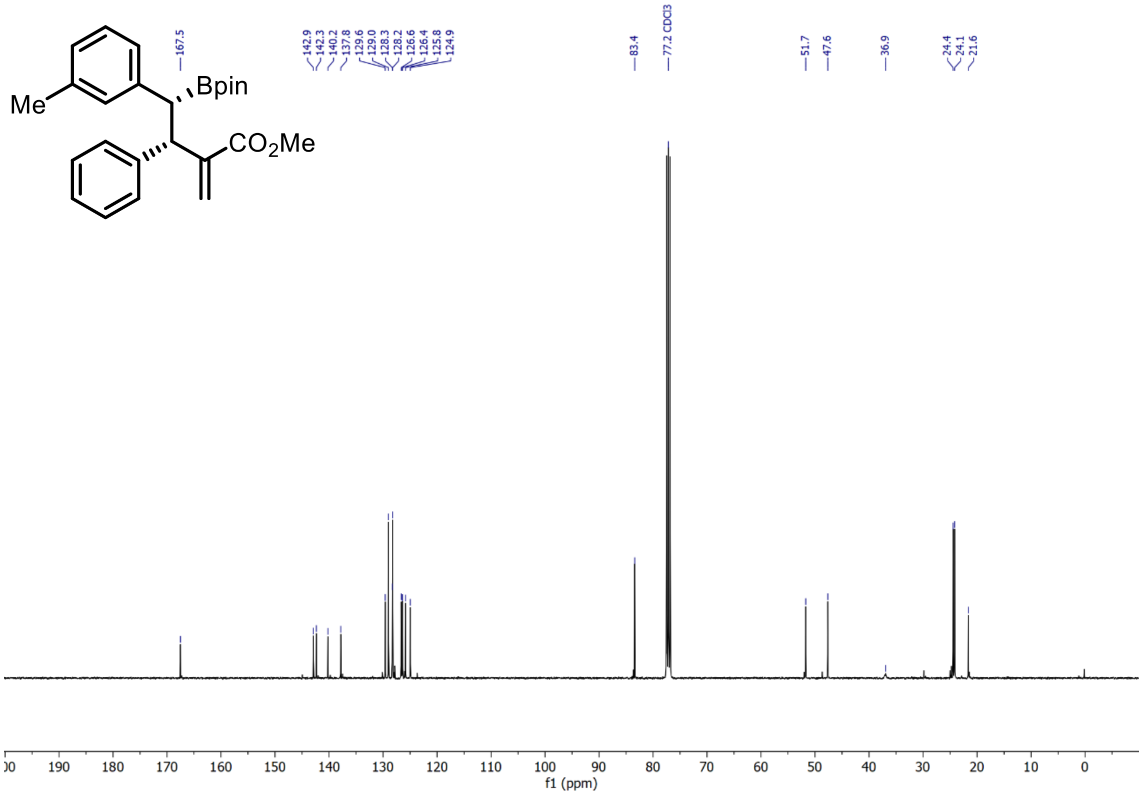

^{13}C NMR (101 MHz, $CDCl_3$) spectra of **3g'**.

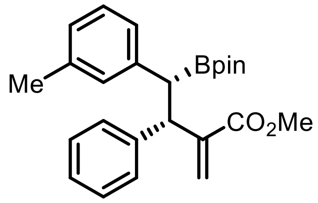

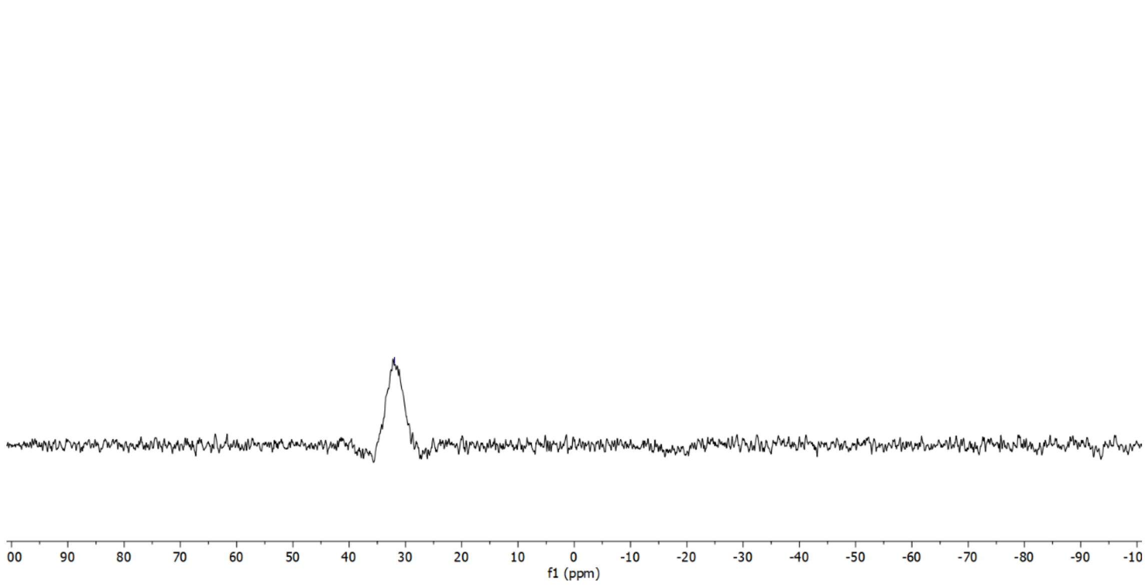

^{11}B NMR (128 MHz, $CDCl_3$) spectra of **3g’**.

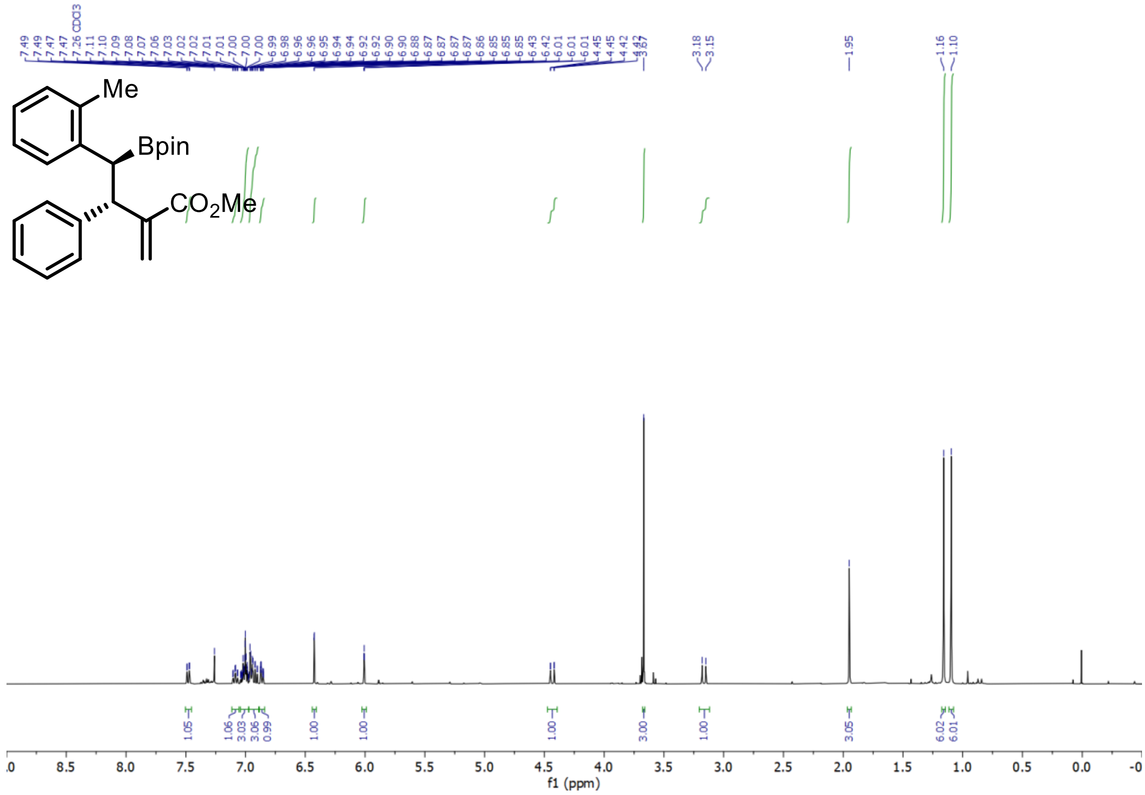

^{1}H NMR (400 MHz, $CDCl_3$) spectra of **3h**.

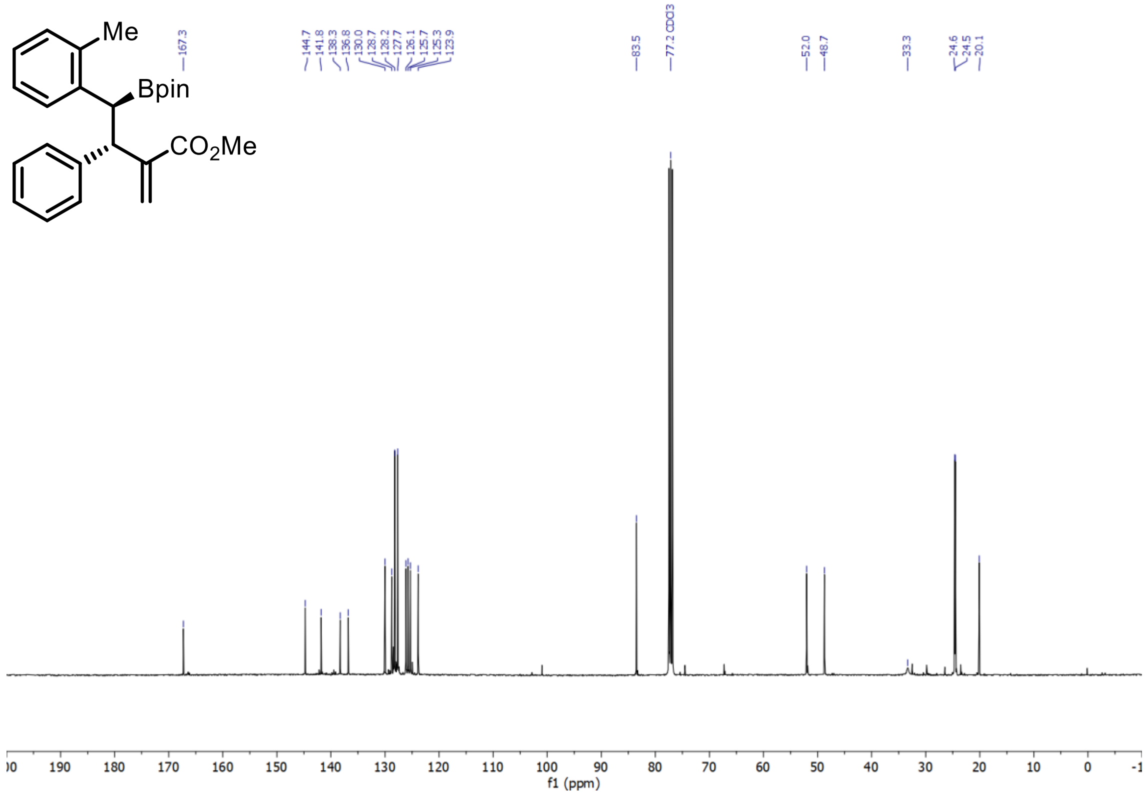

^{13}C NMR (101 MHz, $CDCl_3$) spectra of **3h**.

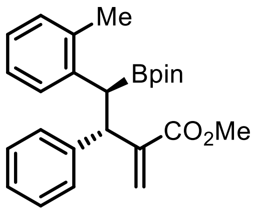

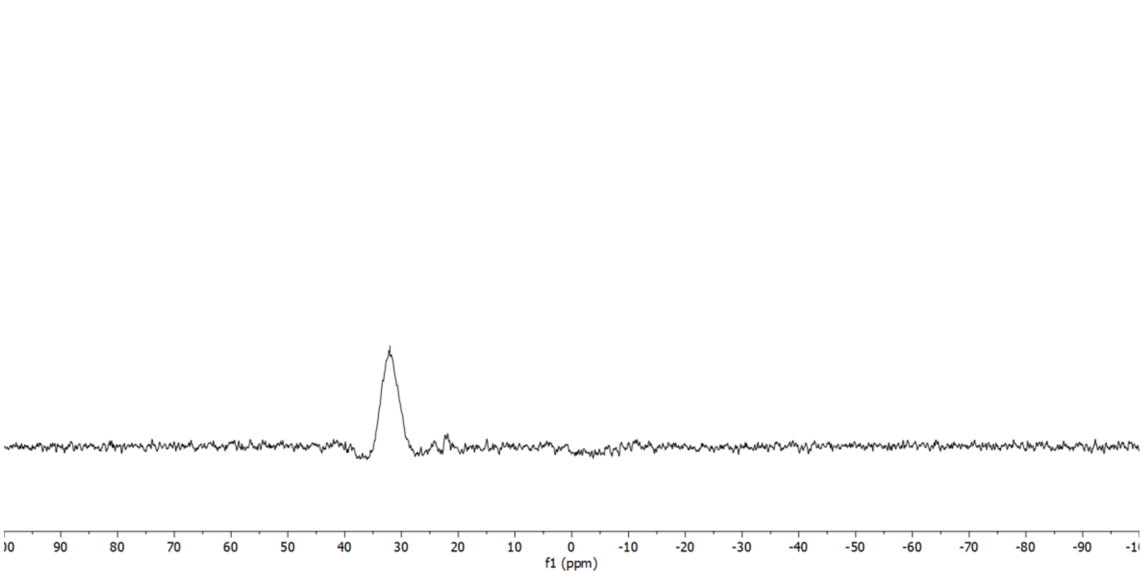

^{11}B NMR (128 MHz, $CDCl_3$) spectra of **3h**.

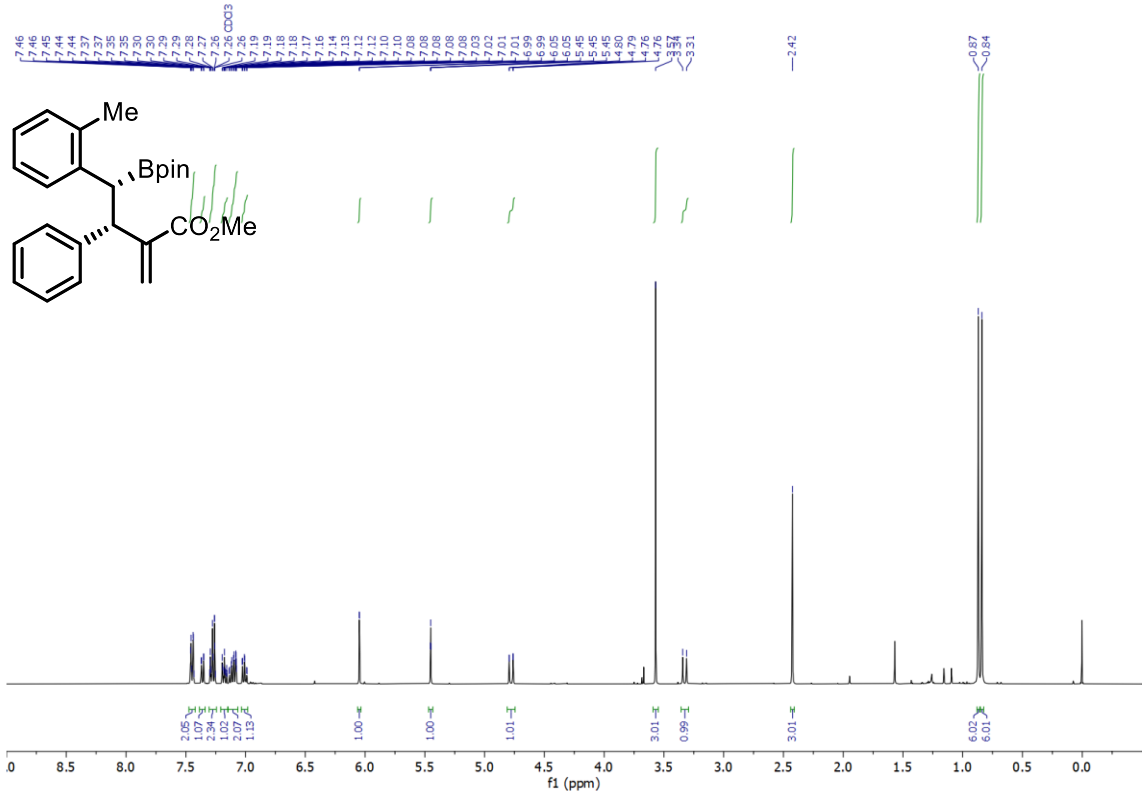

^{1}H NMR (400 MHz, $CDCl_3$) spectra of **3h'**.

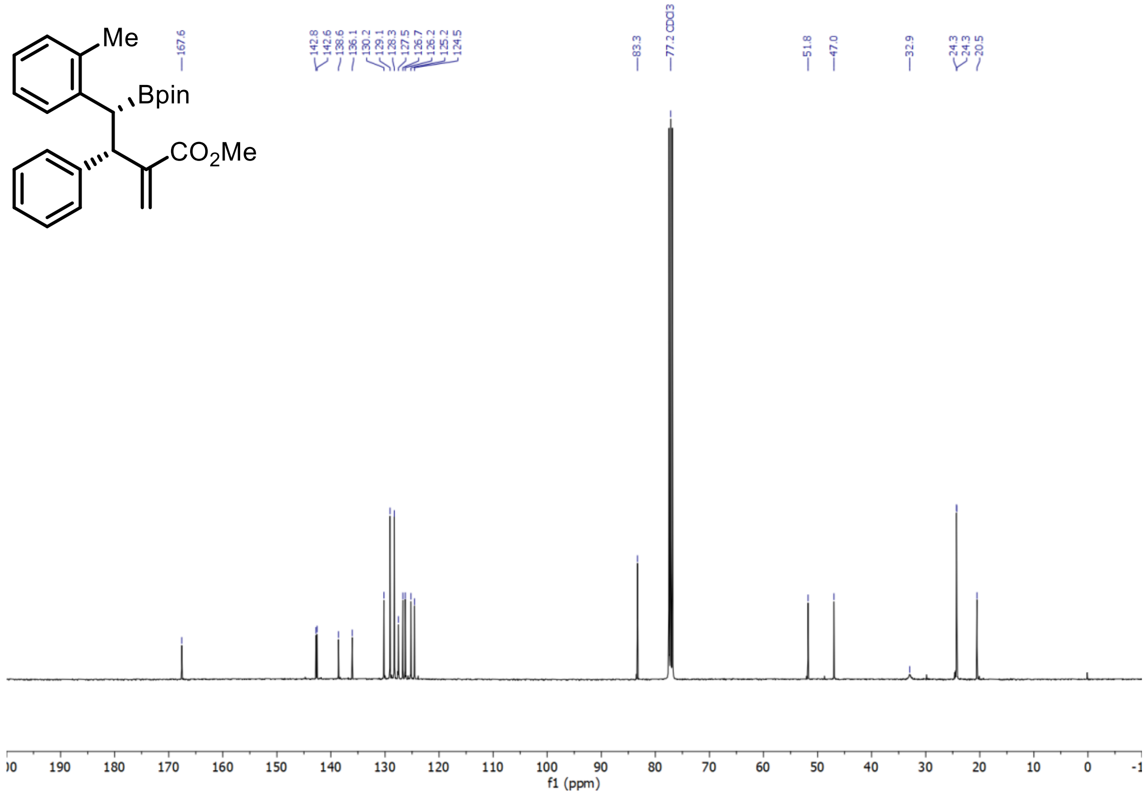

^{13}C NMR (101 MHz, $CDCl_3$) spectra of **3h'**.

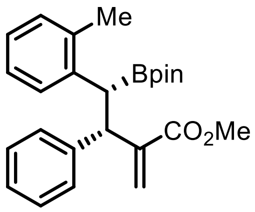

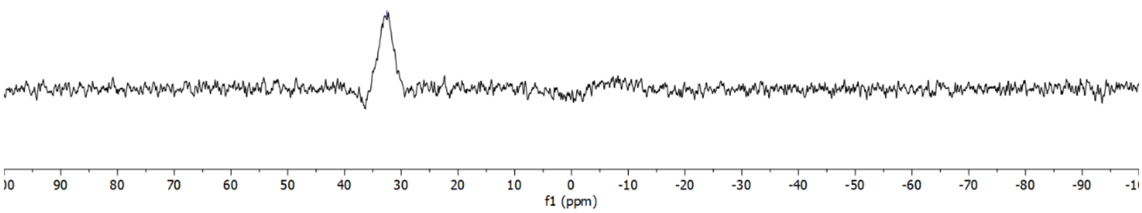

^{11}B NMR (128 MHz, $CDCl_3$) spectra of **3h'**.

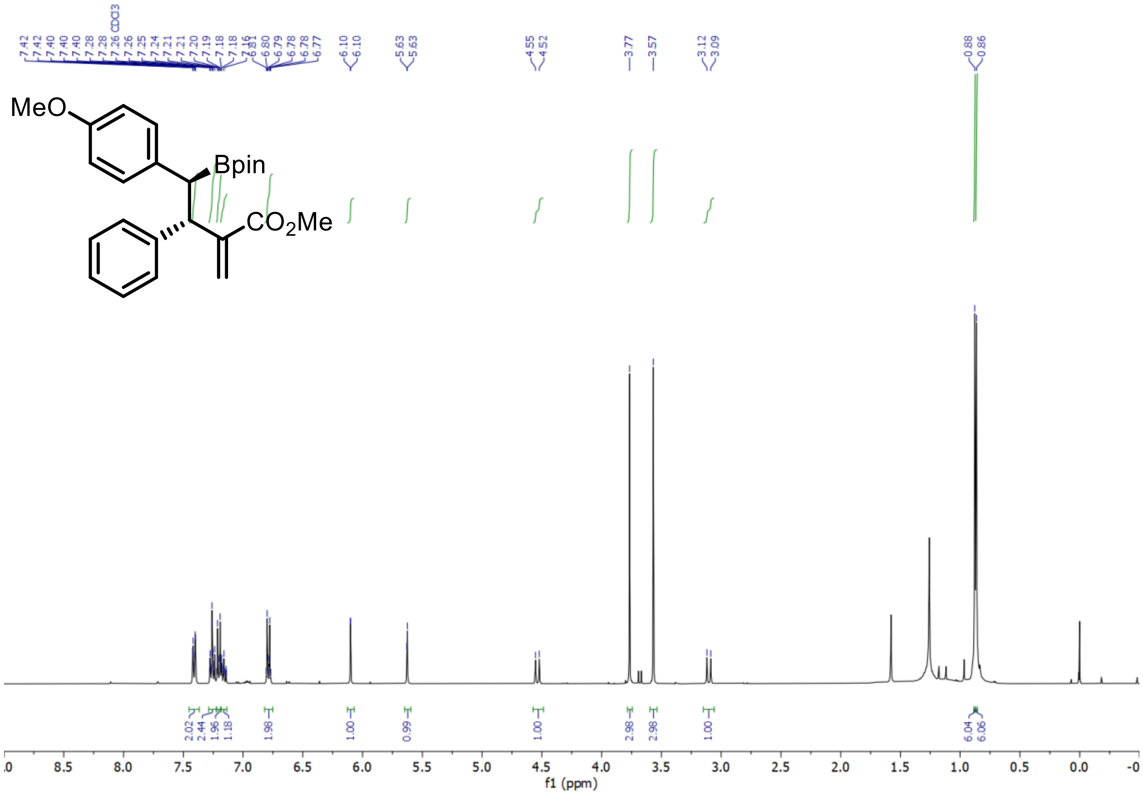

^{1}H NMR (400 MHz, $CDCl_3$) spectra of **3i**.

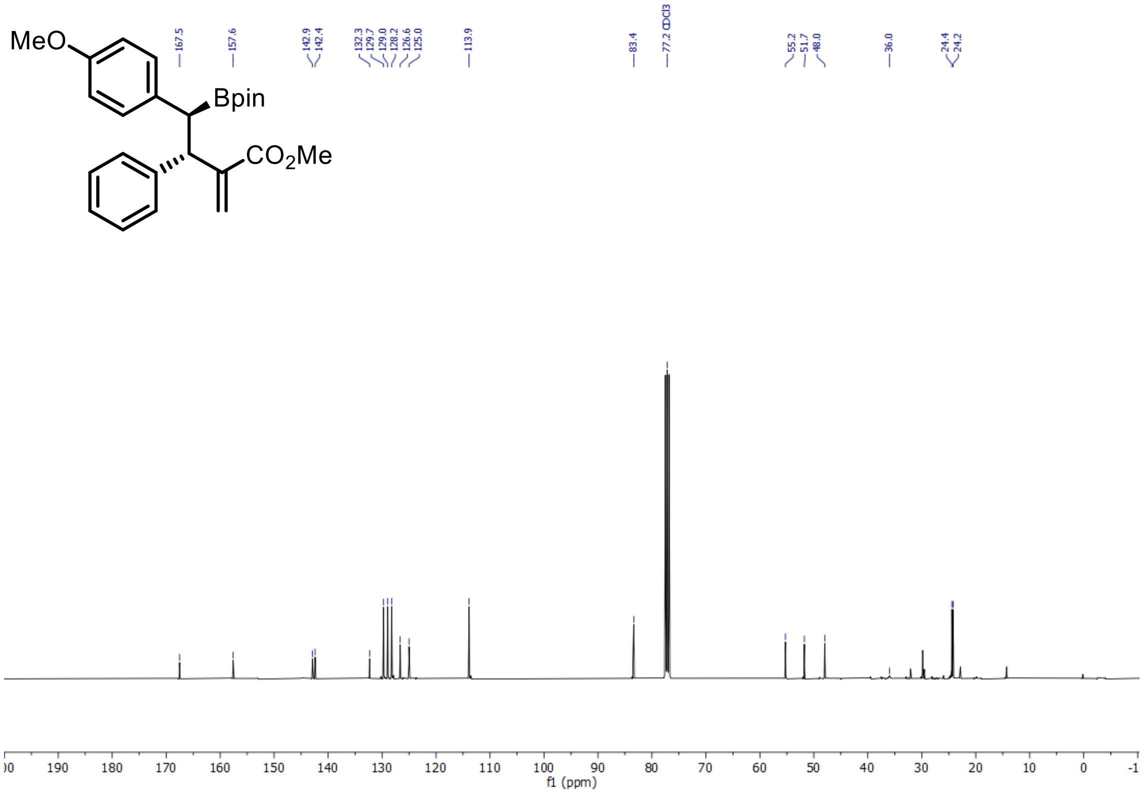

^{13}C NMR (101 MHz, $CDCl_3$) spectra of **3i**.

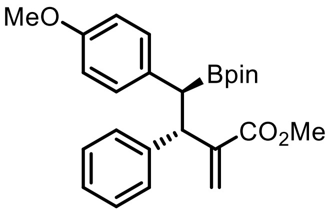

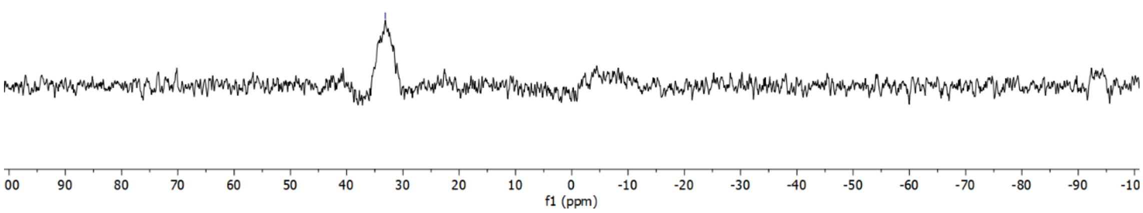

^{11}B NMR (128 MHz, $CDCl_3$) spectra of **3i**.

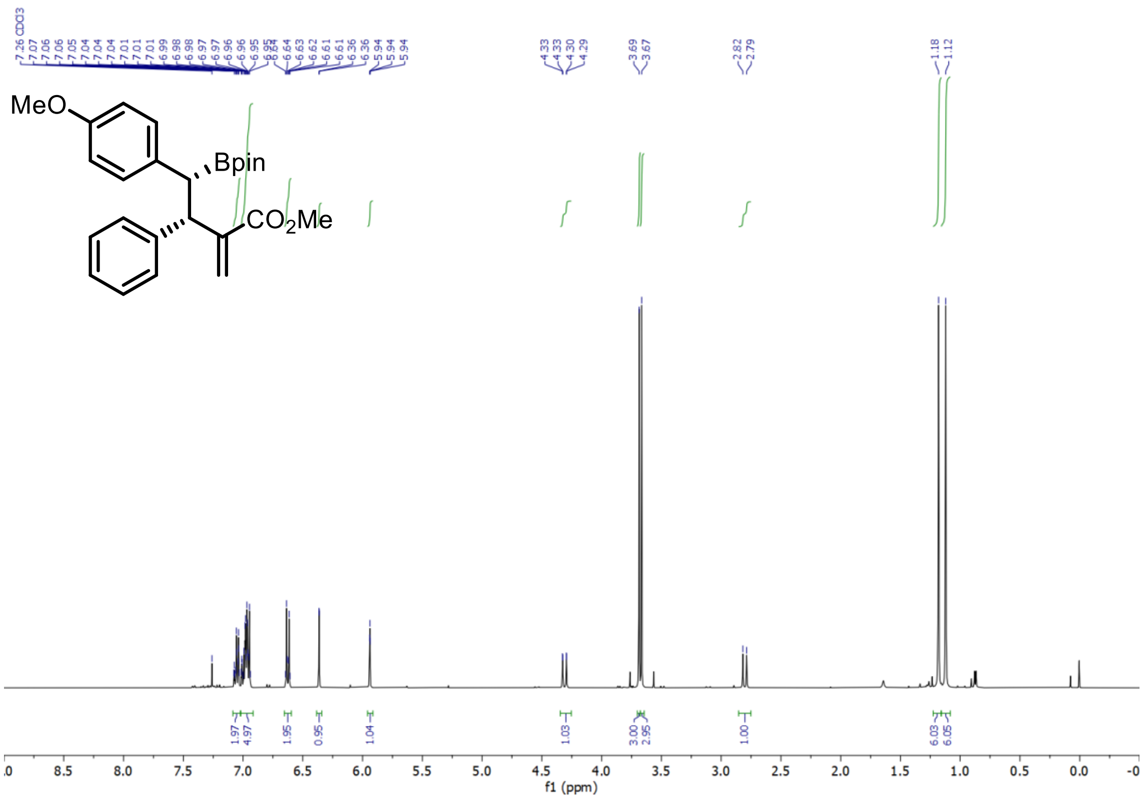

^{1}H NMR (400 MHz, $CDCl_3$) spectra of **3i'**.

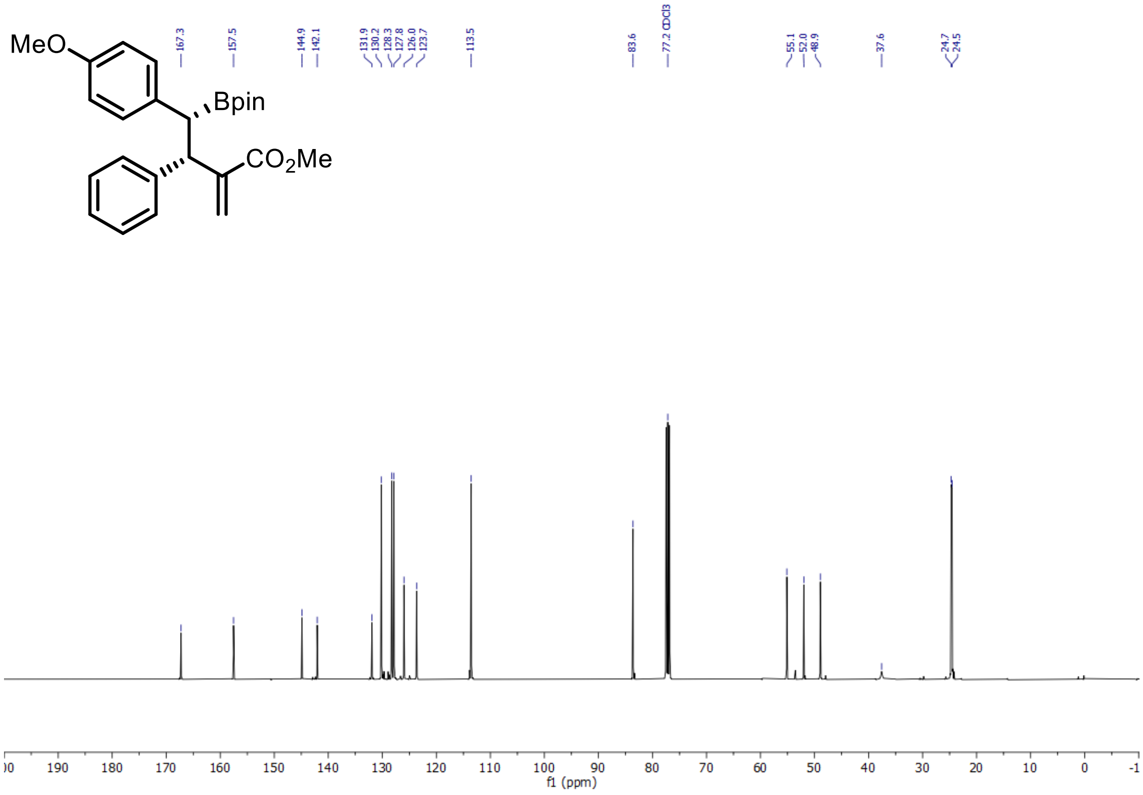

^{13}C NMR (101 MHz, $CDCl_3$) spectra of **3i'**.

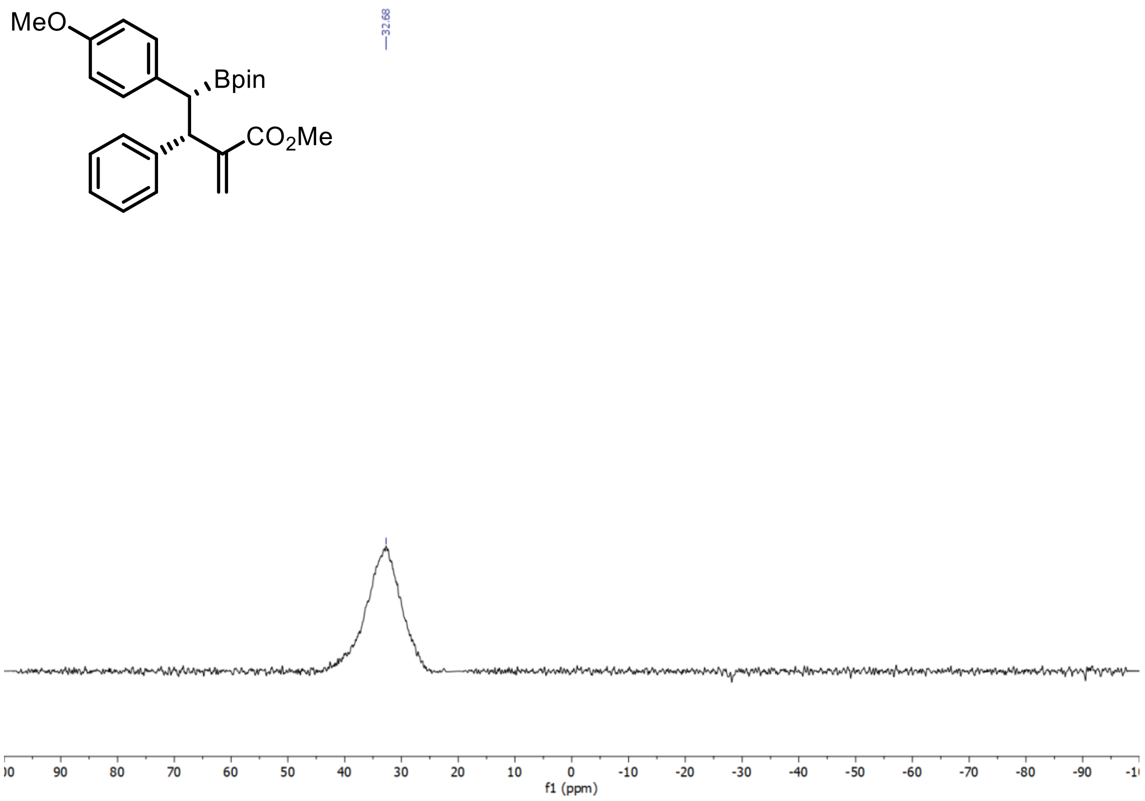

^{11}B NMR (128 MHz, $CDCl_3$) spectra of **3i’**.

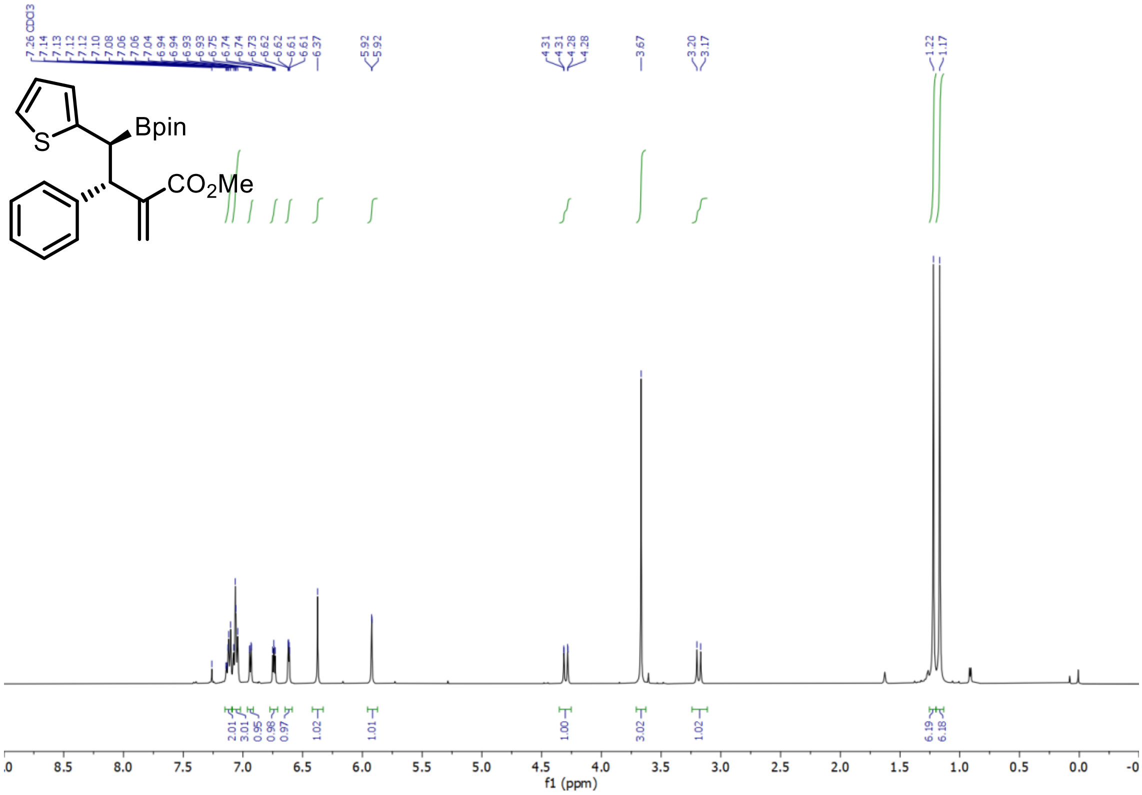

^{1}H NMR (400 MHz, $CDCl_3$) spectra of **3j**.

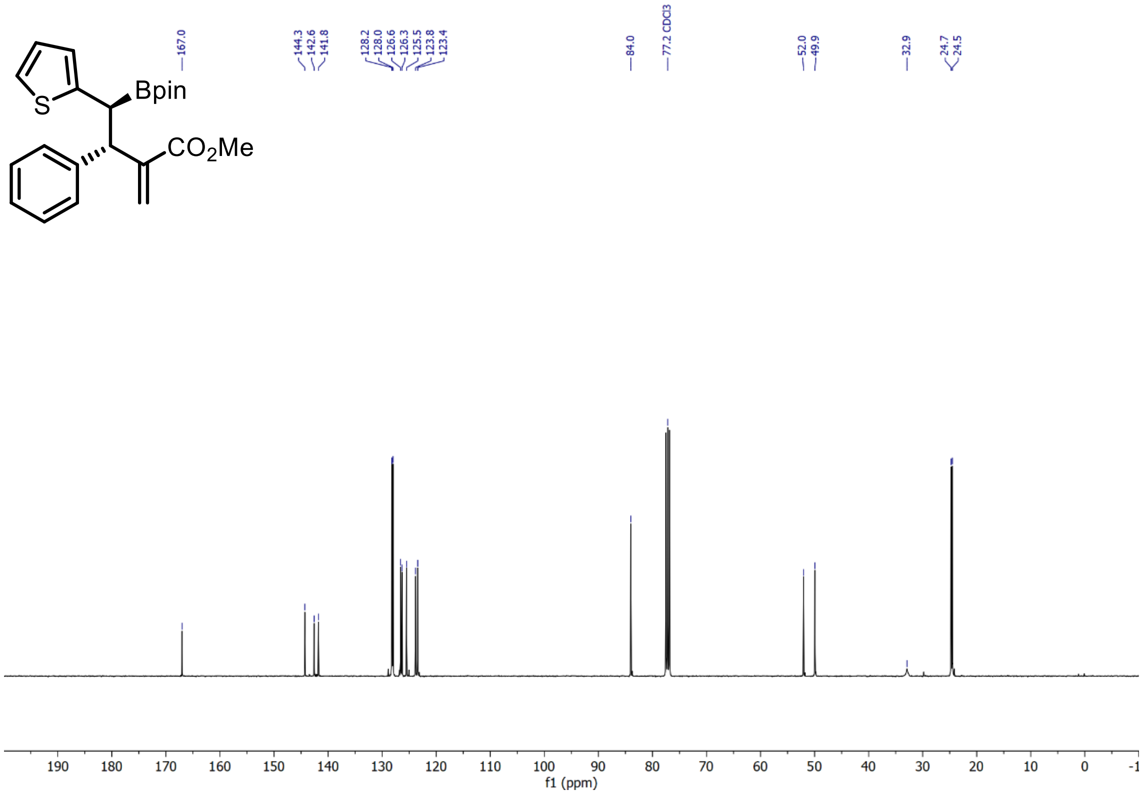

^{13}C NMR (101 MHz, $CDCl_3$) spectra of **3j**.

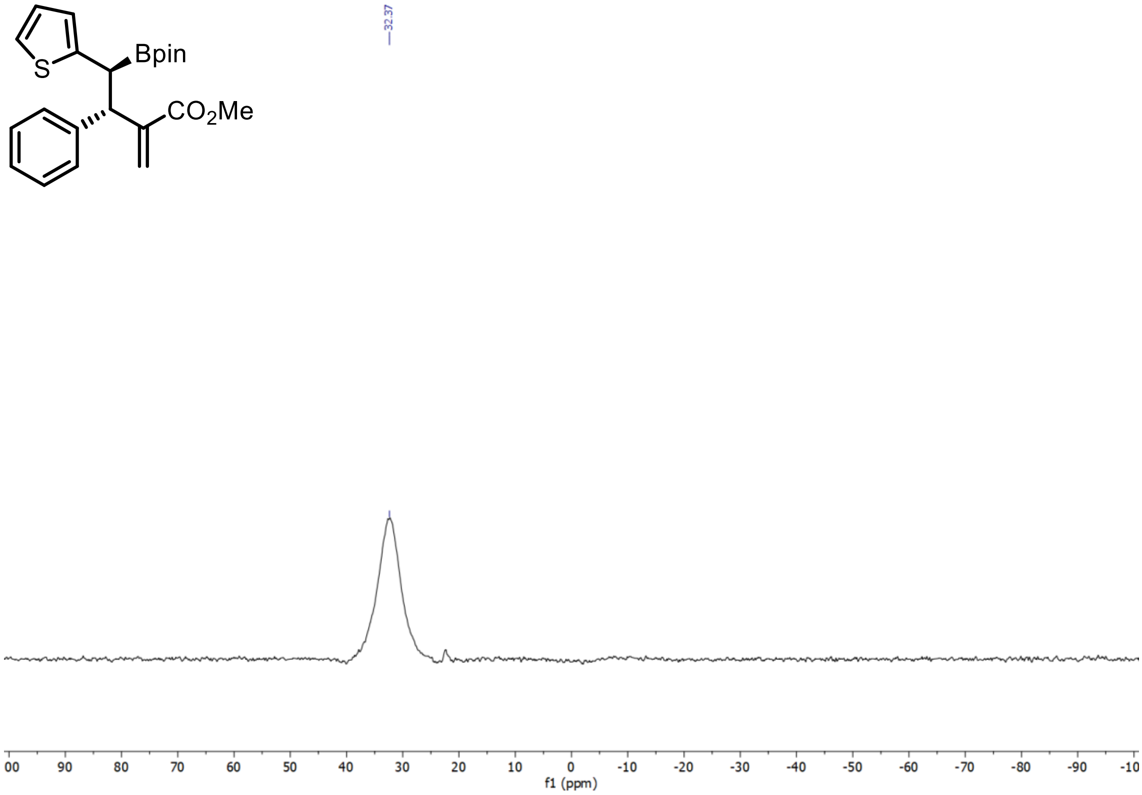

^{11}B NMR (128 MHz, $CDCl_3$) spectra of **3j**.

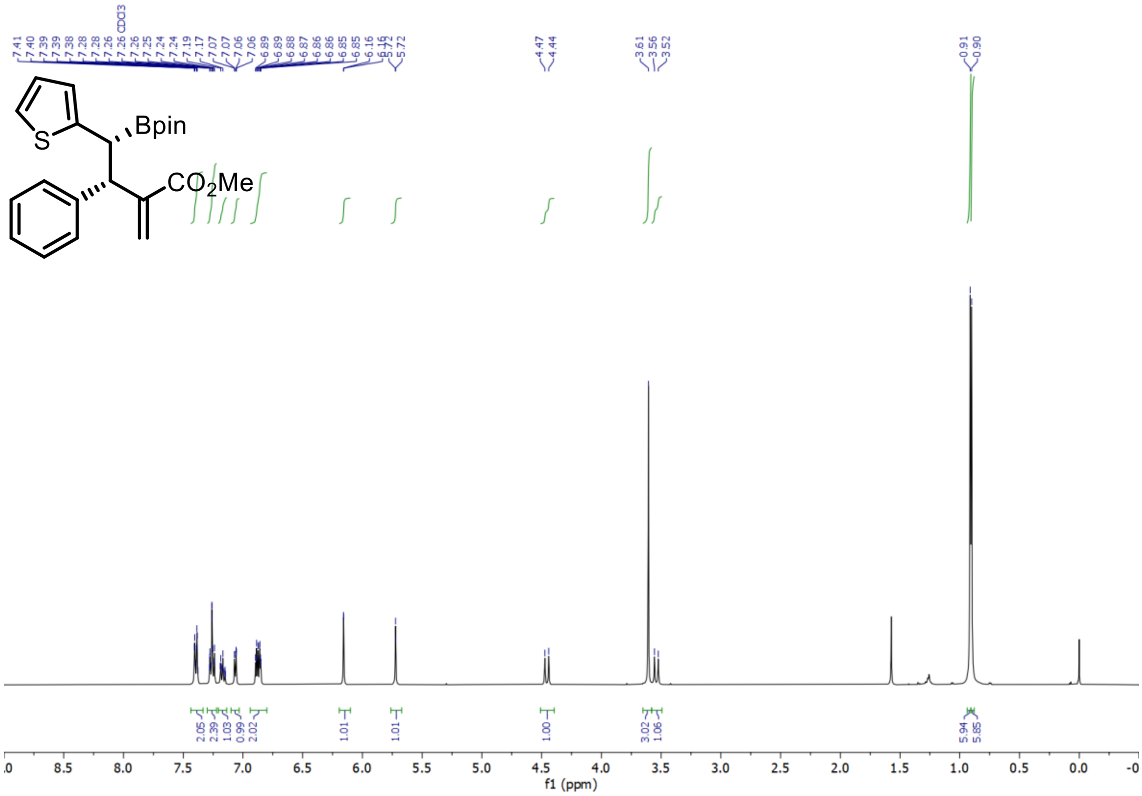

^{1}H NMR (400 MHz, $CDCl_3$) spectra of **3j'**.

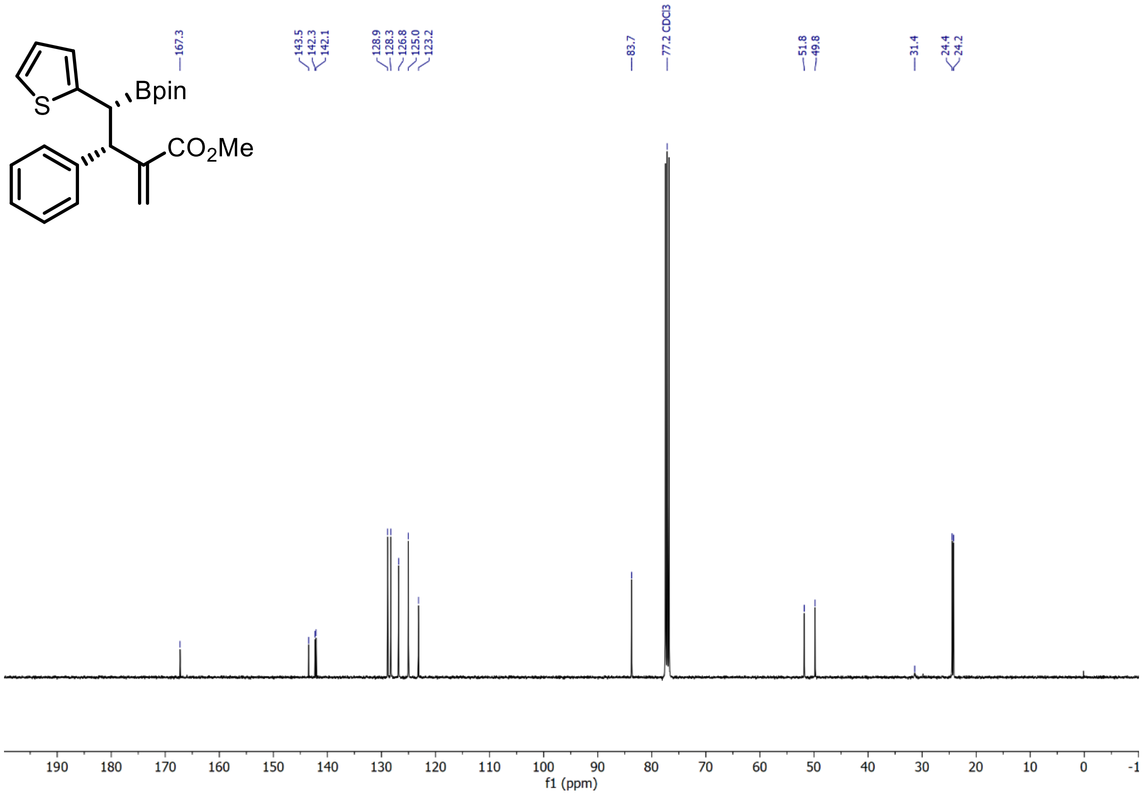

^{13}C NMR (101 MHz, $CDCl_3$) spectra of **3j'**.

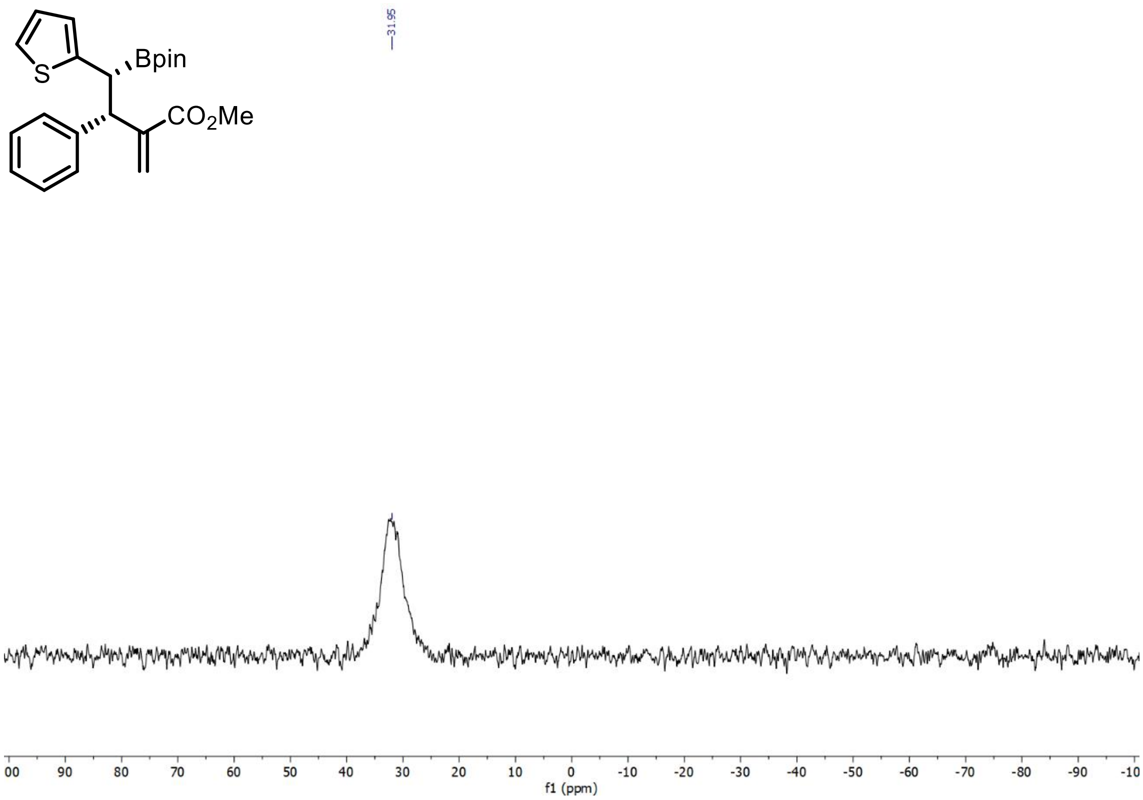

^{11}B NMR (128 MHz, $CDCl_3$) spectra of **3j'**.

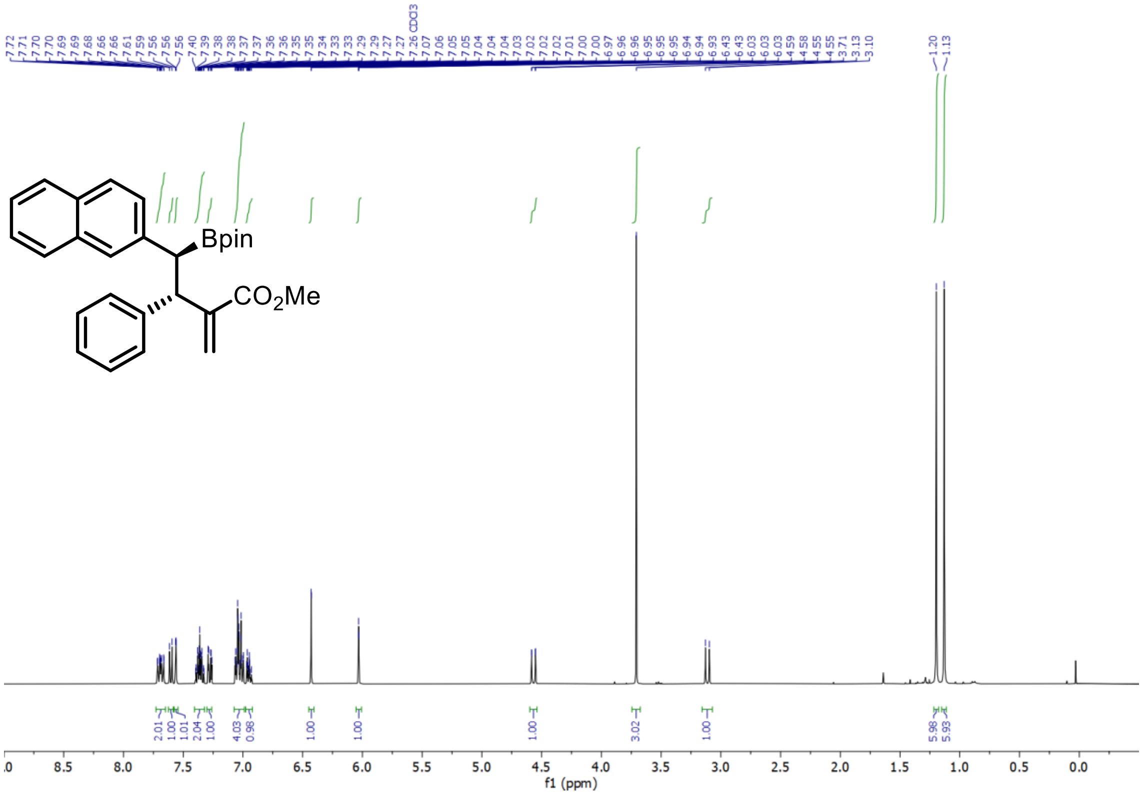

^{1}H NMR (400 MHz, $CDCl_3$) spectra of **3k**.

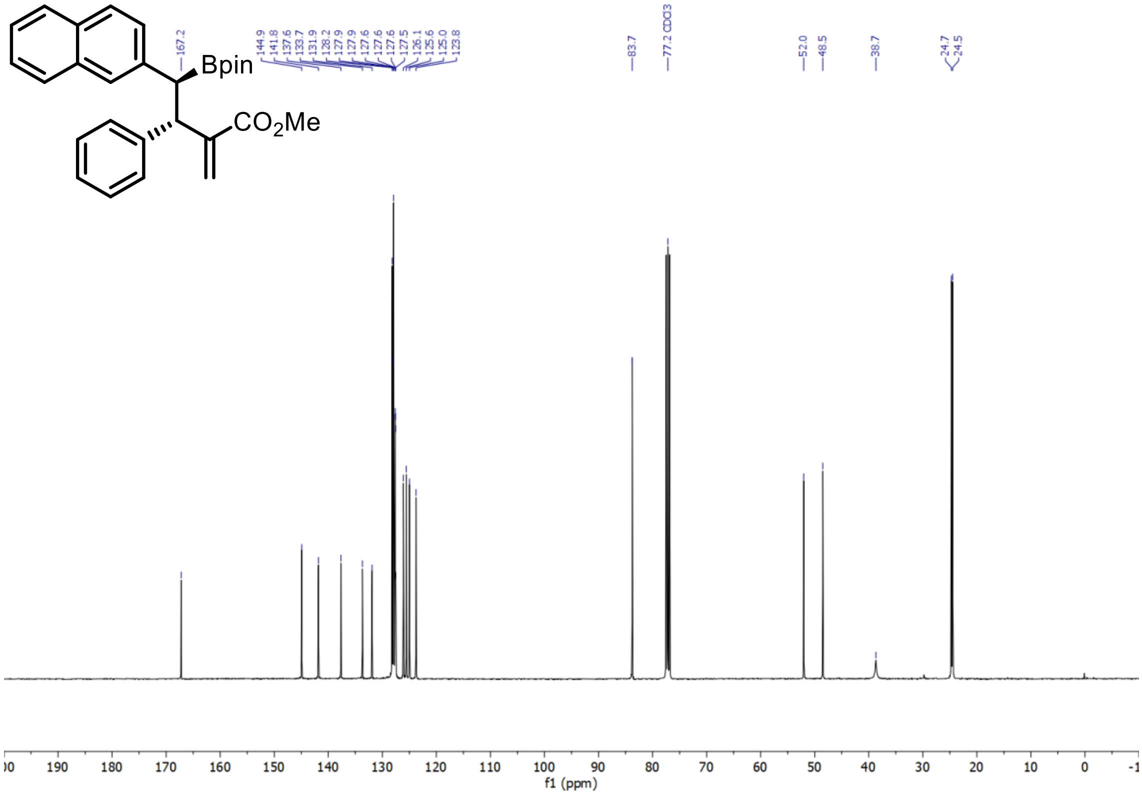

^{13}C NMR (101 MHz, $CDCl_3$) spectra of **3k**.

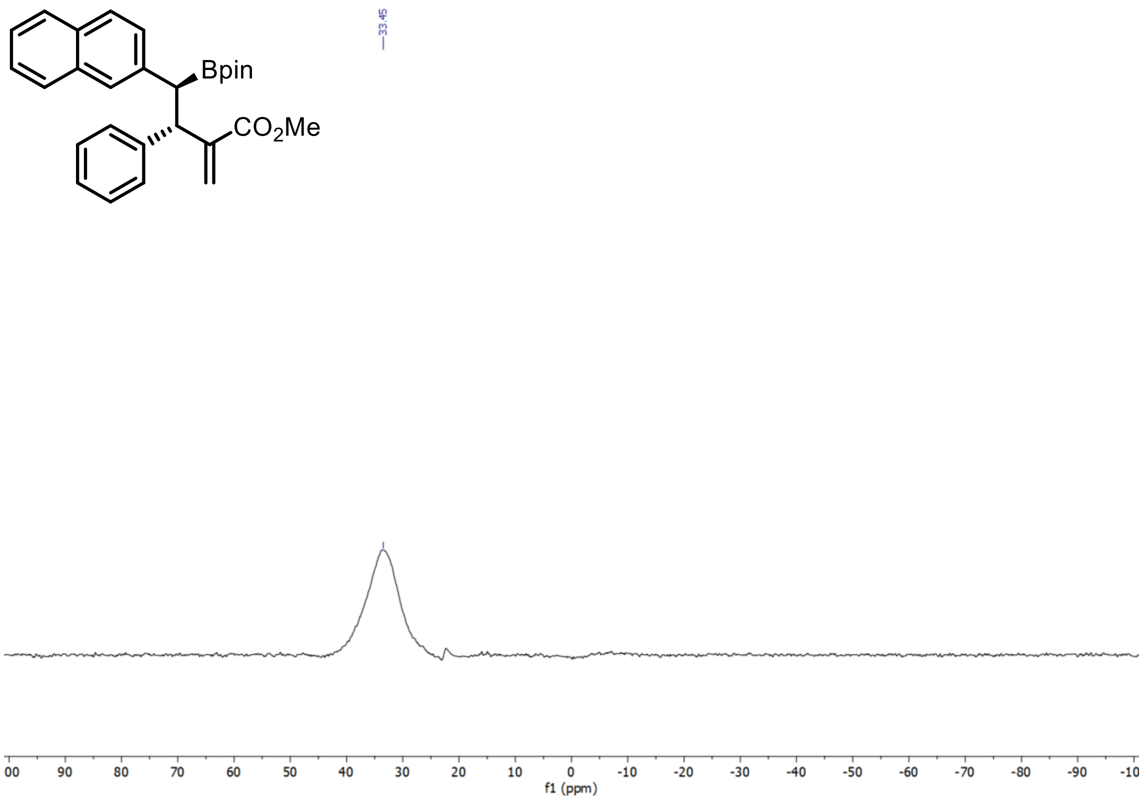

^{11}B NMR (128 MHz, $CDCl_3$) spectra of **3k**.

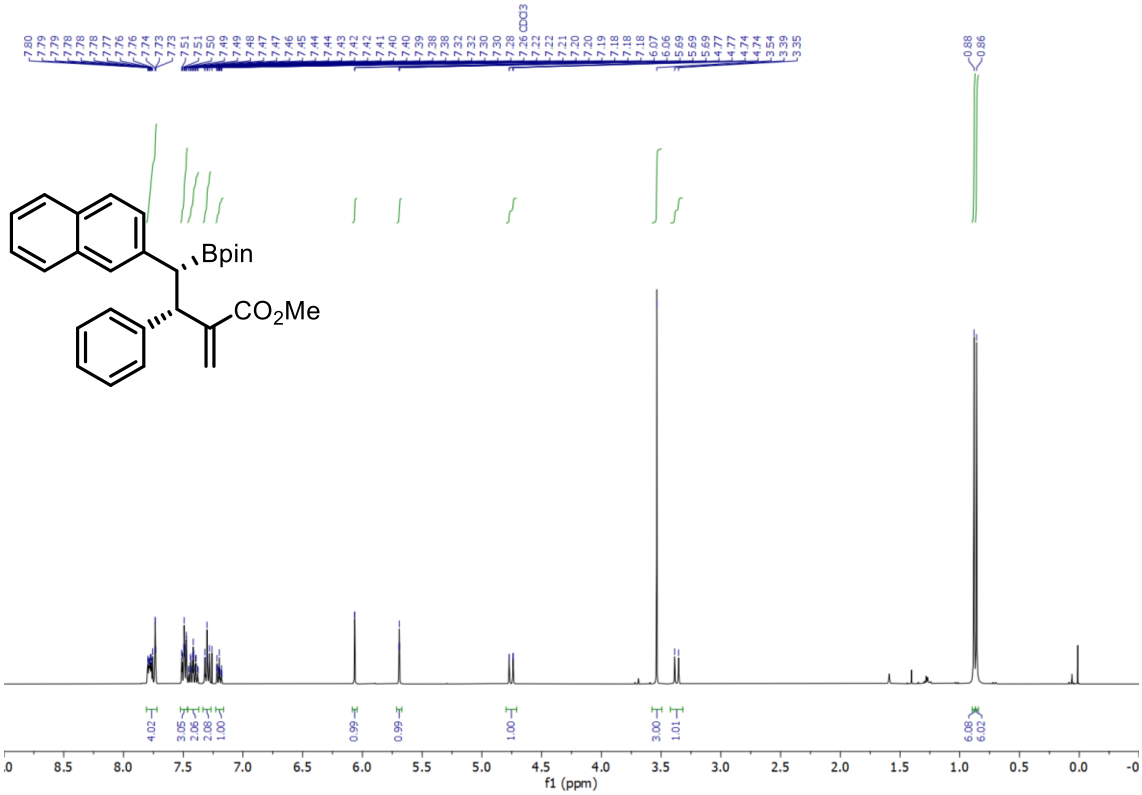

^{1}H NMR (400 MHz, $CDCl_3$) spectra of **3k’**.

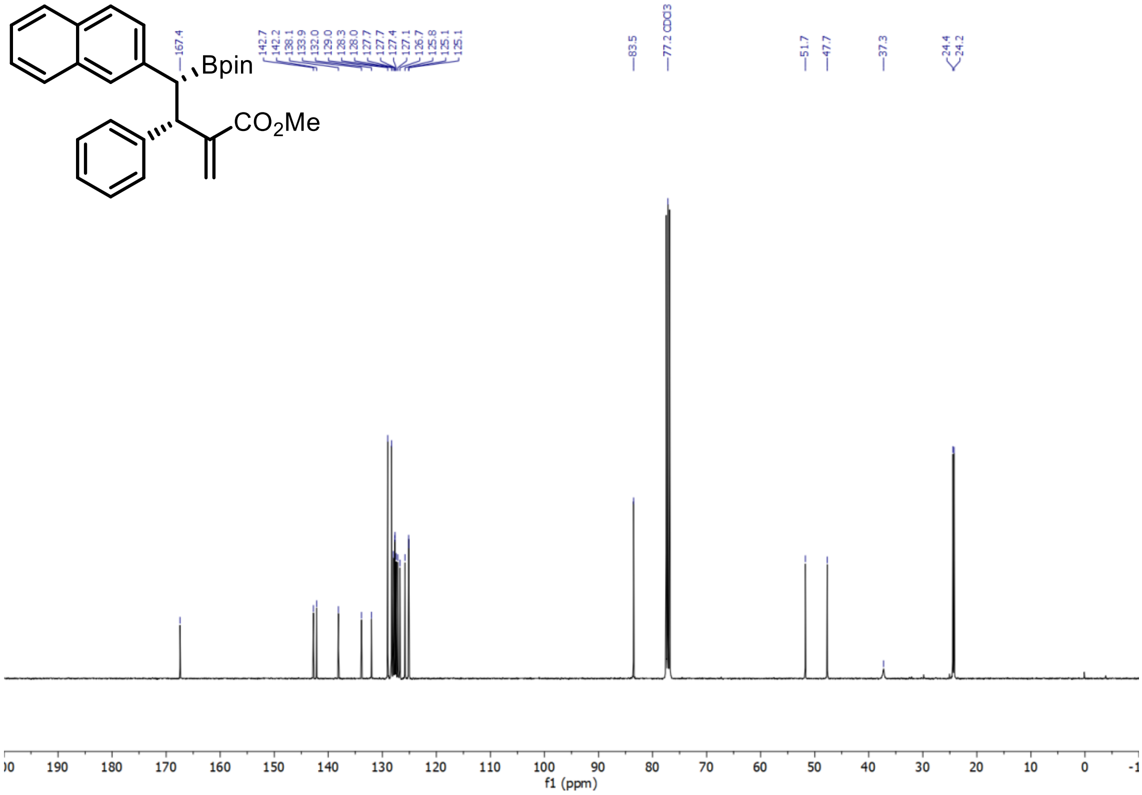

^{13}C NMR (101 MHz, $CDCl_3$) spectra of **3k’**.

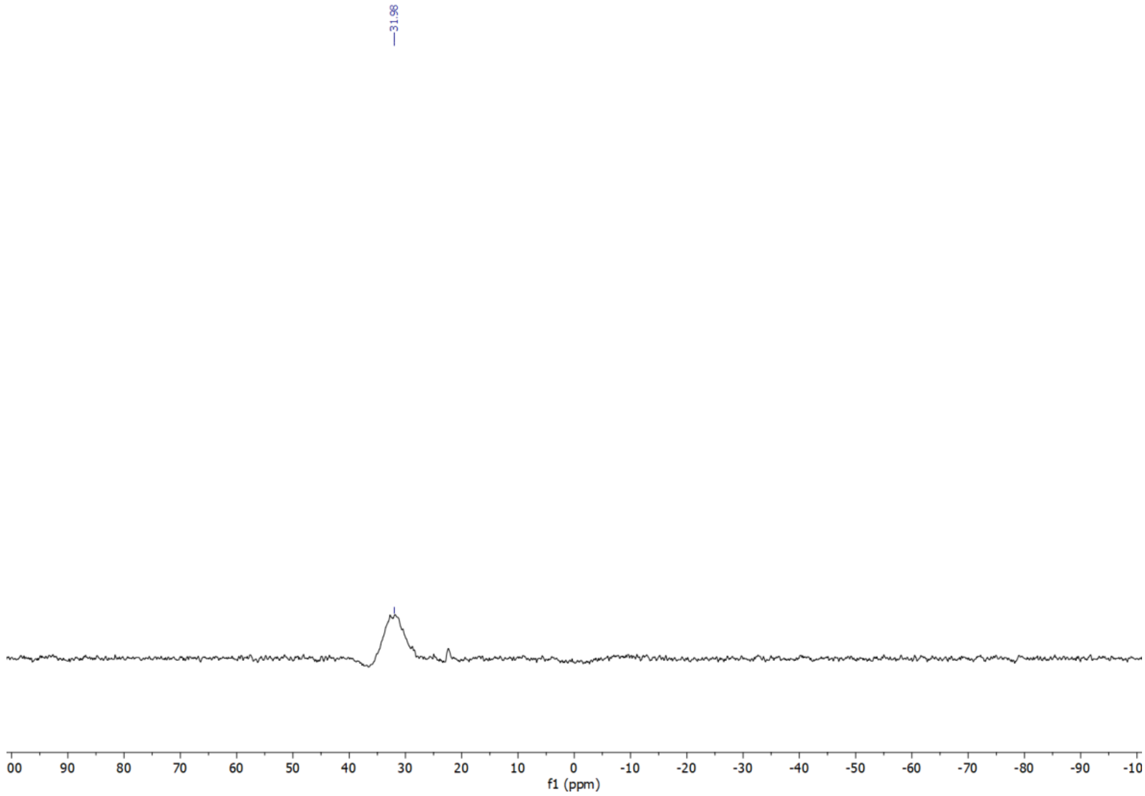

^{11}B NMR (128 MHz, $CDCl_3$) spectra of **3k'**.

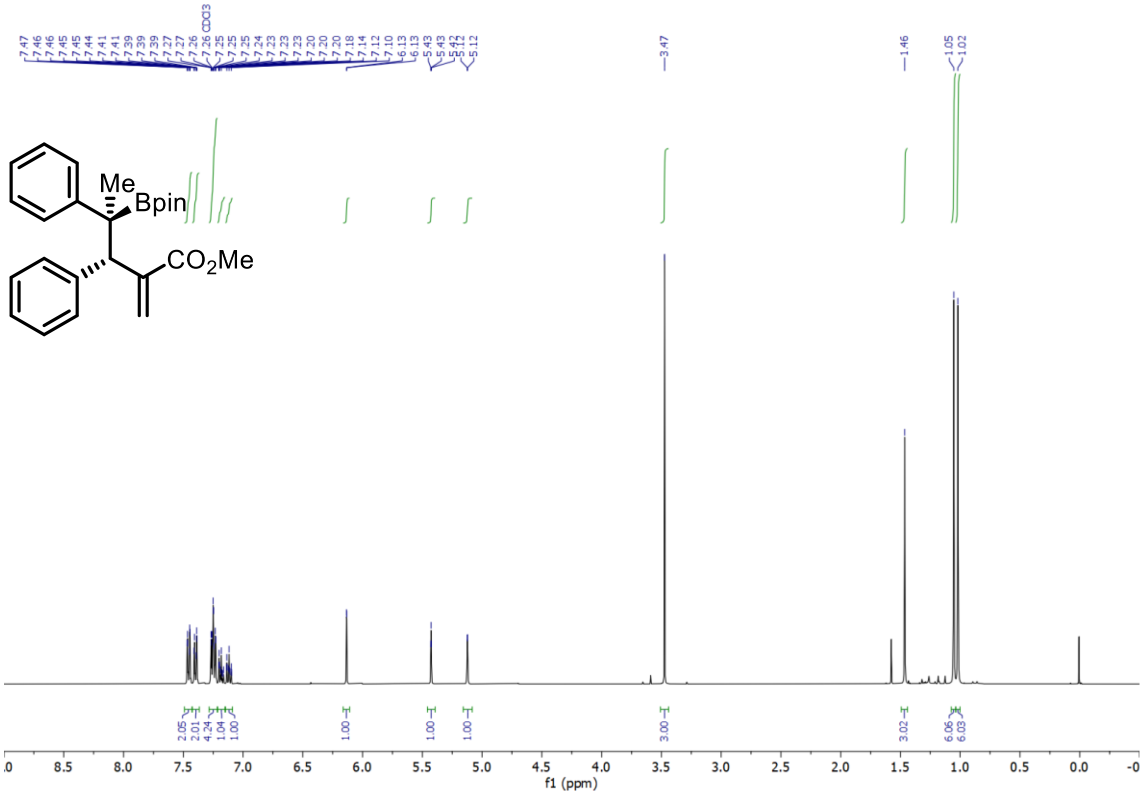

^{1}H NMR (400 MHz, $CDCl_3$) spectra of **3l**.

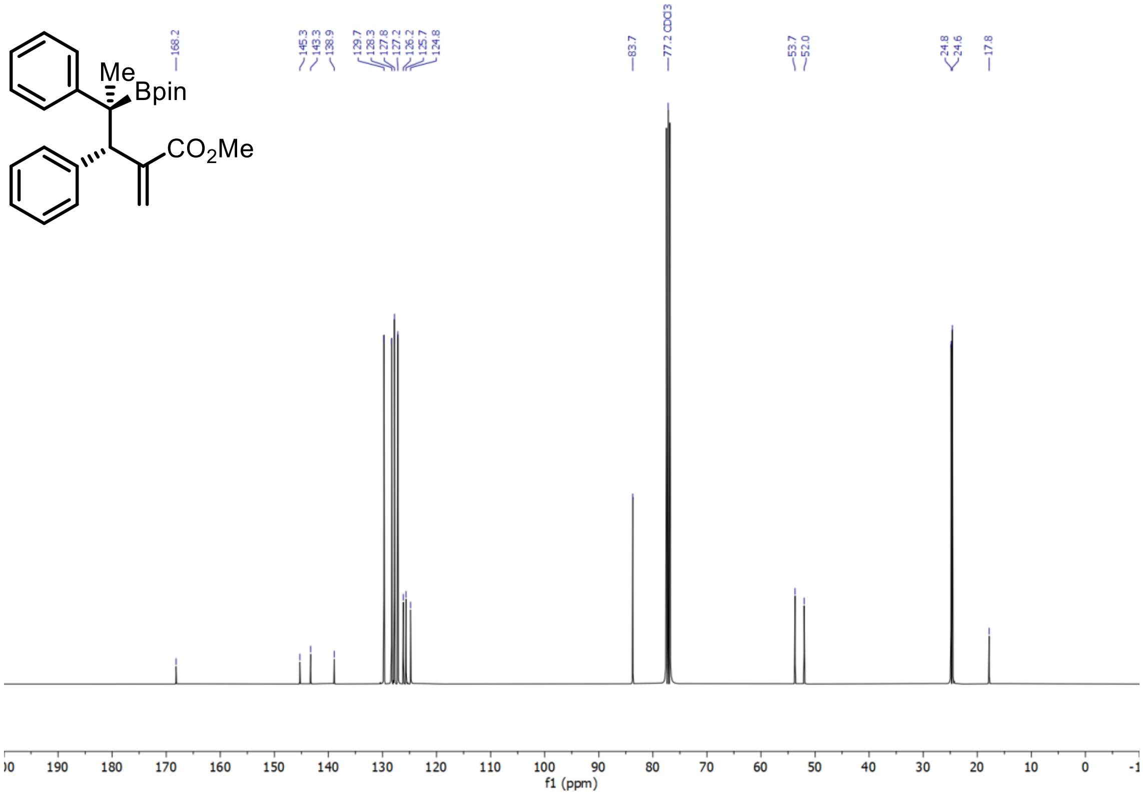

^{13}C NMR (101 MHz, $CDCl_3$) spectra of **3l**.

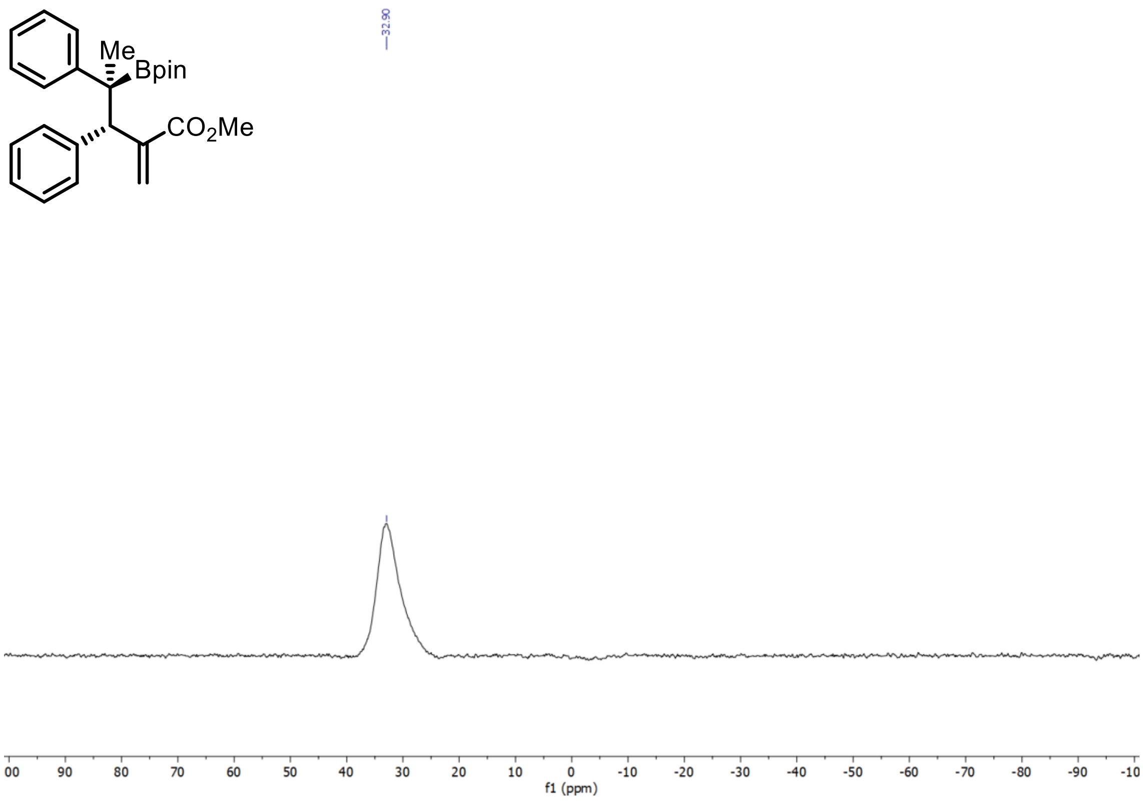

^{11}B NMR (128 MHz, $CDCl_3$) spectra of **3l**.

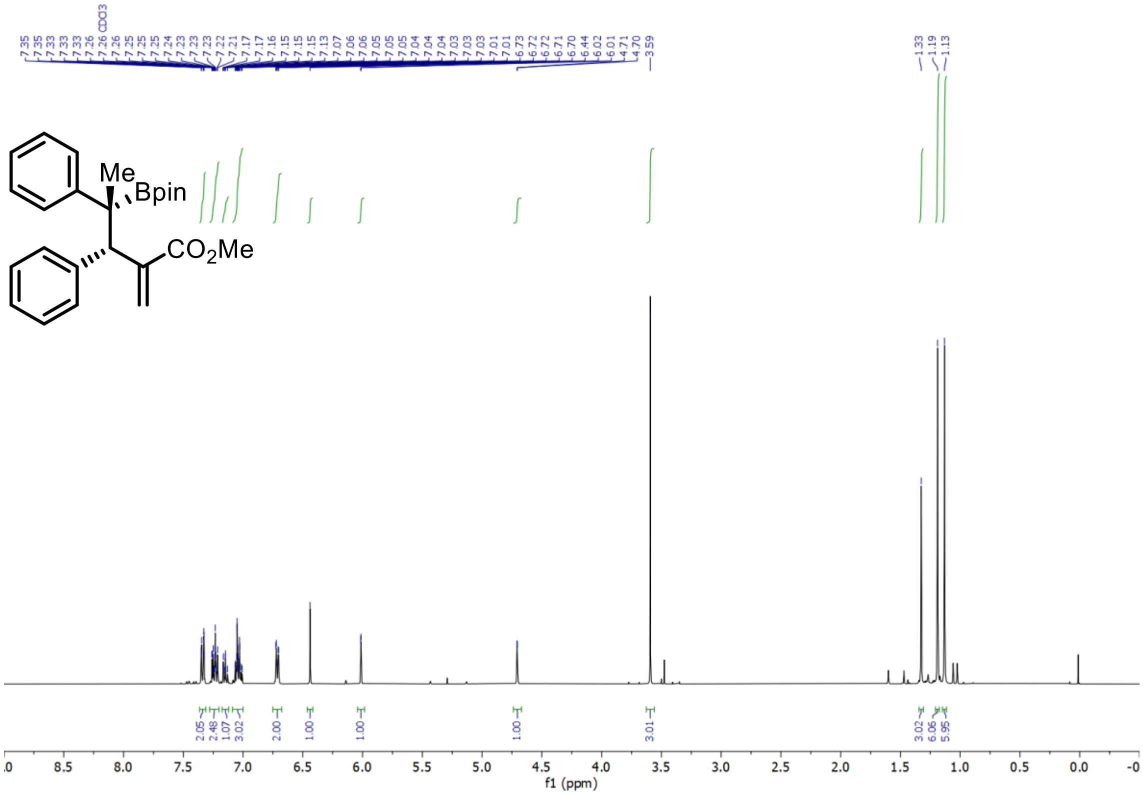

^{1}H NMR (400 MHz, $CDCl_3$) spectra of **3l’**.

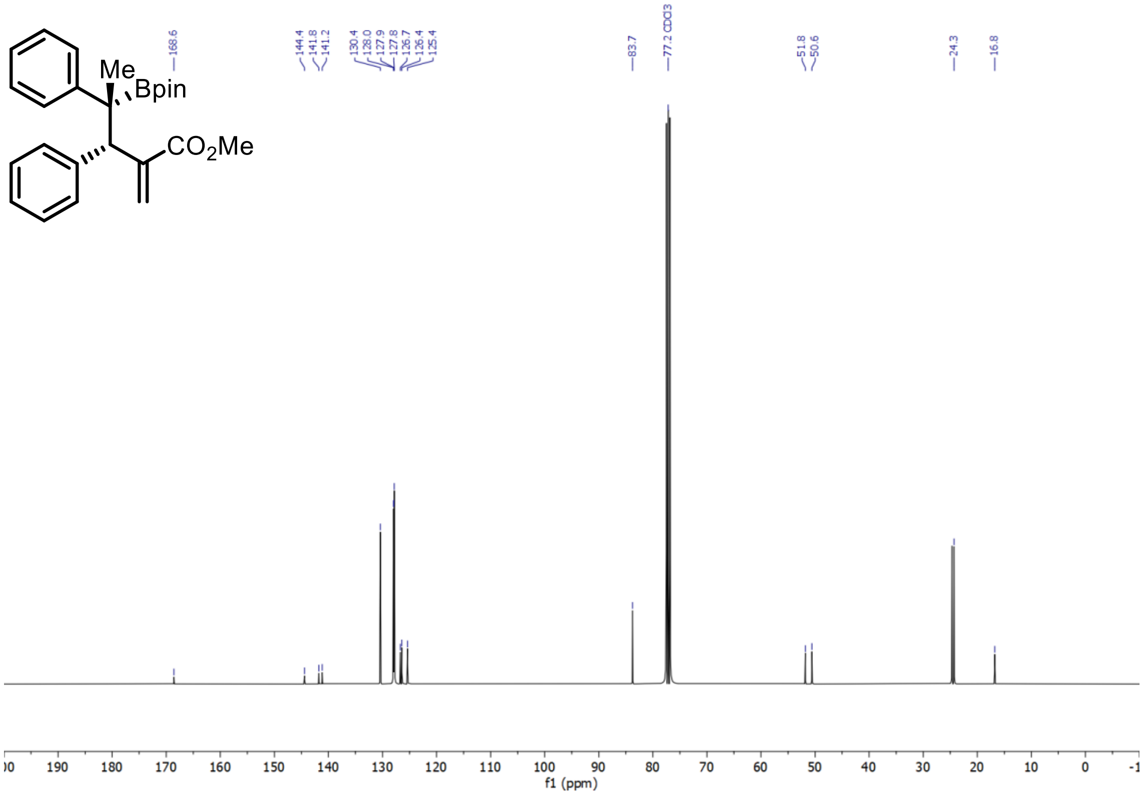

^{13}C NMR (101 MHz, $CDCl_3$) spectra of **3l’**.

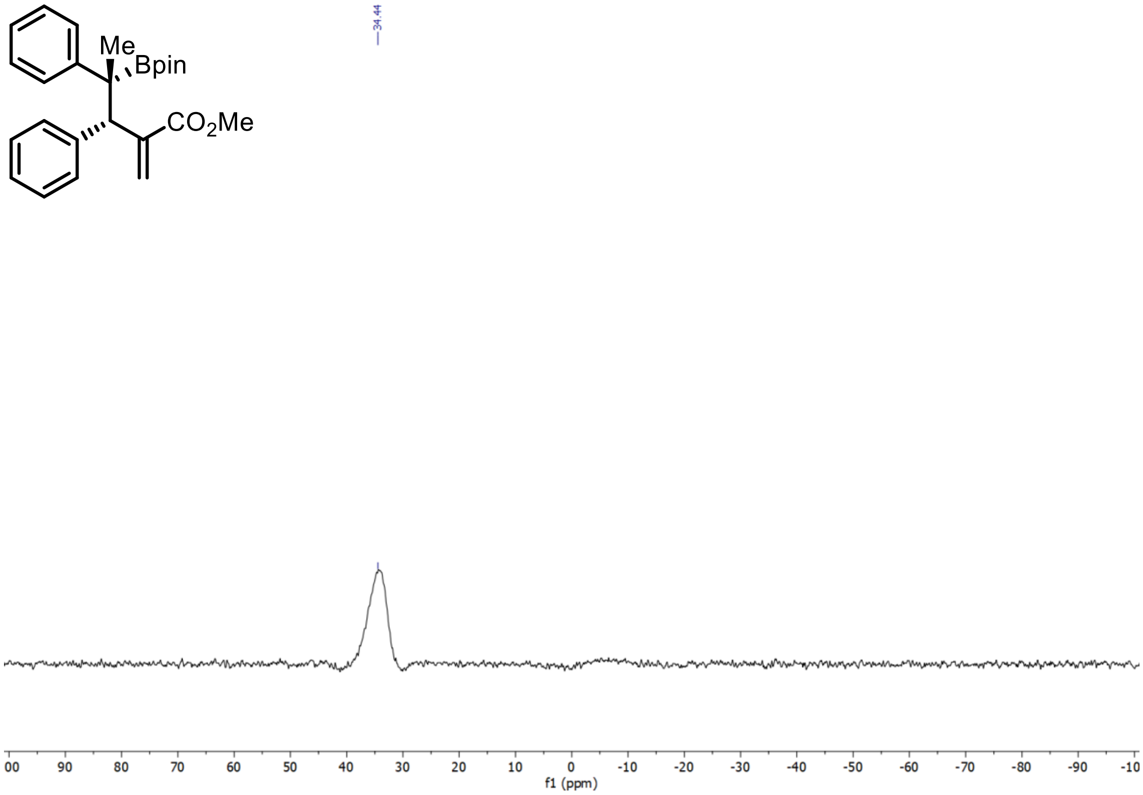

^{11}B NMR (128 MHz, $CDCl_3$) spectra of **3l’**.

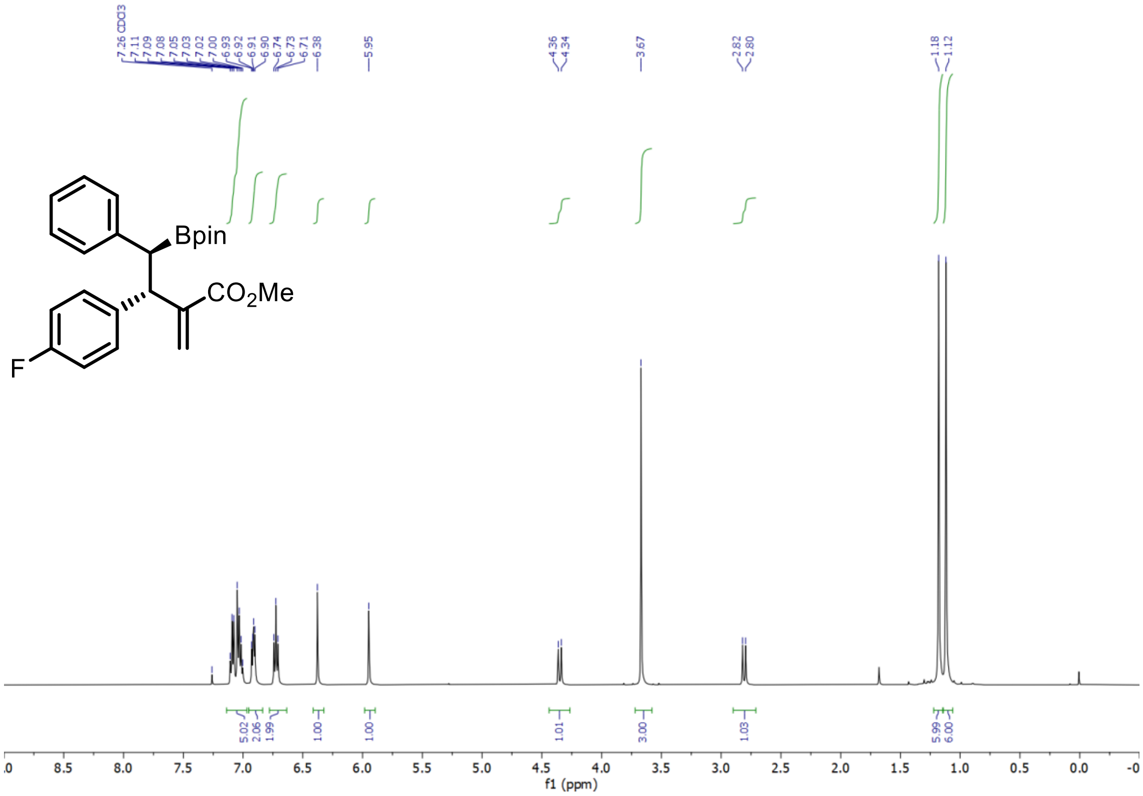

^{1}H NMR (500 MHz, $CDCl_3$) spectra of **3m**.

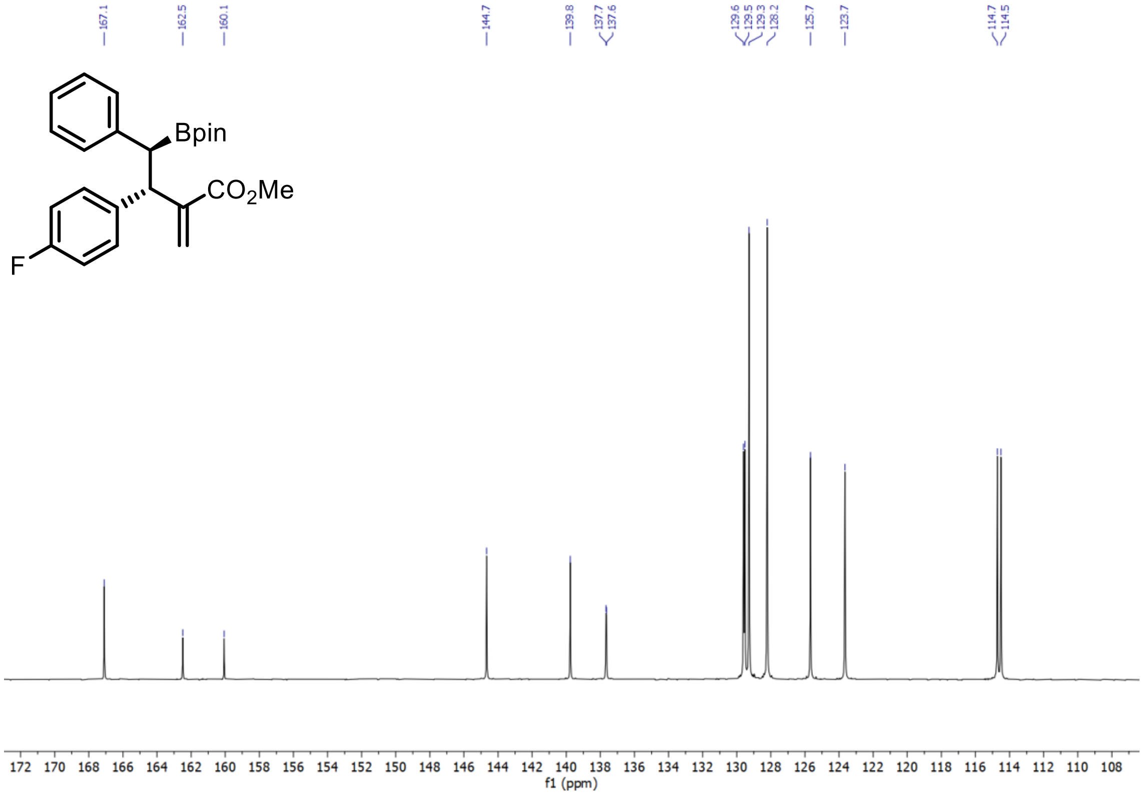

^{13}C NMR (101 MHz, $CDCl_3$) spectra of **3m**.

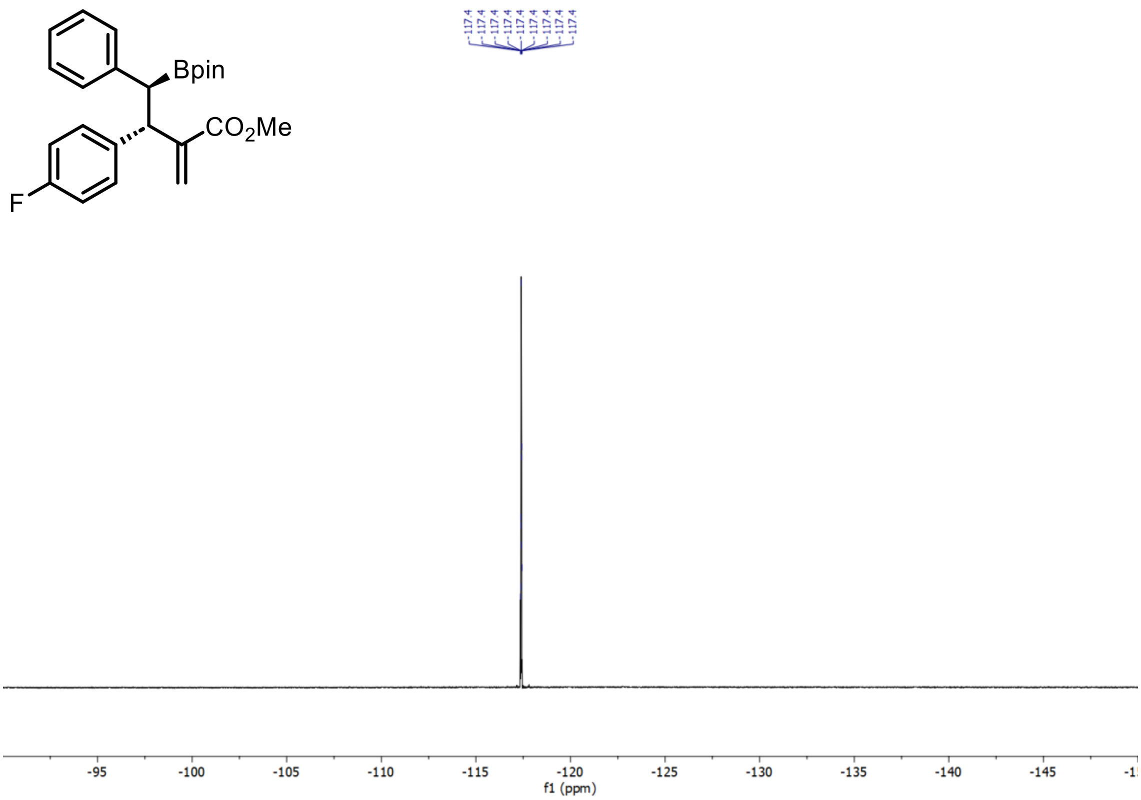

^{19}F NMR (376 MHz, $CDCl_3$) spectra of **3m**.

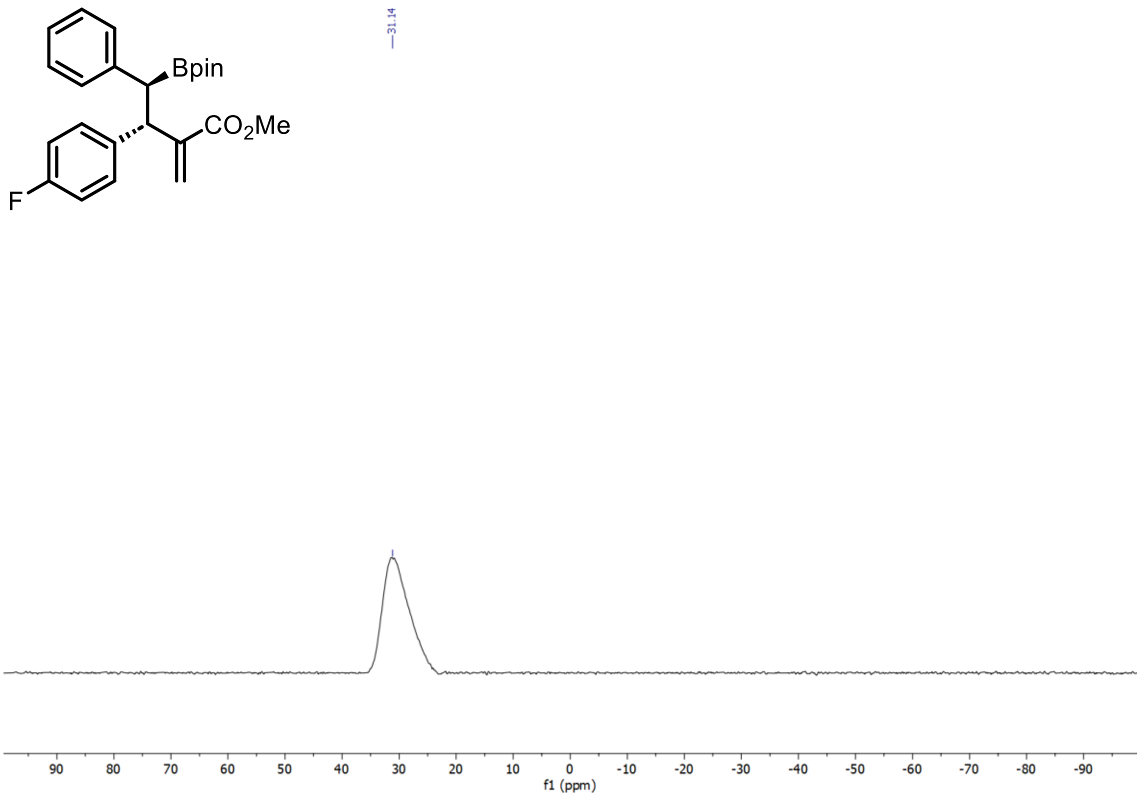

^{11}B NMR (128 MHz, $CDCl_3$) spectra of **3m**.

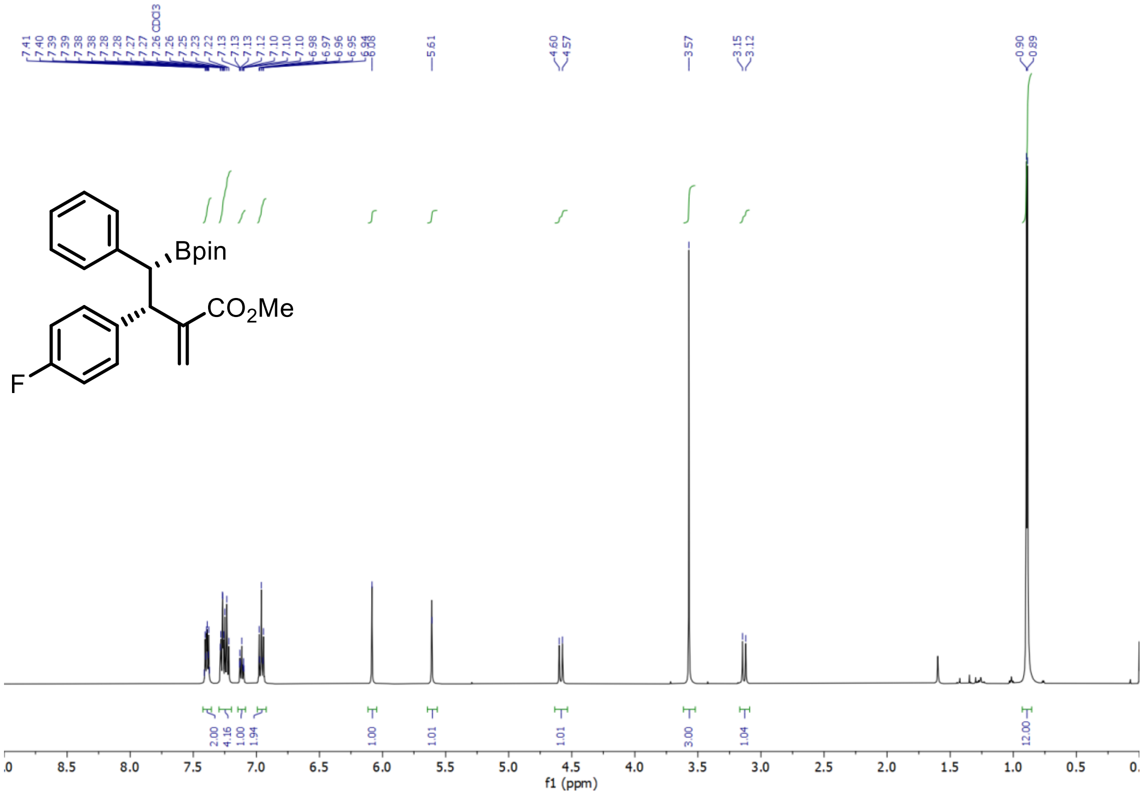

^{1}H NMR (500 MHz, $CDCl_3$) spectra of **3m’**.

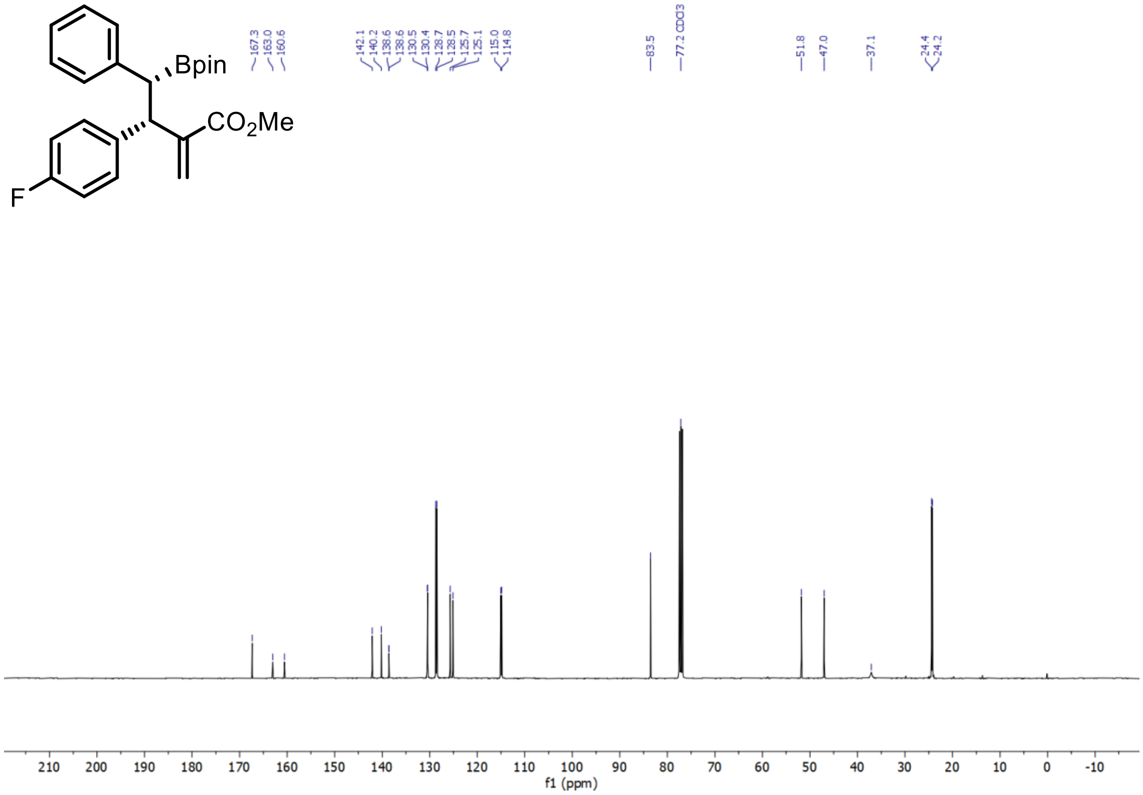

^{13}C NMR (101 MHz, $CDCl_3$) spectra of **3m’**.

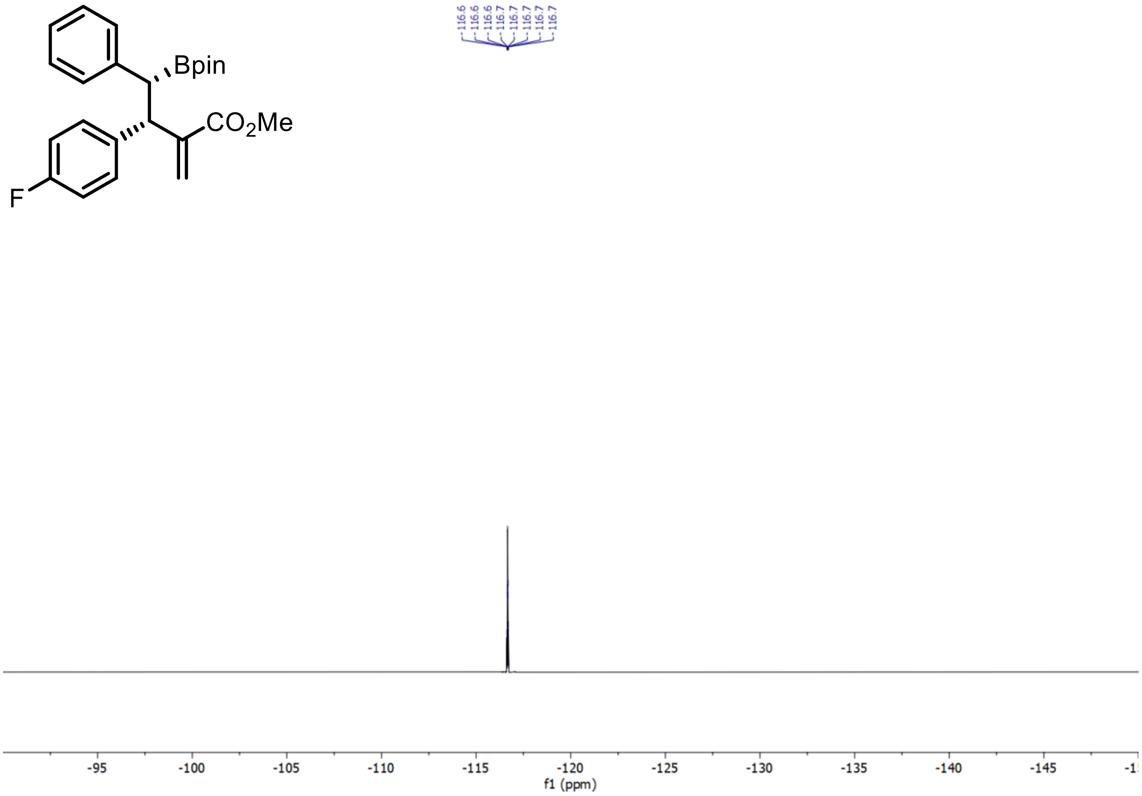

^{19}F NMR (376 MHz, $CDCl_3$) spectra of **3m'**.

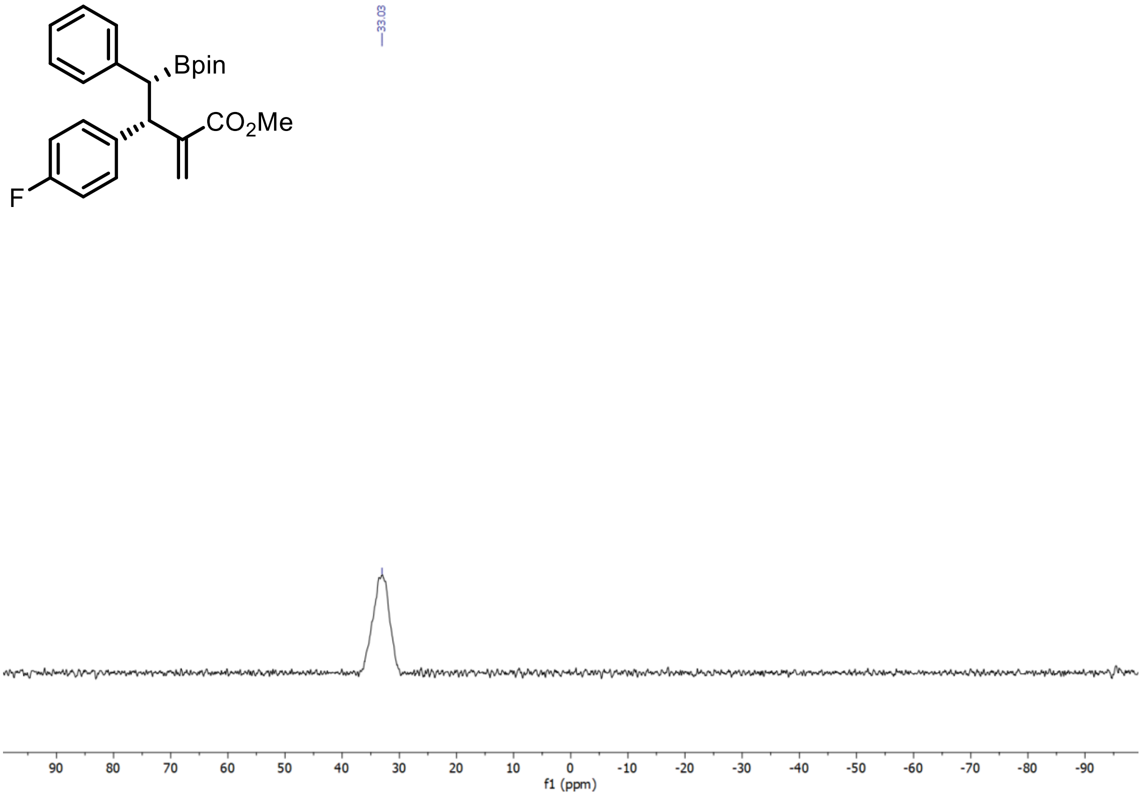

^{11}B NMR (128 MHz, $CDCl_3$) spectra of **3m'**.

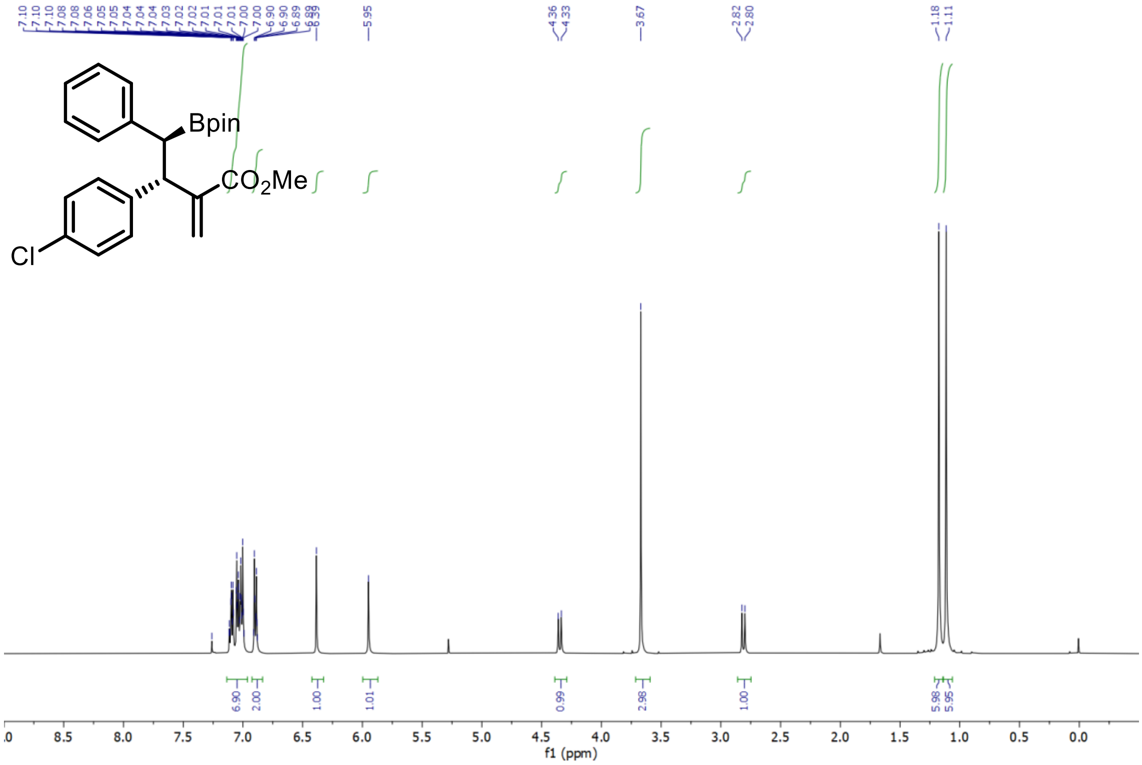

^{1}H NMR (500 MHz, $CDCl_3$) spectra of **3n**.

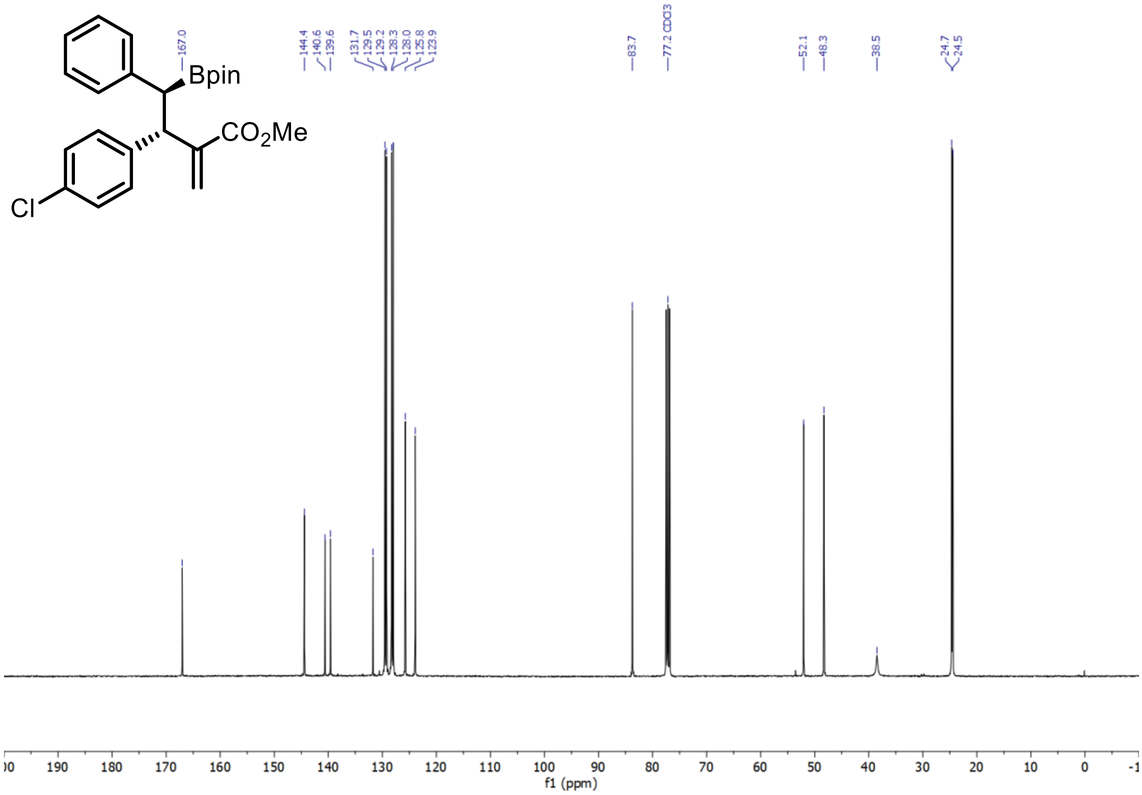

^{13}C NMR (101 MHz, $CDCl_3$) spectra of **3n**.

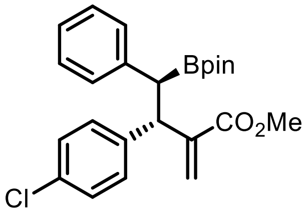

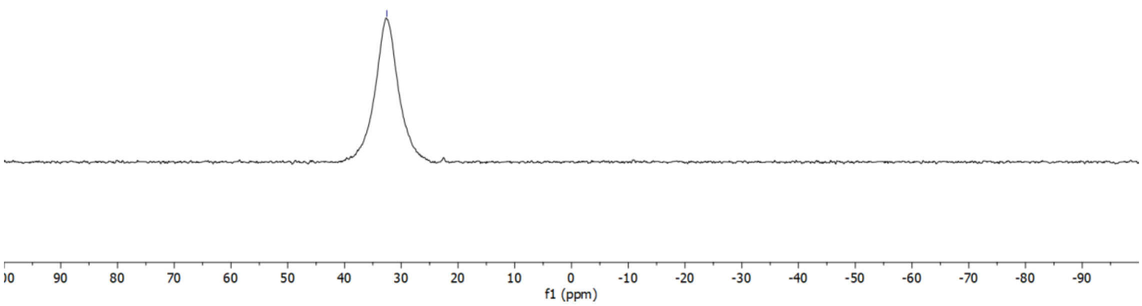

^{11}B NMR (160 MHz, $CDCl_3$) spectra of **3n**.

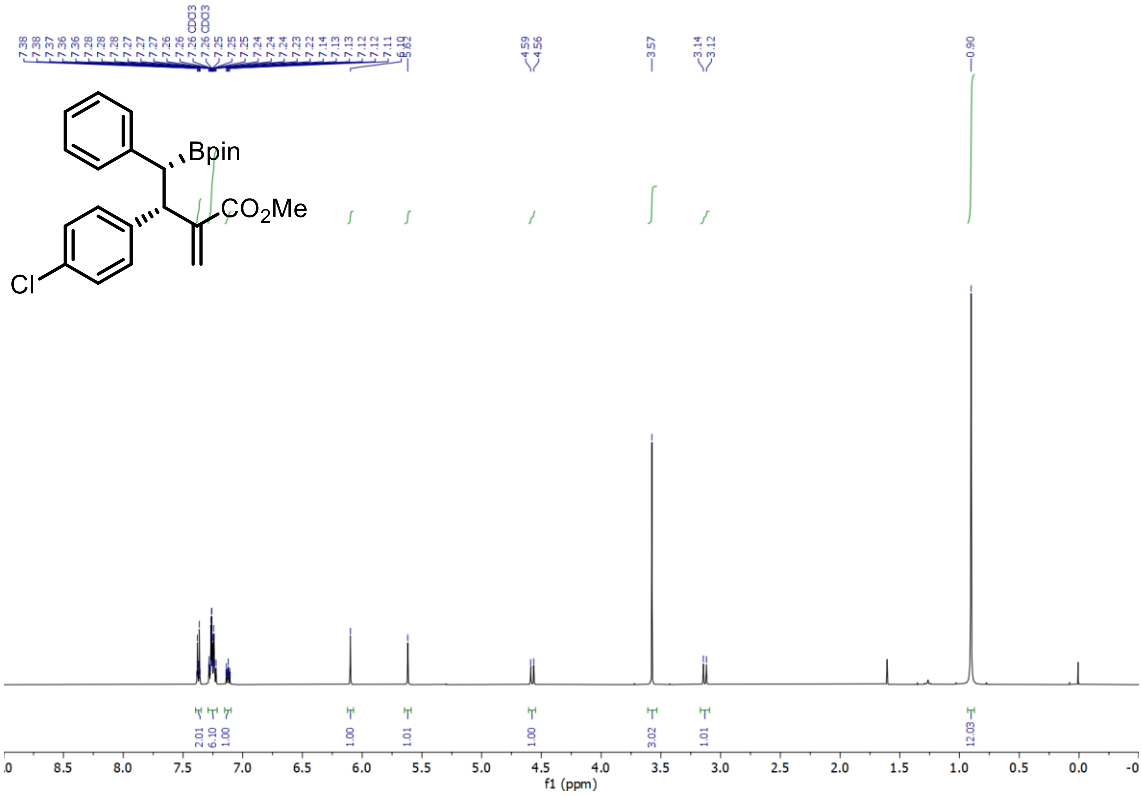

^{1}H NMR (500 MHz, $CDCl_3$) spectra of **3n’**.

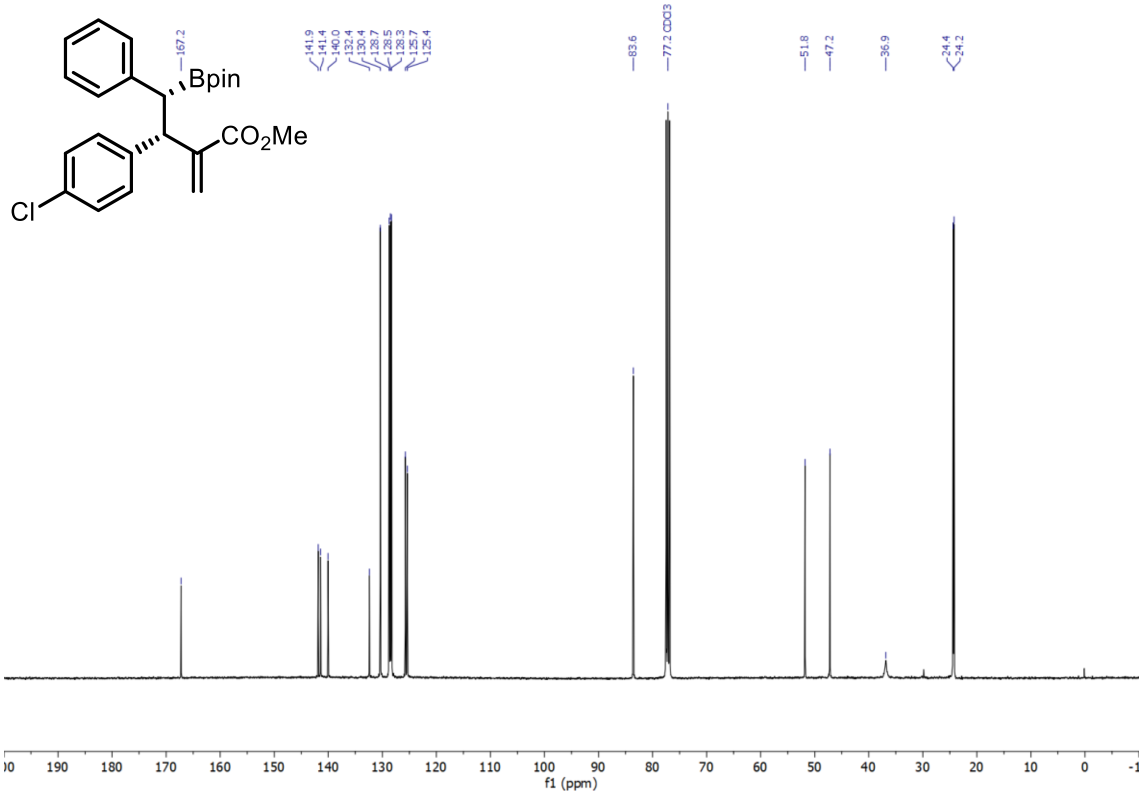

^{13}C NMR (101 MHz, $CDCl_3$) spectra of **3n’**.

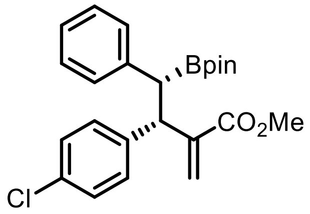

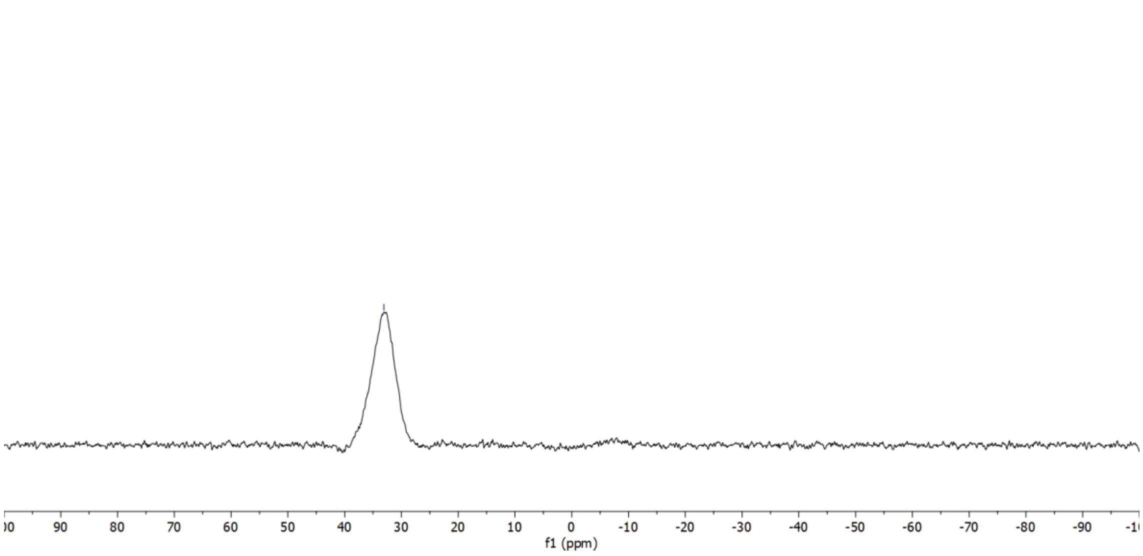

^{11}B NMR (128 MHz, $CDCl_3$) spectra of **3n'**.

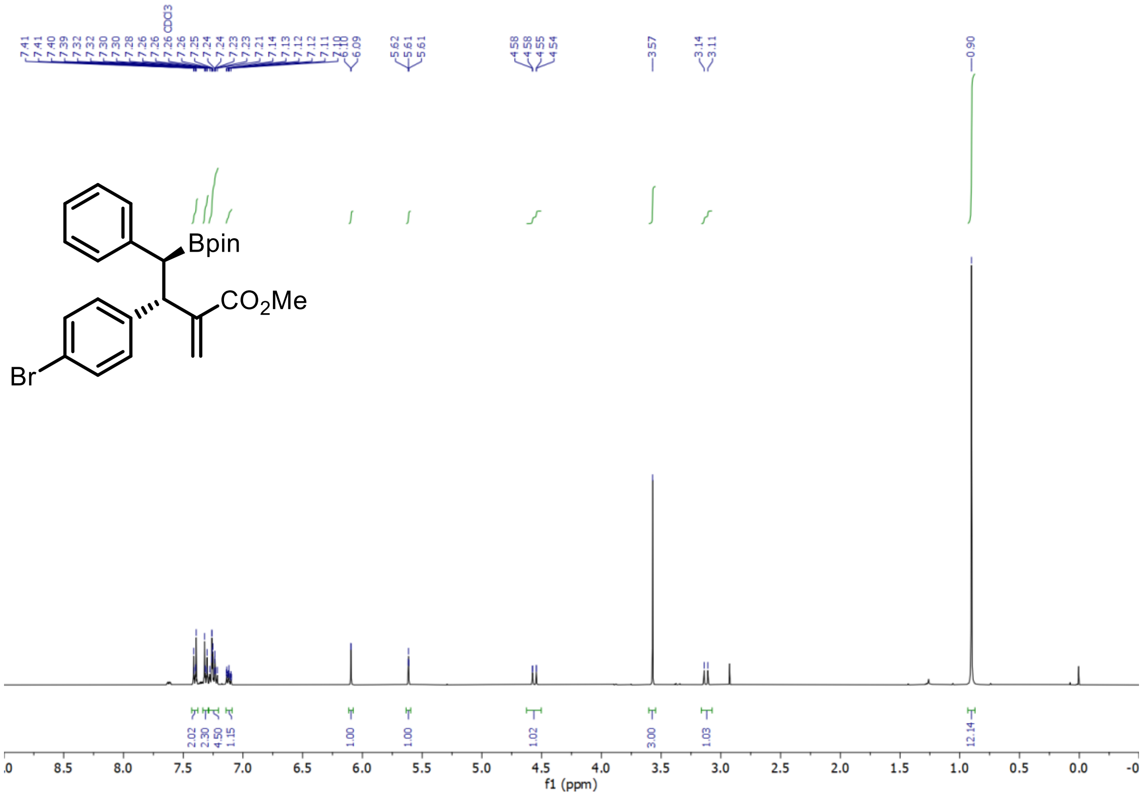

^{1}H NMR (400 MHz, $CDCl_3$) spectra of **3o**.

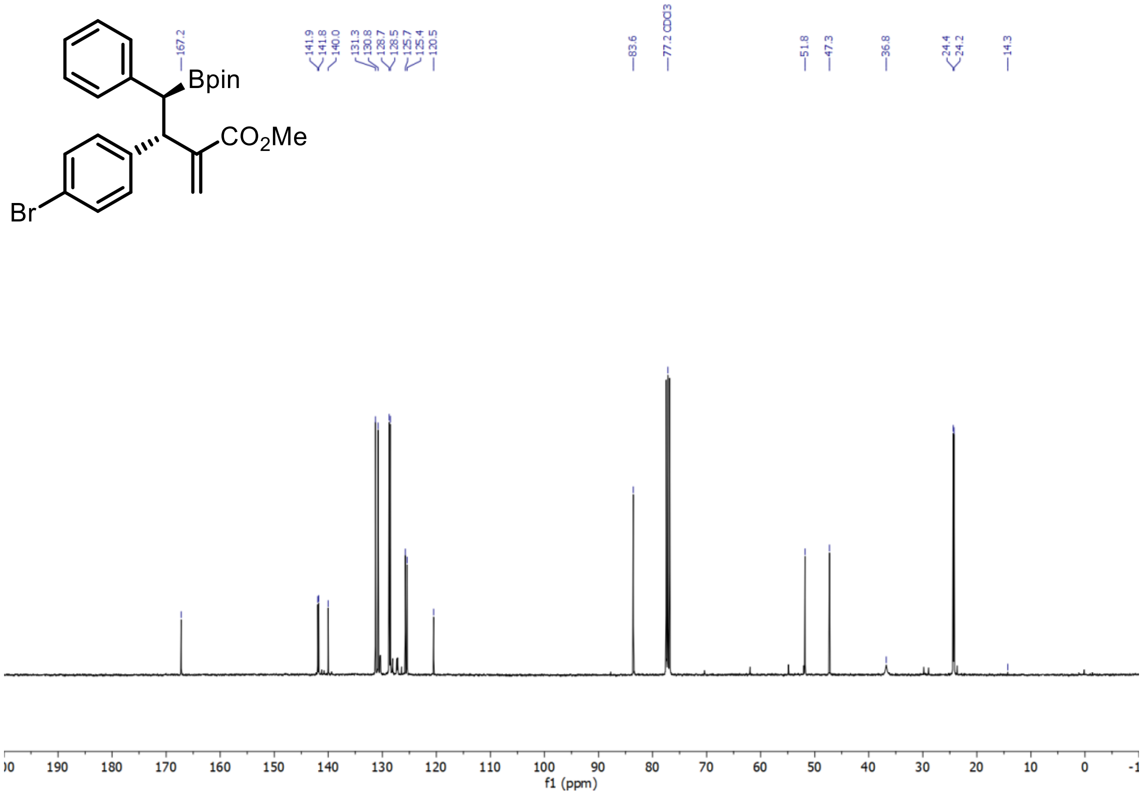

^{13}C NMR (101 MHz, $CDCl_3$) spectra of **3o**.

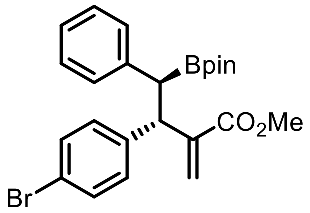

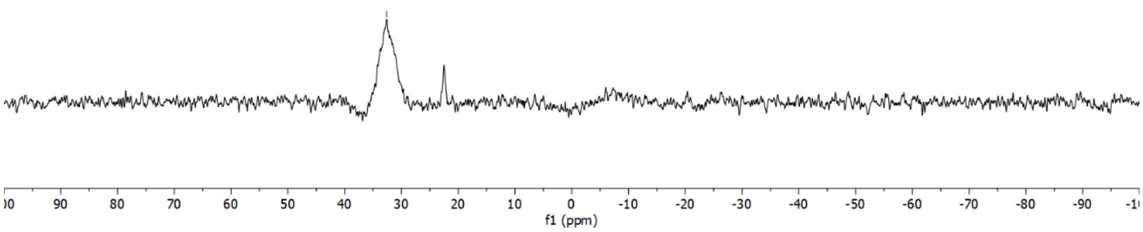

^{11}B NMR (128 MHz, $CDCl_3$) spectra of **3o**.

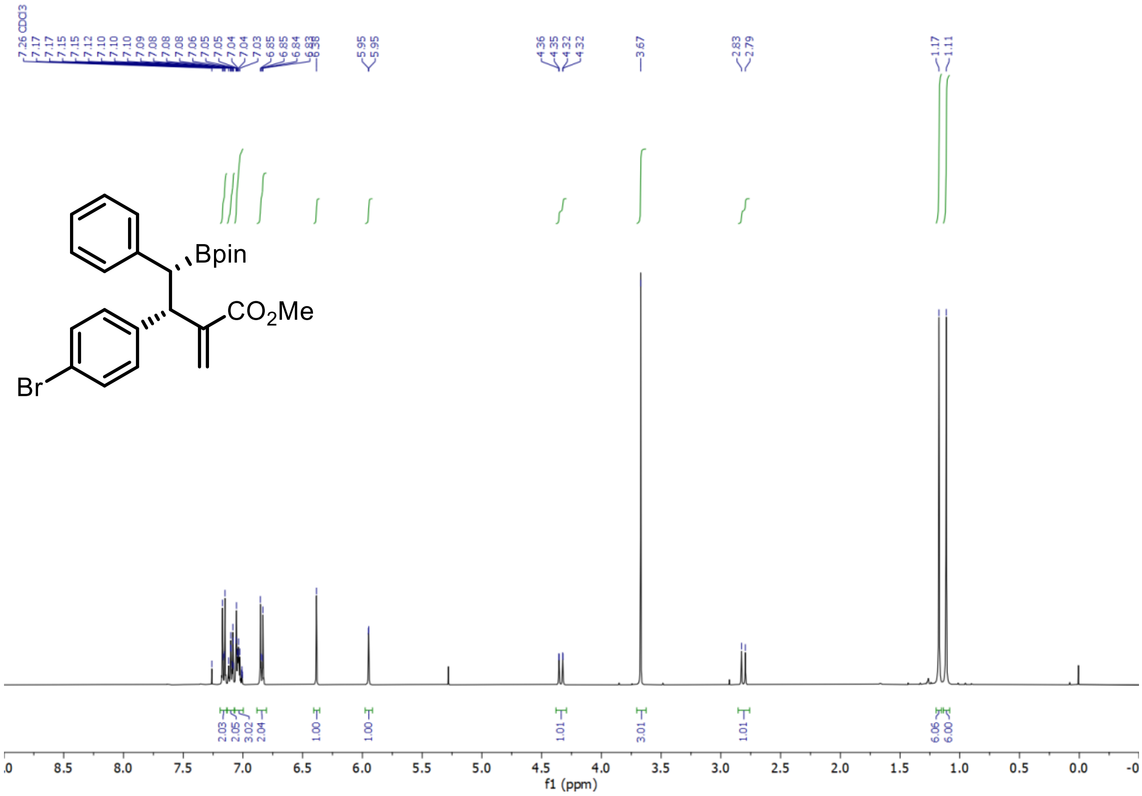

^{1}H NMR (400 MHz, $CDCl_3$) spectra of **3o'**.

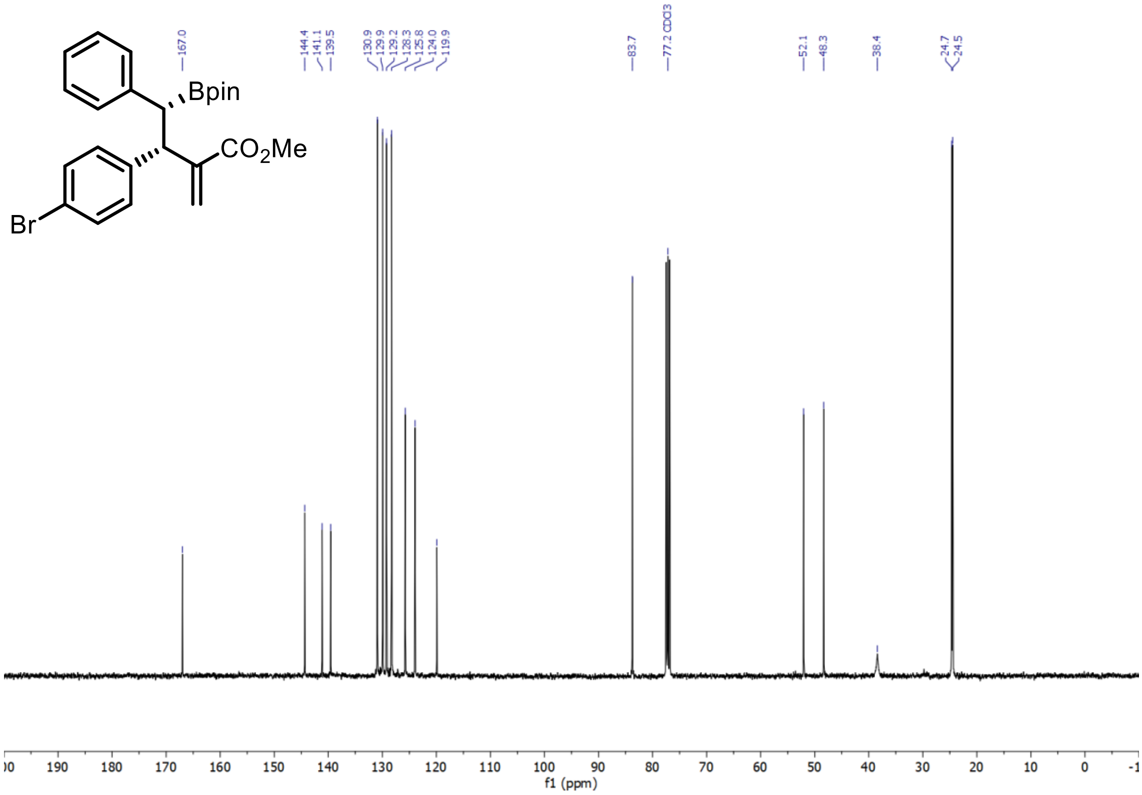

^{13}C NMR (101 MHz, $CDCl_3$) spectra of **3o'**.

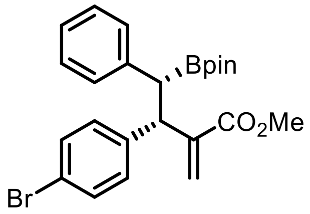

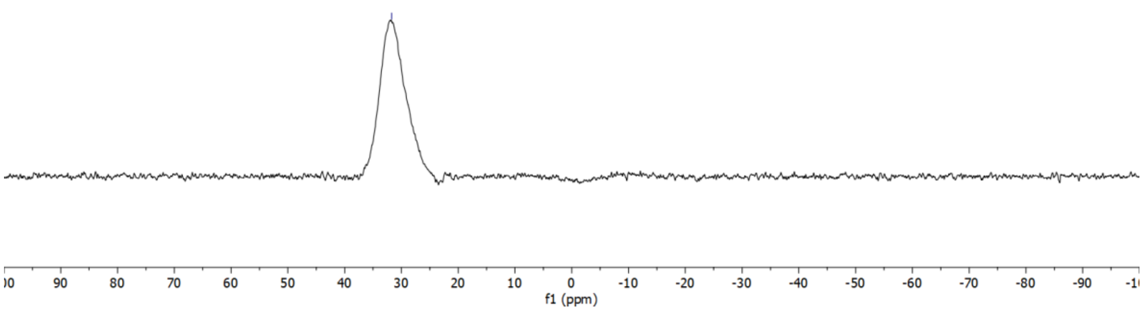

^{11}B NMR (128 MHz, $CDCl_3$) spectra of **3o’**.

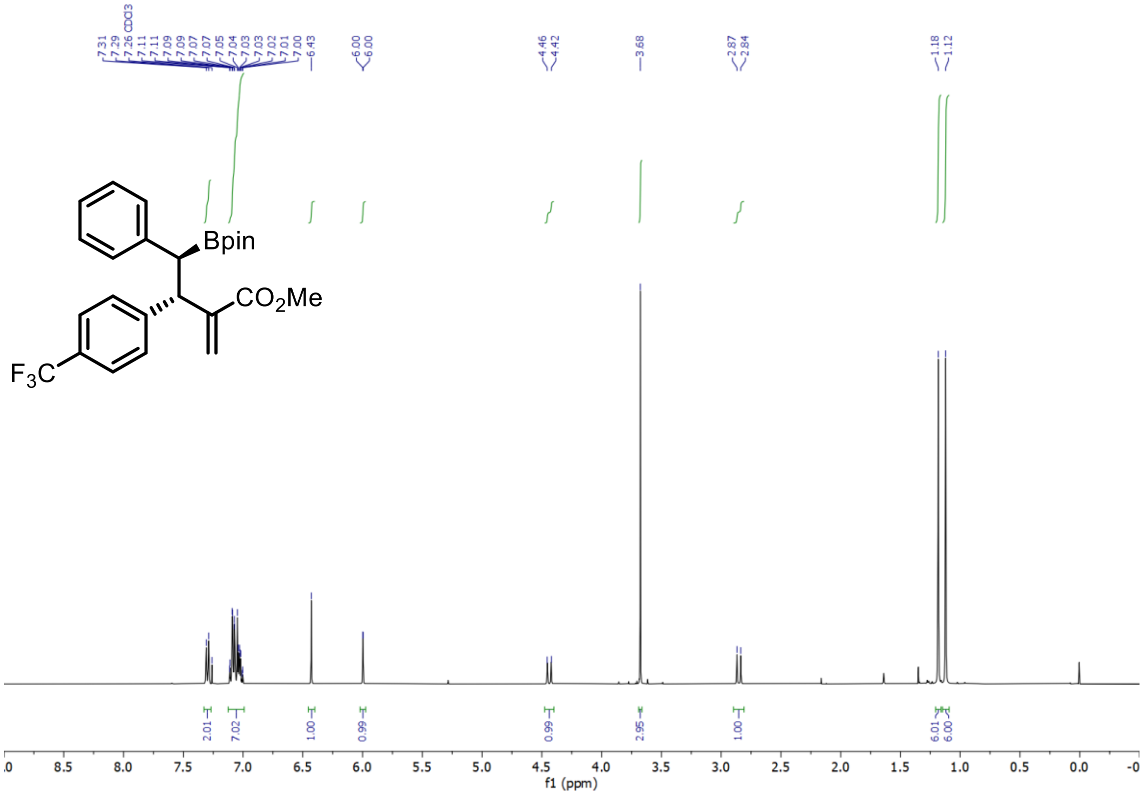

^{1}H NMR (400 MHz, $CDCl_3$) spectra of **3p**.

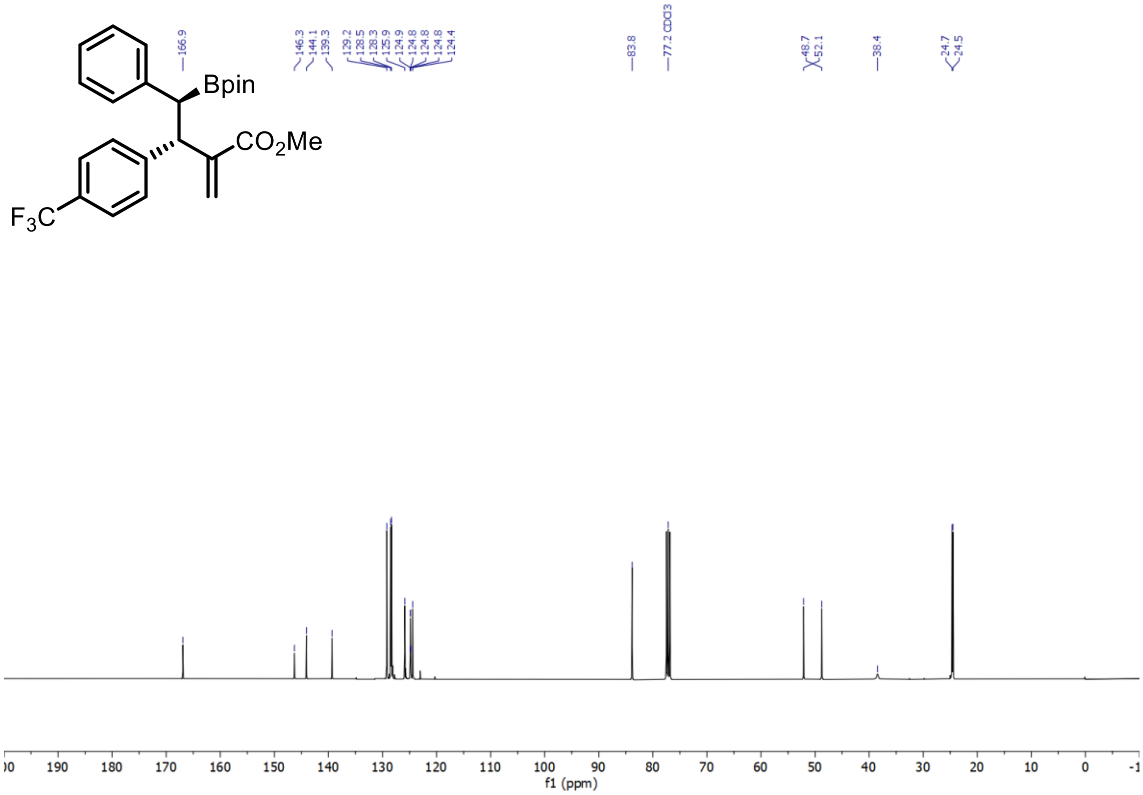

^{13}C NMR (101 MHz, $CDCl_3$) spectra of **3p**.

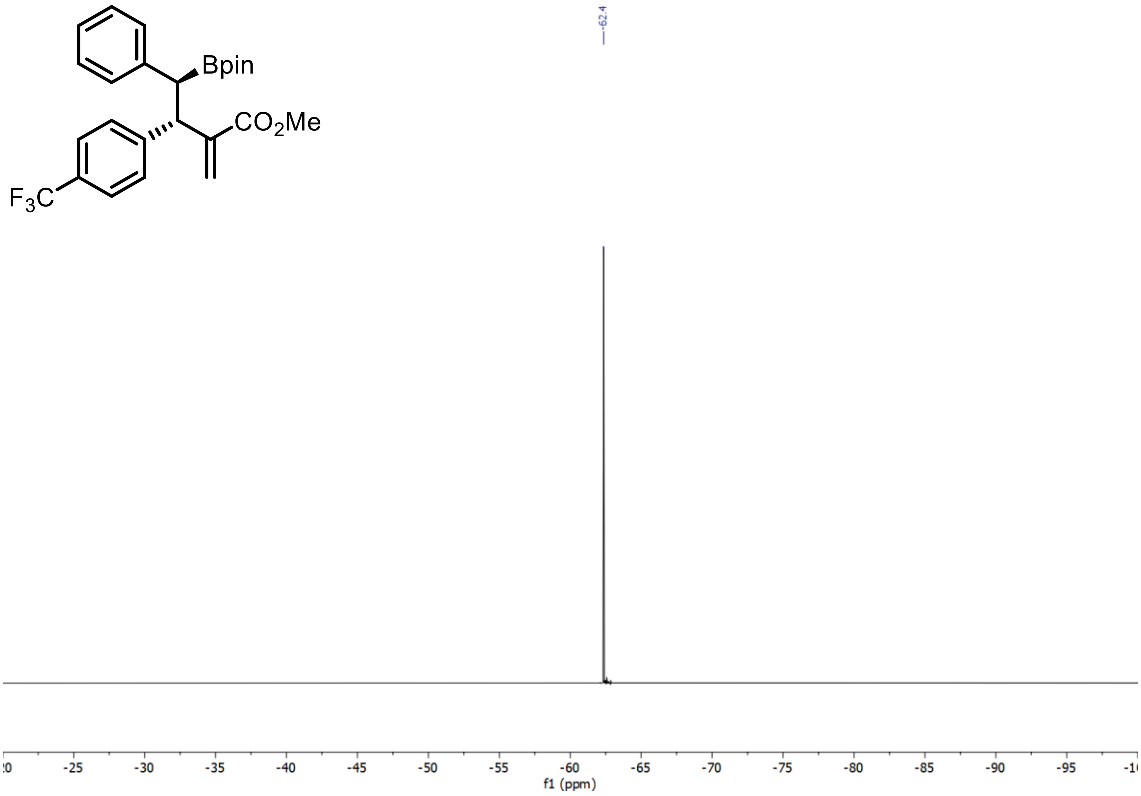

^{19}F NMR (376 MHz, CDCl$_3$) spectra of **3p**.

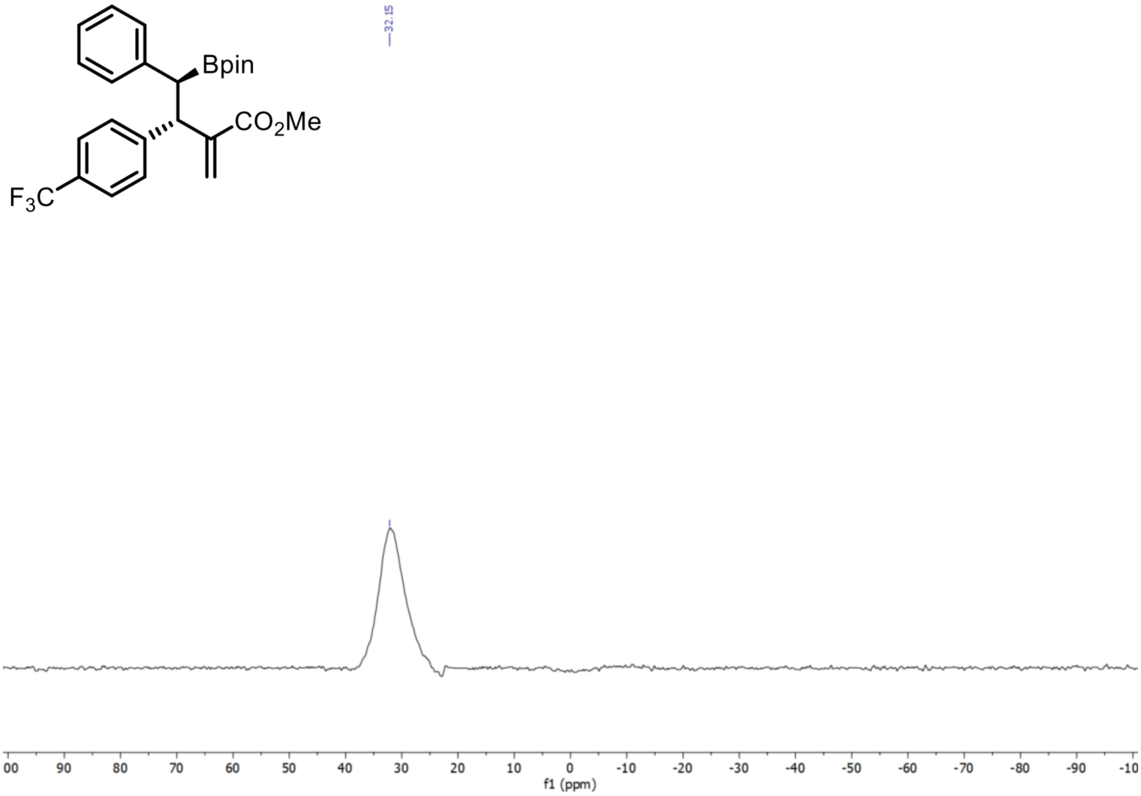

^{11}B NMR (128 MHz, CDCl$_3$) spectra of **3p**.

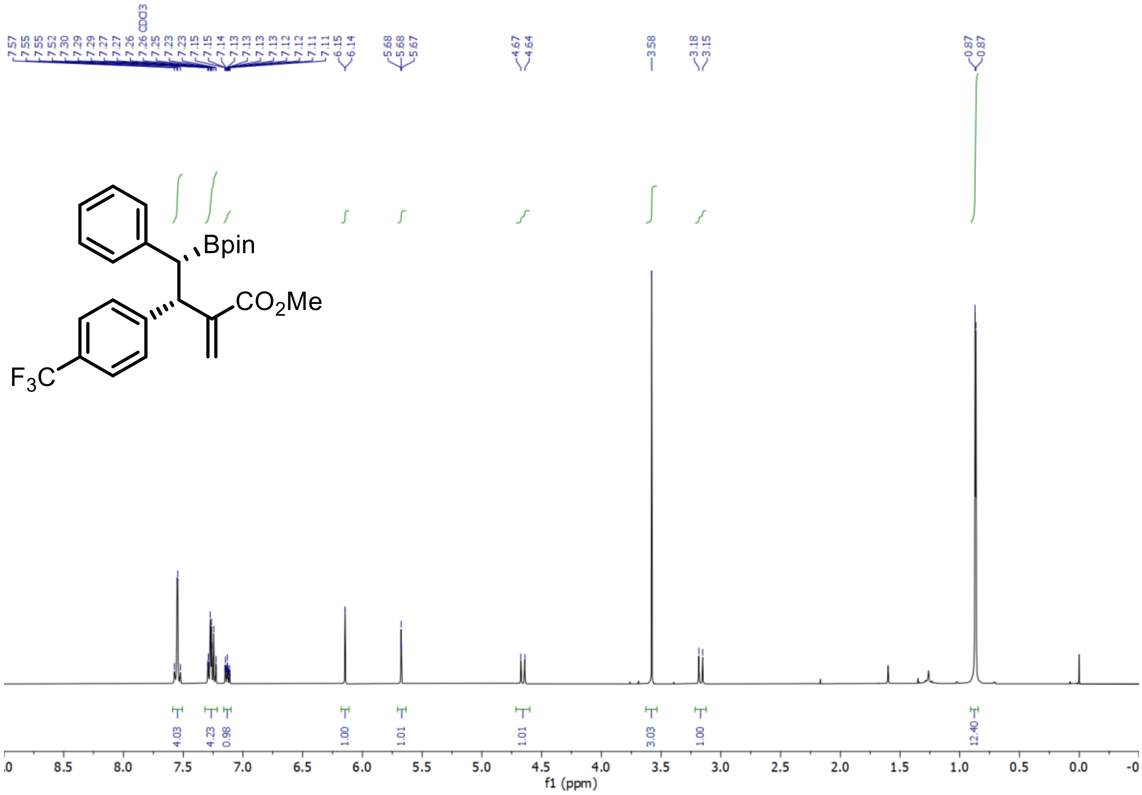

^{1}H NMR (400 MHz, $CDCl_3$) spectra of **3p’**.

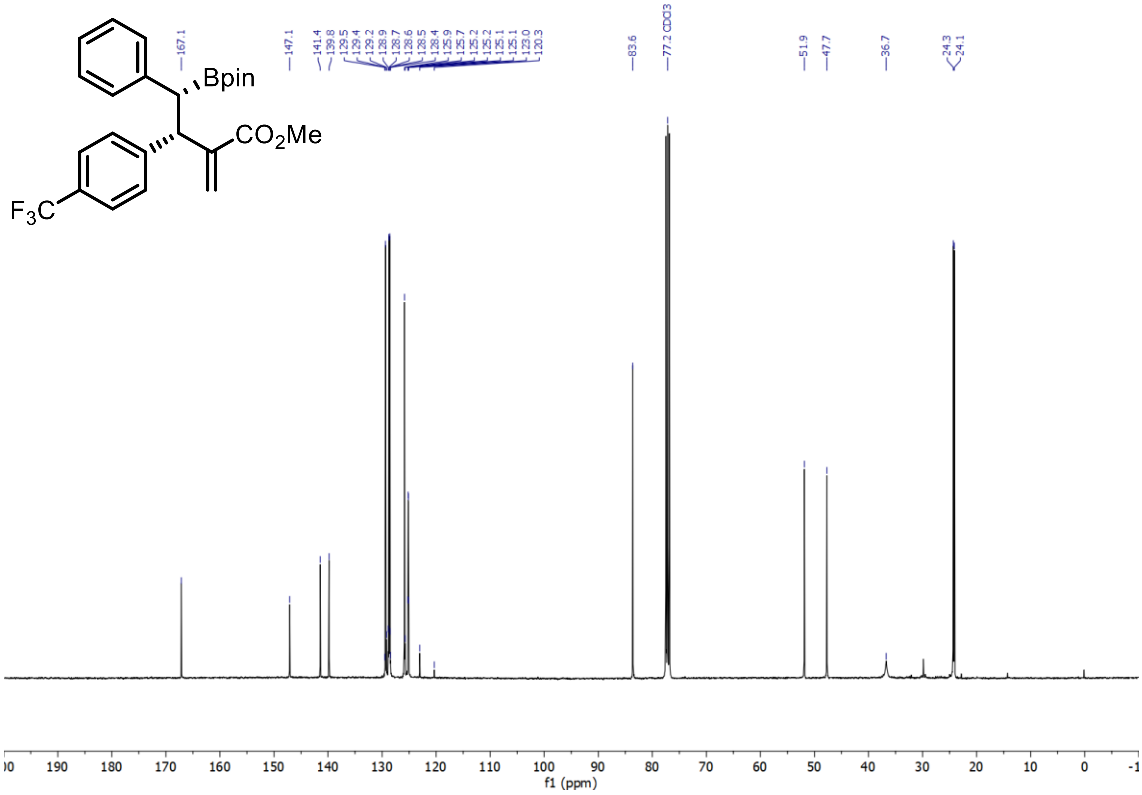

^{13}C NMR (101 MHz, $CDCl_3$) spectra of **3p’**.

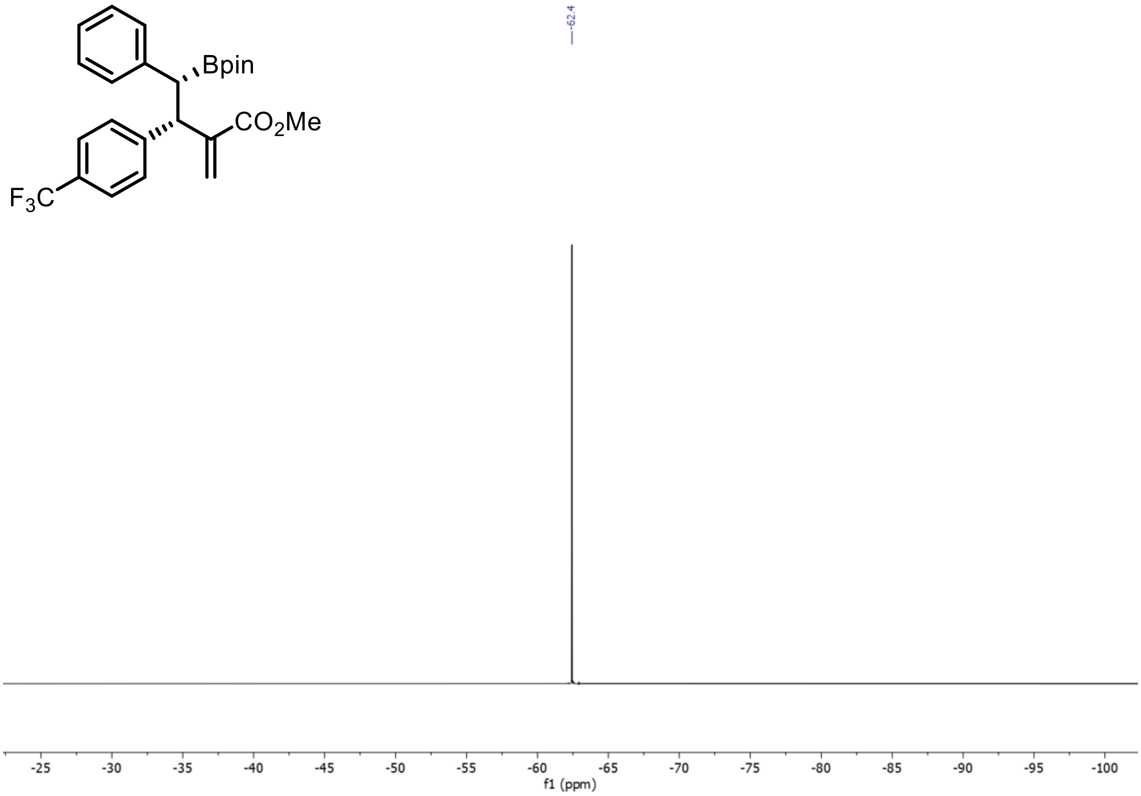

^{19}F NMR (376 MHz, $CDCl_3$) spectra of **3p’**.

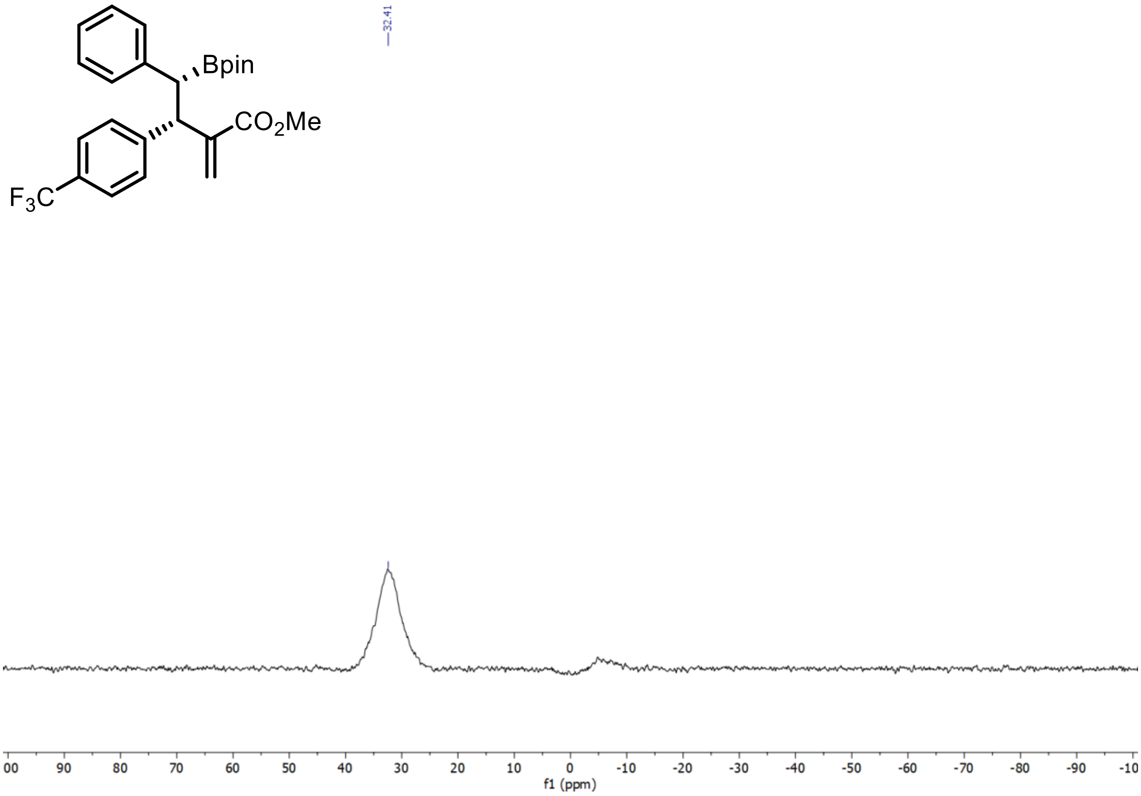

^{11}B NMR (128 MHz, $CDCl_3$) spectra of **3p’**.

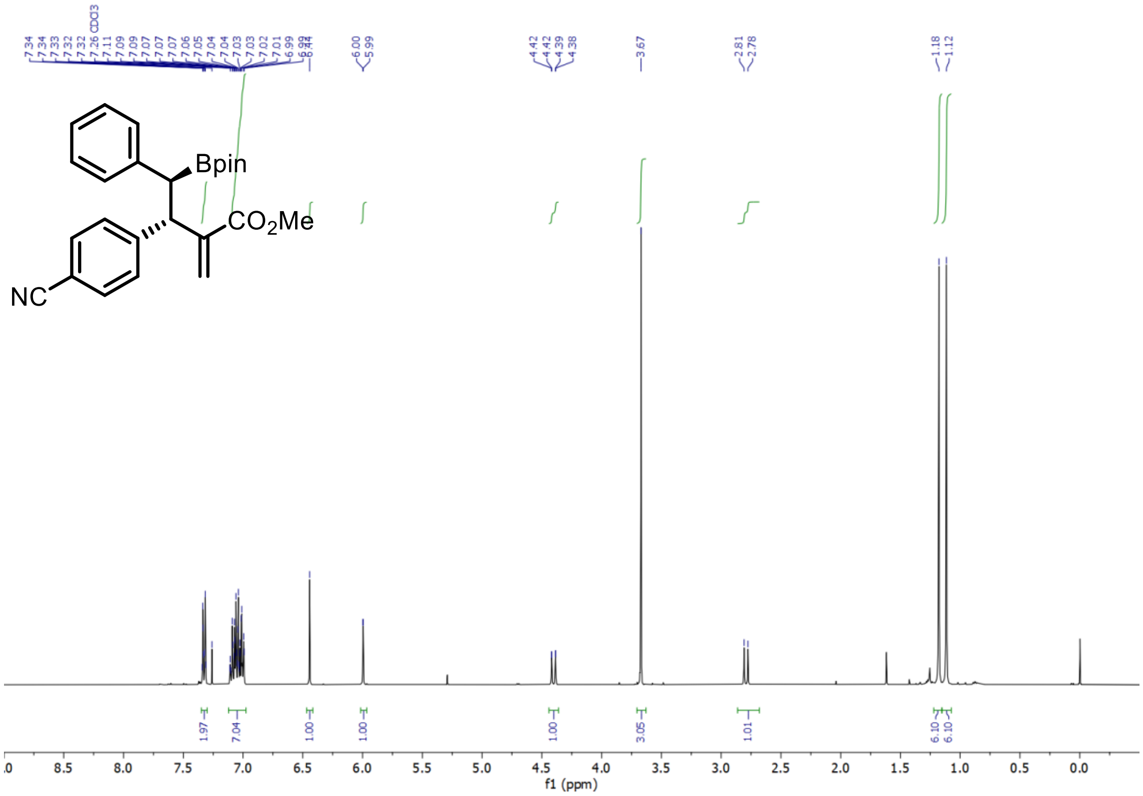

^{1}H NMR (400 MHz, $CDCl_3$) spectra of **3q**.

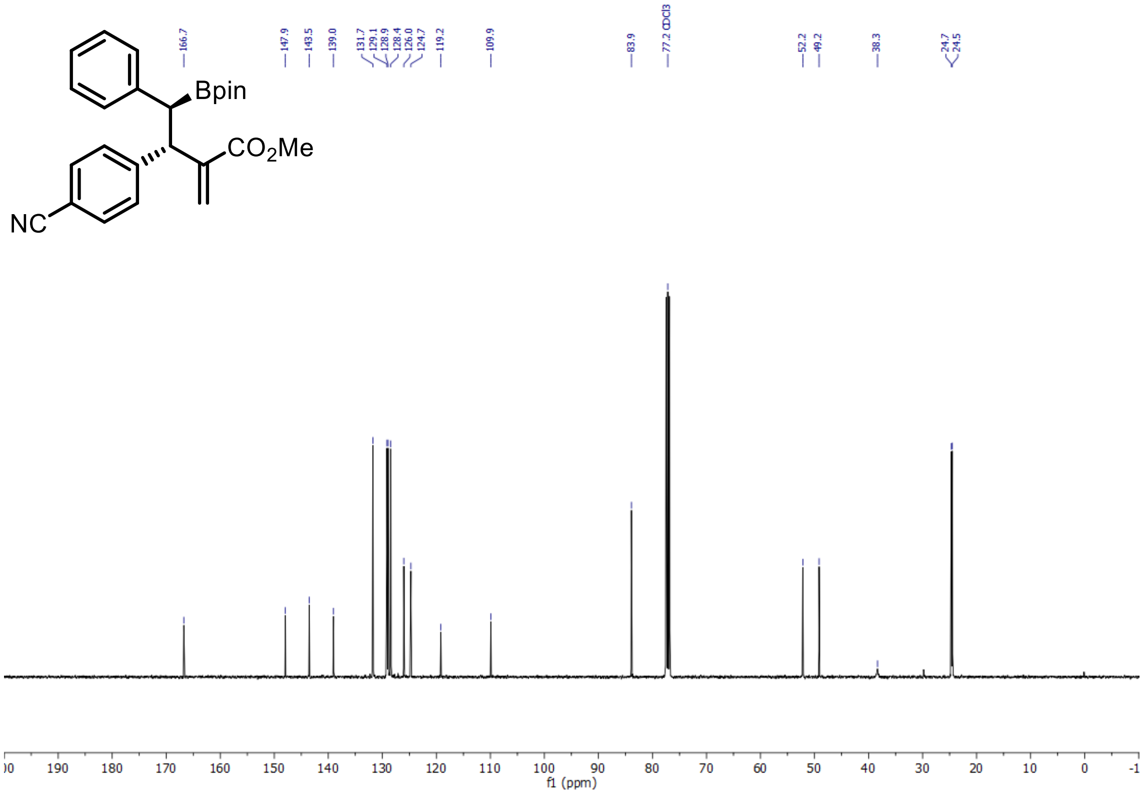

^{13}C NMR (101 MHz, $CDCl_3$) spectra of **3q**.

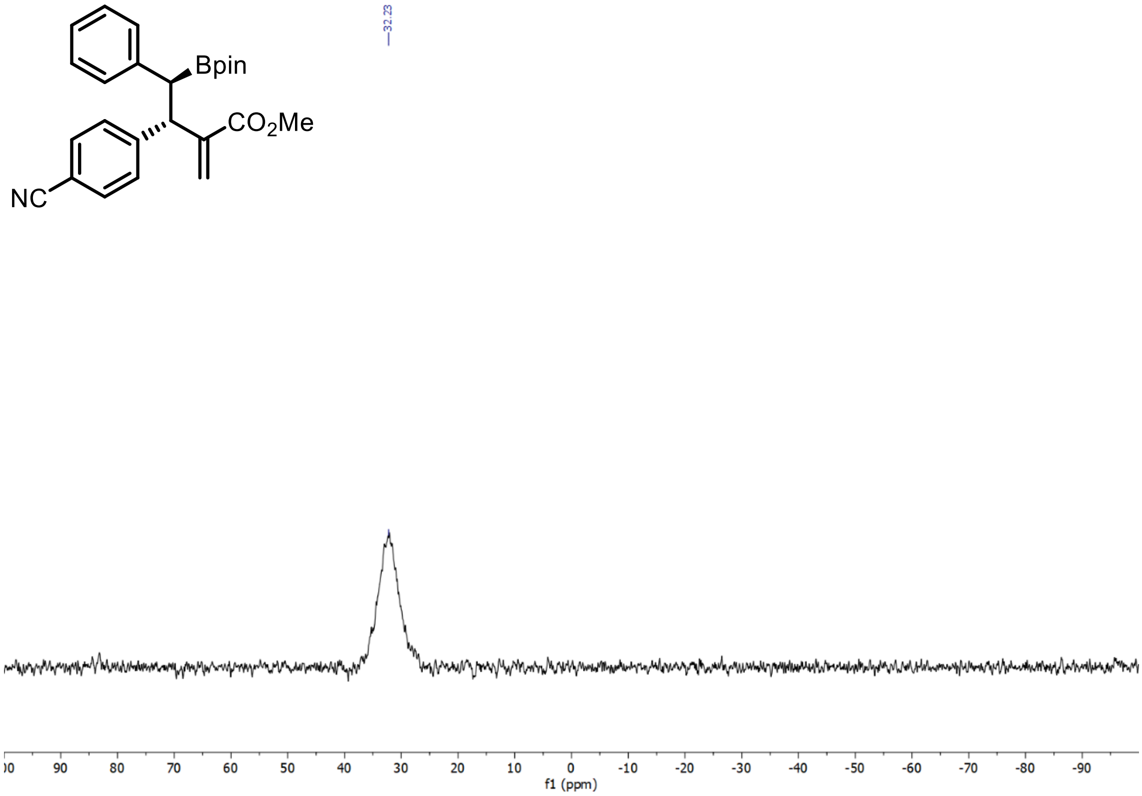

^{11}B NMR (160 MHz, $CDCl_3$) spectra of **3q**.

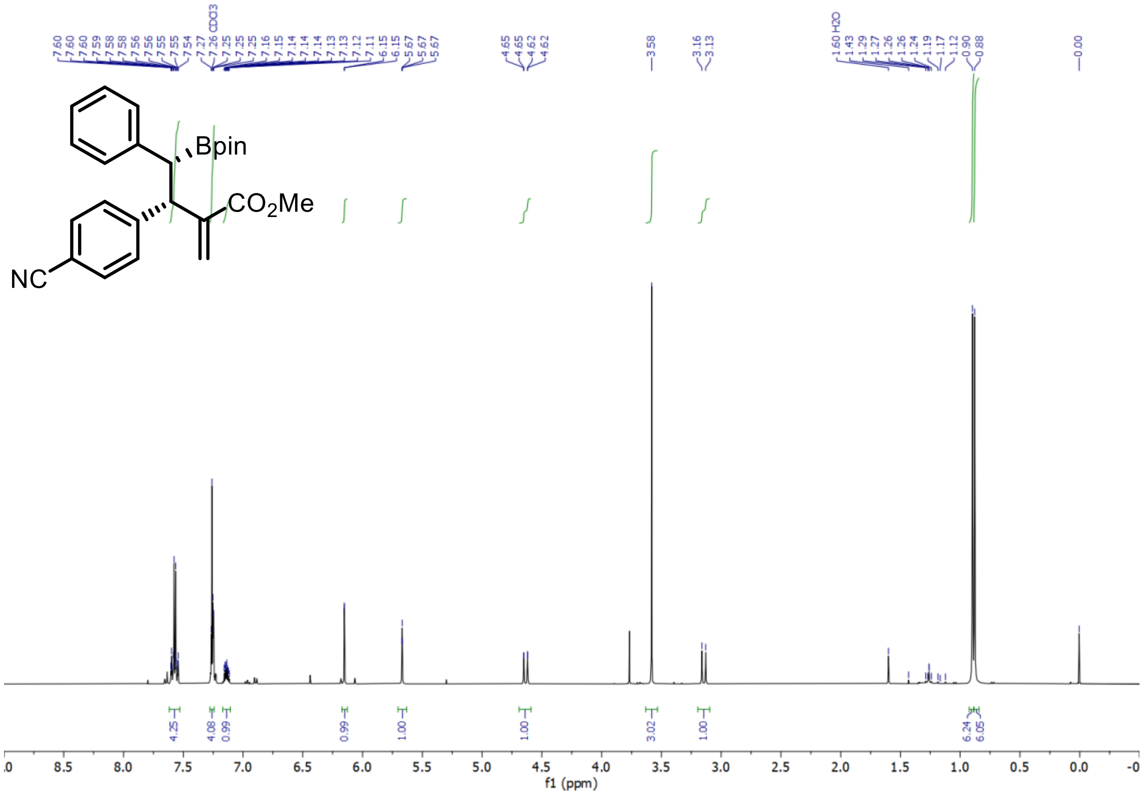

^{1}H NMR (400 MHz, $CDCl_3$) spectra of **3q'**.

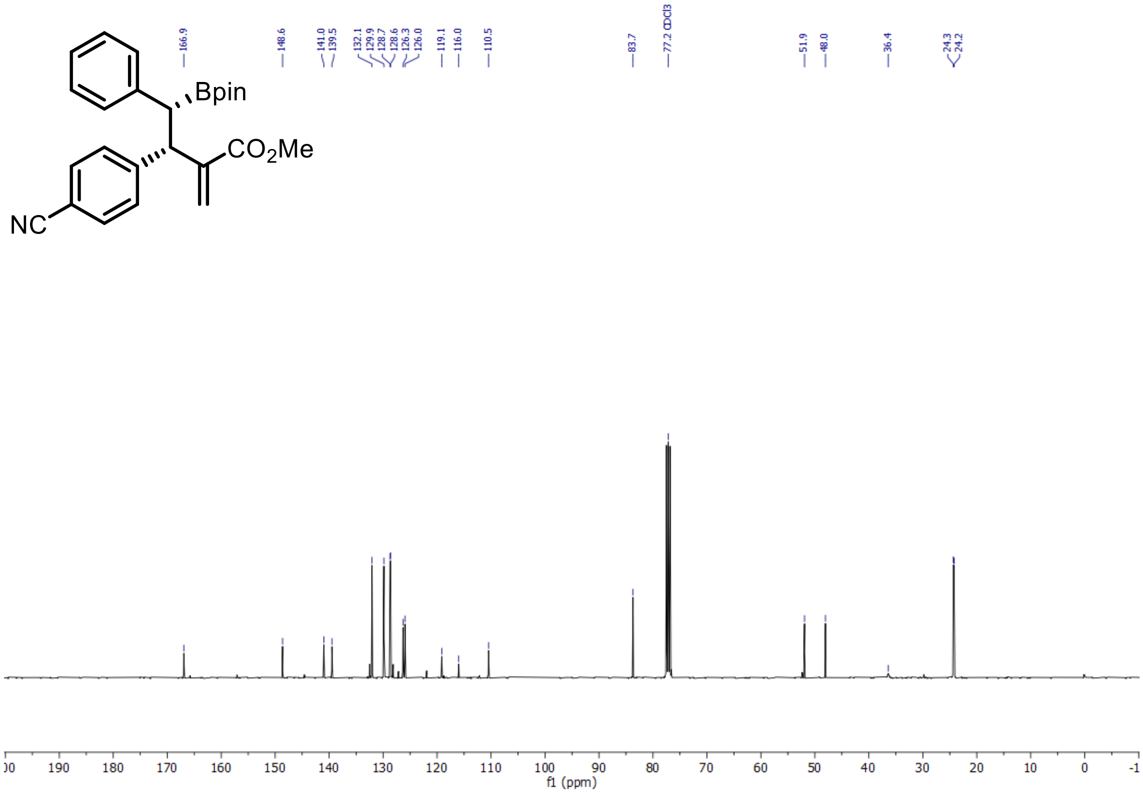

^{13}C NMR (101 MHz, $CDCl_3$) spectra of **3q'**.

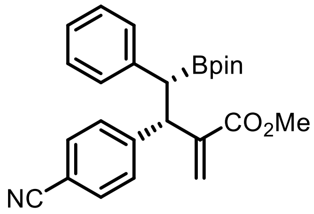

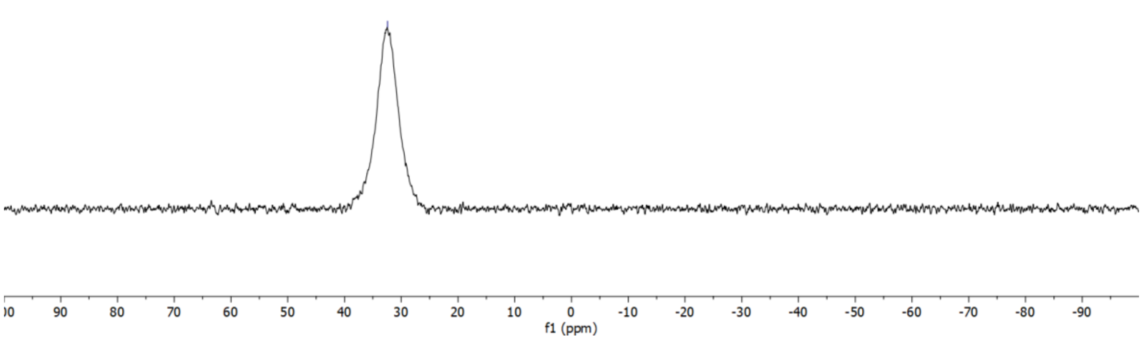

^{11}B NMR (160 MHz, $CDCl_3$) spectra of **3q'**.

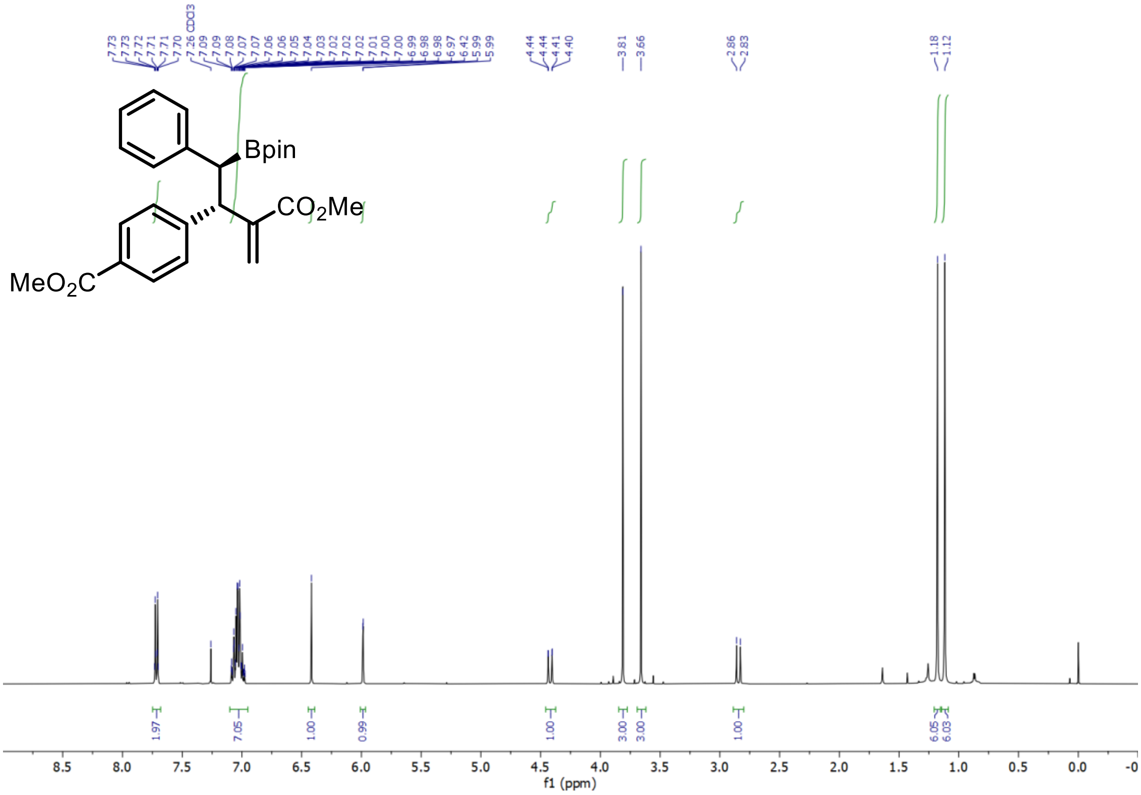

^{1}H NMR (400 MHz, $CDCl_3$) spectra of **3r**.

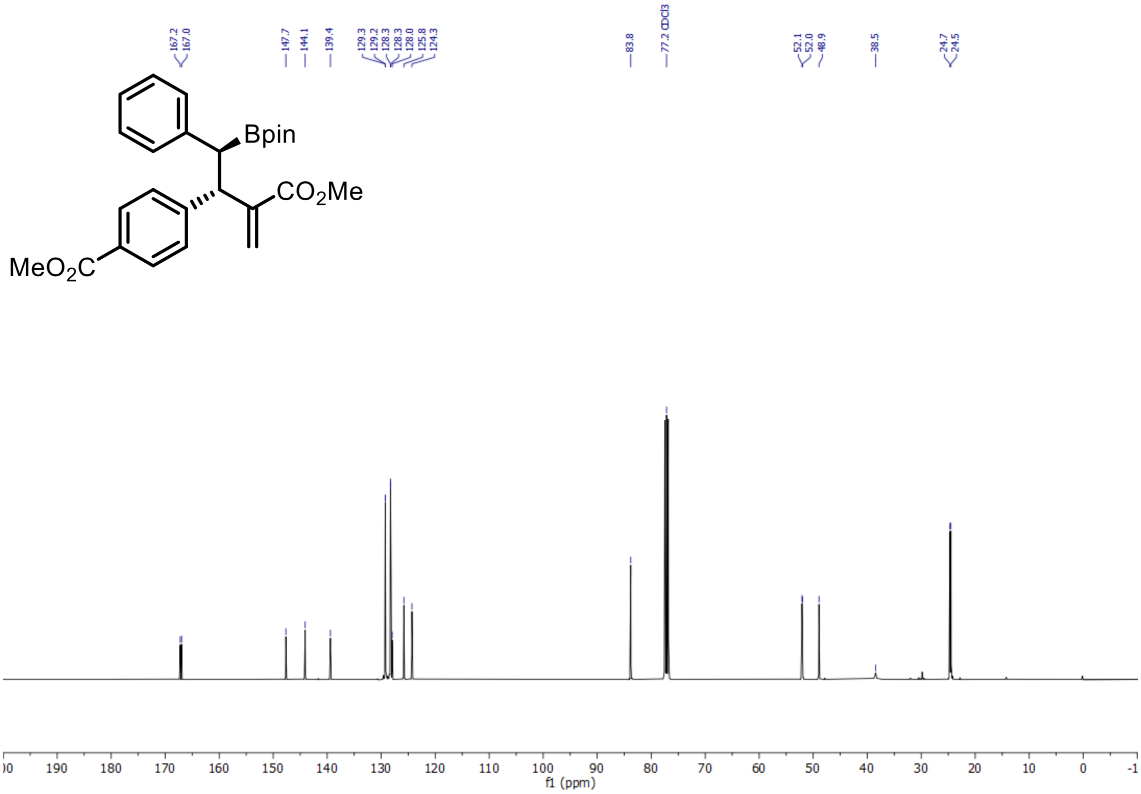

^{13}C NMR (101 MHz, $CDCl_3$) spectra of **3r**.

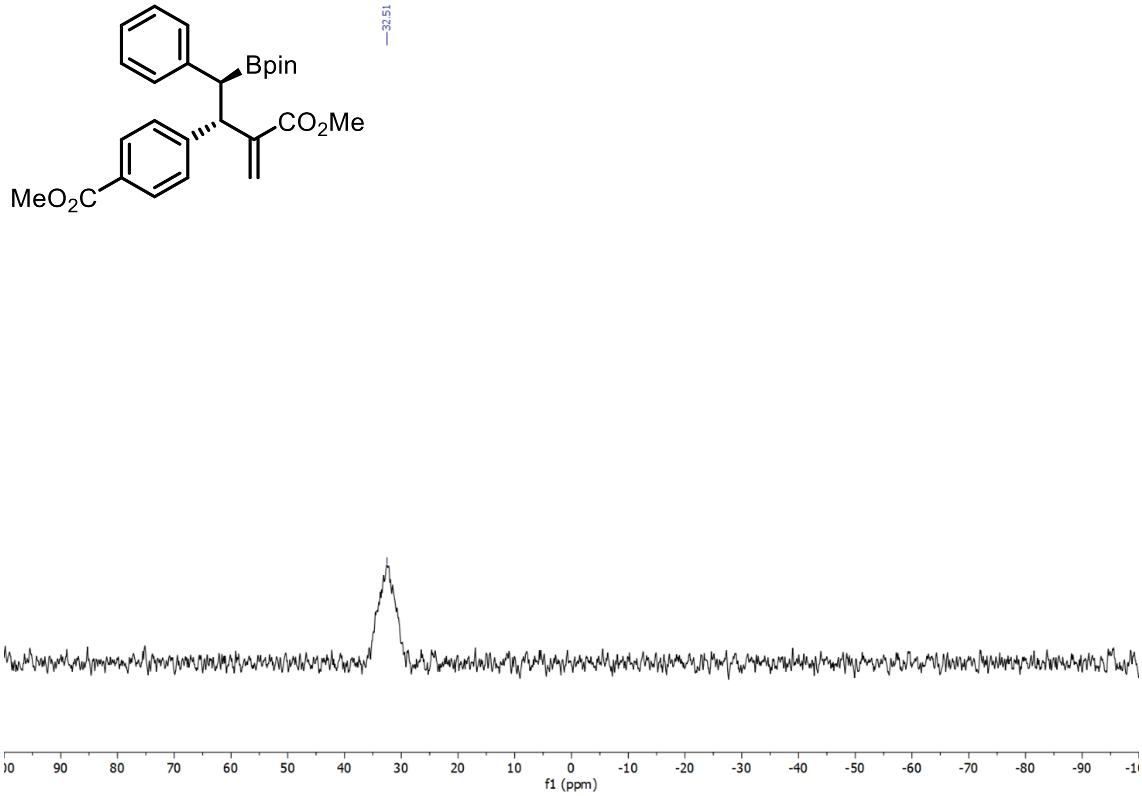

^{11}B NMR (128 MHz, $CDCl_3$) spectra of **3r**.

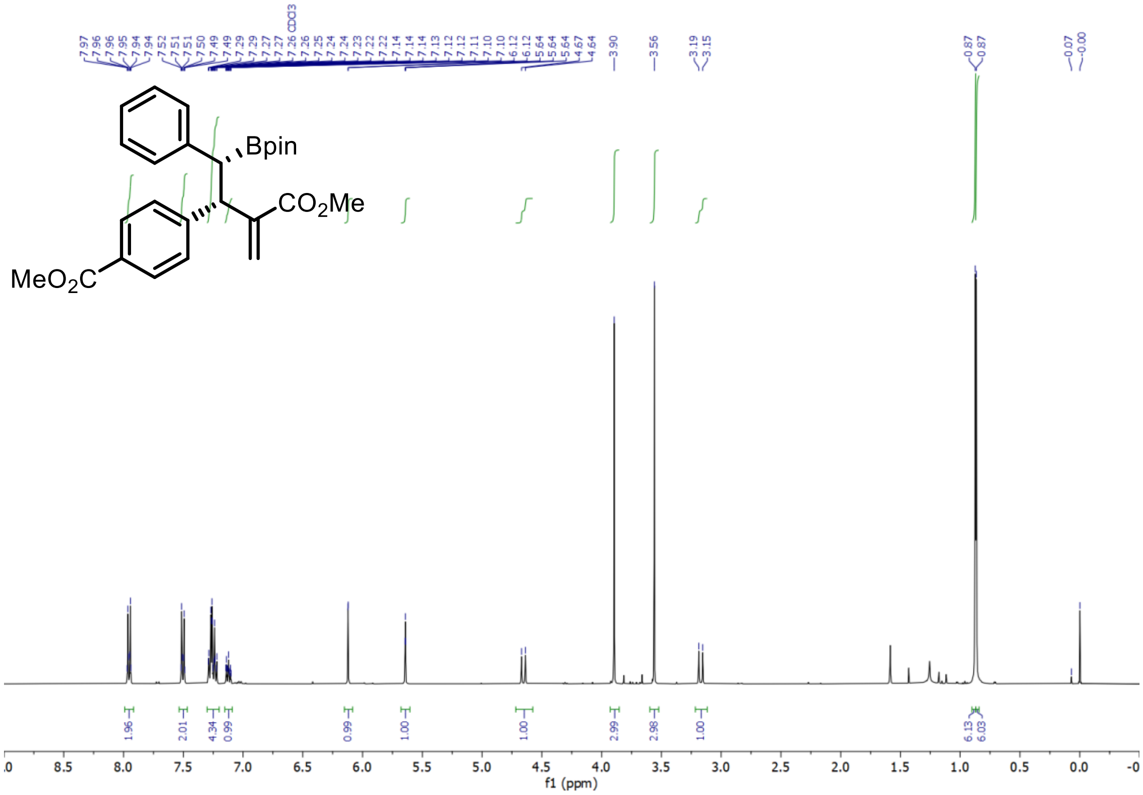

^{1}H NMR (400 MHz, $CDCl_3$) spectra of **3r’**.

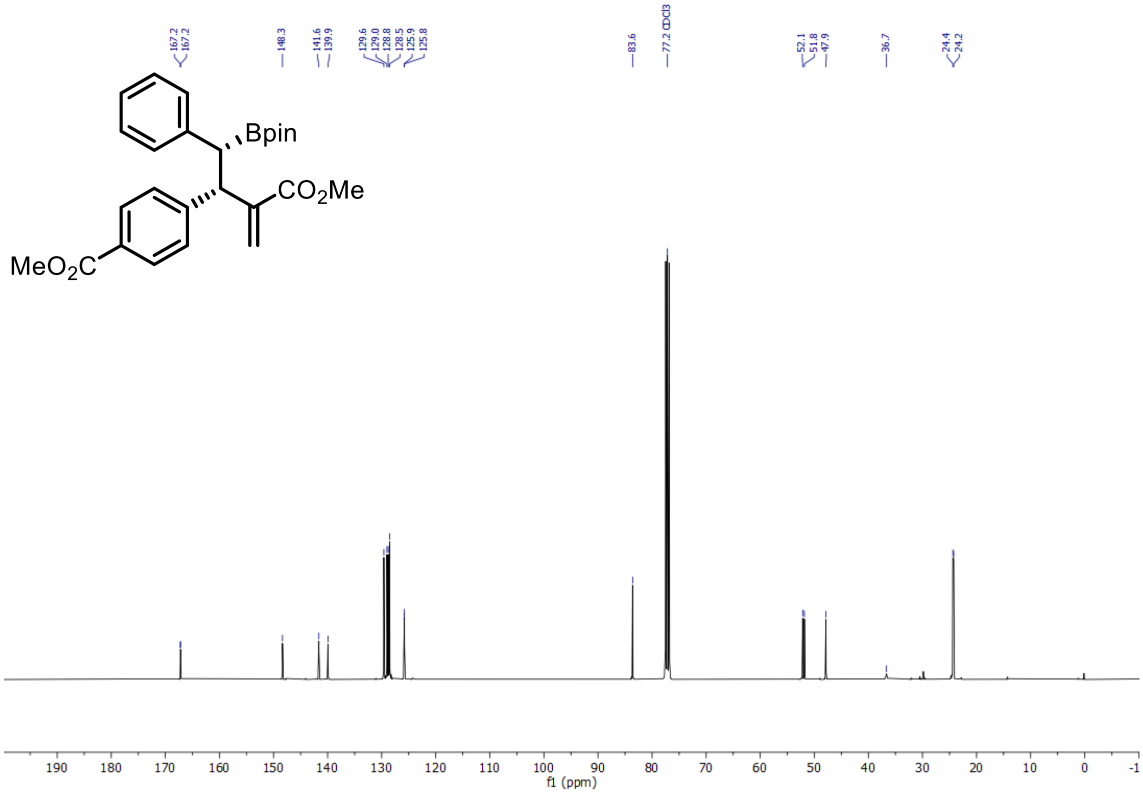

^{13}C NMR (101 MHz, $CDCl_3$) spectra of **3r’**.

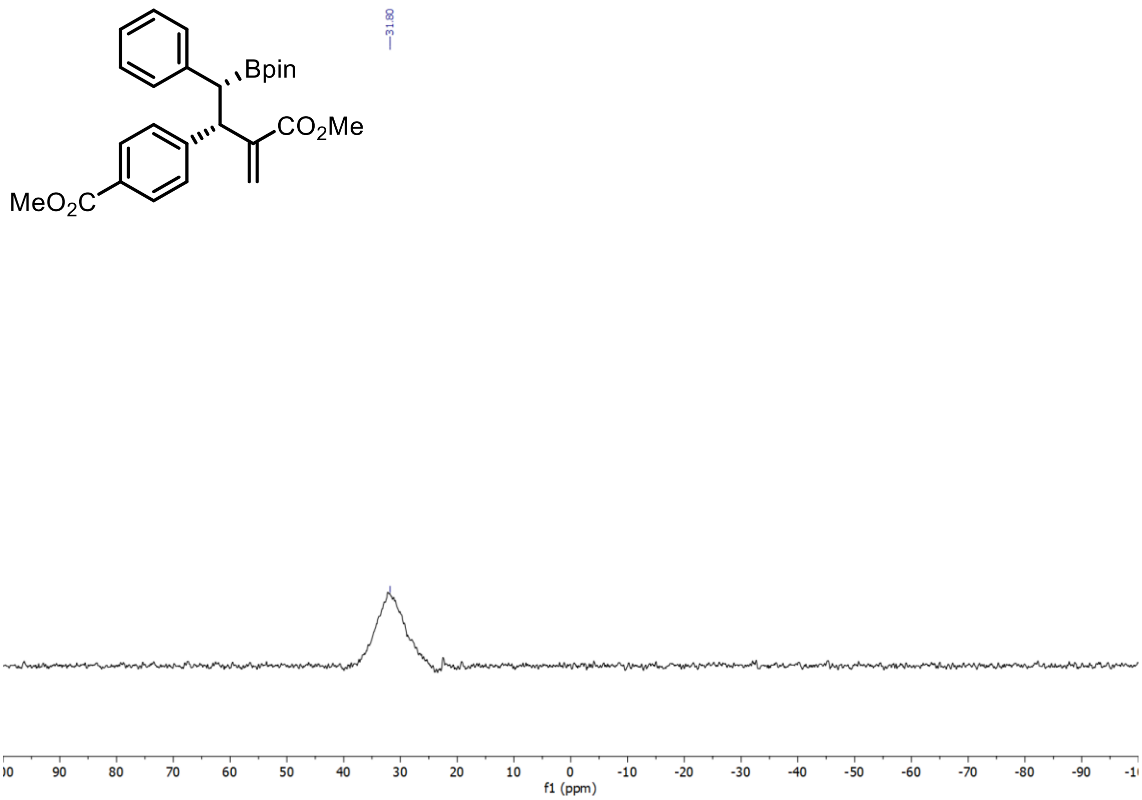

^{11}B NMR (128 MHz, $CDCl_3$) spectra of **3r’**.

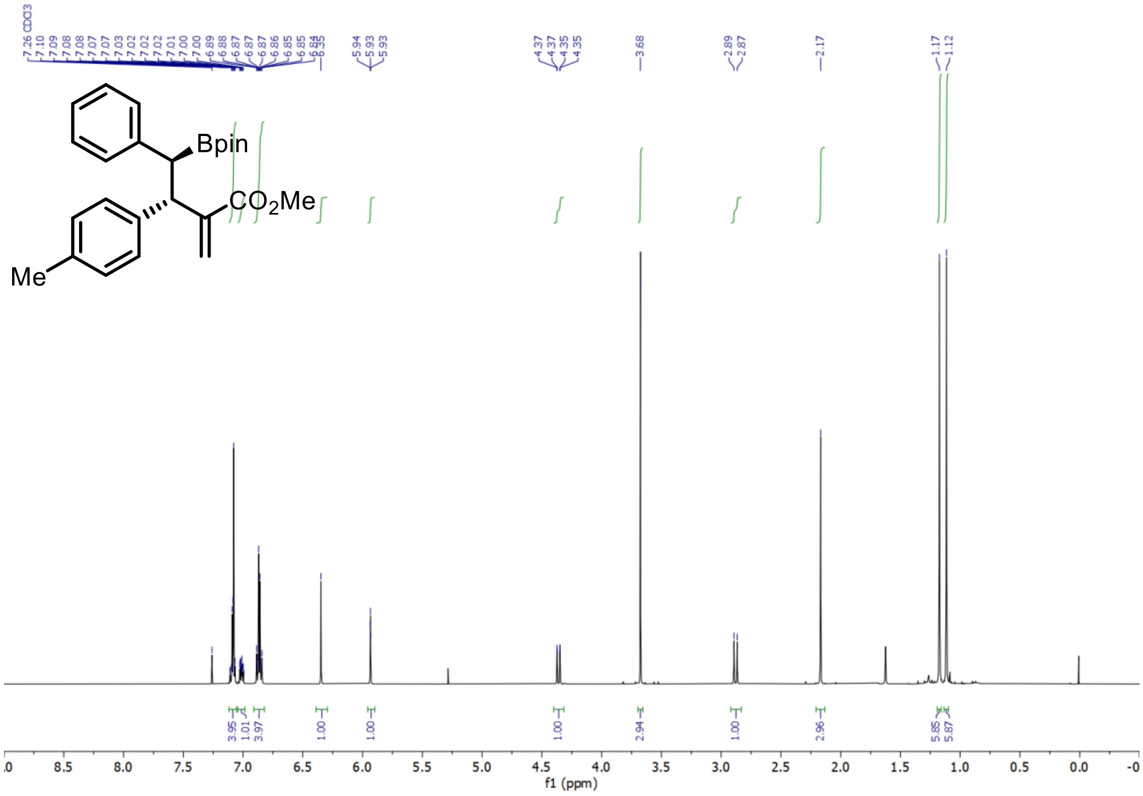

^{1}H NMR (500 MHz, $CDCl_3$) spectra of **3s**.

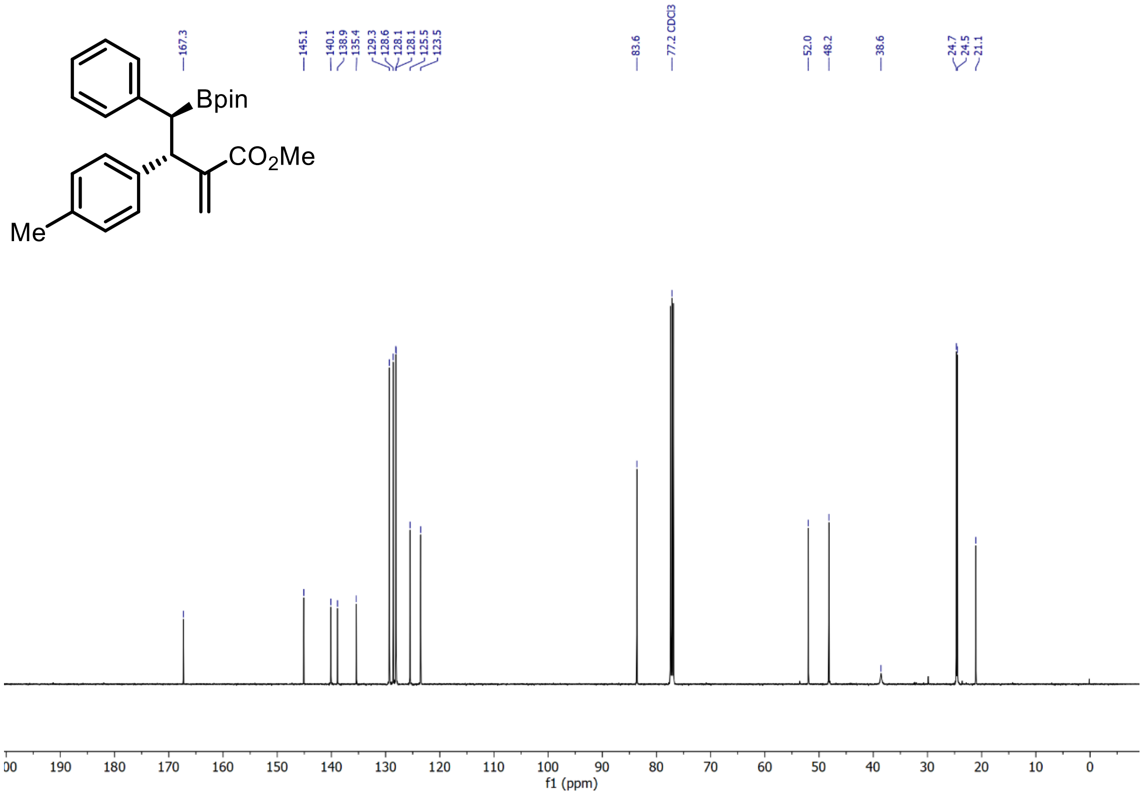

^{13}C NMR (126 MHz, $CDCl_3$) spectra of **3s**.

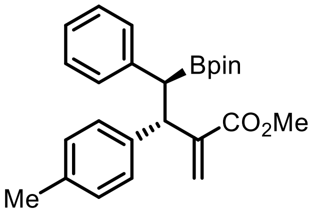

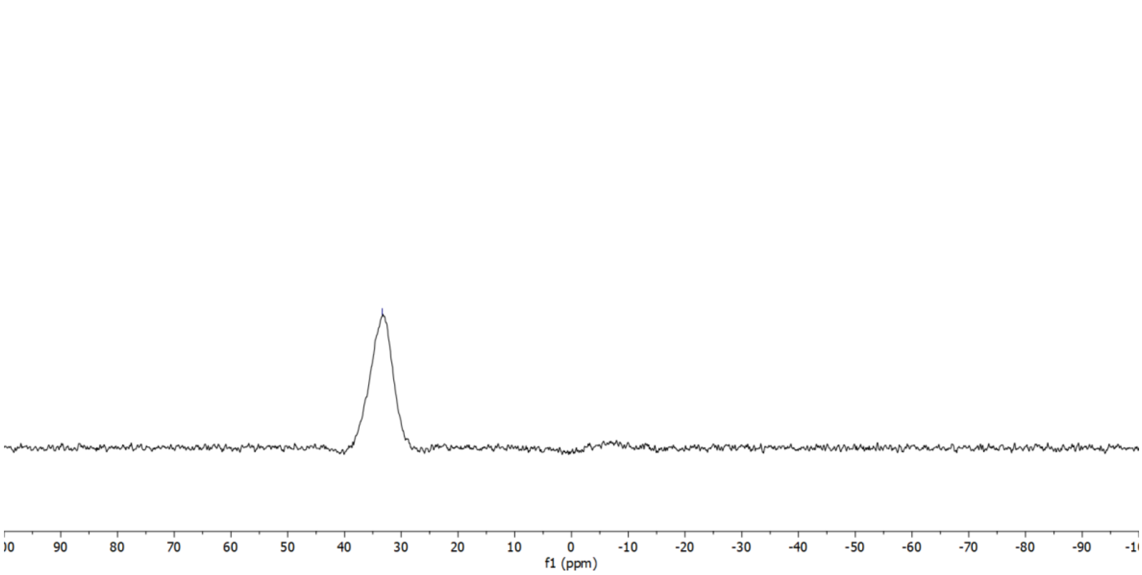

^{11}B NMR (128 MHz, $CDCl_3$) spectra of **3s**.

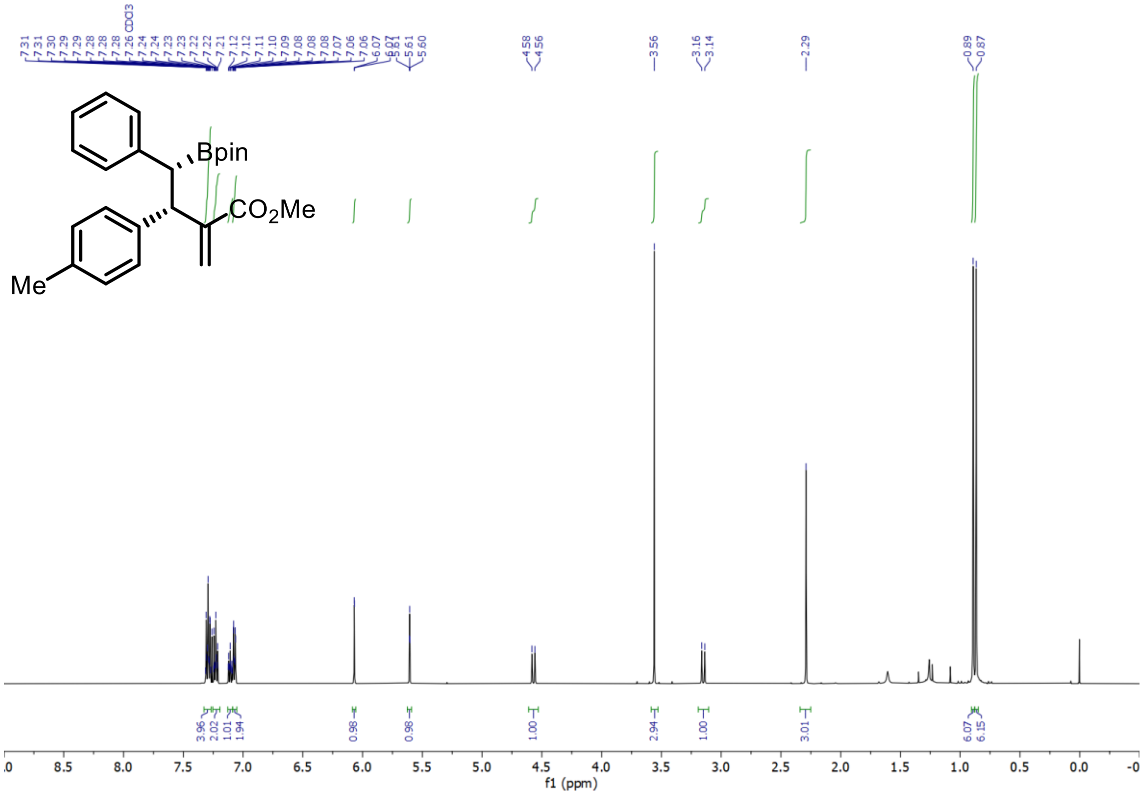

^{1}H NMR (500 MHz, $CDCl_3$) spectra of **3s’**.

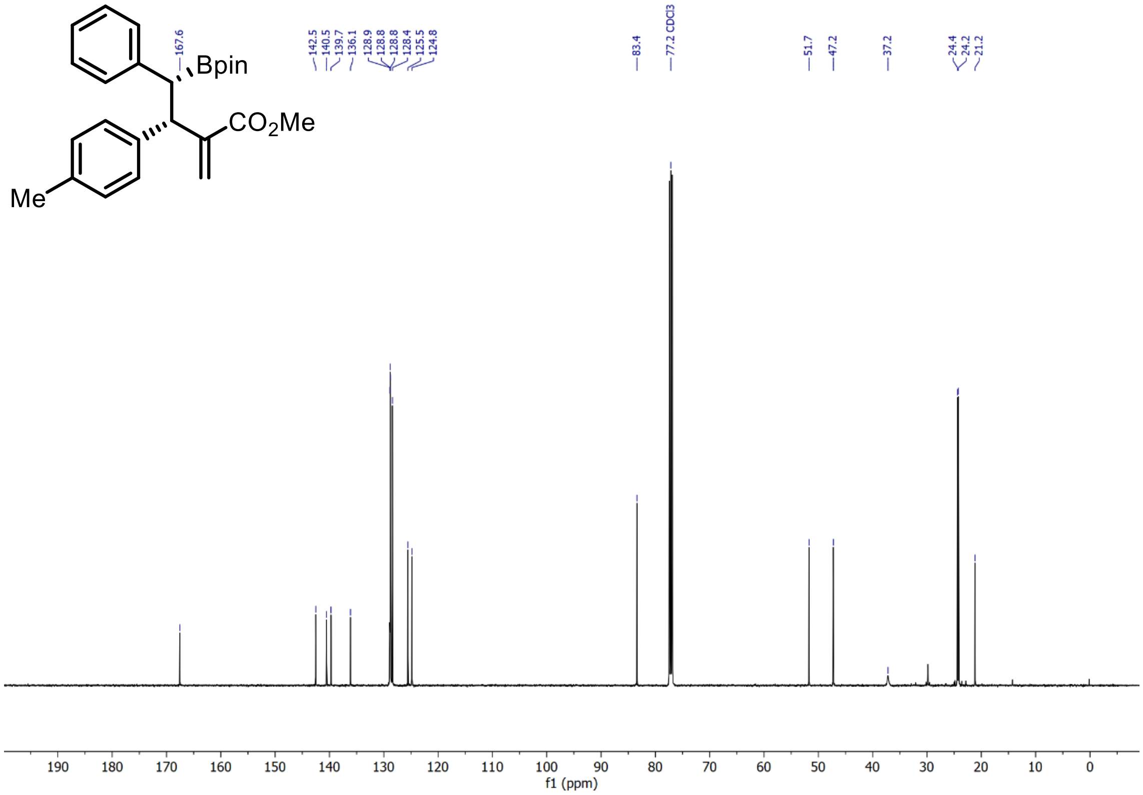

^{13}C NMR (126 MHz, $CDCl_3$) spectra of **3s’**.

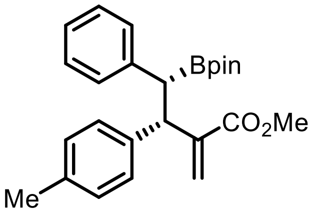

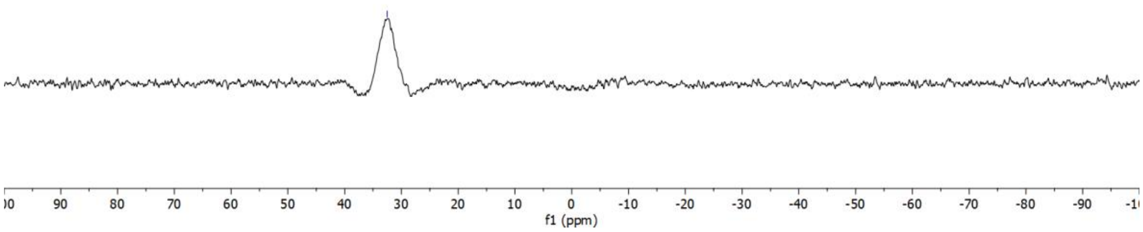

^{11}B NMR (128 MHz, $CDCl_3$) spectra of **3s’**.

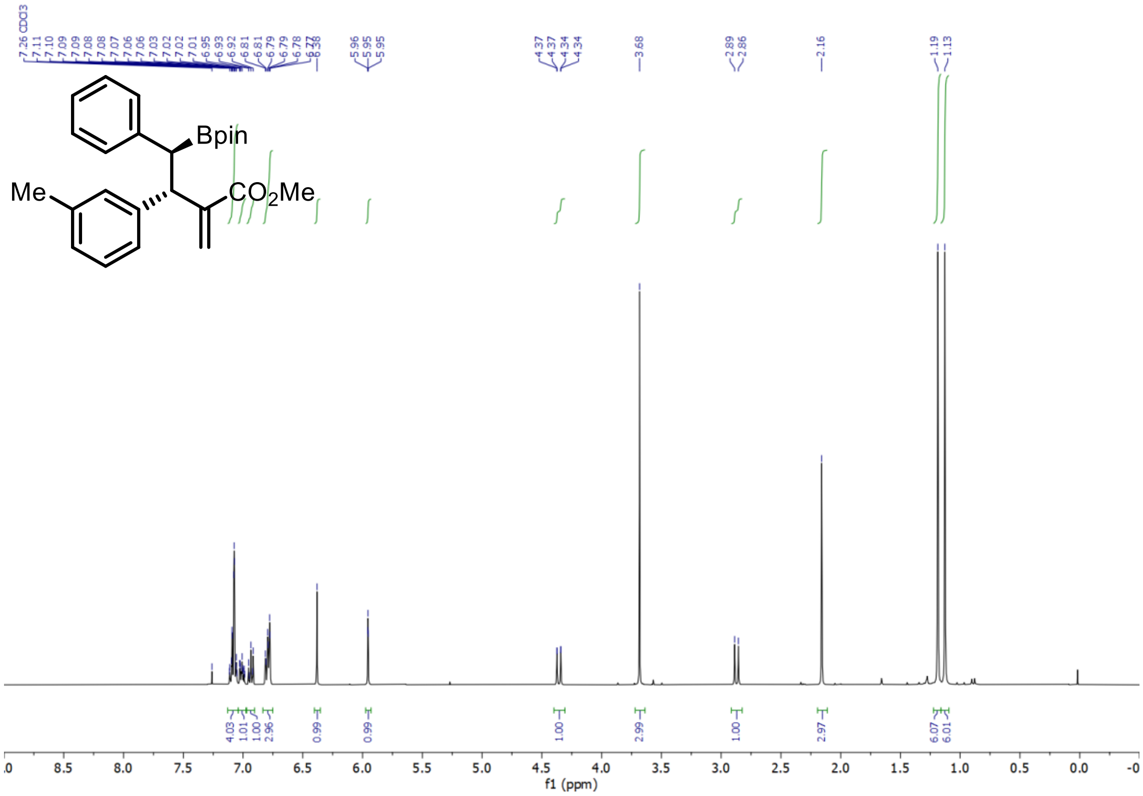

^{1}H NMR (400 MHz, $CDCl_3$) spectra of **3t**.

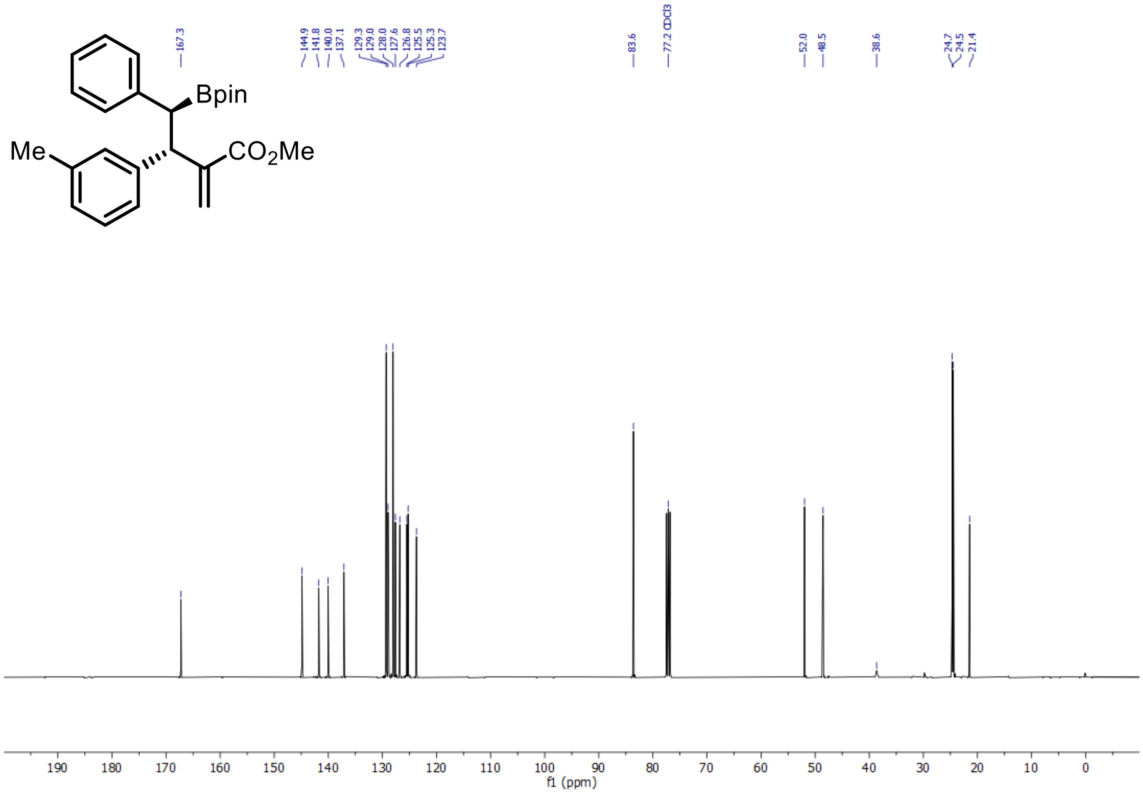

^{13}C NMR (101 MHz, $CDCl_3$) spectra of **3t**.

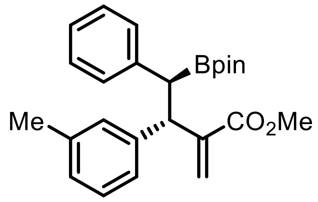

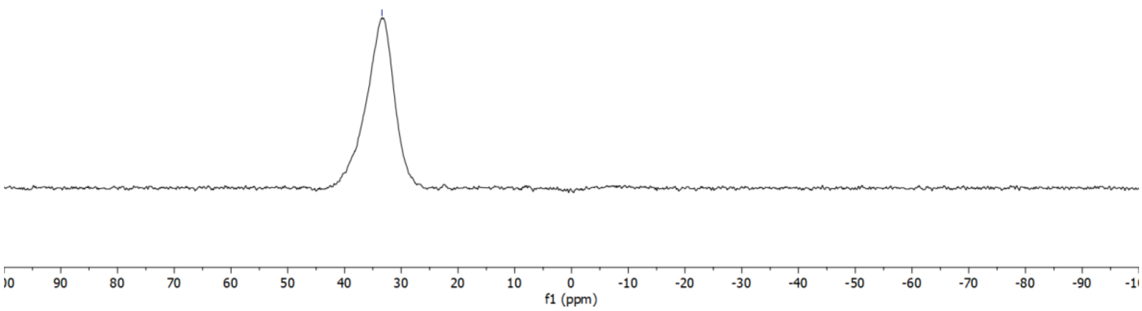

^{11}B NMR (128 MHz, $CDCl_3$) spectra of **3t**.

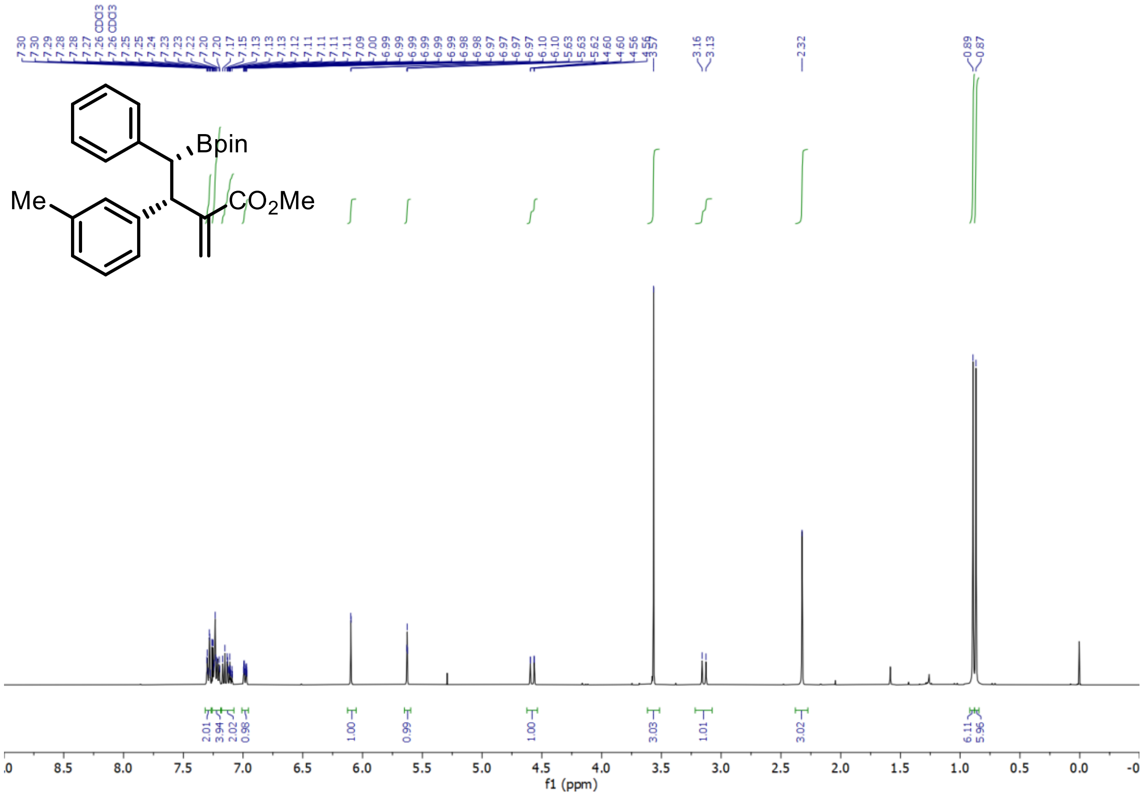

^{1}H NMR (400 MHz, $CDCl_3$) spectra of **3t’**.

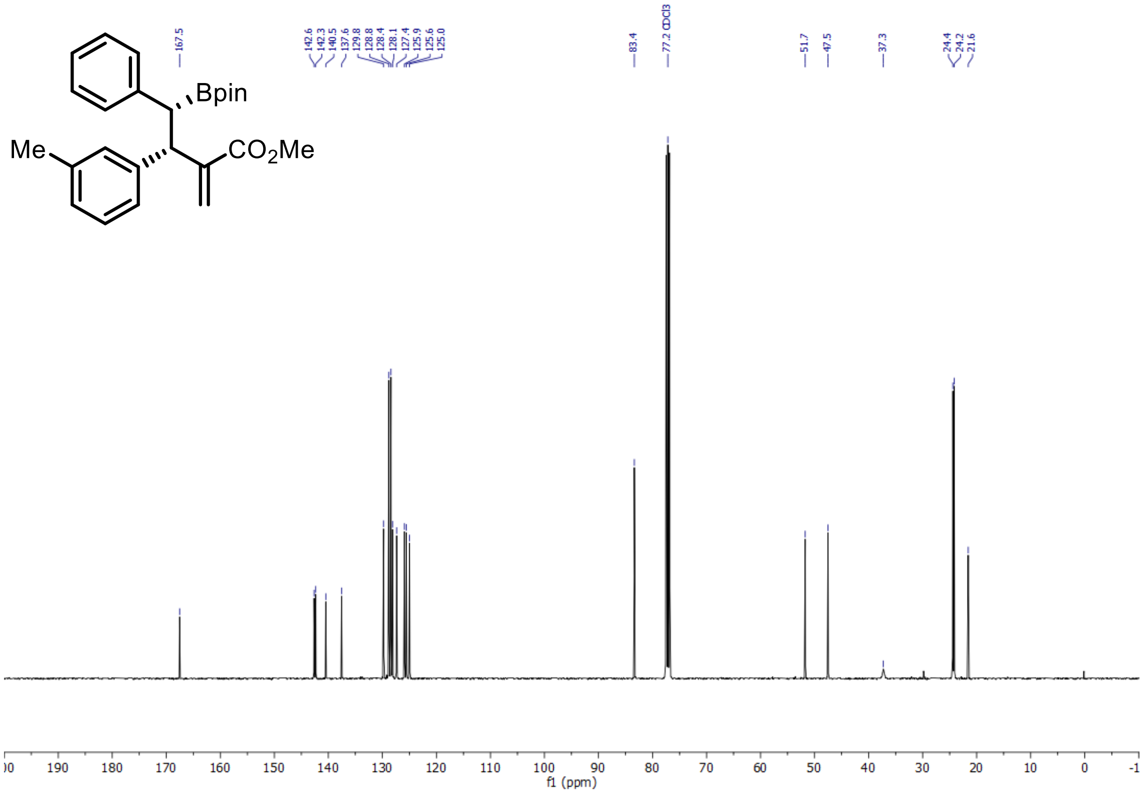

^{13}C NMR (101 MHz, $CDCl_3$) spectra of **3t’**.

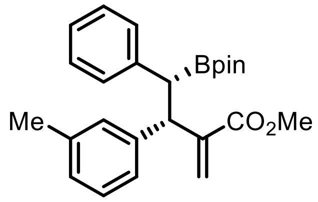

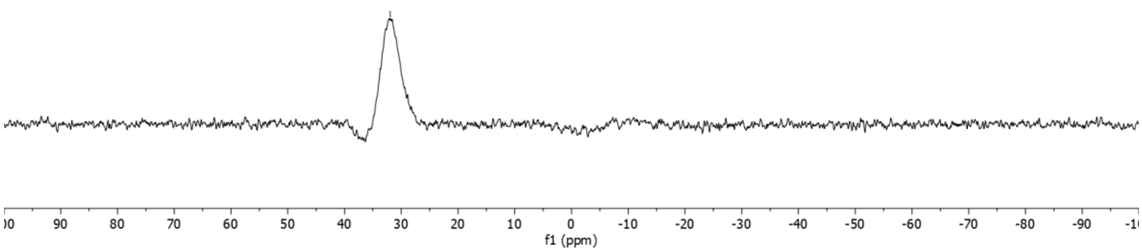

^{11}B NMR (128 MHz, $CDCl_3$) spectra of **3t’**.

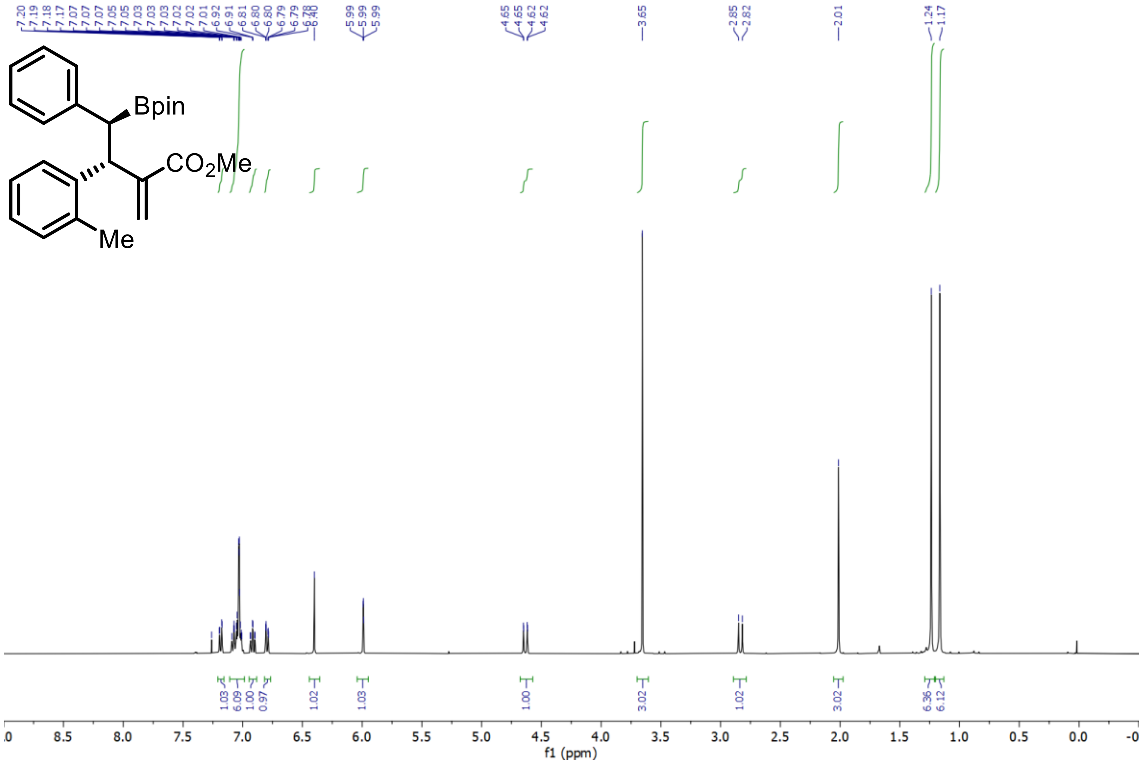

^{1}H NMR (400 MHz, $CDCl_3$) spectra of **3u**.

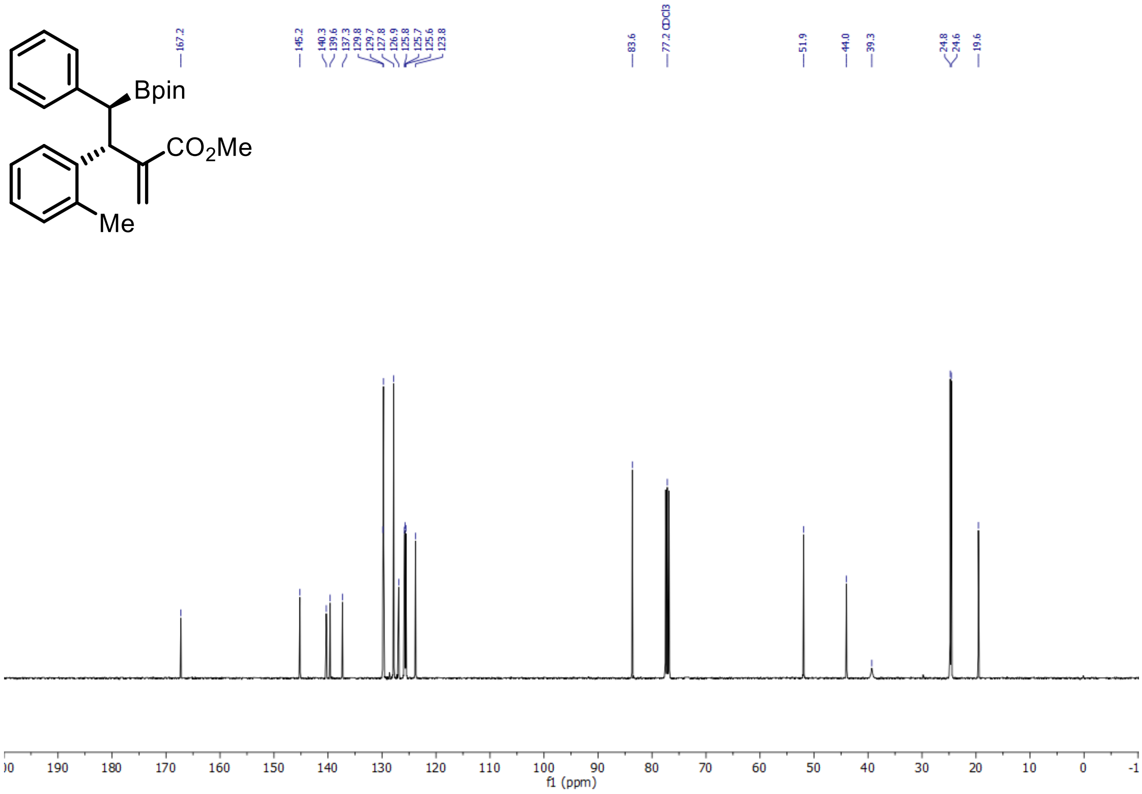

^{13}C NMR (101 MHz, $CDCl_3$) spectra of **3u**.

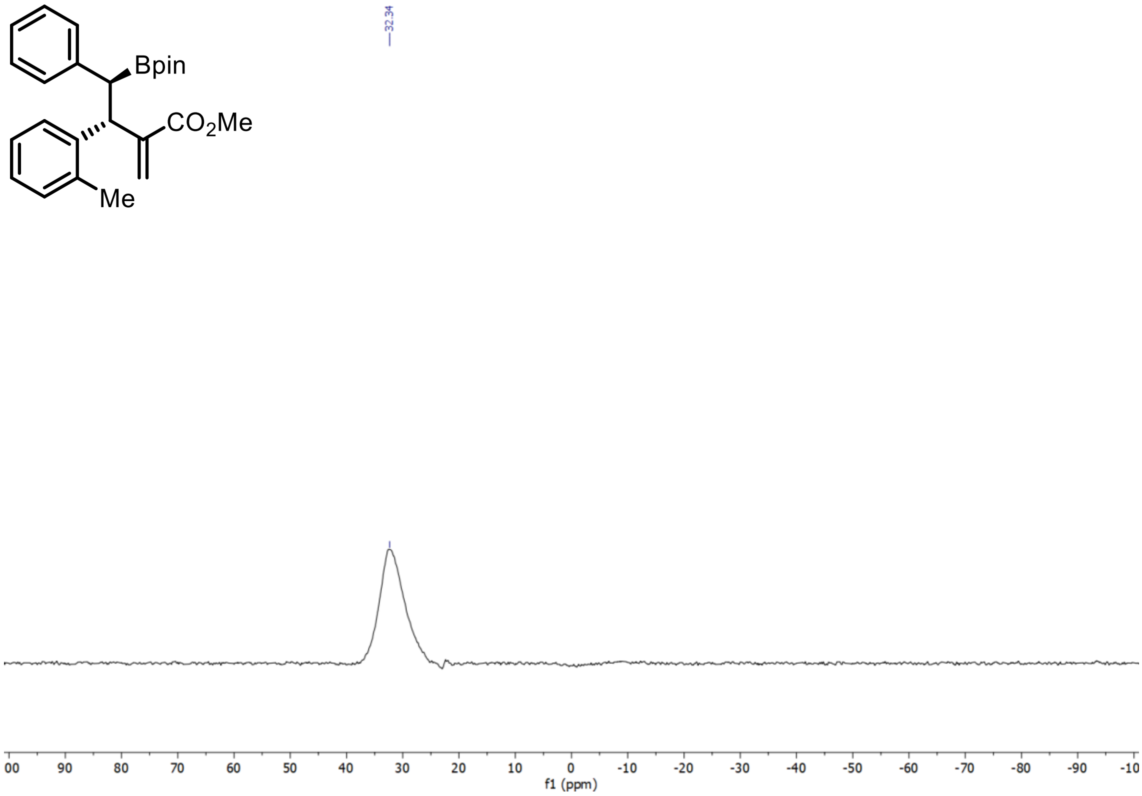

^{11}B NMR (128 MHz, $CDCl_3$) spectra of **3u**.

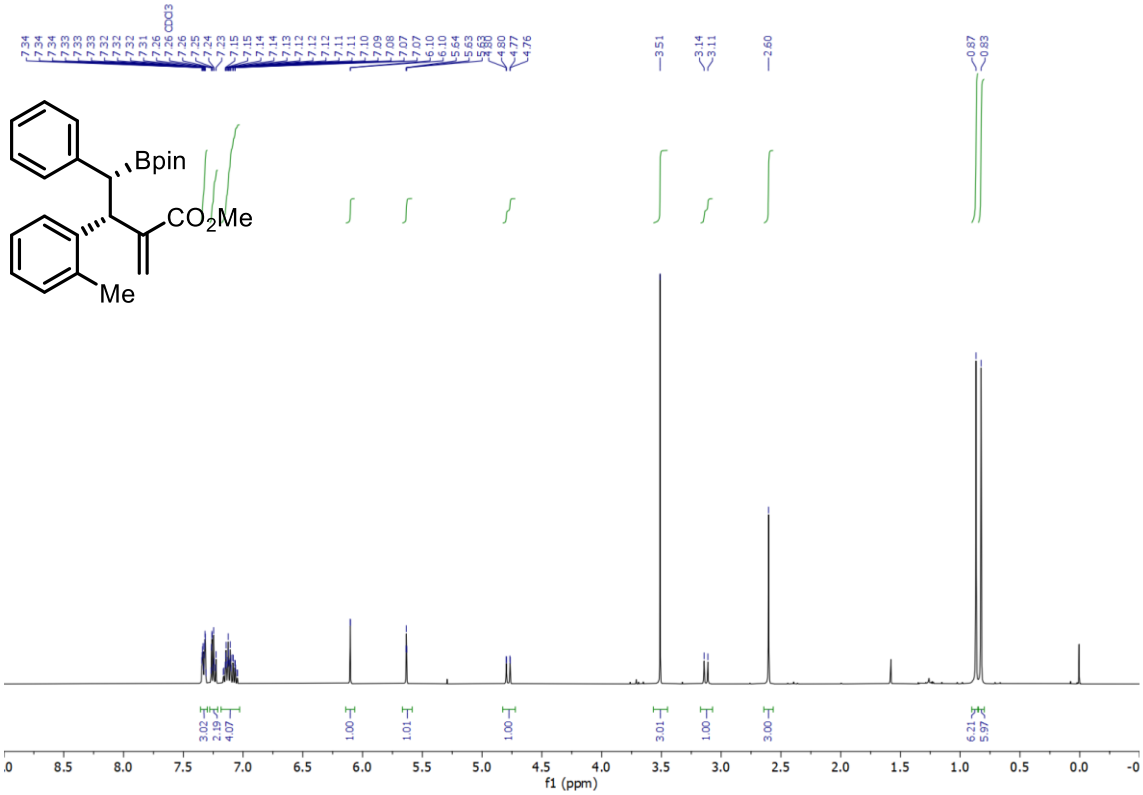

^{1}H NMR (400 MHz, $CDCl_3$) spectra of **3u’**.

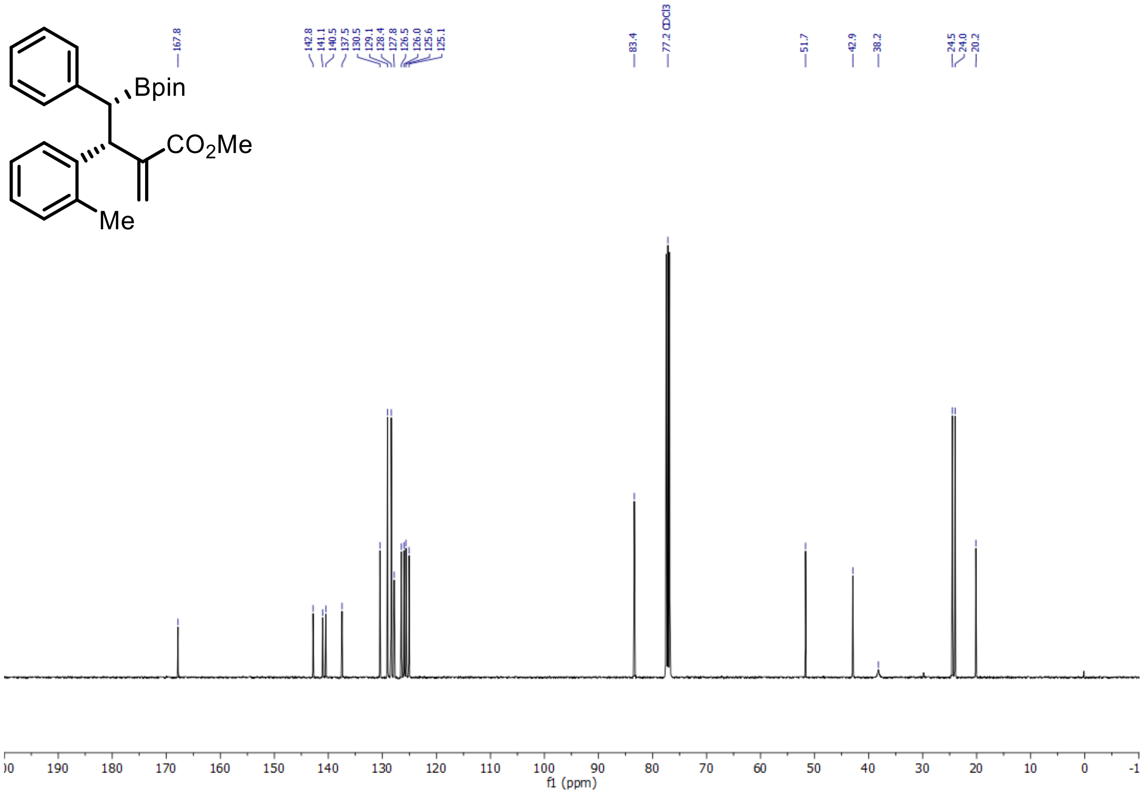

^{13}C NMR (101 MHz, $CDCl_3$) spectra of **3u’**.

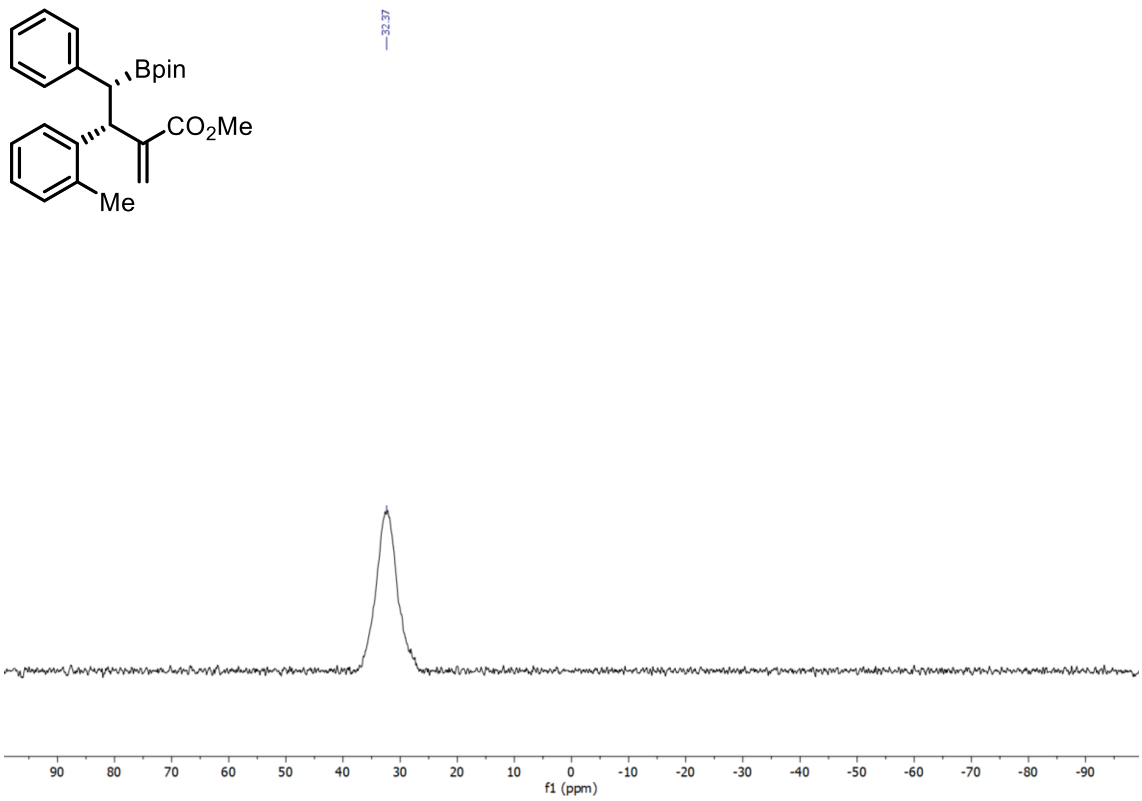

^{11}B NMR (128 MHz, $CDCl_3$) spectra of **3u’**.

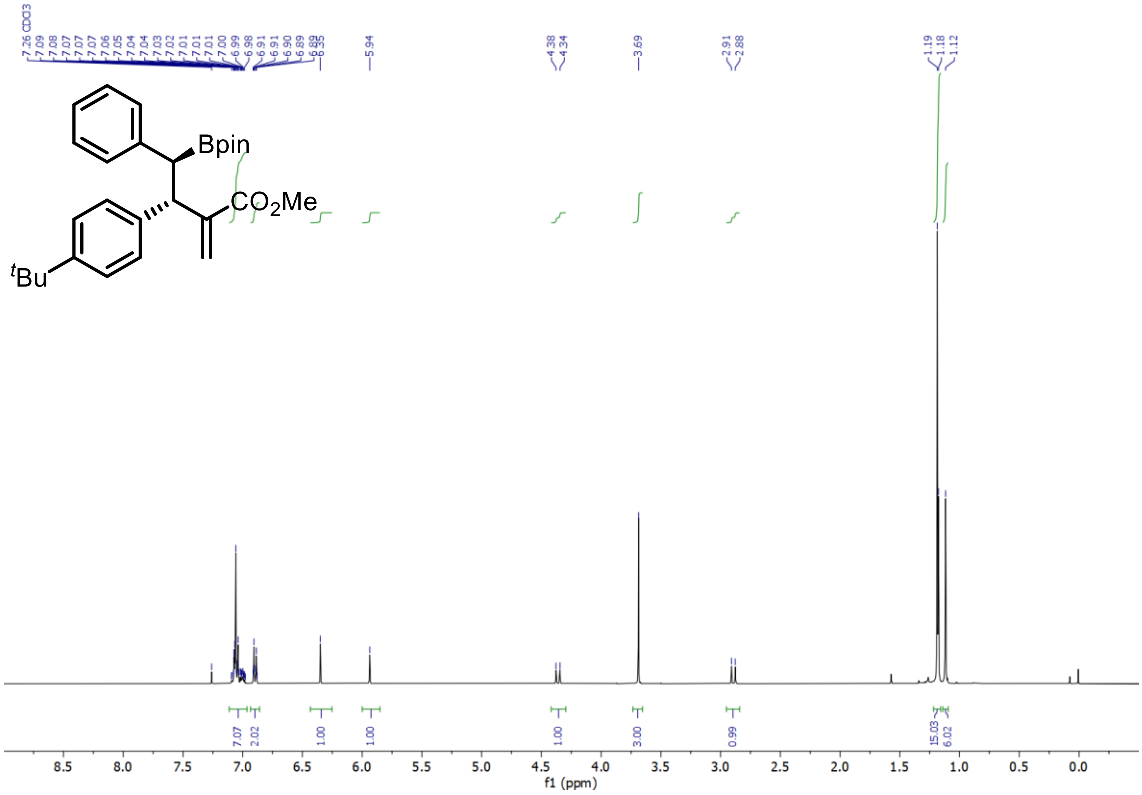

^{1}H NMR (400 MHZ, $CDCl_3$) spectra of **3v**.

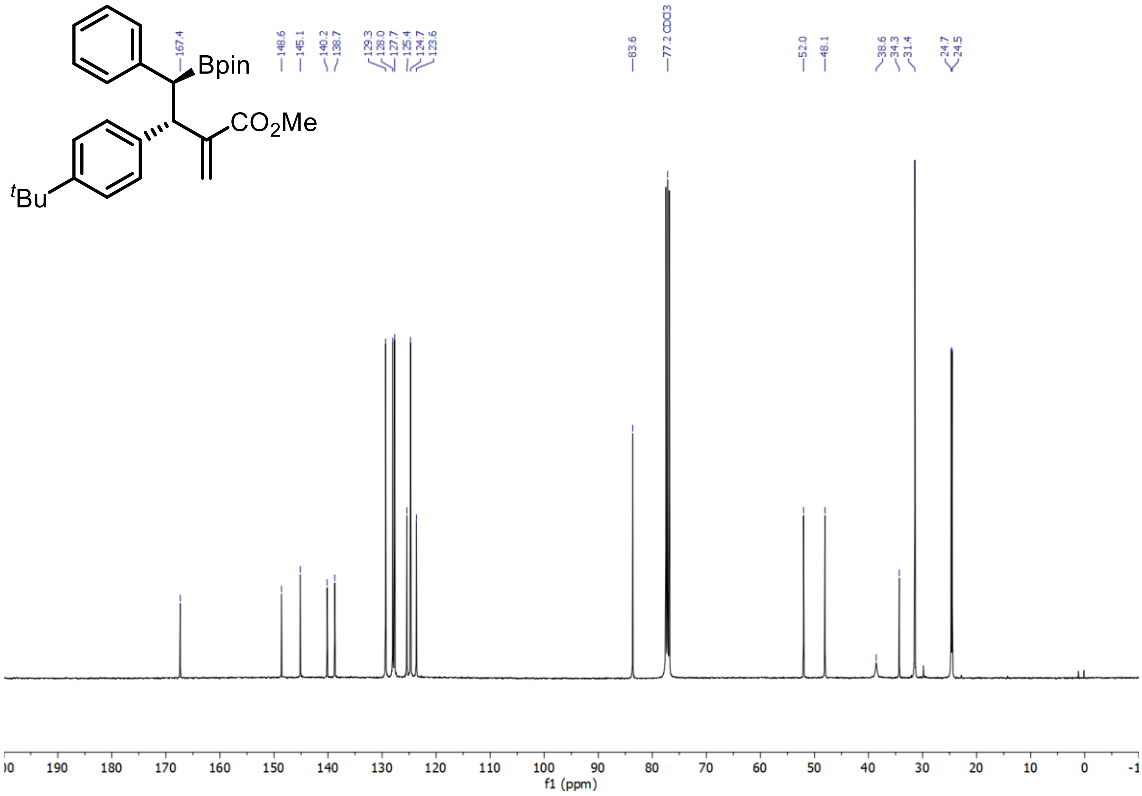

^{13}C NMR (101 MHZ, $CDCl_3$) spectra of **3v**.

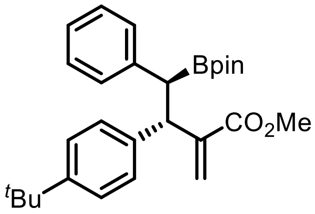

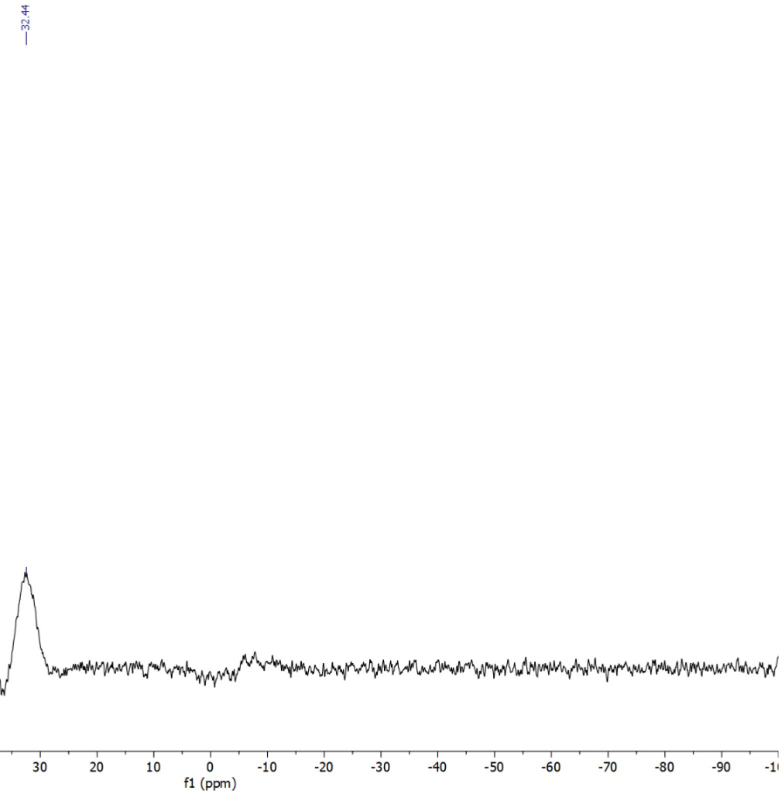

^{11}B NMR (128 MHZ, $CDCl_3$) spectra of **3v**.

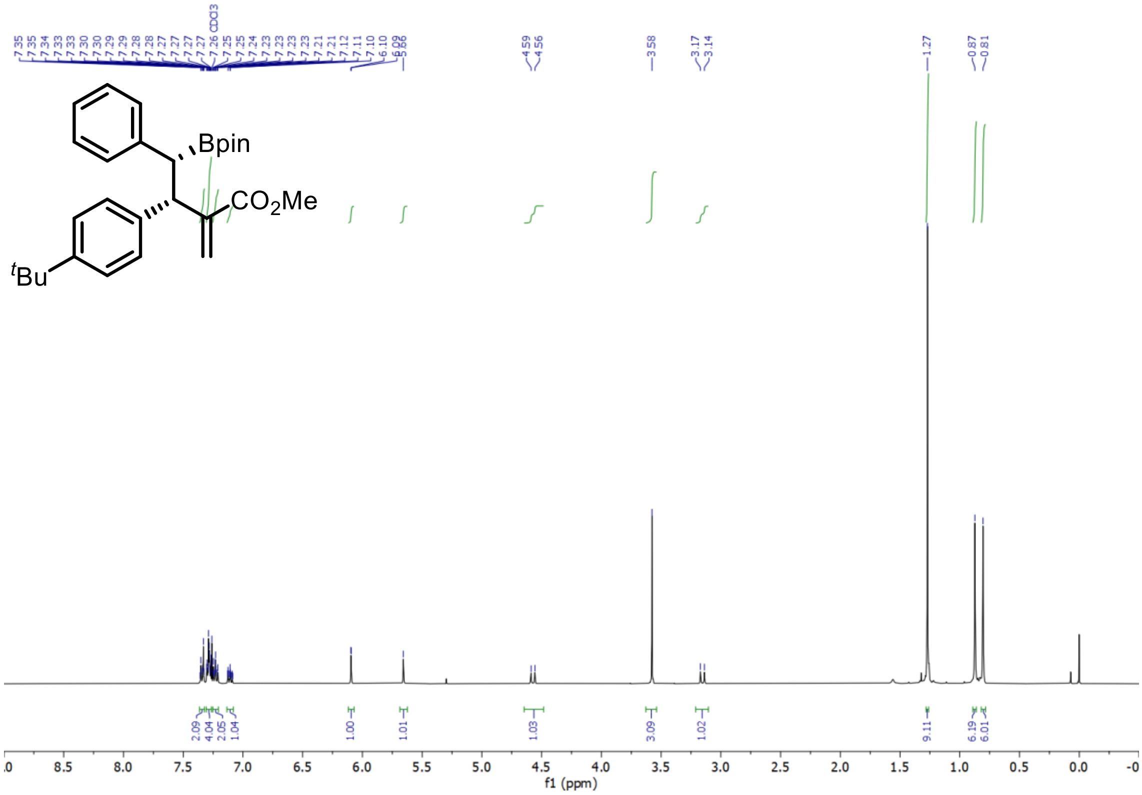

^{1}H NMR (400 MHZ, $CDCl_3$) spectra of **3v’**.

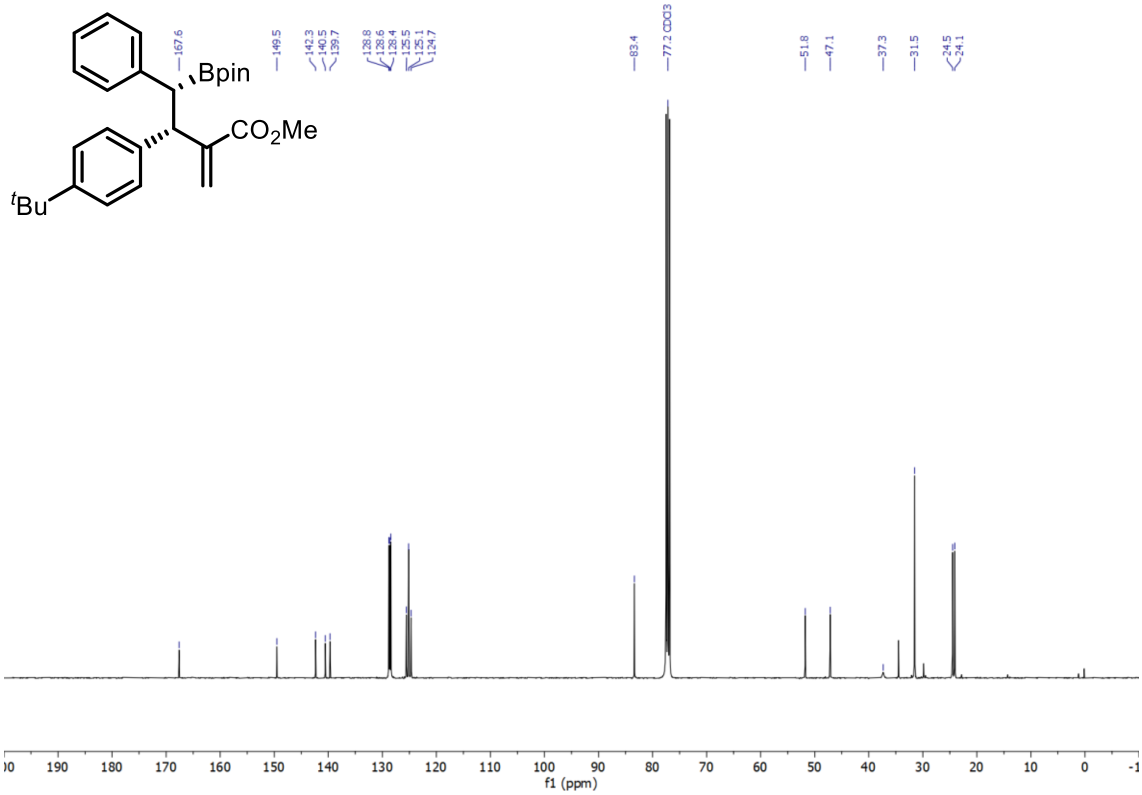

^{13}C NMR (101 MHZ, $CDCl_3$) spectra of **3v’**.

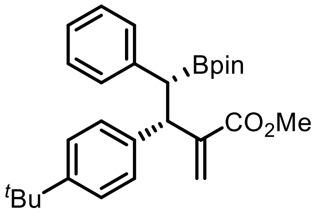

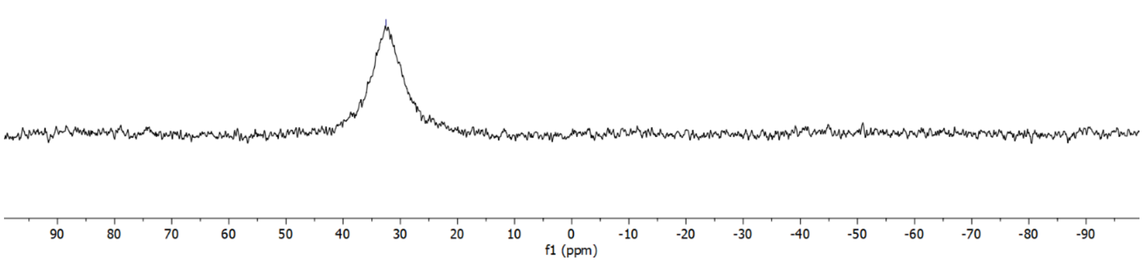

^{11}B NMR (128 MHZ, $CDCl_3$) spectra of **3v’**.

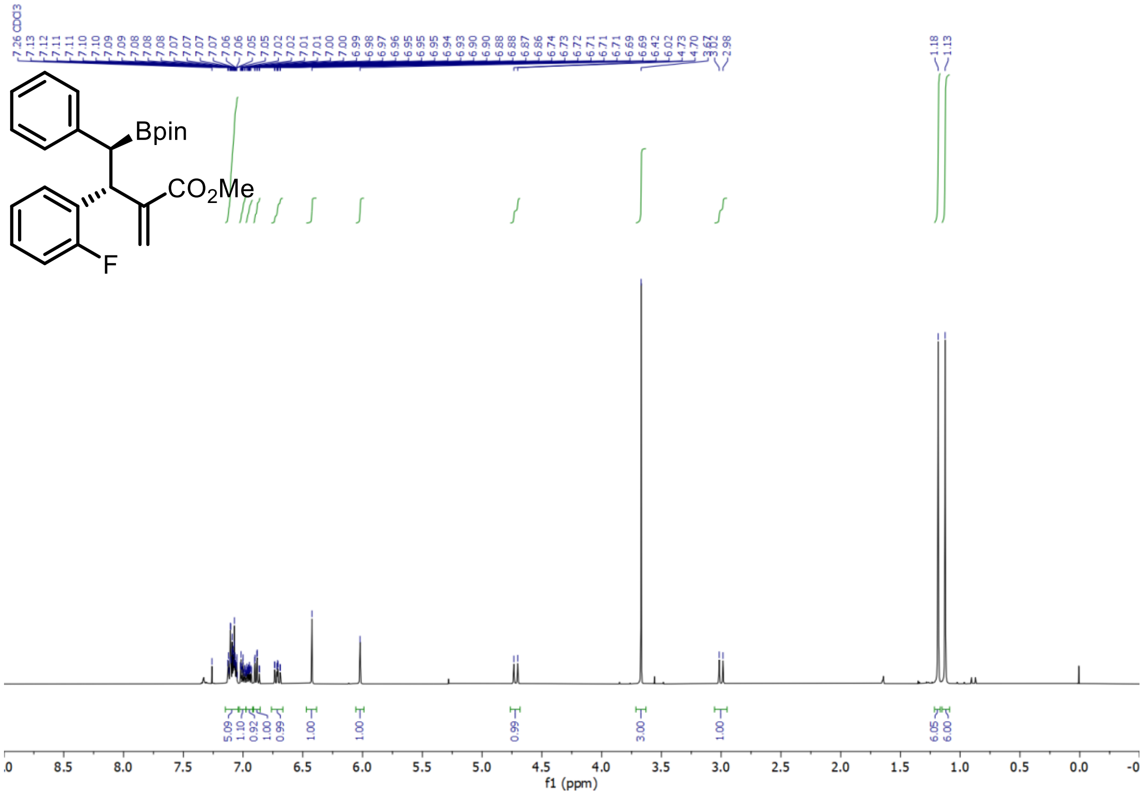

^{1}H NMR (400 MHz, $CDCl_3$) spectra of **3w**.

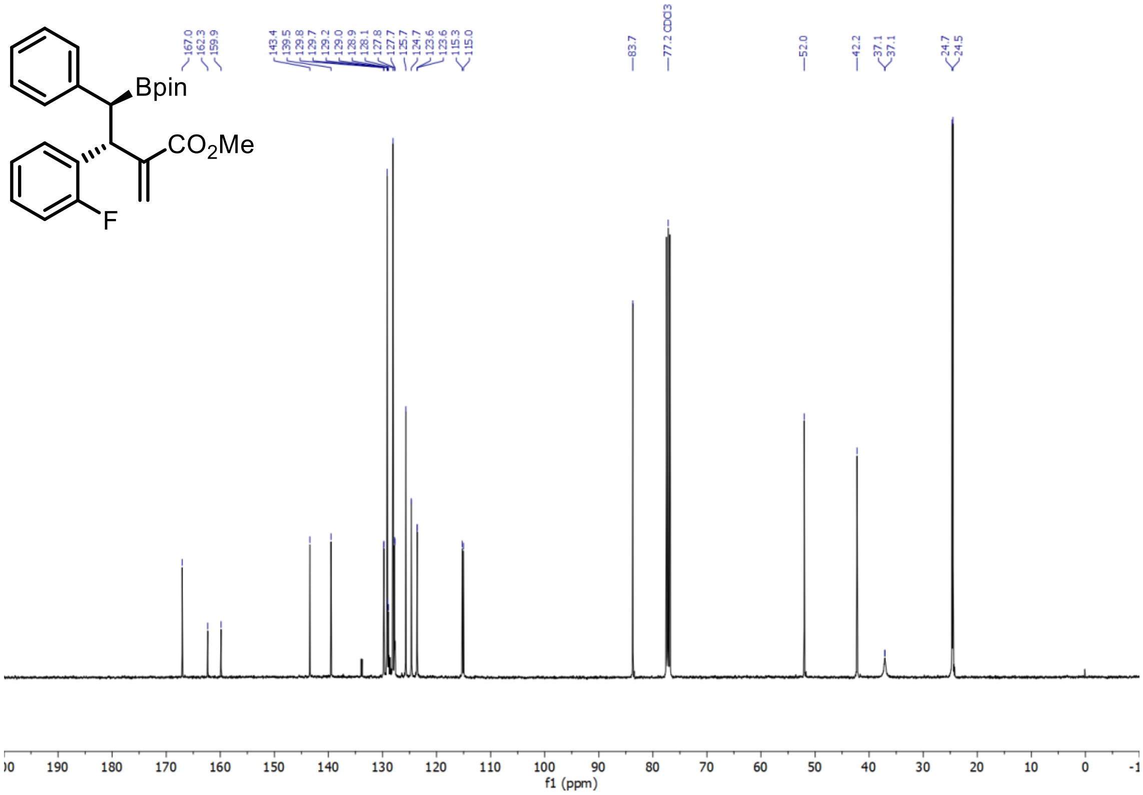

^{13}C NMR (101 MHz, $CDCl_3$) spectra of **3w**.

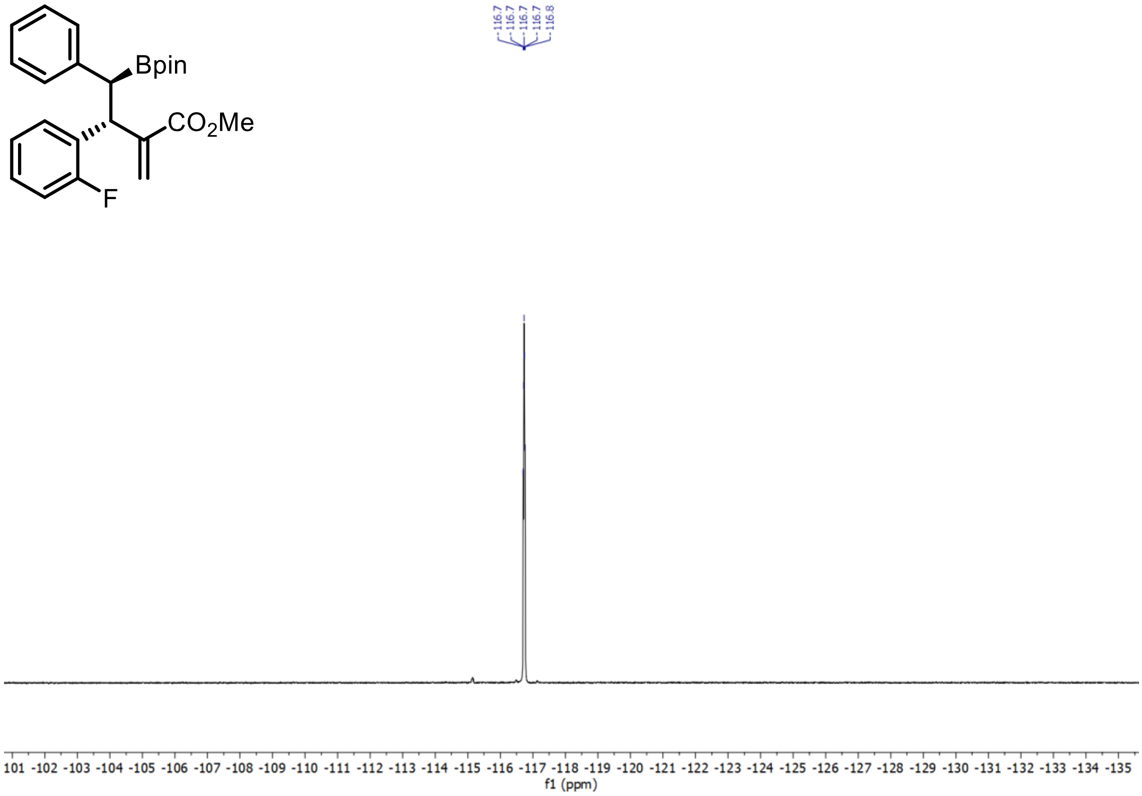

^{19}F NMR (376 MHz, $CDCl_3$) spectra of **3w**.

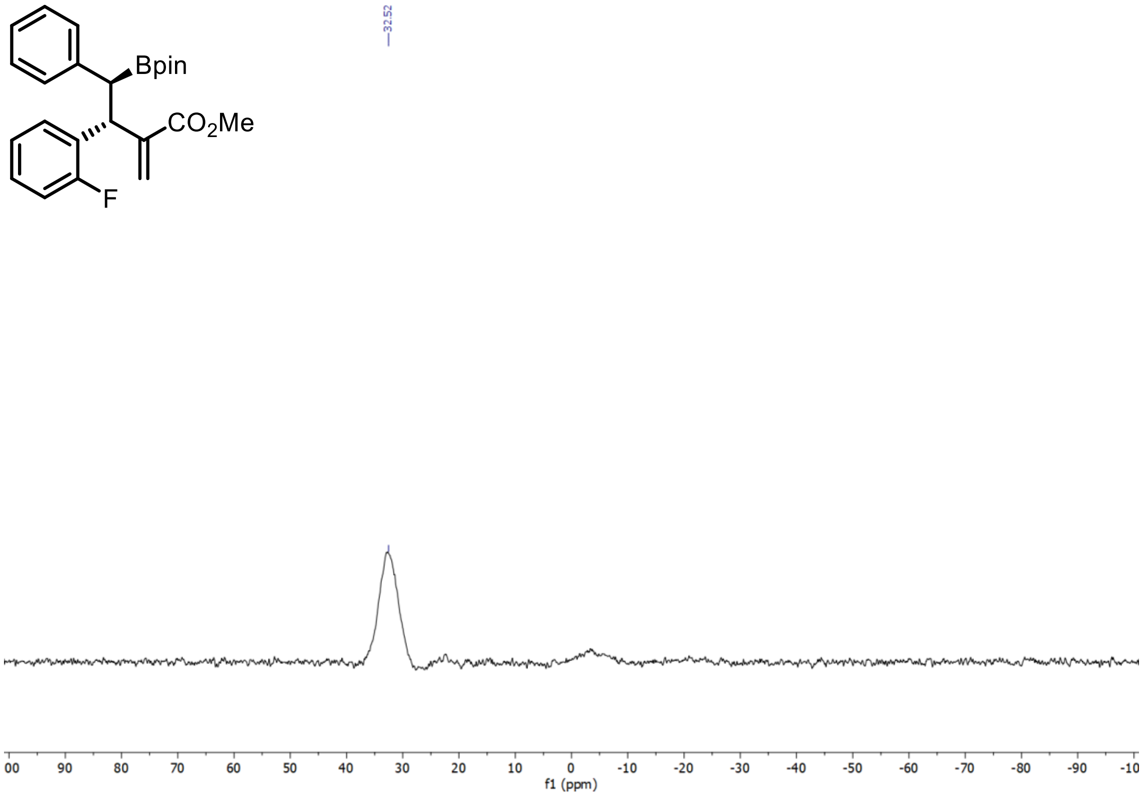

^{11}B NMR (128 MHz, $CDCl_3$) spectra of **3w**.

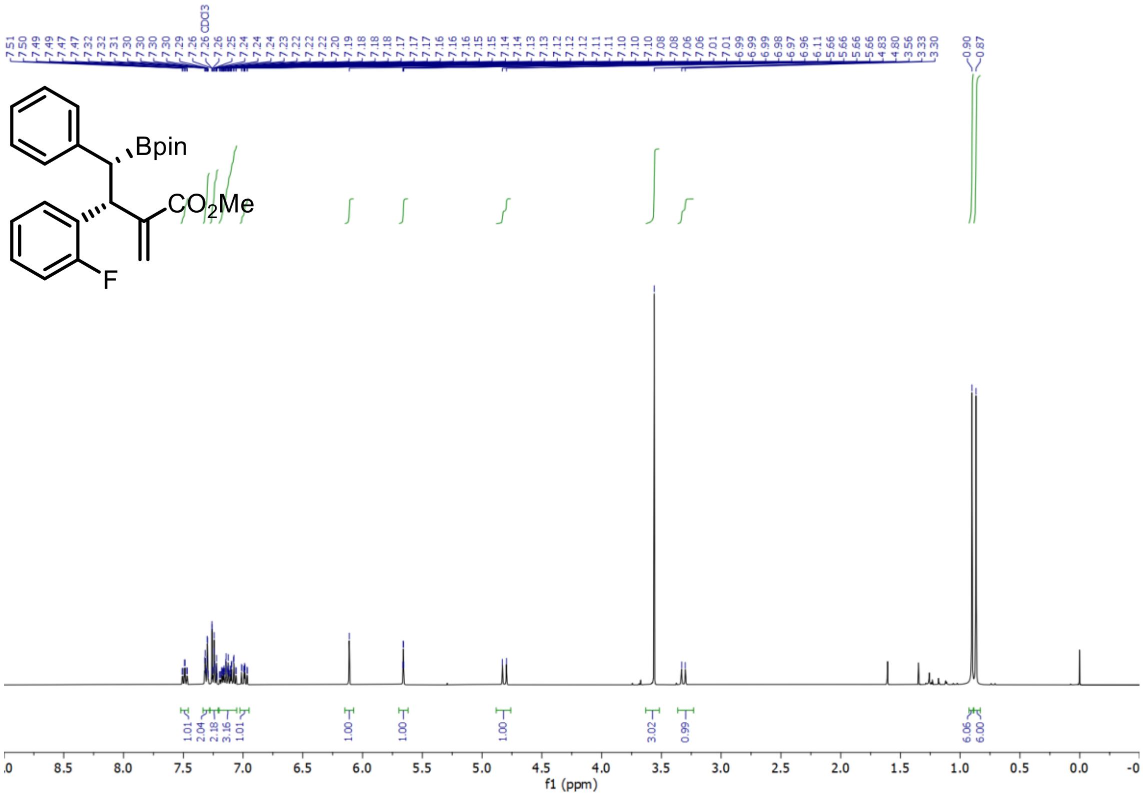

^{1}H NMR (400 MHz, $CDCl_3$) spectra of **3w’**.

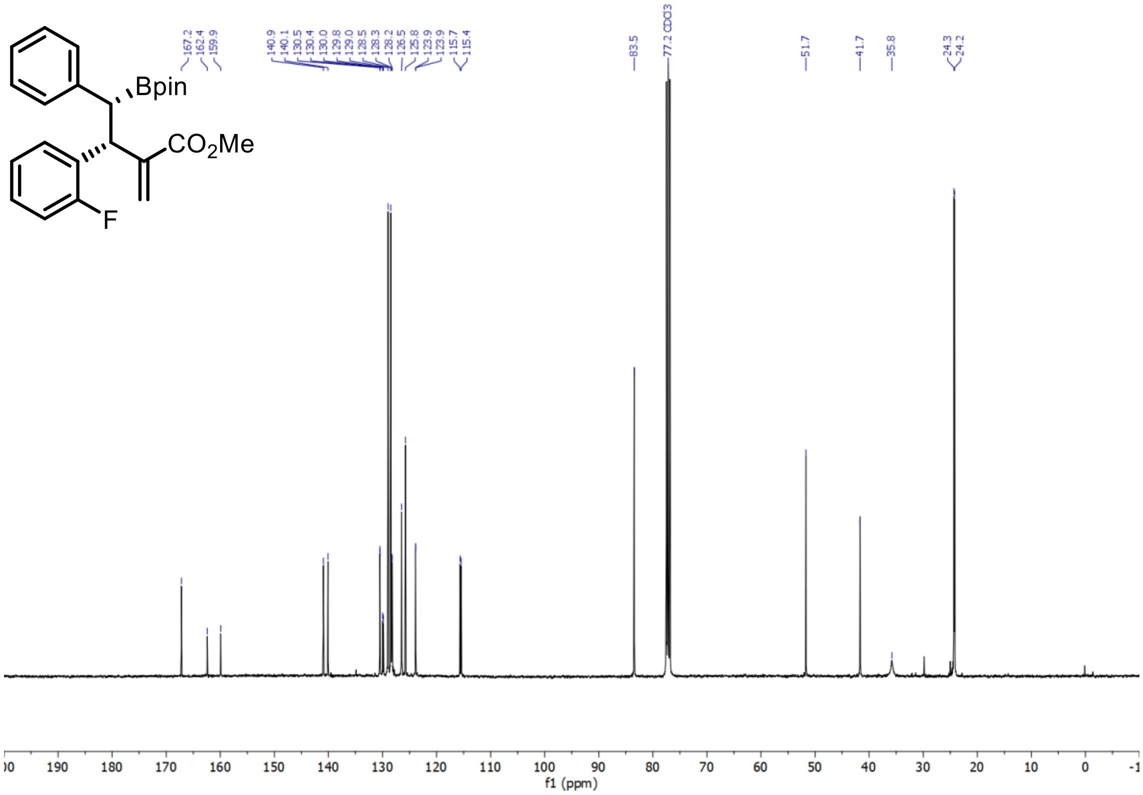

^{13}C NMR (101 MHz, $CDCl_3$) spectra of **3w’**.

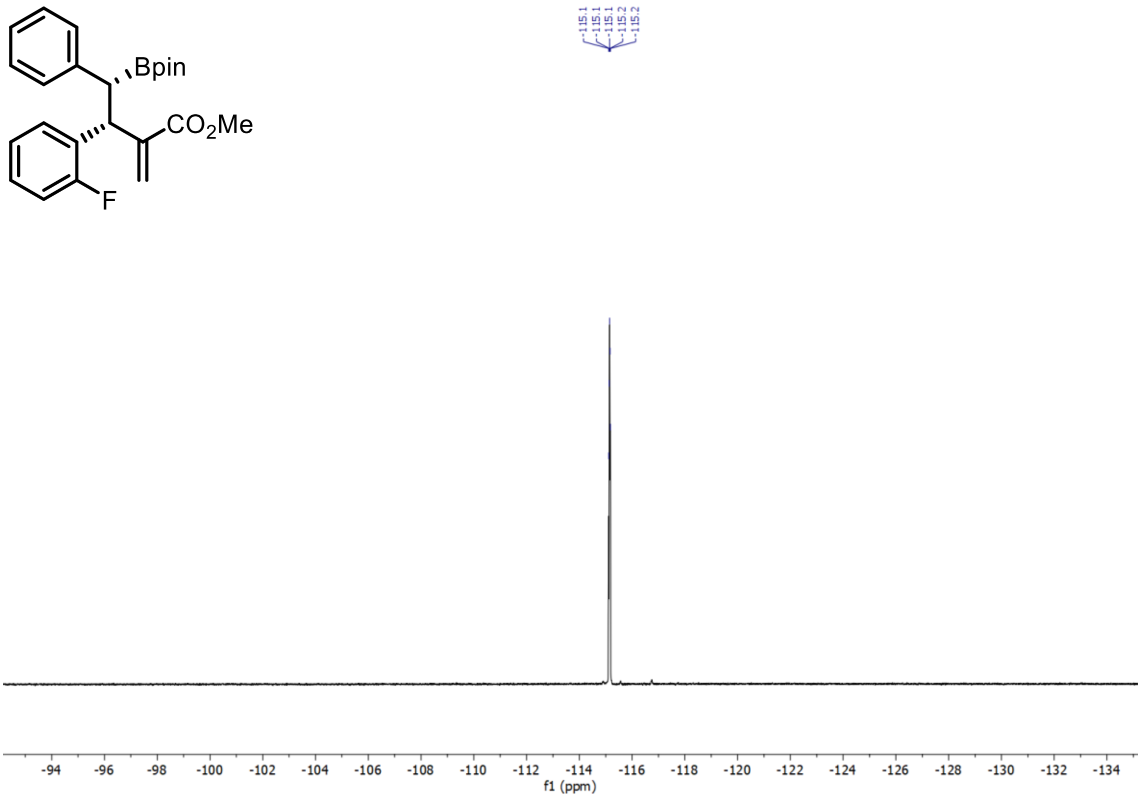

^{19}F NMR (376 MHz, $CDCl_3$) spectra of **3w'**.

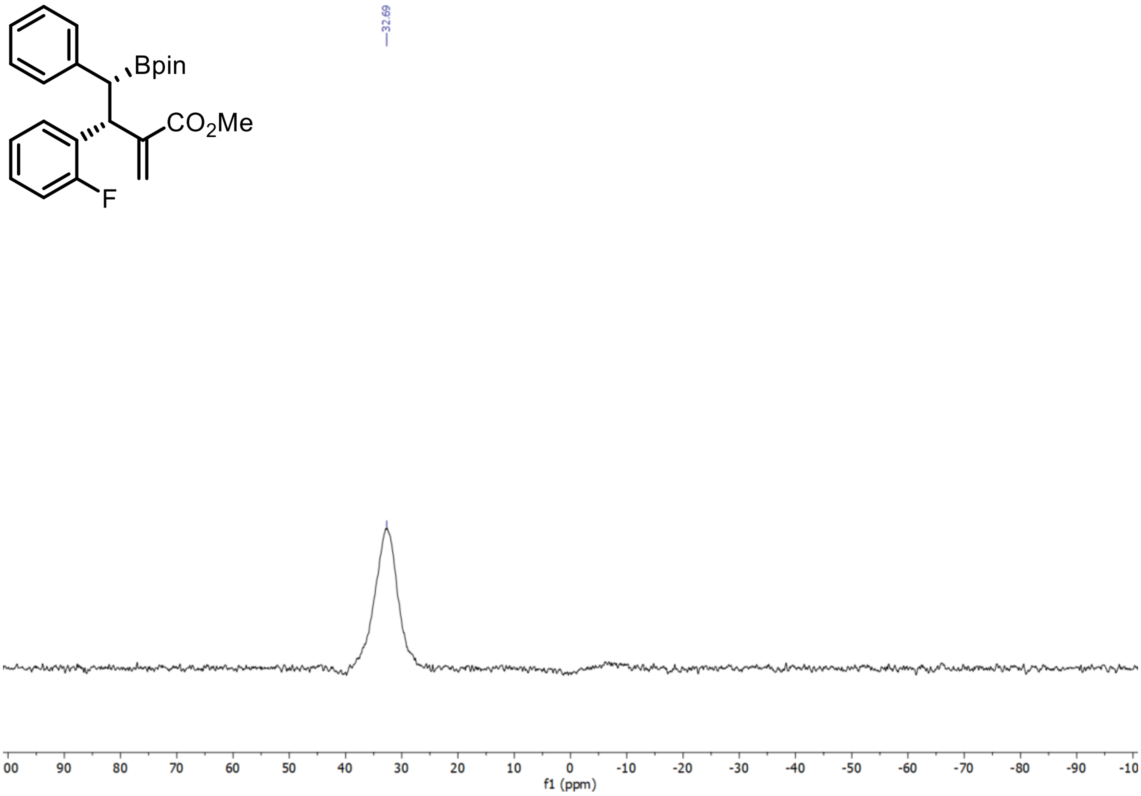

^{11}B NMR (128 MHz, $CDCl_3$) spectra of **3w'**.

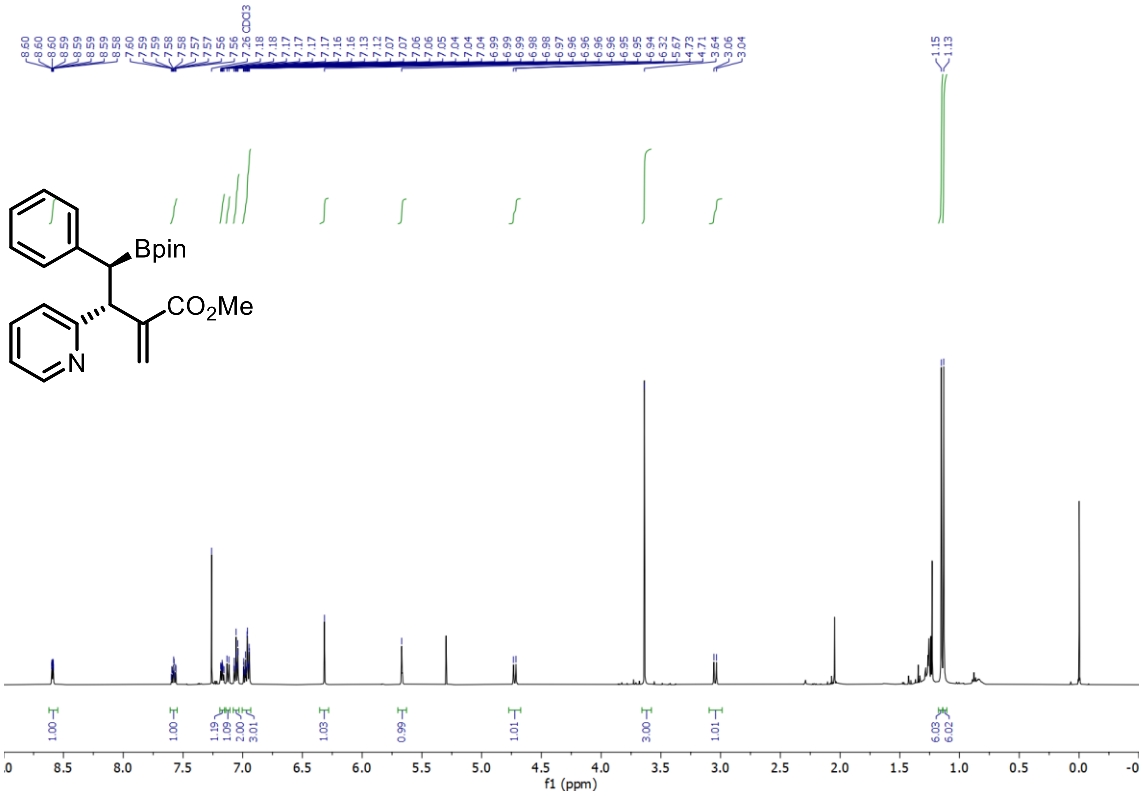

^{1}H NMR (500 MHz, $CDCl_3$) spectra of **3x**.

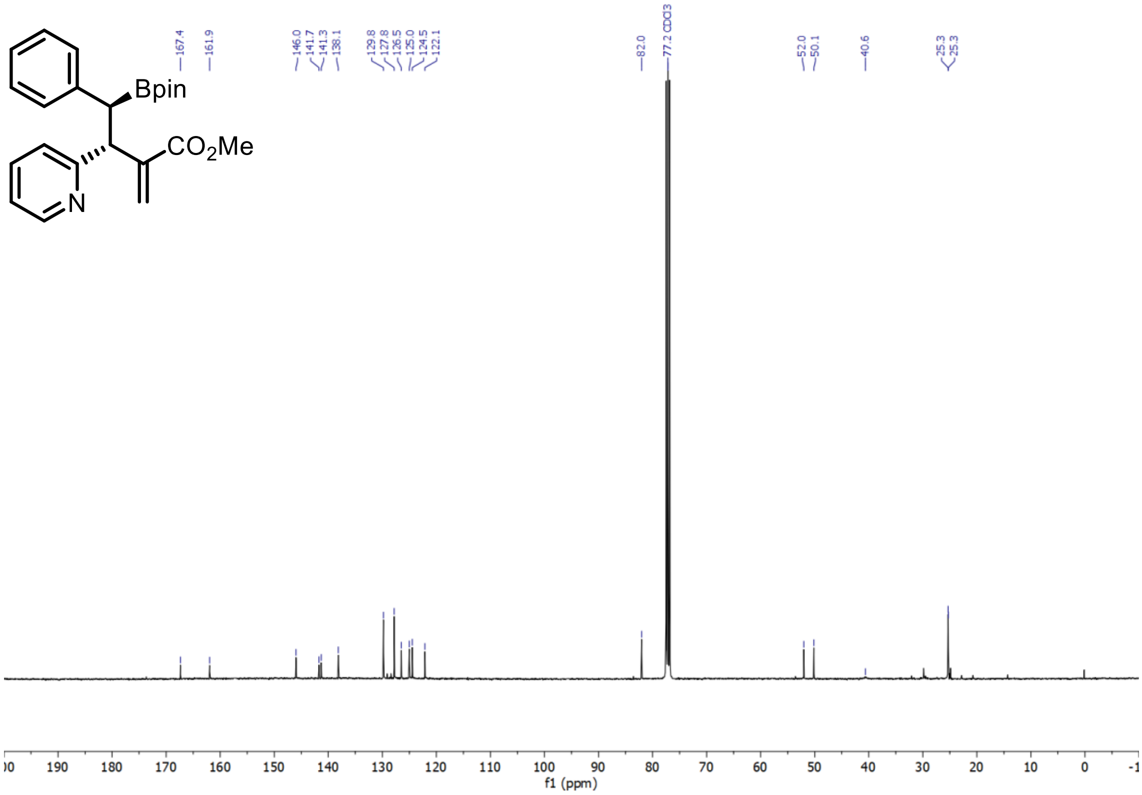

^{13}C NMR (101 MHz, $CDCl_3$) spectra of **3x**.

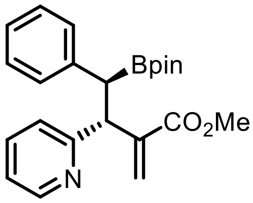

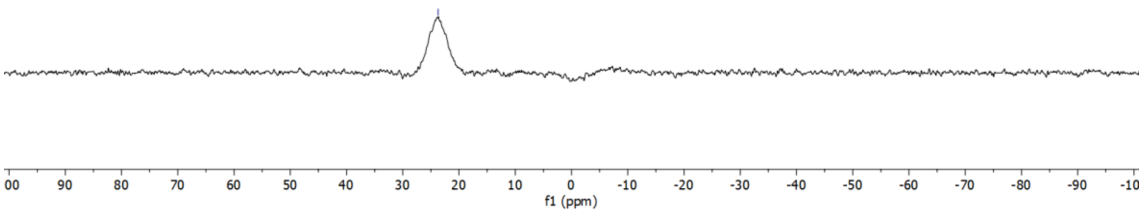

^{11}B NMR (128 MHz, $CDCl_3$) spectra of **3x**.

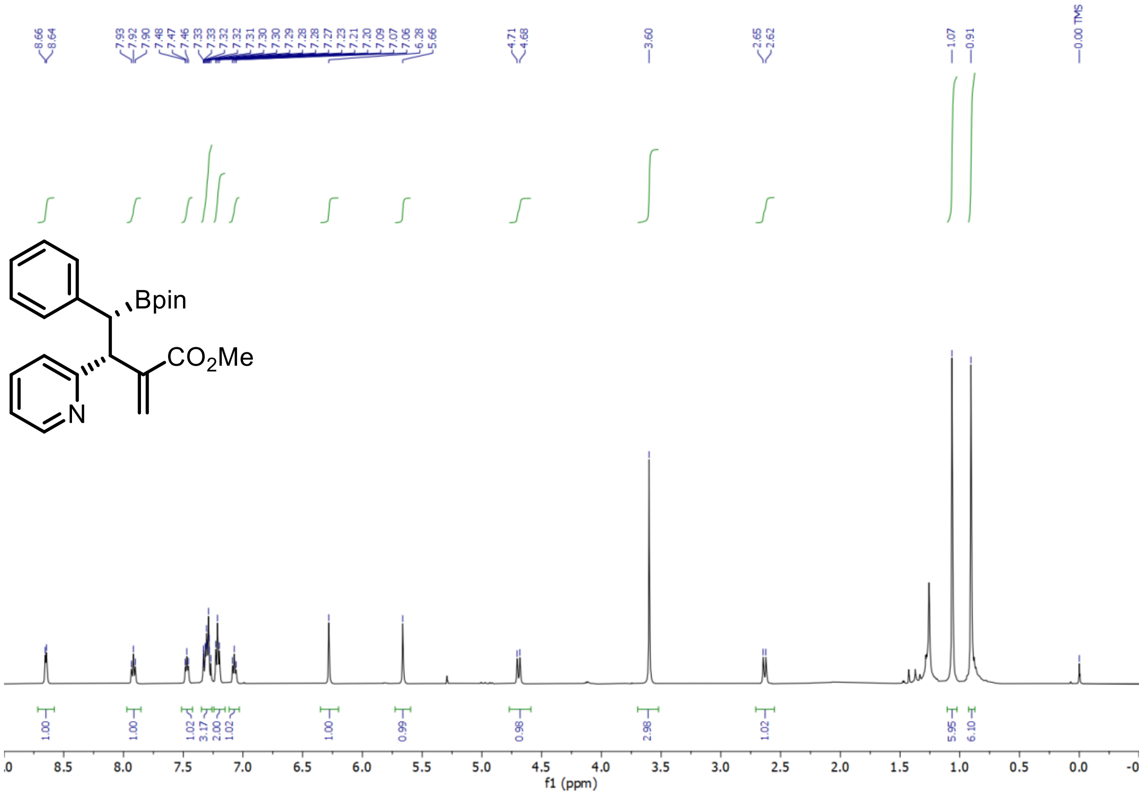

^{1}H NMR (500 MHz, $CDCl_3$) spectra of **3x’**.

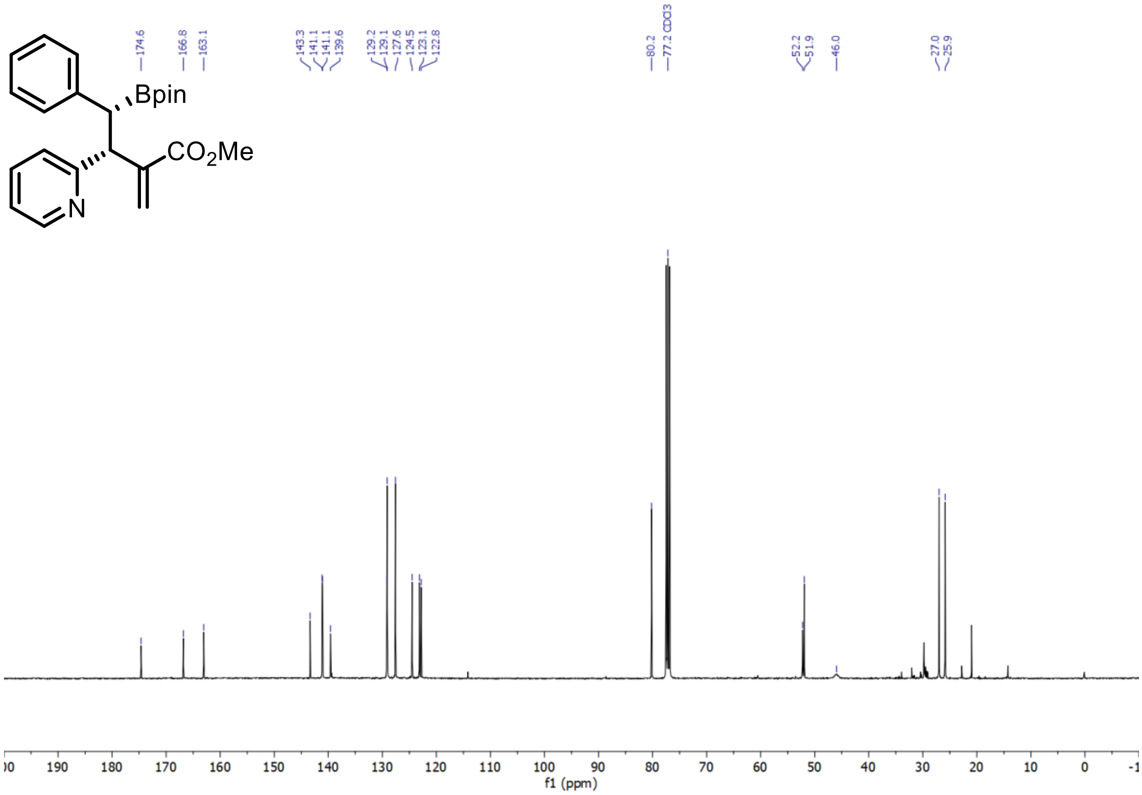

^{13}C NMR (101 MHz, $CDCl_3$) spectra of **3x’**.

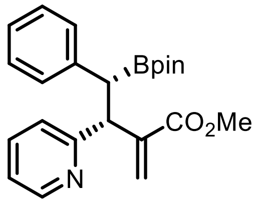

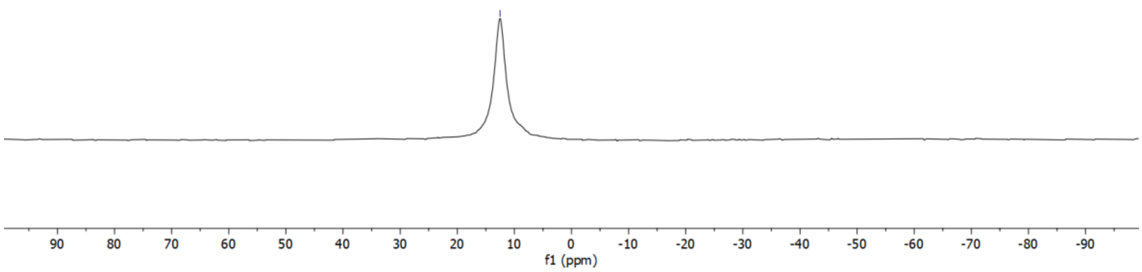

^{11}B NMR (128 MHz, $CDCl_3$) spectra of **3x’**.

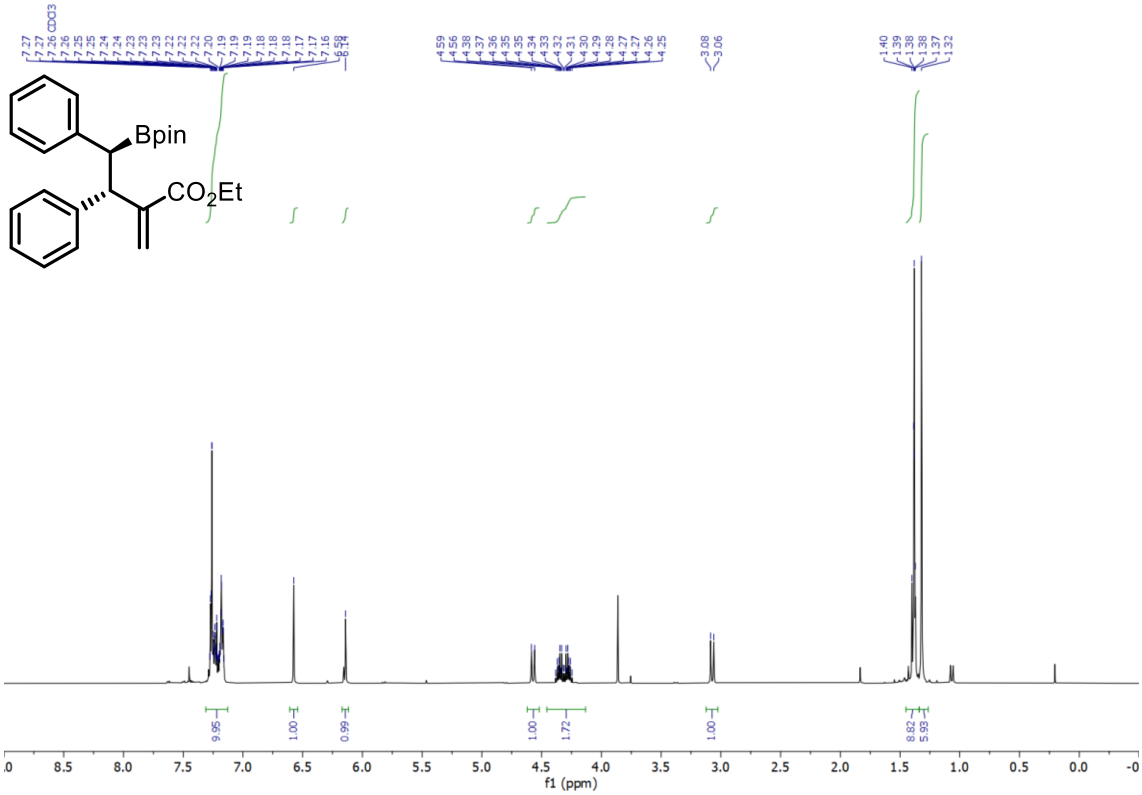

^{1}H NMR (500 MHz, $CDCl_3$) spectra of **3y**.

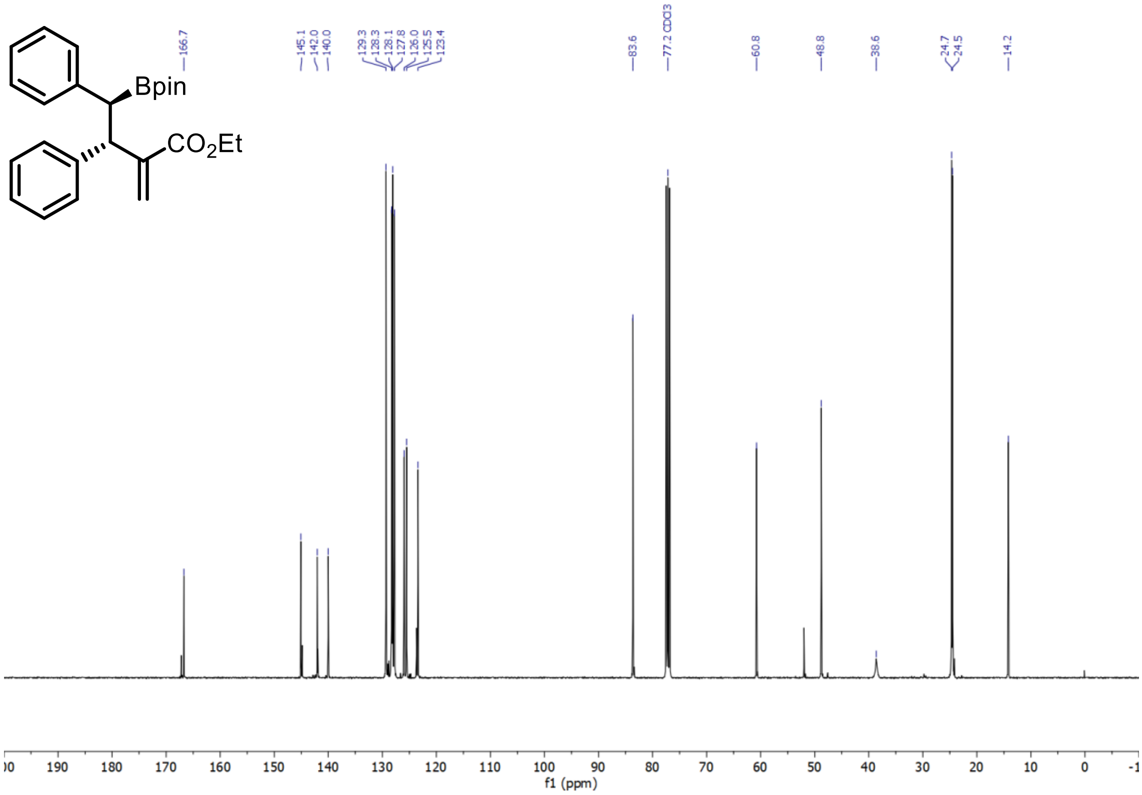

^{13}C NMR (101 MHz, $CDCl_3$) spectra of **3y**.

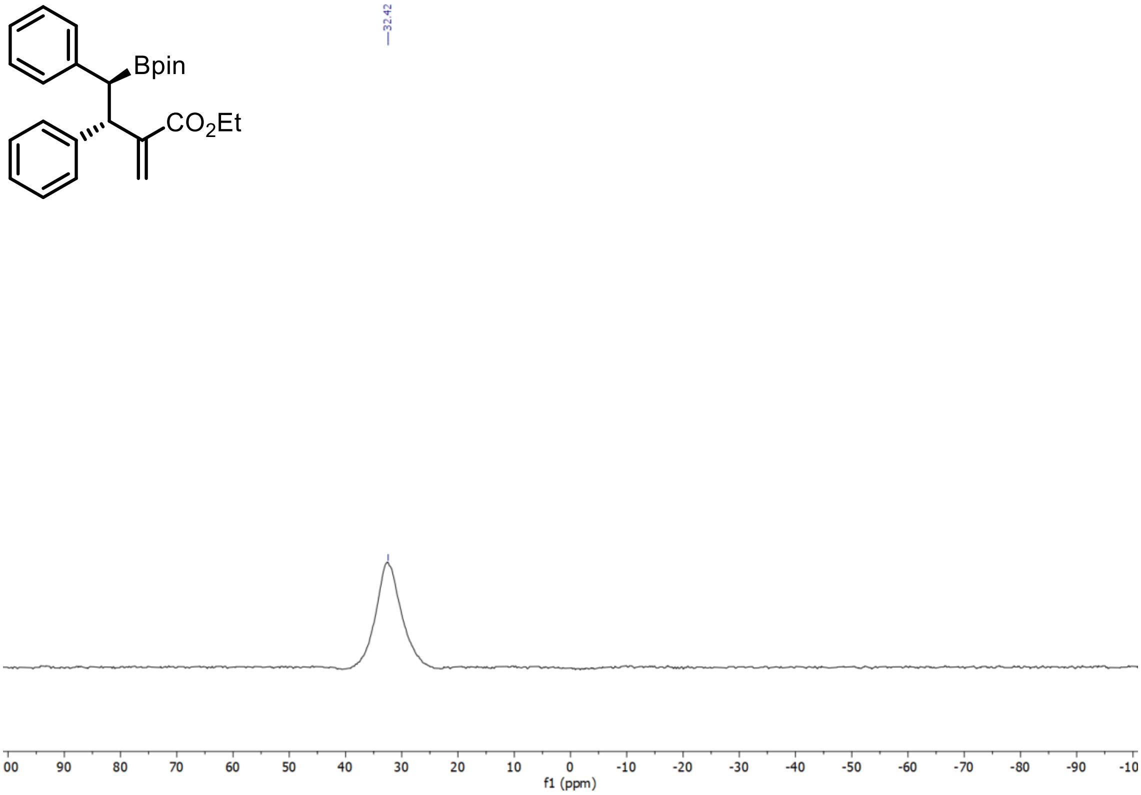

^{11}B NMR (128 MHz, $CDCl_3$) spectra of **3y**.

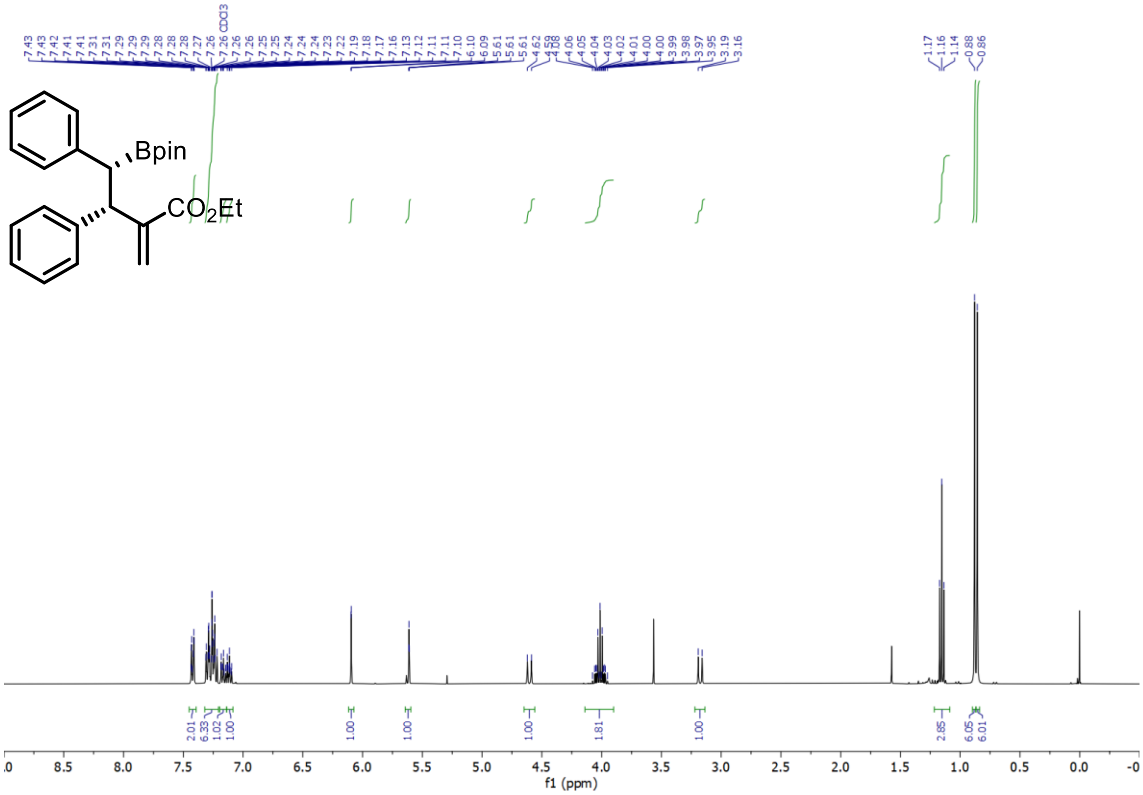

^{1}H NMR (400 MHz, $CDCl_3$) spectra of **3y'**.

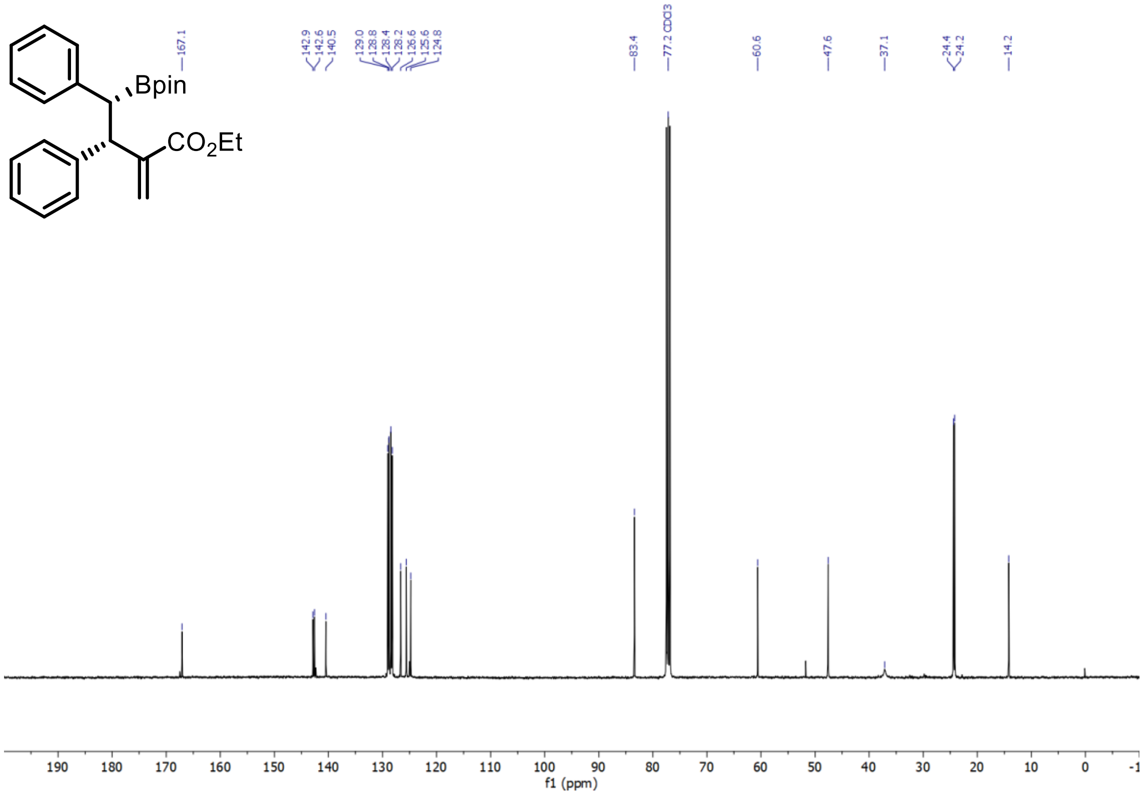

^{13}C NMR (101 MHz, $CDCl_3$) spectra of **3y'**.

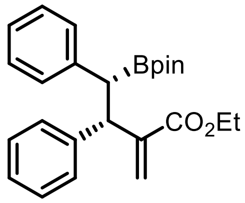

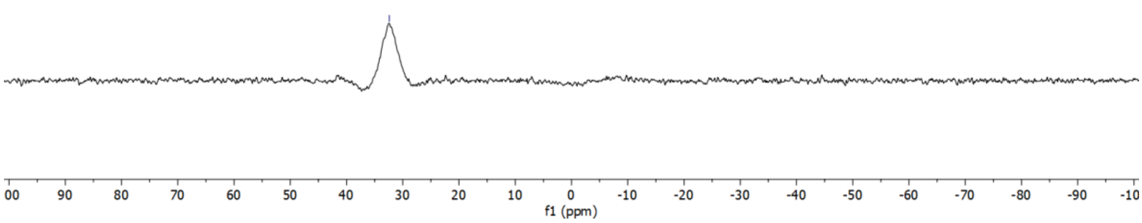

^{11}B NMR (128 MHz, $CDCl_3$) spectra of **3y'**.

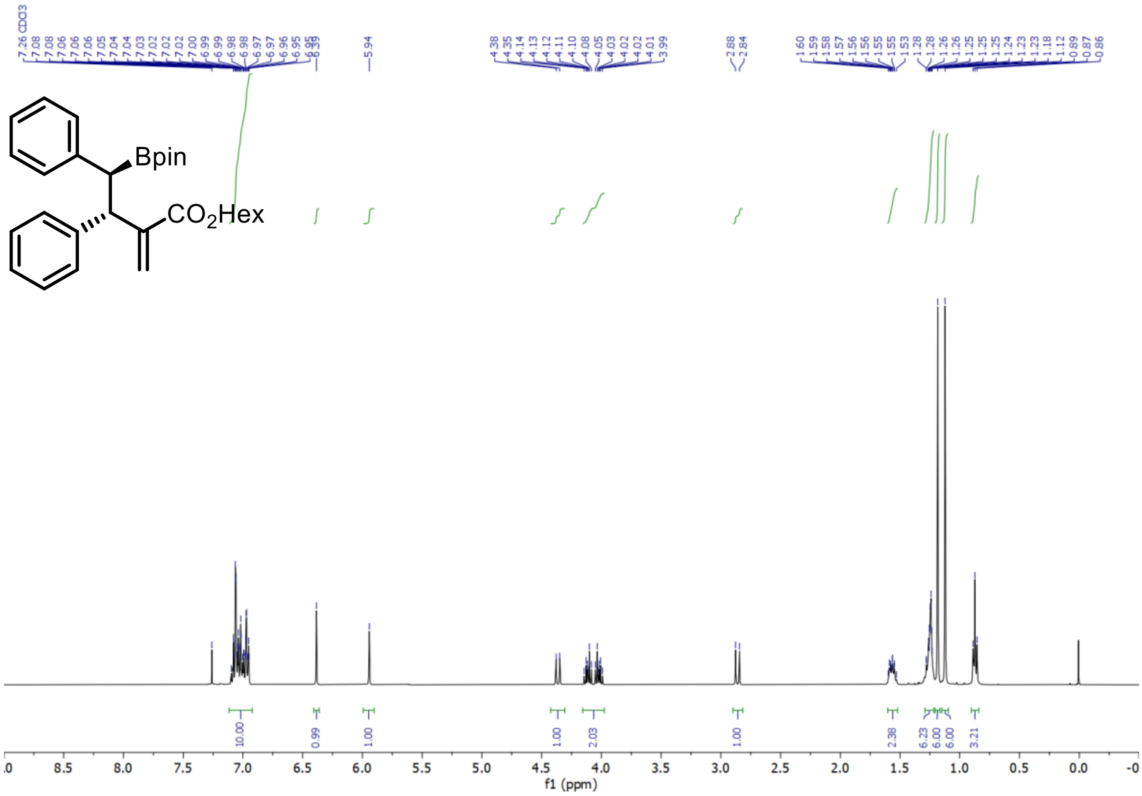

^{1}H NMR (400 MHz, $CDCl_3$) spectra of **3z**.

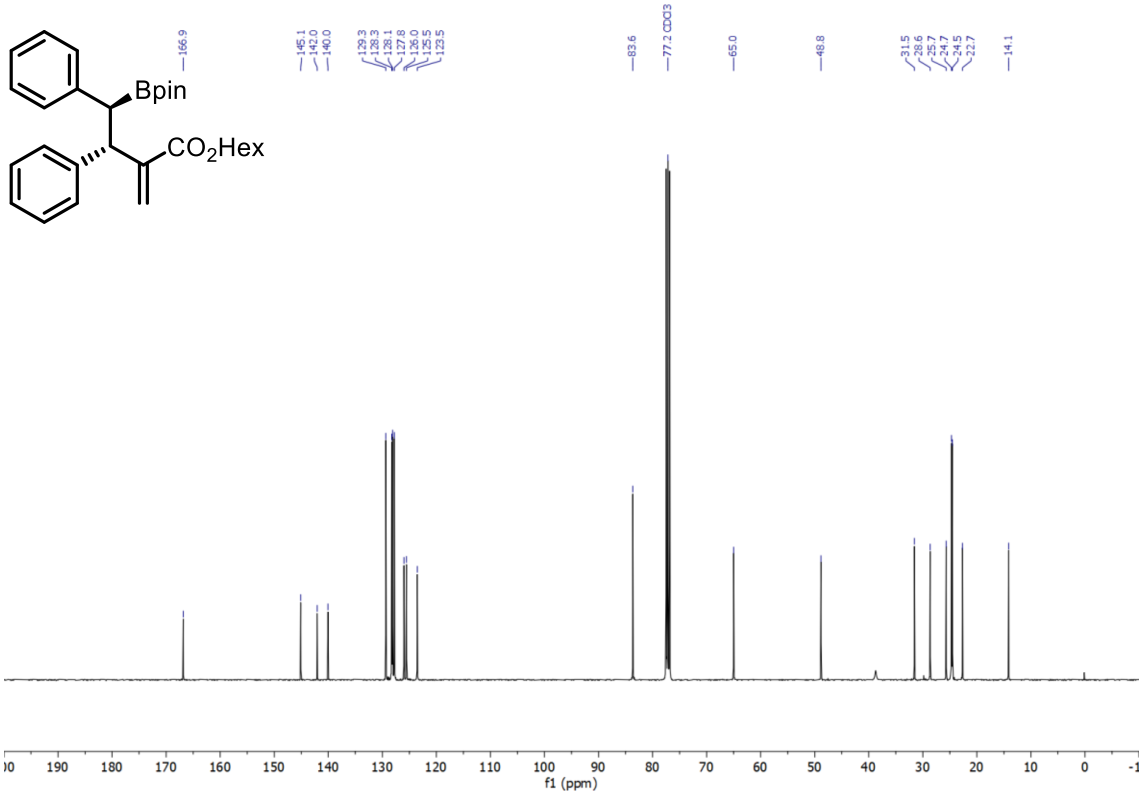

^{13}C NMR (101 MHz, $CDCl_3$) spectra of **3z**.

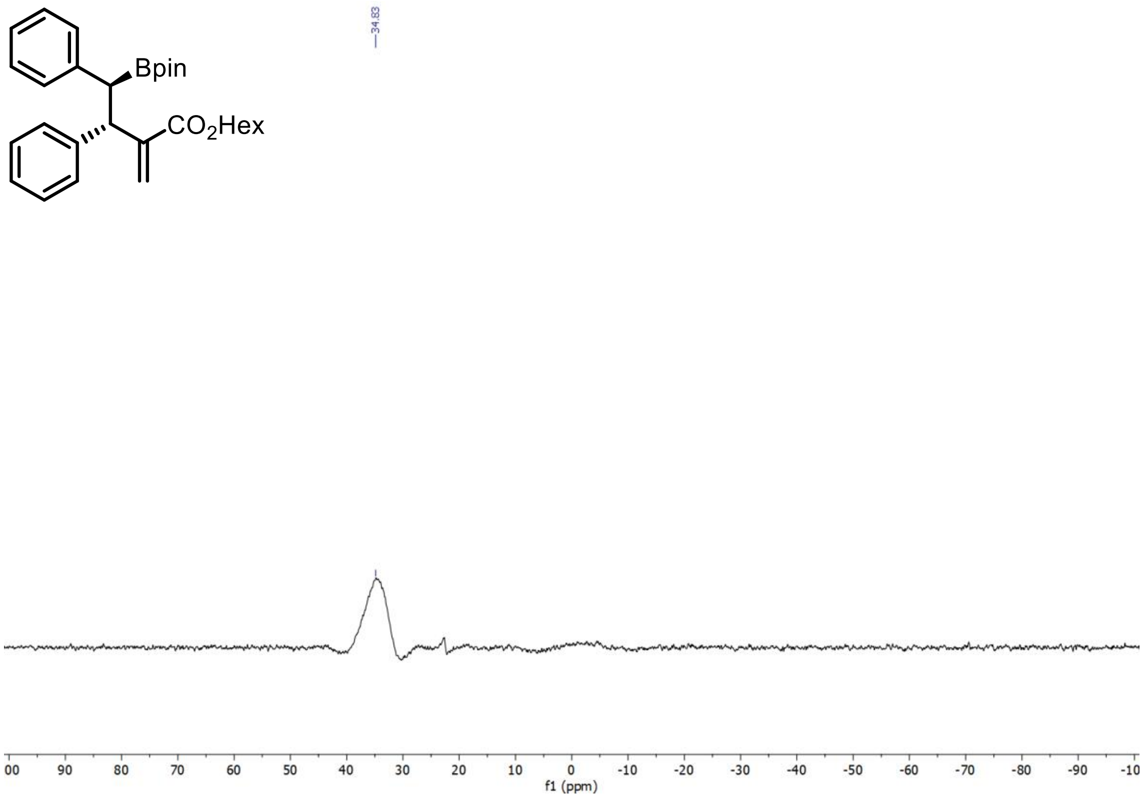

^{11}B NMR (128 MHz, $CDCl_3$) spectra of **3z**.

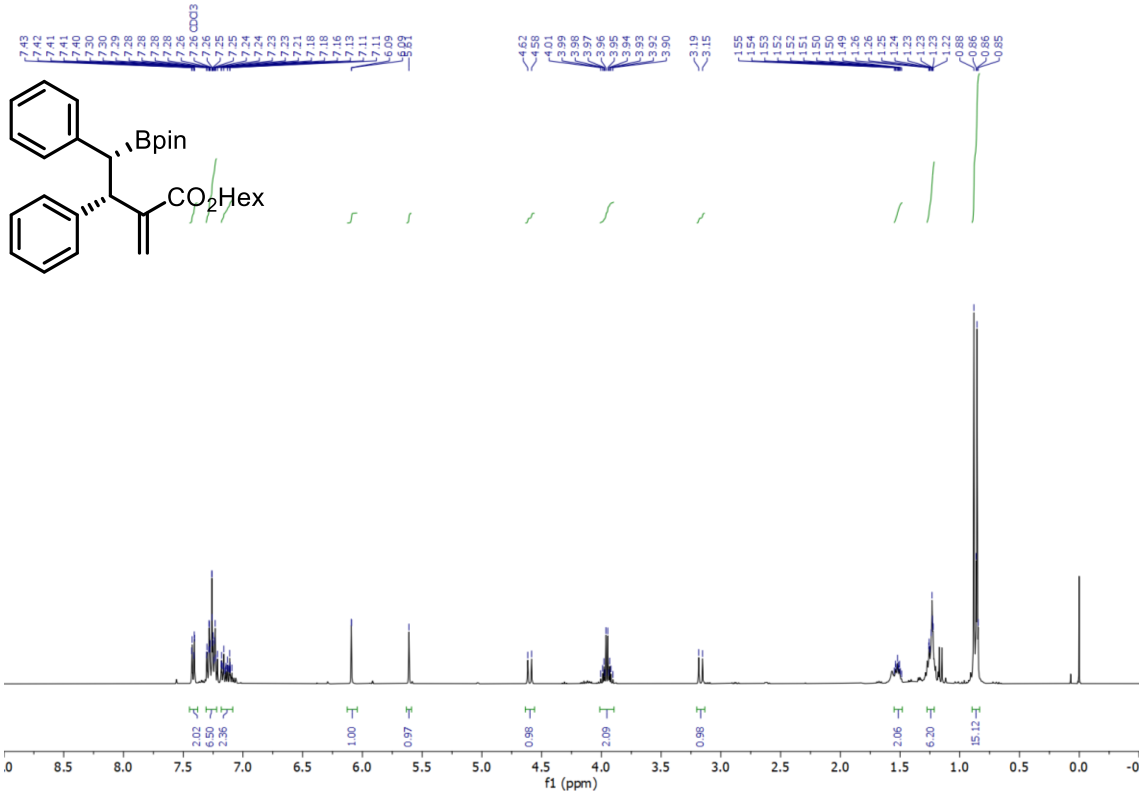

^{1}H NMR (400 MHz, $CDCl_3$) spectra of **3z’**.

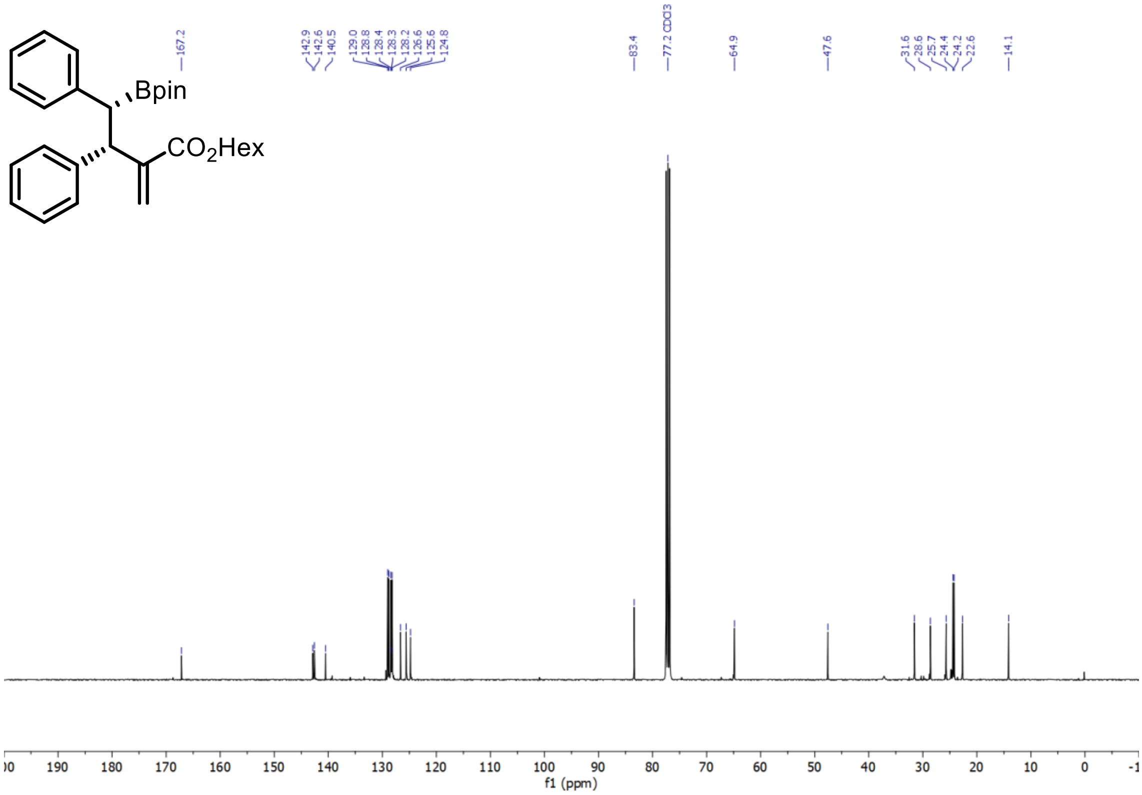

^{13}C NMR (101 MHz, $CDCl_3$) spectra of **3z’**.

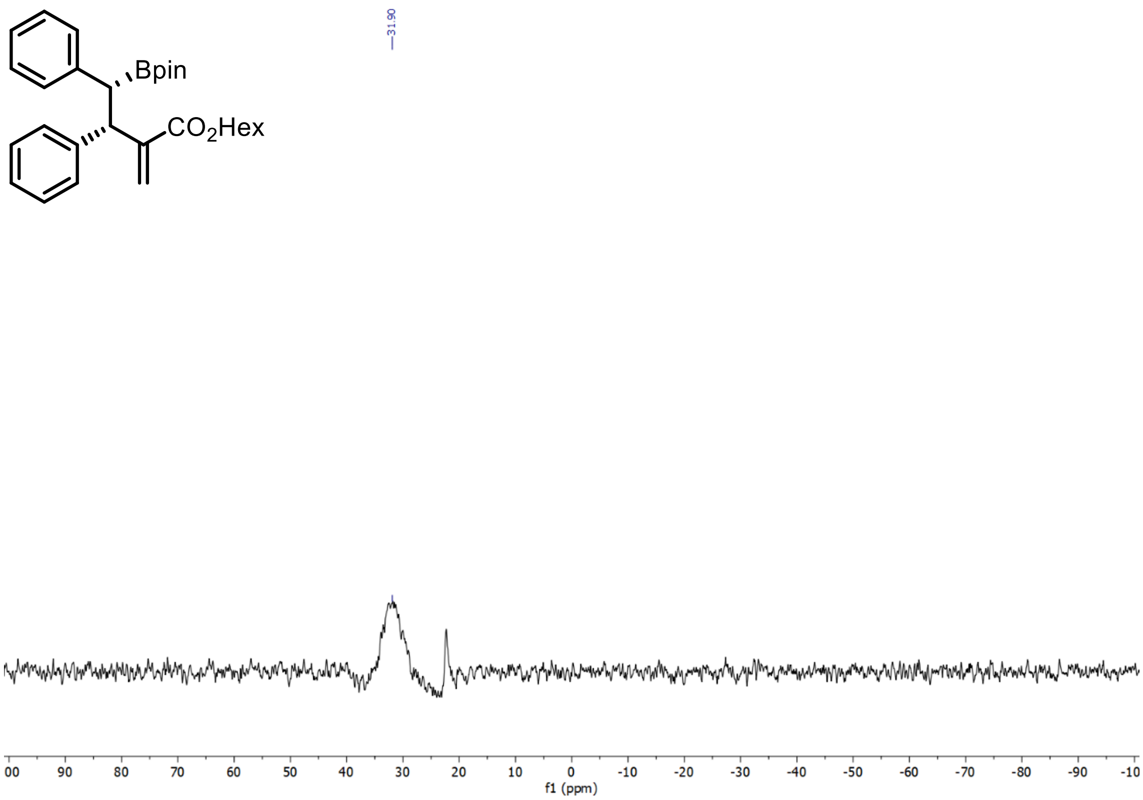

^{11}B NMR (128 MHz, $CDCl_3$) spectra of **3z’**.

^{1}H NMR (400 MHz, $CDCl_3$) spectra of **6a**.

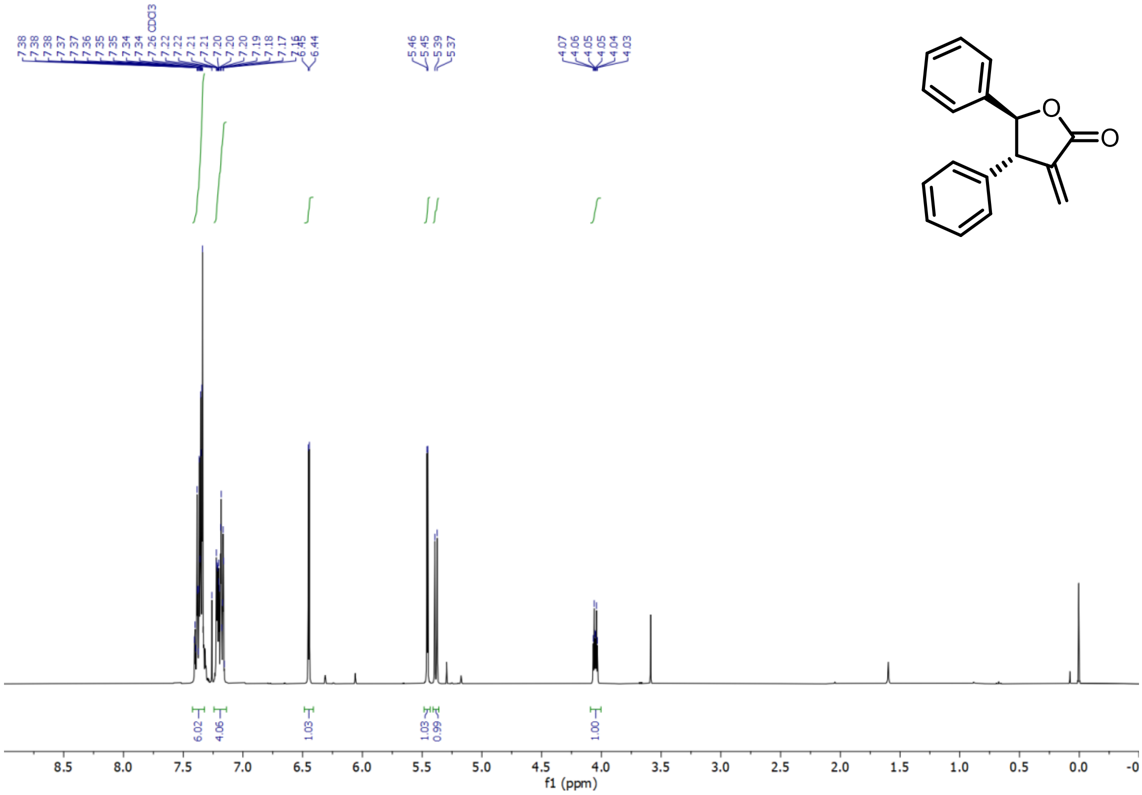

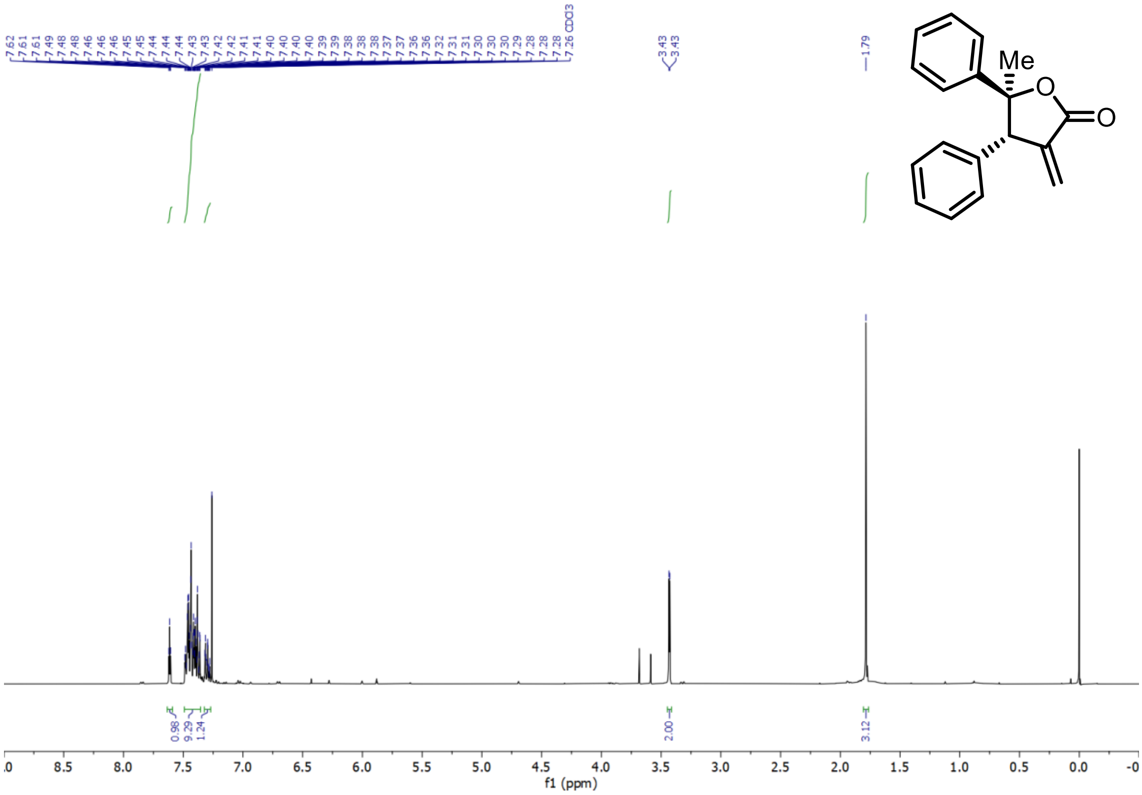

^{1}H NMR (400 MHz, $CDCl_3$) spectra of **6b**.

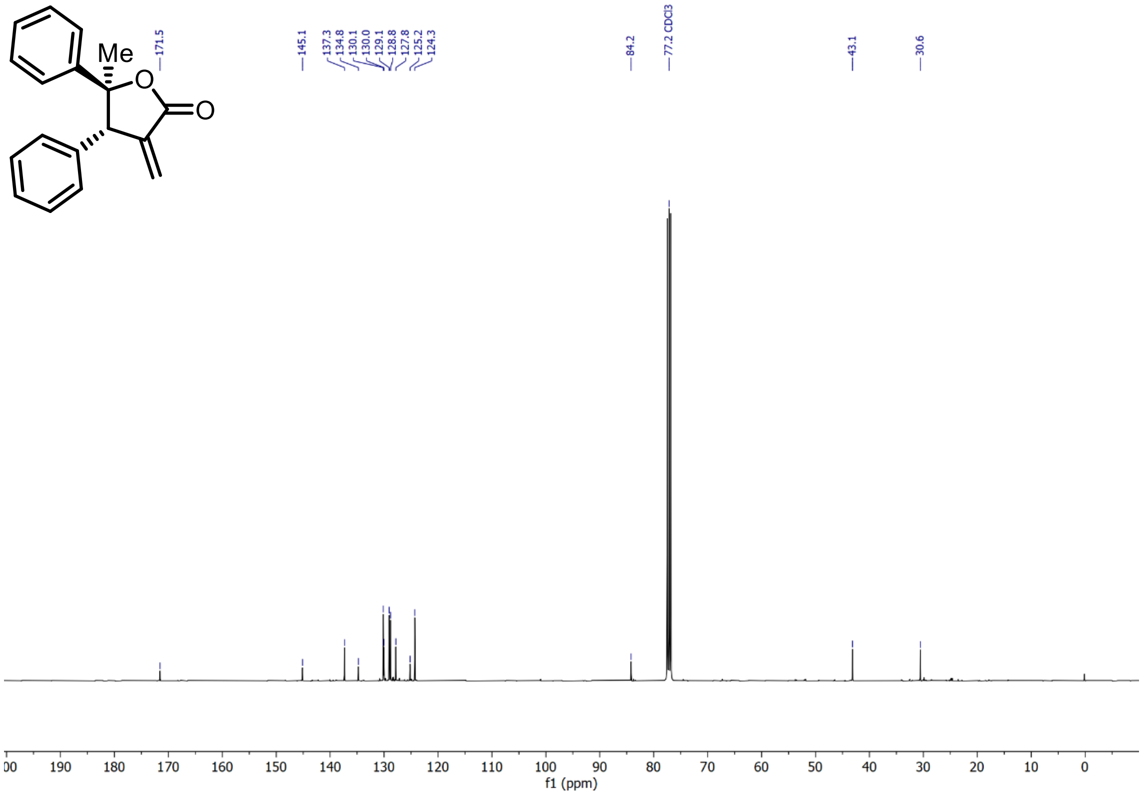

^{13}C NMR (101 MHz, $CDCl_3$) spectra of **6b**.

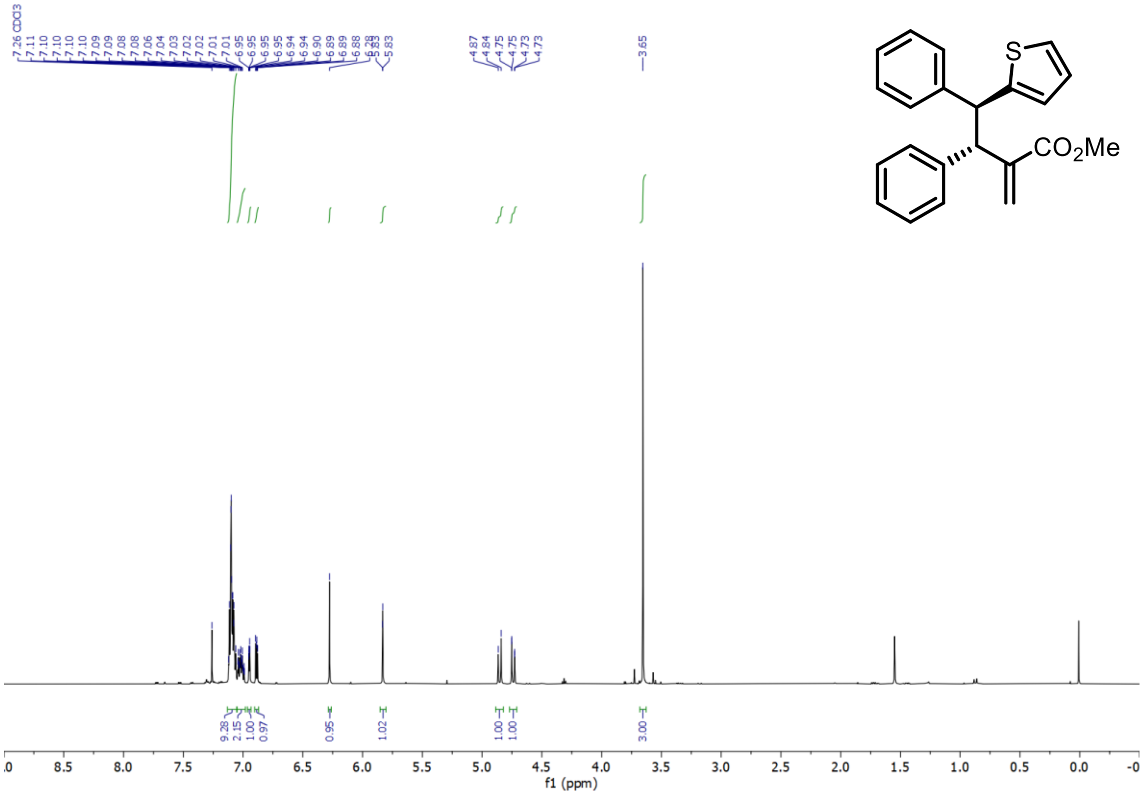

^{1}H NMR (500 MHz, $CDCl_3$) spectra of **7a**.

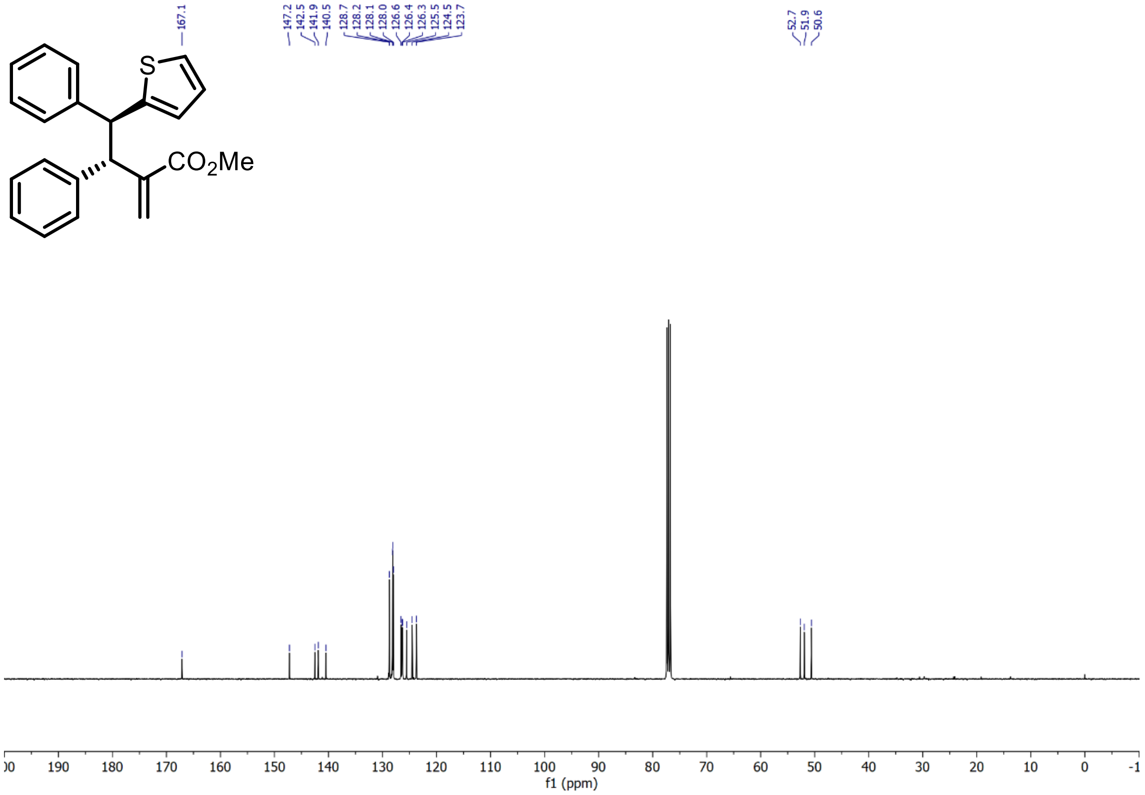

^{13}C NMR (101 MHz, $CDCl_3$) spectra of **7a**.

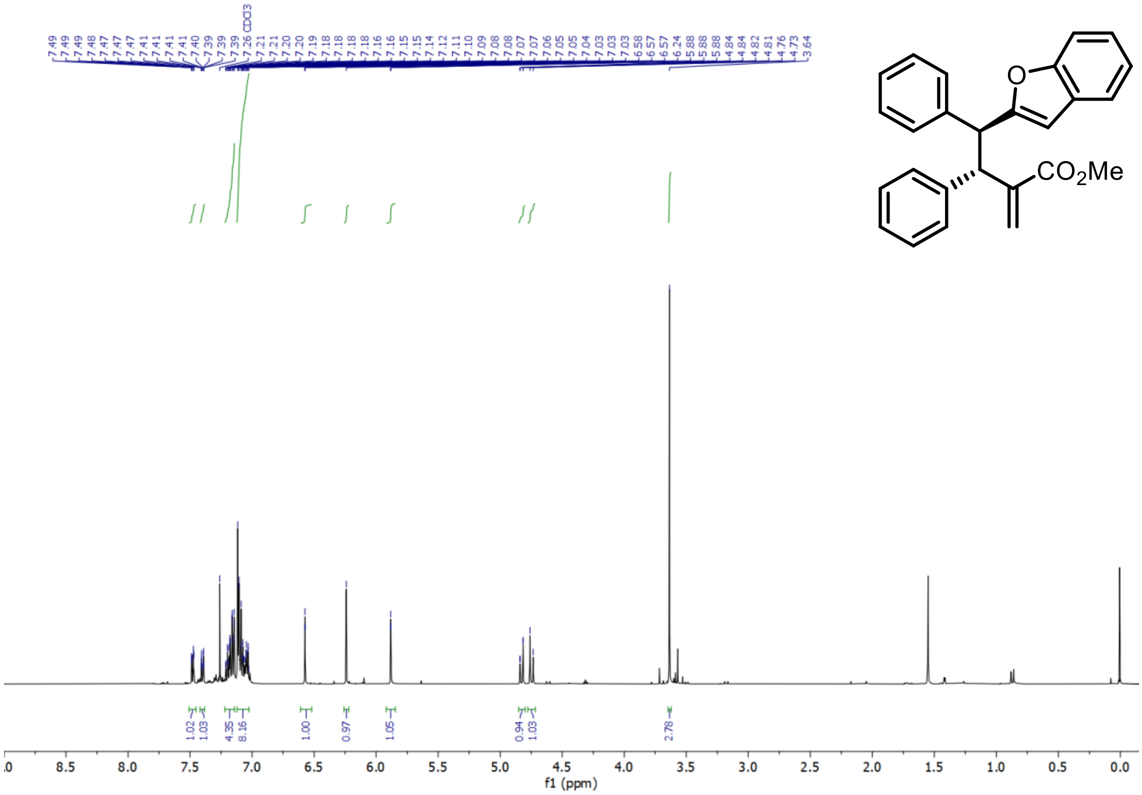

^{1}H NMR (500 MHz, $CDCl_3$) spectra of **7b**.

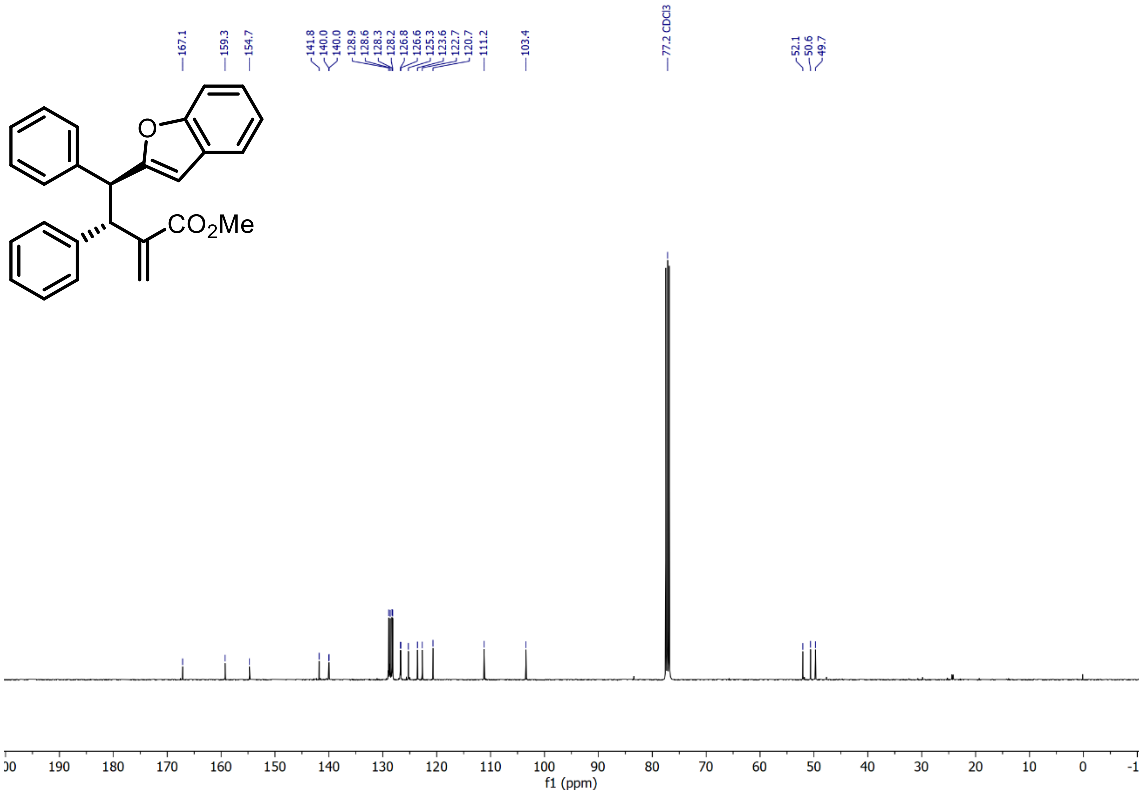

^{13}C NMR (101 MHz, $CDCl_3$) spectra of **7b**.

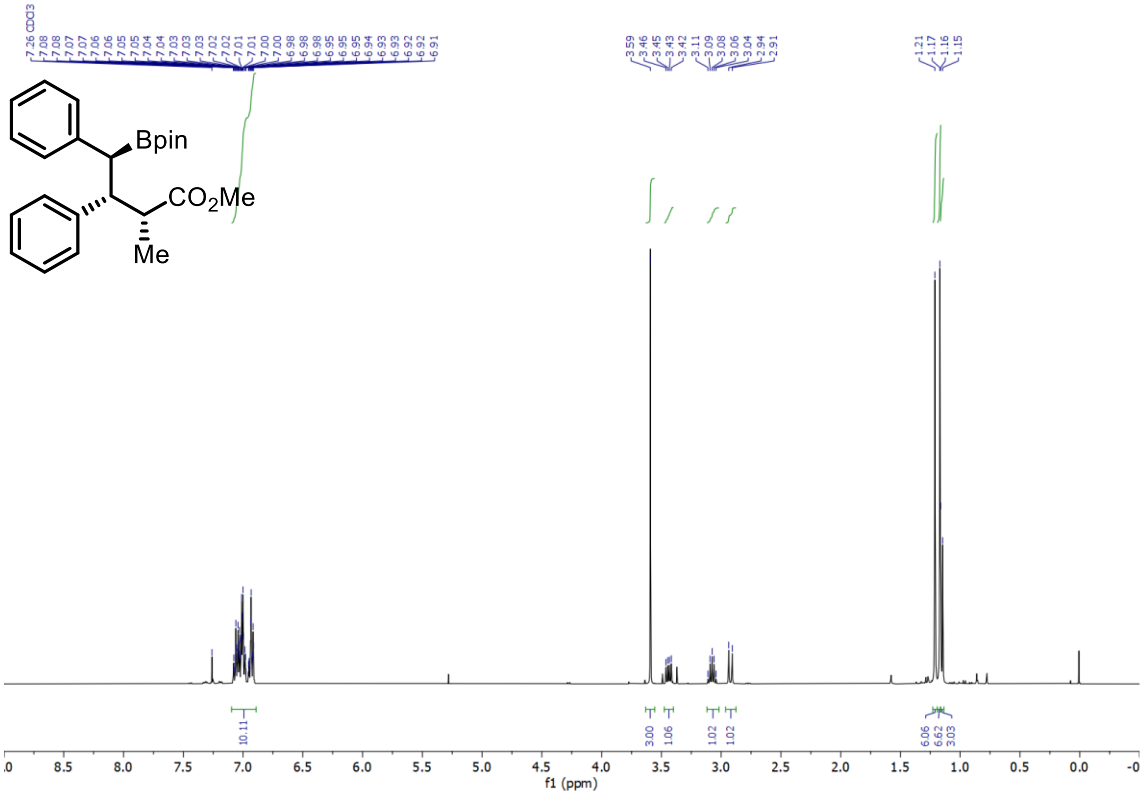

^{1}H NMR (400 MHz, $CDCl_3$) spectra of **8**.

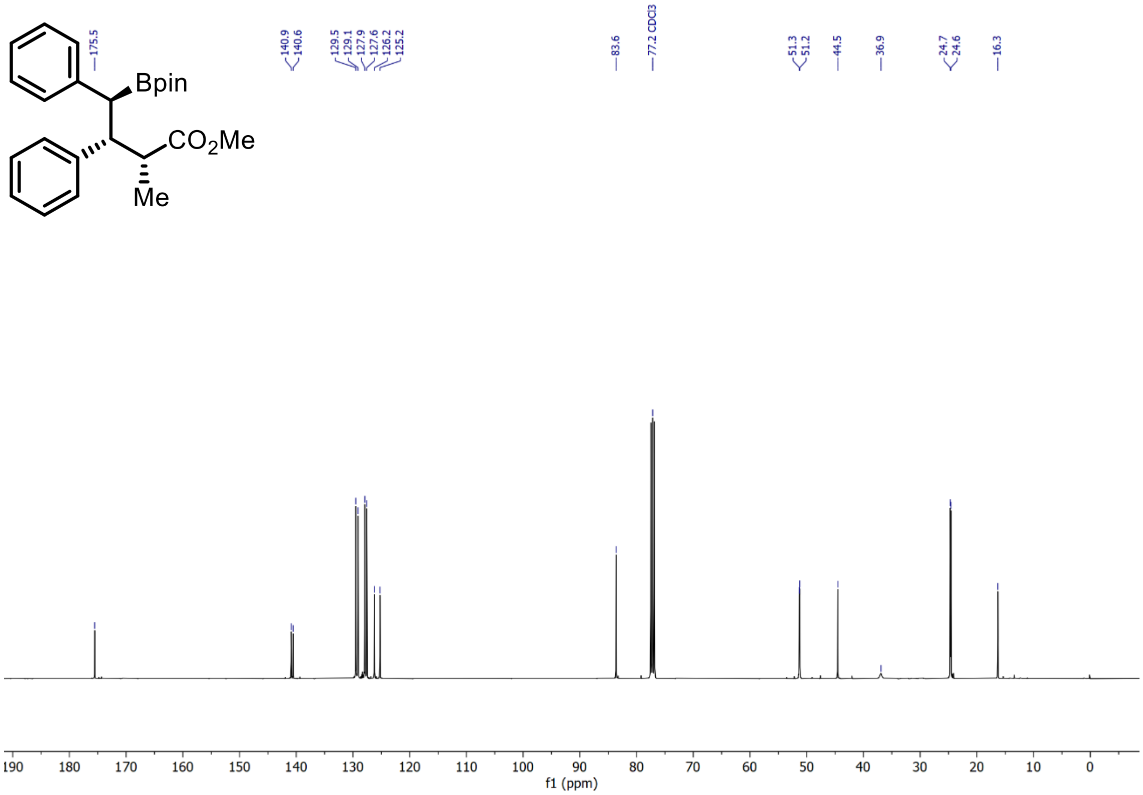

^{13}C NMR (101 MHz, $CDCl_3$) spectra of **8**.

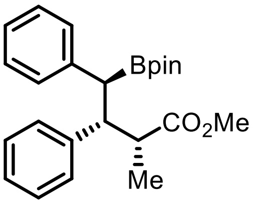

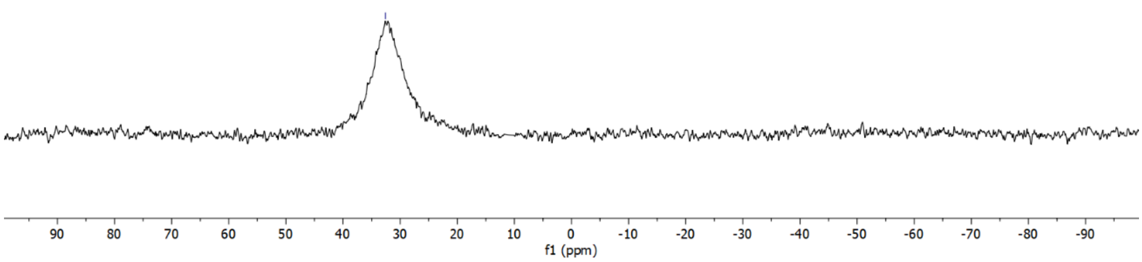

^{11}B NMR (128 MHz, $CDCl_3$) spectra of **8**.

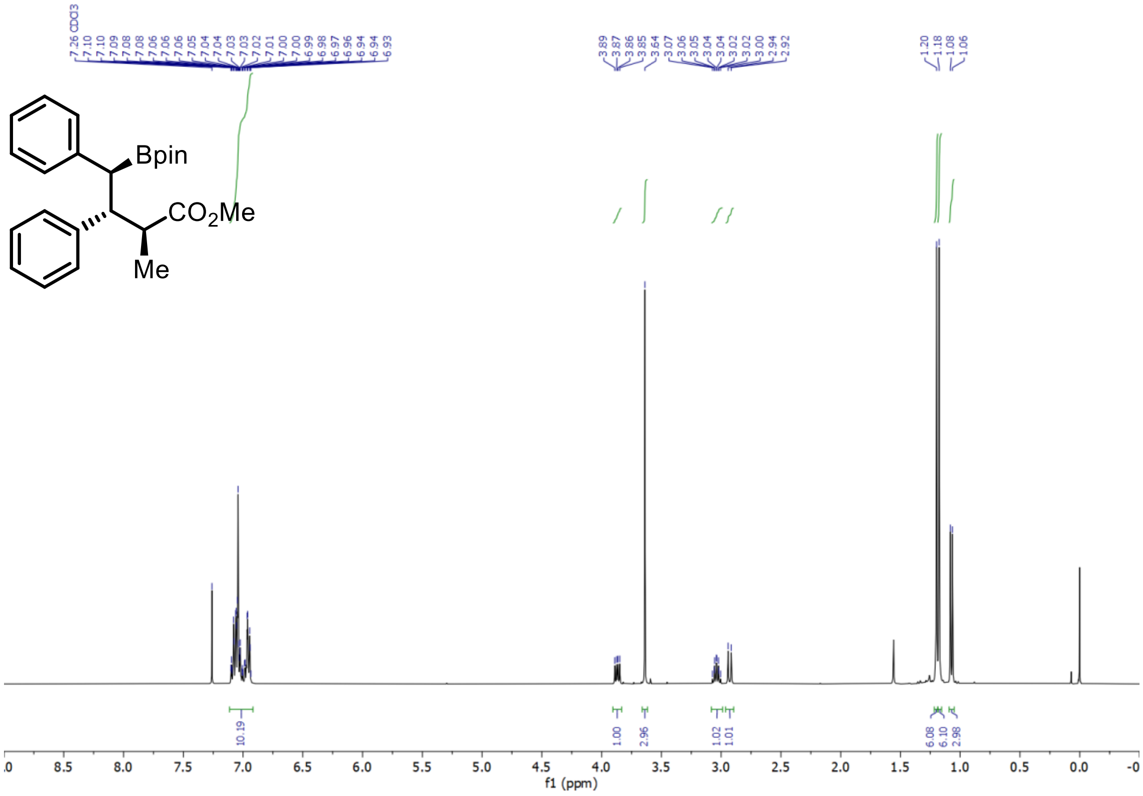

^{1}H NMR (400 MHz, $CDCl_3$) spectra of **8’**.

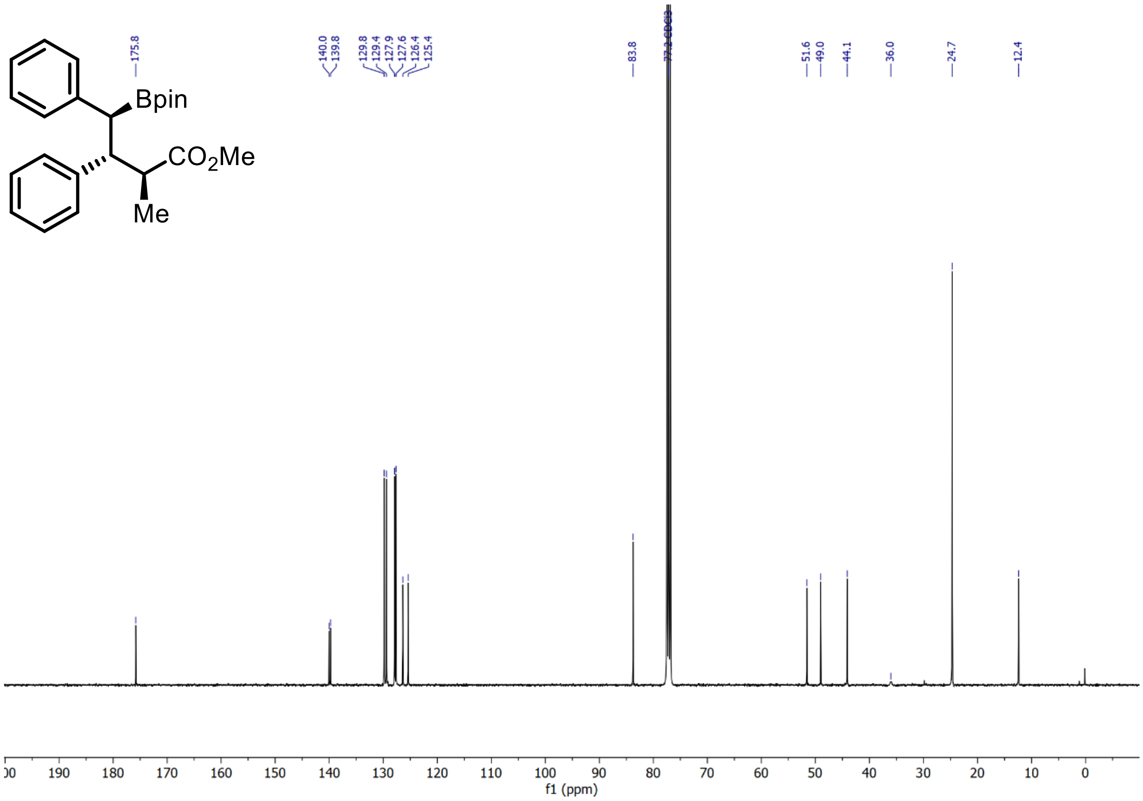

^{13}C NMR (101 MHz, $CDCl_3$) spectra of **8’**.

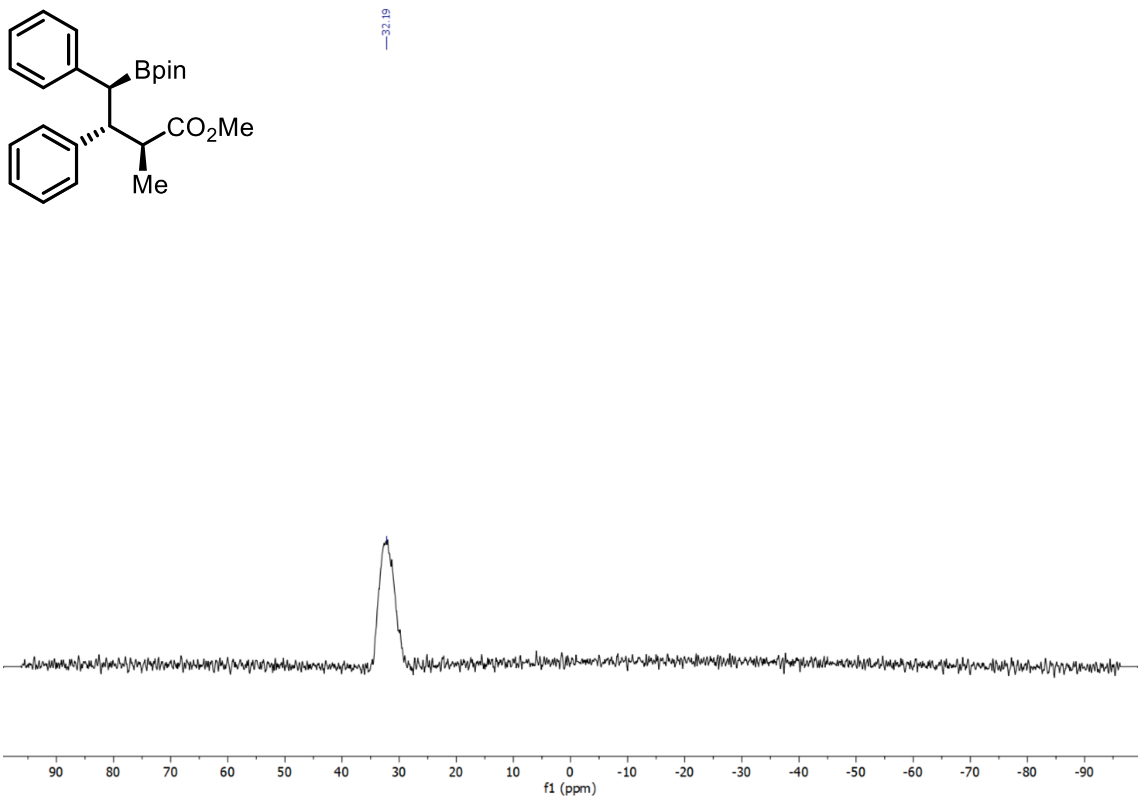

^{11}B NMR (128 MHz, $CDCl_3$) spectra of **8’**.

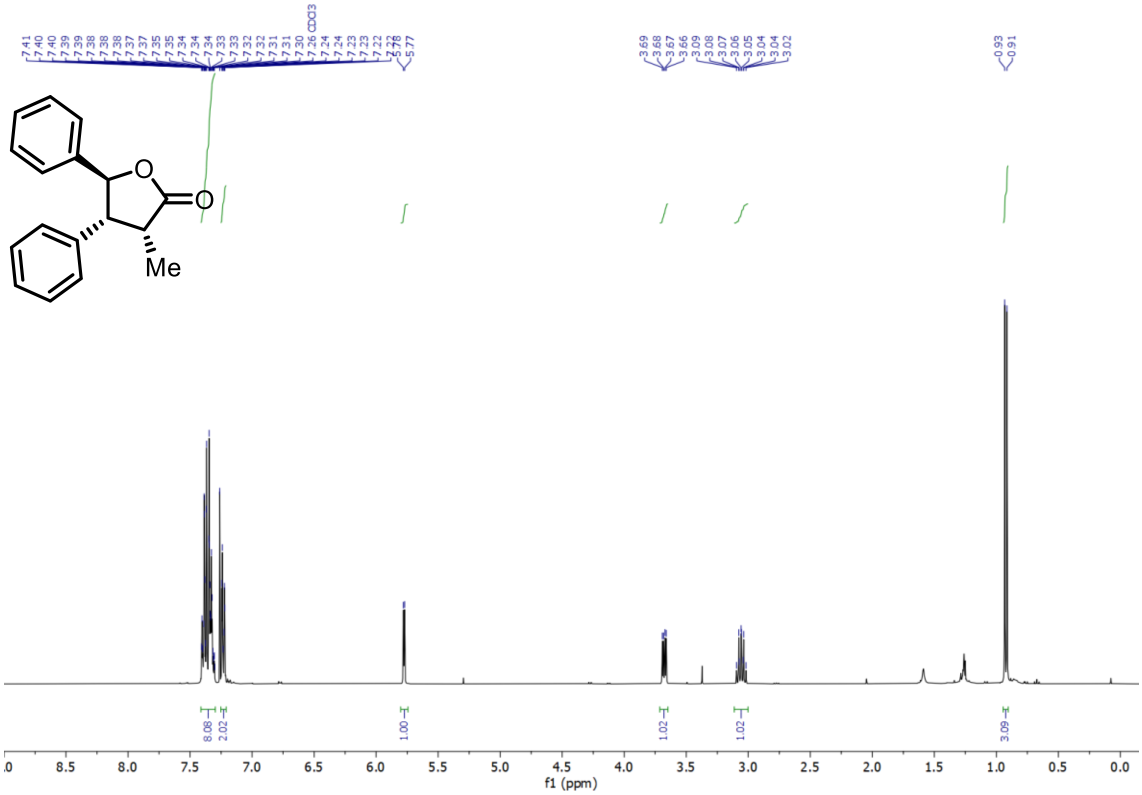

^{1}H NMR (400 MHz, $CDCl_3$) spectra of **9**.

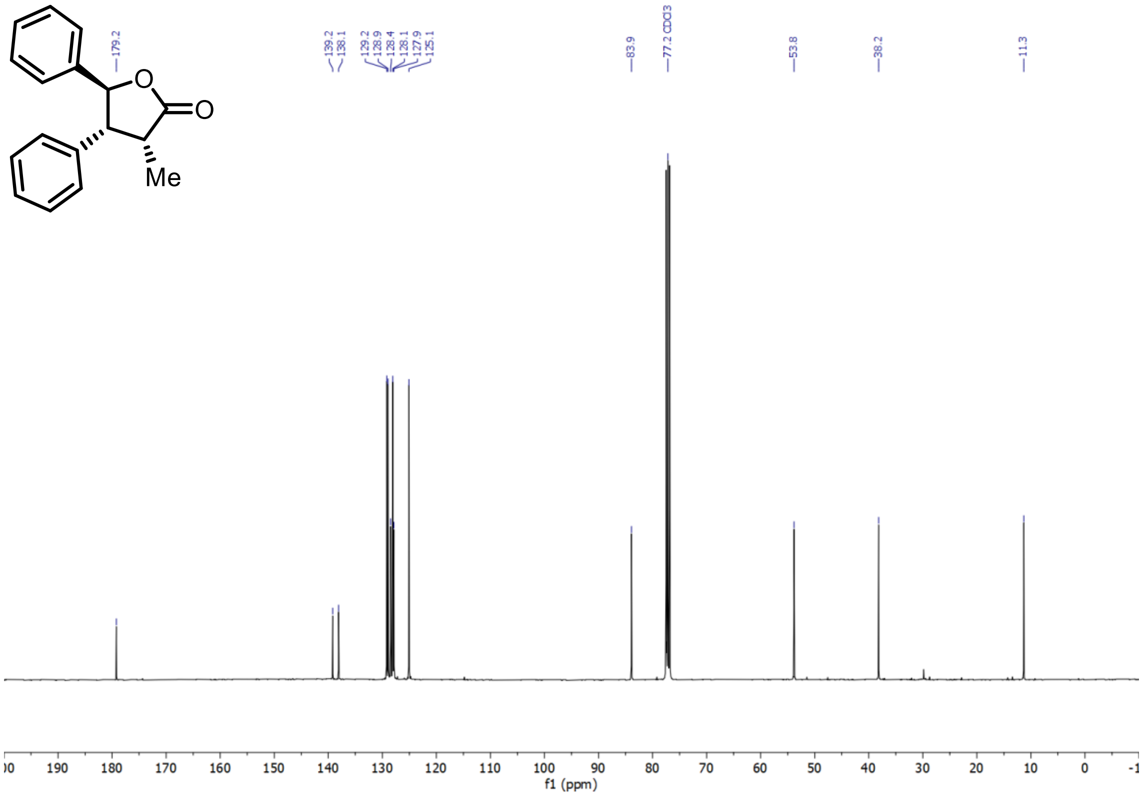

^{13}C NMR (101 MHz, $CDCl_3$) spectra of **9**.

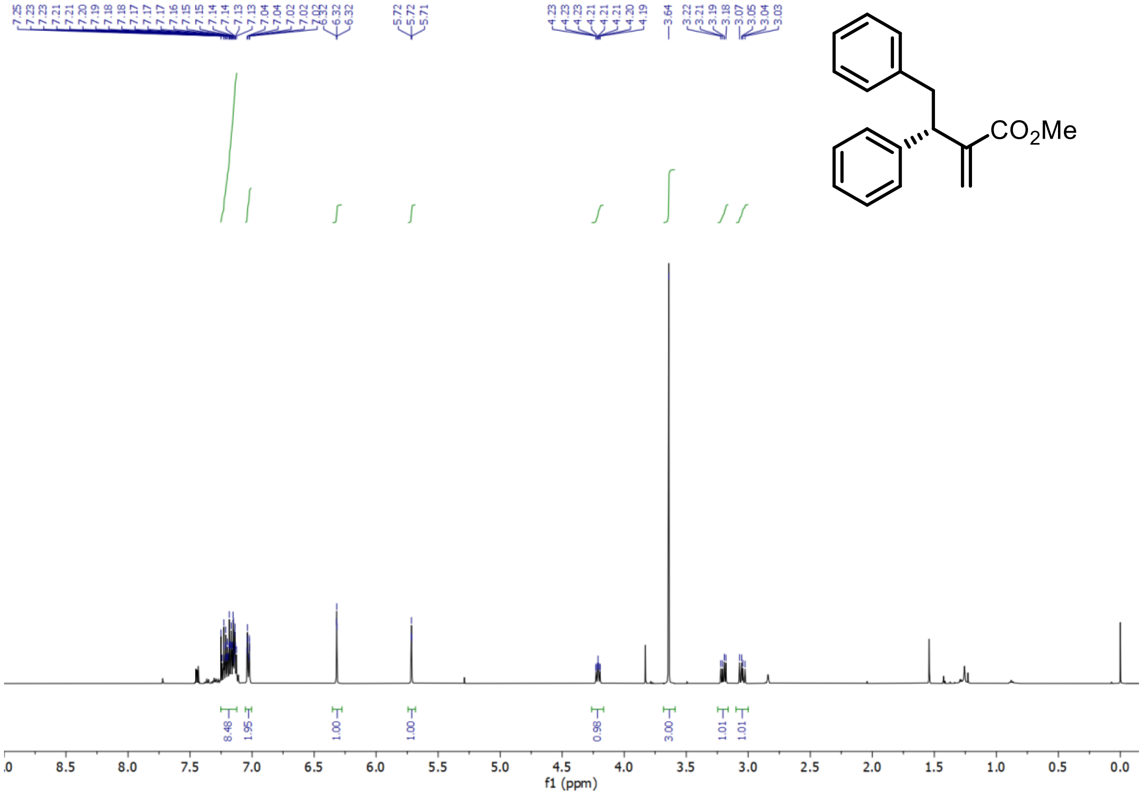

^{1}H NMR (500 MHz, CDCl$_3$) spectra of **10**.

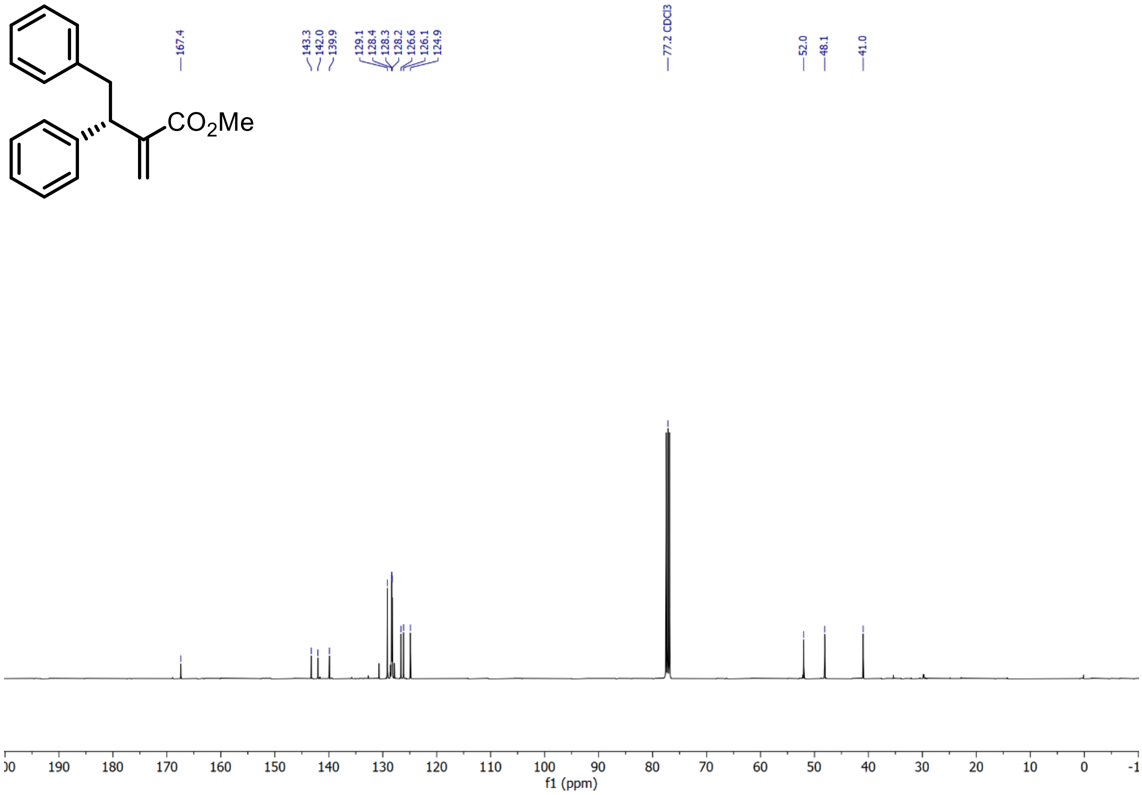

^{13}C NMR (101 MHz, CDCl$_3$) spectra of **10**.

L. Chiral HPLC Chromatograms

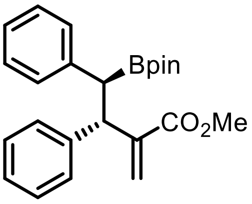

3a

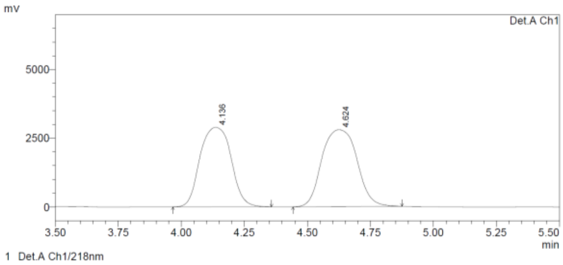

PeakTable

Detector A Ch1 218nm

Peak#	Ret. Time	Area	Height	Area %	Height %
1	4.136	24823811	2881150	47.780	50.846
2	4.624	27130219	2785314	52.220	49.154
Total		51954029	5666464	100.000	100.000

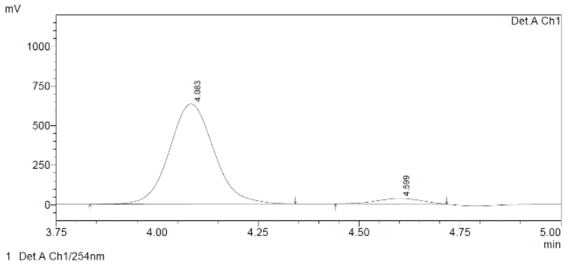

PeakTable

Detector A Ch1 254nm

Peak#	Ret. Time	Area	Height	Area %	Height %
1	4.083	4832382	627901	94.761	94.686
2	4.599	267158	35238	5.239	5.314
Total		5099541	663139	100.000	100.000

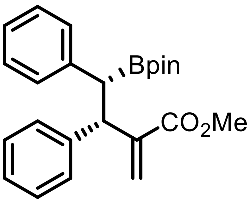

3a’

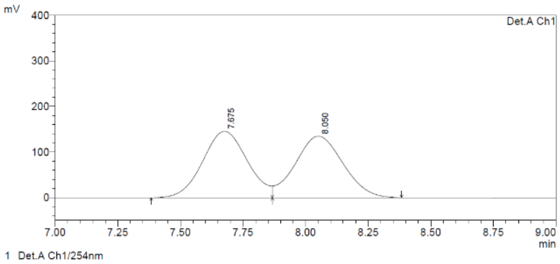

PeakTable

Detector A Ch1 254nm

Peak#	Ret. Time	Area	Height	Area %	Height %
1	7.675	1804968	145284	50.181	51.861
2	8.050	1791981	134857	49.819	48.139
Total		3596948	280141	100.000	100.000

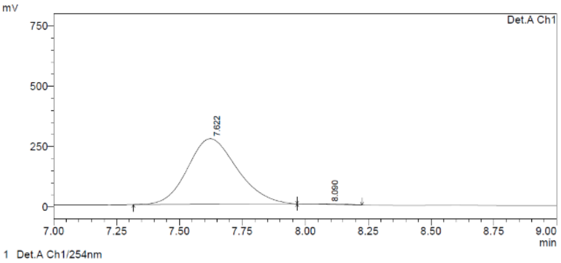

PeakTable

Detector A Ch1 254nm

Peak#	Ret. Time	Area	Height	Area %	Height %
1	7.622	3738255	271019	99.637	99.199
2	8.090	13619	2187	0.363	0.801
Total		3751874	273206	100.000	100.000

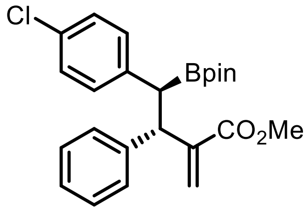

3b’

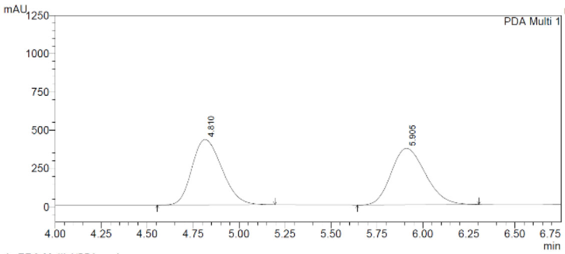

1 PDA Multi 1/254nm 4nm

PeakTable

PDA Ch1 254nm 4nm

Peak#	Ret. Time	Area	Height	Area %	Height %
1	4.810	4828817	424939	50.321	53.668
2	5.905	4767124	366851	49.679	46.332
Total		9595941	791789	100.000	100.000

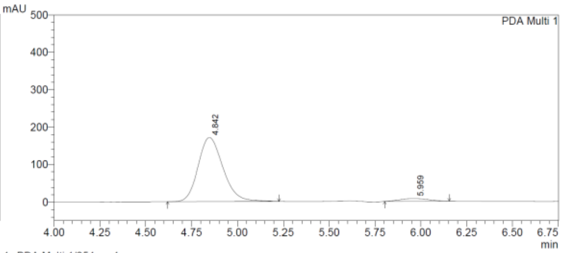

1 PDA Multi 1/254nm 4nm

PeakTable

PDA Ch1 254nm 4nm

Peak#	Ret. Time	Area	Height	Area %	Height %
1	4.842	1608338	170275	95.318	95.767
2	5.959	79010	7527	4.682	4.233
Total		1687348	177802	100.000	100.000

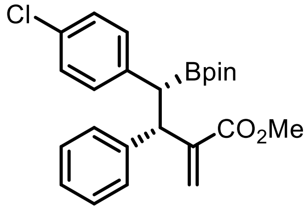

3b’

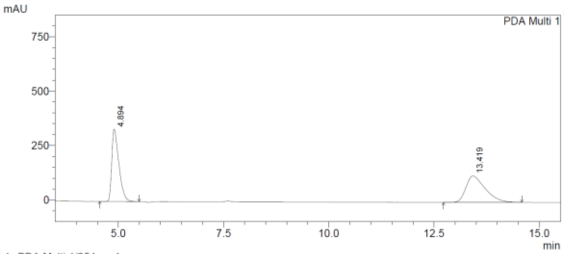

1 PDA Multi 1/254nm 4nm

PeakTable

PDA Ch1 254nm 4nm

Peak#	Ret. Time	Area	Height	Area %	Height %
1	4.894	3895450	331757	50.088	73.158
2	13.419	3881707	121724	49.912	26.842
Total		7777157	453480	100.000	100.000

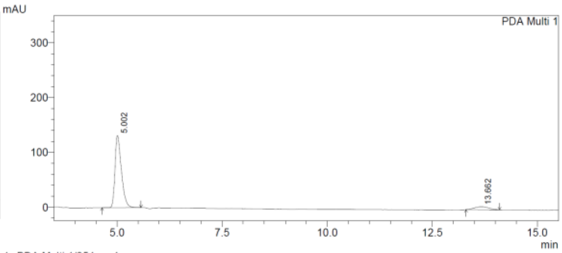

1 PDA Multi 1/254nm 4nm

PeakTable

PDA Ch1 254nm 4nm

Peak#	Ret. Time	Area	Height	Area %	Height %
1	5.002	1400046	131465	91.301	95.925
2	13.662	133401	5585	8.699	4.075
Total		1533447	137050	100.000	100.000

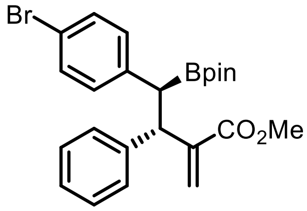

3c

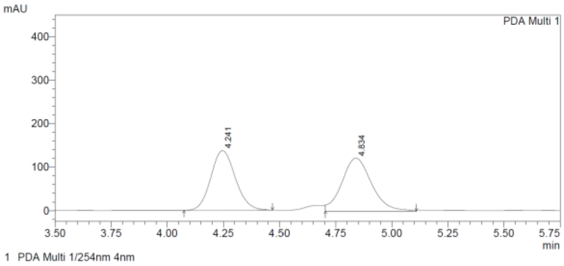

1 PDA Multi 1/254nm 4nm

PeakTable

PDA Ch1 254nm 4nm

Peak#	Ret. Time	Area	Height	Area %	Height %
1	4.241	1042236	137067	48.496	53.141
2	4.834	1106891	120865	51.504	46.859
Total		2149127	257932	100.000	100.000

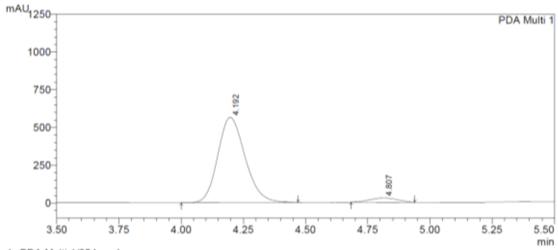

1 PDA Multi 1/254nm 4nm

PeakTable

PDA Ch1 254nm 4nm

Peak#	Ret. Time	Area	Height	Area %	Height %
1	4.192	4309480	563313	95.200	95.239
2	4.807	217265	28162	4.800	4.761
Total		4526746	591475	100.000	100.000

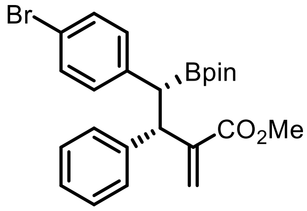

3c’

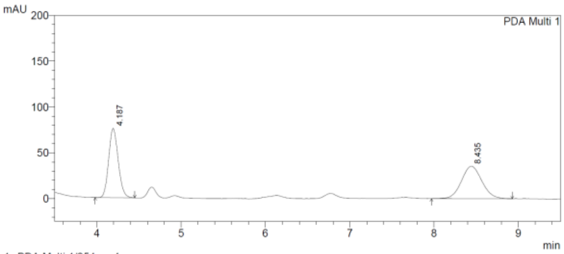

1 PDA Multi 1/254nm 4nm

PeakTable

PDA Ch1 254nm 4nm

Peak#	Ret. Time	Area	Height	Area %	Height %
1	4.187	595694	75743	49.867	68.144
2	8.435	598876	35408	50.133	31.856
Total		1194570	111152	100.000	100.000

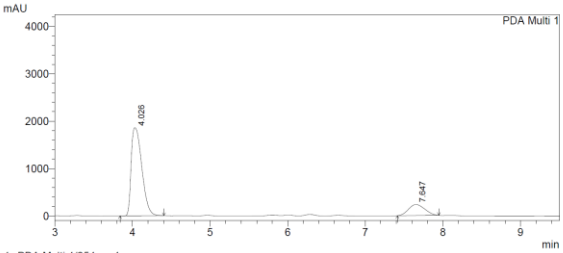

1 PDA Multi 1/254nm 4nm

PeakTable

PDA Ch1 254nm 4nm

Peak#	Ret. Time	Area	Height	Area %	Height %
1	4.026	17659381	1862150	83.858	88.946
2	7.647	3399368	231415	16.142	11.054
Total		21058749	2093565	100.000	100.000

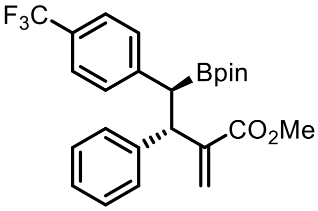

3d

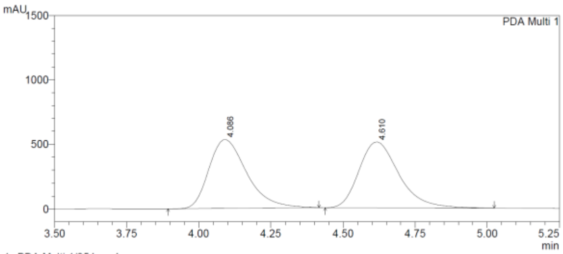

1 PDA Multi 1/254nm 4nm

PeakTable

PDA Ch1 254nm 4nm

Peak#	Ret. Time	Area	Height	Area %	Height %
1	4.086	4864076	529577	49.761	50.965
2	4.610	4910827	509521	50.239	49.035
Total		9774903	1039098	100.000	100.000

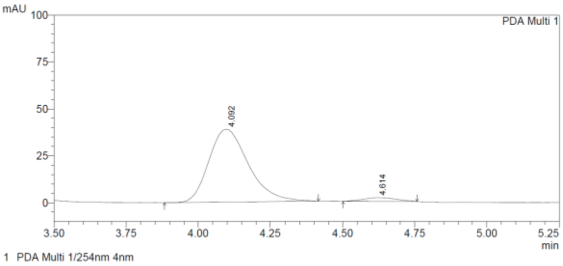

1 PDA Multi 1/254nm 4nm

PeakTable

PDA Ch1 254nm 4nm

Peak#	Ret. Time	Area	Height	Area %	Height %
1	4.092	370633	38846	95.863	95.004
2	4.614	15996	2043	4.137	4.996
Total		386629	40889	100.000	100.000

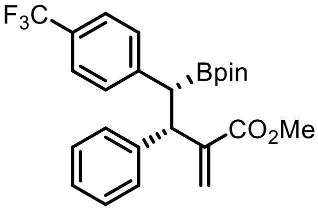

3d’

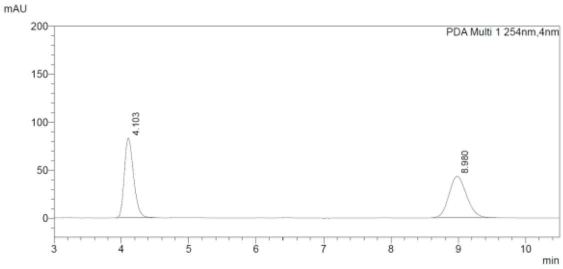

<Peak Table>

PDA Ch1 254nm

Peak#	Ret. Time	Area	Height	Conc.	Unit	Mark	Name
1	4.103	792776	83131	50.237			
2	8.980	785291	43127	49.763			
Total		1578067	126259				

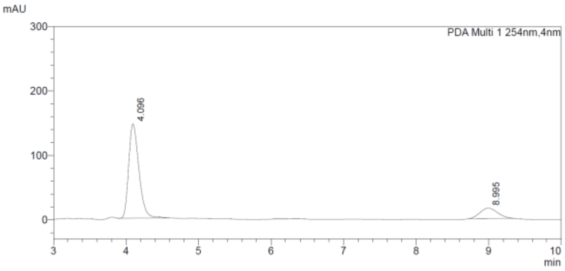

<Peak Table>

PDA Ch1 254nm

Peak#	Ret. Time	Area	Height	Conc.	Unit	Mark	Name
1	4.096	1425416	146327	83.718			
2	8.995	277222	16251	16.282			
Total		1702638	162578				

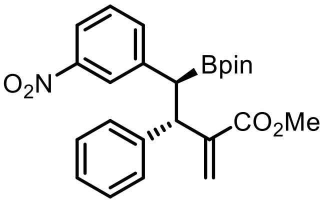

3e

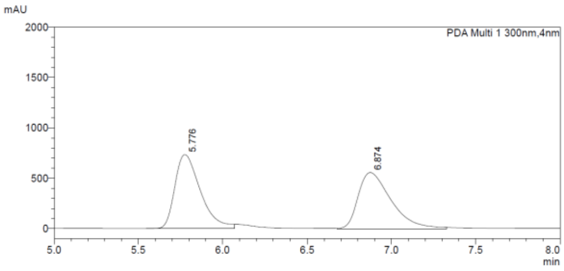

<Peak Table>

PDA Ch1 300nm

Peak#	Ret. Time	Area	Height	Conc.	Unit	Mark	Name
1	5.776	7774252	734150	50.070			
2	6.874	7752665	561409	49.930			
Total		15526918	1295559				

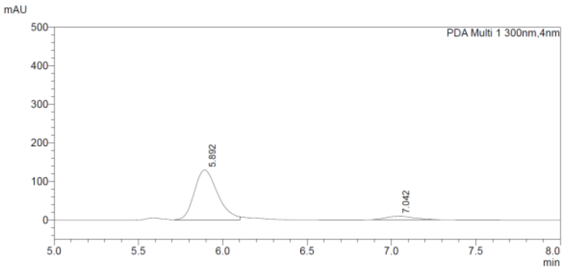

<Peak Table>

PDA Ch1 300nm

Peak#	Ret. Time	Area	Height	Conc.	Unit	Mark	Name
1	5.892	1246247	128754	92.249			
2	7.042	104713	9210	7.751			
Total		1350960	137964				

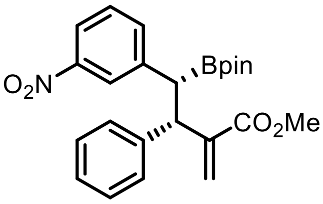

3e'

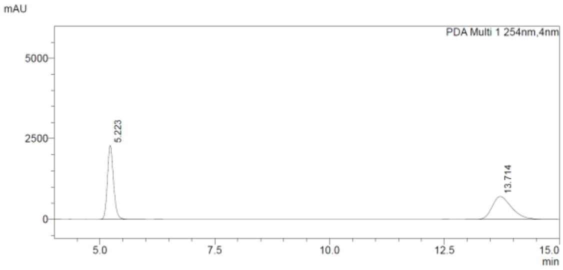

<Peak Table>

PDA Ch1 254nm

Peak#	Ret. Time	Area	Height	Conc.	Unit	Mark	Name
1	5.223	21009817	2284613	50.177			
2	13.714	20861366	712509	49.823			
Total		41871183	2997122				

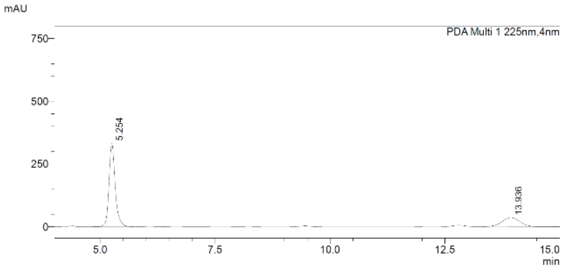

<Peak Table>

PDA Ch1 225nm

Peak#	Ret. Time	Area	Height	Conc.	Unit	Mark	Name
1	5.254	3152024	332815	74.889			
2	13.936	1056915	37273	25.111			
Total		4208939	370088				

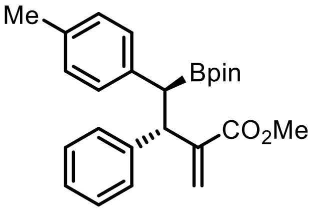

3f

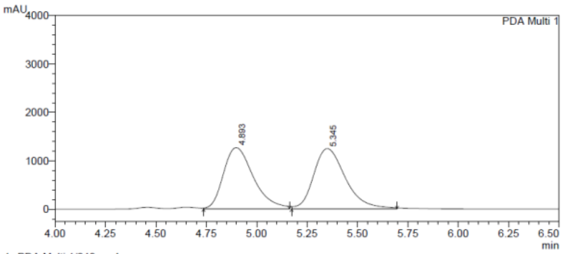

1 PDA Multi 1/240nm 4nm

PeakTable

PDA Ch1 240nm 4nm

Peak#	Ret. Time	Area	Height	Area %	Height %
1	4.893	13200765	1266834	49.016	50.445
2	5.345	13730685	1244474	50.984	49.555
Total		26931450	2511308	100.000	100.000

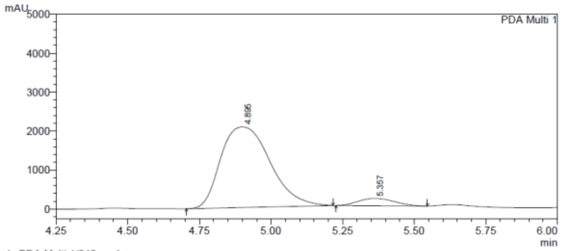

1 PDA Multi 1/240nm 4nm

PeakTable

PDA Ch1 240nm 4nm

Peak#	Ret. Time	Area	Height	Area %	Height %
1	4.895	24902406	2068318	93.880	91.590
2	5.357	1623510	189922	6.120	8.410
Total		26525916	2258240	100.000	100.000

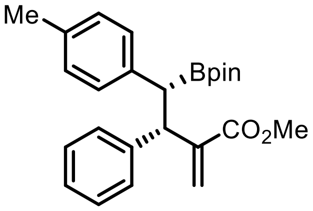

3f'

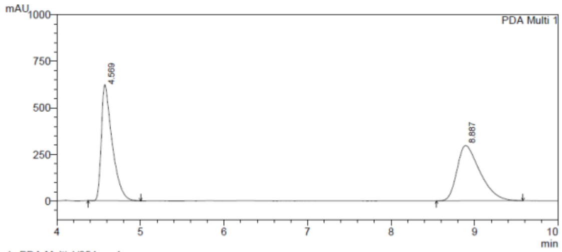

1 PDA Multi 1/254nm 4nm

PeakTable

PDA Ch1 254nm 4nm

Peak#	Ret. Time	Area	Height	Area %	Height %
1	4.569	5646919	623590	49.847	67.745
2	8.887	5681494	296901	50.153	32.255
Total		11328413	920492	100.000	100.000

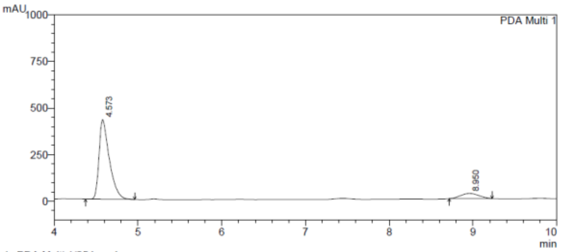

1 PDA Multi 1/254nm 4nm

PeakTable

PDA Ch1 254nm 4nm

Peak#	Ret. Time	Area	Height	Area %	Height %
1	4.573	3664530	428003	89.593	93.921
2	8.950	425673	27700	10.407	6.079
Total		4090202	455703	100.000	100.000

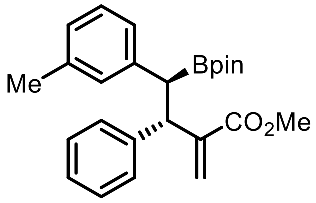

3g

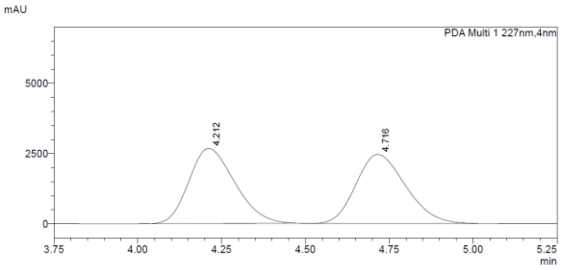

<Peak Table>

PDA Ch1 227nm

Peak#	Ret. Time	Area	Height	Conc.	Unit	Mark	Name
1	4.212	25363880	2672979	50.036			
2	4.716	25327169	2458231	49.964			
Total		50691049	5131210				

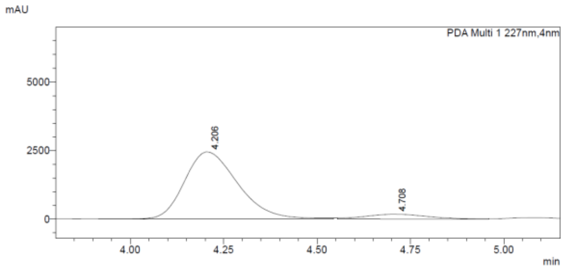

<Peak Table>

PDA Ch1 227nm

Peak#	Ret. Time	Area	Height	Conc.	Unit	Mark	Name
1	4.206	24441786	2452696	92.017			
2	4.708	2120450	180823	7.983			
Total		26562236	2633519				

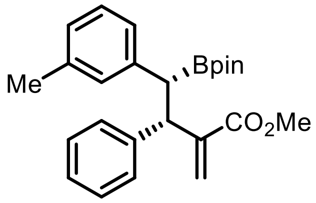

3g'

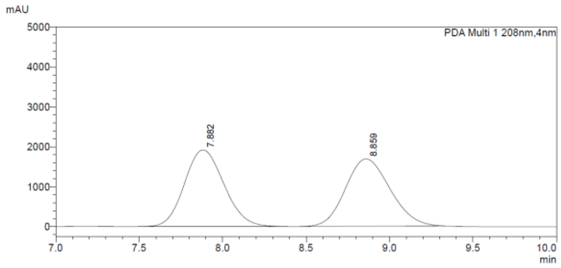

<Peak Table>

PDA Ch1 208nm

Peak#	Ret. Time	Area	Height	Conc.	Unit	Mark	Name
1	7.882	31585917	1919293	50.190			
2	8.859	31346973	1684288	49.810			
Total		62932891	3603581				

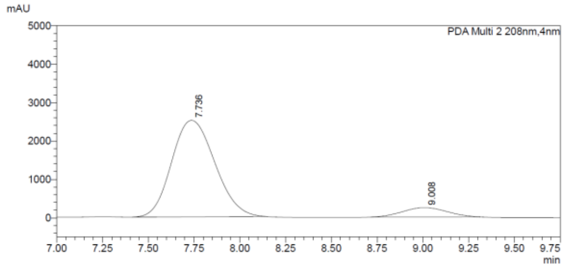

<Peak Table>

PDA Ch2 208nm

Peak#	Ret. Time	Area	Height	Conc.	Unit	Mark	Name
1	7.736	41274986	2512182	90.957			
2	9.008	4103412	244344	9.043			
Total		45378398	2756527				

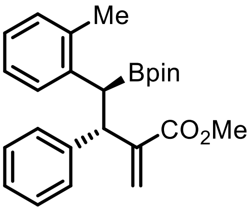

3h

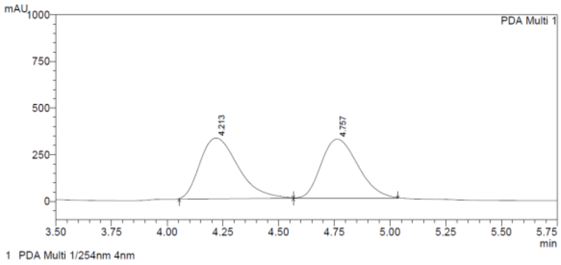

PeakTable

PDA Ch1 254nm 4nm

Peak#	Ret. Time	Area	Height	Area %	Height %
1	4.213	3902019	325439	51.635	50.676
2	4.757	3654941	316757	48.365	49.324
Total		7556960	642195	100.000	100.000

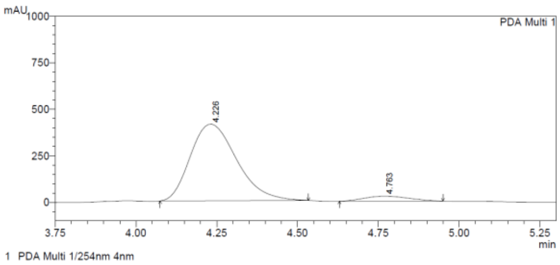

PeakTable

PDA Ch1 254nm 4nm

Peak#	Ret. Time	Area	Height	Area %	Height %
1	4.226	4122229	412223	94.245	93.963
2	4.763	251699	26484	5.755	6.037
Total		4373928	438707	100.000	100.000

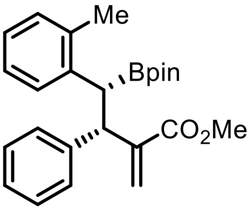

3h’

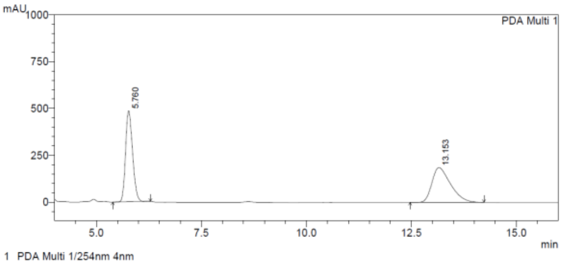

PeakTable

PDA Ch1 254nm 4nm

Peak#	Ret. Time	Area	Height	Area %	Height %
1	5.760	5673088	485744	49.731	72.322
2	13.153	5734383	185898	50.269	27.678
Total		11407471	671642	100.000	100.000

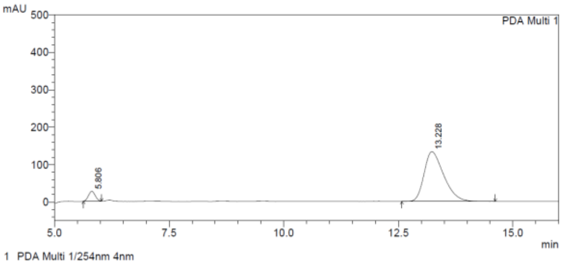

PeakTable

PDA Ch1 254nm 4nm

Peak#	Ret. Time	Area	Height	Area %	Height %
1	5.806	269653	26457	6.379	16.601
2	13.228	3957272	132909	93.621	83.399
Total		4226925	159366	100.000	100.000

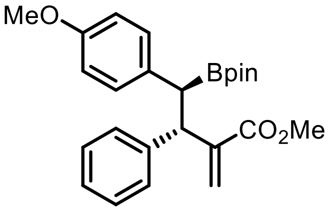

3i

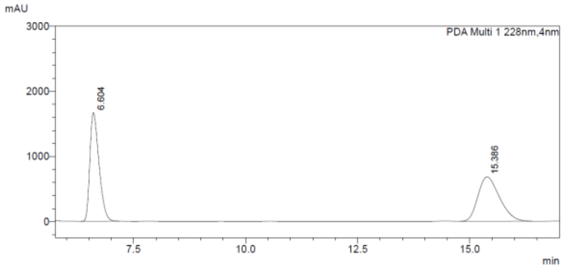

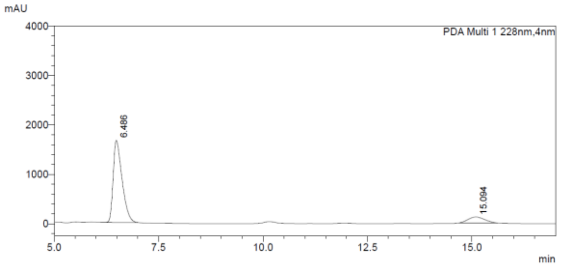

<Peak Table>
PDA Ch1 228nm

Peak#	Ret. Time	Area	Height	Conc.	Unit	Mark	Name
1	6.604	23673599	1670773	50.918			
2	15.386	22819714	682349	49.082			
Total		46493313	2353122				

<Peak Table>
PDA Ch1 228nm

Peak#	Ret. Time	Area	Height	Conc.	Unit	Mark	Name
1	6.486	24684393	1662469	88.132			
2	15.094	3324059	120381	11.868			
Total		28008452	1782850				

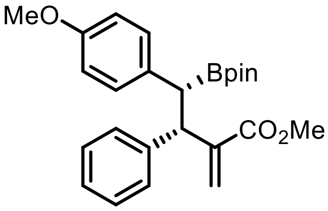

3i'

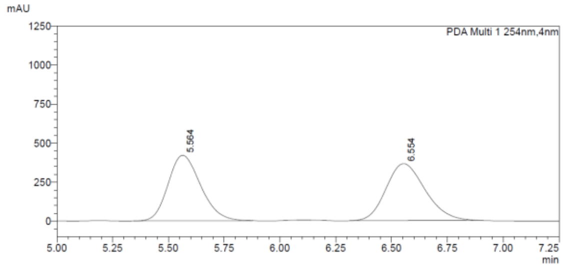

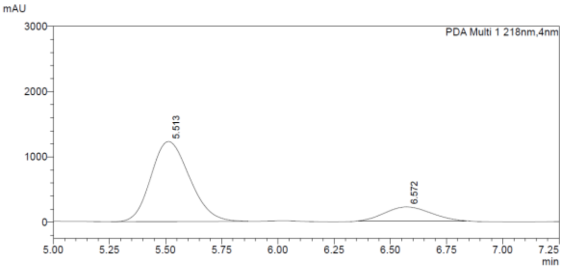

<Peak Table>
PDA Ch1 254nm

Peak#	Ret. Time	Area	Height	Conc.	Unit	Mark	Name
1	5.564	4270917	419955	48.872			
2	6.554	4467981	364775	51.128			
Total		8738899	784730				

<Peak Table>
PDA Ch1 218nm

Peak#	Ret. Time	Area	Height	Conc.	Unit	Mark	Name
1	5.513	14678018	1228172	83.040			
2	6.572	2997913	214316	16.960			
Total		17675931	1442488				

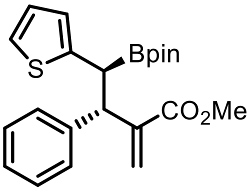

3j

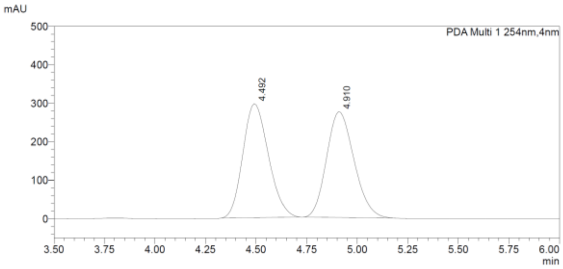

<Peak Table>

PDA Ch1 254nm

Peak#	Ret. Time	Area	Height	Conc.	Unit	Mark	Name
1	4.492	2601221	295921	50.066			
2	4.910	2594390	274490	49.934			
Total		5195611	570411				

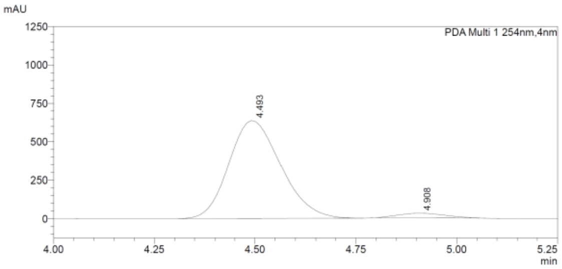

<Peak Table>

PDA Ch1 254nm

Peak#	Ret. Time	Area	Height	Conc.	Unit	Mark	Name
1	4.493	5686181	635647	95.943			
2	4.908	240434	30091	4.057			
Total		5926615	665738				

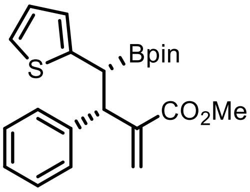

3j’

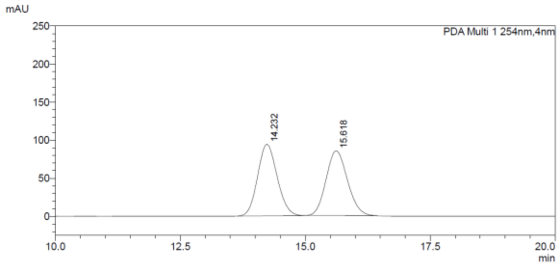

<Peak Table>

PDA Ch1 254nm

Peak#	Ret. Time	Area	Height	Conc.	Unit	Mark	Name
1	14.232	2554118	93896	50.220			
2	15.618	2531763	85010	49.780			
Total		5085882	178906				

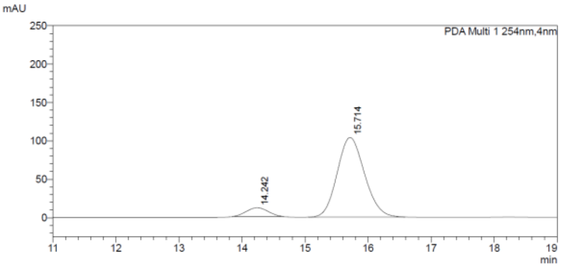

<Peak Table>

PDA Ch1 254nm

Peak#	Ret. Time	Area	Height	Conc.	Unit	Mark	Name
1	14.242	280569	11582	8.223			
2	15.714	3131642	103749	91.777			
Total		3412211	115331				

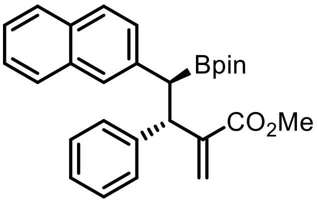

3k

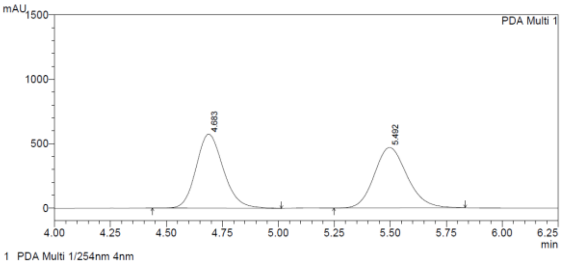

1 PDA Multi 1/254nm 4nm

PeakTable

PDA Ch1 254nm 4nm

Peak#	Ret. Time	Area	Height	Area %	Height %
1	4.683	4869544	573583	50.467	55.009
2	5.492	4779451	469121	49.533	44.991
Total		9648995	1042704	100.000	100.000

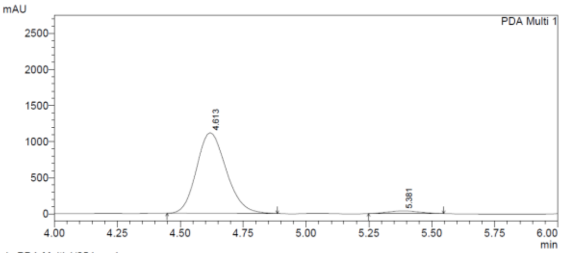

1 PDA Multi 1/254nm 4nm

PeakTable

PDA Ch1 254nm 4nm

Peak#	Ret. Time	Area	Height	Area %	Height %
1	4.613	9201956	1118247	96.627	96.809
2	5.381	321204	36858	3.373	3.191
Total		9523160	1155105	100.000	100.000

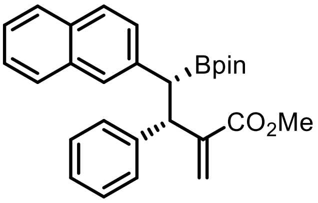

3k'

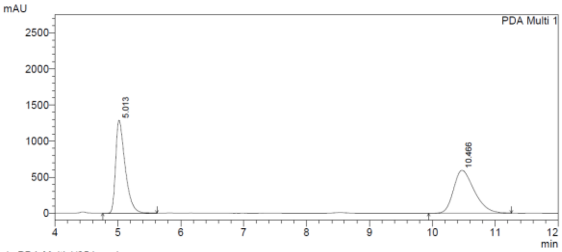

1 PDA Multi 1/254nm 4nm

PeakTable

PDA Ch1 254nm 4nm

Peak#	Ret. Time	Area	Height	Area %	Height %
1	5.013	13434819	1286571	49.603	68.370
2	10.466	13649626	595209	50.397	31.630
Total		27084445	1881780	100.000	100.000

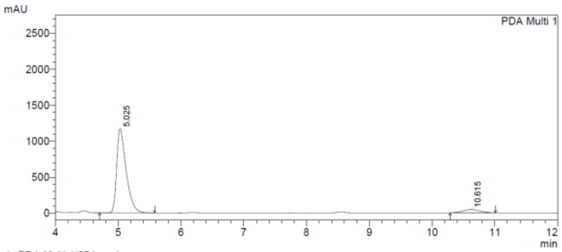

1 PDA Multi 1/254nm 4nm

PeakTable

PDA Ch1 254nm 4nm

Peak#	Ret. Time	Area	Height	Area %	Height %
1	5.025	12183902	1168651	92.830	96.253
2	10.615	941087	45493	7.170	3.747
Total		13124989	1214144	100.000	100.000

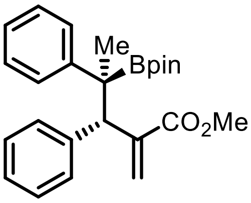

3l

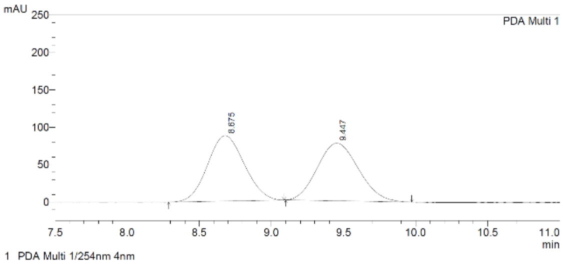

PeakTable

PDA Ch1 254nm 4nm

Peak#	Ret. Time	Area	Height	Area %	Height %
1	8.675	1555209	87583	50.278	53.194
2	9.447	1538014	77066	49.722	46.806
Total		3093224	164649	100.000	100.000

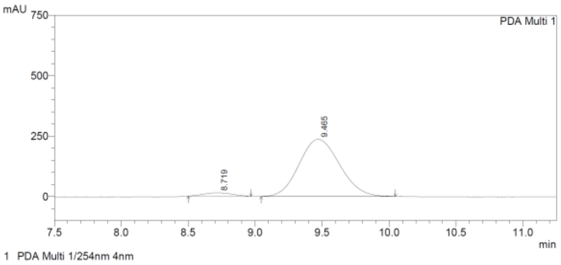

PeakTable

PDA Ch1 254nm 4nm

Peak#	Ret. Time	Area	Height	Area %	Height %
1	8.719	205238	13820	3.950	5.522
2	9.465	4990764	236450	96.050	94.478
Total		5196003	250270	100.000	100.000

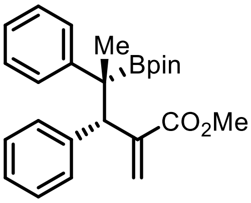

3l'

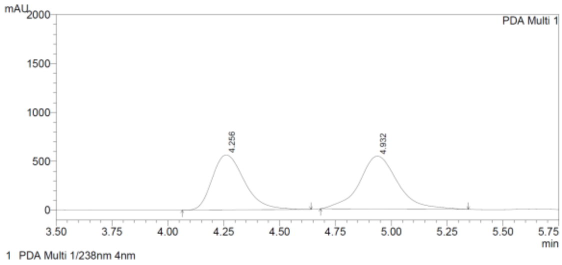

PeakTable

PDA Ch1 238nm 4nm

Peak#	Ret. Time	Area	Height	Area %	Height %
1	4.256	5629915	562125	46.704	50.966
2	4.932	6424656	540815	53.296	49.034
Total		12054571	1102941	100.000	100.000

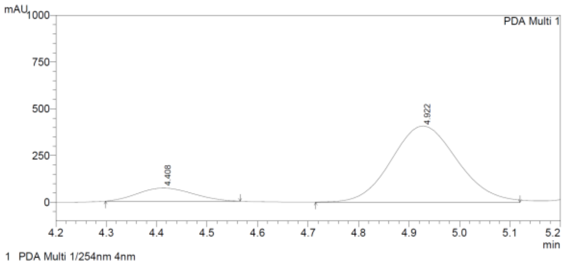

PeakTable

PDA Ch1 254nm 4nm

Peak#	Ret. Time	Area	Height	Area %	Height %
1	4.408	537296	70629	12.549	14.729
2	4.922	3744334	408907	87.451	85.271
Total		4281630	479536	100.000	100.000

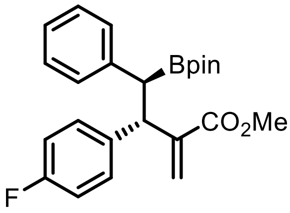

3m

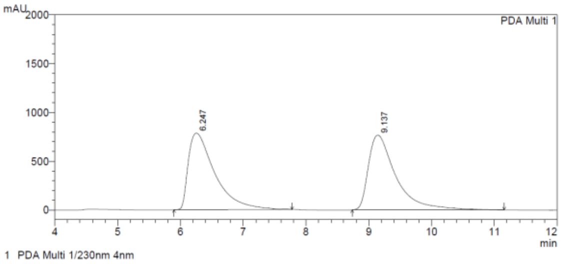

1 PDA Multi 1/230nm 4nm

PeakTable

PDA Ch1 230nm 4nm

Peak#	Ret. Time	Area	Height	Area %	Height %
1	6.247	23110569	784778	49.781	50.594
2	9.137	23313519	766337	50.219	49.406
Total		46424088	1551115	100.000	100.000

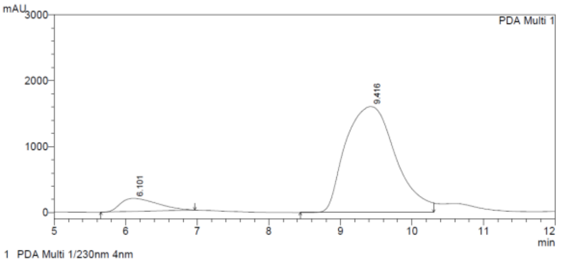

1 PDA Multi 1/230nm 4nm

PeakTable

PDA Ch1 230nm 4nm

Peak#	Ret. Time	Area	Height	Area %	Height %
1	6.101	7029357	197982	8.261	10.971
2	9.416	78057383	1606630	91.739	89.029
Total		85086740	1804612	100.000	100.000

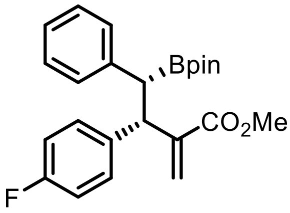

3m'

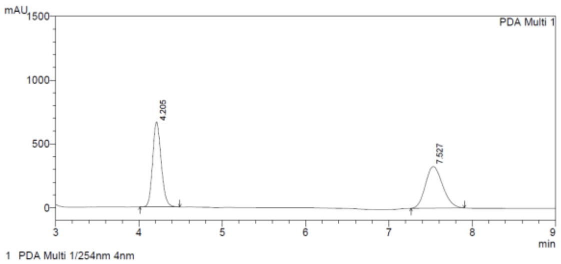

1 PDA Multi 1/254nm 4nm

PeakTable

PDA Ch1 254nm 4nm

Peak#	Ret. Time	Area	Height	Area %	Height %
1	4.205	4745096	666447	50.309	67.138
2	7.527	4686778	326200	49.691	32.862
Total		9431874	992648	100.000	100.000

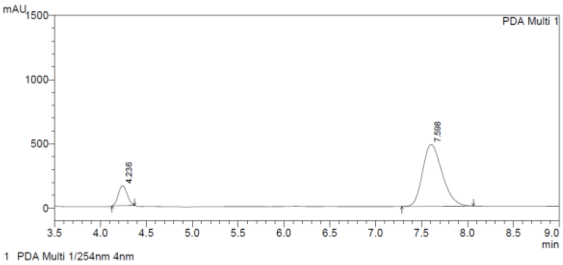

1 PDA Multi 1/254nm 4nm

PeakTable

PDA Ch1 254nm 4nm

Peak#	Ret. Time	Area	Height	Area %	Height %
1	4.236	1043225	156191	12.764	24.499
2	7.598	7130060	481349	87.236	75.501
Total		8173285	637540	100.000	100.000

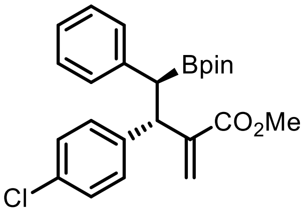

3n

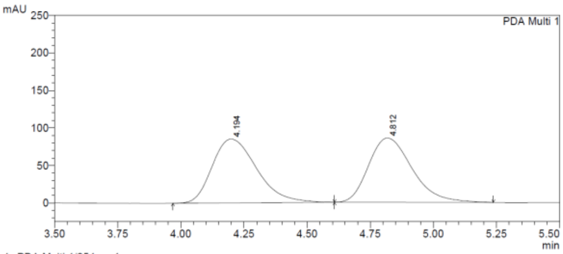

1 PDA Multi 1/254nm 4nm

PeakTable

PDA Ch1 254nm 4nm

Peak#	Ret. Time	Area	Height	Area %	Height %
1	4.194	1042873	85139	50.376	49.881
2	4.812	1027314	85545	49.624	50.119
Total		2070186	170684	100.000	100.000

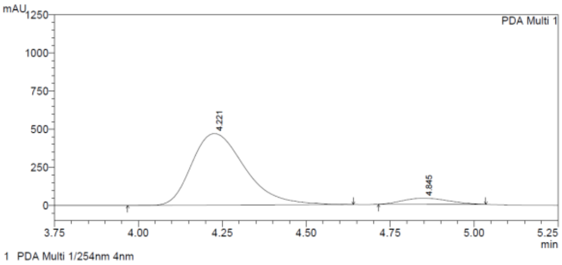

1 PDA Multi 1/254nm 4nm

PeakTable

PDA Ch1 254nm 4nm

Peak#	Ret. Time	Area	Height	Area %	Height %
1	4.221	5170654	469287	93.113	92.102
2	4.845	382413	40241	6.887	7.898
Total		5553067	509528	100.000	100.000

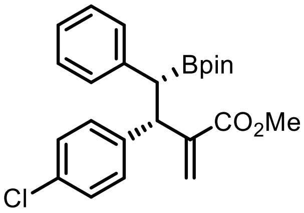

3n’

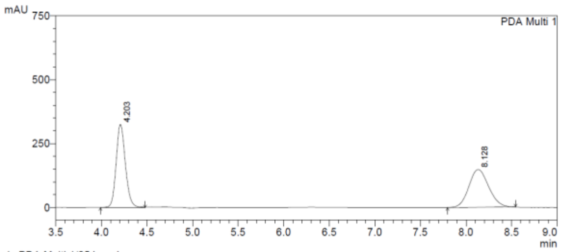

1 PDA Multi 1/254nm 4nm

PeakTable

PDA Ch1 254nm 4nm

Peak#	Ret. Time	Area	Height	Area %	Height %
1	4.203	2318072	325928	50.979	68.778
2	8.128	2229038	147953	49.021	31.222
Total		4547109	473881	100.000	100.000

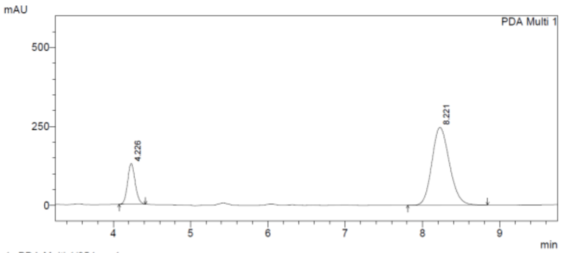

1 PDA Multi 1/254nm 4nm

PeakTable

PDA Ch1 254nm 4nm

Peak#	Ret. Time	Area	Height	Area %	Height %
1	4.226	879511	128851	18.454	34.375
2	8.221	3886386	245986	81.546	65.625
Total		4765896	374836	100.000	100.000

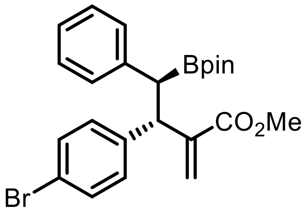

3o

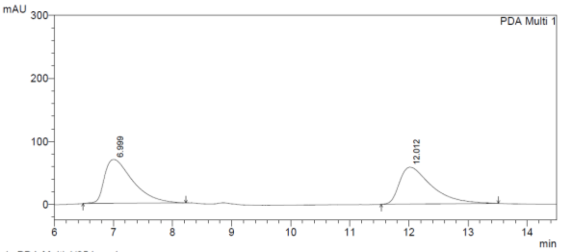

1 PDA Multi 1/254nm 4nm

PeakTable

PDA Ch1 254nm 4nm

Peak#	Ret. Time	Area	Height	Area %	Height %
1	6.999	2334383	69643	51.218	54.307
2	12.012	2223365	58596	48.782	45.693
Total		4557748	128238	100.000	100.000

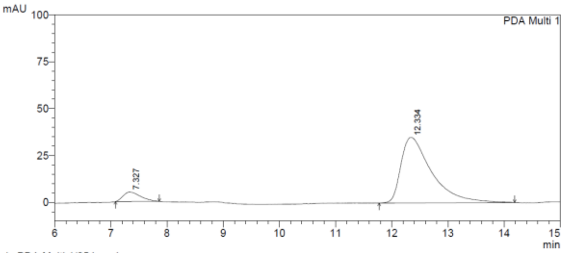

1 PDA Multi 1/254nm 4nm

PeakTable

PDA Ch1 254nm 4nm

Peak#	Ret. Time	Area	Height	Area %	Height %
1	7.327	107082	5017	7.442	12.538
2	12.334	1331783	34996	92.558	87.462
Total		1438865	40013	100.000	100.000

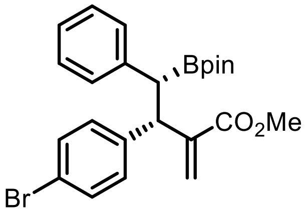

3o’

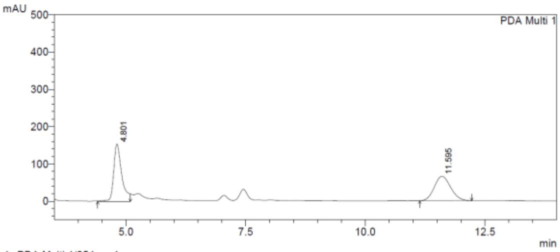

1 PDA Multi 1/254nm 4nm

PeakTable

PDA Ch1 254nm 4nm

Peak#	Ret. Time	Area	Height	Area %	Height %
1	4.801	1835001	154407	54.060	70.195
2	11.595	1559363	65560	45.940	29.805
Total		3394364	219967	100.000	100.000

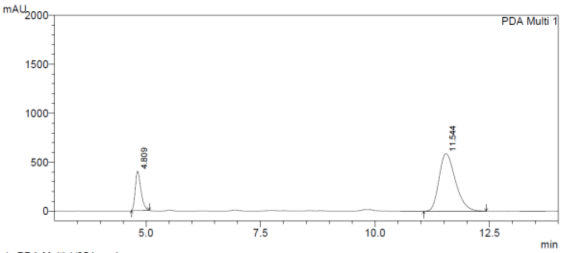

1 PDA Multi 1/254nm 4nm

PeakTable

PDA Ch1 254nm 4nm

Peak#	Ret. Time	Area	Height	Area %	Height %
1	4.809	3449110	401375	19.072	40.589
2	11.544	14635406	587512	80.928	59.411
Total		18084515	988887	100.000	100.000

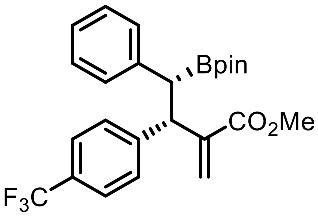

3p

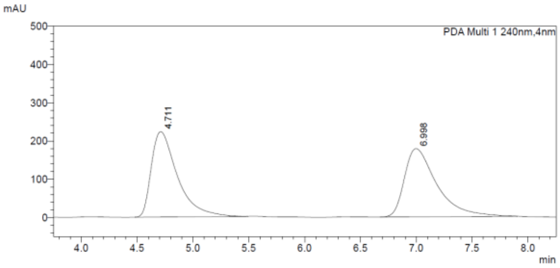

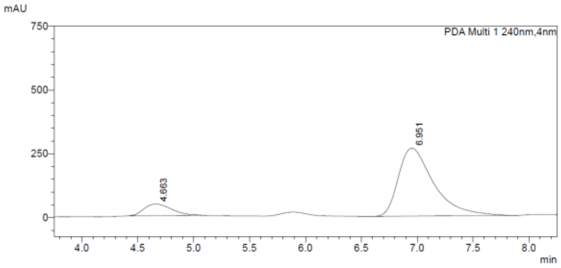

<Peak Table>
PDA Ch1 240nm

Peak#	Ret. Time	Area	Height	Conc.	Unit	Mark	Name
1	4.711	3624986	222977	50.193			
2	6.998	3597083	178256	49.807			
Total		7222070	401233				

<Peak Table>
PDA Ch1 240nm

Peak#	Ret. Time	Area	Height	Conc.	Unit	Mark	Name
1	4.663	777110	45213	11.707			
2	6.951	5860887	266322	88.293			
Total		6637997	311535				

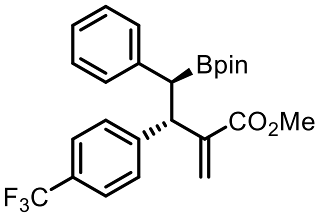

3p'

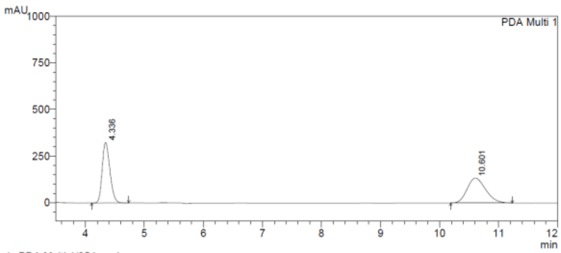

1 PDA Multi 1/254nm 4nm

PeakTable

PDA Ch1 254nm 4nm

Peak#	Ret. Time	Area	Height	Area %	Height %
1	4.336	2964872	324902	50.342	70.849
2	10.601	2924630	133681	49.658	29.151
Total		5889502	458584	100.000	100.000

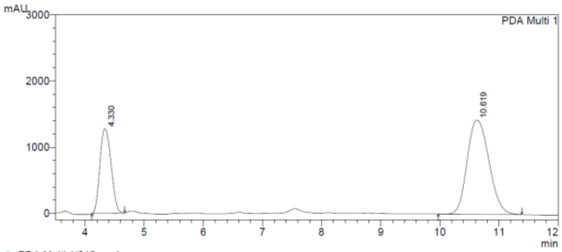

1 PDA Multi 1/218nm 4nm

PeakTable

PDA Ch1 218nm 4nm

Peak#	Ret. Time	Area	Height	Area %	Height %
1	4.330	16702133	1276957	31.417	47.340
2	10.619	36461128	1420481	68.583	52.660
Total		53163261	2697438	100.000	100.000

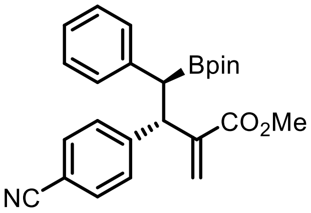

3q

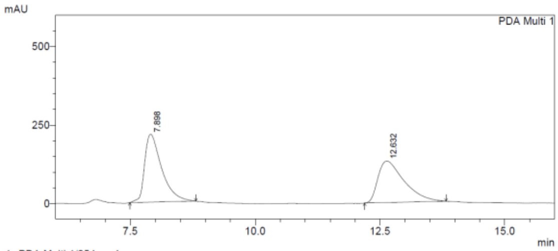

1 PDA Multi 1/254nm 4nm

PeakTable

PDA Ch1 254nm 4nm

Peak#	Ret. Time	Area	Height	Area %	Height %
1	7.898	5158669	216489	51.593	62.039
2	12.632	4840060	132465	48.407	37.961
Total		9998730	348955	100.000	100.000

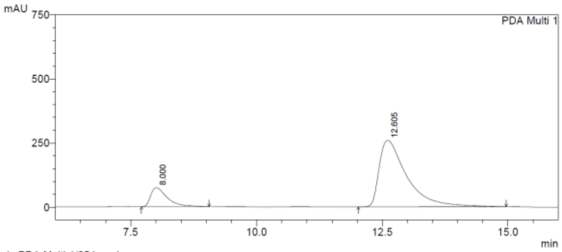

1 PDA Multi 1/254nm 4nm

PeakTable

PDA Ch1 254nm 4nm

Peak#	Ret. Time	Area	Height	Area %	Height %
1	8.000	1689049	74062	14.469	22.191
2	12.605	9984856	259689	85.531	77.809
Total		11673906	333751	100.000	100.000

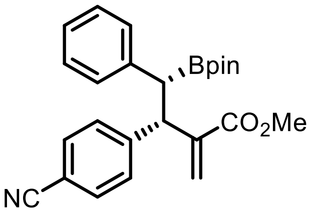

3q'

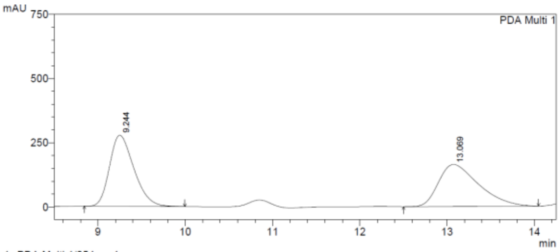

1 PDA Multi 1/254nm 4nm

PeakTable

PDA Ch1 254nm 4nm

Peak#	Ret. Time	Area	Height	Area %	Height %
1	9.244	5374838	276711	50.671	62.850
2	13.069	5232481	163560	49.329	37.150
Total		10607320	440270	100.000	100.000

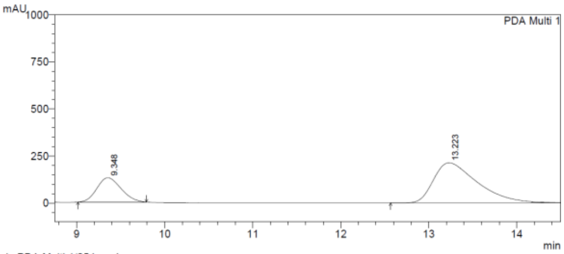

1 PDA Multi 1/254nm 4nm

PeakTable

PDA Ch1 254nm 4nm

Peak#	Ret. Time	Area	Height	Area %	Height %
1	9.348	2460463	128872	24.936	37.692
2	13.223	7406817	213033	75.064	62.308
Total		9867281	341905	100.000	100.000

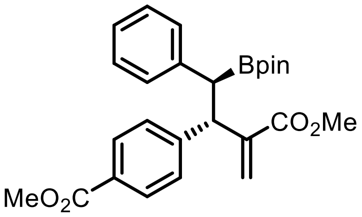

3r

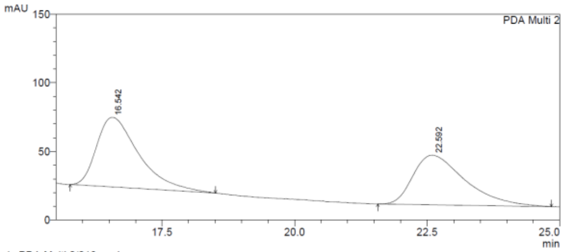

1 PDA Multi 2/218nm 4nm

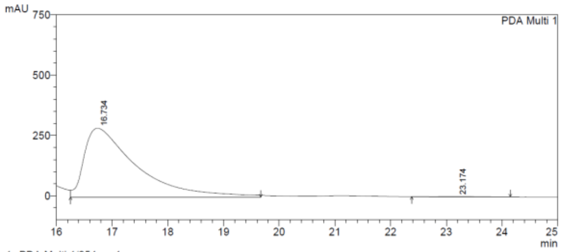

1 PDA Multi 1/254nm 4nm

PeakTable

PDA Ch2 218nm 4nm

Peak#	Ret. Time	Area	Height	Area %	Height %
1	16.542	2811242	50719	53.146	58.384
2	22.592	2478381	36153	46.854	41.616
Total		5289623	86872	100.000	100.000

PeakTable

PDA Ch1 254nm 4nm

Peak#	Ret. Time	Area	Height	Area %	Height %
1	16.734	18573701	285505	99.752	99.639
2	23.174	46201	1034	0.248	0.361
Total		18619903	286539	100.000	100.000

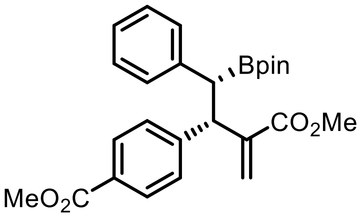

3r'

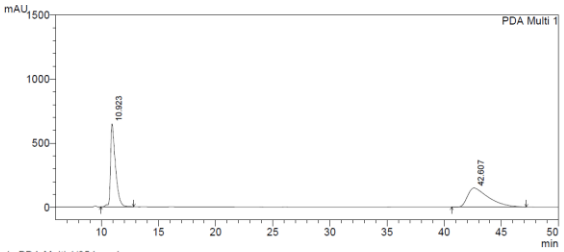

1 PDA Multi 1/254nm 4nm

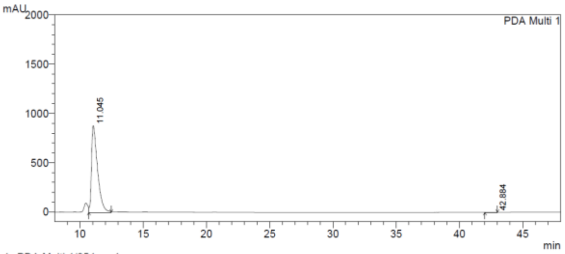

1 PDA Multi 1/254nm 4nm

PeakTable

PDA Ch1 254nm 4nm

Peak#	Ret. Time	Area	Height	Area %	Height %
1	10.923	19384836	647351	50.641	81.405
2	42.607	18893762	147876	49.359	18.595
Total		38278598	795226	100.000	100.000

PeakTable

PDA Ch1 254nm 4nm

Peak#	Ret. Time	Area	Height	Area %	Height %
1	11.045	29109552	880826	100.006	99.996
2	42.884	-1779	35	-0.006	0.004
Total		29107773	880861	100.000	100.000

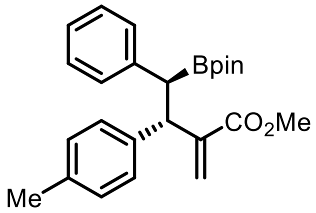

3s

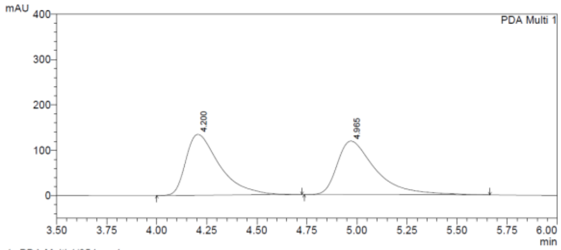

1 PDA Multi 1/254nm 4nm

PeakTable

PDA Ch1 254nm 4nm

Peak#	Ret. Time	Area	Height	Area %	Height %
1	4.200	1572771	134265	49.911	53.195
2	4.965	1578394	118134	50.089	46.805
Total		3151166	252399	100.000	100.000

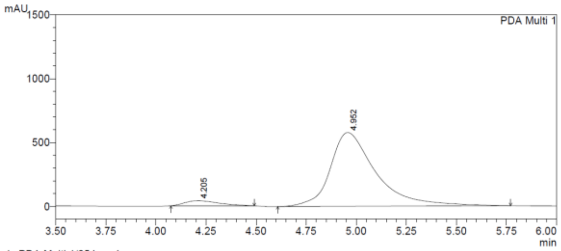

1 PDA Multi 1/254nm 4nm

PeakTable

PDA Ch1 254nm 4nm

Peak#	Ret. Time	Area	Height	Area %	Height %
1	4.205	473609	39639	4.930	6.419
2	4.952	9132740	577838	95.070	93.581
Total		9606348	617477	100.000	100.000

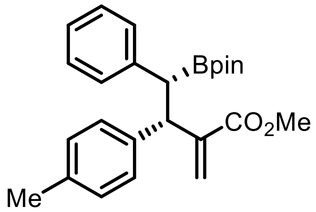

3s'

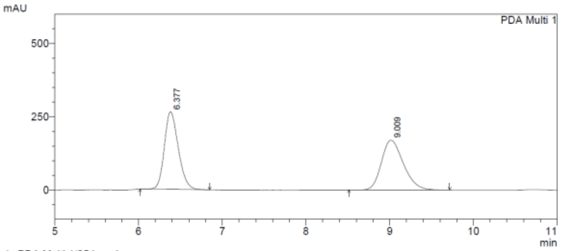

1 PDA Multi 1/254nm 4nm

PeakTable

PDA Ch1 254nm 4nm

Peak#	Ret. Time	Area	Height	Area %	Height %
1	6.377	3075602	265098	49.885	60.914
2	9.009	3089737	170105	50.115	39.086
Total		6165339	435203	100.000	100.000

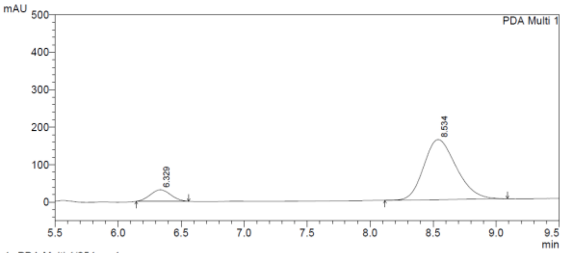

1 PDA Multi 1/254nm 4nm

PeakTable

PDA Ch1 254nm 4nm

Peak#	Ret. Time	Area	Height	Area %	Height %
1	6.329	349751	30950	10.833	16.184
2	8.534	2878774	160283	89.167	83.816
Total		3228525	191233	100.000	100.000

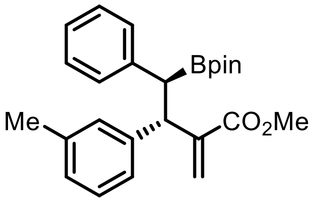

3t

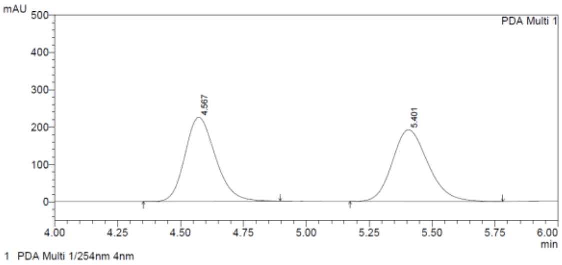

PeakTable

PDA Ch1 254nm 4nm

Peak#	Ret. Time	Area	Height	Area %	Height %
1	4.567	1894443	224915	50.016	53.955
2	5.401	1893196	191943	49.984	46.045
Total		3787639	416857	100.000	100.000

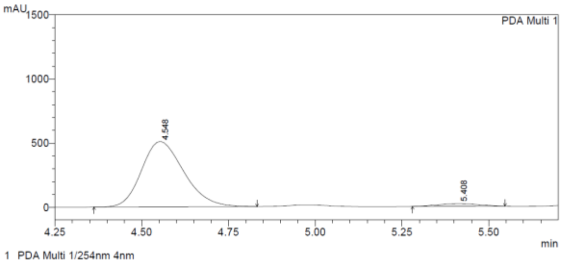

PeakTable

PDA Ch1 254nm 4nm

Peak#	Ret. Time	Area	Height	Area %	Height %
1	4.548	4322652	510024	96.136	96.070
2	5.408	173762	20862	3.864	3.930
Total		4496415	530886	100.000	100.000

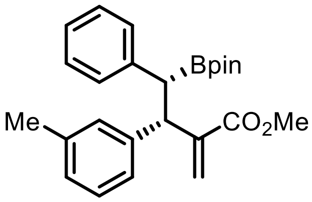

3t'

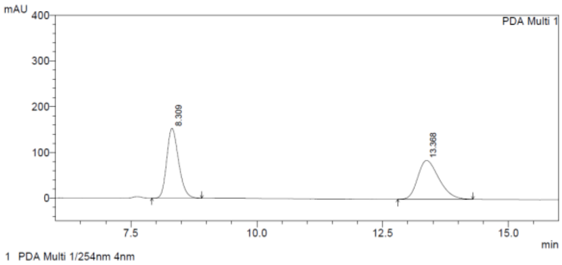

PeakTable

PDA Ch1 254nm 4nm

Peak#	Ret. Time	Area	Height	Area %	Height %
1	8.309	2502296	152559	50.219	64.279
2	13.368	2480504	84782	49.781	35.721
Total		4982801	237341	100.000	100.000

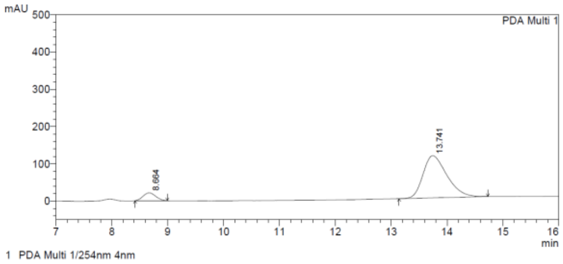

PeakTable

PDA Ch1 254nm 4nm

Peak#	Ret. Time	Area	Height	Area %	Height %
1	8.664	330280	21071	8.932	15.702
2	13.741	3367305	113122	91.068	84.298
Total		3697586	134193	100.000	100.000

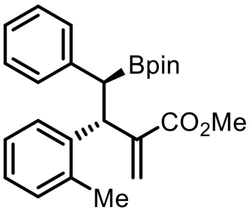

3u

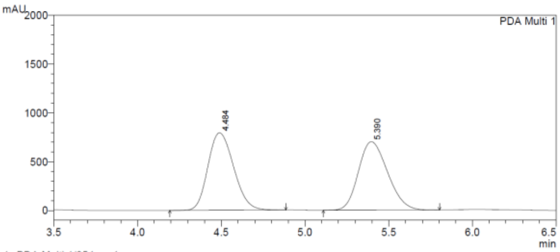

1 PDA Multi 1/254nm 4nm

PeakTable

PDA Ch1 254nm 4nm

Peak#	Ret. Time	Area	Height	Area %	Height %
1	4.484	8496363	793096	49.980	53.056
2	5.390	8503284	701743	50.020	46.944
Total		16999647	1494840	100.000	100.000

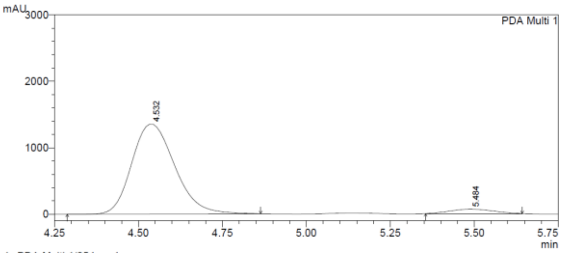

1 PDA Multi 1/254nm 4nm

PeakTable

PDA Ch1 254nm 4nm

Peak#	Ret. Time	Area	Height	Area %	Height %
1	4.532	12025123	1353140	95.374	95.333
2	5.484	583297	66240	4.626	4.667
Total		12608420	1419380	100.000	100.000

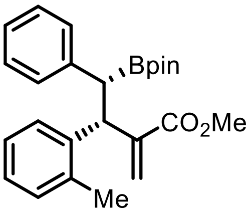

3u'

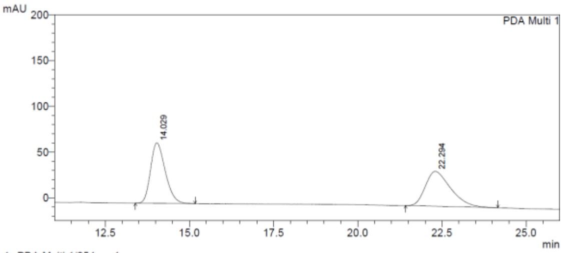

1 PDA Multi 1/254nm 4nm

PeakTable

PDA Ch1 254nm 4nm

Peak#	Ret. Time	Area	Height	Area %	Height %
1	14.029	1998413	66003	50.082	63.413
2	22.294	1991888	38081	49.918	36.587
Total		3990301	104084	100.000	100.000

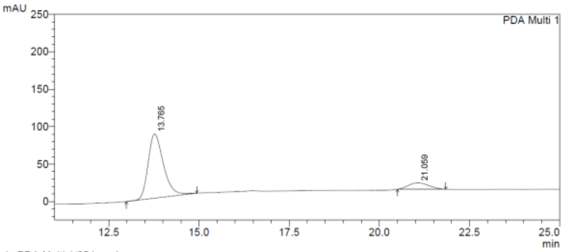

1 PDA Multi 1/254nm 4nm

PeakTable

PDA Ch1 254nm 4nm

Peak#	Ret. Time	Area	Height	Area %	Height %
1	13.765	2535828	85844	88.221	90.880
2	21.059	338562	8615	11.779	9.120
Total		2874390	94459	100.000	100.000

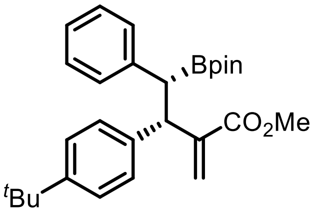

3v

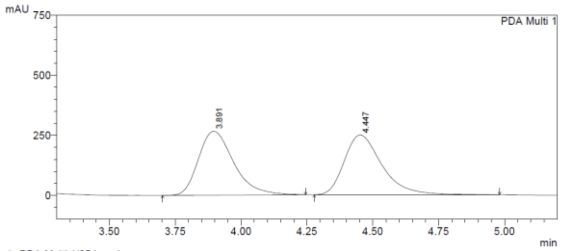

1 PDA Multi 1/254nm 4nm

PeakTable

PDA Ch1 254nm 4nm

Peak#	Ret. Time	Area	Height	Area %	Height %
1	3.891	2482016	266833	50.055	51.803
2	4.447	2476526	248261	49.945	48.197
Total		4958542	515094	100.000	100.000

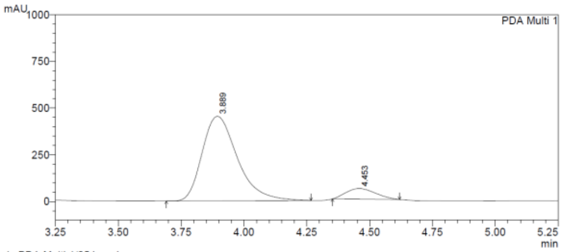

1 PDA Multi 1/254nm 4nm

PeakTable

PDA Ch1 254nm 4nm

Peak#	Ret. Time	Area	Height	Area %	Height %
1	3.889	4418400	453994	90.541	88.906
2	4.453	461590	56653	9.459	11.094
Total		4879991	510648	100.000	100.000

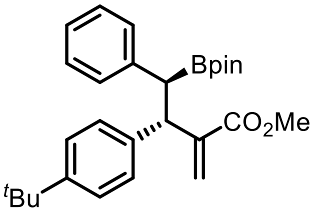

3v’

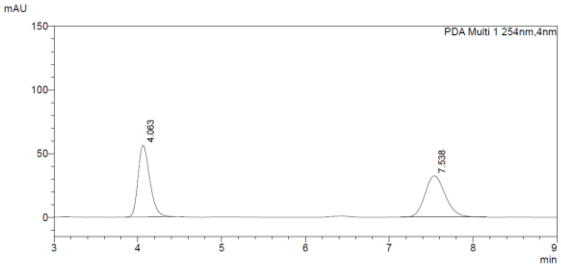

<Peak Table>

PDA Ch1 254nm

Peak#	Ret. Time	Area	Height	Conc.	Unit	Mark	Name
1	4.063	547767	56238	49.947			
2	7.538	548934	32473	50.053			
Total		1096702	88712				

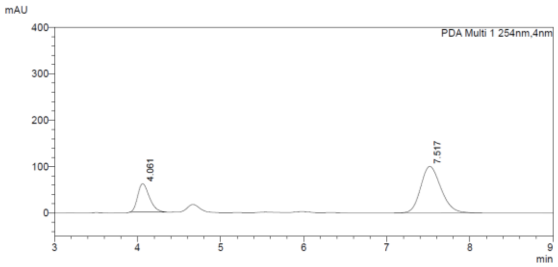

<Peak Table>

PDA Ch1 254nm

Peak#	Ret. Time	Area	Height	Conc.	Unit	Mark	Name
1	4.061	603909	61522	26.288			
2	7.517	1693391	100103	73.712			
Total		2297300	161625				

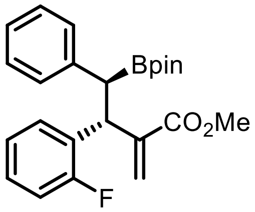

3w

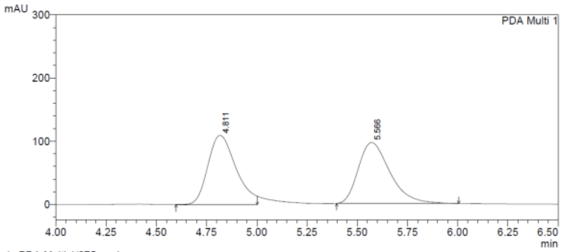

1 PDA Multi 1/275nm 4nm

PeakTable

PDA Ch1 275nm 4nm

Peak#	Ret. Time	Area	Height	Area %	Height %
1	4.811	1070917	109554	50.266	53.191
2	5.566	1059598	96410	49.734	46.809
Total		2130515	205964	100.000	100.000

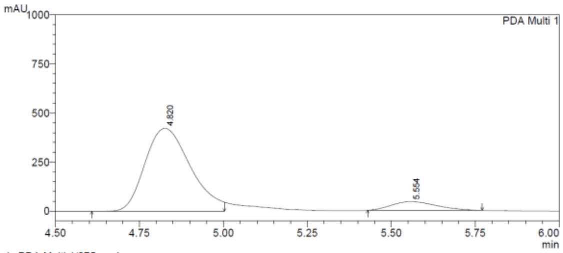

1 PDA Multi 1/275nm 4nm

PeakTable

PDA Ch1 275nm 4nm

Peak#	Ret. Time	Area	Height	Area %	Height %
1	4.820	3996453	422638	90.711	90.556
2	5.554	409245	44077	9.289	9.444
Total		4405698	466715	100.000	100.000

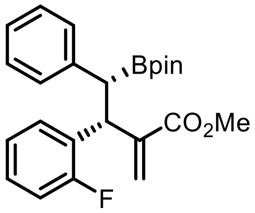

3w'

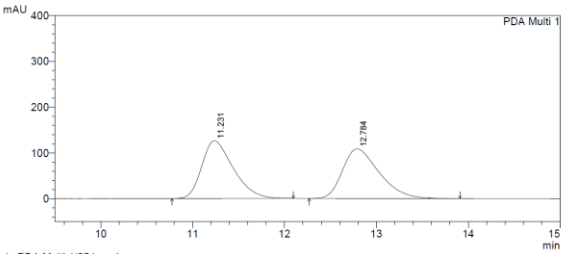

1 PDA Multi 1/254nm 4nm

PeakTable

PDA Ch1 254nm 4nm

Peak#	Ret. Time	Area	Height	Area %	Height %
1	11.231	2994247	126002	49.916	53.829
2	12.784	3004311	108074	50.084	46.171
Total		5998558	234076	100.000	100.000

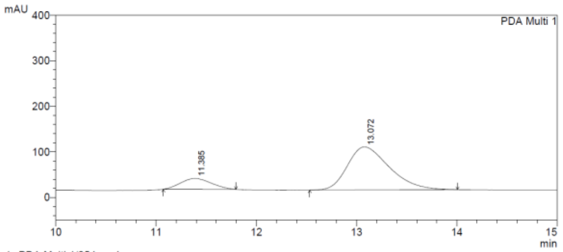

1 PDA Multi 1/254nm 4nm

PeakTable

PDA Ch1 254nm 4nm

Peak#	Ret. Time	Area	Height	Area %	Height %
1	11.385	502009	23590	15.428	19.989
2	13.072	2751776	94425	84.572	80.011
Total		3253785	118015	100.000	100.000

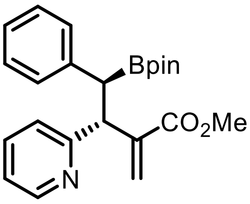

3x

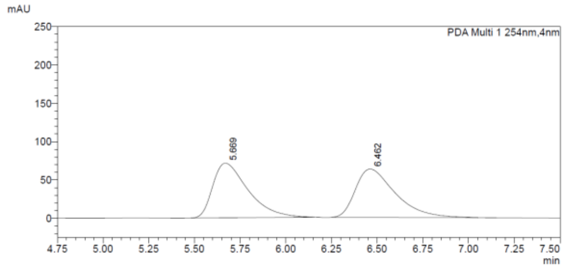

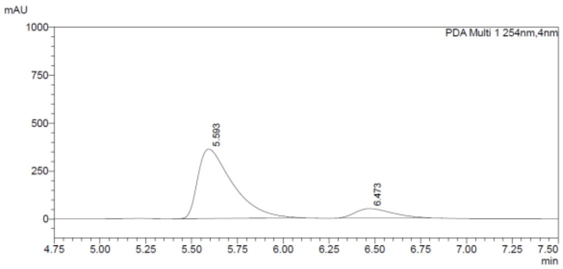

<Peak Table>

PDA Ch1 254nm

Peak#	Ret. Time	Area	Height	Conc.	Unit	Mark	Name
1	5.669	959055	71210	50.116			
2	6.462	954621	63079	49.884			
Total		1913676	134289				

<Peak Table>

PDA Ch1 254nm

Peak#	Ret. Time	Area	Height	Conc.	Unit	Mark	Name
1	5.593	4800511	362277	87.866			
2	6.473	662919	48259	12.134			
Total		5463430	410536				

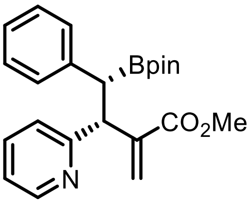

3x'

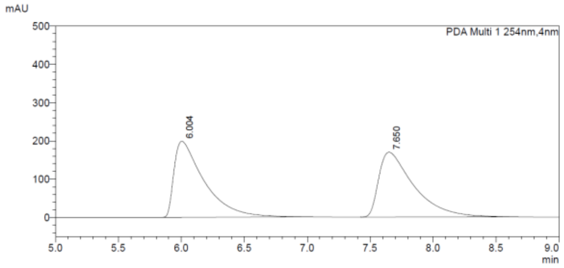

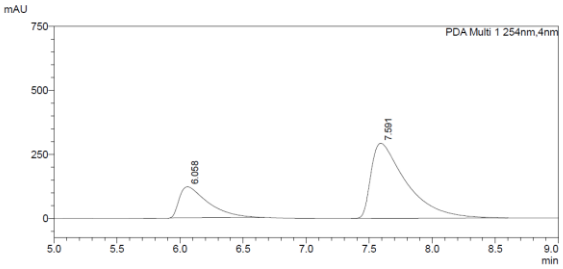

<Peak Table>

PDA Ch1 254nm

Peak#	Ret. Time	Area	Height	Conc.	Unit	Mark	Name
1	6.004	3325465	198793	50.130			
2	7.650	3308193	169515	49.870			
Total		6633658	368309				

<Peak Table>

PDA Ch1 254nm

Peak#	Ret. Time	Area	Height	Conc.	Unit	Mark	Name
1	6.058	1929774	121399	25.265			
2	7.591	5708354	293177	74.735			
Total		7638127	414575				

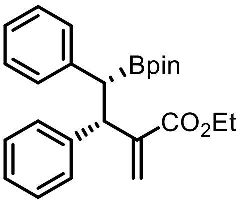

3y

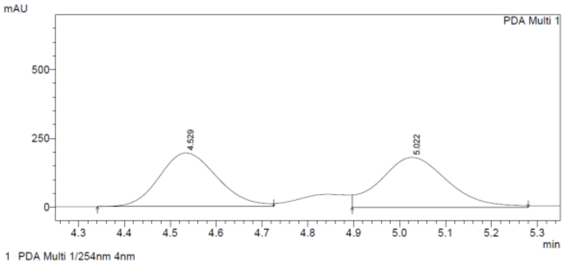

1 PDA Multi 1/254nm 4nm

PeakTable

PDA Ch1 254nm 4nm

Peak#	Ret. Time	Area	Height	Area %	Height %
1	4.529	1723397	195781	48.484	51.905
2	5.022	1831179	181408	51.516	48.095
Total		3554576	377189	100.000	100.000

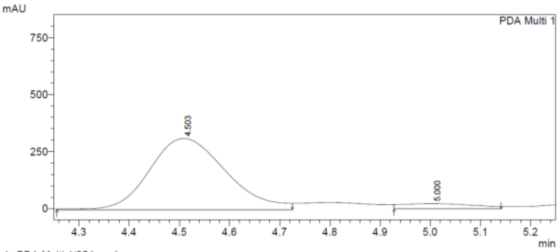

1 PDA Multi 1/254nm 4nm

PeakTable

PDA Ch1 254nm 4nm

Peak#	Ret. Time	Area	Height	Area %	Height %
1	4.503	3319202	312992	93.090	92.893
2	5.000	246385	23946	6.910	7.107
Total		3565587	336938	100.000	100.000

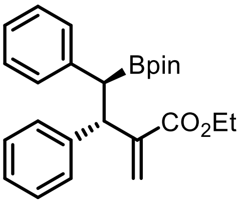

3y'

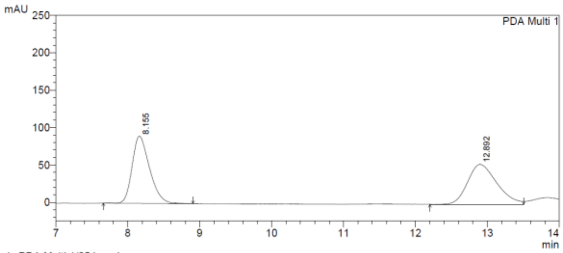

1 PDA Multi 1/254nm 4nm

PeakTable

PDA Ch1 254nm 4nm

Peak#	Ret. Time	Area	Height	Area %	Height %
1	8.155	1509535	89865	49.750	62.674
2	12.892	1524726	53519	50.250	37.326
Total		3034261	143383	100.000	100.000

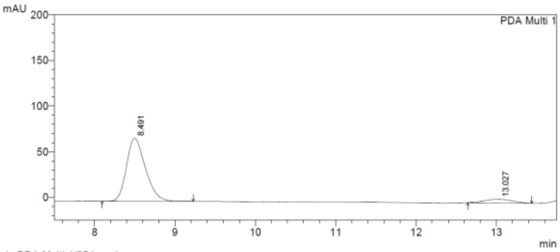

1 PDA Multi 1/254nm 4nm

PeakTable

PDA Ch1 254nm 4nm

Peak#	Ret. Time	Area	Height	Area %	Height %
1	8.491	1126513	68826	92.117	94.343
2	13.027	96400	4127	7.883	5.657
Total		1222913	72953	100.000	100.000

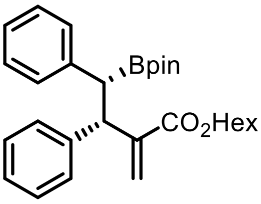

3z

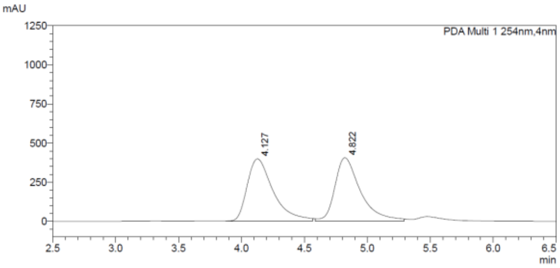

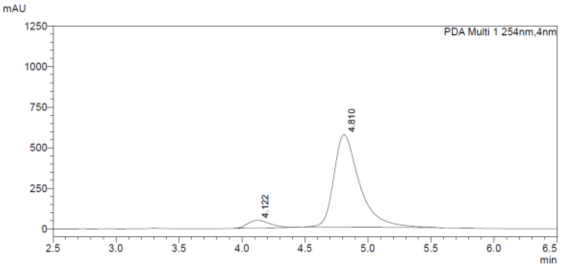

<Peak Table>

PDA Ch1 254nm

Peak#	Ret. Time	Area	Height	Conc.	Unit	Mark	Name
1	4.127	5750861	398792	49.887			
2	4.822	5776876	406045	50.113			
Total		11527737	804838				

<Peak Table>

PDA Ch1 254nm

Peak#	Ret. Time	Area	Height	Conc.	Unit	Mark	Name
1	4.122	585088	47131	6.712			
2	4.810	8131603	570636	93.288			
Total		8716691	617767				

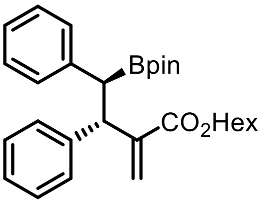

3z'

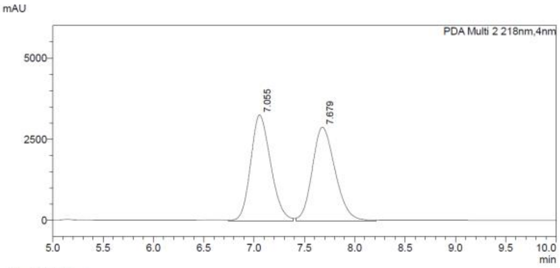

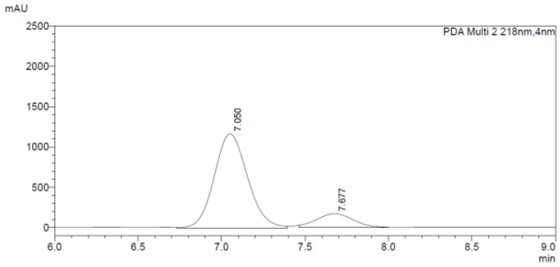

<Peak Table>

PDA Ch2 218nm

Peak#	Ret. Time	Area	Height	Conc.	Unit	Mark	Name
1	7.055	45057472	3257912	49.602			
2	7.679	45781373	2889730	50.398			
Total		90838844	6147642				

<Peak Table>

PDA Ch2 218nm

Peak#	Ret. Time	Area	Height	Conc.	Unit	Mark	Name
1	7.050	16519842	1175555	86.106			
2	7.677	2665611	172577	13.894			
Total		19185452	1348132				

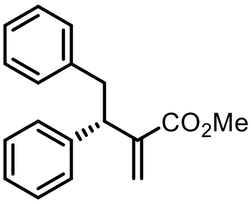

10

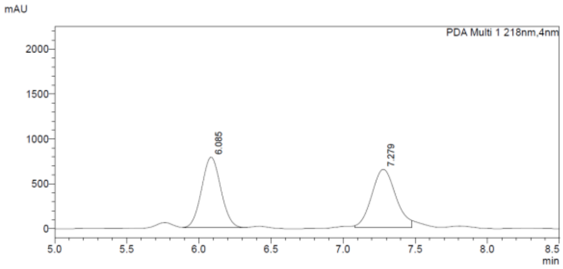

<Peak Table>

PDA Ch1 218nm

Peak#	Ret. Time	Area	Height	Conc.	Unit	Mark	Name
1	6.085	7215501	780646	48.707			
2	7.279	7598688	651181	51.293			
Total		14814189	1431827				

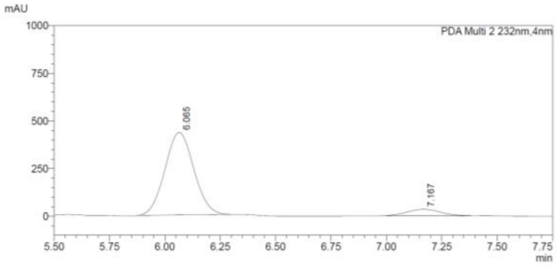

<Peak Table>

PDA Ch2 232nm

Peak#	Ret. Time	Area	Height	Conc.	Unit	Mark	Name
1	6.065	3956683	433402	92.089			
2	7.167	339900	33312	7.911			
Total		4296583	466714				

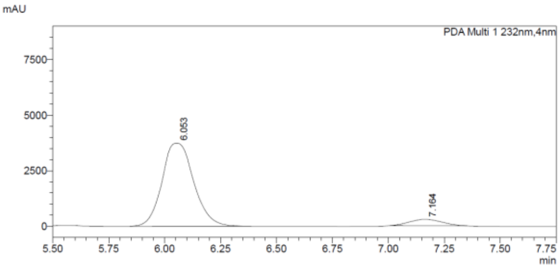

<Peak Table>

PDA Ch1 232nm

Peak#	Ret. Time	Area	Height	Conc.	Unit	Mark	Name
1	6.053	36679801	3736851	92.661			
2	7.164	2905195	288595	7.339			
Total		39584995	4025446				
